# A comprehensive understanding of carbon–carbon bond formation by alkyne migratory insertion into manganacycles†

**DOI:** 10.1039/d2sc02562k

**Published:** 2022-07-08

**Authors:** L. Anders Hammarback, Jonathan B. Eastwood, Thomas J. Burden, Callum J. Pearce, Ian P. Clark, Michael Towrie, Alan Robinson, Ian J. S. Fairlamb, Jason M. Lynam

**Affiliations:** Department of Chemistry, University of York Heslington York YO10 5DD UK ian.fairlamb@york.ac.uk jason.lynam@york.ac.uk; Central Laser Facility, Research Complex at Harwell, STFC Rutherford Appleton Laboratory, Harwell Campus Didcot Oxfordshire OX11 0QX UK; Syngenta Crop Protection AG Münchwilen Breitenloh 5,4333 Switzerland

## Abstract

Migratory insertion (MI) is one of the most important processes underpinning the transition metal-catalysed formation of C–C and C–X bonds. In this work, a comprehensive model of MI is presented, based on the direct observation of the states involved in the coupling of alkynes with cyclometallated ligands, augmented with insight from computational chemistry. Time-resolved spectroscopy demonstrates that photolysis of complexes [Mn(C^N)(CO)_4_] (C^N = cyclometalated ligand) results in ultra-fast dissociation of a CO ligand. Performing the experiment in a toluene solution of an alkyne results in the initial formation of a solvent complex *fac*-[Mn(C^N)(toluene)(CO)_3_]. Solvent substitution gives an η^2^-alkyne complex *fac*-[Mn(C^N)(η^2^-R^1^C_2_R^2^)(CO)_3_] which undergoes MI of the unsaturated ligand into the Mn–C bond. These data allowed for the dependence of second order rate constants for solvent substitution and first order rate constants for C–C bond formation to be determined. A systematic investigation into the influence of the alkyne and C^N ligand on this process is reported. The experimental data enabled the development of a computational model for the MI reaction which demonstrated that a synergic interaction between the metal and the nascent C–C bond controls both the rate and regiochemical outcome of the reaction. The time-resolved spectroscopic method enabled the observation of a multi-step reaction occurring over 8 orders of magnitude in time, including the formation of solvent complexes, ligand substitution and two sequential C–C bond formation steps.

## Abstract 2

Time-resolved spectroscopy and computational chemistry have informed a unified model of alkyne migratory insertion, an important processes underpinning the transition metal-catalysed formation of C–C and C–X bonds.

## Graphical abstract

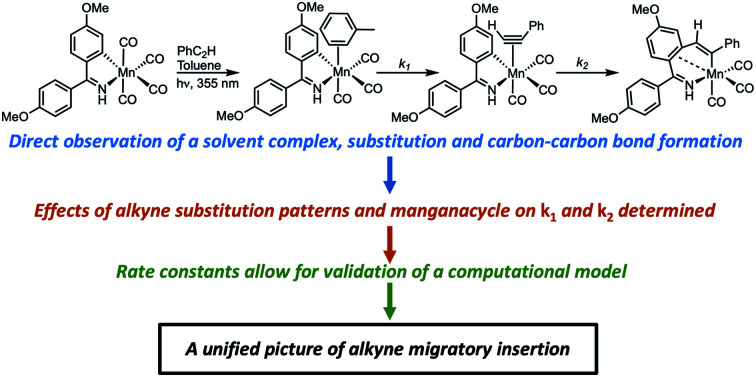

## Introduction

A comprehensive understanding of the mechanistic processes that underpin catalytic reactions is vital to the rational development of new and improved systems with enhanced performance. Such mechanistic insight underpins the discovery of new synthetic transformations leading to atom- and step-efficient routes for structural modification. However, the observation and quantification of the individual microscopic steps across a catalytic reaction coordinate remains deeply problematic as they are frequently fast, entailing that intermediate species are short-lived and difficult to observe in ensemble ‘real’ reaction mixtures.^1^

Migratory insertion (MI) is one of the most important fundamental mechanistic processes in transition metal chemistry and catalysis. The term encompasses many transformations and may be generalised as an intramolecular coupling between an unsaturated L-type ligand (*e.g.* CO, alkene or alkyne) and an X-type ligand^2^ (*e.g.* hydride, alkyl, aryl, alkenyl alkynyl, alkoxide^3–5^ and amido^6^). Specific examples include (1) the combination of carbonyl and hydrocarbyl ligands to give acyl groups, a vital step in the carbonylation of methanol to acetic acid^7^ and the Pauson–Khand reaction^8–10^ (2) reaction between a metal hydride and an alkene as part of a hydrogenation process^11^ and (3) alkene insertion into a metal alkyl complex during polymerisation reactions.^12,13^ Processes such as β-hydride elimination and decarbonylation are the microscopic reverse of MI.^14^

**Fig. 1:**
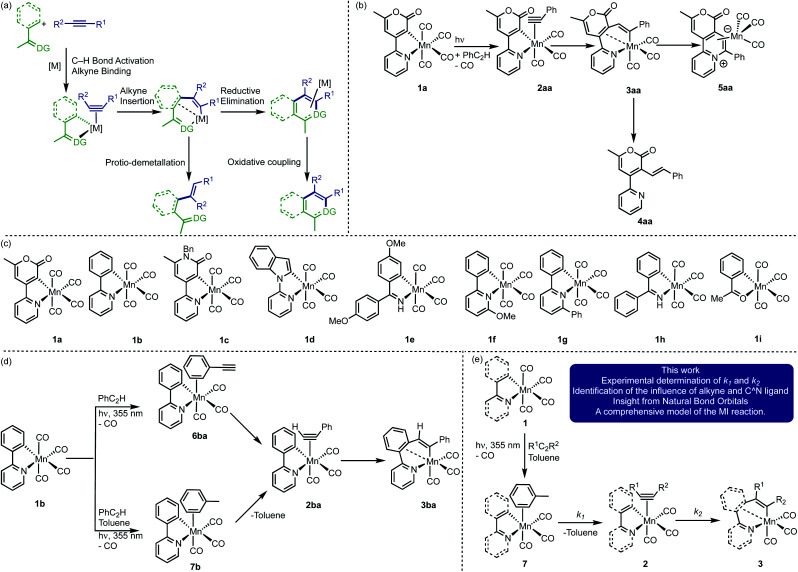
(a) General mechanistic processes for d^6^-metal catalysed C–H bond functionalisation reactions. (b) Previous synthetic work and results from low temperature NMR spectroscopic studies. (c) Complexes used in this study (d) previous insight from TR^M^PS. (e) Insight obtained from this work.

MI processes underpin many of the recent synthetic advances in C–H bond functionalisation reactions. Typically, a proximal heteroatom-containing directing group enables selective C–H bond activation, resulting in the formation of a metallacyclic intermediate (Fig. 1a). A subsequent MI reaction with an unsaturated substrate, such as an alkene or alkyne (sometimes referred to as the acceptor molecule) is responsible for the C–C bond formation step. This results in a ring-expansion reaction to give new metallacycles which are key branching points in the reaction. Depending on the conditions employed and the nature of the catalyst, a range of products from either redox-neutral or oxidative coupling may be obtained (Fig. 1a). There have been significant advances in this area using d^6^-metal pre-catalysts based on Mn(i),^15–18^ Co(iii),^19–23^ Ru(ii)^24–26^ and Rh(iii)^27–30^ scaffolds.

Model studies have provided insight into MI steps within these catalytic cycles. For example, kinetic studies using cyclometallated Co(η^5^-C_5_Me_5_) complexes have revealed that the rate-controlling process involves loss of a ligand to generate a 16-electron species – alkyne coordination and C–C bond formation are therefore fast under these conditions.^31–34^

Photolysis of the cyclomanganated 2-pyrone complex [Mn(2-pyrone)(CO)_4_], 1a, in the presence of PhC_2_H, results in CO-dissociation and formation of the 7-membered manganacycle, 3aa, most likely *via* alkyne complex 2aa (Fig. 1b).^35^ The role of 3aa as a key intermediate in Mn(i)-catalysed reactions was demonstrated by its ability to undergo protio-demetallation to generate 4aa or reductive elimination to form 5aa.

These studies provide important insight into the role of the metallacyclic intermediate, but also demonstrate that many of the intermediates in this process, such as putative alkyne complexes, are short-lived. Although the MI step may not be rate limiting, it plays an important role in controlling the regio-selectivity of the reaction. Therefore, being able to directly observe all the states in an MI reaction between a coordinated alkyne with a metallacyclic intermediate would enable an understanding of the factors that control this step in catalysis and more broadly in applied synthesis.

Time-resolved spectroscopy provides a solution to achieve this goal, circumventing the problems with observing specific intermediates in ensemble mixtures. Light can selectivity trigger pre-catalyst activation and a subsequent spectroscopic probe reveals the interactions between the activated catalyst and reaction components. Therefore, the direct observation of catalytic reaction intermediates and their subsequent fate over a range of timescales is possible.^36–38^ Our recent application of time-resolved multiple probe spectroscopy (TR^M^PS) with infra-red detection to complexes 1a–d (Fig. 1c) demonstrates how this can be applied to study catalytically relevant processes. Photolysis of 1b in neat PhC_2_H results in loss of CO and initial binding of the alkyne through the aryl-substituent to give 6ba (Fig. 1d).^39^ Rearrangement of this complex on a ps timescale then gives 2ba. The MI reaction to form 3ba then occurred on a μs timescale. Experiments between 1b in toluene solutions of PhC_2_H revealed the initial formation of toluene complex, 7b, indicating that, following CO-loss, initial coordination to the Mn occurs in a statistical fashion (toluene is the most dominant species in the experiment). Substitution of the coordinated toluene by PhC_2_H gives 2ba, followed by formation of 3ba.

These findings present an opportunity to quantify all of the components of the MI process. It was reasoned that varying the nature of the cyclomanganated ligand in complexes 1 (Fig. 1c) and the substituents on the alkyne would enable the rate constants for the solvent–substitution reaction, *k*_1_, and the C–C bond formation step, *k*_2_, to be determined (Fig. 1e). When coupled with data from computational chemistry, this would provide unique insight into the factors that control the MI reaction. The successful implementation of this strategy is now reported.

## Results and discussion

### Methodology

In order to gain insight into the factors controlling all of the steps within a MI reaction, the reaction between a range of metallacycles, 1a–i, and alkynes was investigated using TR^M^PS with IR detection. Experiments were performed with a pump wavelength of 355 nm and the changes to the vibrational modes in the metal carbonyl region between *ca.* 1850 and 2100 cm^−1^ recorded. The temporal flexibility of the TR^M^PS experiment was exploited so that processes occurring at pump-probe delays of between 1 ps and 1 ms could be observed.^40,41^

The time-resolved infra-red spectroscopic data are presented as difference spectra with the bands due to species lost on photolysis shown as negative peaks, whereas the subsequent photoproducts are shown as positive features.

### Time-resolved infra-red (TR-IR) studies

#### a. Determination of order of reaction in PhC_2_H and Mn complex

**Fig. 2:**
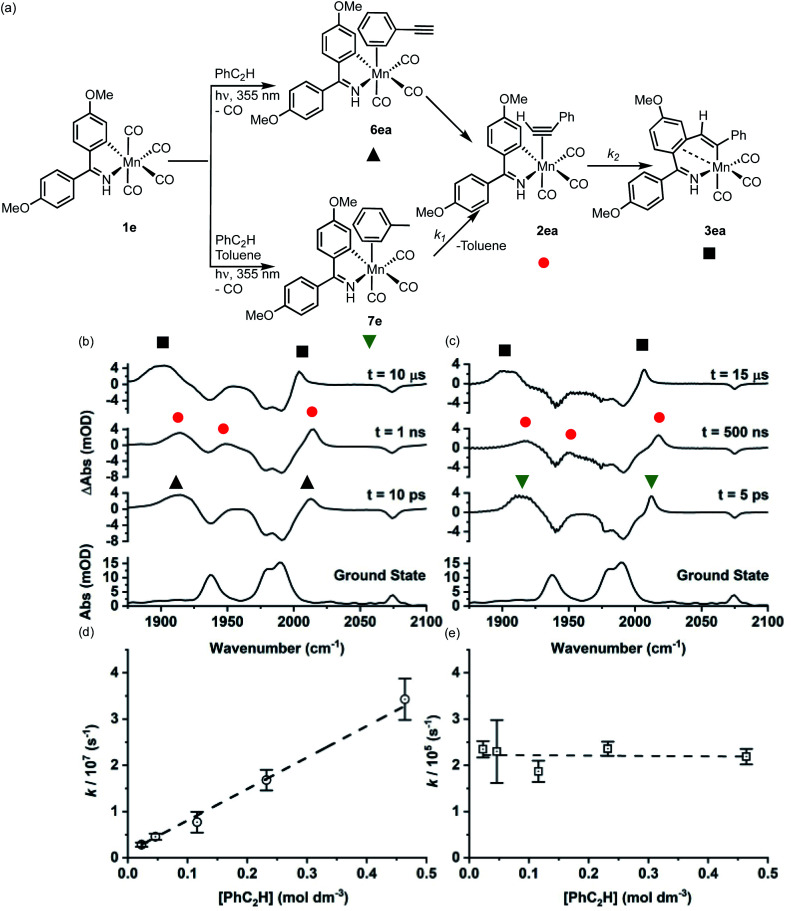
(a) Reaction scheme showing the structure of intermediates formed on photolysis. (b) Bottom ground state IR spectrum of 1e in toluene solution, above TRIR data for 1e in neat PhC_2_H recorded at various pump-probe delays. (c) Bottom ground state IR spectrum of 1e in toluene solution, above TRIR data for 1e in a toluene solution of PhC_2_H recorded at various pump-probe delays. (d) Plot of *k*_1obs_*versus* [PhC_2_H] determined from experiments of 1e in a toluene solution of PhC_2_H, error bars indicate 95% confidence limits for the rate constants. (e) Plot of *k*_2_*versus* [PhC_2_H] determined from experiments of 1e in a toluene solution of PhC_2_H error bars indicate 95% confidence limits for the rate constants.

The photochemically induced reactions of 1b and 1e with PhC_2_H were selected as exemplar systems to quantify the rate constants for the MI reaction and determine the order in Mn and alkyne for each step. Using 1e as an example, photolysis in either neat PhC_2_H or in a toluene solution results in the observation of four negative bands corresponding to the ground state IR spectrum of the Mn(i) carbonyl complex. This confirmed that 1e was lost on irradiation (Fig. 2b and c). In a toluene solution of PhC_2_H (Fig. 2c) new bands were observed at 2012 and 1914 cm^−1^ within 5 ps, corresponding to the formation of 7e. The positions and intensity of the three Mn–CO stretching bands confirming that the complex contains three *fac*-coordinated CO ligands.^42,43^ Identical spectra were observed in an experiment performed in the absence of PhC_2_H, confirming the assignment as the toluene-coordinated complex.

Over the course of *ca.* 1 μs the bands assigned to 7e were replaced by three new peaks at 2017, 1950, 1918 cm^−1^. The shifts to higher wavenumbers are consistent with less π-backbonding to the CO ligands, as expected with the inclusion of an η^2^-bound alkyne (a good π-acceptor) into the coordination sphere of the metal. Therefore, these peaks were assigned to 2ea. The three bands for 2ea were then observed to decrease in intensity over the course of *ca.* 10 μs to be replaced by three new peaks at 2006, 1909, 1899 cm^−1^, which were assigned to metallocycle 3ea (Fig. 2a).

These observations correspond to the light-induced loss of CO from 1e, followed by formation of the solvent complex 7e. Substitution of the Mn-bound toluene by PhC_2_H then affords 2ea and subsequent MI reaction gives 3ea. That 7e is the initially formed product demonstrates that the initial binding event is under kinetic control, with solvent molecules being in excess in the reaction.

Analysis of the temporal behaviour of the reactions as a function of [alkyne] allowed for a kinetic analysis of these mechanistic steps. Experiments were performed under pseudo first-order conditions with a large excess of alkyne present.^44^ Fits to exponential growth and decay functions allowed for observed first order rate constants (*k*_obs_) to be determined as a function of [alkyne] for both transformations 7e → 2ea and 2ea → 3ea. Plots of *k*_obs_*versus* [PhC_2_H] (Fig. 2d and e) allowed for the second order rate constant for 7e → 2ea (*k*_1_) of (7.17 ± 0.26) × 10^7^ mol^−1^ dm^3^ s^−1^ and first order rate constant 2ea → 3ea (*k*_2_) (2.25 ± 0.16) × 10^5^ s^−1^ to be determined. Plots of ln *k*_1obs_*vs.* ln [PhC_2_H] and ln *k*_2_*vs.* ln [PhC_2_H] gave gradients of (0.83 ± 0.04) and (−0.01 ± 0.03), consistent with first and zero-order dependence of each step on the alkyne, respectively (see ESI†).

When the experiment was performed in neat PhC_2_H (Fig. 2b) the initially formed photoproduct corresponded to the arene-bound complex 6ea. Over the course of 100 ps 6ea isomerised to the 2ea and then, on a μs timescale, 3ea was observed to form. Both the band positions of 2ea and 3ea and the value of *k*_2_ were identical within 95% confidence limits to those observed in toluene solution, supporting the intramolecular nature of the MI reaction.

Repeating this series of experiments in a toluene solution of PhC_2_H with 1b as Mn-precursor returned an essentially identical series of observations, with *k*_1_ = (3.74 ± 0.16) × 10^7^ mol^−1^ dm^3^ s^−1^ and *k*_2_ = (1.43 ± 0.03) × 10^5^ s^−1^. These results demonstrate that, compared to the cyclomanganated imine complex, 1e, the rate constant for the substitution of toluene by PhC_2_H and for MI were slower.

#### b. Effects of alkyne substituents and cyclomanganated ligand

A series of experiments were devised to probe the effect of the structure of the alkyne and cyclomanganated ligand on the rates of the solvent substitution and MI reactions. In these experiments an identical concentration of alkyne and manganese complex was used so that the resulting pseudo first order rate constants for the loss of 6/gain of 2, *k*_1(obs)_, were directly comparable between different alkynes and cyclomanganted complexes. As the MI reaction is zero order in alkyne, the first order rate constant for the formation of 3 is equivalent to *k*_2_. The data are collated in Table 1.

**Table 1:** Collated spectroscopic and kinetic parameters

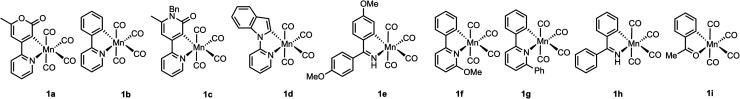						
Complex	Alkyne	*ν*_(CO)_ complex 2/cm^−1^	*ν*_(CO)_ complex 3/cm^−1^	*k*_1(obs)_/10^6^ s^−1^	*k*_2_/10^5^ s^−1^	Experimental Δ*G*^‡^_298_/kJ mol^−1^
1a^a^	PhC_2_H	2015, 1962, 1923	2008, 1928, 1903	Neat	10.4 ± 1.5	39
1b	PhC_2_H	2009, 1944, 1912	2008, 1922, 1899	8.32 ± 3.56	1.43 ± 0.03	44
1b	CyC_2_H	2010, 1944, 1908	2006, 1920, 1890	6.30 ± 0.71	0.13 ± 0.01	50
1b^a^	PhCO_2_CH_2_C_2_H	2012, 1947, 1909	2001, 1903, 1892	Neat	1.79 ± 0.49	43
1b	PhC_2_Ph	2004, 1943, 1912	2003, 1904, 1893	11.95 ± 2.08	0.57 ± 0.06	46
1b	^*n*^BuC_2_^*n*^Bu	2006, 1925, 1906	Not observed	4.36 ± 1.00	Not observed	N/A
1b	CF_3_–4-C_6_H_4_–C_2_H	2014, 1946, 1918	2012 1925, 1905	11.72 ± 3.30	4.86 ± 1.37	41
1b	F–4-C_6_H_4_–C_2_H	2011, 1942, 1915	2010, 1923, 1900	12.07 ± 5.71	4.10 ± 1.74	41
1b	MeO–4-C_6_H_4_–C_2_H	2008,^b^ 1910	2009, 1923, 1898	6.36 ± 3.79	0.91 ± 0.32	45
1b	MeCO_2_–4-C_6_H_4_–C_2_H	2013, 1894	2011, 1923, 1902	9.95 ± 0.41	4.79 ± 2.90	41
1b	Me_2_N–4-C_6_H_4_–C_2_H	2002, 1906, 1884	2007 1921, 1989	5.76 ± 1.33	1.11 ± 0.31	44
1c^a^	PhC_2_H	2011, 1916	2002, 1921, 1898	Neat	74.6 ± 12.8	34
1d^a^	PhC_2_H	2016, 1931, 1922	2009, 1909	Neat	0.20 ± 0.01	48
1e	PhC_2_H	2017, 1950, 1918	2006, 1909, 1899	e	2.25 ± 0.16	43
1e	PhC_2_H	2014, 1948, 1914	2004, 1906, 1895	Neat	2.36 ± 0.15	43
1e^c^	PhC_2_Ph	1997, 1905^b^	1997, 1903, 1893	d	0.24 ± 0.01	42
1e	^*n*^BuC_2_^*n*^Bu	2009, 1932, 1910	Not observed	61.7 ± 1.7	Not observed	N/A
1f	PhC_2_H	2014, 1941, 1906	2012, 1915, 1905	32.5 ± 7.4	1.41 ± 0.05	43
1g	PhC_2_H	2012, 1941, 1908	2012, 1915, 1908	55.3 ± 6.5	1.99 ± 0.13	43
1h	PhC_2_H	2004, 1914^b^	1991, 1903, 1896	18.0 ± 2.8	4.23 ± 0.18	41
1i	PhC_2_H	2023, 1950, 1914	2010, 1906, 1894	5.94 ± 1.01	1.99 ± 0.08	43

a Data from ref. 39.

b One band obscured by bleach.

c Experiment in heptane solution.

d Second order rate constant determined to be (2.89 ± 0.14) × 10^9^ mmol^−1^ dm^3^ s^−1^.

e Second order rate constant determined to be (7.17 ± 1.38) × 10^7^ mol^−1^ dm^3^ s^−1^.

Photolysis of toluene solutions of 1b with alkynes R–4-C_6_H_4_–C_2_H (R = NMe_2_, MeO, H, F, CF_3_, MeCO_2_) all resulted in the initial formation of a toluene adduct 7b. This was followed by substitution of the solvent to give the corresponding alkyne complexes 2b. The vibrational modes of the CO ligands in complexes 2b provided insight into the nature of the metal–ligand bonding in these complexes. The frequency of the high-energy symmetric stretch was observed at higher energy when an electron-withdrawing substituted was present on the alkyne (*e.g.* 2014 cm^−1^ for R = CF_3_) compared to when an electron donating group was used (*e.g.* 2002 cm^−1^ for R = NMe_2_). This is consistent with competition for electron density between the π-acidic alkyne and carbonyl ligands. More electron-rich alkynes are poorer acceptor ligands, resulting in a greater degree of π-backdonation to the Mn-based carbonyl ligands and thus the observed shift to lower energy when R = NMe_2_.

This approach also allowed for the effects of different alkynes on solvent substitution and C–C bond formation to be evaluated. On the whole, the values of *k*_1(obs)_ only showed a small dependence on the nature of the alkyne substrates with the most electron donating substituent being formed at a slower rate than those with electron withdrawing groups. However, *k*_2_ exhibited a much greater variation. For the 4-substituted alkynes, R–4-C_6_H_5_C_2_H, *k*_2_ was greatest when an electron withdrawing group was present *e.g.* (4.86 ± 1.37) × 10^5^ s^−1^ for R = CF_3_, compared to (1.11 ± 0.31) × 10^5^ s^−1^ for R = NMe_2_. The MI reaction of internal alkynes was much slower than their terminal analogues. When compared to PhC_2_H, *k*_2_ was approximately half that when PhC_2_Ph was used and an order of magnitude slower for the cyclohexyl derivative CyC_2_H. Repeating the experiment with ^*n*^BuC_2_^*n*^Bu resulted in a different observation. Following the initial formation of 7b, solvent substitution was observed to give the corresponding alkyne complex as demonstrated by the characteristic bands for this type of complex. However, on the timescale of the experiment (1 ms) no evidence for the subsequent MI reaction was obtained.

These broad trends were also observed when 1e was used as a substrate. When compared to PhC_2_H, the rate constant for MI was considerably slower when PhC_2_Ph was employed: (2.25 ± 0.16) × 10^5^ s^−1^ and (0.24 ± 0.01) × 10^5^ s^−1^ respectively. Due to competition with trace water occurring in toluene, the experiments with PhC_2_Ph were performed in heptane solution (water content ≤ 10 ppm). It should be noted that the rate of solvent substitution in heptane is *ca.* two orders of magnitude faster than for toluene, indicating that the former solvent is more weakly bound to the metal. As with 1d, the corresponding experiment with ^*n*^BuC_2_^*n*^Bu resulted in the formation of an η^2^-alkyne complex but no evidence for the subsequent MI reaction was obtained.

The effect of the nature of the cyclomanganated ligand on the MI was probed further. This ligand may be considered to consist of a heteroatom-based directing group and the metalated carbon atom which undergoes the MI reaction. Across the series of complexes 1e–1i, in which the directing group was modified, the MI reaction with PhC_2_H in toluene solution exhibited only a relatively small variation in *k*_2_. The greatest rate constant was for 1h (4.23 ± 0.18) × 10^5^ s^−1^, and the smallest for 1f (1.41 ± 0.05) × 10^5^ s^−1^. All were similar to those observed for 1b (1.43 ± 0.03) × 10^5^ s^−1^. However, the data on the pyrone, 1a, pyridinone, 1c, and indole, 1d, complexes, in which the migrating group was changed, showed a greater variation in *k*_2_. The greatest value of *k*_2_ was observed for pyridinone-based 1c (*k*_2_ = 74.6 ± 12.8 × 10^5^ s^−1^) and the slowest for indole-substituted 1d (*k*_2_ = 0.20 ± 0.01 × 10^5^ s^−1^), which corresponds to a 373-fold difference in rate constant. These data indicate that, for a given alkyne, it is the primarily the nature of the organic group bound to the manganese which governs the MI reaction and the directing group has little overall effect on this process.

### Computational studies

**Fig. 3:**
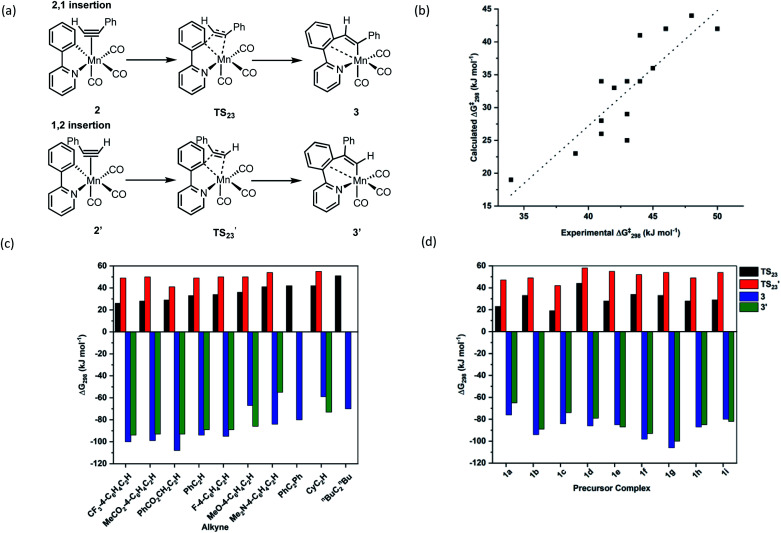
(a) DFT-calculated pathway for C–C bond formation. (b) Correlation between DFT-calculated and experimentally determined free energies of activation for C–C bond formation, the dotted line is a linear least squares regression (*R*^2^ = 0.728). (c) Collated energies showing the effect of different alkynes on the orientation and thermodynamics of insertion into complex 1b. (d) Collated energies showing the effect of different metallacycles on the orientation and thermodynamics of insertion of PhC_2_H.

Further insight into the MI reaction was obtained by studying the states involved using Density Functional Theory (see ESI† for full details of the methodology employed). In these calculations, the alkyne complexes, 2, were taken as the reference state. In the case of unsymmetrically substituted alkynes there are two possible orientations of the alkyne which lead to the MI reaction resulting in 1,2- and 2,1-insertion (Fig. 3a). As the latter process is kinetically preferred (*q.v.*), complexes with the alkyne in the orientation leading to this outcome were used as the reference state.

The MI reactions were modelled for all of the combinations of alkyne and cyclomanganated ligands studied experimentally, for both the 1,2- and 2,1-insertion pathways. In each case a transition state for MI (TS_23_) was located which, through a dynamic reaction coordinate analysis, was shown to link alkyne complex 2 and metallacycle 3 (Fig. 3a). It was also possible to correlate the experimentally and computationally determined free energies of activation for the insertion reaction. In this case, the experimentally determined first order rate constants (*k*_2_) were converted to Gibbs energies using the Eyring equation. The resulting plot (Fig. 3b) showed a good correlation between the two approaches, demonstrating the computational method was a competent and viable model for this reaction. In addition, the predicted barrier to insertion for ^*n*^BuC_2_^*n*^Bu was the highest of those calculated, consistent with the fact that no MI reaction was observed in the TRIR experiments with this alkyne.

This analysis demonstrates this is an excellent system to model through computational chemistry as TR^M^PS has enabled the first order rate constants for the exact elemental step under consideration to be determined.

The calculations provided insight into effects of the different substituents on the rate of the insertion reaction, the orientation of the insertion and ultimately the nature of the C–C bond formation step.

Although the two orientations of the alkyne complex 2 and 2′ were generally found to be at essentially identical energies, the transition states for the 2,1-insertion (black bars, Fig. 3c and d) are found to be uniformly at lower energy than the corresponding 1,2-insertion (red bars). In most cases, the orientation of insertion does not significantly affect the overall thermodynamic change of the insertion process and therefore we propose that the regiochemical outcome of the reaction is kinetically controlled.

**Fig. 4:**
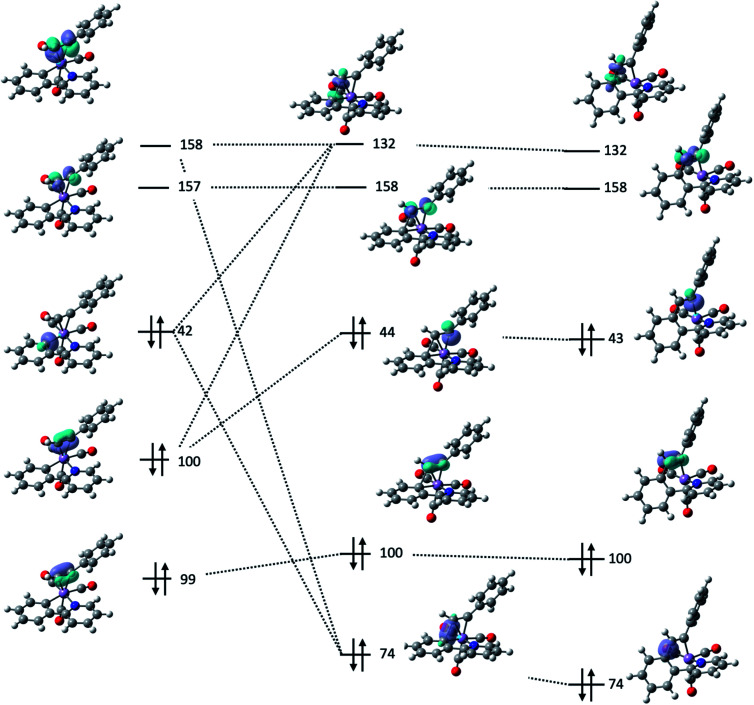
Key NBOs involved in the migratory insertion process for 2ba (left) TS_23ba_ (centre) and 3ba (right).

Further insight into the nature of the MI reaction was obtained by analysis of the electronic structure of states 2, TS_23_ and 3. An evaluation of the canonical molecular orbitals obtained at the D3-pbe0/def2-TZVPP level of theory revealed that several MOs were involved in C–C bond formation step. Therefore, in order to obtain a chemically intuitive view of the MI reaction, the electronic structure of each state was modelled using the Natural Bond Orbital (NBO) approach.^45^ The relevant NBOs involved in the C–C bond formation step for complexes 2ba, TS_23ba_ and 3ba are shown in Fig. 4. The NBOs of the alkyne ligand are π-bonding orbitals 99, 100 and their antibonding counterparts 157 and 158 which all have >90% p-character. Of the two orthogonal sets, orbitals 100 and 158 are directly involved in bonding to the metal, with 100 showing a signification second order perturbation stabilisation (Δ*E*_ij_^(2)^ = 73.59 kcal mol^−1^) reflecting electron donation to a vacant metal-orbital. There is corresponding resonance stabilisation from a filled Mn d-orbital to the antibonding π*-orbital 158 (Δ*E*_ij_^(2)^ = 24.09 kcal mol^−1^) which can be viewed as backdonation in the Dewar–Chatt–Duncanson model. Orbitals 99 and 157 do not show any significant interaction with metal-based orbitals, indicating that the alkyne is best viewed as a formal two-electron donor, consistent with an 18-electron count. The Mn–C bond involved in the MI reaction is described as a carbon-based lone pair (NBO 42), with *ca.* 26% s and 74% p character. The donor interaction to the metal is modelled through resonance stabilisation to an empty M–L anti-bonding orbital (Δ*E*_ij_^(2)^ = 131.18 kcal mol^−1^).

**Fig. 5:**
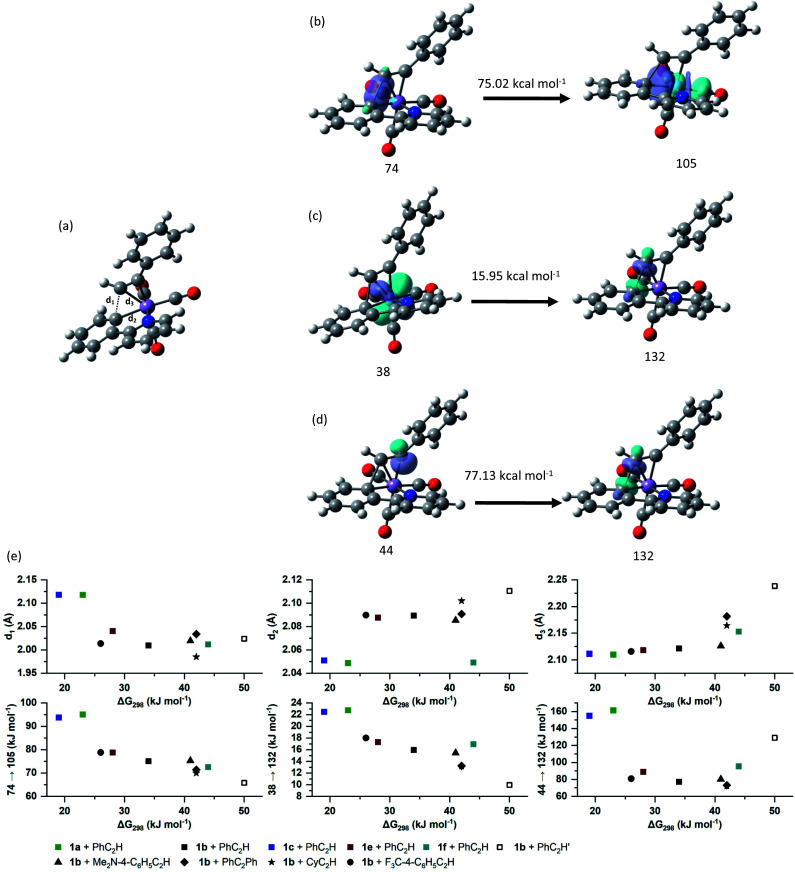
(a) Structure of TS_23ba_. (b)–(d) Selected donor–acceptor interactions in TS_23ba_. The energies given are the second order perturbation energies associated with each interaction. (e) Correlation of computationally derived parameters in TS_23_ with the free energy of activation for the alkyne insertion.

Analysis of the NBOs in TS_23ba_ revealed the key interactions controlling the C–C bond formation. NBO 74 describes the bonding component of the nascent C–C bond: it has an occupancy of 1.65 electrons and *ca.* 27% s and 73% p character. Examination of the second order perturbation analysis reveals the nature of the metal-assistance in the C–C bond step as NBO 74 acts a donor to a vacant metal orbital (NBO 105, Δ*E*_ij_^(2)^ = 75.02 kcal mol^−1^, Fig. 5b). This is complemented by backdonation to the corresponding C–C antibonding orbital (NBO 132). This occurs from a filled metal d-orbital (NBO 38) (Δ*E*_ij_^(2)^ = 15.95 kcal mol^−1^, Fig. 5c) and a hyperconjugative interaction between NBO 132 and the newly-formed Mn–C bond, NBO 44, (Δ*E*_ij_^(2)^ = 77.13 kcal mol^−1^, Fig. 5d). Commensurate with these interactions, the formally C–C antibonding orbital has a population of 0.34 electrons.

Analysis of the NBOs for 3ba demonstrates that the newly formed C–C bond is composed of a filled bonding orbital (NBO 74, occupancy 1.97 electrons) comprised of overlap between two sp^2^-hybridised carbon atoms. The corresponding anti-bonding orbital NBO 132 is essentially vacant (occupancy 0.02 electrons). The newly formed Mn–C bond is described by NBO 43 which is a carbon-based lone pair that is resonance-stabilised by donation to a vacant metal orbital (Δ*E*_ij_^(2)^ = 146.6 kcal mol^−1^). MI reactions result in a formal decrease in electron count (in this case from 18- to 16-electron) and it has been proposed that weak donor interactions between the newly formed metallacycle and the metal help to stabilise the unsaturated nature of the metal.^35^ The NBO calculations provide further evidence for this interaction with a small resonance stabilisation between a π-bond on the arene ring and a vacant metal–ligand anti-bonding orbital (Δ*E*_ij_^(2)^ = 11.89 kcal mol^−1^).

Analysis of these NBOs and how they change during the C–C bond formation step provides important mechanistic insight. The formation of TS_23ba_ is probably best viewed as a combination of NBO 42 (a carbon-based lone pair) with NBO 158 (π*-orbital on the alkyne). Concurrent with this, the formation of NBO 44, which is the carbon-based lone pair responsible for the new Mn–C bond, may be viewed as being derived from NBO 100 (filled p-orbital on the alkyne). Therefore, the two alkyne orbitals engaged in synergic bonding with the Mn are also those involved in the MI process. To provide effective overlap between NBO 42 and NBO 158, it is evident that the alkyne must be aligned with the Mn–C bond in the 2-phenylpyridine, as is observed in both structure 2ba and transition state structure TS_23ba_.

The NBO calculations provided insight into how the structural changes to the alkyne and metallacycle within this series of compounds affected the MI reaction. An analysis of the calculated NBOs in states 2, TS_23_ and 3 across a range of complexes studied in this work demonstrated that the different substitution patterns only had a notable effect on the donor/acceptor interactions in TS_23_.

Fig. 5e shows the correlation between the calculated Gibbs energy of activation for the MI reaction based on individual alkyne/metallacycle combinations and the Δ*E*_ij_^(2)^ values for the three donor/acceptor interactions in Fig. 5b–d. Correlations between the bond metrics in the transition state with the Gibbs energy of activation are also shown. These data provide insight into the critical factors controlling the MI reaction. As detailed above, the 2,1-insertion of the alkyne is kinetically favoured over the 1,2-insertion. In the 1,2-transition state the orientation of the alkyne results in longer Mn–C bonds to the alkyne and may be interpreted as greater steric repulsion in the 1,2-insertion transition state. However, the NBO analysis shows that these elongated distances correlate with weaker donor/acceptor interactions between the nascent C–C bond and the metal (black squares *versus* open squares in Fig. 5e). Although this is slightly compensated by a greater C–C hyperconjugative interaction, it is postulated that the strength of these donor/acceptor interactions is the key factor controlling the rate of the MI reaction.

This is further illustrated by comparing the data for PhC_2_Ph and CyC_2_H. Again, the Mn-alkyne distance are longer than in the PhC_2_H case, which results in weaker donor/acceptor interactions. However, for the 2-pyrone and 2-pyridinone manganacycles which show the fastest rates of MI reaction, then the opposite effect is observed, the alkyne and Mn–C bonds are notably shorter and the donor–acceptor interactions much stronger. Commensurate with this model, the nascent C–C bond is longer in these cases.

### Observation of the fate of manganacycle 3ia

**Fig. 6:**
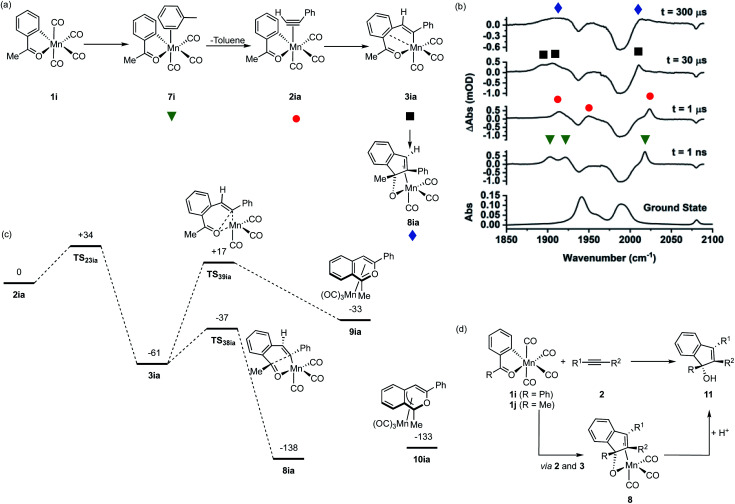
(a) Reaction scheme showing the structure of intermediates formed on photolysis of 1i in a toluene solution of PhC_2_H. (b) Bottom ground state IR spectrum of 1i in toluene solution, above TRIR data for 1i in a toluene solution of PhC_2_H recorded at various pump–probe delays. (c) DFT-calculated pathways for Mn-promoted C–C bond formation. Energies are Free Energies at 298 K in kJ mol^−1^ at the D3-pbe0/def2-TZVPP//bp86/SV(P) level with COSMO solvation in toluene. (d) Previously reported Mn-promoted formation of 1*H*-inden-1-ols (top) and the role of intermediate 8 (bottom).

As shown in Fig. 1a, manganacycles such as 3 have several potential fates which underpins the synthetic versatility of C–H bond activation reactions promoted by d^6^-metal complexes. In the case of complex 3ia, which is derived from acetophenone, the temporal flexibility of the TR^M^PS experiment enabled the direct observation of one of these pathways following the MI reaction. Photolysis of 1i in a toluene solution of PhC_2_H resulted in the expected formation of toluene complex 7i (Fig. 6a), followed by alkyne substitution and insertion to give 2ia and 3ia respectively. However, in contrast to the other systems investigated in this study, the peaks in the TRIR spectra for 3ia were found to subsequently decrease in intensity to be replaced by a new band at 2013 cm^−1^ and a broad feature centred at 1912 cm^−1^ (*k*_obs_ = 1.02 ± 0.03 × 10^4^ s^−1^) which were assigned to 8ia (Fig. 6b).

The stoichiometric reactions of manganacycles containing ketone-based directing groups may give two different potential outcomes. In the first instance, a formal reductive elimination reaction to give a six-membered ring may occur. In the case of cyclomanganated chalcone derivates this results in the formation of pyranyl complexes.^46,47^ This process is analogous to the catalytic oxidative coupling of alkynes with heterocycles and the related formation of 5aa (Fig. 1b).^35^ Alternatively, manganated aromatic ketones have been shown to react with alkynes to give 1*H*-inden-1-ols (Fig. 6d).^48,49^ A key step in such a process would be the formation of a five-membered ring and it was envisaged that could occur from 3ia through nucleophilic attack of the Mn-coordinated carbon atom onto the carbonyl group of the ketone.^46,47^ Both of these potential pathways were successfully modelled by DFT (Fig. 6c). In the case of the first pathway, which would afford a six-membered ring, the transition state for C–O bond formation (TS_39ia_) was located at +17 kJ mol^−1^ with respect to the alkyne complex 2ia which was taken as the reference state. This corresponds to an energic span of 78 kJ mol^−1^ from 3ia. The transition state for the formation of the five-membered ring, TS_38ia_ was located at much lower energy (−37 kJ mol^−1^), a barrier of 24 kJ mol^−1^ from 3ia.

Complex 8ia was assigned as the indenoate complex shown in Fig. 6a. This is on the basis that TS_38ia_ is at significantly lower energy than TS_39ia_ and the predicted barrier is 24 kJ mol^−1^. The predicted scaled IR bands for the metal carbonyl groups predict the experimentally observed blue shift on changing from 3ia to 8ia. In the case of the putative formation of 10ia, a red shift was predicted (see ESI†) which, taken with the larger predicted barrier for its formation, excludes assignment to this complex. Additionally, calculations on the related systems which have been shown experimentally to yield the pyranyl complexes indicated that the alternative pathway would be expected (see ESI†).

The observations from this experiment are therefore assigned to the key C–C bond formation steps that underpin the Mn-promoted formation of 1*H*-inden-1-oles (Fig. 6d). Light-induced CO loss from 1i, is followed by solvent coordination to give 7i; solvent substitution by the alkyne (7i → 2ia, ns timescale) is followed by insertion (2ai → 3ai 10 μs timescale), with final formation of the five-membered ring (3ia → 8ia 100 μs timescale). In the synthetic work, it is then expected that protonation of 8ia affords the 1*H*-inden-1-ol (shown as 11 in Fig. 6d).

More widely, these results demonstrate the potential of TR^M^PS to enable the direct observation of bond-formation steps occurring over several orders of magnitude in time.

## Conclusions

The data from this study indicate that the rate and selectivity of the migratory insertion reactions of alkynes into Mn–C bonds is directly controlled by the extent of synergic interactions involving the nascent C–C σ-bond. These interactions are analogous to the more well-known donor/acceptor bonding that underpins the metal-promoted oxidative addition activation pathway of H_2_, C–H and related element-hydrogen bonds and it is envisaged that parallel arguments about how metal–ligand interactions influence these reactions can be made in this case.

Previous experimental and computational work have rationalised the regioselectivity of the MI process in terms of both steric^50–52^ and/or electronic factors (either orbital coefficients on the alkyne,^53–55^ or the relative electron rich/poor nature of the alkyne carbons).^56,57^ The steric influence of spectator ligands on the MI process has also been highlighted.^58^

It is proposed that the model presented in this work provides a framework to harmonise all of these arguments. For example, as shown in the case of PhC_2_H *vs.* CyC_2_H in the current study, an increase in bulk of the alkyne results in a slower rate of MI. This is rationalised on the basis of increased metal–ligand bond lengths in the transition states of the more crowded cases and a commensurate decrease in the synergic interaction involving the nascent C–C σ-bond. Electronic factors are also consistent with this model. For example, a more electron deficient alkyne would be expected to increase π-backbonding to the C–C σ-bond accelerating the rate of reaction (as also observed experimentally). In addition, the carbon atom of the alkyne with the greatest orbital coefficient would enable greater metal–ligand interactions in the transition state, again enhancing this synergic interaction.

It is also informative to place the results from this study within the context of catalytic reactions that have been proposed to involve the insertion of an alkyne into a manganacycle. As many of these processes proceed at temperatures >100 °C, then the MI step which occurs on a μs timescale is unlikely to be rate controlling. However, our data support the proposition that the regiochemical outcome (1,2 *vs.* 2,1 alkyne insertion) is kinetically controlled through TS_23_. A survey of a number of reactions^38,59–64^ which relay on this MI reaction show that for terminal alkynes 2,1 insertion is universally observed.^65^ This is consistent with the proposed model as the transition state for 1,2-insertion has weaker Mn–C synergic interactions due to a longer *d*_3_ distance (Fig. 5). Examples with unsymmetric internal alkynes only show significant selectivity on insertion when there is a profound difference in substitution pattern (*e.g.*, with MeC_2_Ph or EtC_2_Ph).^62,63^ In these examples, 2,1 insertion dominates presumably due to the same geometric factors which control the insertion of terminal alkynes predominating, enhancing the orbital interactions in TS_23_.

The approach described in this paper also highlights how spectroscopic measurements can be integrated with computational chemistry to provide unique and important insight into catalytic reaction mechanism. Directly observing processes occurring across a wide range of timescales ensures that previously inaccessible mechanistic insight becomes available.

## Data availability

The dataset associated with this article is available at https://doi.org/10.15124/8ccc891d-15a5-4a79-986b-6d20b3777d59.

## Author contributions

JML and IJSF conceived the experimental programme with input on project direction from AR. The TRIR experiments were performed by LAH, JML, IJSF, JBE, TJB and IPC on instrumentation set-up and built by MT. Manganese compounds were prepared and analysed by LAH, JBE and TJB; JML and CJP performed and analysed the DFT calculations. TRIR data were analysed by JML, LAH, IJSF, JBE, TJB, IPC and MT. JML wrote the paper with input from all authors.

## Conflicts of interest

There are no conflicts to declare.

## Supplementary Material

SC-013-D2SC02562K-s001

SC-013-D2SC02562K-s002
