# Supplementary material for: A comprehensive understanding of carbon–carbon bond formation by alkyne migratory insertion into manganacycles
Source: Chem Sci. 2022 Jul 8;13(34):9902–13. doi: 10.1039/d2sc02562k (PMC9431456; doi:10.1039/d2sc02562k)
Supplement: SC-013-D2SC02562K-s001 [file SC-013-D2SC02562K-s001.pdf]

Electronic Supplementary Material (ESI) for Chemical Science.
This journal is © The Royal Society of Chemistry 2022

Calculated coordinates, energies and vibrational frequencies for the computational chemistry in:

A comprehensive understanding of carbon-carbon bond formation through alkyne migratory insertion into manganacycles.

L. Anders Hammarback,[a] Jonathan B. Eastwood,[a] Thomas J. Burden,[a] Callum J. Pearce,[a] Ian P. Clark,[b] Michael Towrie,[b] Alan Robinson,[c] Ian J. S. Fairlamb*[a] and Jason M. Lynam*[a]

[a] Department of Chemistry, University of York, Heslington, York, YO10 5DD, UK ian.fairlamb@york.ac.uk, jason.lynam@york.ac.uk

[b] Central Laser Facility, STFC Rutherford Appleton Laboratory, Harwell Science and Innovation Campus, Didcot, Oxfordshire, OX11 0QX, UK.

[c] Syngenta Crop Protection AG, Münchwilen, Breitenloh 5, 433, Switzerland.

2aa

```
SCF Energy (au) BP86/SV(P)                    -2427.867667755
SCF Energy (au) PBE0/def2-TZVPP               -2427.429781153
SCF Energy (au) PBE0/def2-TZVPP               -2427.4405898830 (Toluene Correction)
Zero Point Energy (au)                        0.2962891
Chemical Potential (kJ mol-1)                 622.29
Dispersion Correction (au) PBE0/def2-TZVPP   -0.06324694
```

```
xyz coordinates
43

Mn    1.4645116    0.2343712    0.5909323
C     1.7816619    0.4425193    2.3507023
C     3.1502517   -0.3752202    0.3332030
C     1.9986404    1.9297007    0.2215559
O     2.3604200    3.0246807    0.0388304
O     4.2519496   -0.6744970    0.1185508
O     1.9860597    0.5565279    3.4908732
C    -1.3688135   -0.1875101    1.3527343
C    -1.0731050    1.9759850    0.4993644
C    -2.4254157    2.2929513    0.6800968
C    -3.2774363    1.3187041    1.2262739
C    -2.7416292    0.0711722    1.5626743
C    -0.6964351   -1.4521885    1.6603519
C     0.6639318   -1.4851738    1.3612258
C     1.4083399   -2.7154182    1.6139593
O     0.6707753   -3.7894906    2.2061021
C    -0.6513101   -3.7241982    2.4956329
C    -1.3727373   -2.5906643    2.2321973
H    -0.3769659    2.7177069    0.0793093
H    -2.7928098    3.2910274    0.3962989
H    -4.3469456    1.5296005    1.3888023
H    -3.3841721   -0.7119417    1.9907119
O     2.5769801   -2.9477696    1.3720105
C    -1.1727391   -4.9896275    3.1068736
H    -2.4446006   -2.5726096    2.4751610
N    -0.5455730    0.7755408    0.8231995
C     0.8586201    1.2727425   -2.6141879
C     1.9732273    2.0182441   -3.0776447
C     1.8089943    2.9990900   -4.0649810
C     0.5359603    3.2632459   -4.6022414
C    -0.5765511    2.5332325   -4.1481913
C    -0.4219365    1.5470344   -3.1628167
H     2.9715633    1.8130324   -2.6613145
H     2.6864145    3.5651591   -4.4186222
H     0.4115198    4.0380631   -5.3765206
H    -1.5768983    2.7310968   -4.5679957
H    -1.2907383    0.9686119   -2.8108704
C     1.0036831    0.2255046   -1.6318907
C     0.9714273   -0.9282526   -1.1151016
H     0.8955204   -2.0110916   -1.2178750
H    -1.0093753   -5.8521617    2.4222750
H    -2.2559519   -4.9078343    3.3298297
H    -0.6283127   -5.2198957    4.0505213
```

```
$vibrational spectrum
#  mode     symmetry     wave number   IR intensity    selection rules
#                         cm**(-1)        km/mol         IR     RAMAN
     1                      0.00          0.00000         -       -
     2                      0.00          0.00000         -       -
     3                      0.00          0.00000         -       -
     4                      0.00          0.00000         -       -
     5                      0.00          0.00000         -       -
```

6		0.00	0.00000	-	-
7	a	12.83	0.33682	YES	YES
8	a	28.85	0.53951	YES	YES
9	a	32.31	0.18001	YES	YES
10	a	37.53	1.95531	YES	YES
11	a	60.15	0.05712	YES	YES
12	a	69.85	0.11556	YES	YES
13	a	75.55	0.07607	YES	YES
14	a	81.69	0.39008	YES	YES
15	a	91.73	1.64917	YES	YES
16	a	99.05	0.97666	YES	YES
17	a	109.88	0.12991	YES	YES
18	a	119.87	0.10005	YES	YES
19	a	123.49	1.49253	YES	YES
20	a	137.69	0.14153	YES	YES
21	a	148.69	1.92865	YES	YES
22	a	153.79	1.84421	YES	YES
23	a	159.65	1.34880	YES	YES
24	a	163.44	2.66553	YES	YES
25	a	192.46	1.04417	YES	YES
26	a	219.28	0.63073	YES	YES
27	a	249.36	0.36492	YES	YES
28	a	257.61	1.89803	YES	YES
29	a	294.76	2.37808	YES	YES
30	a	302.79	4.27708	YES	YES
31	a	322.17	10.76010	YES	YES
32	a	330.22	13.32318	YES	YES
33	a	398.32	4.09474	YES	YES
34	a	401.54	0.07874	YES	YES
35	a	414.56	12.49803	YES	YES
36	a	428.96	1.22830	YES	YES
37	a	455.65	0.76864	YES	YES
38	a	457.06	9.03126	YES	YES
39	a	467.19	5.31735	YES	YES
40	a	479.14	10.45341	YES	YES
41	a	486.43	4.00283	YES	YES
42	a	493.29	15.20698	YES	YES
43	a	500.80	11.90031	YES	YES
44	a	521.26	39.87505	YES	YES
45	a	532.31	6.17382	YES	YES
46	a	537.42	1.03599	YES	YES
47	a	544.56	5.83322	YES	YES
48	a	561.68	1.89963	YES	YES
49	a	581.56	3.53684	YES	YES
50	a	590.09	1.38142	YES	YES

2aa′

SCF Energy (au) BP86/SV(P)	-2427.867511092
SCF Energy (au) PBE0/def2-TZVPP	-2427.429306722
SCF Energy (au) PBE0/def2-TZVPP	-2427.4398029146 (Toluene Correction)
Zero Point Energy (au)	0.2965857
Chemical Potential (kJ mol^{-1})	627.02
Dispersion Correction (au) PBE0/def2-TZVPP	-0.06502154

xyz coordinates
43

Mn	1.5147523	1.8031815	0.1393490
C	1.7127939	2.1387468	1.9018085
C	3.2493306	1.2886291	-0.0008400
C	1.9281023	3.5153518	-0.2748048
O	2.1937308	4.6255178	-0.5219659
O	4.3733880	1.0453489	-0.1588772
O	1.8492529	2.3558813	3.0367268
C	-1.3079859	1.1705764	0.7982681
C	-1.1705494	3.2587826	-0.2561876
C	-2.5571983	3.4451580	-0.1964264
C	-3.3444393	2.4413384	0.3921436
C	-2.7119508	1.2979551	0.8908193
C	-0.5340596	0.0226442	1.2788665
C	0.8392402	0.0971972	1.0533379
C	1.6869353	-0.9837612	1.5445888
O	1.0171909	-2.0733219	2.1814486
C	-0.3230854	-2.1275936	2.3677577
C	-1.1327676	-1.1103397	1.9381859
H	-0.5218856	4.0264367	-0.7056722
H	-3.0024165	4.3659229	-0.6039621
H	-4.4391991	2.5486993	0.4604218
H	-3.3026868	0.4929847	1.3514589
O	2.8988123	-1.0795146	1.4755477
C	-0.7602480	-3.3773999	3.0697511
H	-2.2161667	-1.1829428	2.1088455
N	-0.5509876	2.1614445	0.2266401
H	1.5437899	2.6770580	-2.7278962
C	1.3490132	1.8945099	-1.9927118
C	1.1035637	0.6840225	-1.6983683
C	0.7228759	-0.6759224	-2.0148629
H	-0.4580107	-4.2776405	2.4886854
H	-1.8604484	-3.3925945	3.2075539
H	-0.2712121	-3.4569603	4.0667827
C	-0.0173112	-3.3078536	-2.7473764
C	-0.8258252	-2.2191517	-3.1181057
C	-0.4617625	-0.9130270	-2.7582148
C	1.5263381	-1.7809469	-1.6370200
C	1.1589866	-3.0817693	-2.0096625
H	-0.3036529	-4.3333910	-3.0337641
H	-1.7490587	-2.3869893	-3.6976299
H	-1.0910542	-0.0579195	-3.0525480
H	2.4456298	-1.6090748	-1.0558656
H	1.8002357	-3.9292733	-1.7162254

$vibrational spectrum

# mode	symmetry	wave number	IR intensity	selection rules	
#		cm**(-1)	km/mol	IR	RAMAN
1		0.00	0.00000	-	-
2		0.00	0.00000	-	-
3		0.00	0.00000	-	-
4		0.00	0.00000	-	-
5		0.00	0.00000	-	-

6		0.00	0.00000	-	-
7	a	24.31	0.19772	YES	YES
8	a	26.79	0.77570	YES	YES
9	a	44.61	0.64194	YES	YES
10	a	50.58	0.51026	YES	YES
11	a	66.00	0.33144	YES	YES
12	a	72.98	0.69452	YES	YES
13	a	77.43	0.43971	YES	YES
14	a	83.41	0.66789	YES	YES
15	a	95.16	0.95452	YES	YES
16	a	100.70	0.38104	YES	YES
17	a	106.25	0.32277	YES	YES
18	a	124.35	0.25320	YES	YES
19	a	127.92	0.85768	YES	YES
20	a	138.50	0.05127	YES	YES
21	a	150.94	0.32827	YES	YES
22	a	158.78	1.28351	YES	YES
23	a	166.59	0.35274	YES	YES
24	a	180.24	1.66707	YES	YES
25	a	188.29	2.27493	YES	YES
26	a	212.55	0.37909	YES	YES
27	a	247.14	0.72321	YES	YES
28	a	260.96	0.15337	YES	YES
29	a	294.50	3.08395	YES	YES
30	a	308.25	8.36217	YES	YES
31	a	326.79	2.69630	YES	YES
32	a	337.65	8.35710	YES	YES
33	a	398.14	3.36635	YES	YES
34	a	403.18	2.34038	YES	YES
35	a	408.53	10.62109	YES	YES
36	a	428.45	10.50586	YES	YES
37	a	457.68	0.58035	YES	YES
38	a	460.91	0.98099	YES	YES
39	a	468.88	4.53489	YES	YES
40	a	479.50	3.90853	YES	YES
41	a	484.74	7.08867	YES	YES
42	a	493.18	4.58799	YES	YES
43	a	506.36	23.83062	YES	YES
44	a	525.23	14.38684	YES	YES
45	a	535.01	3.26780	YES	YES
46	a	541.18	0.57910	YES	YES
47	a	559.77	3.82363	YES	YES
48	a	564.37	1.56700	YES	YES
49	a	583.10	1.77315	YES	YES
50	a	593.14	1.93748	YES	YES

2ba

```
SCF Energy (au)BP86/SV(P)                     -2277.516190056
SCF Energy (au)PBE0/def2-TZVPP                -2277.070640511
SCF Energy (au)PBE0/def2-TZVPP                -2277.0778650907 (Toluene Correction)
Zero Point Energy (au)                        0.2897773
Chemical Potential (kJ mol-1)                 615.66
Dispersion Correction (au) PBE0/def2-TZVPP  -0.06110662
```

xyz coordinates
41

```
Mn    1.3218603    0.2161020    0.6121444
C     1.7788652    0.6991612    2.2861199
C     3.0068573   -0.2730204    0.2487755
C     1.5913757    1.8997864   -0.0160227
O     1.8059907    2.9928522   -0.3644069
O     4.1007668   -0.6058230    0.0151684
O     2.0820196    1.0031429    3.3682155
C    -1.3180498   -0.4492854    1.7886866
C    -1.4278286    1.5987142    0.6433287
C    -2.7843206    1.7644718    0.9428058
C    -3.4261769    0.7711469    1.7027561
C    -2.6879074   -0.3376577    2.1237114
C    -0.4446517   -1.5624103    2.1751019
C     0.9072958   -1.4767290    1.7297269
C     1.7860999   -2.5181232    2.0955425
C     1.3438020   -3.6150948    2.8558307
C     0.0029626   -3.6950653    3.2795373
C    -0.8868254   -2.6697164    2.9404323
H    -0.8883399    2.3555919    0.0531607
H    -3.3179336    2.6588013    0.5861152
H    -4.4929401    0.8621691    1.9652861
H    -3.1679721   -1.1282940    2.7185786
H     2.8433831   -2.4844404    1.7833064
H     2.0545812   -4.4155643    3.1237603
H    -0.3442870   -4.5537096    3.8769362
H    -1.9343081   -2.7352412    3.2784777
N    -0.7068340    0.5308017    1.0490580
C     0.4186177    0.7604854   -2.6526220
C     1.4639894    1.4883082   -3.2758567
C     1.2035674    2.2810818   -4.4019640
C    -0.1001745    2.3746674   -4.9216796
C    -1.1459588    1.6622080   -4.3088994
C    -0.8936096    0.8615517   -3.1851924
H     2.4862082    1.4183505   -2.8726273
H     2.0294261    2.8347075   -4.8783822
H    -0.3004026    3.0034950   -5.8047658
H    -2.1704367    1.7271619   -4.7118877
H    -1.7103084    0.2966645   -2.7081434
C     0.6611091   -0.1065099   -1.5221846
C     0.6813710   -1.1757627   -0.8449443
H     0.5791166   -2.2589761   -0.7729843
```

```
$vibrational spectrum
#  mode     symmetry     wave number   IR intensity    selection rules
#                         cm**(-1)        km/mol          IR     RAMAN
     1                        0.00        0.00000         -        -
     2                        0.00        0.00000         -        -
     3                        0.00        0.00000         -        -
     4                        0.00        0.00000         -        -
     5                        0.00        0.00000         -        -
     6                        0.00        0.00000         -        -
     7        a              18.14        0.20009        YES      YES
```

8	a	29.04	0.15401	YES	YES
9	a	39.82	0.05836	YES	YES
10	a	57.15	0.31155	YES	YES
11	a	69.46	0.07269	YES	YES
12	a	74.18	0.09666	YES	YES
13	a	86.23	1.28775	YES	YES
14	a	90.54	0.26084	YES	YES
15	a	99.10	0.43721	YES	YES
16	a	109.57	0.13096	YES	YES
17	a	113.25	0.10437	YES	YES
18	a	117.38	0.59846	YES	YES
19	a	130.55	0.29161	YES	YES
20	a	162.17	1.81464	YES	YES
21	a	165.28	5.13092	YES	YES
22	a	188.98	1.29120	YES	YES
23	a	222.35	0.64998	YES	YES
24	a	242.82	2.12591	YES	YES
25	a	262.80	0.48548	YES	YES
26	a	282.79	1.96336	YES	YES
27	a	319.03	24.25280	YES	YES
28	a	358.43	1.93112	YES	YES
29	a	400.88	0.01385	YES	YES
30	a	410.80	1.69648	YES	YES
31	a	420.57	10.80509	YES	YES
32	a	437.44	2.27242	YES	YES
33	a	454.64	16.81944	YES	YES
34	a	456.28	1.42207	YES	YES
35	a	472.05	4.20216	YES	YES
36	a	480.46	6.23593	YES	YES
37	a	486.64	1.87109	YES	YES
38	a	492.13	5.61083	YES	YES
39	a	502.05	7.64352	YES	YES
40	a	508.50	13.02943	YES	YES
41	a	524.55	36.11294	YES	YES
42	a	539.45	10.10604	YES	YES
43	a	551.07	5.77890	YES	YES
44	a	557.04	1.74908	YES	YES
45	a	609.09	21.17504	YES	YES
46	a	614.47	7.83987	YES	YES
47	a	623.39	7.80591	YES	YES
48	a	625.85	11.54609	YES	YES
49	a	643.05	9.93463	YES	YES
50	a	653.95	72.74795	YES	YES

2ba′

SCF Energy (au) BP86/SV(P) -2277.513915506
SCF Energy (au)PBE0/def2-TZVPP -2277.068149867
SCF Energy (au) PBE0/def2-TZVPP -2277.0753739100 (Toluene Correction)
Zero Point Energy (au) 0.2897742
Chemical Potential (kJ mol^{-1}) 615.97
Dispersion Correction (au) PBE0/def2-TZVPP -0.06339944

xyz coordinates
41

```
Mn    1.2258585    1.7959458    0.2361359
C     1.3726238    2.2084123    1.9870298
C     2.9819188    1.4607606    0.1062994
C     1.4143764    3.5362389   -0.2348193
O     1.5584791    4.6599386   -0.5184893
O     4.1193272    1.2134699    0.0247307
O     1.4762395    2.4873278    3.1120387
C    -1.4586829    0.7709355    0.9713587
C    -1.6429054    2.8509145   -0.1047515
C    -3.0388337    2.8435896   -0.0183476
C    -3.6627762    1.7421360    0.5941326
C    -2.8677031    0.7049515    1.0873249
C    -0.5234072   -0.2504435    1.4522467
C     0.8585732    0.0090274    1.2153396
C     1.7918361   -0.9298249    1.7020787
C     1.3783681   -2.0989935    2.3648978
C     0.0097808   -2.3548680    2.5709695
C    -0.9371225   -1.4300543    2.1171572
H    -1.1144621    3.6948098   -0.5748638
H    -3.6178365    3.6883408   -0.4221460
H    -4.7602400    1.6941301    0.6854248
H    -3.3329288   -0.1690477    1.5661469
H     2.8730423   -0.7571295    1.5689616
H     2.1337988   -2.8169591    2.7274855
H    -0.3163077   -3.2707270    3.0901531
H    -2.0076893   -1.6308725    2.2885396
N    -0.8676015    1.8541483    0.3737200
H     1.0761801    2.5805694   -2.6558619
C     0.9964970    1.8046790   -1.8925299
C     0.8866792    0.5828388   -1.5683411
C     0.6452584   -0.8197349   -1.8227326
C     0.1610902   -3.5448254   -2.4166148
C     1.4167314   -3.1382082   -1.9305350
C     1.6585020   -1.7912392   -1.6274982
C    -0.6176938   -1.2392218   -2.3130126
C    -0.8537739   -2.5906422   -2.6051716
H    -0.0258660   -4.6064105   -2.6487203
H     2.2185010   -3.8805090   -1.7827013
H     2.6422624   -1.4735862   -1.2495178
H    -1.4095959   -0.4891813   -2.4676116
H    -1.8404977   -2.9006858   -2.9879057
```

$vibrational spectrum

# mode	symmetry	wave number	IR intensity	selection rules	
#		cm**(-1)	km/mol	IR	RAMAN
1		0.00	0.00000	-	-
2		0.00	0.00000	-	-
3		0.00	0.00000	-	-
4		0.00	0.00000	-	-
5		0.00	0.00000	-	-
6		0.00	0.00000	-	-
7	a	17.53	0.21377	YES	YES

8	a	27.79	0.28921	YES	YES
9	a	45.99	0.13612	YES	YES
10	a	51.80	0.05357	YES	YES
11	a	64.59	0.06234	YES	YES
12	a	74.37	0.68844	YES	YES
13	a	87.29	0.50886	YES	YES
14	a	95.39	0.82393	YES	YES
15	a	97.32	0.37309	YES	YES
16	a	105.55	0.26004	YES	YES
17	a	113.04	0.17732	YES	YES
18	a	118.41	0.31008	YES	YES
19	a	133.64	0.12211	YES	YES
20	a	171.70	2.18610	YES	YES
21	a	174.78	1.43021	YES	YES
22	a	190.88	0.74865	YES	YES
23	a	218.04	0.51180	YES	YES
24	a	233.98	1.36411	YES	YES
25	a	264.26	0.52714	YES	YES
26	a	284.82	0.40484	YES	YES
27	a	325.68	12.47841	YES	YES
28	a	358.71	2.00023	YES	YES
29	a	399.66	1.03863	YES	YES
30	a	405.23	14.09467	YES	YES
31	a	418.07	2.17454	YES	YES
32	a	435.09	3.43305	YES	YES
33	a	457.07	1.70795	YES	YES
34	a	461.68	1.61109	YES	YES
35	a	469.82	4.73183	YES	YES
36	a	483.44	7.46993	YES	YES
37	a	488.14	0.26797	YES	YES
38	a	493.44	2.16853	YES	YES
39	a	502.03	3.50546	YES	YES
40	a	504.26	20.34088	YES	YES
41	a	528.28	12.34652	YES	YES
42	a	537.56	7.68174	YES	YES
43	a	556.97	2.32393	YES	YES
44	a	567.16	3.05453	YES	YES
45	a	612.23	15.52465	YES	YES
46	a	614.71	25.81800	YES	YES
47	a	623.17	8.97027	YES	YES
48	a	626.79	23.25932	YES	YES
49	a	642.59	20.75191	YES	YES
50	a	649.69	29.46792	YES	YES

2bb

```
SCF Energy (au) BP86/SV(P)                    -2505.237954427
SCF Energy (au) PBE0/def2-TZVPP               -2504.790885075
SCF Energy (au) PBE0/def2-TZVPP               -2504.7995679455 (Toluene Correction)
Zero Point Energy (au)                        0.3320389
Chemical Potential (kJ mol-1)                 708.87
Dispersion Correction (au) PBE0/def2-TZVPP   -0.06626996

xyz coordinates
47

Mn    1.6180000    0.5959888    1.9890749
C     1.6037650    1.0178592    3.7432338
C     3.3429124    0.1140385    2.0743823
C     2.0168363    2.3041750    1.5309996
O     2.2898686    3.4104105    1.2738312
O     4.4599601   -0.2173966    2.1300973
O     1.6041205    1.2900572    4.8745535
C    -1.2314929   -0.1224433    2.4276854
C    -1.0754915    1.9328046    1.3034667
C    -2.4658745    2.0706299    1.2336300
C    -3.2650505    1.0631172    1.7984665
C    -2.6424082   -0.0364777    2.3952798
C    -0.4700380   -1.2378566    3.0025114
C     0.9489658   -1.1392561    2.9070916
C     1.7198929   -2.1869073    3.4518553
C     1.1128659   -3.3042942    4.0519685
C    -0.2897640   -3.3964114    4.1319232
C    -1.0772339   -2.3642047    3.6093205
H    -0.4188630    2.7004377    0.8690315
H    -2.9019077    2.9514510    0.7386382
H    -4.3648766    1.1315375    1.7692289
H    -3.2468539   -0.8421105    2.8369312
H     2.8210083   -2.1434225    3.4119037
H     1.7424419   -4.1107013    4.4656753
H    -0.7662818   -4.2702563    4.6055253
H    -2.1750040   -2.4390751    3.6791968
N    -0.4669697    0.8778050    1.8856129
C     1.9148815    1.3242364   -1.3107508
C     1.6162769    0.4110700   -0.1848834
C     1.3516612   -0.7098415    0.3370742
H     1.1006053   -1.7696478    0.3104107
H     3.0138820    1.3998505   -1.4583892
O     1.4025969    0.8166996   -2.5617656
H     1.5066926    2.3409297   -1.1390848
C     0.1106493    1.1518490   -2.8526820
O    -0.5779918    1.8559542   -2.1311994
C    -0.3415613    0.5661932   -4.1515026
C    -1.2980410   -0.4808569   -6.5823375
C    -2.1297592    0.3353950   -5.7937381
C    -1.6539717    0.8572538   -4.5829244
C     0.4903588   -0.2538503   -4.9450346
C     0.0102220   -0.7740441   -6.1565301
H    -1.6719762   -0.8915143   -7.5350248
H    -3.1552568    0.5653103   -6.1266224
H    -2.2846628    1.4989506   -3.9479393
H     1.5114632   -0.4793423   -4.6037506
H     0.6614034   -1.4140937   -6.7744408
```

```
$vibrational spectrum
#  mode     symmetry     wave number   IR intensity    selection rules
#                         cm**(-1)        km/mol         IR     RAMAN
     1                      0.00         0.00000         -        -
     2                      0.00         0.00000         -        -
     3                      0.00         0.00000         -        -
     4                      0.00         0.00000         -        -
     5                      0.00         0.00000         -        -
     6                      0.00         0.00000         -        -
     7         a           13.78         0.63109        YES      YES
     8         a           16.17         0.06385        YES      YES
     9         a           26.51         0.94380        YES      YES
    10         a           40.83         0.11857        YES      YES
    11         a           44.50         0.14099        YES      YES
    12         a           60.04         0.64516        YES      YES
    13         a           69.03         0.21975        YES      YES
    14         a           72.21         0.85703        YES      YES
    15         a           84.34         1.45753        YES      YES
    16         a           90.90         0.52749        YES      YES
    17         a           96.39         0.34389        YES      YES
    18         a           97.44         0.34176        YES      YES
    19         a          108.60         0.17659        YES      YES
    20         a          112.21         0.23145        YES      YES
    21         a          134.87         0.21442        YES      YES
    22         a          147.43         0.82215        YES      YES
    23         a          158.85         0.08209        YES      YES
    24         a          184.11         0.56099        YES      YES
    25         a          192.44         2.38537        YES      YES
    26         a          210.96         1.77207        YES      YES
    27         a          233.43         1.65430        YES      YES
    28         a          247.96         0.63882        YES      YES
    29         a          261.35         0.30126        YES      YES
    30         a          284.47         0.09530        YES      YES
    31         a          304.39         0.64005        YES      YES
    32         a          335.44        25.56220        YES      YES
    33         a          352.01        10.82510        YES      YES
    34         a          358.09         2.98049        YES      YES
    35         a          403.84         0.00135        YES      YES
    36         a          404.89         3.35779        YES      YES
    37         a          418.21         9.17855        YES      YES
    38         a          437.11         1.26946        YES      YES
    39         a          443.49         0.54007        YES      YES
    40         a          456.37        13.63186        YES      YES
    41         a          456.91         1.18641        YES      YES
    42         a          470.12         3.60864        YES      YES
    43         a          474.64         4.67313        YES      YES
    44         a          485.86         2.37910        YES      YES
    45         a          492.24         4.47263        YES      YES
    46         a          503.55         2.17480        YES      YES
    47         a          534.83        27.57216        YES      YES
    48         a          551.35         6.93128        YES      YES
    49         a          555.83         2.85141        YES      YES
    50         a          586.75        24.75587        YES      YES
```

2bb′

SCF Energy (au) BP86/SV(P) -2505.236933617
SCF Energy (au) PBE0/def2-TZVPP -2504.790820918
SCF Energy (au) PBE0/def2-TZVPP -2504.8004063460 (Toluene Correction)
Zero Point Energy (au) 0.3315667
Chemical Potential (kJ mol^{-1}) 706.66
Dispersion Correction (au) PBE0/def2-TZVPP -0.06608119

xyz coordinates
47

Mn	1.2071453	2.0845764	1.5175484
C	1.5188067	2.4327129	3.2632450
C	2.9535568	1.8067143	1.2210547
C	1.3109007	3.8417076	1.0832671
O	1.3966017	4.9769109	0.8238098
O	4.0859643	1.6046888	1.0311148
O	1.7333257	2.6615735	4.3833466
C	-1.3840104	1.0164804	2.5067439
C	-1.6943190	3.1234148	1.5175162
C	-3.0701762	3.1092395	1.7689236
C	-3.6164244	1.9914272	2.4235040
C	-2.7682459	0.9435312	2.7903655
C	-0.4006485	-0.0225468	2.8330711
C	0.9456160	0.2488490	2.4458893
C	1.9236663	-0.7190291	2.7573426
C	1.5843701	-1.9224081	3.4016184
C	0.2501914	-2.1840534	3.7654303
C	-0.7378730	-1.2338403	3.4845280
H	-1.2264610	3.9816417	1.0106712
H	-3.6935940	3.9614037	1.4575101
H	-4.6949615	1.9379273	2.6444659
H	-3.1737855	0.0566284	3.2988505
H	2.9806457	-0.5440618	2.4946808
H	2.3709950	-2.6637524	3.6227759
H	-0.0168191	-3.1263013	4.2706177
H	-1.7801840	-1.4411310	3.7777907
N	-0.8674528	2.1174004	1.8745210
H	0.7237230	2.9428306	-1.3284374
C	0.7759412	2.1447418	-0.5880217
C	0.7649805	0.9334115	-0.2159492
C	0.6374431	-0.5091082	-0.4762539
H	1.5424555	-1.0611915	-0.1361967
O	0.4464628	-0.7112175	-1.8951661
H	-0.2152662	-0.9533645	0.0840899
C	0.3120342	-2.0094032	-2.2835204
C	0.1420535	-2.1399345	-3.7650974
O	0.3329019	-2.9454306	-1.5003728
C	-0.1749361	-2.5062572	-6.5373558
C	-0.0310494	-1.2097279	-6.0112969
C	0.1273187	-1.0236341	-4.6295330
C	-0.0029913	-3.4390717	-4.2974052
C	-0.1608425	-3.6207548	-5.6784601
H	-0.2979793	-2.6493881	-7.6239929
H	-0.0414565	-0.3366498	-6.6845058
H	0.2423172	-0.0138216	-4.2085190
H	0.0128639	-4.2944664	-3.6037226
H	-0.2728045	-4.6372664	-6.0904863

```
$vibrational spectrum
#  mode     symmetry     wave number   IR intensity    selection rules
#                         cm**(-1)        km/mol         IR     RAMAN
     1                      0.00          0.00000         -       -
     2                      0.00          0.00000         -       -
     3                      0.00          0.00000         -       -
     4                      0.00          0.00000         -       -
     5                      0.00          0.00000         -       -
     6                      0.00          0.00000         -       -
     7        a            12.69          0.77150        YES     YES
     8        a            14.31          0.44876        YES     YES
     9        a            27.98          0.18749        YES     YES
    10        a            41.43          0.34307        YES     YES
    11        a            51.94          0.21434        YES     YES
    12        a            56.54          0.08029        YES     YES
    13        a            68.27          0.37237        YES     YES
    14        a            70.82          0.45542        YES     YES
    15        a            83.57          0.05551        YES     YES
    16        a            85.91          0.91697        YES     YES
    17        a            96.63          0.69206        YES     YES
    18        a           101.41          0.21988        YES     YES
    19        a           107.13          0.45072        YES     YES
    20        a           111.31          0.30546        YES     YES
    21        a           128.18          0.55150        YES     YES
    22        a           135.51          0.37011        YES     YES
    23        a           157.84          0.40895        YES     YES
    24        a           186.62          0.71237        YES     YES
    25        a           188.13          0.18813        YES     YES
    26        a           206.37          1.05808        YES     YES
    27        a           220.77          1.27731        YES     YES
    28        a           235.13          0.37823        YES     YES
    29        a           265.42          0.69843        YES     YES
    30        a           281.00          2.87185        YES     YES
    31        a           293.67         13.05060        YES     YES
    32        a           322.49          3.95365        YES     YES
    33        a           354.65          5.38279        YES     YES
    34        a           358.97          1.99179        YES     YES
    35        a           396.54         16.64607        YES     YES
    36        a           403.91          0.00075        YES     YES
    37        a           418.13          3.11457        YES     YES
    38        a           436.08          2.26241        YES     YES
    39        a           445.18          0.20183        YES     YES
    40        a           454.14          2.00641        YES     YES
    41        a           458.13          1.36604        YES     YES
    42        a           469.31          2.75263        YES     YES
    43        a           473.59          6.60098        YES     YES
    44        a           487.20          4.16042        YES     YES
    45        a           491.46          3.65311        YES     YES
    46        a           504.47          3.40863        YES     YES
    47        a           529.10         20.02646        YES     YES
    48        a           547.18          6.22080        YES     YES
    49        a           557.27          2.47483        YES     YES
    50        a           585.62          8.21677        YES     YES
```

2bc

```
SCF Energy (au) BP86/SV(P)                  -2281.120982087
SCF Energy (au) PBE0/def2-TZVPP             -2280.691348894
SCF Energy (au) PBE0/def2-TZVPP             -2280.6975966102 (Toluene Correction)
Zero Point Energy (au)                      0.3575200
Chemical Potential (kJ mol-1)               791.47
Dispersion Correction (au) PBE0/def2-TZVPP  -0.06509361
```

xyz coordinates
47

```
Mn    1.2025069    0.1620360    1.0932740
C     1.9568143    0.6239857    2.6631145
C     2.7626127   -0.4707803    0.4843492
C     1.4816754    1.8042180    0.3880616
O     1.6872442    2.8755222   -0.0350056
O     3.7740952   -0.8988125    0.0895477
O     2.4567165    0.9146208    3.6736798
C    -1.2674439   -0.3023652    2.6745928
C    -1.3912158    1.7610984    1.5612277
C    -2.6659539    2.0309656    2.0698859
C    -3.2584096    1.0819363    2.9213569
C    -2.5540902   -0.0861767    3.2217314
C    -0.4365673   -1.4859299    2.9195833
C     0.8303787   -1.5029309    2.2647405
C     1.6666344   -2.6177159    2.4886735
C     1.2610305   -3.6855246    3.3088101
C     0.0017371   -3.6623661    3.9393346
C    -0.8426867   -2.5634264    3.7452354
H    -0.8890702    2.4838705    0.8994692
H    -3.1751348    2.9700773    1.8036640
H    -4.2600129    1.2541116    3.3483852
H    -2.9958712   -0.8431618    3.8858832
H     2.6605532   -2.6671882    2.0133031
H     1.9364916   -4.5450173    3.4598796
H    -0.3169334   -4.4982914    4.5831522
H    -1.8256840   -2.5481467    4.2443786
N    -0.7037795    0.6342783    1.8461413
C     0.1497651    0.7721964   -2.1486257
C     0.2718754   -0.0683968   -0.9320319
C     0.1891642   -1.1366730   -0.2631221
H    -0.0993771   -2.1746493   -0.1043557
C     1.4730397    0.8028500   -2.9612945
C    -1.0183065    0.2671813   -3.0382063
H    -0.0792297    1.8227280   -1.8535928
C    -1.1565103    1.1132381   -4.3163661
H    -1.9654677    0.2801394   -2.4548417
H    -0.8234627   -0.7970045   -3.3086006
H     2.2961270    1.1998921   -2.3286030
C     1.3263403    1.6449803   -4.2404805
H     1.7477958   -0.2449558   -3.2238571
H     1.1602417    2.7121905   -3.9595975
H     2.2790566    1.6176752   -4.8155415
C     0.1575487    1.1588161   -5.1149762
H    -1.4552353    2.1520586   -4.0381253
H    -1.9808817    0.7083946   -4.9460352
H     0.0445918    1.8137098   -6.0082216
H     0.3872873    0.1367419   -5.4999742
```

$vibrational spectrum

# mode	symmetry	wave number	IR intensity	selection rules	
#		cm**(-1)	km/mol	IR	RAMAN
1		0.00	0.00000	-	-
2		0.00	0.00000	-	-
3		0.00	0.00000	-	-
4		0.00	0.00000	-	-
5		0.00	0.00000	-	-
6		0.00	0.00000	-	-
7	a	21.31	0.24853	YES	YES
8	a	34.94	0.01906	YES	YES
9	a	38.46	0.04270	YES	YES
10	a	56.12	0.16270	YES	YES
11	a	66.63	0.03245	YES	YES
12	a	77.27	0.13836	YES	YES
13	a	83.57	0.79291	YES	YES
14	a	91.64	0.35616	YES	YES
15	a	98.95	0.67833	YES	YES
16	a	107.88	0.25597	YES	YES
17	a	112.19	0.26611	YES	YES
18	a	116.96	0.11094	YES	YES
19	a	125.21	0.31890	YES	YES
20	a	144.42	2.49941	YES	YES
21	a	170.19	2.12557	YES	YES
22	a	188.62	1.02686	YES	YES
23	a	213.81	0.39124	YES	YES
24	a	230.38	0.08836	YES	YES
25	a	242.25	1.38666	YES	YES
26	a	245.53	2.60861	YES	YES
27	a	264.09	0.25243	YES	YES
28	a	284.24	0.01913	YES	YES
29	a	348.77	2.37378	YES	YES
30	a	358.13	2.17632	YES	YES
31	a	381.93	9.42387	YES	YES
32	a	409.55	0.56836	YES	YES
33	a	417.09	5.99506	YES	YES
34	a	425.66	0.41850	YES	YES
35	a	436.05	0.68267	YES	YES
36	a	454.69	13.16624	YES	YES
37	a	457.31	1.49122	YES	YES
38	a	473.52	2.84621	YES	YES
39	a	483.72	3.74094	YES	YES
40	a	493.07	1.51086	YES	YES
41	a	495.13	1.75850	YES	YES
42	a	497.07	5.09040	YES	YES
43	a	505.51	2.43664	YES	YES
44	a	535.73	28.50850	YES	YES
45	a	548.84	10.33325	YES	YES
46	a	557.11	1.46236	YES	YES
47	a	563.44	9.53427	YES	YES
48	a	615.81	12.89888	YES	YES
49	a	624.34	4.08364	YES	YES
50	a	627.92	29.19440	YES	YES

2bc′

SCF Energy (au) BP86/SV(P)	-2281.119860015
SCF Energy (au) PBE0/def2-TZVPP	-2280.690435817
SCF Energy (au) PBE0/def2-TZVPP	-2280.6964385324 (Toluene Correction)
Zero Point Energy (au)	0.3576711
Chemical Potential (kJ mol^{-1})	793.30
Dispersion Correction (au) PBE0/def2-TZVPP	-0.06716635

xyz coordinates

47

Mn	1.0348289	2.0037251	0.7072396
C	1.3546343	2.3648770	2.4445458
C	2.7685807	1.6679846	0.4096337
C	1.1942521	3.7522247	0.2578736
O	1.3200183	4.8827259	-0.0095936
O	3.8951655	1.4302241	0.2189937
O	1.5757112	2.6034661	3.5623050
C	-1.5965905	0.9998528	1.6467973
C	-1.8317482	3.1290658	0.6865140
C	-3.2127062	3.1468550	0.9091137
C	-3.8017169	2.0327022	1.5320069
C	-2.9882074	0.9577381	1.8992821
C	-0.6444245	-0.0631017	1.9870364
C	0.7165889	0.1839616	1.6335863
C	1.6659530	-0.7997902	1.9866181
C	1.2861615	-1.9957063	2.6221302
C	-0.0641999	-2.2359852	2.9407155
C	-1.0246722	-1.2672621	2.6284229
H	-1.3310466	3.9832302	0.2046771
H	-3.8068641	4.0207289	0.6004812
H	-4.8858393	2.0035153	1.7292105
H	-3.4267504	0.0739880	2.3855938
H	2.7350754	-0.6397247	1.7674973
H	2.0539780	-2.7462203	2.8771043
H	-0.3635825	-3.1712938	3.4410703
H	-2.0788268	-1.4523673	2.8937919
N	-1.0386625	2.0959762	1.0422653
H	0.5636553	2.8721143	-2.1221330
C	0.6035118	2.0483260	-1.4099698
C	0.5517948	0.8292798	-1.0716342
C	0.3951208	-0.6125177	-1.3884540
C	1.7673471	-1.2751013	-1.6862411
C	-0.5722618	-0.8124266	-2.5852298
H	-0.0375554	-1.1334973	-0.5047080
C	-0.7282709	-2.3028996	-2.9361699
H	-0.1700283	-0.2584620	-3.4657544
H	-1.5610590	-0.3609318	-2.3471509
H	2.2469902	-0.7321242	-2.5336279
C	1.6047150	-2.7634174	-2.0409011
H	2.4376173	-1.1564950	-0.8086280
H	-1.4020842	-2.4135281	-3.8158544
H	-1.2286084	-2.8281054	-2.0881917
C	0.6309114	-2.9683156	-3.2143939
H	2.5998664	-3.2014382	-2.2815466
H	1.2262434	-3.3119724	-1.1461211
H	0.4947536	-4.0544028	-3.4193741
H	1.0722309	-2.5254750	-4.1388293

```
$vibrational spectrum
#  mode     symmetry     wave number   IR intensity    selection rules
#                         cm**(-1)        km/mol         IR     RAMAN
     1                      0.00         0.00000         -       -
     2                      0.00         0.00000         -       -
     3                      0.00         0.00000         -       -
     4                      0.00         0.00000         -       -
     5                      0.00         0.00000         -       -
     6                      0.00         0.00000         -       -
     7         a           24.72         0.36389        YES     YES
     8         a           41.59         0.07488        YES     YES
     9         a           45.25         0.06575        YES     YES
    10         a           52.15         0.11210        YES     YES
    11         a           63.64         0.04811        YES     YES
    12         a           76.29         0.28325        YES     YES
    13         a           87.06         0.59299        YES     YES
    14         a           94.90         0.57956        YES     YES
    15         a           96.08         0.16813        YES     YES
    16         a          104.24         0.55311        YES     YES
    17         a          111.89         0.16725        YES     YES
    18         a          117.23         0.10103        YES     YES
    19         a          125.86         0.20698        YES     YES
    20         a          155.34         0.95071        YES     YES
    21         a          176.64         2.31596        YES     YES
    22         a          189.40         0.72962        YES     YES
    23         a          217.68         0.38679        YES     YES
    24         a          228.82         1.35028        YES     YES
    25         a          239.91         0.04320        YES     YES
    26         a          252.70         0.27808        YES     YES
    27         a          267.89         0.60585        YES     YES
    28         a          285.93         0.07364        YES     YES
    29         a          357.82         1.81800        YES     YES
    30         a          358.68         1.20955        YES     YES
    31         a          379.42        12.58201        YES     YES
    32         a          413.80         2.47893        YES     YES
    33         a          421.06         2.16931        YES     YES
    34         a          425.08         1.18950        YES     YES
    35         a          436.27         1.59759        YES     YES
    36         a          457.23         1.43469        YES     YES
    37         a          460.02         1.13592        YES     YES
    38         a          472.28         2.42811        YES     YES
    39         a          485.41         5.28717        YES     YES
    40         a          489.47         2.52597        YES     YES
    41         a          494.19         9.12418        YES     YES
    42         a          498.54         0.71289        YES     YES
    43         a          508.60         2.78867        YES     YES
    44         a          529.06        16.45843        YES     YES
    45         a          554.23         2.74309        YES     YES
    46         a          558.08         2.78602        YES     YES
    47         a          580.21         5.14682        YES     YES
    48         a          615.69        26.75293        YES     YES
    49         a          623.84         7.32930        YES     YES
    50         a          627.13        24.83024        YES     YES
```

2bd

SCF Energy (au) BP86/SV(P)	-2411.386418687
SCF Energy (au) PBE0/def2-TZVPP	-2410.929730749
SCF Energy (au) PBE0/def2-TZVPP	-2410.9384397830 (Toluene Correction)
Zero Point Energy (au)	0.3601077
Chemical Potential (kJ mol^{-1})	781.98
Dispersion Correction (au) PBE0/def2-TZVPP	-0.06936495

xyz coordinates
49

```
Mn    1.3925957   -0.2876905    1.7876510
C     1.8013007    0.3501230    3.4179483
C     3.0913075   -0.7763907    1.5034861
C     1.6475177    1.3305527    1.0040488
O     1.8535412    2.3906773    0.5573821
O     4.1949478   -1.1118628    1.3195940
O     2.0741051    0.7505419    4.4776491
C    -1.2733784   -0.9329752    2.9018538
C    -1.3729850    1.0364967    1.6250640
C    -2.7435242    1.1929112    1.8591491
C    -3.3985417    0.2359856    2.6539271
C    -2.6578172   -0.8284409    3.1737159
C    -0.3957473   -1.9991732    3.3944334
C     0.9719780   -1.9081982    2.9989035
C     1.8536763   -2.9048410    3.4696009
C     1.4022611   -3.9601422    4.2816231
C     0.0462868   -4.0446748    4.6538200
C    -0.8488814   -3.0647430    4.2109765
H    -0.8228403    1.7657238    1.0107653
H    -3.2774785    2.0527496    1.4261788
H    -4.4767091    0.3212295    2.8676273
H    -3.1472115   -1.5898039    3.7984506
H     2.9227283   -2.8677717    3.1999172
H     2.1173776   -4.7245731    4.6315816
H    -0.3082608   -4.8711275    5.2912563
H    -1.9088144   -3.1333749    4.5079584
N    -0.6495121    0.0106224    2.1251006
C     0.5362402    0.0227771   -1.5621652
C     1.5876226    0.6627500   -2.2691490
C     1.3426892    1.4197705   -3.4145819
C     0.0190698    1.5921664   -3.9213900
C    -1.0380191    0.9549214   -3.2052885
C    -0.7817999    0.1962930   -2.0610430
H     2.6225990    0.5547868   -1.9084296
H     2.1984547    1.8871607   -3.9215151
N    -0.2266218    2.3434817   -5.0577128
H    -2.0784857    1.0466807   -3.5477473
H    -1.6206983   -0.2895092   -1.5372084
C     0.7777766   -0.8031245   -0.4136716
C     0.8486570   -1.7866419    0.3766648
H     0.8017729   -2.8648223    0.5341916
C    -1.5867253    2.5044316   -5.5419388
C     0.8740447    2.9812261   -5.7608789
H    -1.5795323    3.1316451   -6.4551821
H    -2.2440036    3.0052468   -4.7904017
H    -2.0562801    1.5261755   -5.8054631
H     1.4160308    3.7142408   -5.1163019
H     0.4790910    3.5301722   -6.6385994
H     1.6201958    2.2383414   -6.1318513
```

$vibrational spectrum

# mode	symmetry	wave number	IR intensity	selection rules	
#		cm**(-1)	km/mol	IR	RAMAN
1		0.00	0.00000	-	-
2		0.00	0.00000	-	-
3		0.00	0.00000	-	-
4		0.00	0.00000	-	-
5		0.00	0.00000	-	-
6		0.00	0.00000	-	-
7	a	9.77	0.32390	YES	YES
8	a	19.49	0.47829	YES	YES
9	a	27.15	0.84414	YES	YES
10	a	46.55	0.42645	YES	YES
11	a	59.63	0.00369	YES	YES
12	a	65.68	0.40984	YES	YES
13	a	73.58	0.27310	YES	YES
14	a	77.07	1.07304	YES	YES
15	a	85.22	1.87030	YES	YES
16	a	86.06	0.62738	YES	YES
17	a	93.48	0.56900	YES	YES
18	a	101.53	1.07347	YES	YES
19	a	105.80	0.14299	YES	YES
20	a	110.21	0.77764	YES	YES
21	a	113.50	1.28888	YES	YES
22	a	119.50	10.86627	YES	YES
23	a	145.24	8.95151	YES	YES
24	a	176.05	12.15749	YES	YES
25	a	189.53	2.39487	YES	YES
26	a	211.64	0.15863	YES	YES
27	a	222.89	0.47711	YES	YES
28	a	238.63	2.17760	YES	YES
29	a	247.84	5.48849	YES	YES
30	a	261.30	2.39248	YES	YES
31	a	271.68	15.76820	YES	YES
32	a	287.07	1.13771	YES	YES
33	a	340.00	40.93114	YES	YES
34	a	358.73	1.91607	YES	YES
35	a	380.91	7.35874	YES	YES
36	a	416.00	11.15977	YES	YES
37	a	416.77	1.74031	YES	YES
38	a	430.09	6.89579	YES	YES
39	a	444.10	19.38440	YES	YES
40	a	454.05	5.54465	YES	YES
41	a	456.73	3.22540	YES	YES
42	a	463.11	1.77583	YES	YES
43	a	477.34	6.19032	YES	YES
44	a	488.73	3.79858	YES	YES
45	a	490.92	32.76320	YES	YES
46	a	493.72	5.07364	YES	YES
47	a	503.77	0.62555	YES	YES
48	a	515.82	15.69978	YES	YES
49	a	530.05	43.15597	YES	YES
50	a	541.11	9.60241	YES	YES

2bd′

SCF Energy (au) BP86/SV(P) -2411.383229723
SCF Energy (au) PBE0/def2-TZVPP -2410.926493690
SCF Energy (au) PBE0/def2-TZVPP -2410.9348447962 (Toluene Correction)
Zero Point Energy (au) 0.3602809
Chemical Potential (kJ mol^{-1}) 784.73
Dispersion Correction (au) PBE0/def2-TZVPP -0.07187072

xyz coordinates
49

Mn	1.2663945	2.7708307	0.7416893
C	1.4115506	3.1773508	2.4898686
C	3.0235475	2.4470980	0.6081276
C	1.4377476	4.5142685	0.2781122
O	1.5718707	5.6406263	-0.0035219
O	4.1629481	2.2064819	0.5236885
O	1.5137044	3.4534435	3.6166834
C	-1.4098168	1.6986085	1.4244989
C	-1.6060832	3.7981651	0.3895549
C	-3.0028394	3.7713887	0.4556699
C	-3.6212881	2.6491378	1.0354332
C	-2.8195531	1.6122698	1.5180890
C	-0.4678684	0.6812651	1.8996707
C	0.9129646	0.9617008	1.6764839
C	1.8511667	0.0264272	2.1633669
C	1.4456810	-1.1538910	2.8107245
C	0.0782650	-1.4282234	3.0020761
C	-0.8747426	-0.5094966	2.5486916
H	-1.0820154	4.6583592	-0.0550827
H	-3.5867634	4.6172874	0.0613717
H	-4.7193202	2.5851346	1.1094389
H	-3.2796616	0.7219630	1.9712661
H	2.9317907	0.2123774	2.0422032
H	2.2063352	-1.8663679	3.1738634
H	-0.2418906	-2.3522338	3.5107730
H	-1.9446100	-0.7226850	2.7103168
N	-0.8239537	2.8016353	0.8578603
H	1.1464438	3.5642565	-2.1452500
C	1.0506260	2.7722205	-1.4007081
C	0.9395331	1.5400747	-1.1284499
C	0.6900209	0.1433434	-1.3608377
C	0.1781382	-2.6299613	-1.9369723
C	1.4415419	-2.1922956	-1.4386935
C	1.6820764	-0.8477882	-1.1527721
C	-0.5672552	-0.2881822	-1.8552967
C	-0.8214450	-1.6326957	-2.1363533
N	-0.0659869	-3.9654548	-2.2155184
H	2.2525575	-2.9136067	-1.2664274
H	2.6699657	-0.5470040	-0.7712270
H	-1.3600945	0.4573031	-2.0274221
H	-1.8144344	-1.9063088	-2.5204744
C	-1.3609442	-4.3745029	-2.7290627
C	0.9772797	-4.9540055	-2.0061756
H	-1.6005157	-3.8869288	-3.7054739
H	-2.1894685	-4.1344632	-2.0195169
H	-1.3624895	-5.4706853	-2.8908988
H	1.3071293	-4.9967255	-0.9402114
H	1.8802211	-4.7528585	-2.6321530
H	0.5935398	-5.9566532	-2.2810229

$vibrational spectrum

# mode	symmetry	wave number	IR intensity	selection rules	
#		cm**(-1)	km/mol	IR	RAMAN
1		0.00	0.00000	-	-
2		0.00	0.00000	-	-
3		0.00	0.00000	-	-
4		0.00	0.00000	-	-
5		0.00	0.00000	-	-
6		0.00	0.00000	-	-
7	a	11.44	0.42500	YES	YES
8	a	22.17	0.05493	YES	YES
9	a	36.74	0.87475	YES	YES
10	a	48.62	0.05701	YES	YES
11	a	58.52	1.12493	YES	YES
12	a	66.92	0.69761	YES	YES
13	a	74.69	0.34991	YES	YES
14	a	78.56	0.56269	YES	YES
15	a	84.94	0.59614	YES	YES
16	a	92.36	1.30066	YES	YES
17	a	94.03	0.15738	YES	YES
18	a	97.12	1.58291	YES	YES
19	a	104.13	0.18552	YES	YES
20	a	109.18	0.28495	YES	YES
21	a	117.19	0.83929	YES	YES
22	a	128.57	2.09589	YES	YES
23	a	149.49	6.73147	YES	YES
24	a	184.48	5.05036	YES	YES
25	a	194.26	4.53220	YES	YES
26	a	214.95	0.25485	YES	YES
27	a	216.70	1.13080	YES	YES
28	a	232.49	1.84431	YES	YES
29	a	250.02	1.07986	YES	YES
30	a	263.54	0.20893	YES	YES
31	a	279.47	8.73793	YES	YES
32	a	286.00	0.78289	YES	YES
33	a	338.56	29.90249	YES	YES
34	a	359.12	2.14220	YES	YES
35	a	389.80	11.97136	YES	YES
36	a	414.66	1.31078	YES	YES
37	a	416.05	1.78230	YES	YES
38	a	428.09	10.10769	YES	YES
39	a	441.38	4.97791	YES	YES
40	a	453.54	0.48186	YES	YES
41	a	458.02	1.92772	YES	YES
42	a	467.96	3.93729	YES	YES
43	a	475.17	0.36120	YES	YES
44	a	488.63	5.04201	YES	YES
45	a	491.82	6.89705	YES	YES
46	a	494.74	10.14941	YES	YES
47	a	503.51	1.69264	YES	YES
48	a	515.44	19.65428	YES	YES
49	a	532.86	21.08297	YES	YES
50	a	537.10	6.36060	YES	YES

2be

```
SCF Energy (au) BP86/SV(P                      -2391.960168240
SCF Energy (au) PBE0/def2-TZVPP                -2391.515765535
SCF Energy (au) PBE0/def2-TZVPP                -2391.5241006317 (Toluene Correction)
Zero Point Energy (au)                         0.3213065
Chemical Potential (kJ mol-1)                  689.58
Dispersion Correction (au) PBE0/def2-TZVPP -0.06471746

xyz coordinates
45

Mn    1.2454519   -0.1636177    1.2461568
C     1.6903274    0.3277975    2.9190853
C     2.9289665   -0.6687297    0.9011828
C     1.5333880    1.5091092    0.6006049
O     1.7617757    2.5966867    0.2410407
O     4.0223501   -1.0121397    0.6782891
O     1.9851445    0.6352659    4.0029599
C    -1.4146805   -0.8122930    2.3802386
C    -1.4917666    1.2371547    1.2347438
C    -2.8522728    1.4111914    1.5105874
C    -3.5136263    0.4212793    2.2581960
C    -2.7895105   -0.6922078    2.6912325
C    -0.5548900   -1.9308340    2.7806847
C     0.8047510   -1.8522571    2.3571412
C     1.6701394   -2.9000083    2.7373162
C     1.2087281   -3.9945510    3.4897093
C    -0.1393528   -4.0664895    3.8914818
C    -1.0169583   -3.0354668    3.5381285
H    -0.9371454    1.9909849    0.6548643
H    -3.3738921    2.3091286    1.1452779
H    -4.5843606    0.5185292    2.5017612
H    -3.2847640   -1.4804512    3.2767930
H     2.7327403   -2.8730782    2.4426186
H     1.9100487   -4.7995708    3.7689854
H    -0.5016334   -4.9231798    4.4828214
H    -2.0703379   -3.0946166    3.8588525
N    -0.7844127    0.1642747    1.6516163
C     0.3793082    0.3930496   -2.0429389
C     1.4345356    1.0980323   -2.6697050
C     1.2055671    1.9129660   -3.7871178
C    -0.1023718    2.0528749   -4.3032530
C    -1.1681430    1.3593666   -3.6828095
C    -0.9317977    0.5456915   -2.5743106
H     2.4585896    0.9989824   -2.2773754
H     2.0563759    2.4375977   -4.2458196
O    -0.4334359    2.8199435   -5.3773085
H    -2.1811248    1.4757238   -4.0991596
H    -1.7678363    0.0040392   -2.1041006
C     0.6035550   -0.4910616   -0.9271151
C     0.6165874   -1.5478216   -0.2322546
H     0.5125044   -2.6303703   -0.1533227
C     0.5904440    3.5378490   -6.0467717
H     0.0933132    4.0802171   -6.8759145
H     1.3640469    2.8545614   -6.4703382
H     1.0856745    4.2764476   -5.3727552

$vibrational spectrum
#  mode     symmetry     wave number   IR intensity    selection rules
#                         cm**(-1)        km/mol          IR     RAMAN
     1                         0.00         0.00000        -       -
     2                         0.00         0.00000        -       -
     3                         0.00         0.00000        -       -
```

4		0.00	0.00000	-	-
5		0.00	0.00000	-	-
6		0.00	0.00000	-	-
7	a	14.73	0.48528	YES	YES
8	a	27.54	0.12557	YES	YES
9	a	33.83	1.31325	YES	YES
10	a	49.40	0.41729	YES	YES
11	a	63.10	0.05113	YES	YES
12	a	68.82	0.12965	YES	YES
13	a	84.84	1.50868	YES	YES
14	a	87.60	0.96268	YES	YES
15	a	94.72	0.65057	YES	YES
16	a	98.26	0.23069	YES	YES
17	a	107.68	0.58782	YES	YES
18	a	111.22	0.26776	YES	YES
19	a	113.72	0.43342	YES	YES
20	a	124.37	0.81217	YES	YES
21	a	142.73	7.98195	YES	YES
22	a	152.38	4.16835	YES	YES
23	a	188.93	1.98886	YES	YES
24	a	208.51	4.11258	YES	YES
25	a	232.84	2.10901	YES	YES
26	a	242.91	6.62047	YES	YES
27	a	251.98	0.82755	YES	YES
28	a	262.32	0.48982	YES	YES
29	a	275.23	8.01602	YES	YES
30	a	286.09	0.47693	YES	YES
31	a	357.60	4.93075	YES	YES
32	a	371.46	41.59569	YES	YES
33	a	401.58	1.57177	YES	YES
34	a	410.70	0.87504	YES	YES
35	a	417.33	7.84680	YES	YES
36	a	431.89	5.36836	YES	YES
37	a	447.02	9.62630	YES	YES
38	a	456.52	1.15156	YES	YES
39	a	461.81	2.74505	YES	YES
40	a	475.05	13.77999	YES	YES
41	a	477.54	10.19568	YES	YES
42	a	488.10	3.35668	YES	YES
43	a	493.60	4.70623	YES	YES
44	a	503.81	2.30819	YES	YES
45	a	521.30	53.32399	YES	YES
46	a	530.71	22.79117	YES	YES
47	a	541.23	7.10764	YES	YES
48	a	551.30	6.72379	YES	YES
49	a	557.16	1.63201	YES	YES
50	a	610.92	29.31754	YES	YES

2be′

```
SCF Energy (au) BP86/SV(P)                      -2391.957322776
SCF Energy (au) PBE0/def2-TZVPP                 -2391.512741707
SCF Energy (au) PBE0/def2-TZVPP                 -2391.5210557956 (Toluene Correction)
Zero Point Energy (au)                          0.3212850
Chemical Potential (kJ mol-1)                   689.45
Dispersion Correction (au) PBE0/def2-TZVPP  -0.06706478

xyz coordinates
45

Mn    1.2018114    2.3230176    0.7548611
C     1.1966094    2.6674406    2.5251254
C     2.9759053    2.0685991    0.7475998
C     1.3417653    4.0885188    0.3717193
O     1.4525414    5.2294814    0.1461870
O     4.1263842    1.8728426    0.7439207
O     1.2027896    2.9051979    3.6646116
C    -1.4774602    1.1365428    1.2069601
C    -1.6775184    3.2640542    0.2317406
C    -3.0735233    3.1863285    0.1953522
C    -3.6891506    2.0223060    0.6893591
C    -2.8858991    0.9971163    1.1939489
C    -0.5349590    0.1369759    1.7179660
C     0.8469663    0.4745153    1.6144229
C     1.7813724   -0.4410436    2.1429074
C     1.3738901   -1.6595390    2.7139420
C     0.0079458   -1.9916137    2.7849735
C    -0.9429351   -1.0919669    2.2907844
H    -1.1555815    4.1579091   -0.1438556
H    -3.6592854    4.0254717   -0.2104464
H    -4.7864733    1.9171727    0.6805989
H    -3.3436826    0.0755080    1.5820225
H     2.8596110   -0.2102367    2.1153004
H     2.1313858   -2.3568796    3.1110001
H    -0.3134988   -2.9462274    3.2324524
H    -2.0125979   -1.3508374    2.3596834
N    -0.8945810    2.2786934    0.7214380
H     1.2437389    3.2338625   -2.1008219
C     1.1369187    2.4156359   -1.3866901
C     1.0657742    1.1735248   -1.1394614
C     0.9027471   -0.2214474   -1.4706394
C     0.5774429   -2.9415021   -2.2325676
C     1.7520298   -2.5162861   -1.5678323
C     1.9080735   -1.1843100   -1.1866765
C    -0.2675862   -0.6588157   -2.1352765
C    -0.4362164   -1.9999745   -2.5139696
O     0.5206688   -4.2637539   -2.5571430
H     2.5333940   -3.2634943   -1.3587178
H     2.8282443   -0.8669552   -0.6735143
H    -1.0597815    0.0716795   -2.3632990
H    -1.3607870   -2.2966877   -3.0307668
C    -0.6259038   -4.7517284   -3.2312324
H    -0.7672266   -4.2549073   -4.2209286
H    -1.5520862   -4.6194973   -2.6224653
H    -0.4512763   -5.8346902   -3.3925732

$vibrational spectrum
#  mode     symmetry     wave number   IR intensity    selection rules
#                         cm**(-1)        km/mol          IR     RAMAN
     1                       0.00         0.00000          -       -
     2                       0.00         0.00000          -       -
     3                       0.00         0.00000          -       -
```

4		0.00	0.00000	-	-
5		0.00	0.00000	-	-
6		0.00	0.00000	-	-
7	a	13.39	0.19688	YES	YES
8	a	19.98	0.14787	YES	YES
9	a	44.37	0.00775	YES	YES
10	a	48.89	0.01718	YES	YES
11	a	61.20	0.59746	YES	YES
12	a	68.25	0.68447	YES	YES
13	a	81.88	0.18327	YES	YES
14	a	87.08	1.58370	YES	YES
15	a	95.70	0.24980	YES	YES
16	a	97.57	0.86361	YES	YES
17	a	102.60	0.63889	YES	YES
18	a	110.71	0.22605	YES	YES
19	a	117.52	0.87571	YES	YES
20	a	122.30	0.55115	YES	YES
21	a	150.40	5.39288	YES	YES
22	a	161.61	1.44765	YES	YES
23	a	190.00	0.15297	YES	YES
24	a	216.68	1.37135	YES	YES
25	a	225.75	4.27679	YES	YES
26	a	237.39	0.07127	YES	YES
27	a	253.95	0.92664	YES	YES
28	a	264.92	0.43227	YES	YES
29	a	278.53	4.50877	YES	YES
30	a	285.83	0.30984	YES	YES
31	a	358.53	2.62385	YES	YES
32	a	375.40	30.36449	YES	YES
33	a	396.79	5.19120	YES	YES
34	a	411.11	1.55916	YES	YES
35	a	417.35	3.54155	YES	YES
36	a	431.69	7.33705	YES	YES
37	a	441.25	3.09284	YES	YES
38	a	457.69	1.41522	YES	YES
39	a	467.45	2.67182	YES	YES
40	a	473.68	0.88691	YES	YES
41	a	487.20	13.32081	YES	YES
42	a	489.99	5.98088	YES	YES
43	a	492.50	15.77534	YES	YES
44	a	502.67	4.65991	YES	YES
45	a	517.38	1.53708	YES	YES
46	a	531.20	25.42967	YES	YES
47	a	538.74	7.43532	YES	YES
48	a	556.96	2.89561	YES	YES
49	a	566.53	2.71462	YES	YES
50	a	614.64	47.84106	YES	YES

2bf

```
SCF Energy (au) BP86/SV(P)                      -2376.686035034
SCF Energy (au) PBE0/def2-TZVPP                 -2376.263171392
SCF Energy (au) PBE0/def2-TZVPP                 -2376.2704120363 (Toluene Correction)
Zero Point Energy (au)                          0.2818722
Chemical Potential (kJ mol-1)                   591.01
Dispersion Correction (au) PBE0/def2-TZVPP      -0.06135801
```

```
xyz coordinates
41

Mn    1.3698504    0.2105297    0.6366223
C     1.8030365    0.6855900    2.3195994
C     3.0564347   -0.2930315    0.2993493
C     1.6658100    1.8905940    0.0127788
O     1.8958418    2.9812785   -0.3345833
O     4.1506892   -0.6357557    0.0829671
O     2.0890447    0.9837826    3.4077538
C    -1.3001451   -0.4349008    1.7536845
C    -1.3619793    1.6277135    0.6309972
C    -2.7232705    1.8043899    0.9005427
C    -3.3934818    0.8079514    1.6314084
C    -2.6767037   -0.3132861    2.0565742
C    -0.4461613   -1.5590405    2.1509347
C     0.9172178   -1.4817969    1.7399506
C     1.7783799   -2.5325499    2.1201434
C     1.3081757   -3.6313270    2.8607364
C    -0.0437438   -3.7037738    3.2492435
C    -0.9163816   -2.6685955    2.8960569
H    -0.8006641    2.3863890    0.0640316
H    -3.2386840    2.7090901    0.5432493
H    -4.4654821    0.9065288    1.8686038
H    -3.1790790   -1.1058875    2.6299977
H     2.8433144   -2.5053590    1.8343217
H     2.0055263   -4.4394494    3.1405788
H    -0.4129731   -4.5643381    3.8304449
H    -1.9726005   -2.7281497    3.2068953
N    -0.6620770    0.5465688    1.0391254
C     0.4232149    0.7503603   -2.6191902
C     1.4166841    1.5477296   -3.2432736
C     1.1096203    2.3325225   -4.3616439
C    -0.2022105    2.3320446   -4.8574375
C    -1.2107086    1.5617863   -4.2621677
C    -0.8952720    0.7751300   -3.1468047
H     2.4446425    1.5412858   -2.8500957
H     1.8755802    2.9494154   -4.8565640
F    -0.5016656    3.0885787   -5.9302302
H    -2.2287345    1.5845994   -4.6811698
H    -1.6753528    0.1596039   -2.6721317
C     0.7167115   -0.1111110   -1.4981636
C     0.7573191   -1.1846136   -0.8280762
H     0.6802769   -2.2704970   -0.7650598
```

```
$vibrational spectrum
#  mode     symmetry     wave number   IR intensity    selection rules
#                         cm**(-1)        km/mol          IR     RAMAN
     1                         0.00         0.00000        -       -
     2                         0.00         0.00000        -       -
     3                         0.00         0.00000        -       -
     4                         0.00         0.00000        -       -
     5                         0.00         0.00000        -       -
     6                         0.00         0.00000        -       -
     7         a              15.26         0.30305       YES     YES
```

8	a	22.55	0.21713	YES	YES
9	a	36.75	0.03404	YES	YES
10	a	54.19	0.12635	YES	YES
11	a	68.66	0.12992	YES	YES
12	a	69.04	0.03680	YES	YES
13	a	85.76	1.19921	YES	YES
14	a	89.39	0.32021	YES	YES
15	a	98.28	0.65821	YES	YES
16	a	108.74	0.22748	YES	YES
17	a	111.52	0.15179	YES	YES
18	a	112.93	0.28324	YES	YES
19	a	121.44	0.12117	YES	YES
20	a	139.59	2.64905	YES	YES
21	a	158.76	3.46708	YES	YES
22	a	189.00	1.22604	YES	YES
23	a	214.26	1.85074	YES	YES
24	a	239.50	0.51428	YES	YES
25	a	259.39	6.47716	YES	YES
26	a	264.27	3.68135	YES	YES
27	a	284.68	0.09891	YES	YES
28	a	357.70	3.02508	YES	YES
29	a	378.20	28.05821	YES	YES
30	a	391.70	11.00793	YES	YES
31	a	409.93	0.75986	YES	YES
32	a	415.89	1.48373	YES	YES
33	a	419.65	11.81778	YES	YES
34	a	435.47	6.80079	YES	YES
35	a	451.76	20.06276	YES	YES
36	a	456.40	2.32529	YES	YES
37	a	461.08	1.55757	YES	YES
38	a	477.37	5.21520	YES	YES
39	a	486.93	2.06431	YES	YES
40	a	493.26	5.14778	YES	YES
41	a	502.04	11.11529	YES	YES
42	a	503.59	1.08353	YES	YES
43	a	526.90	38.61188	YES	YES
44	a	538.69	9.57228	YES	YES
45	a	550.87	7.09999	YES	YES
46	a	556.89	1.66527	YES	YES
47	a	609.50	33.01311	YES	YES
48	a	622.64	9.01751	YES	YES
49	a	625.26	5.49474	YES	YES
50	a	630.58	8.65305	YES	YES

2bf′

```
SCF Energy (au) BP86/SV(P)                    -2376.684012464
SCF Energy (au) PBE0/def2-TZVPP               -2376.261119915
SCF Energy (au) PBE0/def2-TZVPP               -2376.2683674155 (Toluene Correction)
Zero Point Energy (au)                        0.2819030
Chemical Potential (kJ mol-1)                 591.74
Dispersion Correction (au) PBE0/def2-TZVPP    -0.06372121
```

```
xyz coordinates
41

Mn    1.2369841    1.8112428    0.2458945
C     1.3710057    2.2239260    1.9979497
C     2.9973830    1.4946875    0.1231146
C     1.4070378    3.5547432   -0.2208095
O     1.5385251    4.6804844   -0.5020000
O     4.1374258    1.2590269    0.0458268
O     1.4653967    2.5035421    3.1234923
C    -1.4385143    0.7493141    0.9600496
C    -1.6425046    2.8281922   -0.1147058
C    -3.0389787    2.7999555   -0.0423265
C    -3.6527406    1.6883888    0.5621785
C    -2.8474098    0.6622517    1.0618302
C    -0.4931389   -0.2595865    1.4480186
C     0.8870150    0.0197608    1.2230263
C     1.8292920   -0.9052704    1.7195257
C     1.4267886   -2.0811428    2.3776020
C     0.0601603   -2.3574599    2.5696724
C    -0.8958064   -1.4457124    2.1080509
H    -1.1218721    3.6804176   -0.5784063
H    -3.6263729    3.6366933   -0.4506317
H    -4.7502280    1.6239560    0.6421708
H    -3.3044980   -0.2195121    1.5341995
H     2.9092528   -0.7158254    1.5984144
H     2.1890698   -2.7877433    2.7481177
H    -0.2575013   -3.2783312    3.0851389
H    -1.9647731   -1.6620028    2.2701512
N    -0.8574199    1.8421104    0.3700461
H     1.1178314    2.5991783   -2.6467347
C     1.0251692    1.8237949   -1.8842664
C     0.9074673    0.6019790   -1.5617475
C     0.6359327   -0.7934439   -1.8185723
C     0.0850213   -3.4861763   -2.3995169
C     1.3372732   -3.1407576   -1.8718695
C     1.6054862   -1.7987903   -1.5754792
C    -0.6198875   -1.1771431   -2.3554369
C    -0.8990480   -2.5194103   -2.6455898
F    -0.1785803   -4.7781744   -2.6779051
H     2.0861359   -3.9282089   -1.6949677
H     2.5847550   -1.5154101   -1.1615720
H    -1.3812274   -0.4052900   -2.5485932
H    -1.8699069   -2.8282538   -3.0633400
```

```
$vibrational spectrum
#  mode     symmetry     wave number   IR intensity    selection rules
#                         cm**(-1)        km/mol          IR     RAMAN
     1                       0.00        0.00000         -        -
     2                       0.00        0.00000         -        -
     3                       0.00        0.00000         -        -
     4                       0.00        0.00000         -        -
     5                       0.00        0.00000         -        -
     6                       0.00        0.00000         -        -
     7        a             15.39        0.27467        YES      YES
```

8	a	21.55	0.51847	YES	YES
9	a	44.50	0.11890	YES	YES
10	a	50.19	0.06929	YES	YES
11	a	63.50	0.12389	YES	YES
12	a	70.81	0.39479	YES	YES
13	a	86.26	0.60158	YES	YES
14	a	93.80	0.51872	YES	YES
15	a	96.25	0.54080	YES	YES
16	a	103.98	0.46765	YES	YES
17	a	111.32	0.04869	YES	YES
18	a	112.85	0.20982	YES	YES
19	a	127.43	0.18839	YES	YES
20	a	148.93	1.14170	YES	YES
21	a	165.79	2.17073	YES	YES
22	a	189.15	0.17288	YES	YES
23	a	218.42	0.44821	YES	YES
24	a	231.41	2.55714	YES	YES
25	a	255.37	1.15210	YES	YES
26	a	271.20	1.74803	YES	YES
27	a	284.92	0.21224	YES	YES
28	a	358.46	2.27539	YES	YES
29	a	382.81	26.61717	YES	YES
30	a	389.11	7.98426	YES	YES
31	a	409.08	0.41539	YES	YES
32	a	411.80	2.83525	YES	YES
33	a	420.64	1.59546	YES	YES
34	a	434.52	4.52181	YES	YES
35	a	448.98	7.51490	YES	YES
36	a	457.87	1.61266	YES	YES
37	a	468.81	5.63922	YES	YES
38	a	476.20	2.44933	YES	YES
39	a	488.08	1.56393	YES	YES
40	a	490.83	1.81398	YES	YES
41	a	499.72	3.43406	YES	YES
42	a	505.68	14.63655	YES	YES
43	a	529.19	23.69735	YES	YES
44	a	535.60	4.82454	YES	YES
45	a	557.17	2.54145	YES	YES
46	a	565.67	3.11213	YES	YES
47	a	613.82	45.67900	YES	YES
48	a	622.84	13.82057	YES	YES
49	a	625.83	15.93175	YES	YES
50	a	629.85	3.56307	YES	YES

2bg

```
SCF Energy (au) BP86/SV(P)                   -2614.328819566
SCF Energy (au) PBE0/def2-TZVPP              -2613.948640333
SCF Energy (au) PBE0/def2-TZVPP              -2613.9559675194 (Toluene Correction)
Zero Point Energy (au)                       0.2944290
Chemical Potential (kJ mol-1)                612.46
Dispersion Correction (au) PBE0/def2-TZVPP  -0.06476787

xyz coordinates
44

Mn    1.3766062   -0.0299156    1.0645268
C     1.8229581    0.4356415    2.7480525
C     3.0622173   -0.5265104    0.7094753
C     1.6638503    1.6585459    0.4552845
O     1.8874604    2.7516652    0.1141967
O     4.1554677   -0.8631109    0.4807096
O     2.1184501    0.7296068    3.8343661
C    -1.2766004   -0.6795420    2.2218831
C    -1.3620294    1.3794322    1.0940160
C    -2.7193760    1.5530719    1.3844939
C    -3.3748007    0.5573279    2.1295807
C    -2.6482706   -0.5605927    2.5468182
C    -0.4139833   -1.8012923    2.6080498
C     0.9421134   -1.7237824    2.1748488
C     1.8120399   -2.7718873    2.5409442
C     1.3560960   -3.8700920    3.2912667
C     0.0112576   -3.9433639    3.7031411
C    -0.8691605   -2.9102356    3.3632339
H    -0.8122469    2.1383073    0.5159744
H    -3.2432126    2.4549779    1.0324646
H    -4.4429880    0.6539621    2.3839604
H    -3.1391444   -1.3524911    3.1310133
H     2.8717417   -2.7431955    2.2367681
H     2.0593057   -4.6767524    3.5601350
H    -0.3465864   -4.8032959    4.2922161
H    -1.9196367   -2.9708702    3.6924543
N    -0.6527397    0.3016419    1.4946763
C     0.4549691    0.5132800   -2.1782245
C     1.4807382    1.2754454   -2.7931028
C     1.2051413    2.0684467   -3.9116446
C    -0.1007871    2.1280631   -4.4365857
C    -1.1299957    1.3826546   -3.8324164
C    -0.8568091    0.5831906   -2.7158327
H     2.5032306    1.2358872   -2.3878685
H     2.0116705    2.6551033   -4.3782887
C    -0.3766414    2.9532026   -5.6723436
H    -2.1527451    1.4310007   -4.2369787
H    -1.6623480   -0.0018611   -2.2452578
C     0.7144502   -0.3458806   -1.0471444
C     0.7396745   -1.4246914   -0.3831151
H     0.6447374   -2.5096058   -0.3253393
F     0.3742405    4.0809757   -5.7000792
F    -1.6778780    3.3258943   -5.7536733
F    -0.0904370    2.2616444   -6.8066550

$vibrational spectrum
#  mode     symmetry     wave number   IR intensity    selection rules
#                         cm**(-1)        km/mol          IR     RAMAN
     1                        0.00         0.00000         -       -
     2                        0.00         0.00000         -       -
     3                        0.00         0.00000         -       -
     4                        0.00         0.00000         -       -
```

5		0.00	0.00000	-	-
6		0.00	0.00000	-	-
7	a	12.17	0.41522	YES	YES
8	a	20.39	0.15130	YES	YES
9	a	35.27	0.10531	YES	YES
10	a	37.43	0.03820	YES	YES
11	a	49.21	0.09937	YES	YES
12	a	62.82	0.21459	YES	YES
13	a	69.00	0.07337	YES	YES
14	a	83.96	0.19366	YES	YES
15	a	85.66	1.32995	YES	YES
16	a	92.84	0.36282	YES	YES
17	a	95.29	0.95302	YES	YES
18	a	106.47	0.10456	YES	YES
19	a	109.91	0.10488	YES	YES
20	a	113.97	0.26757	YES	YES
21	a	122.89	0.94308	YES	YES
22	a	152.58	1.00454	YES	YES
23	a	178.29	2.11843	YES	YES
24	a	187.68	2.07863	YES	YES
25	a	204.44	0.65822	YES	YES
26	a	223.75	0.27808	YES	YES
27	a	240.54	0.77371	YES	YES
28	a	260.93	0.55345	YES	YES
29	a	280.59	0.60115	YES	YES
30	a	291.71	0.57093	YES	YES
31	a	320.94	39.44566	YES	YES
32	a	358.15	1.90390	YES	YES
33	a	382.50	3.39938	YES	YES
34	a	397.47	9.23041	YES	YES
35	a	403.54	6.39425	YES	YES
36	a	417.84	10.02122	YES	YES
37	a	429.45	1.95527	YES	YES
38	a	446.92	4.61272	YES	YES
39	a	456.06	1.37970	YES	YES
40	a	456.24	8.85877	YES	YES
41	a	473.87	3.19751	YES	YES
42	a	485.82	2.00936	YES	YES
43	a	491.78	5.65688	YES	YES
44	a	496.06	1.61189	YES	YES
45	a	501.48	5.88190	YES	YES
46	a	515.47	9.01132	YES	YES
47	a	535.60	26.33736	YES	YES
48	a	548.45	5.41518	YES	YES
49	a	556.91	3.03587	YES	YES
50	a	561.76	8.27135	YES	YES

2bg′

```
SCF Energy (au) BP86/SV(P)                   -2614.327168892
SCF Energy (au) PBE0/def2-TZVPP              -2613.947026262
SCF Energy (au) PBE0/def2-TZVPP              -2613.9542738044 (Toluene Correction)
Zero Point Energy (au)                       0.2946089
Chemical Potential (kJ mol-1)                615.65
Dispersion Correction (au) PBE0/def2-TZVPP -0.06779443

xyz coordinates
44

Mn    1.2573837    2.1963969    0.4486841
C     1.3531009    2.5665551    2.2135486
C     3.0245241    1.9082488    0.3486739
C     1.4107084    3.9540834    0.0305177
O     1.5310693    5.0887235   -0.2159537
O     4.1686241    1.6910536    0.2840612
O     1.4228473    2.8195984    3.3467836
C    -1.4121583    1.0754608    1.0929445
C    -1.6333439    3.1754795    0.0628049
C    -3.0302405    3.1192127    0.1047146
C    -3.6349183    1.9818055    0.6689859
C    -2.8208450    0.9593536    1.1620807
C    -0.4584825    0.0722705    1.5772752
C     0.9207441    0.3822284    1.3919805
C     1.8703617   -0.5376373    1.8828441
C     1.4759048   -1.7400836    2.4966351
C     0.1110156   -2.0476951    2.6480375
C    -0.8522662   -1.1404826    2.1924812
H    -1.1195922    4.0480721   -0.3696454
H    -3.6251749    3.9540940   -0.2963112
H    -4.7323954    1.8944362    0.7229211
H    -3.2707823    0.0580324    1.6033314
H     2.9487511   -0.3250674    1.7901990
H     2.2430310   -2.4437773    2.8624149
H    -0.2003424   -2.9907485    3.1256045
H    -1.9199092   -1.3831785    2.3217010
N    -0.8401231    2.1925027    0.5405406
H     1.1982519    3.0457681   -2.4312254
C     1.0905043    2.2587945   -1.6825979
C     0.9622207    1.0325429   -1.3807726
C     0.6868258   -0.3602690   -1.6397991
C     0.0939562   -3.0796722   -2.1473174
C     1.3701303   -2.7079213   -1.6823147
C     1.6628496   -1.3646825   -1.4238984
C    -0.5884926   -0.7447573   -2.1270631
C    -0.8805765   -2.0905795   -2.3769088
C    -0.2506350   -4.5380390   -2.3353709
H     2.1393655   -3.4781489   -1.5177800
H     2.6599586   -1.0783748   -1.0571861
H    -1.3505437    0.0288712   -2.3095423
H    -1.8735156   -2.3768497   -2.7570294
F     0.8329323   -5.2770393   -2.6769699
F    -1.1879230   -4.7182721   -3.2986776
F    -0.7528007   -5.0803084   -1.1934013

$vibrational spectrum
#  mode     symmetry     wave number   IR intensity    selection rules
#                         cm**(-1)        km/mol         IR     RAMAN
     1                        0.00         0.00000        -       -
     2                        0.00         0.00000        -       -
     3                        0.00         0.00000        -       -
     4                        0.00         0.00000        -       -
```

5		0.00	0.00000	-	-
6		0.00	0.00000	-	-
7	a	14.98	0.63488	YES	YES
8	a	24.73	0.22465	YES	YES
9	a	43.41	0.03090	YES	YES
10	a	47.63	0.31629	YES	YES
11	a	49.76	0.06600	YES	YES
12	a	61.87	0.26106	YES	YES
13	a	67.06	0.16659	YES	YES
14	a	82.71	0.48376	YES	YES
15	a	89.55	0.21796	YES	YES
16	a	94.33	0.63058	YES	YES
17	a	98.47	0.91191	YES	YES
18	a	103.09	0.30264	YES	YES
19	a	109.36	0.12101	YES	YES
20	a	118.51	0.23764	YES	YES
21	a	126.63	0.43690	YES	YES
22	a	159.54	0.91062	YES	YES
23	a	184.02	0.14154	YES	YES
24	a	193.52	0.22917	YES	YES
25	a	210.22	0.93389	YES	YES
26	a	217.04	0.33259	YES	YES
27	a	235.08	1.62107	YES	YES
28	a	264.01	0.57022	YES	YES
29	a	282.63	0.22548	YES	YES
30	a	298.91	0.34890	YES	YES
31	a	316.84	22.44646	YES	YES
32	a	358.59	1.90104	YES	YES
33	a	384.74	1.73648	YES	YES
34	a	399.82	0.67506	YES	YES
35	a	402.85	10.35245	YES	YES
36	a	417.48	3.87632	YES	YES
37	a	430.27	2.50205	YES	YES
38	a	438.67	2.11925	YES	YES
39	a	457.39	1.48654	YES	YES
40	a	465.98	1.18742	YES	YES
41	a	470.47	2.76870	YES	YES
42	a	485.67	6.06921	YES	YES
43	a	489.03	1.53234	YES	YES
44	a	497.56	4.15803	YES	YES
45	a	502.00	11.68884	YES	YES
46	a	521.01	4.96104	YES	YES
47	a	528.15	14.73262	YES	YES
48	a	556.28	1.44075	YES	YES
49	a	558.45	2.58503	YES	YES
50	a	566.31	2.08477	YES	YES

2bh

```
SCF Energy (au) BP86/SV(P)                    -2505.243220756
SCF Energy (au) PBE0/def2-TZVPP               -2504.795978988
SCF Energy (au) PBE0/def2-TZVPP               -2504.8052396137 (Toluene Correction)
Zero Point Energy (au)                        0.3311844
Chemical Potential (kJ mol-1)                 706.41
Dispersion Correction (au) PBE0/def2-TZVPP -0.06732812

xyz coordinates
47

Mn    1.3037687   -0.3393686    1.5642927
C     1.7730387    0.1344454    3.2388633
C     2.9894863   -0.8119268    1.1812376
C     1.5546961    1.3527175    0.9477769
O     1.7573715    2.4484425    0.6023689
O     4.0837222   -1.1330871    0.9345719
O     2.0847189    0.4335773    4.3194731
C    -1.3138688   -1.0269365    2.7797729
C    -1.4579333    1.0217737    1.6394196
C    -2.8103401    1.1770946    1.9618594
C    -3.4317669    0.1781784    2.7313711
C    -2.6784524   -0.9257404    3.1383201
C    -0.4265261   -2.1349737    3.1495659
C     0.9175908   -2.0393267    2.6841830
C     1.8101083   -3.0749629    3.0315404
C     1.3873740   -4.1777526    3.7946080
C     0.0540064   -4.2683357    4.2389227
C    -0.8483498   -3.2481047    3.9178066
H    -0.9341412    1.7839641    1.0418882
H    -3.3566609    2.0677959    1.6155462
H    -4.4945293    0.2610368    3.0119807
H    -3.1425832   -1.7205382    3.7401988
H     2.8618655   -3.0326314    2.7023972
H     2.1077454   -4.9742865    4.0480141
H    -0.2779135   -5.1315735    4.8383119
H    -1.8898483   -3.3223666    4.2718269
N    -0.7224954   -0.0414370    2.0314249
C     0.3817212    0.2025585   -1.6825569
C     1.4305623    0.9225264   -2.3113210
C     1.1772920    1.7120587   -3.4373745
C    -0.1314288    1.8162960   -3.9587425
C    -1.1811545    1.1092983   -3.3339399
C    -0.9323909    0.3121893   -2.2126835
H     2.4533471    0.8470271   -1.9109724
H     1.9984106    2.2594003   -3.9235976
C    -0.4621050    2.6479676   -5.1560877
H    -2.1956412    1.1981318   -3.7536394
H    -1.7525776   -0.2447689   -1.7327883
C     0.6246510   -0.6587717   -0.5500646
C     0.6522165   -1.7324185    0.1221244
H     0.5521967   -2.8167169    0.1840923
O     0.6347577    3.2637250   -5.6739877
O    -1.5811101    2.7704014   -5.6290021
C     0.3952956    4.0772873   -6.8291640
H     1.3816802    4.4905983   -7.1168170
H    -0.3167022    4.8971929   -6.5931624
H    -0.0291043    3.4703400   -7.6578580

$vibrational spectrum
#  mode     symmetry     wave number   IR intensity    selection rules
#                          cm**(-1)        km/mol          IR     RAMAN
     1                          0.00         0.00000        -       -
```

2		0.00	0.00000	-	-
3		0.00	0.00000	-	-
4		0.00	0.00000	-	-
5		0.00	0.00000	-	-
6		0.00	0.00000	-	-
7	a	12.57	0.40934	YES	YES
8	a	21.74	0.40464	YES	YES
9	a	27.08	1.41359	YES	YES
10	a	46.22	0.15075	YES	YES
11	a	59.22	0.08189	YES	YES
12	a	65.99	0.43548	YES	YES
13	a	72.96	0.34214	YES	YES
14	a	81.48	0.30844	YES	YES
15	a	84.43	1.94921	YES	YES
16	a	90.25	0.19612	YES	YES
17	a	93.42	0.21922	YES	YES
18	a	103.84	0.38580	YES	YES
19	a	109.74	0.07931	YES	YES
20	a	113.53	0.35221	YES	YES
21	a	120.73	0.30032	YES	YES
22	a	125.90	0.91882	YES	YES
23	a	147.48	1.71613	YES	YES
24	a	152.66	4.50491	YES	YES
25	a	177.22	1.29846	YES	YES
26	a	188.89	1.54772	YES	YES
27	a	209.11	1.48568	YES	YES
28	a	234.98	0.55588	YES	YES
29	a	245.53	4.50037	YES	YES
30	a	262.35	0.79599	YES	YES
31	a	283.14	0.95130	YES	YES
32	a	311.13	27.87040	YES	YES
33	a	327.93	11.67023	YES	YES
34	a	350.73	23.39475	YES	YES
35	a	359.49	5.82019	YES	YES
36	a	404.30	0.02491	YES	YES
37	a	416.19	3.06781	YES	YES
38	a	426.53	2.24478	YES	YES
39	a	440.49	1.77115	YES	YES
40	a	449.10	1.71625	YES	YES
41	a	455.95	1.03867	YES	YES
42	a	457.74	24.14923	YES	YES
43	a	473.30	2.85469	YES	YES
44	a	485.96	1.78627	YES	YES
45	a	491.68	6.24711	YES	YES
46	a	501.47	4.97107	YES	YES
47	a	514.16	12.33024	YES	YES
48	a	525.09	28.57055	YES	YES
49	a	538.51	13.49851	YES	YES
50	a	549.73	5.41364	YES	YES

2bh′

SCF Energy (au) BP86/SV(P) -2505.241127470
SCF Energy (au) PBE0/def2-TZVPP -2504.793700599
SCF Energy (au) PBE0/def2-TZVPP -2504.8031262880 (Toluene Correction)
Zero Point Energy (au) 0.3312181
Chemical Potential (kJ mol^{-1}) 706.73
Dispersion Correction (au) PBE0/def2-TZVPP -0.06985329

xyz coordinates
47

Mn	1.3525788	2.6404515	0.6752257
C	1.4623236	3.0505351	2.4306510
C	3.1180559	2.3426824	0.5704972
C	1.5125479	4.3868787	0.2159357
O	1.6366598	5.5146486	-0.0592851
O	4.2612268	2.1210474	0.5048932
O	1.5409259	3.3295203	3.5572676
C	-1.3186215	1.5605827	1.3798475
C	-1.5332292	3.6333060	0.2947321
C	-2.9296576	3.5961481	0.3631720
C	-3.5376511	2.4833359	0.9711769
C	-2.7271811	1.4647459	1.4779097
C	-0.3682498	0.5597142	1.8746643
C	1.0111152	0.8482912	1.6580389
C	1.9583656	-0.0697541	2.1565151
C	1.5609619	-1.2495664	2.8106551
C	0.1954744	-1.5350771	2.9966655
C	-0.7651302	-0.6299190	2.5318795
H	-1.0168526	4.4866881	-0.1716124
H	-3.5214392	4.4269324	-0.0508772
H	-4.6349005	2.4121097	1.0484499
H	-3.1798632	0.5823249	1.9532209
H	3.0373138	0.1269373	2.0393990
H	2.3263312	-1.9524765	3.1813687
H	-0.1177088	-2.4595193	3.5083338
H	-1.8332349	-0.8548593	2.6874203
N	-0.7433560	2.6546985	0.7866600
H	1.2544836	3.4323463	-2.2177885
C	1.1606076	2.6610036	-1.4510573
C	1.0487005	1.4378508	-1.1274324
C	0.8093861	0.0385225	-1.3890164
C	0.3167967	-2.6897427	-1.9836557
C	1.5686989	-2.2825122	-1.4724613
C	1.8129129	-0.9403944	-1.1709397
C	-0.4473827	-0.3759304	-1.9026725
C	-0.6896091	-1.7225133	-2.1952754
C	0.1185604	-4.1403013	-2.2799951
H	2.3449415	-3.0478617	-1.3160285
H	2.7914949	-0.6294426	-0.7751307
H	-1.2314324	0.3781846	-2.0747522
H	-1.6651795	-2.0356814	-2.5961769
O	-1.1267215	-4.4022476	-2.7663730
O	0.9619567	-5.0071900	-2.1138995
C	-1.3865221	-5.7761092	-3.0780381
H	-0.6831782	-6.1448367	-3.8557727
H	-2.4287497	-5.8093542	-3.4516302
H	-1.2765696	-6.4141971	-2.1747089

$vibrational spectrum

# mode	symmetry	wave number cm**(-1)	IR intensity km/mol	selection rules IR	RAMAN
1		0.00	0.00000	-	-
2		0.00	0.00000	-	-
3		0.00	0.00000	-	-
4		0.00	0.00000	-	-
5		0.00	0.00000	-	-
6		0.00	0.00000	-	-
7	a	12.77	0.48046	YES	YES
8	a	16.49	0.07359	YES	YES
9	a	34.31	0.26905	YES	YES
10	a	48.86	0.02328	YES	YES
11	a	55.73	1.09007	YES	YES
12	a	63.10	0.70619	YES	YES
13	a	67.93	0.07690	YES	YES
14	a	82.43	0.35489	YES	YES
15	a	88.48	0.45152	YES	YES
16	a	90.74	0.41964	YES	YES
17	a	96.71	0.40586	YES	YES
18	a	99.94	0.43111	YES	YES
19	a	107.38	0.34149	YES	YES
20	a	114.78	0.64331	YES	YES
21	a	116.87	0.28711	YES	YES
22	a	123.33	0.47748	YES	YES
23	a	151.43	2.54072	YES	YES
24	a	158.10	1.69009	YES	YES
25	a	184.44	0.56521	YES	YES
26	a	191.79	0.56599	YES	YES
27	a	216.79	0.56283	YES	YES
28	a	226.23	2.33570	YES	YES
29	a	241.58	0.48368	YES	YES
30	a	265.96	0.88216	YES	YES
31	a	283.59	0.15454	YES	YES
32	a	310.54	23.14858	YES	YES
33	a	326.65	1.88924	YES	YES
34	a	357.04	8.70207	YES	YES
35	a	361.20	13.57125	YES	YES
36	a	403.29	0.05072	YES	YES
37	a	416.59	9.13494	YES	YES
38	a	422.54	1.21557	YES	YES
39	a	434.43	5.46699	YES	YES
40	a	457.11	2.85989	YES	YES
41	a	458.87	3.97714	YES	YES
42	a	466.11	1.90131	YES	YES
43	a	470.44	2.27312	YES	YES
44	a	486.24	6.29781	YES	YES
45	a	489.75	3.99537	YES	YES
46	a	500.49	2.36214	YES	YES
47	a	508.50	10.92025	YES	YES
48	a	526.16	19.44830	YES	YES
49	a	534.84	2.05306	YES	YES
50	a	556.88	1.91812	YES	YES

2bi

SCF Energy (au) BP86/SV(P)	-2508.412127961
SCF Energy (au) PBE0/def2-TZVPP	-2507.929859714
SCF Energy (au) PBE0/def2-TZVPP	-2507.9381342801 (Toluene Correction)
Zero Point Energy (au)	0.3689319
Chemical Potential (kJ mol^{-1})	802.70
Dispersion Correction (au) PBE0/def2-TZVPP	-0.08008127

xyz coordinates
51

Mn	1.2130299	1.1283460	0.7677901
C	1.7035251	1.6930919	2.4075242
C	2.9060224	0.7196004	0.3489977
C	1.3402844	2.8290054	0.1489828
O	1.4529387	3.9427431	-0.1788935
O	4.0048144	0.4309846	0.0798274
O	2.0398828	2.0723864	3.4558032
C	-1.3170678	0.3238844	2.0891977
C	-1.6520221	2.2439038	0.7770437
C	-3.0119197	2.3031509	1.0993219
C	-3.5366106	1.3238318	1.9611220
C	-2.6842758	0.3326735	2.4536215
C	-0.3319129	-0.6649198	2.5369774
C	0.9908736	-0.4985782	2.0326552
C	1.9782768	-1.4050739	2.4707026
C	1.6657888	-2.4618788	3.3435286
C	0.3502888	-2.6308128	3.8149470
C	-0.6441053	-1.7318737	3.4136953
H	-1.2015770	2.9926609	0.1072473
H	-3.6387367	3.1062909	0.6823404
H	-4.6015919	1.3339608	2.2454455
H	-3.0718391	-0.4473817	3.1247977
H	3.0216985	-1.2941308	2.1310262
H	2.4590368	-3.1596738	3.6619684
H	0.1037007	-3.4586847	4.4994147
H	-1.6710583	-1.8636835	3.7923921
N	-0.8207181	1.2920030	1.2544310
C	0.4207025	1.6500918	-2.5074411
C	1.4867883	2.4143679	-3.0457378
C	1.3169154	3.1360790	-4.2354252
C	0.0808729	3.1232991	-4.9064612
C	-0.9868061	2.3750914	-4.3801494
C	-0.8224232	1.6450879	-3.1936522
H	2.4588884	2.4257396	-2.5283637
H	2.1609446	3.7168114	-4.6429110
H	-0.0496867	3.6969551	-5.8387960
H	-1.9592198	2.3570001	-4.9000822
H	-1.6581221	1.0554551	-2.7841453
C	0.5779432	0.8415585	-1.3142645
C	0.5270337	-0.2875373	-0.7223688
C	0.2609239	-1.7109594	-0.7565203
C	-0.2803551	-4.4856857	-0.9456125
C	1.0329260	-4.0329921	-0.7238185
C	1.3037063	-2.6616307	-0.6212917
C	-1.0599086	-2.1783404	-0.9771882
C	-1.3244028	-3.5529628	-1.0703561
H	-0.4891628	-5.5657011	-1.0214352
H	1.8581302	-4.7581454	-0.6286880
H	2.3329935	-2.3096110	-0.4541603
H	-1.8783950	-1.4475910	-1.0773034
H	-2.3570132	-3.8982063	-1.2457354

$vibrational spectrum

# mode	symmetry	wave number cm**(-1)	IR intensity km/mol	selection rules IR	selection rules RAMAN
1		0.00	0.00000	-	-
2		0.00	0.00000	-	-
3		0.00	0.00000	-	-
4		0.00	0.00000	-	-
5		0.00	0.00000	-	-
6		0.00	0.00000	-	-
7	a	11.30	0.22300	YES	YES
8	a	22.06	0.05960	YES	YES
9	a	26.37	0.05122	YES	YES
10	a	29.53	0.26518	YES	YES
11	a	42.86	0.19185	YES	YES
12	a	47.27	0.19769	YES	YES
13	a	58.61	0.22167	YES	YES
14	a	69.78	0.25509	YES	YES
15	a	77.43	0.33119	YES	YES
16	a	86.68	0.52791	YES	YES
17	a	93.21	0.55682	YES	YES
18	a	99.48	0.43536	YES	YES
19	a	110.27	0.03955	YES	YES
20	a	112.40	0.07220	YES	YES
21	a	118.74	0.41670	YES	YES
22	a	128.61	0.09602	YES	YES
23	a	155.09	0.23330	YES	YES
24	a	172.92	0.64525	YES	YES
25	a	184.50	2.96286	YES	YES
26	a	190.36	1.46917	YES	YES
27	a	217.37	2.47697	YES	YES
28	a	227.91	3.62925	YES	YES
29	a	246.59	2.66370	YES	YES
30	a	263.96	0.15492	YES	YES
31	a	285.78	0.04935	YES	YES
32	a	357.97	2.19617	YES	YES
33	a	380.42	15.73501	YES	YES
34	a	396.16	3.45386	YES	YES
35	a	400.56	0.70936	YES	YES
36	a	402.72	0.46689	YES	YES
37	a	419.58	7.53622	YES	YES
38	a	434.67	7.51619	YES	YES
39	a	447.78	18.79443	YES	YES
40	a	455.69	1.81735	YES	YES
41	a	466.54	3.39152	YES	YES
42	a	473.94	4.02064	YES	YES
43	a	487.42	1.59525	YES	YES
44	a	494.62	8.89332	YES	YES
45	a	500.57	40.66585	YES	YES
46	a	510.17	18.38623	YES	YES
47	a	532.27	6.91702	YES	YES
48	a	541.86	10.49425	YES	YES
49	a	546.65	14.41346	YES	YES
50	a	556.32	1.85781	YES	YES

2bj

SCF Energy (au) BP86/SV(P) -2360.882425609
SCF Energy (au) PBE0/def2-TZVPP -2360.449384567
SCF Energy (au) PBE0/def2-TZVPP -2360.4555509522 (Toluene Correction)
Zero Point Energy (au) 0.4312242
Chemical Potential (kJ mol^{-1}) 961.18
Dispersion Correction (au) PBE0/def2-TZVPP -0.07158498

xyz coordinates
55

Mn	0.9063604	1.1654989	1.2513929
C	1.4579883	1.7774516	2.8523784
C	2.5778462	0.7095393	0.8022817
C	1.0471178	2.8159490	0.5310960
O	1.1618663	3.9010142	0.1081574
O	3.6636985	0.3933969	0.5122264
O	1.8356109	2.1749044	3.8800338
C	-1.6068028	0.4517863	2.6622762
C	-1.9209358	2.3794980	1.3603483
C	-3.2626150	2.4940528	1.7393193
C	-3.7889360	1.5380768	2.6254461
C	-2.9554606	0.5154195	3.0851400
C	-0.6429715	-0.5760285	3.0695296
C	0.6614645	-0.4621593	2.5021412
C	1.6210372	-1.4248806	2.8842198
C	1.3013794	-2.4730750	3.7653559
C	0.0050144	-2.5810691	4.3047530
C	-0.9629187	-1.6313920	3.9585385
H	-1.4682090	3.1144091	0.6768561
H	-3.8740846	3.3216238	1.3478960
H	-4.8395069	1.5912199	2.9550566
H	-3.3440641	-0.2459782	3.7769777
H	2.6490455	-1.3666327	2.4885330
H	2.0741428	-3.2123366	4.0379284
H	-0.2459702	-3.3996086	4.9989108
H	-1.9742205	-1.7143196	4.3901050
N	-1.1078899	1.3957218	1.8015117
C	0.1463747	1.6673289	-2.0651955
C	0.2249000	0.8325196	-0.8403622
C	0.1834660	-0.2677396	-0.2129212
C	-0.0838458	-1.7237446	-0.1591217
H	0.8271177	-2.2503474	0.2092813
C	-0.5210201	-2.3071547	-1.5199947
H	-0.8611075	-1.9334766	0.6115687
C	-0.7974492	-3.8183739	-1.4622855
H	0.2702275	-2.0995950	-2.2773694
H	-1.4349885	-1.7730181	-1.8707999
C	-1.2269150	-4.4107003	-2.8113143
H	-1.5858337	-4.0181631	-0.6987829
H	0.1167171	-4.3439965	-1.0984921
H	-1.4183266	-5.5036238	-2.7325177
H	-0.4421516	-4.2618467	-3.5870862
H	-2.1600508	-3.9322296	-3.1856164
C	1.5045309	1.8713812	-2.7748815
H	-0.5587833	1.1609294	-2.7667554
H	-0.2988621	2.6637806	-1.8441008
H	2.2194946	2.3506684	-2.0687930
C	1.3891197	2.7256069	-4.0470473
H	1.9342444	0.8736357	-3.0233639
C	2.7307898	2.9294680	-4.7625803
H	0.9547438	3.7182795	-3.7817327
H	0.6615254	2.2502414	-4.7469750

```
H     3.4687910    3.4359572   -4.1005359
H     2.6138692    3.5544444   -5.6753434
H     3.1754356    1.9576868   -5.0752911

$vibrational spectrum
#  mode     symmetry     wave number   IR intensity    selection rules
#                         cm**(-1)        km/mol         IR     RAMAN
     1                      0.00         0.00000         -       -
     2                      0.00         0.00000         -       -
     3                      0.00         0.00000         -       -
     4                      0.00         0.00000         -       -
     5                      0.00         0.00000         -       -
     6                      0.00         0.00000         -       -
     7         a           11.02         0.17245        YES     YES
     8         a           25.23         0.14043        YES     YES
     9         a           27.66         0.17493        YES     YES
    10         a           40.79         0.02801        YES     YES
    11         a           48.69         0.22577        YES     YES
    12         a           55.13         0.08372        YES     YES
    13         a           61.84         0.07792        YES     YES
    14         a           63.89         0.05944        YES     YES
    15         a           73.91         0.05961        YES     YES
    16         a           84.70         0.87413        YES     YES
    17         a           86.28         0.56138        YES     YES
    18         a           91.79         0.23567        YES     YES
    19         a          101.15         0.08415        YES     YES
    20         a          105.03         0.28350        YES     YES
    21         a          105.92         0.16061        YES     YES
    22         a          115.10         0.13407        YES     YES
    23         a          123.42         0.37657        YES     YES
    24         a          127.51         0.13969        YES     YES
    25         a          134.65         0.14269        YES     YES
    26         a          167.09         1.19662        YES     YES
    27         a          185.09         1.62408        YES     YES
    28         a          192.02         1.72794        YES     YES
    29         a          207.29         0.25131        YES     YES
    30         a          212.04         0.94338        YES     YES
    31         a          230.67         0.44577        YES     YES
    32         a          245.13         0.09361        YES     YES
    33         a          251.99         0.09030        YES     YES
    34         a          257.44         0.71190        YES     YES
    35         a          284.92         0.04044        YES     YES
    36         a          287.84         1.99290        YES     YES
    37         a          342.65         1.35575        YES     YES
    38         a          351.28        10.47142        YES     YES
    39         a          358.57         2.50273        YES     YES
    40         a          377.52         7.71193        YES     YES
    41         a          413.42         0.40739        YES     YES
    42         a          417.98         3.93799        YES     YES
    43         a          436.39         0.17315        YES     YES
    44         a          456.70         1.43676        YES     YES
    45         a          459.24         3.11548        YES     YES
    46         a          472.34         2.92312        YES     YES
    47         a          487.20         4.26455        YES     YES
    48         a          493.34         0.83376        YES     YES
    49         a          504.92         1.30037        YES     YES
    50         a          511.34         0.08654        YES     YES
```

2ca

```
SCF Energy (au) BP86/SV(P)                  -2678.190493143
SCF Energy (au) PBE0/def2-TZVPP             -2677.706850638
SCF Energy (au) PBE0/def2-TZVPP             -2677.7183769724 (Toluene Correction)
Zero Point Energy (au)                      0.4157036
Chemical Potential (kJ mol-1)               911.24
Dispersion Correction (au) PBE0/def2-TZVPP  -0.08425883

xyz coordinates
57

Mn    0.7905336    1.5285988   -0.7423591
C     1.3273161    1.7047316    0.9650669
C     2.4456986    0.9692036   -1.2253026
C     1.2218679    3.2420649   -1.1491495
O     1.5213430    4.3518560   -1.3572636
O     3.5159990    0.7182341   -1.6022172
O     1.6847253    1.7901848    2.0709940
C    -1.9172557    1.0193716    0.3528721
C    -1.7957953    3.1873913   -0.5318246
C    -3.1303060    3.4572047   -0.2048991
C    -3.8817482    2.4552653    0.4318321
C    -3.2678746    1.2300441    0.7097169
C    -1.1664315   -0.2164499    0.5949210
C     0.1556683   -0.2054871    0.1489748
C     0.9967886   -1.3733598    0.3826172
N     0.3757456   -2.4857002    1.0476719
C    -0.9469338   -2.4827290    1.4670707
C    -1.7248080   -1.3636875    1.2467454
H    -1.1767336    3.9503746   -1.0275713
H    -3.5624970    4.4401795   -0.4476092
H    -4.9345600    2.6283193    0.7088086
H    -3.8313851    0.4263969    1.2057034
O     2.1917129   -1.4589280    0.0571630
C    -1.4998894   -3.7096034    2.1450369
H    -2.7692106   -1.3801603    1.5893197
N    -1.1933158    2.0084472   -0.2649113
C    -0.2026858    2.5515868   -3.8505661
C     0.8330593    3.3355169   -4.4204606
C     0.5392223    4.3032118   -5.3907774
C    -0.7879149    4.5174378   -5.8053061
C    -1.8237181    3.7496072   -5.2446583
C    -1.5387683    2.7755920   -4.2763370
H     1.8734261    3.1710737   -4.0999638
H     1.3574875    4.8993708   -5.8272260
H    -1.0141382    5.2828874   -6.5658297
H    -2.8665484    3.9080653   -5.5667153
H    -2.3482659    2.1684774   -3.8410303
C     0.0717449    1.5172662   -2.8807799
C     0.0979632    0.3619270   -2.3637414
H    -0.0035840   -0.7198586   -2.4531376
H    -1.5173194   -4.5908682    1.4635559
H    -2.5420262   -3.5195711    2.4730421
H    -0.9011095   -3.9959174    3.0374664
C     1.2519414   -3.6507605    1.2827453
C     1.8231970   -3.7377665    2.6904001
H     2.0819419   -3.5235044    0.5560890
H     0.7002069   -4.5790914    1.0321895
C     2.9472178   -3.9354974    5.2820534
C     2.3163704   -5.0476178    4.7009390
C     1.7566335   -4.9455809    3.4153647
C     2.4693801   -2.6285701    3.2789892
C     3.0218256   -2.7272481    4.5658847
```

```
H     3.3831164   -4.0094872    6.2921715
H     2.2541763   -6.0011177    5.2516107
H     1.2615839   -5.8247528    2.9659924
H     2.5537513   -1.6850764    2.7160355
H     3.5231786   -1.8514973    5.0105932

$vibrational spectrum
#  mode     symmetry     wave number   IR intensity    selection rules
#                         cm**(-1)        km/mol         IR     RAMAN
     1                        0.00         0.00000       -       -
     2                        0.00         0.00000       -       -
     3                        0.00         0.00000       -       -
     4                        0.00         0.00000       -       -
     5                        0.00         0.00000       -       -
     6                        0.00         0.00000       -       -
     7         a             11.75         0.05816      YES     YES
     8         a             14.17         0.44665      YES     YES
     9         a             18.27         0.27503      YES     YES
    10         a             25.24         0.43345      YES     YES
    11         a             28.41         0.17720      YES     YES
    12         a             33.39         0.16288      YES     YES
    13         a             52.56         2.02624      YES     YES
    14         a             61.46         0.06639      YES     YES
    15         a             70.66         0.31006      YES     YES
    16         a             77.87         0.45878      YES     YES
    17         a             82.82         0.65931      YES     YES
    18         a             91.77         2.72653      YES     YES
    19         a             98.13         0.53621      YES     YES
    20         a            101.45         0.06260      YES     YES
    21         a            111.21         0.12231      YES     YES
    22         a            118.61         0.89381      YES     YES
    23         a            121.91         1.04289      YES     YES
    24         a            152.08         0.91687      YES     YES
    25         a            152.54         0.72509      YES     YES
    26         a            160.88         4.90822      YES     YES
    27         a            183.22         0.71152      YES     YES
    28         a            194.04         1.37194      YES     YES
    29         a            208.22         1.82511      YES     YES
    30         a            222.93         0.28827      YES     YES
    31         a            244.70         1.18420      YES     YES
    32         a            247.38         0.73847      YES     YES
    33         a            261.67         1.67246      YES     YES
    34         a            279.46         0.50349      YES     YES
    35         a            311.39         3.41575      YES     YES
    36         a            323.37        15.15492      YES     YES
    37         a            333.50        13.91151      YES     YES
    38         a            369.01         6.10197      YES     YES
    39         a            388.19         1.01913      YES     YES
    40         a            401.34         0.06661      YES     YES
    41         a            402.35         0.33753      YES     YES
    42         a            416.94        18.50278      YES     YES
    43         a            429.83         3.80817      YES     YES
    44         a            439.12        14.05357      YES     YES
    45         a            455.41         8.65813      YES     YES
    46         a            462.46        13.72343      YES     YES
    47         a            465.15         2.29291      YES     YES
    48         a            470.80         0.71839      YES     YES
    49         a            479.01         8.66726      YES     YES
    50         a            486.70         6.50033      YES     YES
```

2ca′

```
SCF Energy (au) BP86/SV(P)                     -2678.190378706
SCF Energy (au) PBE0/def2-TZVPP                -2677.706376595
SCF Energy (au) PBE0/def2-TZVPP                -2677.7174819824 (Toluene Correction)
Zero Point Energy (au)                         0.4159746
Chemical Potential (kJ mol-1)                  916.06
Dispersion Correction (au) PBE0/def2-TZVPP    -0.08639897
```

xyz coordinates
57

```
Mn    0.7961401    2.5847105   -1.0851902
C     1.2500491    2.7717503    0.6500830
C     2.4813310    2.0544182   -1.5070732
C     1.1939145    4.3117903   -1.4411958
O     1.4534377    5.4315807   -1.6517531
O     3.5663854    1.8259195   -1.8522930
O     1.5603437    2.8875984    1.7671783
C    -1.9187337    1.9701171   -0.0555799
C    -1.8776272    4.1124029   -1.0050859
C    -3.2343521    4.3236173   -0.7320304
C    -3.9555682    3.3032904   -0.0892464
C    -3.2903241    2.1206326    0.2481533
C    -1.1141102    0.7822773    0.2480661
C     0.2167596    0.8397673   -0.1695494
C     1.1073012   -0.2669546    0.1537331
N     0.5226619   -1.3819293    0.8429219
C    -0.8113962   -1.4330731    1.2170725
C    -1.6355385   -0.3635921    0.9287325
H    -1.2791530    4.8905777   -1.5034612
H    -3.7078776    5.2752248   -1.0190629
H    -5.0252499    3.4288905    0.1451857
H    -3.8303504    1.3024026    0.7462112
O     2.3204438   -0.3020275   -0.1188913
C    -1.3260705   -2.6620627    1.9203533
H    -2.6889701   -0.4207582    1.2375034
N    -1.2260492    2.9768015   -0.6777181
H     0.4305322    3.6470920   -3.8642917
C     0.3284714    2.8248482   -3.1539352
C     0.1072887    1.5993603   -2.8967459
C    -0.3320158    0.2701243   -3.2666099
H    -1.2831841   -3.5629399    1.2663069
H    -2.3851514   -2.5114458    2.2126235
H    -0.7412688   -2.8920748    2.8379881
C     1.4470592   -2.4902952    1.1537664
C     1.9717429   -2.4950662    2.5821842
H     2.2958274   -2.3481379    0.4523575
H     0.9511891   -3.4540546    0.9211390
C     3.0186002   -2.5396166    5.2130484
C     2.4738845   -3.7060846    4.6516553
C     1.9522292   -3.6802039    3.3463164
C     2.5307111   -1.3301185    3.1518695
C     3.0448033   -1.3531060    4.4581470
H     3.4249358   -2.5541871    6.2379724
H     2.4496076   -4.6430481    5.2327775
H     1.5253732   -4.6016461    2.9121475
H     2.5769806   -0.4018958    2.5601036
H     3.4779642   -0.4344876    4.8877950
C    -1.1888456   -2.2916190   -4.1159708
C    -2.0009716   -1.1662434   -4.3406083
C    -1.5782448    0.1047369   -3.9237175
C     0.4736494   -0.8723298   -3.0325241
C     0.0486945   -2.1367172   -3.4651808
```

```
H    -1.5195209   -3.2892602   -4.4495344
H    -2.9725869   -1.2768177   -4.8508210
H    -2.2099208    0.9891133   -4.1055410
H     1.4407025   -0.7534001   -2.5191253
H     0.6940667   -3.0138514   -3.2906557

$vibrational spectrum
#  mode     symmetry     wave number   IR intensity    selection rules
#                         cm**(-1)        km/mol          IR     RAMAN
     1                        0.00         0.00000         -       -
     2                        0.00         0.00000         -       -
     3                        0.00         0.00000         -       -
     4                        0.00         0.00000         -       -
     5                        0.00         0.00000         -       -
     6                        0.00         0.00000         -       -
     7         a             13.98         0.16013        YES     YES
     8         a             18.45         0.16169        YES     YES
     9         a             23.39         0.91198        YES     YES
    10         a             26.84         0.00583        YES     YES
    11         a             34.42         0.15750        YES     YES
    12         a             49.10         0.02982        YES     YES
    13         a             52.96         1.01254        YES     YES
    14         a             63.51         0.73153        YES     YES
    15         a             73.89         0.63088        YES     YES
    16         a             79.38         0.96375        YES     YES
    17         a             82.15         1.07061        YES     YES
    18         a             95.65         0.70579        YES     YES
    19         a            100.84         0.36578        YES     YES
    20         a            104.48         0.08703        YES     YES
    21         a            109.67         0.84019        YES     YES
    22         a            122.89         1.57095        YES     YES
    23         a            128.37         0.61223        YES     YES
    24         a            153.86         0.54673        YES     YES
    25         a            158.56         0.16737        YES     YES
    26         a            178.88         0.53684        YES     YES
    27         a            185.41         2.14388        YES     YES
    28         a            192.23         1.63138        YES     YES
    29         a            206.72         1.76481        YES     YES
    30         a            213.42         0.34350        YES     YES
    31         a            243.95         1.05407        YES     YES
    32         a            247.83         1.08144        YES     YES
    33         a            262.98         0.36073        YES     YES
    34         a            281.31         1.23538        YES     YES
    35         a            310.09         5.88444        YES     YES
    36         a            330.66         9.26868        YES     YES
    37         a            342.45         5.75582        YES     YES
    38         a            367.77         4.50765        YES     YES
    39         a            391.01         1.25030        YES     YES
    40         a            402.56         0.10780        YES     YES
    41         a            403.68         2.37536        YES     YES
    42         a            409.01        13.17384        YES     YES
    43         a            431.49        11.95146        YES     YES
    44         a            442.41        14.97434        YES     YES
    45         a            457.92         0.40690        YES     YES
    46         a            464.95         1.20181        YES     YES
    47         a            468.56         6.69897        YES     YES
    48         a            471.25         3.35441        YES     YES
    49         a            478.00         3.89641        YES     YES
    50         a            485.52         6.40456        YES     YES
```

2da

SCF Energy (au) BP86/SV(P)	-2409.004534403	
SCF Energy (au) PBE0/def2-TZVPP	-2408.542817634	
SCF Energy (au) PBE0/def2-TZVPP	-2408.5510777761	(Toluene Correction)
Zero Point Energy (au)	0.3178444	
Chemical Potential (kJ mol^{-1})	679.64	
Dispersion Correction (au) PBE0/def2-TZVPP	-0.06709809	

xyz coordinates
45

Mn	1.5197310	0.5470025	0.3436074
C	1.9098030	0.8269867	2.0810763
C	3.1834914	-0.0444268	0.0203656
C	1.9301135	2.2698775	-0.0671300
O	2.2237609	3.3814057	-0.2662464
O	4.2544275	-0.4565929	-0.1836553
O	2.1616682	1.0008020	3.2032102
C	-1.2049045	-0.1258313	1.2768286
C	-1.1589754	2.0562942	0.4177521
C	-2.5219656	2.2484819	0.6575306
C	-3.2447226	1.1885736	1.2332793
C	-2.5904925	-0.0046829	1.5445731
N	-0.4345005	-1.2439296	1.5412602
C	0.9499691	-1.2028048	1.1921142
C	1.5082306	-2.4082537	1.5598700
C	0.4804587	-3.2427178	2.1402405
C	-0.7448065	-2.4960049	2.1266952
H	-0.5477021	2.8540095	-0.0310344
H	-2.9972974	3.2064907	0.3991233
H	-4.3222679	1.2891001	1.4423592
H	-3.1456491	-0.8365420	1.9889839
H	2.5624864	-2.6854686	1.4310032
C	0.4951000	-4.5502559	2.6685547
N	-0.5072941	0.9101560	0.7131965
C	0.8271748	1.5978722	-2.8620085
C	1.9509091	2.3147130	-3.3463859
C	1.7939842	3.2932883	-4.3372283
C	0.5191695	3.5832855	-4.8561966
C	-0.6026395	2.8814165	-4.3811110
C	-0.4550859	1.8969480	-3.3931721
H	2.9505721	2.0905373	-2.9429216
H	2.6781598	3.8380295	-4.7070955
H	0.4001929	4.3572543	-5.6321738
H	-1.6046992	3.1003949	-4.7859256
H	-1.3312893	1.3404153	-3.0245511
C	0.9579810	0.5504308	-1.8770337
C	0.8890639	-0.5923575	-1.3453656
H	0.7411585	-1.6707348	-1.3980957
H	1.4336625	-5.1283198	2.6819725
C	-0.6914407	-5.1000768	3.1740729
H	-0.6902216	-6.1214082	3.5892289
C	-1.8900312	-4.3559507	3.1584690
H	-2.8156297	-4.7983334	3.5616334
C	-1.9319458	-3.0495780	2.6376779
H	-2.8877072	-2.5094957	2.6526528

$vibrational spectrum

# mode	symmetry	wave number	IR intensity	selection rules	
#		cm**(-1)	km/mol	IR	RAMAN
1		0.00	0.00000	-	-
2		0.00	0.00000	-	-
3		0.00	0.00000	-	-

4		0.00	0.00000	-	-
5		0.00	0.00000	-	-
6		0.00	0.00000	-	-
7	a	16.31	0.07965	YES	YES
8	a	17.65	0.09997	YES	YES
9	a	25.65	0.26847	YES	YES
10	a	47.19	0.24354	YES	YES
11	a	56.47	0.07018	YES	YES
12	a	64.10	0.09528	YES	YES
13	a	67.11	0.32495	YES	YES
14	a	79.62	0.42679	YES	YES
15	a	87.94	0.74734	YES	YES
16	a	99.82	0.51131	YES	YES
17	a	104.85	1.07356	YES	YES
18	a	111.88	0.11920	YES	YES
19	a	122.33	0.21282	YES	YES
20	a	143.47	1.40069	YES	YES
21	a	157.32	5.11267	YES	YES
22	a	168.97	2.48278	YES	YES
23	a	178.12	0.99525	YES	YES
24	a	215.31	0.22371	YES	YES
25	a	238.99	0.23718	YES	YES
26	a	256.73	6.23442	YES	YES
27	a	280.88	0.52625	YES	YES
28	a	306.39	21.32459	YES	YES
29	a	315.88	1.87150	YES	YES
30	a	352.24	1.47949	YES	YES
31	a	366.05	5.07142	YES	YES
32	a	400.09	0.38943	YES	YES
33	a	407.71	7.20090	YES	YES
34	a	422.47	1.06470	YES	YES
35	a	431.12	1.93796	YES	YES
36	a	456.79	15.18417	YES	YES
37	a	473.69	2.51350	YES	YES
38	a	478.93	13.77269	YES	YES
39	a	486.33	3.55622	YES	YES
40	a	493.78	13.48945	YES	YES
41	a	501.81	16.05292	YES	YES
42	a	513.21	1.64317	YES	YES
43	a	516.95	0.49905	YES	YES
44	a	523.19	37.80879	YES	YES
45	a	537.83	7.06320	YES	YES
46	a	554.10	7.08855	YES	YES
47	a	571.12	0.17654	YES	YES
48	a	589.91	8.62866	YES	YES
49	a	611.07	12.74284	YES	YES
50	a	613.67	2.03699	YES	YES

2da′

```
SCF Energy (au) BP86/SV(P)                    -2409.003236277
SCF Energy (au) PBE0/def2-TZVPP               -2408.541068212
SCF Energy (au) PBE0/def2-TZVPP               -2408.5491894788 (Toluene Correction)
Zero Point Energy (au)                        0.3178436
Chemical Potential (kJ mol-1)                 680.78
Dispersion Correction (au) PBE0/def2-TZVPP -0.06958346

xyz coordinates
45

Mn    1.6114787    2.0224123   -0.1294482
C     1.9124242    2.3882443    1.6130026
C     3.3069043    1.4797935   -0.3640030
C     1.9537395    3.7493582   -0.5687954
O     2.1911893    4.8621185   -0.8290349
O     4.3945181    1.0868426   -0.5076838
O     2.1133826    2.6348062    2.7313371
C    -1.0999543    1.1950939    0.7151392
C    -1.1734098    3.3233061   -0.2672068
C    -2.5598082    3.4139375   -0.1227346
C    -3.2331398    2.3243121    0.4589504
C    -2.5076960    1.2075558    0.8767864
N    -0.2598291    0.1667125    1.0975266
C     1.1285164    0.2877215    0.7892940
C     1.7639615   -0.8264179    1.2966744
C     0.7824824   -1.6835292    1.9209497
C    -0.4947175   -1.0432808    1.7940818
H    -0.5986462    4.1430608   -0.7250315
H    -3.0927997    4.3144785   -0.4625970
H    -4.3280388    2.3403880    0.5843860
H    -3.0244406    0.3456729    1.3111972
H     2.8428089   -1.0225844    1.2437251
C     0.8787449   -2.9251389    2.5820625
N    -0.4526673    2.2542483    0.1355482
H     1.4252327    2.9305479   -2.9825719
C     1.2749429    2.1317796   -2.2549764
C     1.0302276    0.9214792   -1.9744502
C     0.6010528   -0.4326949   -2.2310418
H     1.8581874   -3.4207374    2.6835846
C    -0.2794590   -3.5132887    3.1097021
H    -0.2150666   -4.4835600    3.6292674
C    -1.5294780   -2.8709534    2.9860483
H    -2.4316533   -3.3406685    3.4109535
C    -1.6528098   -1.6320255    2.3307129
H    -2.6452131   -1.1660218    2.2707994
C    -0.2569970   -3.0608890   -2.8308959
C    -1.1188276   -1.9713558   -3.0487209
C    -0.6974520   -0.6670022   -2.7534160
C     1.4590036   -1.5386594   -2.0074787
C     1.0315616   -2.8379490   -2.3143524
H    -0.5904122   -4.0856771   -3.0632895
H    -2.1299911   -2.1378258   -3.4561038
H    -1.3683259    0.1890256   -2.9296280
H     2.4680464   -1.3648277   -1.6056212
H     1.7124272   -3.6878085   -2.1426469

$vibrational spectrum
#  mode     symmetry     wave number   IR intensity    selection rules
#                         cm**(-1)        km/mol         IR     RAMAN
     1                        0.00         0.00000        -       -
     2                        0.00         0.00000        -       -
     3                        0.00         0.00000        -       -
```

4		0.00	0.00000	-	-
5		0.00	0.00000	-	-
6		0.00	0.00000	-	-
7	a	15.45	0.16430	YES	YES
8	a	20.66	0.16312	YES	YES
9	a	27.61	0.30383	YES	YES
10	a	49.13	0.08630	YES	YES
11	a	59.94	0.16090	YES	YES
12	a	65.57	0.28800	YES	YES
13	a	68.58	0.53915	YES	YES
14	a	75.86	0.08201	YES	YES
15	a	88.40	0.25179	YES	YES
16	a	98.66	0.56138	YES	YES
17	a	103.05	0.65599	YES	YES
18	a	106.88	0.54148	YES	YES
19	a	126.59	0.02338	YES	YES
20	a	150.94	0.53832	YES	YES
21	a	162.96	2.42157	YES	YES
22	a	168.46	1.32486	YES	YES
23	a	178.57	1.51189	YES	YES
24	a	212.26	0.21873	YES	YES
25	a	238.54	0.76712	YES	YES
26	a	257.32	1.47958	YES	YES
27	a	283.38	3.04667	YES	YES
28	a	310.87	5.24429	YES	YES
29	a	327.36	9.96005	YES	YES
30	a	352.85	0.86261	YES	YES
31	a	366.38	4.12868	YES	YES
32	a	398.02	4.32884	YES	YES
33	a	402.62	7.57207	YES	YES
34	a	421.35	4.23673	YES	YES
35	a	430.23	9.78177	YES	YES
36	a	461.07	5.52510	YES	YES
37	a	475.56	2.62213	YES	YES
38	a	479.33	5.02750	YES	YES
39	a	488.60	0.62185	YES	YES
40	a	494.56	6.21874	YES	YES
41	a	497.45	14.72589	YES	YES
42	a	511.89	1.50073	YES	YES
43	a	519.62	1.34959	YES	YES
44	a	529.49	16.84794	YES	YES
45	a	534.71	3.87538	YES	YES
46	a	567.27	4.37452	YES	YES
47	a	571.42	0.03474	YES	YES
48	a	592.92	6.68595	YES	YES
49	a	611.93	9.00493	YES	YES
50	a	613.42	6.37682	YES	YES

2ea

SCF Energy (au) BP86/SV(P) -2583.758694363
SCF Energy (au) PBE0/def2-TZVPP -2583.308834131
SCF Energy (au) PBE0/def2-TZVPP -2583.3186219410 (Toluene Correction)
Zero Point Energy (au) 0.3858375
Chemical Potential (kJ mol^{-1}) 840.51
Dispersion Correction (au) PBE0/def2-TZVPP -0.07229098

xyz coordinates
53

Mn	2.2230988	0.6317792	-0.0380673
C	2.6156062	1.1476731	1.6392340
C	3.9387085	0.1811112	-0.3247176
C	2.4125292	2.3193182	-0.6911169
O	2.5413774	3.4196005	-1.0551924
O	5.0506470	-0.1331539	-0.4981009
O	2.8767051	1.4700797	2.7277647
C	-0.4539945	-0.0297274	0.9096872
C	-1.9246317	0.0913870	1.0871830
C	0.3558878	-1.1297064	1.4172808
C	1.7432603	-1.0509487	1.0754004
C	2.6077351	-2.0542027	1.5517480
C	2.1177715	-3.1166002	2.3474416
C	0.7427944	-3.1752783	2.6929163
C	-0.1235398	-2.1864952	2.2356941
H	3.6801806	-2.0058785	1.3091043
O	2.8869000	-4.1224309	2.8426613
H	0.3950079	-4.0008383	3.3326722
H	-1.1848113	-2.2197783	2.5316610
N	0.2295362	0.8918881	0.2643551
C	1.2662731	1.0751896	-3.2954025
C	2.2345091	1.8668127	-3.9638754
C	1.8687155	2.6586890	-5.0606656
C	0.5347223	2.6874656	-5.5058725
C	-0.4352712	1.9114622	-4.8473054
C	-0.0776139	1.1120552	-3.7519775
H	3.2792071	1.8489973	-3.6164200
H	2.6348500	3.2643171	-5.5724379
H	0.2515102	3.3164568	-6.3659739
H	-1.4830307	1.9265356	-5.1911149
H	-0.8346306	0.4992213	-3.2369763
C	1.6163849	0.2176182	-2.1884952
C	1.7524455	-0.8361841	-1.5029239
H	1.7703661	-1.9228945	-1.4187534
C	-4.7416295	0.3982927	1.3390996
C	-4.1874919	-0.8306805	0.9085777
C	-2.8044347	-0.9812409	0.7916161
C	-2.4915732	1.3143236	1.5082184
C	-3.8796569	1.4757364	1.6412510
O	-6.0976318	0.4422766	1.4283706
H	-4.8744120	-1.6562320	0.6648427
H	-2.3950795	-1.9390571	0.4327292
H	-1.8296331	2.1579555	1.7662896
H	-4.2759343	2.4409076	1.9886844
H	-0.3377541	1.6556536	-0.1300338
C	-6.7187729	1.6476577	1.8463775
H	-6.4076413	1.9356514	2.8783423
H	-7.8093098	1.4493254	1.8391284
H	-6.4964900	2.4895023	1.1487841
C	4.2807820	-4.1239894	2.5719257
H	4.7805171	-3.2159617	2.9829648
H	4.4885289	-4.1896897	1.4784253

```
H     4.6884108   -5.0239717    3.0749915

$vibrational spectrum
#  mode     symmetry     wave number   IR intensity    selection rules
#                         cm**(-1)        km/mol         IR     RAMAN
     1                      0.00         0.00000         -        -
     2                      0.00         0.00000         -        -
     3                      0.00         0.00000         -        -
     4                      0.00         0.00000         -        -
     5                      0.00         0.00000         -        -
     6                      0.00         0.00000         -        -
     7        a            12.66         0.11198        YES      YES
     8        a            23.85         0.14135        YES      YES
     9        a            28.24         0.19019        YES      YES
    10        a            35.42         0.79697        YES      YES
    11        a            38.32         0.42459        YES      YES
    12        a            47.51         0.69116        YES      YES
    13        a            61.68         0.10633        YES      YES
    14        a            65.84         0.17130        YES      YES
    15        a            75.47         0.44825        YES      YES
    16        a            85.53         1.07252        YES      YES
    17        a            87.59         0.33363        YES      YES
    18        a            94.62         0.20706        YES      YES
    19        a           101.89         0.26170        YES      YES
    20        a           103.56         2.42543        YES      YES
    21        a           114.60         0.52142        YES      YES
    22        a           121.22         1.35366        YES      YES
    23        a           146.22         0.28143        YES      YES
    24        a           154.04         1.68102        YES      YES
    25        a           160.20         0.18190        YES      YES
    26        a           168.67         8.51326        YES      YES
    27        a           180.21         2.01157        YES      YES
    28        a           190.96         1.43101        YES      YES
    29        a           204.10         0.46082        YES      YES
    30        a           236.74         0.90063        YES      YES
    31        a           246.28         0.61014        YES      YES
    32        a           256.47         2.28427        YES      YES
    33        a           266.71         1.10966        YES      YES
    34        a           300.24         1.39938        YES      YES
    35        a           316.99        24.15601        YES      YES
    36        a           319.55         2.82845        YES      YES
    37        a           342.78         0.96020        YES      YES
    38        a           361.53         5.33052        YES      YES
    39        a           400.11         0.10893        YES      YES
    40        a           409.14         5.85992        YES      YES
    41        a           415.96         2.75722        YES      YES
    42        a           422.26         1.54838        YES      YES
    43        a           444.19        13.52271        YES      YES
    44        a           454.37         1.42207        YES      YES
    45        a           458.56         2.83272        YES      YES
    46        a           475.83         3.34516        YES      YES
    47        a           478.06         8.31028        YES      YES
    48        a           488.23         4.80640        YES      YES
    49        a           495.22        10.53210        YES      YES
    50        a           502.27        14.32391        YES      YES
```

2ea′

```
SCF Energy (au) BP86/SV(P)                   -2583.755936542
SCF Energy (au) PBE0/def2-TZVPP              -2583.306604497
SCF Energy (au) PBE0/def2-TZVPP              -2583.3161494270 (Toluene Correction)
Zero Point Energy (au)                       0.3858163
Chemical Potential (kJ mol-1)                841.85
Dispersion Correction (au) PBE0/def2-TZVPP  -0.07594655

xyz coordinates
53

Mn    1.9919400    1.9445815   -0.2125446
C     2.1534808    2.3744254    1.5290994
C     3.7492214    1.5853193   -0.3113953
C     2.1488295    3.6785107   -0.7263564
O     2.2404524    4.7993586   -1.0377011
O     4.8853661    1.3177075   -0.3697285
O     2.2698990    2.6577750    2.6525923
C    -0.7159006    1.1112818    0.4928948
C     0.1043036    0.0054276    0.9687169
C     1.5091645    0.1721588    0.7533872
C     2.3773587   -0.8410027    1.1958039
C     1.8728113   -2.0152812    1.8030053
C     0.4752568   -2.1871422    1.9748359
C    -0.3961908   -1.1855143    1.5574072
H     3.4624674   -0.7284284    1.0533317
O     2.6454017   -3.0414031    2.2472929
H     0.1110908   -3.1192603    2.4333817
H    -1.4825776   -1.3313196    1.6747097
N    -0.0377682    2.0698256   -0.1025837
H     1.6963008    2.6226932   -3.1216314
C     1.7555164    1.8524320   -2.3511876
C     1.7682332    0.6340095   -2.0105774
C     1.8077286   -0.8002035   -2.1790042
C     1.9200425   -3.6011320   -2.5779877
C     3.0989686   -2.8350822   -2.6115416
C     3.0485007   -1.4479013   -2.4103047
C     0.6263831   -1.5815302   -2.1436240
C     0.6864749   -2.9683920   -2.3432424
H     1.9626934   -4.6916637   -2.7344687
H     4.0704284   -3.3222218   -2.7994052
H     3.9712861   -0.8468055   -2.4342545
H    -0.3402928   -1.0879230   -1.9607610
H    -0.2421060   -3.5621325   -2.3141260
C    -2.1920915    1.1993839    0.6436670
C    -5.0083214    1.4914085    0.9169598
C    -4.2119099    1.1238512    2.0274378
C    -2.8306057    0.9750933    1.8900714
C    -2.9975772    1.5734107   -0.4539791
C    -4.3889449    1.7150685   -0.3325568
O    -6.3429842    1.6032685    1.1508950
H    -4.7089911    0.9678227    2.9977093
H    -2.2261438    0.7135808    2.7732747
H    -2.5283966    1.7355727   -1.4391278
H    -4.9787167    1.9923799   -1.2184360
H    -0.6034216    2.8775078   -0.3994740
C    -7.1972825    1.9790936    0.0823794
H    -7.1680455    1.2368679   -0.7501530
H    -6.9375345    2.9876305   -0.3178743
H    -8.2215719    2.0095391    0.5047845
C     4.0556463   -2.9552201    2.1058369
H     4.4730160   -2.0857501    2.6654880
H     4.3560879   -2.8782928    1.0352177
```

```
H     4.4630239   -3.8933836    2.5338468

$vibrational spectrum
#  mode     symmetry     wave number   IR intensity    selection rules
#                         cm**(-1)        km/mol         IR     RAMAN
     1                        0.00         0.00000        -       -
     2                        0.00         0.00000        -       -
     3                        0.00         0.00000        -       -
     4                        0.00         0.00000        -       -
     5                        0.00         0.00000        -       -
     6                        0.00         0.00000        -       -
     7         a             14.75         0.06828       YES     YES
     8         a             28.47         0.19583       YES     YES
     9         a             33.94         0.28112       YES     YES
    10         a             36.54         0.25294       YES     YES
    11         a             38.48         0.16597       YES     YES
    12         a             49.80         1.04111       YES     YES
    13         a             54.04         0.26221       YES     YES
    14         a             64.84         0.11492       YES     YES
    15         a             76.74         0.35850       YES     YES
    16         a             84.30         1.11652       YES     YES
    17         a             89.15         0.20915       YES     YES
    18         a             93.26         0.14079       YES     YES
    19         a             96.89         1.50910       YES     YES
    20         a            107.51         0.21592       YES     YES
    21         a            113.30         0.33480       YES     YES
    22         a            120.74         2.23271       YES     YES
    23         a            149.98         1.82201       YES     YES
    24         a            158.02         1.52750       YES     YES
    25         a            163.84         1.18213       YES     YES
    26         a            175.37         3.19118       YES     YES
    27         a            176.20         2.30265       YES     YES
    28         a            183.71         0.31709       YES     YES
    29         a            192.21         0.53953       YES     YES
    30         a            242.22         0.72845       YES     YES
    31         a            249.25         0.73961       YES     YES
    32         a            255.05         0.48763       YES     YES
    33         a            268.62         1.23736       YES     YES
    34         a            296.84         2.72505       YES     YES
    35         a            305.94        17.11122       YES     YES
    36         a            317.81         1.02634       YES     YES
    37         a            340.78         0.34148       YES     YES
    38         a            362.30         4.98498       YES     YES
    39         a            398.05        10.51671       YES     YES
    40         a            399.04         1.53104       YES     YES
    41         a            415.91         3.33119       YES     YES
    42         a            420.81         0.98381       YES     YES
    43         a            444.89         0.66886       YES     YES
    44         a            454.90         1.69616       YES     YES
    45         a            461.02         2.68253       YES     YES
    46         a            479.34         5.64347       YES     YES
    47         a            484.40        10.14586       YES     YES
    48         a            486.59         7.70917       YES     YES
    49         a            495.03         1.45340       YES     YES
    50         a            507.34         4.79824       YES     YES
```

2fa

SCF Energy (au) BP86/SV(P)	-2391.961394095
SCF Energy (au) PBE0/def2-TZVPP	-2391.518095263
SCF Energy (au) PBE0/def2-TZVPP	-2391.5259797091 (Toluene Correction)
Zero Point Energy (au)	0.3211004
Chemical Potential (kJ mol^{-1})	689.84
Dispersion Correction (au) PBE0/def2-TZVPP	-0.06699866

xyz coordinates
45

Mn	1.4798866	-0.2187247	0.6042757
C	1.9858282	0.1850369	2.2864095
C	3.1078742	-0.8153644	0.1948714
C	1.9261647	1.4494384	0.0171510
O	2.3355042	2.4877551	-0.3203568
O	4.1697173	-1.2232451	-0.0717724
O	2.3152063	0.4401459	3.3737385
C	-1.1801954	-0.8102208	1.8566652
C	-1.2646296	1.3042492	0.8362574
C	-2.6149049	1.4850452	1.2047053
C	-3.2443526	0.4625696	1.9267944
C	-2.5298906	-0.6901692	2.2573493
C	-0.3367492	-1.9750680	2.1535723
C	1.0015422	-1.9308193	1.6664095
C	1.8409481	-3.0288489	1.9547913
C	1.3735263	-4.1396970	2.6779099
C	0.0448376	-4.1782901	3.1430338
C	-0.8044324	-3.0978098	2.8820827
O	-0.5611747	2.2223023	0.1479445
H	-3.1579348	2.4008478	0.9352112
H	-4.2982282	0.5728714	2.2309414
H	-3.0099922	-1.4987815	2.8248023
H	2.8878098	-3.0300007	1.6082221
H	2.0544672	-4.9834827	2.8831749
H	-0.3244333	-5.0473483	3.7115733
H	-1.8414812	-3.1328419	3.2538322
N	-0.5632583	0.1880328	1.1483020
C	0.5092592	0.5040600	-2.6026110
C	1.5628757	1.2155775	-3.2293841
C	1.3059008	2.0406282	-4.3338314
C	-0.0025131	2.1825144	-4.8298025
C	-1.0570294	1.4841539	-4.2145901
C	-0.8076042	0.6527288	-3.1127119
H	2.5900246	1.1076915	-2.8487855
H	2.1398662	2.5794532	-4.8133716
H	-0.1994433	2.8342939	-5.6969663
H	-2.0855771	1.5825957	-4.6007656
H	-1.6311973	0.0979856	-2.6352136
C	0.7397492	-0.3979229	-1.4957523
C	0.6903825	-1.4982035	-0.8680577
H	0.5017714	-2.5721248	-0.8433323
C	-1.1618881	3.4526384	-0.2331367
H	-0.3662870	4.0176456	-0.7566458
H	-1.5042302	4.0308366	0.6560919
H	-2.0157150	3.2878659	-0.9290253

$vibrational spectrum

# mode	symmetry	wave number	IR intensity	selection rules	
#		cm**(-1)	km/mol	IR	RAMAN
1		0.00	0.00000	-	-
2		0.00	0.00000	-	-
3		0.00	0.00000	-	-

4		0.00	0.00000	-	-
5		0.00	0.00000	-	-
6		0.00	0.00000	-	-
7	a	15.69	0.21459	YES	YES
8	a	29.61	0.05641	YES	YES
9	a	34.01	0.12405	YES	YES
10	a	43.43	0.08971	YES	YES
11	a	63.13	1.35943	YES	YES
12	a	75.59	1.13964	YES	YES
13	a	80.60	0.41255	YES	YES
14	a	90.18	0.49284	YES	YES
15	a	93.53	0.02787	YES	YES
16	a	100.58	0.83466	YES	YES
17	a	114.14	0.30028	YES	YES
18	a	118.32	0.18218	YES	YES
19	a	122.21	0.39617	YES	YES
20	a	129.25	0.90286	YES	YES
21	a	154.99	1.38789	YES	YES
22	a	156.86	0.72739	YES	YES
23	a	167.85	3.83857	YES	YES
24	a	182.29	1.19717	YES	YES
25	a	218.81	0.80044	YES	YES
26	a	229.75	0.95873	YES	YES
27	a	242.21	3.23163	YES	YES
28	a	254.84	0.06903	YES	YES
29	a	288.66	0.56483	YES	YES
30	a	305.04	0.17203	YES	YES
31	a	324.49	21.26696	YES	YES
32	a	334.75	2.71008	YES	YES
33	a	402.42	0.01934	YES	YES
34	a	407.91	0.84390	YES	YES
35	a	415.07	4.41464	YES	YES
36	a	435.04	8.05753	YES	YES
37	a	454.51	15.75093	YES	YES
38	a	459.11	2.46109	YES	YES
39	a	471.16	4.40160	YES	YES
40	a	478.30	3.94107	YES	YES
41	a	490.99	6.31778	YES	YES
42	a	499.39	3.20063	YES	YES
43	a	508.02	18.13741	YES	YES
44	a	513.95	2.54387	YES	YES
45	a	529.00	31.57498	YES	YES
46	a	536.56	11.31513	YES	YES
47	a	541.47	12.12873	YES	YES
48	a	555.01	2.26347	YES	YES
49	a	581.31	2.25487	YES	YES
50	a	606.94	24.76174	YES	YES

2ej

SCF Energy (au) BP86/SV(P)	-2814.654527730
SCF Energy (au) PBE0/def2-TZVPP	-2814.168815490
SCF Energy (au) PBE0/def2-TZVPP	-2814.1792225655 (Toluene Correction)
Zero Point Energy (au)	0.4650797
Chemical Potential (kJ mol^{-1})	1029.53
Dispersion Correction (au) PBE0/def2-TZVPP	-0.09205668

xyz coordinates
63

Mn	1.9456160	1.1548608	0.1958505
C	2.3174964	1.7535376	1.8490724
C	3.6459403	0.6209492	-0.0252364
C	2.2417920	2.8057008	-0.5079093
O	2.4490777	3.8871159	-0.8928812
O	4.7469566	0.2515105	-0.1584022
O	2.5659906	2.1407198	2.9191820
C	-0.7687233	0.6675232	1.1429281
C	-2.2330554	0.8694433	1.2963279
C	-0.0099007	-0.4218190	1.7424040
C	1.3817626	-0.4320683	1.4019474
C	2.1968402	-1.4323580	1.9608592
C	1.6568351	-2.4100915	2.8285623
C	0.2808277	-2.3773202	3.1737144
C	-0.5390031	-1.3877409	2.6386067
H	3.2693013	-1.4571753	1.7161129
O	2.3758798	-3.4141733	3.3952372
H	-0.1049106	-3.1348798	3.8732171
H	-1.6006016	-1.3485107	2.9327252
N	-0.0394002	1.5042181	0.4342321
C	0.7799226	1.6009485	-2.9389564
C	1.6257514	2.4310501	-3.7179503
C	1.0916549	3.2341651	-4.7350415
C	-0.2917779	3.2368847	-4.9889727
C	-1.1411542	2.4223127	-4.2198555
C	-0.6147849	1.6105739	-3.2041981
H	2.7091332	2.4347565	-3.5215034
H	1.7642898	3.8688724	-5.3354938
H	-0.7074816	3.8743582	-5.7867409
H	-2.2266108	2.4146907	-4.4148064
H	-1.2773778	0.9640588	-2.6069194
C	1.3055038	0.7353344	-1.9069689
C	1.5375929	-0.3836632	-1.3569005
C	1.7895150	-1.8090780	-1.4301288
C	-5.0344965	1.3238325	1.5025429
C	-4.5355261	0.0372441	1.1888465
C	-3.1605106	-0.1846951	1.0941285
C	-2.7457344	2.1499997	1.6003473
C	-4.1252938	2.3847820	1.7093693
O	-6.3870314	1.4358920	1.5812182
H	-5.2583003	-0.7757992	1.0176185
H	-2.7951668	-1.1888858	0.8259732
H	-2.0473340	2.9833999	1.7849273
H	-4.4783142	3.3945124	1.9645007
H	-0.5664635	2.2572249	-0.0305651
C	-6.9544541	2.7007651	1.8840578
H	-6.6311035	3.0681702	2.8864834
H	-8.0526205	2.5505129	1.8903394
H	-6.6950736	3.4643635	1.1130867
C	3.7623117	-3.5203748	3.1025461
H	4.3241281	-2.6193473	3.4428990
H	3.9408832	-3.6724222	2.0130853

```
H     4.1242530   -4.4071241    3.6610765
C     2.2998198   -4.5825249   -1.6992044
C     1.1606521   -4.1354233   -1.0061845
C     0.9082016   -2.7631267   -0.8644706
C     2.9391798   -2.2701009   -2.1219546
C     3.1886010   -3.6441445   -2.2533474
H     2.4955186   -5.6624250   -1.8064831
H     0.4604443   -4.8642520   -0.5655675
H     0.0205352   -2.4157940   -0.3143619
H     3.6347926   -1.5346342   -2.5551036
H     4.0852046   -3.9843320   -2.7979179

$vibrational spectrum
#  mode     symmetry     wave number   IR intensity    selection rules
#                         cm**(-1)        km/mol         IR     RAMAN
     1                        0.00         0.00000        -       -
     2                        0.00         0.00000        -       -
     3                        0.00         0.00000        -       -
     4                        0.00         0.00000        -       -
     5                        0.00         0.00000        -       -
     6                        0.00         0.00000        -       -
     7        a              11.41         0.09327       YES     YES
     8        a              16.85         0.05804       YES     YES
     9        a              20.99         0.16957       YES     YES
    10        a              28.16         0.10066       YES     YES
    11        a              36.00         0.86956       YES     YES
    12        a              39.01         0.31055       YES     YES
    13        a              45.19         0.10445       YES     YES
    14        a              48.27         0.84812       YES     YES
    15        a              53.24         0.12915       YES     YES
    16        a              61.97         0.17545       YES     YES
    17        a              70.80         0.30147       YES     YES
    18        a              77.93         0.10995       YES     YES
    19        a              83.73         0.90023       YES     YES
    20        a              90.22         0.23146       YES     YES
    21        a              94.80         0.17699       YES     YES
    22        a              98.30         0.47627       YES     YES
    23        a             104.33         0.89218       YES     YES
    24        a             109.49         0.93333       YES     YES
    25        a             118.15         1.82754       YES     YES
    26        a             146.42         1.64226       YES     YES
    27        a             150.95         1.34614       YES     YES
    28        a             158.67         0.88868       YES     YES
    29        a             169.35         2.88200       YES     YES
    30        a             181.04         2.40710       YES     YES
    31        a             188.68         3.26368       YES     YES
    32        a             191.67         1.53467       YES     YES
    33        a             206.86         0.62635       YES     YES
    34        a             230.76         3.27932       YES     YES
    35        a             234.86         2.02230       YES     YES
    36        a             248.55         0.36116       YES     YES
    37        a             253.94         1.23472       YES     YES
    38        a             267.87         0.75825       YES     YES
    39        a             296.61         3.74998       YES     YES
    40        a             320.41         2.34102       YES     YES
    41        a             341.10         0.88454       YES     YES
    42        a             360.93         5.44085       YES     YES
    43        a             376.09        10.14822       YES     YES
    44        a             382.42        32.74494       YES     YES
    45        a             401.20         0.01273       YES     YES
    46        a             402.61         0.11511       YES     YES
    47        a             415.93         2.26611       YES     YES
    48        a             421.73         1.09351       YES     YES
```

49	a	440.76	4.23288	YES	YES
50	a	453.33	1.25458	YES	YES

2fa′

```
SCF Energy (au) BP86/SV(P)                    -2391.959491985
SCF Energy (au) PBE0/def2-TZVPP               -2391.516131723
SCF Energy (au) PBE0/def2-TZVPP               -2391.5242788848 (Toluene Correction)
Zero Point Energy (au)                        0.3212083
Chemical Potential (kJ mol-1)                 690.67
Dispersion Correction (au) PBE0/def2-TZVPP -0.06847143

xyz coordinates
45

Mn    1.3963769    1.3583796    0.3444836
C     1.4957594    1.7909642    2.0933701
C     3.1423484    1.0164151    0.2487564
C     1.6670801    3.0927734   -0.1289336
O     1.9417192    4.1914285   -0.4091898
O     4.2810258    0.7630211    0.1844692
O     1.5645760    2.0826934    3.2182820
C    -1.3013999    0.2766545    1.0687616
C    -1.5652880    2.3506976   -0.0038004
C    -2.9709816    2.2805102    0.0915204
C    -3.5348398    1.1533677    0.7050525
C    -2.7031045    0.1460634    1.1961401
C    -0.3361981   -0.7167661    1.5532949
C     1.0401974   -0.4292863    1.3235092
C     1.9895755   -1.3493861    1.8180369
C     1.5987860   -2.5251865    2.4814980
C     0.2347955   -2.8085505    2.6814675
C    -0.7272833   -1.9041925    2.2201260
O    -0.9183472    3.3876342   -0.5742201
H    -3.6082712    3.0827651   -0.3038207
H    -4.6300732    1.0672355    0.7956806
H    -3.1312896   -0.7437030    1.6772621
H     3.0676964   -1.1558228    1.6898941
H     2.3676865   -3.2262140    2.8491260
H    -0.0759951   -3.7294558    3.2012154
H    -1.7934148   -2.1283994    2.3874980
N    -0.7487716    1.3797544    0.4702178
H     1.2478329    2.1441806   -2.5348533
C     1.1781634    1.3641921   -1.7754152
C     1.0614090    0.1419872   -1.4550057
C     0.8178416   -1.2600164   -1.7094089
C     0.3304515   -3.9864540   -2.2994260
C     1.5879058   -3.5794731   -1.8181482
C     1.8312532   -2.2322700   -1.5175448
C    -0.4470777   -1.6804654   -2.1938190
C    -0.6847403   -3.0319935   -2.4845658
H     0.1423019   -5.0482405   -2.5298388
H     2.3899640   -4.3218948   -1.6721158
H     2.8161788   -1.9143457   -1.1427412
H    -1.2399391   -0.9305886   -2.3445678
H    -1.6733543   -3.3420701   -2.8625993
C    -1.6486398    4.5142629   -1.0398888
H    -2.3272451    4.2424261   -1.8811164
H    -0.8856423    5.2328984   -1.3984561
H    -2.2390287    4.9844696   -0.2201868

$vibrational spectrum
#  mode     symmetry     wave number   IR intensity    selection rules
#                         cm**(-1)        km/mol          IR     RAMAN
     1                        0.00         0.00000        -        -
     2                        0.00         0.00000        -        -
     3                        0.00         0.00000        -        -
```

4		0.00	0.00000	-	-
5		0.00	0.00000	-	-
6		0.00	0.00000	-	-
7	a	17.56	0.32176	YES	YES
8	a	30.56	0.34957	YES	YES
9	a	35.61	0.34595	YES	YES
10	a	46.86	0.06387	YES	YES
11	a	53.70	0.06745	YES	YES
12	a	73.53	1.80693	YES	YES
13	a	80.07	0.76770	YES	YES
14	a	92.33	0.60385	YES	YES
15	a	96.13	0.24797	YES	YES
16	a	101.05	0.13766	YES	YES
17	a	109.72	0.88510	YES	YES
18	a	118.53	0.25899	YES	YES
19	a	120.29	0.21308	YES	YES
20	a	126.82	0.65942	YES	YES
21	a	156.16	0.25574	YES	YES
22	a	164.73	0.43699	YES	YES
23	a	176.58	2.86296	YES	YES
24	a	184.23	1.52408	YES	YES
25	a	219.10	0.21957	YES	YES
26	a	232.13	1.09117	YES	YES
27	a	233.24	1.55831	YES	YES
28	a	263.08	0.18058	YES	YES
29	a	289.97	0.69181	YES	YES
30	a	308.04	0.41727	YES	YES
31	a	325.07	11.81392	YES	YES
32	a	334.42	1.62532	YES	YES
33	a	400.32	0.77081	YES	YES
34	a	407.09	10.83810	YES	YES
35	a	409.88	5.26984	YES	YES
36	a	430.73	0.31742	YES	YES
37	a	458.21	1.77025	YES	YES
38	a	461.90	4.09505	YES	YES
39	a	472.38	4.53511	YES	YES
40	a	482.55	7.02191	YES	YES
41	a	493.96	3.82026	YES	YES
42	a	498.09	3.42824	YES	YES
43	a	506.42	21.10995	YES	YES
44	a	508.51	0.81419	YES	YES
45	a	529.26	8.32951	YES	YES
46	a	537.81	17.08554	YES	YES
47	a	541.32	2.62297	YES	YES
48	a	568.59	3.32784	YES	YES
49	a	581.32	2.91337	YES	YES
50	a	612.10	26.50879	YES	YES

2ga

```
SCF Energy (au) BP86/SV(P)                    -2508.397058357
SCF Energy (au) PBE0/def2-TZVPP               -2507.918314687
SCF Energy (au) PBE0/def2-TZVPP               -2507.9263616631 (Toluene Correction)
Zero Point Energy (au)                        0.3679099
Chemical Potential (kJ mol-1)                 804.72
Dispersion Correction (au) PBE0/def2-TZVPP -0.08238683
```

xyz coordinates
51

Mn	1.4627019	-0.6136569	0.6719518
C	1.9354949	0.0695481	2.2687287
C	3.0921388	-1.2780301	0.4018875
C	1.9643077	0.8904391	-0.2341898
O	2.4492548	1.7919919	-0.7928015
O	4.1559398	-1.7320077	0.2404221
O	2.2409436	0.4997903	3.3074133
C	-1.2743036	-1.2048065	1.6578451
C	-1.3235574	0.9260137	0.6467378
C	-2.7339246	0.9150606	0.6335650
C	-3.4234327	-0.1952212	1.1390735
C	-2.6868249	-1.2467250	1.6858767
C	-0.4209420	-2.2522340	2.2349893
C	0.9717282	-2.1633040	1.9506955
C	1.8238570	-3.1294950	2.5254005
C	1.3197737	-4.1548839	3.3444704
C	-0.0604725	-4.2411538	3.6088029
C	-0.9265764	-3.2923808	3.0539203
C	-0.6419949	2.2018926	0.2795213
H	-3.2694568	1.8023754	0.2637775
H	-4.5254493	-0.2231023	1.1354352
H	-3.1980078	-2.1159172	2.1239789
H	2.9098132	-3.0934209	2.3361770
H	2.0124835	-4.8924719	3.7849519
H	-0.4575073	-5.0421799	4.2535378
H	-2.0039605	-3.3563542	3.2794642
N	-0.6094863	-0.1505738	1.0806026
C	0.3156615	-0.4401438	-2.5752413
C	1.2673483	0.2390870	-3.3774685
C	0.8691893	0.8875930	-4.5549764
C	-0.4793001	0.8783310	-4.9545738
C	-1.4323542	0.2068282	-4.1678224
C	-1.0430860	-0.4459915	-2.9889960
H	2.3248815	0.2482745	-3.0744248
H	1.6234532	1.4069923	-5.1688521
H	-0.7868842	1.3911799	-5.8809002
H	-2.4908057	0.1869449	-4.4772127
H	-1.7867481	-0.9773241	-2.3740579
C	0.6874200	-1.1659350	-1.3824163
C	0.7767075	-2.1594344	-0.6038190
H	0.7188242	-3.2323749	-0.4197090
C	0.4681783	4.7342046	-0.2978530
C	0.6605090	4.1523752	0.9671899
C	0.1111930	2.8946908	1.2554809
C	-0.8466833	2.8017314	-0.9816079
C	-0.2847516	4.0553750	-1.2705521
H	0.9067758	5.7197436	-0.5261296
H	1.2440403	4.6821833	1.7380140
H	0.2553213	2.4465547	2.2516542
H	-1.4278246	2.2673028	-1.7507523
H	-0.4336055	4.5026188	-2.2672091

```
$vibrational spectrum
#  mode     symmetry     wave number   IR intensity    selection rules
#                         cm**(-1)        km/mol         IR     RAMAN
     1                       0.00         0.00000         -       -
     2                       0.00         0.00000         -       -
     3                       0.00         0.00000         -       -
     4                       0.00         0.00000         -       -
     5                       0.00         0.00000         -       -
     6                       0.00         0.00000         -       -
     7         a            10.36         0.19938        YES     YES
     8         a            27.51         0.04880        YES     YES
     9         a            38.08         0.05465        YES     YES
    10         a            42.39         0.27325        YES     YES
    11         a            57.15         0.17486        YES     YES
    12         a            68.93         0.12536        YES     YES
    13         a            74.12         0.12993        YES     YES
    14         a            75.27         0.85869        YES     YES
    15         a            81.84         0.06469        YES     YES
    16         a            89.05         0.58480        YES     YES
    17         a            93.51         0.42378        YES     YES
    18         a           111.22         0.16990        YES     YES
    19         a           113.13         0.03525        YES     YES
    20         a           117.12         0.61225        YES     YES
    21         a           121.99         0.37536        YES     YES
    22         a           139.20         0.58041        YES     YES
    23         a           155.50         4.72657        YES     YES
    24         a           160.00         0.76063        YES     YES
    25         a           175.36         1.07927        YES     YES
    26         a           210.04         0.69555        YES     YES
    27         a           220.26         0.16892        YES     YES
    28         a           237.95         1.93218        YES     YES
    29         a           242.94         2.19533        YES     YES
    30         a           294.11         2.09996        YES     YES
    31         a           314.01         0.68797        YES     YES
    32         a           321.10        19.21421        YES     YES
    33         a           337.85         1.48906        YES     YES
    34         a           372.54         1.02664        YES     YES
    35         a           400.01         0.24974        YES     YES
    36         a           401.56         0.02427        YES     YES
    37         a           410.61         5.14172        YES     YES
    38         a           431.09         4.52154        YES     YES
    39         a           450.68        15.38779        YES     YES
    40         a           459.24         1.28575        YES     YES
    41         a           465.75         4.47669        YES     YES
    42         a           476.92         5.61536        YES     YES
    43         a           477.59         5.69098        YES     YES
    44         a           489.88         4.79517        YES     YES
    45         a           498.86         8.36890        YES     YES
    46         a           505.49        15.89184        YES     YES
    47         a           512.07         0.41507        YES     YES
    48         a           525.04        22.93634        YES     YES
    49         a           539.12         9.81531        YES     YES
    50         a           545.76         4.36875        YES     YES
```

2ga′

```
SCF Energy (au) BP86/SV(P)                    -2508.396040454
SCF Energy (au) PBE0/def2-TZVPP               -2507.916677625
SCF Energy (au) PBE0/def2-TZVPP               -2507.9248487015 (Toluene Correction)
Zero Point Energy (au)                        0.3677349
Chemical Potential (kJ mol-1)                 803.07
Dispersion Correction (au) PBE0/def2-TZVPP -0.08281505

xyz coordinates
51

Mn    1.4391057    0.6238850    0.2962395
C     1.6057090    1.2800530    1.9682203
C     3.1384581    0.0991255    0.2177723
C     1.9242572    2.2136855   -0.4425164
O     2.3853360    3.1769319   -0.9117753
O     4.2454900   -0.2706716    0.1786023
O     1.7088666    1.7165425    3.0427783
C    -1.3073847   -0.3107272    0.9690128
C    -1.5041005    1.7935720   -0.0768454
C    -2.8934610    1.6153724   -0.2371849
C    -3.4956725    0.4332819    0.2151002
C    -2.6999867   -0.5227177    0.8463202
C    -0.4005191   -1.2565522    1.6317917
C     0.9936664   -1.0095990    1.4871117
C     1.8834806   -1.8698532    2.1640286
C     1.4185878   -2.9515258    2.9324487
C     0.0385806   -3.1996671    3.0476429
C    -0.8663112   -2.3526857    2.3985808
C    -0.9366685    3.1277568   -0.4318752
H    -3.4886153    2.4275855   -0.6811821
H    -4.5799235    0.2729178    0.0967109
H    -3.1447986   -1.4512244    1.2310743
H     2.9721522   -1.7048341    2.1004869
H     2.1417944   -3.6047800    3.4502864
H    -0.3304881   -4.0452930    3.6506908
H    -1.9468188   -2.5412764    2.5099494
N    -0.7183081    0.8205497    0.4609888
H     1.1918898    1.0978682   -2.6496469
C     1.1094174    0.4155304   -1.8019133
C     0.9619543   -0.7563221   -1.3339748
C     0.6429493   -2.1626103   -1.4536003
C     0.0072647   -4.9014407   -1.7918630
C     1.2330252   -4.5245521   -1.2139585
C     1.5483836   -3.1703207   -1.0374688
C    -0.5901544   -2.5528379   -2.0368187
C    -0.9018073   -3.9104278   -2.2019866
H    -0.2391899   -5.9682252   -1.9226733
H     1.9521015   -5.2959201   -0.8921393
H     2.5079090   -2.8774387   -0.5853995
H    -1.2990600   -1.7747098   -2.3615368
H    -1.8639889   -4.1971431   -2.6585568
C    -0.0654151    5.7462825   -1.0362026
C     0.0314850    5.2560744    0.2776686
C    -0.3981314    3.9555611    0.5794579
C    -1.0496012    3.6351764   -1.7444527
C    -0.6052271    4.9326472   -2.0465734
H     0.2807037    6.7657957   -1.2734075
H     0.4487774    5.8915806    1.0758772
H    -0.3250503    3.5756515    1.6111820
H    -1.4787104    2.9993103   -2.5369369
H    -0.6819526    5.3106181   -3.0795345
```

$vibrational spectrum

# mode	symmetry	wave number cm**(-1)	IR intensity km/mol	selection rules IR	RAMAN
1		0.00	0.00000	-	-
2		0.00	0.00000	-	-
3		0.00	0.00000	-	-
4		0.00	0.00000	-	-
5		0.00	0.00000	-	-
6		0.00	0.00000	-	-
7	a	14.68	0.02458	YES	YES
8	a	20.00	0.02170	YES	YES
9	a	26.94	0.27393	YES	YES
10	a	44.89	0.09041	YES	YES
11	a	52.65	0.10546	YES	YES
12	a	55.18	0.14935	YES	YES
13	a	68.98	0.10168	YES	YES
14	a	75.19	0.90378	YES	YES
15	a	80.23	0.43735	YES	YES
16	a	95.98	0.04786	YES	YES
17	a	96.61	0.15507	YES	YES
18	a	105.08	0.15543	YES	YES
19	a	111.34	0.29671	YES	YES
20	a	118.14	0.38801	YES	YES
21	a	124.61	0.95451	YES	YES
22	a	137.24	0.09103	YES	YES
23	a	158.46	0.86799	YES	YES
24	a	174.47	1.38164	YES	YES
25	a	183.09	1.57158	YES	YES
26	a	200.25	0.43881	YES	YES
27	a	216.64	0.84383	YES	YES
28	a	229.46	1.29740	YES	YES
29	a	240.10	0.08552	YES	YES
30	a	297.63	0.15925	YES	YES
31	a	311.68	0.62241	YES	YES
32	a	330.86	11.24064	YES	YES
33	a	337.66	1.74335	YES	YES
34	a	370.52	1.39688	YES	YES
35	a	399.39	0.24768	YES	YES
36	a	399.93	0.79518	YES	YES
37	a	407.82	14.42548	YES	YES
38	a	426.95	0.94010	YES	YES
39	a	453.00	0.03982	YES	YES
40	a	459.14	3.39036	YES	YES
41	a	462.50	8.72856	YES	YES
42	a	476.80	6.22106	YES	YES
43	a	481.75	0.17525	YES	YES
44	a	490.98	5.09356	YES	YES
45	a	499.22	0.51778	YES	YES
46	a	505.01	18.43589	YES	YES
47	a	512.38	4.66827	YES	YES
48	a	531.55	4.58873	YES	YES
49	a	541.02	12.91639	YES	YES
50	a	549.53	3.33764	YES	YES

2ha

```
SCF Energy (au) BP86/SV(P)                      -2354.867702753
SCF Energy (au) PBE0/def2-TZVPP                 -2354.415337120
SCF Energy (au) PBE0/def2-TZVPP                 -2354.4233622997 (Toluene Correction)
Zero Point Energy (au)                          0.3227920
Chemical Potential (kJ mol-1)                   692.09
Dispersion Correction (au) PBE0/def2-TZVPP -0.06448300

xyz coordinates
45

Mn    1.9844632    0.3615964    0.2805713
C     2.4729053    0.7706078    1.9633091
C     3.6802028   -0.0913158   -0.1161913
C     2.1691997    2.0841028   -0.2778616
O     2.2954408    3.2028442   -0.5801308
O     4.7776609   -0.3989431   -0.3667064
O     2.7934382    1.0254737    3.0533302
C    -0.6474250   -0.2993731    1.3472536
C    -2.0987172   -0.1416954    1.6399885
C     0.1625301   -1.4533976    1.7369729
C     1.5284218   -1.3774515    1.3141167
C     2.3774492   -2.4442437    1.6743531
C     1.8981386   -3.5411240    2.4143880
C     0.5545604   -3.5942075    2.8362105
C    -0.3123366   -2.5468645    2.5030714
H     3.4404841   -2.4287957    1.3790219
H     2.5876445   -4.3616677    2.6781061
H     0.1908380   -4.4464731    3.4329670
H    -1.3580410   -2.5659727    2.8511029
N     0.0191421    0.6347120    0.7090967
C     0.9878452    1.0097481   -2.9360200
C     1.9878718    1.7884232   -3.5718213
C     1.6561809    2.6351458   -4.6380820
C     0.3254007    2.7326849   -5.0831244
C    -0.6758427    1.9707337   -4.4556397
C    -0.3522845    1.1163009   -3.3916256
H     3.0304467    1.7157479   -3.2248581
H     2.4468762    3.2286812   -5.1259904
H     0.0691267    3.4043029   -5.9190083
H    -1.7212712    2.0399266   -4.7997030
H    -1.1341259    0.5134927   -2.9024547
C     1.3062087    0.0918749   -1.8676401
C     1.4093925   -1.0031709   -1.2426333
H     1.3803549   -2.0928587   -1.2210400
C    -4.8598289    0.2380123    2.1238249
C    -4.4023850   -0.9573970    1.5420035
C    -3.0322054   -1.1508820    1.3058817
C    -2.5709113    1.0600181    2.2188912
C    -3.9402320    1.2457316    2.4628171
H    -5.9358974    0.3841697    2.3141531
H    -5.1204428   -1.7467476    1.2648043
H    -2.6820451   -2.0814766    0.8311990
H    -1.8499617    1.8443132    2.5038226
H    -4.2907055    2.1823146    2.9268384
H    -0.5475650    1.4430989    0.4124348

$vibrational spectrum
#  mode     symmetry     wave number   IR intensity    selection rules
#                         cm**(-1)        km/mol          IR     RAMAN
     1                        0.00         0.00000         -       -
     2                        0.00         0.00000         -       -
     3                        0.00         0.00000         -       -
```

4		0.00	0.00000	-	-
5		0.00	0.00000	-	-
6		0.00	0.00000	-	-
7	a	13.29	0.15192	YES	YES
8	a	23.03	0.07633	YES	YES
9	a	33.10	0.10247	YES	YES
10	a	43.98	0.05862	YES	YES
11	a	51.74	0.15394	YES	YES
12	a	61.36	0.17907	YES	YES
13	a	71.43	0.28741	YES	YES
14	a	78.72	0.06019	YES	YES
15	a	85.78	0.82130	YES	YES
16	a	91.19	0.05240	YES	YES
17	a	99.69	0.24511	YES	YES
18	a	109.24	0.33295	YES	YES
19	a	115.10	0.66219	YES	YES
20	a	126.70	0.21360	YES	YES
21	a	150.72	0.94188	YES	YES
22	a	160.87	5.74710	YES	YES
23	a	185.99	0.60228	YES	YES
24	a	204.17	1.78023	YES	YES
25	a	214.52	2.04916	YES	YES
26	a	236.75	1.95956	YES	YES
27	a	274.26	2.42220	YES	YES
28	a	308.38	1.57647	YES	YES
29	a	317.62	23.99863	YES	YES
30	a	353.85	0.52186	YES	YES
31	a	399.49	0.02689	YES	YES
32	a	403.76	0.21821	YES	YES
33	a	410.32	4.31237	YES	YES
34	a	421.74	6.72909	YES	YES
35	a	446.90	9.46617	YES	YES
36	a	451.86	1.39235	YES	YES
37	a	464.55	11.74879	YES	YES
38	a	477.59	6.21398	YES	YES
39	a	488.60	4.57895	YES	YES
40	a	490.74	0.53493	YES	YES
41	a	495.96	12.78844	YES	YES
42	a	501.78	3.93526	YES	YES
43	a	503.02	13.71281	YES	YES
44	a	524.14	33.20199	YES	YES
45	a	536.84	10.50917	YES	YES
46	a	546.50	11.89717	YES	YES
47	a	584.32	10.55322	YES	YES
48	a	609.23	6.23246	YES	YES
49	a	611.60	6.04316	YES	YES
50	a	618.08	26.29849	YES	YES

2ha′

SCF Energy (au) BP86/SV(P) -2354.864921936
SCF Energy (au) PBE0/def2-TZVPP -2354.412686755
SCF Energy (au) PBE0/def2-TZVPP -2354.4205956618 (Toluene Correction)
Zero Point Energy (au) 0.3226891
Chemical Potential (kJ mol^{-1}) 689.13
Dispersion Correction (au) PBE0/def2-TZVPP -0.06741741

xyz coordinates
45

Mn	1.7370355	1.9156621	-0.0185669
C	1.9577555	2.3012045	1.7280394
C	3.5025465	1.6204490	-0.1985573
C	1.8152550	3.6694973	-0.4796261
O	1.8576291	4.8024604	-0.7541614
O	4.6427294	1.3986452	-0.3138999
O	2.1139695	2.5589014	2.8526198
C	-0.9128298	0.9532138	0.7371207
C	-0.0320248	-0.1264507	1.1817887
C	1.3579263	0.1145890	0.9381381
C	2.2687341	-0.8681919	1.3733419
C	1.8252121	-2.0586472	1.9791169
C	0.4511831	-2.3013110	2.1698957
C	-0.4790054	-1.3349730	1.7693790
H	3.3531148	-0.7201772	1.2347946
H	2.5643202	-2.8133496	2.2986332
H	0.1092259	-3.2436898	2.6277984
H	-1.5577047	-1.5208744	1.8999189
N	-0.2877921	1.9497253	0.1541413
H	1.4157086	2.6883447	-2.9025956
C	1.4505005	1.8998432	-2.1490172
C	1.4570271	0.6712439	-1.8394499
C	1.3933284	-0.7536490	-2.0724049
C	1.2754370	-3.5277641	-2.6245405
C	2.5071684	-2.9174475	-2.3265894
C	2.5693084	-1.5455561	-2.0451657
C	0.1564402	-1.3787876	-2.3703728
C	0.1022075	-2.7536656	-2.6432421
H	1.2301733	-4.6077036	-2.8416829
H	3.4320346	-3.5175530	-2.3114330
H	3.5339045	-1.0675370	-1.8131480
H	-0.7618670	-0.7708876	-2.3936988
H	-0.8675413	-3.2241721	-2.8760245
C	-2.3914229	0.9752072	0.9151925
C	-5.1970723	1.1358492	1.2411788
C	-4.3749026	0.8234653	2.3378931
C	-2.9827916	0.7378493	2.1781861
C	-3.2278479	1.2961468	-0.1798602
C	-4.6198514	1.3707898	-0.0187747
H	-6.2904248	1.1971927	1.3691612
H	-4.8210463	0.6497995	3.3310303
H	-2.3425002	0.5151240	3.0466631
H	-2.7787729	1.4666498	-1.1727490
H	-5.2587493	1.6098494	-0.8848484
H	-0.8957280	2.7306852	-0.1336224

$vibrational spectrum

# mode	symmetry	wave number	IR intensity	selection rules	
#		cm**(-1)	km/mol	IR	RAMAN
1		0.00	0.00000	-	-
2		0.00	0.00000	-	-
3		0.00	0.00000	-	-

4		0.00	0.00000	-	-
5		0.00	0.00000	-	-
6		0.00	0.00000	-	-
7	a	5.23	0.02192	YES	YES
8	a	16.44	0.10699	YES	YES
9	a	38.20	0.10104	YES	YES
10	a	42.72	0.17829	YES	YES
11	a	54.28	0.13952	YES	YES
12	a	54.98	0.02477	YES	YES
13	a	69.67	0.51001	YES	YES
14	a	79.26	0.02376	YES	YES
15	a	85.42	0.70115	YES	YES
16	a	94.94	0.21828	YES	YES
17	a	99.78	0.07365	YES	YES
18	a	104.02	0.58385	YES	YES
19	a	112.90	0.20273	YES	YES
20	a	127.26	0.35518	YES	YES
21	a	157.98	1.20516	YES	YES
22	a	169.08	0.84161	YES	YES
23	a	175.46	2.55549	YES	YES
24	a	203.10	0.94757	YES	YES
25	a	216.55	1.45099	YES	YES
26	a	229.83	0.29463	YES	YES
27	a	272.63	3.00912	YES	YES
28	a	305.65	0.53639	YES	YES
29	a	312.82	13.43462	YES	YES
30	a	353.75	0.62675	YES	YES
31	a	397.88	2.80142	YES	YES
32	a	400.74	9.57004	YES	YES
33	a	404.39	1.69232	YES	YES
34	a	419.99	0.68333	YES	YES
35	a	447.42	0.40847	YES	YES
36	a	455.05	2.94910	YES	YES
37	a	471.57	3.81417	YES	YES
38	a	482.56	9.46046	YES	YES
39	a	483.31	3.76855	YES	YES
40	a	490.41	6.60203	YES	YES
41	a	496.41	2.36391	YES	YES
42	a	499.52	17.06561	YES	YES
43	a	504.97	7.12513	YES	YES
44	a	520.67	15.02069	YES	YES
45	a	535.37	8.58747	YES	YES
46	a	560.04	8.32210	YES	YES
47	a	586.03	3.19765	YES	YES
48	a	610.13	0.63566	YES	YES
49	a	612.40	6.56616	YES	YES
50	a	619.97	64.61582	YES	YES

2ia

SCF Energy (au) BP86/SV(P)	-2183.122606432
SCF Energy (au) PBE0/def2-TZVPP	-2182.706625903
SCF Energy (au) PBE0/def2-TZVPP	-2182.7132650811 (Toluene Correction)
Zero Point Energy (au)	0.2582288
Chemical Potential (kJ mol^{-1})	536.57
Dispersion Correction (au) PBE0/def2-TZVPP	-0.05063461

xyz coordinates
37

Mn	1.0276323	0.3512433	0.9392188
C	1.1370934	0.6341023	2.7177790
C	2.7601907	-0.0495923	0.8708951
C	1.3101813	2.1264881	0.5941055
O	1.5153634	3.2602778	0.4386312
O	3.8873994	-0.3513020	0.8262138
O	1.2212734	0.7993244	3.8667371
C	-1.7321304	-0.3567563	1.3365823
C	-3.2367423	-0.2400642	1.3456914
C	-1.0221630	-1.5557674	1.7671824
C	0.4036238	-1.4598931	1.6766759
C	1.1509095	-2.5788773	2.1038483
C	0.5148944	-3.7386671	2.5790587
C	-0.8932832	-3.8219904	2.6497290
C	-1.6626598	-2.7286071	2.2444057
H	2.2532812	-2.5552050	2.0663755
H	1.1268527	-4.5975959	2.9050630
H	-1.3780831	-4.7378682	3.0247820
H	-2.7632359	-2.7795799	2.2979940
O	-1.0463227	0.6258788	0.9473827
C	0.5587890	1.3296505	-2.2859772
C	1.3550578	1.2794408	-3.4595080
C	1.1786239	2.2285641	-4.4762772
C	0.2087685	3.2381839	-4.3436773
C	-0.5854823	3.2952924	-3.1842105
C	-0.4119255	2.3550292	-2.1598384
H	2.1150387	0.4882493	-3.5600353
H	1.8061901	2.1801339	-5.3817235
H	0.0737329	3.9844462	-5.1440526
H	-1.3470002	4.0851299	-3.0747019
H	-1.0301008	2.3943804	-1.2493785
C	0.7254424	0.3200752	-1.2659808
C	0.8311265	-0.8453558	-0.7786278
H	0.8181768	-1.9306874	-0.8852514
H	-3.5372666	0.7642561	0.9869631
H	-3.6380547	-0.3971449	2.3717041
H	-3.6951916	-1.0151920	0.6922219

# mode	symmetry	wave number	IR intensity	selection rules	
#		cm**(-1)	km/mol	IR	RAMAN
1		0.00	0.00000	-	-
2		0.00	0.00000	-	-
3		0.00	0.00000	-	-
4		0.00	0.00000	-	-
5		0.00	0.00000	-	-
6		0.00	0.00000	-	-
7	a	22.87	0.42459	YES	YES
8	a	33.85	0.18411	YES	YES
9	a	46.95	0.12673	YES	YES
10	a	57.10	0.04978	YES	YES
11	a	67.42	0.90009	YES	YES

12	a	77.84	0.13583	YES	YES
13	a	88.20	0.66367	YES	YES
14	a	95.47	0.27775	YES	YES
15	a	106.01	0.31080	YES	YES
16	a	109.13	0.52012	YES	YES
17	a	117.66	0.08236	YES	YES
18	a	119.11	0.12682	YES	YES
19	a	132.46	0.08321	YES	YES
20	a	169.13	3.07848	YES	YES
21	a	170.62	1.97929	YES	YES
22	a	210.75	1.15734	YES	YES
23	a	222.46	1.43356	YES	YES
24	a	242.47	1.64021	YES	YES
25	a	254.89	0.37087	YES	YES
26	a	288.22	0.78470	YES	YES
27	a	325.61	19.55637	YES	YES
28	a	402.00	0.15435	YES	YES
29	a	405.50	0.34124	YES	YES
30	a	411.25	1.72472	YES	YES
31	a	418.63	4.36502	YES	YES
32	a	440.28	9.11232	YES	YES
33	a	459.61	9.70023	YES	YES
34	a	470.59	1.75045	YES	YES
35	a	477.20	2.96730	YES	YES
36	a	488.01	10.85217	YES	YES
37	a	495.04	2.33308	YES	YES
38	a	505.71	2.73942	YES	YES
39	a	519.54	19.67222	YES	YES
40	a	524.75	36.41111	YES	YES
41	a	536.80	13.03176	YES	YES
42	a	548.38	3.82310	YES	YES
43	a	600.77	14.13686	YES	YES
44	a	608.60	4.43846	YES	YES
45	a	610.84	39.97431	YES	YES
46	a	613.49	3.98419	YES	YES
47	a	633.19	2.48601	YES	YES
48	a	649.36	55.39115	YES	YES
49	a	661.00	16.37113	YES	YES
50	a	677.34	63.83913	YES	YES

2ia′

SCF Energy (au) BP86/SV(P)	-2183.119255411	
SCF Energy (au) PBE0/def2-TZVPP	-2182.703507634	
SCF Energy (au) PBE0/def2-TZVPP	-2182.7102422094	(Toluene Correction)
Zero Point Energy (au)	0.2582085	
Chemical Potential (kJ mol^{-1})	535.77	
Dispersion Correction (au) PBE0/def2-TZVPP	-0.05302657	

xyz coordinates
37

Mn	0.8983506	2.1448421	0.2688325
C	0.9141654	2.6039852	2.0158285
C	2.6524396	1.8402962	0.2910139
C	1.0410226	3.8978391	-0.2149611
O	1.1379995	5.0217186	-0.4998359
O	3.7932662	1.5904865	0.3081505
O	0.9364859	2.9056166	3.1391041
C	-1.8075626	1.2690843	0.7174211
C	-3.3169564	1.2705979	0.7003573
C	-1.0157638	0.1978747	1.3120318
C	0.3991048	0.3910100	1.2188449
C	1.2199172	-0.5869534	1.8179770
C	0.6658534	-1.7165200	2.4456782
C	-0.7312506	-1.9033912	2.5086360
C	-1.5737069	-0.9425776	1.9432115
H	2.3172948	-0.4767215	1.8023292
H	1.3356915	-2.4674974	2.8992107
H	-1.1522350	-2.7932599	3.0039253
H	-2.6680678	-1.0712190	1.9941553
O	-1.1936215	2.2423079	0.2064007
H	0.8117751	2.8996123	-2.6174311
C	0.7709592	2.1278030	-1.8471192
C	0.7261974	0.9043305	-1.5060129
C	0.5963222	-0.5095824	-1.7921790
H	-3.6832412	2.1744501	0.1742166
H	-3.7192499	1.2647297	1.7380287
H	-3.7087963	0.3619751	0.1922516
C	0.3439853	-3.2490311	-2.4608312
C	-0.6892896	-2.3484622	-2.7736553
C	-0.5671662	-0.9900332	-2.4453026
C	1.6281109	-1.4273549	-1.4744203
C	1.5025775	-2.7816281	-1.8145887
H	0.2475396	-4.3157889	-2.7222773
H	-1.5990167	-2.7059184	-3.2843124
H	-1.3704902	-0.2800854	-2.6988473
H	2.5380412	-1.0612958	-0.9746253
H	2.3193148	-3.4812396	-1.5712059

# mode	symmetry	wave number	IR intensity	selection rules IR	selection rules RAMAN
#		cm**(-1)	km/mol	IR	RAMAN
1		0.00	0.00000	-	-
2		0.00	0.00000	-	-
3		0.00	0.00000	-	-
4		0.00	0.00000	-	-
5		0.00	0.00000	-	-
6		0.00	0.00000	-	-
7	a	16.26	0.07232	YES	YES
8	a	27.27	0.39170	YES	YES
9	a	49.44	0.19270	YES	YES
10	a	59.02	0.06082	YES	YES
11	a	63.52	0.04027	YES	YES

12	a	73.78	1.26466	YES	YES
13	a	88.99	0.65309	YES	YES
14	a	97.88	0.16892	YES	YES
15	a	104.69	0.28633	YES	YES
16	a	115.69	0.22641	YES	YES
17	a	117.80	0.85020	YES	YES
18	a	127.98	0.10742	YES	YES
19	a	131.86	0.42863	YES	YES
20	a	165.40	1.61920	YES	YES
21	a	182.02	1.60815	YES	YES
22	a	201.91	0.59779	YES	YES
23	a	222.49	1.74088	YES	YES
24	a	231.59	1.17169	YES	YES
25	a	254.48	0.63146	YES	YES
26	a	291.18	0.61839	YES	YES
27	a	328.62	12.16511	YES	YES
28	a	400.08	0.31887	YES	YES
29	a	406.66	6.49271	YES	YES
30	a	409.60	3.66119	YES	YES
31	a	416.98	3.55152	YES	YES
32	a	450.74	2.33025	YES	YES
33	a	460.02	1.33503	YES	YES
34	a	470.60	3.95403	YES	YES
35	a	478.52	6.71100	YES	YES
36	a	492.43	11.27503	YES	YES
37	a	497.15	3.54688	YES	YES
38	a	502.61	9.37704	YES	YES
39	a	511.36	10.56242	YES	YES
40	a	526.42	16.87395	YES	YES
41	a	535.87	6.33441	YES	YES
42	a	566.15	5.67608	YES	YES
43	a	601.71	15.77670	YES	YES
44	a	608.29	5.91649	YES	YES
45	a	612.50	14.51577	YES	YES
46	a	616.09	56.93462	YES	YES
47	a	633.33	2.95790	YES	YES
48	a	647.28	41.26479	YES	YES
49	a	652.92	19.21428	YES	YES
50	a	679.40	77.09036	YES	YES

2ja

SCF Energy (au) BP86/SV(P)	-2452.080768239
SCF Energy (au) PBE0/def2-TZVPP	-2451.620892726
SCF Energy (au) PBE0/def2-TZVPP	-2451.6286920118 (Toluene Correction)
Zero Point Energy (au)	0.3431713
Chemical Potential (kJ mol^{-1})	738.84
Dispersion Correction (au) PBE0/def2-TZVPP	-0.06762876

xyz coordinates
48

Mn	1.3428078	0.8045502	0.5807547
C	1.3783701	1.2227344	2.3336199
C	3.1025940	0.4977164	0.5670520
C	1.5688369	2.5632149	0.1210029
O	1.7224449	3.6913483	-0.1112692
O	4.2521930	0.2995053	0.5355771
O	1.4098099	1.4889051	3.4667047
C	-1.3953526	0.0611476	0.8785204
C	-2.8797304	0.1605952	0.8509716
C	-0.6783374	-1.0867017	1.3610949
C	0.7053038	-0.9939985	1.3398313
C	1.5223928	-2.1348688	1.7934017
O	-0.7101442	1.0584446	0.4739061
C	0.9310357	1.5353758	-2.7143732
C	1.7985379	1.5006747	-3.8368380
C	1.6046139	2.3843498	-4.9078489
C	0.5482648	3.3121502	-4.8796436
C	-0.3155739	3.3537136	-3.7703583
C	-0.1271098	2.4784202	-2.6927061
H	2.6264471	0.7741179	-3.8549566
H	2.2867829	2.3491936	-5.7734986
H	0.3997020	4.0069114	-5.7228199
H	-1.1450905	4.0795798	-3.7432425
H	-0.7977290	2.5028749	-1.8197662
C	1.1209643	0.5937250	-1.6368683
C	1.2891557	-0.5235868	-1.0649263
H	1.3650205	-1.6113508	-1.0932481
H	-1.2044599	-1.9612831	1.7807001
C	-5.6935604	0.4280399	0.7868146
C	-4.8732260	1.5569739	0.6043783
C	-3.4791073	1.4241220	0.6323170
C	-3.7145290	-0.9672559	1.0353129
C	-5.1103379	-0.8335910	0.9991676
H	-6.7910934	0.5322035	0.7635136
H	-5.3271954	2.5483246	0.4407592
H	-2.8247368	2.2988518	0.4958738
H	-3.2728656	-1.9635792	1.1913143
H	-5.7485923	-1.7217179	1.1370152
C	3.0580331	-4.3695410	2.6573743
C	3.4298663	-3.0650970	3.0284627
C	2.6845134	-1.9624397	2.5882318
C	1.1570995	-3.4620276	1.4377184
C	1.9212272	-4.5624642	1.8534064
H	3.6573577	-5.2335446	2.9890534
H	4.3184064	-2.9022364	3.6605160
H	2.9930370	-0.9489848	2.8854907
H	0.2687670	-3.6236172	0.8047863
H	1.6251862	-5.5798783	1.5477199

$vibrational spectrum

# mode	symmetry	wave number cm**(-1)	IR intensity km/mol	selection rules IR	RAMAN
1		0.00	0.00000	-	-
2		0.00	0.00000	-	-
3		0.00	0.00000	-	-
4		0.00	0.00000	-	-
5		0.00	0.00000	-	-
6		0.00	0.00000	-	-
7	a	19.34	0.18328	YES	YES
8	a	29.78	0.20821	YES	YES
9	a	32.91	0.04331	YES	YES
10	a	34.27	0.08747	YES	YES
11	a	41.54	0.19343	YES	YES
12	a	48.66	0.55510	YES	YES
13	a	57.82	0.19647	YES	YES
14	a	63.27	0.31881	YES	YES
15	a	74.34	0.11516	YES	YES
16	a	92.61	0.34213	YES	YES
17	a	95.48	0.14209	YES	YES
18	a	98.31	1.04767	YES	YES
19	a	104.50	0.24461	YES	YES
20	a	116.99	0.52529	YES	YES
21	a	124.52	0.20012	YES	YES
22	a	145.07	0.11716	YES	YES
23	a	154.08	0.65564	YES	YES
24	a	175.28	4.49094	YES	YES
25	a	193.75	0.92811	YES	YES
26	a	215.30	0.74143	YES	YES
27	a	221.97	0.42138	YES	YES
28	a	232.00	1.96209	YES	YES
29	a	259.41	0.65432	YES	YES
30	a	265.94	0.65064	YES	YES
31	a	318.81	19.77363	YES	YES
32	a	364.90	3.81450	YES	YES
33	a	396.90	1.90010	YES	YES
34	a	399.97	0.14845	YES	YES
35	a	401.99	0.35826	YES	YES
36	a	405.08	2.76880	YES	YES
37	a	417.70	8.06474	YES	YES
38	a	441.71	6.07106	YES	YES
39	a	453.03	1.48347	YES	YES
40	a	468.08	5.77371	YES	YES
41	a	476.58	1.52319	YES	YES
42	a	485.08	6.65317	YES	YES
43	a	488.75	9.69227	YES	YES
44	a	503.43	1.20446	YES	YES
45	a	509.22	27.45127	YES	YES
46	a	521.79	15.92382	YES	YES
47	a	534.58	10.70216	YES	YES
48	a	539.24	4.13881	YES	YES
49	a	563.79	5.43094	YES	YES
50	a	575.98	25.67458	YES	YES

3aa

```
SCF Energy (au) BP86/SV(P)                     -2427.897218753
SCF Energy (au) PBE0/def2-TZVPP                -2427.461062227
SCF Energy (au) PBE0/def2-TZVPP                -2427.4722378136 (Toluene Correction)
Zero Point Energy (au)                         0.2981519
Chemical Potential (kJ mol-1)                  630.17
Dispersion Correction (au) PBE0/def2-TZVPP -0.06351537

xyz coordinates
43

Mn    1.1810811    0.7159650    0.5661474
C     1.6598767    0.9219215    2.3218986
C     2.8124931    0.1034934    0.0887737
C     1.6285850    2.3714621    0.1740137
O     1.8910188    3.4873282   -0.0653848
O     3.8664842   -0.2685205   -0.2369390
O     1.9970452    1.0639864    3.4301209
C    -1.3639759    0.0004371    1.3595888
C    -1.5344690    2.2323180    0.6746315
C    -2.8999818    2.2629891    0.9887676
C    -3.5043130    1.1126164    1.5220845
C    -2.7223835   -0.0387250    1.7130407
C    -0.4323711   -1.1556316    1.4903727
C     0.2282191   -1.6852839    0.3595376
C     0.9522997   -2.9703101    0.5092773
O     1.0602009   -3.4894198    1.8241345
C     0.4521165   -2.9346565    2.9014701
C    -0.3120505   -1.8039179    2.7701581
H    -1.0105112    3.1111142    0.2663304
H    -3.4764221    3.1855249    0.8182644
H    -4.5749929    1.1091453    1.7824192
H    -3.1549511   -0.9672730    2.1167805
O     1.4408513   -3.6214689   -0.3846887
C     0.7252502   -3.6793587    4.1700191
H    -0.7993238   -1.3709488    3.6556688
N    -0.7869478    1.1259126    0.8682712
C     0.4306811    0.7439951   -2.5882684
C     1.4333479    1.6346543   -3.0505655
C     1.3607196    2.2088200   -4.3280906
C     0.2732415    1.9259245   -5.1739001
C    -0.7390712    1.0556150   -4.7303850
C    -0.6602454    0.4704662   -3.4584866
H     2.2928458    1.8603887   -2.4004361
H     2.1611960    2.8881582   -4.6656384
H     0.2122367    2.3866509   -6.1738962
H    -1.6002679    0.8323011   -5.3826107
H    -1.4631317   -0.2007728   -3.1113904
C     0.5017330    0.1159829   -1.2647706
C     0.1302878   -1.1756697   -1.0245816
H    -0.0776536   -1.9218985   -1.8186780
H     0.1921505   -3.2179402    5.0253523
H     1.8172426   -3.6823528    4.3875594
H     0.4118588   -4.7430222    4.0740278

$vibrational spectrum
#  mode     symmetry     wave number   IR intensity    selection rules
#                         cm**(-1)        km/mol         IR     RAMAN
     1                        0.00        0.00000         -       -
     2                        0.00        0.00000         -       -
     3                        0.00        0.00000         -       -
     4                        0.00        0.00000         -       -
     5                        0.00        0.00000         -       -
```

6		0.00	0.00000	-	-
7	a	27.13	0.22894	YES	YES
8	a	31.31	1.15888	YES	YES
9	a	38.64	0.09170	YES	YES
10	a	47.06	3.23123	YES	YES
11	a	51.03	0.03444	YES	YES
12	a	65.80	0.31219	YES	YES
13	a	70.64	0.62300	YES	YES
14	a	83.70	0.71523	YES	YES
15	a	85.74	0.30667	YES	YES
16	a	98.66	0.91459	YES	YES
17	a	100.41	0.85868	YES	YES
18	a	105.58	0.32114	YES	YES
19	a	122.03	0.32392	YES	YES
20	a	142.13	0.21008	YES	YES
21	a	147.67	1.31379	YES	YES
22	a	154.77	0.60684	YES	YES
23	a	164.90	0.61796	YES	YES
24	a	168.59	1.02392	YES	YES
25	a	204.03	1.34226	YES	YES
26	a	234.09	0.82969	YES	YES
27	a	264.90	0.57805	YES	YES
28	a	271.62	0.42055	YES	YES
29	a	287.09	2.49634	YES	YES
30	a	306.14	0.94595	YES	YES
31	a	337.11	1.05520	YES	YES
32	a	370.70	2.97965	YES	YES
33	a	395.16	8.92298	YES	YES
34	a	408.27	0.68182	YES	YES
35	a	432.09	0.64404	YES	YES
36	a	450.92	2.19969	YES	YES
37	a	458.61	0.68661	YES	YES
38	a	471.99	2.82716	YES	YES
39	a	480.60	1.00547	YES	YES
40	a	486.92	0.72672	YES	YES
41	a	495.74	7.17728	YES	YES
42	a	498.30	3.06704	YES	YES
43	a	520.60	3.47485	YES	YES
44	a	534.57	2.34487	YES	YES
45	a	543.14	2.48022	YES	YES
46	a	552.57	3.12640	YES	YES
47	a	564.04	1.11003	YES	YES
48	a	599.29	2.19529	YES	YES
49	a	613.24	1.04141	YES	YES
50	a	616.73	25.69378	YES	YES

3aa′

```
SCF Energy (au) BP86/SV(P)                   -2427.892694005
SCF Energy (au) PBE0/def2-TZVPP              -2427.457091685
SCF Energy (au) PBE0/def2-TZVPP              -2427.4689841208 (Toluene Correction)
Zero Point Energy (au)                       0.2981446
Chemical Potential (kJ mol-1)                631.41
Dispersion Correction (au) PBE0/def2-TZVPP -0.06308410

xyz coordinates
43

Mn    1.3171493    1.8585225    0.5296280
C     1.6298756    2.1496790    2.3126624
C     3.0307253    1.3974938    0.2029409
C     1.6464497    3.5272692    0.0470398
O     1.8291102    4.6279022   -0.3021686
O     4.1385152    1.1270452   -0.0289385
O     1.8701931    2.3678615    3.4338012
C    -1.1847137    0.8917145    1.0692971
C    -1.5637281    3.1280814    0.5011556
C    -2.9455932    2.9756846    0.6882717
C    -3.4474210    1.7276430    1.0919780
C    -2.5502800    0.6634518    1.2902467
C    -0.0901566   -0.1110759    1.2427466
C     0.7083217   -0.4934816    0.1437899
C     1.7304981   -1.5486002    0.3443189
O     1.8298837   -2.0854507    1.6614893
C     1.0341846   -1.7118421    2.6908435
C     0.0741113   -0.7478401    2.5256006
H    -1.1168883    4.0879769    0.1958499
H    -3.6155793    3.8331442    0.5201520
H    -4.5283614    1.5817486    1.2486727
H    -2.8986445   -0.3336100    1.6009322
O     2.4712274   -2.0071104   -0.4899417
C     1.3433680   -2.4441814    3.9587961
H    -0.5466861   -0.4406024    3.3791964
N    -0.7096453    2.1048080    0.6960981
H     0.8184261    1.8829236   -2.3052401
C     0.8588862    1.3098571   -1.3604264
C     0.5171987   -0.0004278   -1.2695444
C     0.0323389   -0.9242508   -2.3214178
H     0.6553275   -2.1414774    4.7733723
H     2.3902219   -2.2377382    4.2764703
H     1.2642493   -3.5430963    3.8012614
C    -0.9610834   -2.6689967   -4.3362568
C    -0.2837614   -1.4860245   -4.6880366
C     0.2098940   -0.6288371   -3.6958179
C    -0.6469555   -2.1199279   -1.9851720
C    -1.1373039   -2.9824307   -2.9782057
H    -1.3423396   -3.3459983   -5.1185200
H    -0.1273649   -1.2356769   -5.7508757
H     0.7615512    0.2801757   -3.9854026
H    -0.8033312   -2.3761770   -0.9232676
H    -1.6618697   -3.9081283   -2.6873795

$vibrational spectrum
#  mode     symmetry     wave number   IR intensity    selection rules
#                         cm**(-1)        km/mol         IR     RAMAN
     1                        0.00        0.00000         -       -
     2                        0.00        0.00000         -       -
     3                        0.00        0.00000         -       -
     4                        0.00        0.00000         -       -
     5                        0.00        0.00000         -       -
```

6		0.00	0.00000	-	-
7	a	29.19	0.39689	YES	YES
8	a	37.85	0.18530	YES	YES
9	a	42.04	0.53987	YES	YES
10	a	45.28	2.33284	YES	YES
11	a	55.60	0.07883	YES	YES
12	a	71.08	1.12178	YES	YES
13	a	72.97	0.64680	YES	YES
14	a	81.99	0.64645	YES	YES
15	a	89.08	0.10909	YES	YES
16	a	92.22	0.21892	YES	YES
17	a	97.74	1.15302	YES	YES
18	a	104.90	0.38369	YES	YES
19	a	125.99	0.51286	YES	YES
20	a	140.22	2.69414	YES	YES
21	a	149.69	0.95855	YES	YES
22	a	161.56	1.03869	YES	YES
23	a	166.50	1.04911	YES	YES
24	a	189.68	0.39795	YES	YES
25	a	206.72	0.93187	YES	YES
26	a	226.68	0.48747	YES	YES
27	a	253.12	0.35894	YES	YES
28	a	281.86	1.61657	YES	YES
29	a	285.37	0.53583	YES	YES
30	a	303.63	3.70548	YES	YES
31	a	340.15	0.60494	YES	YES
32	a	346.30	1.04459	YES	YES
33	a	403.46	0.16006	YES	YES
34	a	423.67	1.61991	YES	YES
35	a	429.09	3.27110	YES	YES
36	a	452.70	3.31077	YES	YES
37	a	466.50	1.35906	YES	YES
38	a	473.72	1.84264	YES	YES
39	a	484.93	2.26677	YES	YES
40	a	487.33	1.39886	YES	YES
41	a	502.24	2.90579	YES	YES
42	a	519.55	11.89092	YES	YES
43	a	524.12	11.54128	YES	YES
44	a	535.06	2.93645	YES	YES
45	a	543.20	2.59277	YES	YES
46	a	556.86	3.38187	YES	YES
47	a	573.17	2.88697	YES	YES
48	a	600.66	33.06235	YES	YES
49	a	612.22	2.75040	YES	YES
50	a	619.54	32.55359	YES	YES

3ba

SCF Energy (au) BP86/SV(P)	-2277.545461326	
SCF Energy (au) PBE0/def2-TZVPP	-2277.104429156	
SCF Energy (au) PBE0/def2-TZVPP	-2277.1124730146	(Toluene Correction)
Zero Point Energy (au)	0.2916084	
Chemical Potential (kJ mol^{-1})	622.44	
Dispersion Correction (au) PBE0/def2-TZVPP	-0.06485876	

xyz coordinates
41

Mn	1.2035844	0.6105743	0.8523064
C	1.6744913	0.8965522	2.5994236
C	2.8473894	0.0456107	0.3779394
C	1.5909450	2.2597372	0.4049332
O	1.8148923	3.3744824	0.1207876
O	3.9082864	-0.3109878	0.0515739
O	2.0053768	1.0941946	3.7003449
C	-1.3227434	-0.2381294	1.6401022
C	-1.5436521	2.0144170	1.0274230
C	-2.9081022	2.0114590	1.3451955
C	-3.4882403	0.8327062	1.8409466
C	-2.6826732	-0.3079262	1.9888013
C	-0.3856671	-1.3933261	1.7627290
C	0.2684065	-1.9506208	0.6110439
C	1.0047266	-3.1538226	0.7947706
C	1.1268247	-3.7633542	2.0454734
C	0.5024462	-3.1928078	3.1746501
C	-0.2550789	-2.0287122	3.0254885
H	-1.0387463	2.9154439	0.6443993
H	-3.5013098	2.9283945	1.2048881
H	-4.5582313	0.7983263	2.1023647
H	-3.0974068	-1.2591955	2.3566214
H	1.4894977	-3.6036451	-0.0875664
H	1.7150206	-4.6901783	2.1489020
H	0.6024968	-3.6636841	4.1659042
H	-0.7641460	-1.5865805	3.8980022
N	-0.7722237	0.9178227	1.1841217
C	0.4618578	0.5683655	-2.2841753
C	1.4959925	1.4046476	-2.7766900
C	1.4177823	1.9803452	-4.0533923
C	0.2933206	1.7557737	-4.8679978
C	-0.7509429	0.9425426	-4.3928556
C	-0.6667157	0.3551167	-3.1214676
H	2.3837147	1.5872005	-2.1510279
H	2.2430676	2.6166848	-4.4145145
H	0.2281842	2.2194463	-5.8664202
H	-1.6426649	0.7667664	-5.0182869
H	-1.4952286	-0.2693487	-2.7481429
C	0.5384954	-0.0687245	-0.9602239
C	0.1581614	-1.3606470	-0.7491599
H	-0.1011875	-2.0549193	-1.5772156

$vibrational spectrum

# mode	symmetry	wave number	IR intensity	selection rules	
#		cm**(-1)	km/mol	IR	RAMAN
1		0.00	0.00000	-	-
2		0.00	0.00000	-	-
3		0.00	0.00000	-	-
4		0.00	0.00000	-	-
5		0.00	0.00000	-	-
6		0.00	0.00000	-	-
7	a	30.29	0.17673	YES	YES

8	a	36.56	0.28054	YES	YES
9	a	45.25	0.15305	YES	YES
10	a	53.89	0.01637	YES	YES
11	a	59.66	0.18616	YES	YES
12	a	71.73	0.36929	YES	YES
13	a	75.91	0.28811	YES	YES
14	a	85.61	0.08148	YES	YES
15	a	95.08	1.34750	YES	YES
16	a	100.20	0.89084	YES	YES
17	a	106.07	0.11045	YES	YES
18	a	109.81	0.57321	YES	YES
19	a	145.93	0.39554	YES	YES
20	a	164.63	1.78407	YES	YES
21	a	176.37	0.65124	YES	YES
22	a	201.46	0.39912	YES	YES
23	a	225.54	0.91587	YES	YES
24	a	241.40	1.04718	YES	YES
25	a	290.50	0.84951	YES	YES
26	a	305.46	0.62450	YES	YES
27	a	340.85	1.99622	YES	YES
28	a	359.19	1.66423	YES	YES
29	a	407.29	0.27628	YES	YES
30	a	411.67	2.09351	YES	YES
31	a	430.32	2.45243	YES	YES
32	a	448.20	0.79627	YES	YES
33	a	458.90	0.39531	YES	YES
34	a	476.54	0.32414	YES	YES
35	a	479.07	5.08508	YES	YES
36	a	491.65	1.11876	YES	YES
37	a	505.52	7.79304	YES	YES
38	a	505.80	6.78980	YES	YES
39	a	534.99	0.55830	YES	YES
40	a	540.90	5.74478	YES	YES
41	a	548.34	4.66755	YES	YES
42	a	555.32	0.92651	YES	YES
43	a	564.16	0.93785	YES	YES
44	a	613.20	0.87636	YES	YES
45	a	621.73	35.32377	YES	YES
46	a	622.92	1.41328	YES	YES
47	a	630.49	26.57959	YES	YES
48	a	633.57	21.99769	YES	YES
49	a	644.97	6.16252	YES	YES
50	a	696.03	24.05804	YES	YES

3ba'

```
SCF Energy (au) BP86/SV(P)                  -2277.542842618
SCF Energy (au) PBE0/def2-TZVPP             -2277.102650356
SCF Energy (au) PBE0/def2-TZVPP             -2277.1108568307  (Toluene Correction)
Zero Point Energy (au)                      0.2916320
Chemical Potential (kJ mol-1)               621.89
Dispersion Correction (au)  PBE0/def2-TZVPP -0.06426298

xyz coordinates
41

Mn    1.4885199    1.7045119    0.8181604
C     1.8265077    2.1026966    2.5777115
C     3.1454414    1.0652797    0.5205397
C     1.9444044    3.2939881    0.2327303
O     2.2063876    4.3558369   -0.1870421
O     4.2131739    0.6509773    0.3046883
O     2.0828108    2.3739325    3.6828999
C    -1.0888999    0.8846646    1.3480670
C    -1.2672276    3.1519178    0.7822458
C    -2.6578844    3.1277864    0.9576351
C    -3.2731840    1.9291202    1.3523987
C    -2.4749497    0.7898686    1.5512143
C    -0.1314261   -0.2465627    1.5563800
C     0.5976287   -0.8229594    0.4644390
C     1.4042200   -1.9613386    0.7375116
C     1.5023706   -2.5030140    2.0212514
C     0.7823654   -1.9282723    3.0902786
C    -0.0299553   -0.8176980    2.8543520
H    -0.7337819    4.0677567    0.4807180
H    -3.2445334    4.0438220    0.7866494
H    -4.3641253    1.8787612    1.4995791
H    -2.9143293   -0.1741581    1.8509878
H     1.9574847   -2.4179608   -0.0991123
H     2.1457900   -3.3806853    2.1980356
H     0.8584594   -2.3507146    4.1052713
H    -0.6028304   -0.3658125    3.6809242
N    -0.5072126    2.0562722    0.9820539
H     1.0338613    1.5503658   -1.9838215
C     0.9936968    1.0196038   -1.0131345
C     0.5364100   -0.2610022   -0.9328100
C     0.0626639   -1.1237395   -2.0460919
C    -0.8974941   -2.7471965   -4.1820293
C    -1.2411541   -3.0468770   -2.8531537
C    -0.7671295   -2.2467719   -1.8009977
C     0.4052510   -0.8428378   -3.3933402
C    -0.0706512   -1.6385431   -4.4441504
H    -1.2654379   -3.3762956   -5.0093935
H    -1.8885083   -3.9120274   -2.6308809
H    -1.0556802   -2.4909255   -0.7649647
H     1.0720201    0.0063175   -3.6141987
H     0.2169276   -1.3980867   -5.4816012

$vibrational spectrum
#  mode     symmetry     wave number   IR intensity    selection rules
#                         cm**(-1)        km/mol          IR     RAMAN
     1                      0.00          0.00000        -        -
     2                      0.00          0.00000        -        -
     3                      0.00          0.00000        -        -
     4                      0.00          0.00000        -        -
     5                      0.00          0.00000        -        -
     6                      0.00          0.00000        -        -
     7        a            27.98          0.21003       YES      YES
```

8	a	35.88	0.03719	YES	YES
9	a	42.40	0.53053	YES	YES
10	a	53.71	0.17874	YES	YES
11	a	55.09	0.13841	YES	YES
12	a	75.80	0.23442	YES	YES
13	a	77.98	0.89256	YES	YES
14	a	86.90	0.33696	YES	YES
15	a	91.37	0.61055	YES	YES
16	a	95.14	0.10495	YES	YES
17	a	100.47	0.86115	YES	YES
18	a	108.09	0.34828	YES	YES
19	a	143.97	1.32281	YES	YES
20	a	172.24	0.81078	YES	YES
21	a	181.93	0.53638	YES	YES
22	a	212.56	0.77347	YES	YES
23	a	220.47	0.60037	YES	YES
24	a	260.51	0.31917	YES	YES
25	a	268.38	0.73732	YES	YES
26	a	316.09	0.36434	YES	YES
27	a	330.62	2.16754	YES	YES
28	a	355.25	0.15889	YES	YES
29	a	404.05	0.10322	YES	YES
30	a	415.22	2.55529	YES	YES
31	a	433.11	3.82045	YES	YES
32	a	454.83	1.38103	YES	YES
33	a	460.18	0.75567	YES	YES
34	a	471.42	3.55094	YES	YES
35	a	475.30	0.66048	YES	YES
36	a	491.02	1.76918	YES	YES
37	a	515.76	7.99125	YES	YES
38	a	517.19	10.00528	YES	YES
39	a	530.15	2.93397	YES	YES
40	a	543.68	6.69378	YES	YES
41	a	551.17	0.59449	YES	YES
42	a	572.72	7.04324	YES	YES
43	a	608.23	17.58280	YES	YES
44	a	612.63	17.55498	YES	YES
45	a	618.95	10.36268	YES	YES
46	a	624.31	3.62383	YES	YES
47	a	627.60	41.99112	YES	YES
48	a	636.32	10.37859	YES	YES
49	a	654.78	7.29210	YES	YES
50	a	693.78	26.60027	YES	YES

3bb

```
SCF Energy (au) BP86/SV(P)                    -2505.270959872
SCF Energy (au) PBE0/def2-TZVPP               -2504.828741005
SCF Energy (au) PBE0/def2-TZVPP               -2504.8384434576 (Toluene Correction)
Zero Point Energy (au)                        0.3336560
Chemical Potential (kJ mol-1)                 711.91
Dispersion Correction (au) PBE0/def2-TZVPP    -0.06961440

xyz coordinates
47
Mn    1.6477972    1.0275833    2.0605900
C     1.8976650    1.2694763    3.8608441
C     3.3020158    0.3626725    1.8050471
C     2.1780726    2.6512926    1.6683046
O     2.4926516    3.7462634    1.3914971
O     4.3695223   -0.0656815    1.6159589
O     2.0955334    1.4417486    4.9969535
C    -0.9715380    0.2473441    2.4287731
C    -1.0499351    2.5466755    1.9737597
C    -2.4450362    2.5677129    2.1013274
C    -3.1157768    1.3761657    2.4180761
C    -2.3670369    0.1994742    2.5838918
C    -0.0896805   -0.9507204    2.5898833
C     0.6469780   -1.4952711    1.4829103
C     1.2885120   -2.7487765    1.6746996
C     1.2464645   -3.4207830    2.8987807
C     0.5497269   -2.8619597    3.9908379
C    -0.1201860   -1.6472937    3.8279927
H    -0.4745746    3.4555027    1.7362322
H    -2.9910714    3.5119022    1.9514598
H    -4.2122378    1.3596749    2.5266123
H    -2.8516628   -0.7613711    2.8163959
H     1.8354101   -3.1864927    0.8232017
H     1.7644995   -4.3876886    3.0112949
H     0.5233452   -3.3822485    4.9618903
H    -0.6904628   -1.2151454    4.6669278
N    -0.3372492    1.4146770    2.1450053
C     1.4343270    1.2627304   -1.0908466
C     1.2152329    0.4607538    0.1506046
C     0.7394831   -0.8075673    0.1684062
H     0.5149719   -1.3942259   -0.7454592
H     2.5194542    1.4570064   -1.2394867
O     1.0014743    0.5985720   -2.3075364
H     0.9106454    2.2410176   -1.0240664
C    -0.2351374    0.9166963   -2.7772483
O    -0.9697675    1.7454896   -2.2628037
C    -0.5837036    0.1342317   -4.0072151
C    -1.3420827   -1.2767439   -6.3226452
C    -2.2212665   -0.3382637   -5.7518477
C    -1.8439204    0.3645661   -4.5991023
C     0.2965975   -0.8071567   -4.5836955
C    -0.0844649   -1.5091943   -5.7374509
H    -1.6386221   -1.8296577   -7.2298127
H    -3.2068873   -0.1543811   -6.2108652
H    -2.5123797    1.1056769   -4.1330868
H     1.2790814   -0.9804965   -4.1195620
H     0.6052185   -2.2437876   -6.1854283

$vibrational spectrum
#  mode     symmetry     wave number   IR intensity    selection rules
#                         cm**(-1)        km/mol          IR     RAMAN
     1                        0.00         0.00000         -       -
     2                        0.00         0.00000         -       -
```

3		0.00	0.00000	-	-
4		0.00	0.00000	-	-
5		0.00	0.00000	-	-
6		0.00	0.00000	-	-
7	a	10.78	1.10029	YES	YES
8	a	21.30	0.05131	YES	YES
9	a	25.19	0.81352	YES	YES
10	a	39.40	0.01817	YES	YES
11	a	42.14	0.18979	YES	YES
12	a	55.45	0.68925	YES	YES
13	a	57.90	0.38211	YES	YES
14	a	64.94	0.45207	YES	YES
15	a	74.83	0.18833	YES	YES
16	a	83.62	0.12810	YES	YES
17	a	86.40	0.08338	YES	YES
18	a	93.65	1.00408	YES	YES
19	a	95.93	0.76442	YES	YES
20	a	107.74	0.05519	YES	YES
21	a	117.15	0.46782	YES	YES
22	a	146.24	0.38746	YES	YES
23	a	155.71	0.99850	YES	YES
24	a	175.99	1.34711	YES	YES
25	a	190.00	1.81081	YES	YES
26	a	214.51	0.56787	YES	YES
27	a	237.60	1.83998	YES	YES
28	a	276.57	2.56669	YES	YES
29	a	290.70	0.88472	YES	YES
30	a	311.95	3.93747	YES	YES
31	a	331.38	17.34403	YES	YES
32	a	354.47	0.52693	YES	YES
33	a	365.80	4.48850	YES	YES
34	a	374.44	1.17807	YES	YES
35	a	404.85	0.01467	YES	YES
36	a	434.65	1.57511	YES	YES
37	a	441.25	0.43459	YES	YES
38	a	446.29	0.05822	YES	YES
39	a	455.43	0.59858	YES	YES
40	a	466.19	2.35891	YES	YES
41	a	475.71	2.30286	YES	YES
42	a	477.44	2.93109	YES	YES
43	a	489.91	1.94362	YES	YES
44	a	495.82	6.22667	YES	YES
45	a	511.62	5.83307	YES	YES
46	a	522.82	10.20532	YES	YES
47	a	538.10	1.68865	YES	YES
48	a	549.48	1.99689	YES	YES
49	a	576.38	3.85253	YES	YES
50	a	610.28	0.55554	YES	YES

3bb′

```
SCF Energy (au) BP86/SV(P)                    -2505.262474030
SCF Energy (au) PBE0/def2-TZVPP               -2504.821881763
SCF Energy (au) PBE0/def2-TZVPP               -2504.8324982709 (Toluene Correction)
Zero Point Energy (au)                        0.3332973
Chemical Potential (kJ mol-1)                 707.95
Dispersion Correction (au) PBE0/def2-TZVPP -0.06822560

xyz coordinates
47

Mn    1.4239376    1.6744286    1.8220771
C     1.8865455    2.2627008    3.4965058
C     3.1056942    1.2035792    1.3873430
C     1.6306999    3.2445483    1.0746210
O     1.7262686    4.2893934    0.5526978
O     4.1875927    0.8961212    1.0815572
O     2.2210702    2.6565818    4.5424955
C    -0.9950372    0.6248463    2.6735470
C    -1.4576079    2.8078057    1.9592185
C    -2.8176036    2.6651682    2.2663124
C    -3.2690759    1.4505949    2.8065614
C    -2.3417691    0.4156411    3.0134971
C     0.0764247   -0.3995511    2.8792772
C     0.7681943   -0.9957410    1.7734712
C     1.6808274   -2.0469290    2.0566569
C     1.9203980   -2.4827095    3.3635035
C     1.2444886   -1.8825392    4.4449250
C     0.3280167   -0.8567160    4.1995850
H    -1.0510607    3.7420424    1.5401705
H    -3.5079445    3.5031200    2.0827257
H    -4.3320018    1.3072214    3.0598091
H    -2.6511245   -0.5576994    3.4248795
H     2.2192233   -2.5183200    1.2184258
H     2.6446682   -3.2935919    3.5462002
H     1.4342444   -2.2179514    5.4773143
H    -0.2138813   -0.3881755    5.0377423
N    -0.5713820    1.8119713    2.1663599
H     0.7444662    1.1648687   -0.9101610
C     0.8359730    0.7575780    0.1133129
C     0.5410352   -0.5397815    0.3604814
C     0.0598516   -1.5885711   -0.6198576
H     0.7383530   -2.4730668   -0.6561824
O    -0.0366306   -1.0211831   -1.9346468
H    -0.9394416   -1.9890683   -0.3240467
C    -0.3792417   -1.8897228   -2.9238423
C    -0.4612079   -1.2276167   -4.2638339
O    -0.5948285   -3.0752282   -2.7243464
C    -0.6481598   -0.0957918   -6.8346892
C    -0.2881988    0.7066557   -5.7371038
C    -0.1944030    0.1455912   -4.4542450
C    -0.8217122   -2.0287012   -5.3688646
C    -0.9151381   -1.4646325   -6.6486519
H    -0.7204377    0.3484577   -7.8416054
H    -0.0772928    1.7790471   -5.8830349
H     0.0882734    0.7675542   -3.5916822
H    -1.0240530   -3.0975207   -5.1960703
H    -1.1970123   -2.0947084   -7.5084098

$vibrational spectrum
#  mode     symmetry     wave number   IR intensity    selection rules
#                         cm**(-1)        km/mol          IR     RAMAN
     1                        0.00          0.00000        -       -
```

2		0.00	0.00000	-	-
3		0.00	0.00000	-	-
4		0.00	0.00000	-	-
5		0.00	0.00000	-	-
6		0.00	0.00000	-	-
7	a	4.99	0.87779	YES	YES
8	a	14.98	0.49000	YES	YES
9	a	27.14	0.57272	YES	YES
10	a	41.47	0.03971	YES	YES
11	a	43.34	0.10929	YES	YES
12	a	50.05	0.78132	YES	YES
13	a	62.65	1.08905	YES	YES
14	a	73.73	0.24892	YES	YES
15	a	76.33	0.74414	YES	YES
16	a	80.36	0.19552	YES	YES
17	a	93.09	0.68049	YES	YES
18	a	93.15	0.12074	YES	YES
19	a	97.84	0.99353	YES	YES
20	a	107.09	0.42463	YES	YES
21	a	113.49	0.31746	YES	YES
22	a	138.60	0.77426	YES	YES
23	a	163.63	2.56034	YES	YES
24	a	165.67	1.61550	YES	YES
25	a	186.51	1.09764	YES	YES
26	a	199.15	1.39561	YES	YES
27	a	209.75	1.17396	YES	YES
28	a	255.17	3.35363	YES	YES
29	a	265.72	1.06408	YES	YES
30	a	299.42	3.21550	YES	YES
31	a	320.48	2.42530	YES	YES
32	a	331.95	3.75774	YES	YES
33	a	364.98	1.97094	YES	YES
34	a	379.24	1.10255	YES	YES
35	a	403.35	0.00028	YES	YES
36	a	434.60	4.97223	YES	YES
37	a	445.03	0.12190	YES	YES
38	a	451.78	0.68727	YES	YES
39	a	458.23	2.59165	YES	YES
40	a	474.34	0.99981	YES	YES
41	a	479.67	3.72907	YES	YES
42	a	482.22	7.97386	YES	YES
43	a	491.27	4.35792	YES	YES
44	a	515.35	9.18263	YES	YES
45	a	525.91	1.57433	YES	YES
46	a	529.50	3.93818	YES	YES
47	a	538.23	2.59232	YES	YES
48	a	562.36	3.58769	YES	YES
49	a	564.16	4.23410	YES	YES
50	a	610.15	0.24927	YES	YES

3bc

SCF Energy (au) BP86/SV(P) -2281.135995277
SCF Energy (au) PBE0/def2-TZVPP -2280.709892308
SCF Energy (au) PBE0/def2-TZVPP -2280.7163380043 (Toluene Correction)
Zero Point Energy (au) 0.3591834
Chemical Potential (kJ mol^{-1}) 796.63
Dispersion Correction (au) PBE0/def2-TZVPP -0.07061984

xyz coordinates
47

Mn	0.9786078	0.4084770	1.2223343
C	1.4551385	0.7317916	2.9633209
C	2.6487590	-0.0631594	0.7487877
C	1.2731209	2.0653396	0.7424884
O	1.4452458	3.1841802	0.4315049
O	3.7297294	-0.3705447	0.4367893
O	1.7956414	0.9609250	4.0552737
C	-1.4787280	-0.6152415	2.0577329
C	-1.8374708	1.6433332	1.5472334
C	-3.1878842	1.5579354	1.9092559
C	-3.6919294	0.3292394	2.3653486
C	-2.8228319	-0.7705066	2.4414410
C	-0.4767104	-1.7177011	2.1490224
C	0.2007391	-2.2195548	0.9882094
C	1.0166975	-3.3725104	1.1547545
C	1.1880167	-3.9861117	2.3980705
C	0.5382259	-3.4679615	3.5375738
C	-0.2931455	-2.3530503	3.4052073
H	-1.3909560	2.5879543	1.1990451
H	-3.8287663	2.4500780	1.8344837
H	-4.7498712	0.2270415	2.6566231
H	-3.1740538	-1.7555089	2.7856520
H	1.5223039	-3.7814818	0.2641371
H	1.8355437	-4.8741353	2.4873512
H	0.6770980	-3.9400769	4.5235565
H	-0.8203979	-1.9511518	4.2863317
N	-1.0056886	0.5825233	1.6225377
C	0.3155452	0.2669481	-1.9794547
C	0.3573697	-0.3225684	-0.5901665
C	0.0442766	-1.6255992	-0.3677651
H	-0.2105193	-2.3278742	-1.1932185
C	1.7013136	0.7314648	-2.4901721
H	-0.0187280	-0.5468414	-2.6714409
C	-0.7056419	1.4391834	-2.1074004
H	2.0152261	1.6281379	-1.9101778
C	1.6581457	1.0609065	-4.0037239
H	2.4660993	-0.0490734	-2.2881233
C	-0.3841621	2.3961246	-3.2829140
H	-0.7203774	2.0173101	-1.1577774
H	-1.7261741	1.0089775	-2.2249673
C	0.2917179	1.6487285	-4.4435602
H	0.2940312	3.2084799	-2.9339137
H	-1.3148005	2.8999546	-3.6280212
H	2.4749332	1.7797358	-4.2395862
H	1.8751732	0.1435980	-4.5970909
H	0.4245959	2.3216262	-5.3197810
H	-0.3844579	0.8306588	-4.7848120

```
$vibrational spectrum
#  mode     symmetry     wave number   IR intensity    selection rules
#                         cm**(-1)        km/mol         IR     RAMAN
     1                      0.00         0.00000         -       -
     2                      0.00         0.00000         -       -
     3                      0.00         0.00000         -       -
     4                      0.00         0.00000         -       -
     5                      0.00         0.00000         -       -
     6                      0.00         0.00000         -       -
     7         a           35.36         0.26877        YES     YES
     8         a           37.03         0.11738        YES     YES
     9         a           48.25         0.04837        YES     YES
    10         a           51.60         0.02182        YES     YES
    11         a           60.04         0.43157        YES     YES
    12         a           65.10         0.52792        YES     YES
    13         a           76.48         0.13860        YES     YES
    14         a           88.60         0.05733        YES     YES
    15         a           96.20         0.79255        YES     YES
    16         a          100.71         0.79076        YES     YES
    17         a          107.42         0.45034        YES     YES
    18         a          108.57         0.28856        YES     YES
    19         a          123.15         0.04763        YES     YES
    20         a          142.86         0.45196        YES     YES
    21         a          161.33         1.25089        YES     YES
    22         a          171.62         0.05950        YES     YES
    23         a          187.52         0.39619        YES     YES
    24         a          212.63         1.39349        YES     YES
    25         a          223.89         0.51838        YES     YES
    26         a          259.86         0.08106        YES     YES
    27         a          290.14         1.01879        YES     YES
    28         a          295.14         1.52142        YES     YES
    29         a          333.21         1.70194        YES     YES
    30         a          355.39         1.34461        YES     YES
    31         a          403.43         0.50782        YES     YES
    32         a          419.53         7.20342        YES     YES
    33         a          443.13         1.20729        YES     YES
    34         a          451.04         0.97650        YES     YES
    35         a          462.27         1.80215        YES     YES
    36         a          464.56         0.30656        YES     YES
    37         a          473.55         0.91469        YES     YES
    38         a          481.66         5.21201        YES     YES
    39         a          493.70         2.55763        YES     YES
    40         a          503.71         8.88712        YES     YES
    41         a          510.53         8.46111        YES     YES
    42         a          528.20         0.73778        YES     YES
    43         a          537.71         0.57584        YES     YES
    44         a          548.05         1.17087        YES     YES
    45         a          550.95         2.53395        YES     YES
    46         a          567.38         2.39919        YES     YES
    47         a          621.92        28.41054        YES     YES
    48         a          622.65         2.27148        YES     YES
    49         a          629.06        34.34545        YES     YES
    50         a          634.10         7.22001        YES     YES
```

3bc′

```
SCF Energy (au) BP86/SV(P)                    -2281.140662981
SCF Energy (au) PBE0/def2-TZVPP               -2280.715719323
SCF Energy (au) PBE0/def2-TZVPP               -2280.7223820342 (Toluene Correction)
Zero Point Energy (au)                        0.3590261
Chemical Potential (kJ mol-1)                 795.17
Dispersion Correction (au) PBE0/def2-TZVPP -0.06962776
```

```
xyz coordinates
47

Mn    1.7008294    1.9178340    1.2440537
C     1.9464929    2.2382414    3.0331653
C     3.3315730    1.1960531    1.0180863
C     2.2868389    3.4888898    0.7343215
O     2.6405981    4.5418127    0.3602348
O     4.3846566    0.7258024    0.8452115
O     2.1525111    2.4639907    4.1595807
C    -0.9782636    1.2554733    1.5666412
C    -0.9342414    3.5476428    1.0845970
C    -2.3307824    3.6335022    1.1661203
C    -3.0673510    2.4772899    1.4704893
C    -2.3784885    1.2697402    1.6733330
C    -0.1295595    0.0478783    1.8081275
C     0.6845288   -0.5178141    0.7703939
C     1.4519761   -1.6688415    1.1034235
C     1.3975069   -2.2547954    2.3717161
C     0.5680687   -1.7075987    3.3710144
C    -0.1827243   -0.5644054    3.0883288
H    -0.3100682    4.4267213    0.8574934
H    -2.8276646    4.6011997    0.9947426
H    -4.1662721    2.5132506    1.5450442
H    -2.9131136    0.3353467    1.9044298
H     2.1172542   -2.0946194    0.3373892
H     2.0127821   -3.1433835    2.5897974
H     0.5222532   -2.1657562    4.3723103
H    -0.8209259   -0.1154719    3.8673750
N    -0.2777518    2.3872866    1.2903563
H     1.3996283    1.8612120   -1.6028164
C     1.2783893    1.3182463   -0.6440395
C     0.7292965    0.0806393   -0.6157631
C     0.1674180   -0.6703298   -1.8229329
C     0.9487741   -1.9443464   -2.2328460
H     0.2553998    0.0463469   -2.6744401
C    -1.3354527   -1.0109098   -1.6610256
H     2.0263430   -1.6978684   -2.3658397
C     0.3755887   -2.5712595   -3.5174003
H     0.8866761   -2.6995264   -1.4142843
C    -1.9210119   -1.6584808   -2.9299712
H    -1.9023449   -0.0867089   -1.4056220
H    -1.4595226   -1.7099529   -0.7988130
C    -1.1197359   -2.8983627   -3.3627757
H    -1.9112953   -0.9089820   -3.7575255
H    -2.9896957   -1.9259817   -2.7643687
H     0.9474940   -3.4898021   -3.7824158
H     0.5125164   -1.8597757   -4.3661075
H    -1.5283127   -3.3092554   -4.3136337
H    -1.2408158   -3.7001716   -2.5951562
```

```
$vibrational spectrum
#  mode     symmetry     wave number   IR intensity    selection rules
#                         cm**(-1)        km/mol         IR     RAMAN
     1                      0.00         0.00000         -        -
     2                      0.00         0.00000         -        -
     3                      0.00         0.00000         -        -
     4                      0.00         0.00000         -        -
     5                      0.00         0.00000         -        -
     6                      0.00         0.00000         -        -
     7         a           23.50         0.32533        YES      YES
     8         a           31.99         0.01860        YES      YES
     9         a           44.13         0.24008        YES      YES
    10         a           51.06         0.22408        YES      YES
    11         a           51.84         0.35126        YES      YES
    12         a           69.06         0.38263        YES      YES
    13         a           77.41         0.09042        YES      YES
    14         a           87.88         0.23113        YES      YES
    15         a           93.53         0.67250        YES      YES
    16         a           96.45         0.52594        YES      YES
    17         a          100.05         0.08474        YES      YES
    18         a          106.99         0.49804        YES      YES
    19         a          131.13         0.27875        YES      YES
    20         a          157.94         0.90602        YES      YES
    21         a          169.99         1.48451        YES      YES
    22         a          203.07         0.56503        YES      YES
    23         a          210.99         0.25113        YES      YES
    24         a          220.28         0.40385        YES      YES
    25         a          238.63         0.03352        YES      YES
    26         a          268.33         0.02872        YES      YES
    27         a          306.85         0.86862        YES      YES
    28         a          314.02         0.39462        YES      YES
    29         a          337.59         1.09218        YES      YES
    30         a          353.62         1.49040        YES      YES
    31         a          389.14         0.87982        YES      YES
    32         a          428.20         1.24568        YES      YES
    33         a          436.17         3.80539        YES      YES
    34         a          439.55         1.18578        YES      YES
    35         a          458.00         1.66801        YES      YES
    36         a          463.29         0.79979        YES      YES
    37         a          471.05         1.32839        YES      YES
    38         a          476.24         1.22475        YES      YES
    39         a          491.21         2.23124        YES      YES
    40         a          515.51         7.94809        YES      YES
    41         a          518.27         3.62172        YES      YES
    42         a          522.65         6.67053        YES      YES
    43         a          537.87         3.65551        YES      YES
    44         a          546.13         2.31482        YES      YES
    45         a          582.34         2.57348        YES      YES
    46         a          604.22        21.23873        YES      YES
    47         a          620.00         8.90392        YES      YES
    48         a          627.22        38.83376        YES      YES
    49         a          629.79         7.92577        YES      YES
    50         a          637.71         8.14397        YES      YES
```

3bd

SCF Energy (au) BP86/SV(P)	-2411.413617596	
SCF Energy (au) PBE0/def2-TZVPP	-2410.960779076	
SCF Energy (au) PBE0/def2-TZVPP	-2410.9697888395	(Toluene Correction)
Zero Point Energy (au)	0.3619092	
Chemical Potential (kJ mol^{-1})	789.69	
Dispersion Correction (au)PBE0/def2-TZVPP	-0.07292669	

xyz coordinates

49

Mn	1.1580946	0.2533469	2.0022092
C	1.6348367	0.5380953	3.7484200
C	2.7925991	-0.3277689	1.5176508
C	1.5653154	1.8951322	1.5564534
O	1.8040972	3.0097385	1.2778736
O	3.8473452	-0.6948563	1.1821893
O	1.9709921	0.7309954	4.8490138
C	-1.4026967	-0.5179689	2.8343411
C	-1.5531558	1.7099038	2.1187935
C	-2.9141328	1.7686986	2.4427297
C	-3.5306391	0.6351080	2.9973705
C	-2.7627536	-0.5232472	3.1929938
C	-0.5156184	-1.7030816	2.9964020
C	0.1482835	-2.2987802	1.8677420
C	0.8337906	-3.5265776	2.0928654
C	0.9013429	-4.1209366	3.3544102
C	0.2704004	-3.5108000	4.4591501
C	-0.4399921	-2.3227292	4.2706203
H	-1.0218140	2.5749738	1.6915378
H	-3.4766530	2.6978981	2.2619131
H	-4.5994638	0.6490149	3.2655475
H	-3.2072983	-1.4427180	3.6042513
H	1.3250606	-4.0080314	1.2309236
H	1.4531173	-5.0665573	3.4859650
H	0.3288523	-3.9680692	5.4600933
H	-0.9536017	-1.8474087	5.1229669
N	-0.8152995	0.5974297	2.3241413
C	0.4378693	0.0965152	-1.1362323
C	1.4061952	1.0155642	-1.6139407
C	1.3827041	1.5187134	-2.9174912
C	0.3575769	1.1454161	-3.8349631
C	-0.6344641	0.2388594	-3.3574599
C	-0.5845963	-0.2648783	-2.0551730
H	2.2229875	1.3305442	-0.9460467
H	2.1765766	2.2157761	-3.2215062
N	0.3208918	1.6473731	-5.1284990
H	-1.4644746	-0.0724593	-4.0085269
H	-1.3814464	-0.9508668	-1.7224392
C	0.4823353	-0.4694157	0.2148392
C	0.0874268	-1.7501554	0.4903919
H	-0.1313716	-2.4849103	-0.3141261
C	-0.7211384	1.2196232	-6.0429968
C	1.3460939	2.5724649	-5.5760493
H	1.3697334	3.5021422	-4.9580529
H	1.1409724	2.8697347	-6.6241407
H	2.3674314	2.1205057	-5.5435120
H	-0.5728768	1.7113929	-7.0250688
H	-1.7409551	1.4924086	-5.6775268
H	-0.7084804	0.1148478	-6.2100478

```
$vibrational spectrum
#  mode     symmetry     wave number   IR intensity    selection rules
#                         cm**(-1)        km/mol         IR     RAMAN
     1                       0.00         0.00000         -       -
     2                       0.00         0.00000         -       -
     3                       0.00         0.00000         -       -
     4                       0.00         0.00000         -       -
     5                       0.00         0.00000         -       -
     6                       0.00         0.00000         -       -
     7         a            21.08         0.31267        YES     YES
     8         a            25.20         1.16210        YES     YES
     9         a            39.43         0.37353        YES     YES
    10         a            43.29         0.93927        YES     YES
    11         a            49.24         0.06683        YES     YES
    12         a            55.98         0.63821        YES     YES
    13         a            65.09         0.47275        YES     YES
    14         a            74.16         0.64624        YES     YES
    15         a            75.81         0.02333        YES     YES
    16         a            85.58         0.10151        YES     YES
    17         a            93.05         2.03898        YES     YES
    18         a            98.03         0.55357        YES     YES
    19         a           100.07         1.20359        YES     YES
    20         a           107.45         0.07850        YES     YES
    21         a           121.05         0.15239        YES     YES
    22         a           134.11         0.51650        YES     YES
    23         a           153.22         1.04832        YES     YES
    24         a           174.25         0.48049        YES     YES
    25         a           197.72         1.30506        YES     YES
    26         a           213.05         0.33096        YES     YES
    27         a           214.24         1.50010        YES     YES
    28         a           222.78         0.43599        YES     YES
    29         a           261.15         3.97850        YES     YES
    30         a           287.96         1.54180        YES     YES
    31         a           300.00         6.92838        YES     YES
    32         a           309.55         1.05348        YES     YES
    33         a           351.99         0.36710        YES     YES
    34         a           365.35         3.37415        YES     YES
    35         a           411.90         2.31770        YES     YES
    36         a           419.13         3.32007        YES     YES
    37         a           423.46         0.65970        YES     YES
    38         a           443.47         1.55412        YES     YES
    39         a           455.43         1.10299        YES     YES
    40         a           462.10         0.58427        YES     YES
    41         a           469.99         3.00002        YES     YES
    42         a           477.73         5.04355        YES     YES
    43         a           483.03        11.93955        YES     YES
    44         a           492.17         1.18165        YES     YES
    45         a           505.41         6.49846        YES     YES
    46         a           511.22         3.50146        YES     YES
    47         a           532.30         1.08792        YES     YES
    48         a           539.35         4.13962        YES     YES
    49         a           541.66        10.15662        YES     YES
    50         a           553.79         0.72502        YES     YES
```

3bd′

```
SCF Energy (au) BP86/SV(P)                  -2411.410447430
SCF Energy (au) PBE0/def2-TZVPP             -2410.9683234203
SCF Energy (au) PBE0/def2-TZVPP             -2410.958998524 (Toluene Correction)
Zero Point Energy (au)                      0.3619800
Chemical Potential (kJ mol-1)               789.09
Dispersion Correction (au) PBE0/def2-TZVPP  -0.07249849

xyz coordinates
49
Mn    1.7594811    2.3780870    1.7905522
C     2.1164445    2.7676239    3.5463937
C     3.4076023    1.7338603    1.4632533
C     2.2143365    3.9673582    1.2063758
O     2.4755736    5.0305307    0.7878539
O     4.4702473    1.3172361    1.2254476
O     2.3853366    3.0343150    4.6505127
C    -0.8169518    1.5653483    2.3349170
C    -0.9917541    3.8351141    1.7792212
C    -2.3812763    3.8152428    1.9639769
C    -2.9979374    2.6172537    2.3586792
C    -2.2019531    1.4746599    2.5473014
C     0.1367893    0.4292305    2.5314332
C     0.8530995   -0.1481106    1.4311450
C     1.6521145   -1.2944059    1.6949435
C     1.7573642   -1.8404689    2.9760550
C     1.0507163   -1.2637499    4.0532881
C     0.2446318   -0.1468652    3.8269131
H    -0.4570121    4.7502102    1.4776112
H    -2.9658978    4.7340201    1.8005746
H    -4.0880015    2.5700200    2.5135215
H    -2.6421761    0.5107777    2.8463223
H     2.1936939   -1.7523399    0.8514653
H     2.3962216   -2.7231491    3.1449226
H     1.1324764   -1.6894560    5.0665279
H    -0.3183687    0.3068091    4.6593589
N    -0.2338650    2.7364166    1.9696384
H     1.2873711    2.2305719   -1.0112830
C     1.2436783    1.6982825   -0.0416089
C     0.7855325    0.4171385    0.0359651
C     0.3205685   -0.4499935   -1.0740953
C    -0.6403717   -2.1028271   -3.2470443
C    -0.9730280   -2.3859338   -1.8917463
C    -0.5033633   -1.5792577   -0.8481322
C     0.6575204   -0.1811621   -2.4238388
C     0.1967957   -0.9714930   -3.4795579
N    -1.1012140   -2.8933772   -4.2925653
H    -1.6118688   -3.2452270   -1.6415554
H    -0.8003567   -1.8353762    0.1830288
H     1.3209240    0.6686278   -2.6539594
H     0.5086177   -0.7108120   -4.5012067
C    -0.7156781   -2.5868255   -5.6574505
C    -1.9011040   -4.0696289   -4.0099143
H    -1.3570415   -4.8108492   -3.3737167
H    -2.1675748   -4.5714991   -4.9617157
H    -2.8533116   -3.8128451   -3.4853204
H    -1.0397056   -1.5628749   -5.9622423
H    -1.1969285   -3.3097895   -6.3461398
H     0.3896025   -2.6504176   -5.8141060
```

```
$vibrational spectrum
#  mode     symmetry     wave number   IR intensity    selection rules
#                         cm**(-1)        km/mol         IR     RAMAN
     1                        0.00         0.00000        -       -
     2                        0.00         0.00000        -       -
     3                        0.00         0.00000        -       -
     4                        0.00         0.00000        -       -
     5                        0.00         0.00000        -       -
     6                        0.00         0.00000        -       -
     7        a              18.99         0.66596       YES     YES
     8        a              27.88         0.03399       YES     YES
     9        a              33.40         0.36254       YES     YES
    10        a              42.85         0.76193       YES     YES
    11        a              51.37         0.25743       YES     YES
    12        a              53.18         0.34361       YES     YES
    13        a              67.75         1.47722       YES     YES
    14        a              73.69         0.26541       YES     YES
    15        a              78.21         0.05442       YES     YES
    16        a              79.66         0.15106       YES     YES
    17        a              85.98         0.22070       YES     YES
    18        a              91.80         0.36237       YES     YES
    19        a              96.10         1.06830       YES     YES
    20        a             100.26         0.00276       YES     YES
    21        a             125.52         1.17836       YES     YES
    22        a             136.18         0.61147       YES     YES
    23        a             165.06         0.63800       YES     YES
    24        a             188.52         0.75308       YES     YES
    25        a             194.01         1.12099       YES     YES
    26        a             200.97         0.76149       YES     YES
    27        a             215.44         0.05230       YES     YES
    28        a             225.48         0.35162       YES     YES
    29        a             262.85         0.32014       YES     YES
    30        a             285.40         0.22519       YES     YES
    31        a             304.78         1.10759       YES     YES
    32        a             318.87         3.40583       YES     YES
    33        a             351.60         0.75539       YES     YES
    34        a             359.42         1.44456       YES     YES
    35        a             386.42         0.45695       YES     YES
    36        a             419.25         0.06425       YES     YES
    37        a             430.54         2.55204       YES     YES
    38        a             435.88         3.72607       YES     YES
    39        a             457.39         1.39231       YES     YES
    40        a             465.04         0.70328       YES     YES
    41        a             474.90         1.30233       YES     YES
    42        a             477.20         5.66843       YES     YES
    43        a             489.75         4.52679       YES     YES
    44        a             507.07         4.70699       YES     YES
    45        a             515.39         6.35917       YES     YES
    46        a             519.83        12.09470       YES     YES
    47        a             528.54         3.87924       YES     YES
    48        a             540.13         3.23204       YES     YES
    49        a             545.49         2.69293       YES     YES
    50        a             565.39         3.44381       YES     YES
```

3be

```
SCF Energy (au) BP86/SV(P)                    -2391.988619360
SCF Energy (au) PBE0/def2-TZVPP               -2391.5574436897
SCF Energy (au) PBE0/def2-TZVPP               -2391.548466315 (Toluene Correction)
Zero Point Energy (au)                        0.3230463
Chemical Potential (kJ mol-1)                 696.07
Dispersion Correction (au) PBE0/def2-TZVPP -0.06844294
```

```
xyz coordinates
45

Mn    1.0783337    0.3015995    1.4730911
C     1.5331549    0.5569657    3.2296976
C     2.7235407   -0.2613077    1.0018111
C     1.4768076    1.9552219    1.0594871
O     1.7082128    3.0750269    0.7988311
O     3.7858959   -0.6148355    0.6762550
O     1.8555017    0.7340766    4.3367940
C    -1.4676057   -0.5315029    2.2360618
C    -1.6583114    1.7225253    1.6187454
C    -3.0252121    1.7354861    1.9244400
C    -3.6228405    0.5642922    2.4176952
C    -2.8313094   -0.5847469    2.5738154
C    -0.5484364   -1.6989853    2.3623694
C     0.1181285   -2.2516676    1.2147237
C     0.8344046   -3.4674634    1.3995329
C     0.9270770   -4.0918218    2.6452063
C     0.2919355   -3.5247154    3.7702885
C    -0.4474334   -2.3491377    3.6201287
H    -1.1404232    2.6174085    1.2388200
H    -3.6069078    2.6586850    1.7769019
H    -4.6953745    0.5422602    2.6698833
H    -3.2598795   -1.5310727    2.9384950
H     1.3282009   -3.9147031    0.5208648
H     1.5012311   -5.0275059    2.7482383
H     0.3695262   -4.0067391    4.7581991
H    -0.9650548   -1.9088440    4.4885654
N    -0.8997333    0.6179357    1.7841440
C     0.3727864    0.2770489   -1.6717431
C     1.3747366    1.1671111   -2.1257371
C     1.3339791    1.7492282   -3.4028118
C     0.2552050    1.4697441   -4.2693719
C    -0.7677767    0.5961581   -3.8328035
C    -0.7039282    0.0129448   -2.5661651
H     2.2277463    1.3973128   -1.4688332
H     2.1476028    2.4240561   -3.7073650
O     0.1054240    1.9924912   -5.5212622
H    -1.6110245    0.3972062   -4.5133675
H    -1.5184473   -0.6533929   -2.2376011
C     0.4281146   -0.3617485   -0.3500053
C     0.0371062   -1.6531094   -0.1424265
H    -0.2044882   -2.3468740   -0.9762725
C     1.0986463    2.8748195   -6.0115048
H     0.7745559    3.1690357   -7.0304530
H     2.0975340    2.3808154   -6.0755190
H     1.1887987    3.7907179   -5.3798433
```

```
$vibrational spectrum
#  mode     symmetry     wave number   IR intensity    selection rules
#                         cm**(-1)        km/mol          IR     RAMAN
     1                        0.00         0.00000         -       -
     2                        0.00         0.00000         -       -
     3                        0.00         0.00000         -       -
```

4		0.00	0.00000	-	-
5		0.00	0.00000	-	-
6		0.00	0.00000	-	-
7	a	24.73	0.42575	YES	YES
8	a	32.77	0.77416	YES	YES
9	a	42.59	0.15089	YES	YES
10	a	46.02	0.87848	YES	YES
11	a	52.13	0.24975	YES	YES
12	a	63.43	0.74685	YES	YES
13	a	74.81	0.35220	YES	YES
14	a	85.09	0.23384	YES	YES
15	a	92.76	2.26815	YES	YES
16	a	96.37	0.91130	YES	YES
17	a	99.38	0.70388	YES	YES
18	a	107.24	0.04566	YES	YES
19	a	110.33	0.13395	YES	YES
20	a	134.31	0.77873	YES	YES
21	a	149.52	0.77319	YES	YES
22	a	156.88	0.95919	YES	YES
23	a	184.06	2.13859	YES	YES
24	a	207.19	1.35787	YES	YES
25	a	225.26	0.81501	YES	YES
26	a	245.18	1.33807	YES	YES
27	a	261.34	0.08205	YES	YES
28	a	288.42	1.41136	YES	YES
29	a	299.88	4.71848	YES	YES
30	a	309.29	1.51426	YES	YES
31	a	355.99	0.36497	YES	YES
32	a	374.05	3.39132	YES	YES
33	a	416.53	2.14670	YES	YES
34	a	420.58	1.90198	YES	YES
35	a	442.85	1.75952	YES	YES
36	a	449.83	0.82556	YES	YES
37	a	458.49	1.00003	YES	YES
38	a	460.39	0.68765	YES	YES
39	a	477.03	2.82516	YES	YES
40	a	481.12	4.21536	YES	YES
41	a	491.75	0.15798	YES	YES
42	a	505.02	7.51385	YES	YES
43	a	510.10	5.19666	YES	YES
44	a	531.44	9.00323	YES	YES
45	a	539.68	6.37763	YES	YES
46	a	543.52	12.21052	YES	YES
47	a	553.19	0.47352	YES	YES
48	a	562.70	0.53993	YES	YES
49	a	606.89	11.00156	YES	YES
50	a	622.36	39.86735	YES	YES

3be′

```
SCF Energy (au) BP86/SV(P)                      -2391.985428432
SCF Energy (au) PBE0/def2-TZVPP                 -2391.546398574
SCF Energy (au) PBE0/def2-TZVPP                 -2391.5556957408 (Toluene Correction)
Zero Point Energy (au)                          0.3230217
Chemical Potential (kJ mol-1)                   695.14
Dispersion Correction (au) PBE0/def2-TZVPP      -0.06784260

xyz coordinates
45

Mn    1.8122988    1.9480148    1.4147212
C     2.1633410    2.2412118    3.1914086
C     3.4260367    1.2198082    1.0881766
C     2.3708124    3.5311227    0.9087900
O     2.7033871    4.5923569    0.5400796
O     4.4661979    0.7486149    0.8543723
O     2.4275983    2.4515952    4.3083436
C    -0.8078624    1.2543860    1.8906140
C    -0.8508899    3.5589190    1.4669078
C    -2.2403562    3.6070803    1.6471064
C    -2.9257347    2.4251631    1.9702234
C    -2.1967927    1.2302014    2.0941101
C     0.0787136    0.0554271    2.0244011
C     0.7642242   -0.5020519    0.8946779
C     1.4890464   -1.7088776    1.0928999
C     1.5535021   -2.3326115    2.3409788
C     0.8802061   -1.7743965    3.4486059
C     0.1461699   -0.5981896    3.2855988
H    -0.2635877    4.4578431    1.2194019
H    -2.7712795    4.5655252    1.5367376
H    -4.0175570    2.4309466    2.1193480
H    -2.6923763    0.2773633    2.3364265
H     2.0070467   -2.1502731    0.2261206
H     2.1351046   -3.2617665    2.4597967
H     0.9310593   -2.2610244    4.4360588
H    -0.3910429   -0.1582303    4.1420564
N    -0.1578763    2.4098254    1.5956229
H     1.3664242    1.9902530   -1.3918890
C     1.2834493    1.4028131   -0.4572734
C     0.7459051    0.1509559   -0.4625141
C     0.2320746   -0.6098678   -1.6295954
C    -0.8102307   -2.0339223   -3.8802903
C    -1.1850094   -2.3990410   -2.5709966
C    -0.6648818   -1.6912546   -1.4726317
C     0.5997018   -0.2684207   -2.9589371
C     0.0913856   -0.9579672   -4.0595924
O    -1.2465295   -2.6482198   -5.0190950
H    -1.8836455   -3.2294423   -2.3891168
H    -0.9813916   -1.9900963   -0.4593405
H     1.3172506    0.5508679   -3.1272071
H     0.3890617   -0.6920816   -5.0863897
C    -2.1423304   -3.7371722   -4.8997078
H    -3.0985747   -3.4365554   -4.4077426
H    -1.6928093   -4.5843190   -4.3276767
H    -2.3592397   -4.0745133   -5.9335893

$vibrational spectrum
#  mode     symmetry     wave number   IR intensity    selection rules
#                         cm**(-1)        km/mol         IR     RAMAN
     1                        0.00         0.00000        -       -
     2                        0.00         0.00000        -       -
     3                        0.00         0.00000        -       -
```

4		0.00	0.00000	-	-
5		0.00	0.00000	-	-
6		0.00	0.00000	-	-
7	a	23.52	0.30109	YES	YES
8	a	30.01	0.16593	YES	YES
9	a	36.90	0.27643	YES	YES
10	a	50.51	0.06423	YES	YES
11	a	52.27	0.21524	YES	YES
12	a	67.09	1.22178	YES	YES
13	a	74.68	0.28231	YES	YES
14	a	78.64	0.28640	YES	YES
15	a	86.87	0.22244	YES	YES
16	a	92.50	0.63221	YES	YES
17	a	95.52	1.22483	YES	YES
18	a	100.13	0.02259	YES	YES
19	a	110.64	1.46995	YES	YES
20	a	134.65	0.58483	YES	YES
21	a	154.92	1.77198	YES	YES
22	a	168.01	0.43068	YES	YES
23	a	191.21	2.88992	YES	YES
24	a	204.06	0.04844	YES	YES
25	a	218.20	1.83517	YES	YES
26	a	245.89	0.16399	YES	YES
27	a	264.59	0.28702	YES	YES
28	a	281.62	1.29168	YES	YES
29	a	306.04	1.87473	YES	YES
30	a	320.05	2.46467	YES	YES
31	a	349.10	0.07857	YES	YES
32	a	357.62	0.91673	YES	YES
33	a	411.52	3.23641	YES	YES
34	a	413.58	0.11381	YES	YES
35	a	433.61	2.99339	YES	YES
36	a	453.82	0.36253	YES	YES
37	a	458.11	0.55712	YES	YES
38	a	465.67	0.55181	YES	YES
39	a	475.22	1.37219	YES	YES
40	a	489.37	4.35182	YES	YES
41	a	505.21	4.77785	YES	YES
42	a	515.59	11.10122	YES	YES
43	a	519.17	12.44789	YES	YES
44	a	530.62	5.92420	YES	YES
45	a	540.35	11.68463	YES	YES
46	a	545.26	3.48926	YES	YES
47	a	563.04	5.39888	YES	YES
48	a	606.72	24.11151	YES	YES
49	a	615.07	18.59324	YES	YES
50	a	622.05	7.04463	YES	YES

3bf

```
SCF Energy (au) BP86/SV(P)                   -2376.715791997
SCF Energy (au) PBE0/def2-TZVPP              -2376.297634233
SCF Energy (au) PBE0/def2-TZVPP              -2376.3055857295 (Toluene Correction)
Zero Point Energy (au)                       0.2836993
Chemical Potential (kJ mol-1)                598.37
Dispersion Correction (au)PBE0/def2-TZVPP    -0.06503775

xyz coordinates
41

Mn    1.2193489    0.6020264    0.8606017
C     1.6842037    0.8796677    2.6111754
C     2.8635536    0.0347297    0.3892382
C     1.6136558    2.2512375    0.4220448
O     1.8430488    3.3662344    0.1421678
O     3.9243350   -0.3226417    0.0636773
O     2.0099078    1.0716719    3.7144017
C    -1.3157925   -0.2381116    1.6367694
C    -1.5220768    2.0153745    1.0217826
C    -2.8892199    2.0182225    1.3273910
C    -3.4786214    0.8418555    1.8179138
C    -2.6790387   -0.3019325    1.9735991
C    -0.3847977   -1.3973172    1.7667139
C     0.2782322   -1.9556667    0.6205201
C     1.0105745   -3.1603577    0.8100076
C     1.1196397   -3.7711764    2.0612806
C     0.4856663   -3.2003028    3.1849117
C    -0.2675173   -2.0340653    3.0299354
H    -1.0102054    2.9143696    0.6432601
H    -3.4772754    2.9376370    1.1816374
H    -4.5511404    0.8118623    2.0692392
H    -3.1011012   -1.2513266    2.3378737
H     1.5021338   -3.6107959   -0.0682391
H     1.7046006   -4.6994786    2.1693927
H     0.5748327   -3.6726192    4.1765020
H    -0.7836713   -1.5916614    3.8981051
N    -0.7566698    0.9154196    1.1849338
C     0.4638524    0.5641322   -2.2739335
C     1.4741388    1.4300413   -2.7659224
C     1.3859130    2.0136955   -4.0373997
C     0.2610546    1.7505013   -4.8313512
C    -0.7687117    0.9135563   -4.3806572
C    -0.6592671    0.3264455   -3.1124551
H     2.3588837    1.6352477   -2.1437123
H     2.1767868    2.6771795   -4.4209382
F     0.1600023    2.3228520   -6.0494858
H    -1.6444173    0.7381501   -5.0252582
H    -1.4724362   -0.3201573   -2.7445602
C     0.5544993   -0.0746596   -0.9524162
C     0.1756649   -1.3671346   -0.7406668
H    -0.0825691   -2.0627050   -1.5680801

$vibrational spectrum
#  mode     symmetry     wave number   IR intensity    selection rules
#                         cm**(-1)        km/mol          IR     RAMAN
     1                        0.00         0.00000        -        -
     2                        0.00         0.00000        -        -
     3                        0.00         0.00000        -        -
     4                        0.00         0.00000        -        -
     5                        0.00         0.00000        -        -
     6                        0.00         0.00000        -        -
     7         a             26.77         0.20929       YES      YES
```

8	a	35.17	0.34218	YES	YES
9	a	43.76	0.07449	YES	YES
10	a	51.77	0.04781	YES	YES
11	a	56.52	0.02224	YES	YES
12	a	65.03	0.45307	YES	YES
13	a	75.62	0.26948	YES	YES
14	a	85.39	0.07668	YES	YES
15	a	94.89	1.43426	YES	YES
16	a	99.61	0.91990	YES	YES
17	a	104.50	0.18700	YES	YES
18	a	107.39	0.10932	YES	YES
19	a	139.09	0.09849	YES	YES
20	a	144.26	0.18890	YES	YES
21	a	160.21	1.62454	YES	YES
22	a	191.69	1.00490	YES	YES
23	a	214.21	1.03482	YES	YES
24	a	232.05	0.45241	YES	YES
25	a	285.99	2.11397	YES	YES
26	a	294.17	1.63832	YES	YES
27	a	303.39	2.14950	YES	YES
28	a	353.23	0.16027	YES	YES
29	a	365.44	1.32245	YES	YES
30	a	401.71	4.96093	YES	YES
31	a	416.92	0.44825	YES	YES
32	a	425.01	1.63155	YES	YES
33	a	443.83	0.90585	YES	YES
34	a	457.88	0.43720	YES	YES
35	a	459.00	0.25517	YES	YES
36	a	476.86	0.88823	YES	YES
37	a	479.54	4.53331	YES	YES
38	a	488.56	0.74087	YES	YES
39	a	503.99	5.66997	YES	YES
40	a	505.86	8.85895	YES	YES
41	a	520.33	11.79803	YES	YES
42	a	537.67	2.67685	YES	YES
43	a	541.17	10.51813	YES	YES
44	a	552.49	0.41224	YES	YES
45	a	558.84	0.56721	YES	YES
46	a	604.48	10.51765	YES	YES
47	a	622.15	39.45123	YES	YES
48	a	622.69	5.24592	YES	YES
49	a	628.44	20.32636	YES	YES
50	a	633.02	19.60220	YES	YES

3bf′

```
SCF Energy (au) BP86/SV(P)                -2376.712862759
SCF Energy (au) PBE0/def2-TZVPP           -2376.295538110
SCF Energy (au) PBE0/def2-TZVPP           -2376.3037174351 (Toluene Correction)
Zero Point Energy (au)                    0.2837056
Chemical Potential (kJ mol-1)             597.71
Dispersion Correction (au)  PBE0/def2-TZVPP -0.06440518

xyz coordinates
41

Mn    1.4841279    1.7117603    0.8161645
C     1.8347473    2.1095485    2.5732290
C     3.1379523    1.0696979    0.5072899
C     1.9390421    3.3004134    0.2278432
O     2.2002880    4.3615391   -0.1939786
O     4.2031005    0.6530320    0.2834789
O     2.0980604    2.3800517    3.6768443
C    -1.0912426    0.8955884    1.3618890
C    -1.2679949    3.1650984    0.8042767
C    -2.6571279    3.1446433    0.9918775
C    -3.2723958    1.9467289    1.3888197
C    -2.4757778    0.8045197    1.5771501
C    -0.1360325   -0.2393286    1.5618573
C     0.5855734   -0.8182385    0.4659734
C     1.3878778   -1.9607722    0.7345586
C     1.4896605   -2.5042610    2.0172579
C     0.7777799   -1.9268438    3.0901668
C    -0.0303131   -0.8122031    2.8587612
H    -0.7343996    4.0804218    0.5014531
H    -3.2424425    4.0630071    0.8290436
H    -4.3620244    1.8993039    1.5462304
H    -2.9154253   -0.1586595    1.8792875
H     1.9362159   -2.4188131   -0.1045801
H     2.1299847   -3.3849637    2.1900484
H     0.8566168   -2.3503587    4.1044705
H    -0.5971524   -0.3585124    3.6884911
N    -0.5095354    2.0666317    0.9942040
H     1.0145027    1.5569886   -1.9830248
C     0.9763957    1.0270270   -1.0116645
C     0.5225045   -0.2547710   -0.9302930
C     0.0619967   -1.1225549   -2.0449167
C    -0.8527700   -2.7478115   -4.1697179
C    -1.2287016   -3.0580846   -2.8570813
C    -0.7701515   -2.2457407   -1.8084125
C     0.4233886   -0.8492925   -3.3891059
C    -0.0298422   -1.6463722   -4.4476822
F    -1.2820083   -3.5260240   -5.1857821
H    -1.8773332   -3.9283335   -2.6700729
H    -1.0765659   -2.4870338   -0.7772746
H     1.0912536   -0.0008119   -3.6073525
H     0.2581675   -1.4362164   -5.4897271

$vibrational spectrum
#  mode     symmetry     wave number   IR intensity    selection rules
#                         cm**(-1)        km/mol          IR     RAMAN
     1                       0.00         0.00000         -        -
     2                       0.00         0.00000         -        -
     3                       0.00         0.00000         -        -
     4                       0.00         0.00000         -        -
     5                       0.00         0.00000         -        -
     6                       0.00         0.00000         -        -
     7        a             24.62         0.36777        YES      YES
```

8	a	34.24	0.13052	YES	YES
9	a	40.74	0.50128	YES	YES
10	a	52.69	0.03101	YES	YES
11	a	53.24	0.17478	YES	YES
12	a	71.92	1.11648	YES	YES
13	a	76.77	0.25782	YES	YES
14	a	83.21	0.23343	YES	YES
15	a	89.53	0.46304	YES	YES
16	a	93.28	0.18580	YES	YES
17	a	97.80	0.77714	YES	YES
18	a	103.60	0.00812	YES	YES
19	a	137.41	1.20567	YES	YES
20	a	153.58	0.57525	YES	YES
21	a	171.04	1.05843	YES	YES
22	a	203.77	0.97908	YES	YES
23	a	207.67	0.08842	YES	YES
24	a	243.63	0.46889	YES	YES
25	a	260.86	0.45042	YES	YES
26	a	303.88	1.62775	YES	YES
27	a	320.42	1.57653	YES	YES
28	a	340.64	0.35798	YES	YES
29	a	357.53	0.88841	YES	YES
30	a	395.13	2.68512	YES	YES
31	a	412.39	0.02398	YES	YES
32	a	424.94	0.63912	YES	YES
33	a	435.67	3.16904	YES	YES
34	a	457.09	0.86556	YES	YES
35	a	462.47	0.17062	YES	YES
36	a	475.11	1.19513	YES	YES
37	a	487.90	3.87155	YES	YES
38	a	496.79	17.14149	YES	YES
39	a	503.35	5.61634	YES	YES
40	a	517.87	11.40446	YES	YES
41	a	525.85	8.38907	YES	YES
42	a	536.67	2.63981	YES	YES
43	a	544.98	3.90885	YES	YES
44	a	563.85	6.90296	YES	YES
45	a	605.97	20.59872	YES	YES
46	a	615.23	21.57449	YES	YES
47	a	622.45	6.64940	YES	YES
48	a	624.34	31.15951	YES	YES
49	a	631.27	3.33761	YES	YES
50	a	635.77	17.39122	YES	YES

3bg

```
SCF Energy (au) BP86/SV(P)                  -2614.359816251
SCF Energy (au) PBE0/def2-TZVPP             -2613.984795259
SCF Energy (au) PBE0/def2-TZVPP             -2613.9929371345 (Toluene Correction)
Zero Point Energy (au)                      0.2962443
Chemical Potential (kJ mol-1)               619.21
Dispersion Correction (au) PBE0/def2-TZVPP -0.06852295

xyz coordinates
44

Mn    1.1861921    0.4233087    1.3165635
C     1.6496781    0.7318831    3.0615198
C     2.8324442   -0.1503251    0.8592402
C     1.5764145    2.0672875    0.8494133
O     1.8015756    3.1767450    0.5480063
O     3.8942168   -0.5116914    0.5419882
O     1.9743651    0.9448360    4.1608879
C    -1.3359910   -0.4259872    2.0901301
C    -1.5663266    1.8264098    1.4793153
C    -2.9334150    1.8137320    1.7861338
C    -3.5094618    0.6301670    2.2749196
C    -2.6977821   -0.5057507    2.4280755
C    -0.3888597   -1.5738836    2.2204790
C     0.2601682   -2.1423136    1.0719082
C     1.0035837   -3.3393906    1.2618963
C     1.1376889   -3.9335189    2.5188968
C     0.5185693   -3.3527583    3.6456958
C    -0.2463025   -2.1944069    3.4895463
H    -1.0640941    2.7313999    1.1019798
H    -3.5317034    2.7268438    1.6427157
H    -4.5812509    0.5884699    2.5276280
H    -3.1091275   -1.4598707    2.7922637
H     1.4842035   -3.7967404    0.3812146
H     1.7309746   -4.8562239    2.6289410
H     0.6280887   -3.8119997    4.6413336
H    -0.7523739   -1.7459878    4.3605277
N    -0.7901591    0.7339893    1.6397458
C     0.4502444    0.3771908   -1.8134071
C     1.5031279    1.1820141   -2.3181999
C     1.4211083    1.7775904   -3.5819796
C     0.2699348    1.6042905   -4.3750452
C    -0.7943677    0.8213249   -3.8886097
C    -0.7019962    0.2157855   -2.6292994
H     2.4086376    1.3296299   -1.7098435
H     2.2543302    2.3941246   -3.9533462
C     0.1970226    2.2168518   -5.7520544
H    -1.7037872    0.6935105   -4.4967259
H    -1.5460078   -0.3813972   -2.2477264
C     0.5271147   -0.2669150   -0.4950082
C     0.1438119   -1.5578492   -0.2905013
H    -0.1236005   -2.2496742   -1.1179296
F     0.9269758    3.3565088   -5.8407486
F    -1.0778491    2.5221585   -6.1055511
F     0.6739844    1.3706320   -6.7049893

$vibrational spectrum
#  mode     symmetry     wave number   IR intensity    selection rules
#                         cm**(-1)        km/mol          IR     RAMAN
     1                       0.00        0.00000          -        -
     2                       0.00        0.00000          -        -
     3                       0.00        0.00000          -        -
     4                       0.00        0.00000          -        -
```

5		0.00	0.00000	-	-
6		0.00	0.00000	-	-
7	a	21.16	0.19647	YES	YES
8	a	30.62	0.37460	YES	YES
9	a	34.38	0.08761	YES	YES
10	a	43.31	0.02830	YES	YES
11	a	45.44	0.00144	YES	YES
12	a	52.76	0.09202	YES	YES
13	a	61.15	0.48361	YES	YES
14	a	76.48	0.23164	YES	YES
15	a	85.17	0.06903	YES	YES
16	a	89.14	0.08270	YES	YES
17	a	94.28	1.18713	YES	YES
18	a	97.77	0.86356	YES	YES
19	a	105.08	0.11509	YES	YES
20	a	115.00	0.60942	YES	YES
21	a	124.46	0.08990	YES	YES
22	a	142.91	1.09096	YES	YES
23	a	165.52	0.91736	YES	YES
24	a	193.41	1.03521	YES	YES
25	a	206.99	1.04327	YES	YES
26	a	209.37	0.23505	YES	YES
27	a	246.59	0.58105	YES	YES
28	a	289.94	0.90528	YES	YES
29	a	302.57	1.09223	YES	YES
30	a	311.33	4.35150	YES	YES
31	a	353.49	1.33456	YES	YES
32	a	365.35	0.63782	YES	YES
33	a	388.01	1.67649	YES	YES
34	a	403.34	2.38918	YES	YES
35	a	406.95	2.43588	YES	YES
36	a	423.41	3.80656	YES	YES
37	a	443.59	2.53152	YES	YES
38	a	455.39	0.11763	YES	YES
39	a	459.59	0.25725	YES	YES
40	a	476.40	0.49494	YES	YES
41	a	480.99	4.10822	YES	YES
42	a	492.52	2.78660	YES	YES
43	a	505.99	5.85205	YES	YES
44	a	506.58	9.33807	YES	YES
45	a	528.92	2.64916	YES	YES
46	a	535.96	1.06907	YES	YES
47	a	549.17	2.80493	YES	YES
48	a	552.38	1.11732	YES	YES
49	a	568.97	0.13806	YES	YES
50	a	576.26	6.76105	YES	YES

3bg'

```
SCF Energy (au) BP86/SV(P)                   -2614.357206437
SCF Energy (au) PBE0/def2-TZVPP              -2613.982644347
SCF Energy (au) PBE0/def2-TZVPP              -2613.9909778406 (Toluene Correction)
Zero Point Energy (au)                       0.2962383
Chemical Potential (kJ mol-1)                618.34
Dispersion Correction (au) PBE0/def2-TZVPP -0.06781524

xyz coordinates
44

Mn    1.6001682    1.9660958    1.2084954
C     1.9402133    2.3601237    2.9702089
C     3.2552590    1.3172544    0.9168275
C     2.0678143    3.5539099    0.6260754
O     2.3383040    4.6136584    0.2077755
O     4.3208450    0.8957304    0.7063910
O     2.1966241    2.6283642    4.0754364
C    -0.9835461    1.1633802    1.7461958
C    -1.1452378    3.4336904    1.1871035
C    -2.5350874    3.4209000    1.3699921
C    -3.1584688    2.2264742    1.7648863
C    -2.3692137    1.0798174    1.9566628
C    -0.0337177    0.0245597    1.9488109
C     0.6938366   -0.5506656    0.8554690
C     1.5003815   -1.6897805    1.1248842
C     1.5970839   -2.2362874    2.4068790
C     0.8769778   -1.6642491    3.4769506
C     0.0669285   -0.5510369    3.2447207
H    -0.6057135    4.3459505    0.8855869
H    -3.1147598    4.3424036    1.2049079
H    -4.2489407    2.1851419    1.9179131
H    -2.8155056    0.1192388    2.2572971
H     2.0553274   -2.1439724    0.2879564
H     2.2393663   -3.1152472    2.5808171
H     0.9515830   -2.0904000    4.4904534
H    -0.5043819   -0.1005059    4.0730469
N    -0.3937996    2.3309129    1.3799587
H     1.1424550    1.8252016   -1.5900900
C     1.1010036    1.2923378   -0.6205604
C     0.6327642    0.0146024   -0.5412096
C     0.1520765   -0.8394015   -1.6544086
C    -0.8006482   -2.4659841   -3.7918210
C    -1.1247657   -2.7817152   -2.4594137
C    -0.6540416   -1.9806723   -1.4108200
C     0.4642913   -0.5384421   -3.0052720
C    -0.0045216   -1.3329374   -4.0560291
C    -1.2384704   -3.3710109   -4.9169275
H    -1.7555102   -3.6578757   -2.2413575
H    -0.9280730   -2.2405682   -0.3753446
H     1.1037146    0.3290837   -3.2325959
H     0.2560351   -1.0779295   -5.0951339
F    -0.3354706   -4.3639575   -5.1412764
F    -1.3787107   -2.6995453   -6.0876251
F    -2.4244684   -3.9766473   -4.6518171

$vibrational spectrum
#  mode     symmetry     wave number   IR intensity    selection rules
#                         cm**(-1)        km/mol         IR     RAMAN
     1                       0.00         0.00000        -        -
     2                       0.00         0.00000        -        -
     3                       0.00         0.00000        -        -
     4                       0.00         0.00000        -        -
```

5		0.00	0.00000	-	-
6		0.00	0.00000	-	-
7	a	19.68	0.52294	YES	YES
8	a	26.46	0.13913	YES	YES
9	a	32.03	0.26819	YES	YES
10	a	41.65	0.40771	YES	YES
11	a	48.83	0.05953	YES	YES
12	a	53.68	0.29348	YES	YES
13	a	63.35	1.14071	YES	YES
14	a	73.77	0.06558	YES	YES
15	a	77.85	0.22656	YES	YES
16	a	84.33	0.31054	YES	YES
17	a	91.53	0.39077	YES	YES
18	a	95.57	0.57094	YES	YES
19	a	99.41	0.23112	YES	YES
20	a	120.35	1.01401	YES	YES
21	a	132.15	0.20509	YES	YES
22	a	159.95	0.84835	YES	YES
23	a	171.94	1.33900	YES	YES
24	a	186.84	0.47245	YES	YES
25	a	195.29	0.80549	YES	YES
26	a	221.48	0.38611	YES	YES
27	a	263.02	1.98195	YES	YES
28	a	267.54	0.28038	YES	YES
29	a	301.13	0.31184	YES	YES
30	a	323.00	3.63535	YES	YES
31	a	348.55	0.80659	YES	YES
32	a	361.83	2.92247	YES	YES
33	a	382.91	0.74989	YES	YES
34	a	397.60	2.23162	YES	YES
35	a	404.48	1.12542	YES	YES
36	a	432.96	8.21703	YES	YES
37	a	436.02	5.05123	YES	YES
38	a	458.17	3.76486	YES	YES
39	a	461.98	3.13311	YES	YES
40	a	474.49	1.24921	YES	YES
41	a	482.14	4.98839	YES	YES
42	a	491.52	1.26753	YES	YES
43	a	515.90	6.81848	YES	YES
44	a	521.01	9.17935	YES	YES
45	a	531.91	0.61700	YES	YES
46	a	543.89	4.41968	YES	YES
47	a	555.20	2.97278	YES	YES
48	a	565.14	1.58730	YES	YES
49	a	577.77	1.37054	YES	YES
50	a	593.16	2.46991	YES	YES

3bh

```
SCF Energy (au) BP86/SV(P)                    -2505.273702407
SCF Energy (au) PBE0/def2-TZVPP               -2504.831435415
SCF Energy (au) PBE0/def2-TZVPP               -2504.8417586438 (Toluene Correction)
Zero Point Energy (au)                        0.3330534
Chemical Potential (kJ mol-1)                 713.51
Dispersion Correction (au) PBE0/def2-TZVPP    -0.07112017

xyz coordinates
47

Mn    1.1127105    0.1658704    1.8333390
C     1.5835667    0.4652460    3.5777114
C     2.7580751   -0.4013803    1.3658922
C     1.4987726    1.8125468    1.3726203
O     1.7223402    2.9241475    1.0778646
O     3.8197636   -0.7593539    1.0442834
O     1.9126438    0.6717683    4.6772089
C    -1.4083933   -0.6864908    2.6225665
C    -1.6393087    1.5654795    2.0113434
C    -3.0045365    1.5549963    2.3260552
C    -3.5795623    0.3725487    2.8187640
C    -2.7685323   -0.7641869    2.9680342
C    -0.4628153   -1.8353476    2.7491352
C     0.1903774   -2.3969144    1.5994388
C     0.9370427   -3.5926930    1.7871187
C     1.0689458   -4.1922977    3.0417279
C     0.4441777   -3.6187550    4.1690795
C    -0.3227804   -2.4613801    4.0156019
H    -1.1382390    2.4694458    1.6301879
H    -3.6023673    2.4687759    2.1851321
H    -4.6500949    0.3322759    3.0769977
H    -3.1792429   -1.7176700    3.3346265
H     1.4210940   -4.0452573    0.9058355
H     1.6643287   -5.1139616    3.1493900
H     0.5510143   -4.0825979    5.1629201
H    -0.8324425   -2.0179584    4.8870611
N    -0.8633106    0.4723700    2.1681784
C     0.3546108    0.1156761   -1.2967836
C     1.3937879    0.9355107   -1.8095598
C     1.3014148    1.5180366   -3.0781817
C     0.1508371    1.3199446   -3.8747206
C    -0.8979050    0.5181877   -3.3735738
C    -0.7967238   -0.0741491   -2.1113860
H     2.2977957    1.0998998   -1.2026855
H     2.1238572    2.1399783   -3.4621786
C    -0.0148038    1.9274728   -5.2272940
H    -1.7927646    0.3752723   -3.9999790
H    -1.6273941   -0.6863229   -1.7237034
C     0.4438958   -0.5211862    0.0233078
C     0.0692481   -1.8140045    0.2365671
H    -0.1943918   -2.5108049   -0.5878937
O     1.0685248    2.6674624   -5.5973594
O    -0.9976746    1.7912167   -5.9403573
C     0.9803481    3.2813351   -6.8872879
H     1.9278126    3.8392272   -7.0223537
H     0.1117819    3.9733183   -6.9386616
H     0.8645159    2.5147030   -7.6840296
```

$vibrational spectrum

# mode	symmetry	wave number cm**(-1)	IR intensity km/mol	selection rules IR	RAMAN
1		0.00	0.00000	-	-
2		0.00	0.00000	-	-
3		0.00	0.00000	-	-
4		0.00	0.00000	-	-
5		0.00	0.00000	-	-
6		0.00	0.00000	-	-
7	a	21.47	0.25461	YES	YES
8	a	29.01	0.98577	YES	YES
9	a	36.31	1.24804	YES	YES
10	a	43.19	0.04438	YES	YES
11	a	49.37	0.17824	YES	YES
12	a	60.58	0.47438	YES	YES
13	a	72.51	0.60654	YES	YES
14	a	76.70	0.17487	YES	YES
15	a	83.77	0.35492	YES	YES
16	a	85.91	0.04484	YES	YES
17	a	94.45	1.25210	YES	YES
18	a	96.61	0.86233	YES	YES
19	a	104.33	0.04465	YES	YES
20	a	110.46	0.11004	YES	YES
21	a	116.69	0.76729	YES	YES
22	a	122.66	0.49100	YES	YES
23	a	145.17	0.74573	YES	YES
24	a	155.67	0.42430	YES	YES
25	a	165.40	1.94312	YES	YES
26	a	196.82	0.30048	YES	YES
27	a	202.74	2.62503	YES	YES
28	a	231.80	0.93245	YES	YES
29	a	265.95	4.40239	YES	YES
30	a	291.06	0.35258	YES	YES
31	a	300.26	8.23611	YES	YES
32	a	319.46	10.79951	YES	YES
33	a	335.23	2.25638	YES	YES
34	a	360.78	1.49515	YES	YES
35	a	397.04	0.64580	YES	YES
36	a	410.01	0.38725	YES	YES
37	a	415.09	1.68924	YES	YES
38	a	434.16	1.71597	YES	YES
39	a	448.98	0.27168	YES	YES
40	a	458.05	0.85427	YES	YES
41	a	471.42	3.20674	YES	YES
42	a	476.46	0.29819	YES	YES
43	a	483.71	5.55441	YES	YES
44	a	493.53	4.04021	YES	YES
45	a	507.03	9.25964	YES	YES
46	a	509.77	7.06654	YES	YES
47	a	536.07	0.97439	YES	YES
48	a	541.46	5.73429	YES	YES
49	a	550.95	1.47145	YES	YES
50	a	560.15	1.29427	YES	YES
51	a	581.11	3.81664	YES	YES

3bh′

SCF Energy (au) BP86/SV(P)	-2505.271309102
SCF Energy (au) PBE0/def2-TZVPP	-2504.829599821
SCF Energy (au) PBE0/def2-TZVPP	-2504.8400771336 (Toluene Correction)
Zero Point Energy (au)	0.3330903
Chemical Potential (kJ mol^{-1})	712.91
Dispersion Correction (au) PBE0/def2-TZVPP	-0.07050338

xyz coordinates
47

Mn	1.6951515	2.2430586	1.7176277
C	2.0101655	2.6557657	3.4800857
C	3.3595998	1.6083577	1.4508410
C	2.1535237	3.8297089	1.1245673
O	2.4173128	4.8883301	0.6988351
O	4.4319548	1.1965283	1.2556827
O	2.2528970	2.9349053	4.5857533
C	-0.8911553	1.4255255	2.2317931
C	-1.0636848	3.6857983	1.6372204
C	-2.4562428	3.6626635	1.7969004
C	-3.0754853	2.4682106	2.1983456
C	-2.2793436	1.3318583	2.4201525
C	0.0665331	0.2994706	2.4640724
C	0.8181627	-0.2785311	1.3889900
C	1.6377436	-1.4017069	1.6847080
C	1.7222910	-1.9311725	2.9746488
C	0.9762750	-1.3577444	4.0261294
C	0.1542544	-0.2591693	3.7681922
H	-0.5275454	4.5985734	1.3312333
H	-3.0414274	4.5761381	1.6082466
H	-4.1680748	2.4187907	2.3328839
H	-2.7219556	0.3712217	2.7259262
H	2.2111109	-1.8581712	0.8615820
H	2.3743136	-2.7985375	3.1693147
H	1.0405027	-1.7712958	5.0455614
H	-0.4361572	0.1931152	4.5820178
N	-0.3055626	2.5929144	1.8583711
H	1.2733847	2.0659007	-1.0847791
C	1.2260194	1.5444683	-0.1094272
C	0.7639596	0.2647934	-0.0173071
C	0.2863037	-0.6035700	-1.1187124
C	-0.6827035	-2.2508569	-3.2398217
C	-0.9766216	-2.5633958	-1.8956706
C	-0.5001765	-1.7566044	-0.8571930
C	0.5785651	-0.3053822	-2.4761989
C	0.1032394	-1.1077699	-3.5164954
C	-1.2145995	-3.1489235	-4.3044051
H	-1.5907120	-3.4538243	-1.6870042
H	-0.7510834	-2.0168623	0.1842040
H	1.2080316	0.5669184	-2.7138496
H	0.3471571	-0.8613470	-4.5608105
O	-0.8560241	-2.7367563	-5.5540155
O	-1.8957086	-4.1438773	-4.1059219
C	-1.3314246	-3.5532820	-6.6293140
H	-2.4423585	-3.5923027	-6.6363219
H	-0.9454243	-4.5917947	-6.5390658
H	-0.9549812	-3.0801377	-7.5575728

$vibrational spectrum

# mode	symmetry	wave number	IR intensity	selection rules	
#		cm**(-1)	km/mol	IR	RAMAN
1		0.00	0.00000	-	-
2		0.00	0.00000	-	-
3		0.00	0.00000	-	-
4		0.00	0.00000	-	-
5		0.00	0.00000	-	-
6		0.00	0.00000	-	-
7	a	17.32	0.40677	YES	YES
8	a	30.33	0.77351	YES	YES
9	a	33.68	1.59592	YES	YES
10	a	45.15	0.07589	YES	YES
11	a	52.02	0.21572	YES	YES
12	a	61.41	0.36558	YES	YES
13	a	69.80	2.06162	YES	YES
14	a	73.43	0.09241	YES	YES
15	a	79.52	0.14497	YES	YES
16	a	84.06	0.11968	YES	YES
17	a	91.95	0.36492	YES	YES
18	a	95.46	0.71358	YES	YES
19	a	98.89	0.16745	YES	YES
20	a	112.27	0.74477	YES	YES
21	a	119.97	0.79742	YES	YES
22	a	124.94	0.35669	YES	YES
23	a	152.47	0.60793	YES	YES
24	a	161.11	2.48913	YES	YES
25	a	169.65	0.53274	YES	YES
26	a	192.71	1.55445	YES	YES
27	a	205.35	0.68783	YES	YES
28	a	252.07	1.34069	YES	YES
29	a	257.35	1.83314	YES	YES
30	a	282.23	2.78482	YES	YES
31	a	304.70	1.73210	YES	YES
32	a	315.40	14.71030	YES	YES
33	a	335.86	5.17570	YES	YES
34	a	354.76	1.22162	YES	YES
35	a	369.92	0.72028	YES	YES
36	a	408.22	0.34369	YES	YES
37	a	431.27	5.32783	YES	YES
38	a	438.07	5.17158	YES	YES
39	a	456.80	1.67779	YES	YES
40	a	461.73	5.07869	YES	YES
41	a	474.36	1.72360	YES	YES
42	a	478.04	4.53376	YES	YES
43	a	478.84	3.91006	YES	YES
44	a	492.21	2.50350	YES	YES
45	a	516.49	8.45721	YES	YES
46	a	521.47	8.82860	YES	YES
47	a	532.75	0.54265	YES	YES
48	a	544.48	4.99197	YES	YES
49	a	561.64	3.10440	YES	YES
50	a	598.51	1.83620	YES	YES

3bi

SCF Energy (au) BP86/SV(P) -2508.437513184
SCF Energy (au) PBE0/def2-TZVPP -2507.961431247
SCF Energy (au) PBE0/def2-TZVPP -2507.9706202452 (Toluene Correction)
Zero Point Energy (au) 0.3703473
Chemical Potential (kJ mol^{-1}) 811.94
Dispersion Correction (au) PBE0/def2-TZVPP -0.08161133

xyz coordinates
51

Mn	1.3161635	1.1650105	1.4833797
C	1.6593210	1.4842760	3.2533358
C	2.9754658	0.5498513	1.1511691
C	1.7828870	2.7957704	1.0178426
O	2.0615356	3.8928395	0.7197043
O	4.0508324	0.1662239	0.9123884
O	1.9147016	1.7039945	4.3703257
C	-1.2654764	0.4009478	2.0629745
C	-1.4245780	2.6380882	1.3808562
C	-2.8135249	2.6368021	1.5736835
C	-3.4361283	1.4687205	2.0420246
C	-2.6486650	0.3311229	2.2903335
C	-0.3040279	-0.7176535	2.2948607
C	0.4669182	-1.2705477	1.2171751
C	1.3111559	-2.3784035	1.5152922
C	1.4018540	-2.9126801	2.8009692
C	0.6286248	-2.3680511	3.8499410
C	-0.2175971	-1.2889934	3.5942423
H	-0.8858776	3.5305258	1.0244945
H	-3.3930862	3.5481466	1.3592108
H	-4.5253477	1.4398180	2.2066012
H	-3.0952519	-0.6107349	2.6451183
H	1.8933520	-2.8203291	0.6906032
H	2.0741194	-3.7644823	2.9952703
H	0.6930241	-2.7894471	4.8662471
H	-0.8236213	-0.8558833	4.4069113
N	-0.6742083	1.5465764	1.6342579
C	0.7019729	1.2990344	-1.6199975
C	1.8435003	1.9545775	-2.1486610
C	1.7585213	2.7274754	-3.3162998
C	0.5255875	2.8925712	-3.9729487
C	-0.6211451	2.2676907	-3.4516204
C	-0.5348133	1.4762490	-2.2960780
H	2.8126037	1.8416152	-1.6360318
H	2.6650371	3.2152519	-3.7125717
H	0.4580007	3.5111171	-4.8832601
H	-1.5954982	2.3933875	-3.9537007
H	-1.4352166	0.9794639	-1.8993542
C	0.7706214	0.5081032	-0.3822832
C	0.3582104	-0.7845400	-0.2063863
C	-0.0842868	-1.7713981	-1.2312206
C	-0.9525822	-3.6806862	-3.1567423
C	-1.4574443	-3.7174792	-1.8456476
C	-1.0252052	-2.7783581	-0.8960580
C	0.4245111	-1.7590372	-2.5546917
C	-0.0064244	-2.6984375	-3.5026079
H	-1.2879851	-4.4186849	-3.9042361
H	-2.1964495	-4.4845197	-1.5584149
H	-1.4334390	-2.8176048	0.1279640
H	1.1795737	-1.0088331	-2.8347555
H	0.4097852	-2.6684663	-4.5236090

$vibrational spectrum

# mode	symmetry	wave number cm**(-1)	IR intensity km/mol	selection rules IR	RAMAN
1		0.00	0.00000	-	-
2		0.00	0.00000	-	-
3		0.00	0.00000	-	-
4		0.00	0.00000	-	-
5		0.00	0.00000	-	-
6		0.00	0.00000	-	-
7	a	25.86	0.09436	YES	YES
8	a	29.50	0.18490	YES	YES
9	a	40.32	0.30306	YES	YES
10	a	41.33	0.44143	YES	YES
11	a	52.94	0.08549	YES	YES
12	a	54.89	0.03200	YES	YES
13	a	59.74	0.25229	YES	YES
14	a	68.71	0.07958	YES	YES
15	a	77.02	1.14270	YES	YES
16	a	82.37	0.69631	YES	YES
17	a	86.47	0.49161	YES	YES
18	a	95.32	0.27536	YES	YES
19	a	97.69	0.57342	YES	YES
20	a	104.79	0.29736	YES	YES
21	a	108.64	0.81777	YES	YES
22	a	145.85	0.50143	YES	YES
23	a	161.14	0.79578	YES	YES
24	a	176.47	0.59376	YES	YES
25	a	190.30	0.24471	YES	YES
26	a	213.84	0.19749	YES	YES
27	a	222.98	0.33355	YES	YES
28	a	229.16	0.75418	YES	YES
29	a	250.61	0.27012	YES	YES
30	a	262.46	0.46384	YES	YES
31	a	322.56	0.72430	YES	YES
32	a	331.75	1.46288	YES	YES
33	a	362.43	0.29753	YES	YES
34	a	402.26	0.16382	YES	YES
35	a	405.84	0.12806	YES	YES
36	a	423.68	4.33802	YES	YES
37	a	440.44	1.56353	YES	YES
38	a	446.26	0.33748	YES	YES
39	a	456.75	2.33162	YES	YES
40	a	462.56	0.15073	YES	YES
41	a	478.00	0.30726	YES	YES
42	a	484.74	4.15259	YES	YES
43	a	494.31	0.50231	YES	YES
44	a	508.46	6.46553	YES	YES
45	a	517.55	3.38325	YES	YES
46	a	532.07	3.93291	YES	YES
47	a	540.44	7.63454	YES	YES
48	a	550.21	2.02811	YES	YES
49	a	571.73	3.60312	YES	YES
50	a	584.40	11.80876	YES	YES

3bj

SCF Energy (au) BP86/SV(P) -2360.901282159
SCF Energy (au) PBE0/def2-TZVPP -2360.472310505
SCF Energy (au) PBE0/def2-TZVPP -2360.4790774360 (Toluene Correction)
Zero Point Energy (au) 0.4322002
Chemical Potential (kJ mol^{-1}) 964.24
Dispersion Correction (au) PBE0/def2-TZVPP -0.07607291

xyz coordinates
55

Mn	1.1113667	1.3348724	1.5861612
C	1.4699877	1.6516653	3.3555589
C	2.8218391	0.9509411	1.1861731
C	1.3330937	3.0123303	1.1264244
O	1.4398562	4.1363383	0.8073253
O	3.9285700	0.7046753	0.9101342
O	1.7404877	1.8868693	4.4664707
C	-1.3153556	0.1806963	2.1987127
C	-1.8014961	2.4149834	1.6870746
C	-3.1674101	2.2149889	1.9303737
C	-3.6086731	0.9444533	2.3338820
C	-2.6660703	-0.0889149	2.4701893
C	-0.2103162	-0.8145822	2.3344299
C	0.5835278	-1.2038861	1.2030727
C	1.5696859	-2.2093300	1.4160756
C	1.7680680	-2.8008236	2.6650358
C	0.9733459	-2.4150911	3.7657916
C	-0.0059727	-1.4356985	3.5963081
H	-1.4013997	3.3956176	1.3832849
H	-3.8705058	3.0535141	1.8076860
H	-4.6756997	0.7575646	2.5365705
H	-2.9662670	-1.1038212	2.7735359
H	2.1907749	-2.5199581	0.5601292
H	2.5487827	-3.5692345	2.7908357
H	1.1263528	-2.8784258	4.7539492
H	-0.6310610	-1.1247324	4.4495925
N	-0.9007362	1.4207614	1.8281105
C	0.5340973	1.4830152	-1.5717078
C	0.6101608	0.6849800	-0.2971277
C	0.3643061	-0.6474948	-0.1797845
C	-0.0538543	-1.6305026	-1.2656334
C	1.9156541	1.8340703	-2.1693745
H	-0.0592331	0.9432910	-2.3499352
H	-0.0129976	2.4375429	-1.3802344
H	0.6437644	-2.5030730	-1.2694781
H	0.0528994	-1.1530217	-2.2640702
C	-1.4947183	-2.1614023	-1.1072581
C	-1.8910644	-3.1959064	-2.1738244
H	-2.2024189	-1.2998908	-1.1401389
H	-1.6118991	-2.6166206	-0.0945834
H	-1.1802749	-4.0550810	-2.1326536
H	-1.7624382	-2.7453962	-3.1863849
C	-3.3279942	-3.7127451	-2.0228591
H	-3.5784653	-4.4590346	-2.8089909
H	-4.0672098	-2.8831574	-2.1005283
H	-3.4804237	-4.2028354	-1.0343510
H	2.5166168	2.3866163	-1.4115243
C	1.8301680	2.6701349	-3.4563536
H	2.4734648	0.8892167	-2.3710670
C	3.2025361	3.0100677	-4.0528118
H	1.2735884	3.6134453	-3.2424775
H	1.2206595	2.1220902	-4.2137046

```
H     3.8228817    3.5909238   -3.3334414
H     3.1062444    3.6169160   -4.9805035
H     3.7711742    2.0880782   -4.3120859

$vibrational spectrum
#  mode     symmetry     wave number   IR intensity    selection rules
#                         cm**(-1)        km/mol         IR     RAMAN
     1                        0.00         0.00000        -       -
     2                        0.00         0.00000        -       -
     3                        0.00         0.00000        -       -
     4                        0.00         0.00000        -       -
     5                        0.00         0.00000        -       -
     6                        0.00         0.00000        -       -
     7         a             13.34         0.11283       YES     YES
     8         a             25.09         0.03129       YES     YES
     9         a             28.09         0.18786       YES     YES
    10         a             37.85         0.64049       YES     YES
    11         a             45.03         0.03562       YES     YES
    12         a             53.49         0.24346       YES     YES
    13         a             58.99         0.36465       YES     YES
    14         a             64.03         0.04008       YES     YES
    15         a             68.06         0.24588       YES     YES
    16         a             79.99         0.05926       YES     YES
    17         a             84.38         0.19486       YES     YES
    18         a             92.13         0.18692       YES     YES
    19         a             94.47         0.83761       YES     YES
    20         a             99.23         0.48696       YES     YES
    21         a            101.84         0.42884       YES     YES
    22         a            111.75         0.05655       YES     YES
    23         a            118.00         0.13203       YES     YES
    24         a            125.34         0.16412       YES     YES
    25         a            135.14         0.13516       YES     YES
    26         a            170.14         1.02048       YES     YES
    27         a            182.18         0.33801       YES     YES
    28         a            193.07         0.50594       YES     YES
    29         a            213.09         0.22804       YES     YES
    30         a            224.04         0.17526       YES     YES
    31         a            241.41         0.41886       YES     YES
    32         a            247.48         0.21790       YES     YES
    33         a            257.31         0.63362       YES     YES
    34         a            287.43         0.63535       YES     YES
    35         a            300.51         0.52641       YES     YES
    36         a            317.16         1.48427       YES     YES
    37         a            343.56         0.72950       YES     YES
    38         a            357.13         0.08179       YES     YES
    39         a            385.22         0.44102       YES     YES
    40         a            417.12         1.89289       YES     YES
    41         a            428.70         4.60147       YES     YES
    42         a            458.93         0.22510       YES     YES
    43         a            459.65         0.88291       YES     YES
    44         a            468.59         4.26916       YES     YES
    45         a            474.45         0.45687       YES     YES
    46         a            484.48         1.64184       YES     YES
    47         a            495.02         5.00263       YES     YES
    48         a            510.81         8.03576       YES     YES
    49         a            527.80         4.57164       YES     YES
    50         a            536.42         0.97409       YES     YES
```

3ca

SCF Energy (au) BP86/SV(P)	-2678.222750796
SCF Energy (au) PBE0/def2-TZVPP	-2677.741199140
SCF Energy (au) PBE0/def2-TZVPP	-2677.7536261288 (Toluene Correction)
Zero Point Energy (au)	0.4177735
Chemical Potential (kJ mol^{-1})	922.06
Dispersion Correction (au) PBE0/def2-TZVPP	-0.08529995

xyz coordinates
57

Mn	0.0636696	1.6111328	-0.8491924
C	1.5305880	2.1895400	0.0801886
C	1.0436225	0.5324623	-1.9059389
C	0.1688237	2.9320425	-2.0074271
O	0.2269811	3.8343760	-2.7520615
O	1.6792676	-0.1601746	-2.5968114
O	2.4830343	2.5639964	0.6417431
C	-1.4234957	1.7436940	1.4825916
C	-1.9635197	3.6789869	0.2797856
C	-2.8252705	4.1154596	1.2951276
C	-2.9710718	3.3305480	2.4511613
C	-2.2573617	2.1245152	2.5466824
C	-0.6267010	0.4849612	1.4533082
C	-0.8050618	-0.4633337	0.4197156
C	-0.1193440	-1.7697392	0.5270746
N	0.7698340	-1.9465157	1.6357918
C	0.9516228	-0.9885818	2.6165126
C	0.2530664	0.2037401	2.5436844
H	-1.8053058	4.2625049	-0.6407915
H	-3.3701369	5.0646763	1.1751830
H	-3.6377278	3.6509903	3.2679992
H	-2.3495205	1.4696870	3.4267453
O	-0.2933570	-2.6860680	-0.2787171
C	1.9011784	-1.2733206	3.7489853
H	0.4115791	0.9607065	3.3249413
N	-1.2724795	2.5243810	0.3818916
C	-2.5266545	0.9478085	-2.6966211
C	-2.0482630	1.4007388	-3.9528735
C	-2.9215011	1.6046010	-5.0314184
C	-4.3021051	1.3820062	-4.8814423
C	-4.7995416	0.9453821	-3.6404013
C	-3.9253567	0.7288513	-2.5653109
H	-0.9691196	1.5761126	-4.0850712
H	-2.5192581	1.9452965	-6.0002335
H	-4.9887656	1.5536703	-5.7270793
H	-5.8814529	0.7740495	-3.5079285
H	-4.3226241	0.3985879	-1.5910971
C	-1.6271725	0.7047443	-1.5632998
C	-1.7629983	-0.3443478	-0.7046295
H	-2.4346265	-1.2097105	-0.8829210
H	1.9823365	-0.3844132	4.4063731
H	2.9174770	-1.5269463	3.3760077
H	1.5564095	-2.1285800	4.3740081
C	1.4697500	-3.2492764	1.6847932
H	1.4258115	-3.6430046	2.7199194
C	2.9070780	-3.2156046	1.1879399
H	0.8632975	-3.9110563	1.0317846
C	5.5741048	-3.2363148	0.2415008
C	5.2677551	-3.7893264	1.4956591
C	3.9427165	-3.7747900	1.9648590
C	3.2231298	-2.6724219	-0.0763338
C	4.5476388	-2.6785393	-0.5415754

```
H     6.6129593   -3.2415146   -0.1280164
H     6.0638343   -4.2325353    2.1167661
H     3.7094368   -4.2123280    2.9517072
H     2.4268794   -2.2536220   -0.7126693
H     4.7759111   -2.2481847   -1.5305696
```

```
$vibrational spectrum
#  mode     symmetry     wave number   IR intensity    selection rules
#                         cm**(-1)        km/mol         IR     RAMAN
     1                        0.00         0.00000         -       -
     2                        0.00         0.00000         -       -
     3                        0.00         0.00000         -       -
     4                        0.00         0.00000         -       -
     5                        0.00         0.00000         -       -
     6                        0.00         0.00000         -       -
     7         a             18.61         0.45792        YES     YES
     8         a             20.78         0.19310        YES     YES
     9         a             29.85         0.31391        YES     YES
    10         a             32.25         0.44016        YES     YES
    11         a             39.02         0.86850        YES     YES
    12         a             45.99         0.33234        YES     YES
    13         a             50.47         0.19399        YES     YES
    14         a             56.23         1.74737        YES     YES
    15         a             68.28         1.39703        YES     YES
    16         a             69.73         0.36509        YES     YES
    17         a             84.03         1.01838        YES     YES
    18         a             85.80         0.48941        YES     YES
    19         a             96.54         1.61114        YES     YES
    20         a             98.74         0.20719        YES     YES
    21         a            102.86         0.31583        YES     YES
    22         a            107.00         0.56136        YES     YES
    23         a            138.11         0.28842        YES     YES
    24         a            144.37         0.21805        YES     YES
    25         a            157.74         0.97101        YES     YES
    26         a            163.60         1.04911        YES     YES
    27         a            185.47         0.24484        YES     YES
    28         a            201.82         1.54103        YES     YES
    29         a            207.76         1.05400        YES     YES
    30         a            233.47         0.65986        YES     YES
    31         a            245.35         1.21255        YES     YES
    32         a            258.57         1.52062        YES     YES
    33         a            272.27         1.59868        YES     YES
    34         a            289.99         0.13259        YES     YES
    35         a            311.62         4.20087        YES     YES
    36         a            329.43         1.98900        YES     YES
    37         a            362.92         2.40152        YES     YES
    38         a            377.48         2.46814        YES     YES
    39         a            395.04        12.41401        YES     YES
    40         a            403.33         0.34388        YES     YES
    41         a            407.17         0.15896        YES     YES
    42         a            426.10         2.97114        YES     YES
    43         a            440.77         1.24828        YES     YES
    44         a            452.05         1.61406        YES     YES
    45         a            459.30         0.36019        YES     YES
    46         a            463.85         1.07994        YES     YES
    47         a            479.01         2.34965        YES     YES
    48         a            485.60         1.09251        YES     YES
    49         a            490.13         4.05355        YES     YES
    50         a            497.90         6.21632        YES     YES
```

3ca′

```
SCF Energy (au) BP86/SV(P)                    -2678.218280289
SCF Energy (au) PBE0/def2-TZVPP               -2677.737373921
SCF Energy (au) PBE0/def2-TZVPP               -2677.7503837273 (Toluene Correction)
Zero Point Energy (au)                        0.4178331
Chemical Potential (kJ mol-1)                 923.56
Dispersion Correction (au) PBE0/def2-TZVPP  -0.08515989

xyz coordinates
57

Mn    0.2297979    2.1745138   -1.3347197
C     1.6171610    2.8337007   -0.3315315
C     1.2911652    1.0907227   -2.2971892
C     0.3343992    3.4203114   -2.5789391
O     0.3609910    4.2469473   -3.4069981
O     1.9707290    0.3987678   -2.9452103
O     2.5256098    3.2620245    0.2634741
C    -1.4768114    2.3038411    0.8307032
C    -1.8780000    4.2761384   -0.3609654
C    -2.8525899    4.6790307    0.5633144
C    -3.1316419    3.8533537    1.6646649
C    -2.4288200    2.6441698    1.8034043
C    -0.6369008    1.0678879    0.8411725
C    -0.7131995    0.1374552   -0.2162608
C     0.1030264   -1.0946837   -0.1478118
N     0.9108383   -1.2652747    1.0291886
C     0.9564707   -0.3417857    2.0553849
C     0.1983591    0.8129073    1.9730585
H    -1.6156147    4.8907832   -1.2371658
H    -3.3824773    5.6328345    0.4151367
H    -3.8916590    4.1451546    2.4072869
H    -2.6179118    1.9615775    2.6460739
O     0.1013544   -1.9604783   -1.0212301
C     1.8320981   -0.6166457    3.2481219
H     0.2658504    1.5549755    2.7813309
N    -1.2054328    3.1165711   -0.2206989
H    -1.9357513    1.5131526   -3.1078177
C    -1.3585129    1.2726871   -2.1953513
C    -1.6781994    0.2414852   -1.3724655
C    -2.7915273   -0.7316675   -1.4904575
H     1.8106211    0.2490556    3.9400695
H     2.8864511   -0.8003277    2.9471307
H     1.4896528   -1.5130903    3.8135666
C     1.6729266   -2.5335254    1.0922998
H     1.5523006   -2.9724171    2.1030852
C     3.1473011   -2.4144786    0.7377724
H     1.1659724   -3.1938085    0.3581996
C     5.8898231   -2.2723622    0.0562233
C     5.4959697   -2.8570271    1.2710702
C     4.1330585   -2.9234494    1.6086874
C     3.5518096   -1.8408847   -0.4872837
C     4.9135787   -1.7658983   -0.8204193
H     6.9582646   -2.2136907   -0.2098935
H     6.2524496   -3.2618886    1.9637387
H     3.8306678   -3.3859600    2.5648116
H     2.7967394   -1.4638642   -1.1954814
H     5.2116198   -1.3127252   -1.7801833
C    -4.9711082   -2.5509902   -1.6925383
C    -4.3148150   -2.3649062   -0.4643236
C    -3.2400184   -1.4672536   -0.3665762
C    -3.4592078   -0.9391965   -2.7228957
C    -4.5351303   -1.8314917   -2.8212454
```

```
H    -5.8131660   -3.2582268   -1.7737531
H    -4.6427955   -2.9243449    0.4282348
H    -2.7419062   -1.3250928    0.6078718
H    -3.1110665   -0.4038709   -3.6210112
H    -5.0327927   -1.9787415   -3.7946602

$vibrational spectrum
#  mode     symmetry     wave number   IR intensity    selection rules
#                         cm**(-1)        km/mol          IR     RAMAN
     1                        0.00         0.00000         -       -
     2                        0.00         0.00000         -       -
     3                        0.00         0.00000         -       -
     4                        0.00         0.00000         -       -
     5                        0.00         0.00000         -       -
     6                        0.00         0.00000         -       -
     7         a             19.21         0.16432        YES     YES
     8         a             23.47         0.45449        YES     YES
     9         a             29.88         0.64780        YES     YES
    10         a             39.48         0.08769        YES     YES
    11         a             42.64         0.43937        YES     YES
    12         a             44.77         2.00535        YES     YES
    13         a             49.07         0.67123        YES     YES
    14         a             55.87         0.47658        YES     YES
    15         a             69.43         1.32303        YES     YES
    16         a             73.15         0.50441        YES     YES
    17         a             81.07         0.52119        YES     YES
    18         a             90.08         0.21952        YES     YES
    19         a             94.00         0.36477        YES     YES
    20         a             97.20         1.86791        YES     YES
    21         a            101.54         0.45680        YES     YES
    22         a            109.42         0.68332        YES     YES
    23         a            137.50         0.11320        YES     YES
    24         a            144.65         2.13129        YES     YES
    25         a            160.28         0.50306        YES     YES
    26         a            181.46         0.53773        YES     YES
    27         a            191.63         0.15253        YES     YES
    28         a            197.91         1.11741        YES     YES
    29         a            224.72         1.58425        YES     YES
    30         a            233.22         0.59602        YES     YES
    31         a            240.86         0.33051        YES     YES
    32         a            257.01         0.35306        YES     YES
    33         a            262.34         1.02785        YES     YES
    34         a            295.93         0.90626        YES     YES
    35         a            316.02         0.73711        YES     YES
    36         a            319.52         4.67888        YES     YES
    37         a            361.51         1.02386        YES     YES
    38         a            378.85         1.70818        YES     YES
    39         a            403.51         0.14089        YES     YES
    40         a            404.39         0.54864        YES     YES
    41         a            422.33         1.59328        YES     YES
    42         a            428.18         1.08852        YES     YES
    43         a            434.15         6.12589        YES     YES
    44         a            455.16         4.69089        YES     YES
    45         a            463.52         0.55268        YES     YES
    46         a            466.18         0.63456        YES     YES
    47         a            477.75         2.37488        YES     YES
    48         a            489.32         1.31633        YES     YES
    49         a            491.10         3.15360        YES     YES
    50         a            507.95         6.32814        YES     YES
```

3da

```
SCF Energy (au) BP86/SV(P)                    -2409.037058188
SCF Energy (au) PBE0/def2-TZVPP               -2408.578577113
SCF Energy (au) PBE0/def2-TZVPP               -2408.5876200366 (Toluene Correction)
Zero Point Energy (au)                        0.3203729
Chemical Potential (kJ mol-1)                 693.96
Dispersion Correction (au) PBE0/def2-TZVPP -0.06859816

xyz coordinates
45

Mn    1.1173719    1.0312839    0.3566833
C     1.5853652    1.5750691    2.0338526
C     2.7562093    0.3798074   -0.0005427
C     1.4880160    2.6264045   -0.3098843
O     1.6808120    3.7068467   -0.7146336
O     3.8142952   -0.0423134   -0.2474111
O     1.9234554    1.9382031    3.0908395
C    -1.2016639    0.0794117    1.0776690
C    -1.8119419    2.2544276    0.5093869
C    -3.1491854    1.9891519    0.8413571
C    -3.4998775    0.7133415    1.3151695
C    -2.5040187   -0.2736911    1.4376583
N    -0.0460866   -0.7976603    1.1110116
C     0.0622448   -1.7181936   -0.0254837
C     0.2544036   -2.9977070    0.4507647
C     0.3053010   -2.9554018    1.8977565
C     0.1182089   -1.6011429    2.2925356
H    -1.4764482    3.2355907    0.1375581
H    -3.9049751    2.7820805    0.7301525
H    -4.5432122    0.4829444    1.5843137
H    -2.7234604   -1.2890434    1.8000928
H     0.4354268   -3.8781585   -0.1795344
C     0.0947015   -1.1989255    3.6301130
N    -0.8640865    1.3059838    0.6397878
C     0.5895501    0.8093864   -2.7920829
C     1.7257682    1.5142081   -3.2664243
C     1.7502745    2.0697311   -4.5537556
C     0.6308816    1.9615782   -5.3988076
C    -0.5109802    1.2790708   -4.9444412
C    -0.5287534    0.7059350   -3.6634394
H     2.6131224    1.6035099   -2.6204160
H     2.6539177    2.5990724   -4.8995892
H     0.6476101    2.4121817   -6.4050893
H    -1.3988180    1.1925165   -5.5935540
H    -1.4314143    0.1808745   -3.3093448
C     0.5521816    0.1851989   -1.4555397
C     0.0988969   -1.1088118   -1.3318299
H    -0.1416297   -1.7453257   -2.2054555
C     0.2843591   -2.1930636    4.6094536
H    -0.0474494   -0.1445334    3.9121403
H     0.2843663   -1.9096302    5.6742443
C     0.4924938   -3.5405921    4.2422694
H     0.6490521   -4.2965513    5.0294823
C     0.5065170   -3.9343051    2.8944337
H     0.6691984   -4.9887601    2.6185332

$vibrational spectrum
#  mode     symmetry     wave number   IR intensity    selection rules
#                         cm**(-1)        km/mol          IR     RAMAN
     1                        0.00         0.00000        -       -
     2                        0.00         0.00000        -       -
     3                        0.00         0.00000        -       -
```

4		0.00	0.00000	-	-
5		0.00	0.00000	-	-
6		0.00	0.00000	-	-
7	a	28.92	0.40565	YES	YES
8	a	37.26	0.06249	YES	YES
9	a	46.50	0.25372	YES	YES
10	a	54.32	0.01770	YES	YES
11	a	60.74	0.30107	YES	YES
12	a	69.85	1.24702	YES	YES
13	a	74.70	0.45702	YES	YES
14	a	81.89	0.81541	YES	YES
15	a	84.73	0.24313	YES	YES
16	a	95.20	0.25017	YES	YES
17	a	98.42	0.55231	YES	YES
18	a	109.24	0.18919	YES	YES
19	a	141.07	0.11060	YES	YES
20	a	152.17	1.49613	YES	YES
21	a	180.10	1.27728	YES	YES
22	a	189.89	0.05206	YES	YES
23	a	208.92	0.97205	YES	YES
24	a	223.73	0.79529	YES	YES
25	a	261.43	0.47915	YES	YES
26	a	268.90	2.74244	YES	YES
27	a	286.18	0.52435	YES	YES
28	a	325.79	2.71826	YES	YES
29	a	363.13	0.70236	YES	YES
30	a	396.44	2.80952	YES	YES
31	a	406.95	1.05324	YES	YES
32	a	423.82	1.01802	YES	YES
33	a	435.22	11.95528	YES	YES
34	a	448.51	2.81685	YES	YES
35	a	459.52	2.65076	YES	YES
36	a	471.73	0.78697	YES	YES
37	a	481.06	0.16180	YES	YES
38	a	490.42	1.12418	YES	YES
39	a	493.26	0.25020	YES	YES
40	a	507.66	6.69919	YES	YES
41	a	517.95	6.01909	YES	YES
42	a	523.84	3.06030	YES	YES
43	a	538.84	2.98628	YES	YES
44	a	557.16	6.48803	YES	YES
45	a	576.80	1.67949	YES	YES
46	a	590.09	2.39371	YES	YES
47	a	596.12	0.77841	YES	YES
48	a	613.34	0.49274	YES	YES
49	a	615.28	7.44090	YES	YES
50	a	629.75	37.33445	YES	YES

3da′

SCF Energy (au) BP86/SV(P) -2409.034407606
SCF Energy (au) PBE0/def2-TZVPP -2408.576885875
SCF Energy (au) PBE0/def2-TZVPP -2408.5859493044 (Toluene Correction)
Zero Point Energy (au) 0.3203032
Chemical Potential (kJ mol^{-1}) 693.18
Dispersion Correction (au) PBE0/def2-TZVPP -0.06741973

xyz coordinates
45

Mn	1.4340079	1.9877711	0.3062720
C	1.8611104	2.6173755	1.9720891
C	3.0597099	1.2662607	0.0417952
C	1.8808437	3.4962167	-0.4980946
O	2.1288308	4.4943936	-1.0556917
O	4.1060042	0.7903219	-0.1480240
O	2.1753840	3.0340469	3.0164158
C	-0.9286730	1.0876959	0.9320402
C	-1.4806415	3.2806788	0.3782828
C	-2.8348031	3.0320744	0.6504191
C	-3.2248963	1.7542155	1.0856839
C	-2.2501376	0.7493288	1.2310873
N	0.2117614	0.1930013	1.0060692
C	0.3495094	-0.7519616	-0.1065452
C	0.5573961	-2.0135000	0.4037625
C	0.5762752	-1.9380122	1.8512294
C	0.3552348	-0.5798653	2.2096329
H	-1.1154830	4.2629505	0.0383258
H	-3.5728545	3.8391420	0.5229589
H	-4.2819412	1.5362782	1.3074368
H	-2.4999159	-0.2679966	1.5676158
H	0.7611768	-2.9052401	-0.2026803
C	0.2927999	-0.1486692	3.5373096
N	-0.5541226	2.3152395	0.5282990
H	1.0509728	1.6100614	-2.4509835
C	0.9377638	1.1242993	-1.4614443
C	0.4414493	-0.1591618	-1.4407637
C	0.0770928	-0.9643189	-2.6393310
C	0.4769828	-1.1177742	4.5419780
H	0.1261389	0.9089574	3.7929121
H	0.4459442	-0.8111523	5.5998927
C	0.7192618	-2.4689522	4.2107376
H	0.8712784	-3.2040692	5.0182099
C	0.7725838	-2.8913423	2.8727324
H	0.9617283	-3.9479961	2.6232848
C	-0.6553254	-2.4477331	-4.9556920
C	-1.3339412	-2.6473684	-3.7412208
C	-0.9712341	-1.9166997	-2.5986553
C	0.7560605	-0.7835335	-3.8694422
C	0.3924769	-1.5111278	-5.0121335
H	-0.9365524	-3.0241700	-5.8526284
H	-2.1586505	-3.3776792	-3.6821766
H	-1.5211851	-2.0737152	-1.6559985
H	1.5978745	-0.0739001	-3.9198092
H	0.9427041	-1.3543704	-5.9551583

$vibrational spectrum

# mode	symmetry	wave number cm**(-1)	IR intensity km/mol	selection rules IR	selection rules RAMAN
1		0.00	0.00000	-	-
2		0.00	0.00000	-	-
3		0.00	0.00000	-	-

4		0.00	0.00000	-	-
5		0.00	0.00000	-	-
6		0.00	0.00000	-	-
7	a	30.85	0.31969	YES	YES
8	a	38.66	0.26769	YES	YES
9	a	44.03	0.45409	YES	YES
10	a	50.44	0.46117	YES	YES
11	a	58.79	0.13910	YES	YES
12	a	70.56	0.77444	YES	YES
13	a	73.15	0.79337	YES	YES
14	a	79.09	0.77549	YES	YES
15	a	85.25	0.31938	YES	YES
16	a	89.55	0.27598	YES	YES
17	a	96.42	0.02125	YES	YES
18	a	98.81	0.32592	YES	YES
19	a	143.17	0.26355	YES	YES
20	a	148.26	1.57227	YES	YES
21	a	186.79	0.69674	YES	YES
22	a	201.64	0.61927	YES	YES
23	a	213.25	0.94402	YES	YES
24	a	243.77	0.81043	YES	YES
25	a	254.48	0.62309	YES	YES
26	a	265.85	1.47357	YES	YES
27	a	284.54	2.00587	YES	YES
28	a	321.31	0.94779	YES	YES
29	a	355.83	1.61262	YES	YES
30	a	405.73	0.07009	YES	YES
31	a	409.56	3.84672	YES	YES
32	a	424.95	1.86710	YES	YES
33	a	428.29	0.73869	YES	YES
34	a	435.15	11.43486	YES	YES
35	a	456.30	2.74287	YES	YES
36	a	469.32	1.54325	YES	YES
37	a	479.73	0.92292	YES	YES
38	a	480.02	2.45529	YES	YES
39	a	490.24	1.33349	YES	YES
40	a	510.11	1.02306	YES	YES
41	a	517.63	12.22340	YES	YES
42	a	535.70	3.25324	YES	YES
43	a	544.60	13.91874	YES	YES
44	a	559.29	0.84540	YES	YES
45	a	586.19	12.01317	YES	YES
46	a	591.95	7.84937	YES	YES
47	a	612.80	1.80156	YES	YES
48	a	614.86	8.34012	YES	YES
49	a	623.92	17.83804	YES	YES
50	a	629.15	19.67110	YES	YES

3ea

```
SCF Energy (au) BP86/SV(P)                     -2583.785980288
SCF Energy (au) PBE0/def2-TZVPP                -2583.339566677
SCF Energy (au) PBE0/def2-TZVPP                -2583.3503113936 (Toluene Correction)
Zero Point Energy (au)                         0.3875299
Chemical Potential (kJ mol-1)                  845.24
Dispersion Correction (au) PBE0/def2-TZVPP -0.07468328
```

```
xyz coordinates
53

Mn    1.9651695    1.7154598   -0.0109753
C     2.3480792    2.1006590    1.7392816
C     3.6973241    1.7465730   -0.5034005
C     1.7741513    3.3973560   -0.4504732
O     1.6051695    4.5182711   -0.7493858
O     4.8192058    1.7633187   -0.8235393
O     2.6033358    2.3533202    2.8488849
C    -0.4596660    0.2929554    0.8343070
C    -1.8865719    0.1335204    1.1967370
C     0.5060078   -0.8137106    1.0092949
C     1.4409848   -1.1567847   -0.0335687
C     2.3040319   -2.2512157    0.2004136
C     2.3034134   -2.9730490    1.4097088
C     1.3855560   -2.6246036    2.4298631
C     0.4961805   -1.5686285    2.2040837
H     3.0127941   -2.5634002   -0.5827856
O     3.2069497   -3.9838538    1.5007735
H     1.3545968   -3.1654988    3.3862966
H    -0.2292973   -1.3047793    2.9906630
N     0.0196209    1.4114102    0.3521336
C     1.4688948    1.2072974   -3.1371296
C     2.2378999    2.2996925   -3.6143953
C     2.1892361    2.6901570   -4.9601881
C     1.3497766    2.0180364   -5.8664389
C     0.5608614    0.9467961   -5.4106367
C     0.6209091    0.5470609   -4.0678958
H     2.8950311    2.8414346   -2.9166392
H     2.8091079    3.5353018   -5.3038577
H     1.3020567    2.3353389   -6.9215125
H    -0.1133541    0.4207023   -6.1077445
H    -0.0163634   -0.2805926   -3.7138587
C     1.5237799    0.7697178   -1.7339513
C     1.4568059   -0.5578925   -1.3974224
H     1.5804506   -1.3178251   -2.1995321
C    -4.6486942   -0.1440778    1.8216597
C    -3.8957116   -1.2585711    1.3794449
C    -2.5409938   -1.1210499    1.0794103
C    -2.6500765    1.2351825    1.6485544
C    -4.0110524    1.1102307    1.9590232
O    -5.9571818   -0.3790843    2.0947564
H    -4.4092359   -2.2272115    1.2753295
H    -1.9733789   -1.9956395    0.7236929
H    -2.1637524    2.2140225    1.7981964
H    -4.5601925    1.9905313    2.3235603
H    -0.6890552    2.1269921    0.1307717
C    -6.7749949    0.6979217    2.5289010
H    -6.4171042    1.1218142    3.4963019
H    -7.7888033    0.2737619    2.6716324
H    -6.8227436    1.5107816    1.7666559
C     3.2675396   -4.7477234    2.6950863
H     2.3081708   -5.2818123    2.8928381
H     3.5230979   -4.1143063    3.5769682
```

```
H     4.0720346   -5.4943078    2.5401061

$vibrational spectrum
#  mode     symmetry     wave number   IR intensity    selection rules
#                         cm**(-1)        km/mol         IR     RAMAN
     1                        0.00         0.00000        -       -
     2                        0.00         0.00000        -       -
     3                        0.00         0.00000        -       -
     4                        0.00         0.00000        -       -
     5                        0.00         0.00000        -       -
     6                        0.00         0.00000        -       -
     7        a              14.51         0.31178       YES     YES
     8        a              20.08         0.83075       YES     YES
     9        a              25.76         0.14120       YES     YES
    10        a              37.40         0.41839       YES     YES
    11        a              41.74         0.36272       YES     YES
    12        a              49.73         0.66072       YES     YES
    13        a              55.73         0.02701       YES     YES
    14        a              59.24         0.47353       YES     YES
    15        a              72.72         1.11749       YES     YES
    16        a              82.52         2.52930       YES     YES
    17        a              85.90         0.32333       YES     YES
    18        a              88.22         0.71131       YES     YES
    19        a              96.11         0.90403       YES     YES
    20        a             103.97         1.16949       YES     YES
    21        a             114.15         0.83859       YES     YES
    22        a             128.32         0.20179       YES     YES
    23        a             146.83         0.31438       YES     YES
    24        a             151.89         1.29281       YES     YES
    25        a             168.11         4.09408       YES     YES
    26        a             179.42         1.14541       YES     YES
    27        a             200.02         0.38012       YES     YES
    28        a             207.62         1.66963       YES     YES
    29        a             226.15         1.86463       YES     YES
    30        a             235.19         0.92040       YES     YES
    31        a             239.41         4.04946       YES     YES
    32        a             255.06         2.65217       YES     YES
    33        a             269.98         4.48469       YES     YES
    34        a             295.05         0.80520       YES     YES
    35        a             302.33         8.55660       YES     YES
    36        a             321.72         4.90520       YES     YES
    37        a             332.49         0.29942       YES     YES
    38        a             369.86         2.17629       YES     YES
    39        a             406.94         0.53710       YES     YES
    40        a             414.79         0.71808       YES     YES
    41        a             423.67        13.01661       YES     YES
    42        a             438.34         2.17459       YES     YES
    43        a             443.47         3.37086       YES     YES
    44        a             459.10         0.73837       YES     YES
    45        a             466.53         1.15636       YES     YES
    46        a             479.54         5.36961       YES     YES
    47        a             486.06         4.36720       YES     YES
    48        a             487.99         0.66771       YES     YES
    49        a             508.96         4.99059       YES     YES
    50        a             517.77        16.82526       YES     YES
```

3ea′

SCF Energy (au) BP86/SV(P) -2583.783208244
SCF Energy (au) PBE0/def2-TZVPP -2583.338085995
SCF Energy (au) PBE0/def2-TZVPP -2583.3490964570 (Toluene Correction)
Zero Point Energy (au) 0.3874885
Chemical Potential (kJ mol^{-1}) 845.08
Dispersion Correction (au) PBE0/def2-TZVPP -0.07655516

xyz coordinates
53

Mn	2.2974223	2.1044248	0.4369364
C	2.5307348	2.4894570	2.2189721
C	3.9961773	1.5489878	0.2111146
C	2.6950408	3.7214756	-0.1135425
O	2.9172068	4.8004252	-0.5113261
O	5.0878474	1.1750973	0.0386081
O	2.7266135	2.7509417	3.3388771
C	-0.3367679	1.2586260	0.8293903
C	0.5650690	0.0931635	1.0974360
C	1.4190804	-0.4432591	0.0796196
C	2.2302767	-1.5631004	0.3996120
C	2.2196428	-2.1378792	1.6817671
C	1.3580732	-1.6084829	2.6786297
C	0.5452219	-0.5219353	2.3808379
H	2.8695718	-1.9689312	-0.3975123
O	2.9841743	-3.1913069	2.0626043
H	1.3581999	-2.0726174	3.6768799
H	-0.1263325	-0.1178035	3.1557622
N	0.3099283	2.3397319	0.4920302
H	2.0412466	1.8893515	-2.3888246
C	1.9315377	1.3882431	-1.4079445
C	1.4722862	0.1082100	-1.3263266
C	1.0943724	-0.7792518	-2.4587611
C	0.3152161	-2.4467361	-4.6381530
C	-0.1268456	-2.7283626	-3.3346308
C	0.2589812	-1.9081774	-2.2623096
C	1.5376021	-0.5183121	-3.7808919
C	1.1510932	-1.3346910	-4.8523306
H	0.0166338	-3.0916677	-5.4811835
H	-0.7832348	-3.5953591	-3.1483465
H	-0.1036392	-2.1414395	-1.2473626
H	2.2121393	0.3334013	-3.9649028
H	1.5166260	-1.1071508	-5.8679953
H	-0.2535891	3.1571260	0.2143462
C	-1.8001614	1.1572467	0.9782788
C	-4.6305559	0.9519304	1.2004177
C	-3.9975071	2.2132014	1.3019330
C	-2.6042064	2.3022971	1.1927338
C	-2.4532462	-0.1014638	0.8965114
C	-3.8394319	-0.2047556	0.9993735
O	-5.9688860	0.7507156	1.2899569
H	-4.5802896	3.1284535	1.4809860
H	-2.1331432	3.2929831	1.3085166
H	-1.8544808	-1.0106545	0.7282756
H	-4.3478546	-1.1784593	0.9226608
C	-6.8248518	1.8678565	1.4820771
H	-7.8555108	1.4617076	1.5106249
H	-6.7418905	2.5971653	0.6426280
H	-6.6108553	2.3890134	2.4446946
C	3.8940188	-3.7532632	1.1255572
H	4.6405768	-3.0004128	0.7809828
H	3.3628970	-4.1761989	0.2411378

```
H     4.4177724   -4.5695610    1.6615739
$vibrational spectrum
#  mode     symmetry     wave number   IR intensity    selection rules
#                         cm**(-1)        km/mol         IR     RAMAN
     1                      0.00          0.00000        -        -
     2                      0.00          0.00000        -        -
     3                      0.00          0.00000        -        -
     4                      0.00          0.00000        -        -
     5                      0.00          0.00000        -        -
     6                      0.00          0.00000        -        -
     7         a           15.63          0.19309       YES      YES
     8         a           21.60          0.77485       YES      YES
     9         a           27.86          0.18949       YES      YES
    10         a           35.39          0.38876       YES      YES
    11         a           41.33          0.19663       YES      YES
    12         a           44.02          0.29313       YES      YES
    13         a           46.80          0.16734       YES      YES
    14         a           56.38          0.54129       YES      YES
    15         a           66.90          0.05337       YES      YES
    16         a           79.52          0.12712       YES      YES
    17         a           82.08          0.21891       YES      YES
    18         a           91.71          0.53619       YES      YES
    19         a           94.79          0.47801       YES      YES
    20         a          104.40          2.24284       YES      YES
    21         a          118.46          1.63451       YES      YES
    22         a          134.87          1.59926       YES      YES
    23         a          146.93          1.29975       YES      YES
    24         a          160.54          2.27608       YES      YES
    25         a          164.22          0.42093       YES      YES
    26         a          178.65          1.05013       YES      YES
    27         a          184.01          0.32613       YES      YES
    28         a          208.70          2.55452       YES      YES
    29         a          212.55          0.03873       YES      YES
    30         a          239.72          0.04436       YES      YES
    31         a          260.67          0.38034       YES      YES
    32         a          270.38          1.24015       YES      YES
    33         a          272.85          4.07023       YES      YES
    34         a          298.06          9.95050       YES      YES
    35         a          313.32          0.41120       YES      YES
    36         a          318.07          1.89895       YES      YES
    37         a          342.89          2.41294       YES      YES
    38         a          403.82          0.05490       YES      YES
    39         a          411.48          0.65777       YES      YES
    40         a          417.18          2.58456       YES      YES
    41         a          423.43          8.11935       YES      YES
    42         a          439.84          3.47479       YES      YES
    43         a          453.26          0.80939       YES      YES
    44         a          454.51          2.07382       YES      YES
    45         a          462.67          2.46979       YES      YES
    46         a          473.90          0.79383       YES      YES
    47         a          486.97          4.80856       YES      YES
    48         a          491.01          1.88003       YES      YES
    49         a          508.87         18.13092       YES      YES
    50         a          515.17         21.25998       YES      YES
```

3ej

SCF Energy (au) BP86/SV(P) -2814.675728998
SCF Energy (au) PBE0/def2-TZVPP -2814.194226758
SCF Energy (au) PBE0/def2-TZVPP -2814.2067699821
Zero Point Energy (au) 0.4661736
Chemical Potential (kJ mol^{-1}) 1036.22
Dispersion Correction (au) PBE0/def2-TZVPP -0.09605401

xyz coordinates
63

Mn	1.8614852	1.8272271	0.7600694
C	2.1430144	2.1835792	2.5350904
C	3.6016826	1.4888872	0.4378122
C	2.0500388	3.5079382	0.2825505
O	2.1338044	4.6332195	-0.0284058
O	4.7256063	1.2775391	0.2046422
O	2.3623552	2.4227320	3.6562518
C	-0.6663726	0.7692076	1.3127486
C	-2.1059430	0.5460214	1.5349114
C	0.3567525	-0.2822895	1.5951215
C	1.2683272	-0.7312598	0.5727640
C	2.2161006	-1.7194815	0.9236190
C	2.3031394	-2.2513658	2.2233758
C	1.3933620	-1.8126449	3.2182310
C	0.4345636	-0.8518925	2.8895608
H	2.9102648	-2.1009480	0.1590779
O	3.2741955	-3.1777885	2.4271941
H	1.4299679	-2.2109247	4.2423712
H	-0.2749580	-0.5153012	3.6629406
N	-0.1318268	1.8801622	0.8840006
C	1.3685189	1.7584076	-2.3716643
C	2.4382710	2.5544955	-2.8558632
C	2.3323582	3.2495815	-4.0700113
C	1.1444819	3.1943154	-4.8210521
C	0.0650148	2.4276527	-4.3461588
C	0.1764819	1.7142486	-3.1433719
H	3.3698502	2.6152232	-2.2702919
H	3.1851013	3.8506290	-4.4281487
H	1.0581312	3.7523870	-5.7681421
H	-0.8747790	2.3805308	-4.9221699
H	-0.6704910	1.1094425	-2.7804303
C	1.4573975	1.0464670	-1.0879726
C	1.1980761	-0.2803654	-0.8690963
C	0.9720158	-1.3465352	-1.8889216
C	-4.8941321	0.1039928	1.9039173
C	-4.0078525	-0.9885286	1.7460380
C	-2.6427381	-0.7687003	1.5695352
C	-3.0037724	1.6266523	1.7078248
C	-4.3771537	1.4209155	1.8878551
O	-6.2033108	-0.2092240	2.0707061
H	-4.4261663	-2.0069376	1.7604853
H	-1.9693135	-1.6301234	1.4357209
H	-2.6227897	2.6613351	1.7321583
H	-5.0348254	2.2906856	2.0302847
H	-0.7729605	2.6285282	0.5818223
C	-7.1518246	0.8383416	2.2158342
H	-6.9520461	1.4508053	3.1261551
H	-8.1385144	0.3447221	2.3199327
H	-7.1681416	1.5035833	1.3209379
C	3.4333409	-3.7393500	3.7213342
H	2.5244138	-4.3010910	4.0423651
H	3.6718864	-2.9596801	4.4819780

```
H     4.2852339   -4.4442321    3.6450391
C     0.5273194   -3.4043260   -3.8078954
C    -0.1128372   -3.4508009   -2.5573833
C     0.1115124   -2.4386197   -1.6112747
C     1.6196019   -1.3247218   -3.1499652
C     1.3975103   -2.3370985   -4.0950872
H     0.3562515   -4.2003302   -4.5515579
H    -0.7944227   -4.2836290   -2.3148456
H    -0.3988472   -2.4876558   -0.6346783
H     2.3189617   -0.5071702   -3.3820013
H     1.9196277   -2.2964399   -5.0658676

$vibrational spectrum
#  mode     symmetry     wave number   IR intensity    selection rules
#                         cm**(-1)        km/mol         IR     RAMAN
     1                        0.00         0.00000        -       -
     2                        0.00         0.00000        -       -
     3                        0.00         0.00000        -       -
     4                        0.00         0.00000        -       -
     5                        0.00         0.00000        -       -
     6                        0.00         0.00000        -       -
     7        a              16.39         0.11460       YES     YES
     8        a              21.37         0.06926       YES     YES
     9        a              27.70         0.35408       YES     YES
    10        a              34.81         0.58605       YES     YES
    11        a              36.88         0.42762       YES     YES
    12        a              45.70         0.11059       YES     YES
    13        a              47.61         0.39275       YES     YES
    14        a              50.03         0.43572       YES     YES
    15        a              54.24         0.47325       YES     YES
    16        a              56.72         0.23312       YES     YES
    17        a              66.85         0.24090       YES     YES
    18        a              73.98         0.70798       YES     YES
    19        a              83.56         0.35825       YES     YES
    20        a              86.20         0.95309       YES     YES
    21        a              89.67         0.50301       YES     YES
    22        a              98.55         0.80454       YES     YES
    23        a             103.82         1.38037       YES     YES
    24        a             117.97         0.55286       YES     YES
    25        a             130.79         1.31610       YES     YES
    26        a             146.21         0.78006       YES     YES
    27        a             149.50         0.24248       YES     YES
    28        a             154.90         1.76928       YES     YES
    29        a             174.48         1.03601       YES     YES
    30        a             188.27         0.72738       YES     YES
    31        a             204.30         1.41449       YES     YES
    32        a             216.12         0.28070       YES     YES
    33        a             229.06         0.17449       YES     YES
    34        a             232.97         1.30367       YES     YES
    35        a             241.26         1.73607       YES     YES
    36        a             250.95         1.60374       YES     YES
    37        a             253.42         1.45897       YES     YES
    38        a             260.82         1.45105       YES     YES
    39        a             295.61         3.47743       YES     YES
    40        a             296.85         2.88335       YES     YES
    41        a             319.22         3.17226       YES     YES
    42        a             346.77         0.76517       YES     YES
    43        a             401.35         0.73305       YES     YES
    44        a             403.69         0.30821       YES     YES
    45        a             406.71         0.54868       YES     YES
    46        a             413.00         0.48532       YES     YES
    47        a             432.96         2.64496       YES     YES
    48        a             436.95        11.61757       YES     YES
```

49	a	445.85	6.05936	YES	YES
50	a	458.16	3.03188	YES	YES

3fa

```
SCF Energy (au) BP86/SV(P)                   -2391.992336901
SCF Energy (au) PBE0/def2-TZVPP              -2391.554013615
SCF Energy (au) PBE0/def2-TZVPP              -2391.5628786450 (Toluene Correction)
Zero Point Energy (au)                       0.3230498
Chemical Potential (kJ mol-1)                696.59
Dispersion Correction (au) PBE0/def2-TZVPP  -0.06995959
```

xyz coordinates
45

```
Mn    1.3893256    0.0876337    0.7929616
C     1.9375193    0.2572834    2.5295937
C     2.9494388   -0.6115109    0.2520024
C     1.9693035    1.6982189    0.3876714
O     2.3914858    2.7574452    0.1236272
O     3.9613387   -1.0608219   -0.1172982
O     2.3248523    0.3716485    3.6248438
C    -1.1604195   -0.6563062    1.6342081
C    -1.2997661    1.6357765    1.1543460
C    -2.6639989    1.6633687    1.5209604
C    -3.2631552    0.4780593    1.9666187
C    -2.5078469   -0.7036217    2.0277464
C    -0.2731292   -1.8579394    1.6496923
C     0.2795776   -2.3939772    0.4371837
C     0.9424123   -3.6500144    0.5190256
C     1.0927035   -4.3297003    1.7297636
C     0.5719453   -3.7794209    2.9200566
C    -0.1155225   -2.5648225    2.8710743
O    -0.5978210    2.6959424    0.7265859
H    -3.2432555    2.5949506    1.4605104
H    -4.3259905    0.4779088    2.2582762
H    -2.9490841   -1.6574740    2.3522989
H     1.3483386   -4.0826774   -0.4104427
H     1.6229371   -5.2962157    1.7540469
H     0.6957706   -4.3060604    3.8800926
H    -0.5497869   -2.1386511    3.7905931
N    -0.5745361    0.4961078    1.2257988
C     0.5399501    0.2680166   -2.3132376
C     1.6306738    1.0208220   -2.8181526
C     1.5528546    1.6673793   -4.0605371
C     0.3748659    1.6006528   -4.8265155
C    -0.7237283    0.8716036   -4.3376641
C    -0.6410425    0.2119474   -3.1015705
H     2.5602130    1.0809591   -2.2308591
H     2.4224991    2.2343528   -4.4334197
H     0.3123101    2.1192231   -5.7977797
H    -1.6560547    0.8162619   -4.9252319
H    -1.5090957   -0.3495544   -2.7178697
C     0.6145950   -0.4458345   -1.0281940
C     0.1482964   -1.7181938   -0.8798387
H    -0.1964605   -2.3395073   -1.7343853
C    -1.2373787    3.9568516    0.5722660
H    -2.0652377    3.9017843   -0.1713883
H    -0.4521083    4.6418740    0.1979794
H    -1.6277885    4.3362318    1.5445606
```

$vibrational spectrum

# mode	symmetry	wave number cm**(-1)	IR intensity km/mol	selection rules IR	RAMAN
1		0.00	0.00000	-	-
2		0.00	0.00000	-	-
3		0.00	0.00000	-	-

4		0.00	0.00000	-	-
5		0.00	0.00000	-	-
6		0.00	0.00000	-	-
7	a	28.02	0.61831	YES	YES
8	a	35.88	0.12888	YES	YES
9	a	40.30	0.55568	YES	YES
10	a	46.39	0.18241	YES	YES
11	a	54.16	0.21157	YES	YES
12	a	59.73	0.20736	YES	YES
13	a	71.76	1.45852	YES	YES
14	a	79.33	0.73701	YES	YES
15	a	86.90	0.34167	YES	YES
16	a	95.76	0.47929	YES	YES
17	a	102.86	0.18488	YES	YES
18	a	115.16	0.56838	YES	YES
19	a	116.40	1.09103	YES	YES
20	a	136.46	0.21938	YES	YES
21	a	145.56	1.21576	YES	YES
22	a	170.97	0.23423	YES	YES
23	a	183.55	0.65378	YES	YES
24	a	191.89	0.07650	YES	YES
25	a	207.95	0.58322	YES	YES
26	a	236.01	0.87400	YES	YES
27	a	261.57	0.19107	YES	YES
28	a	268.32	0.04572	YES	YES
29	a	293.06	1.07449	YES	YES
30	a	314.39	0.85664	YES	YES
31	a	326.70	1.24900	YES	YES
32	a	359.74	1.05307	YES	YES
33	a	407.02	0.18747	YES	YES
34	a	411.91	1.53031	YES	YES
35	a	443.70	2.15286	YES	YES
36	a	454.02	2.16238	YES	YES
37	a	464.43	3.10826	YES	YES
38	a	470.00	2.56835	YES	YES
39	a	475.86	2.54220	YES	YES
40	a	482.34	5.04952	YES	YES
41	a	498.98	1.16430	YES	YES
42	a	517.77	5.41991	YES	YES
43	a	526.65	3.00938	YES	YES
44	a	538.96	3.75754	YES	YES
45	a	546.71	2.40756	YES	YES
46	a	552.90	6.08976	YES	YES
47	a	562.03	1.58074	YES	YES
48	a	587.33	1.67984	YES	YES
49	a	613.53	0.81749	YES	YES
50	a	621.75	26.77573	YES	YES

3fa′

SCF Energy (au) BP86/SV(P)	-2391.990036695	
SCF Energy (au) PBE0/def2-TZVPP	-2391.552440556	
SCF Energy (au) PBE0/def2-TZVPP	-2391.5618337702	(Toluene Correction)
Zero Point Energy (au)	0.3230933	
Chemical Potential (kJ mol^{-1})	696.35	
Dispersion Correction (au) PBE0/def2-TZVPP	-0.06902345	

xyz coordinates
45

Mn	1.5958874	1.2194711	0.8192433
C	1.9119078	1.6502330	2.5727878
C	3.2396413	0.5500429	0.5694944
C	2.1294424	2.7816886	0.2114912
O	2.5047679	3.7997952	-0.2258318
O	4.3043764	0.1080234	0.3896308
O	2.1565181	1.9463207	3.6751769
C	-0.9726965	0.3512824	1.3474514
C	-1.2238104	2.6135044	0.7817135
C	-2.6236647	2.5349199	0.9615354
C	-3.1820694	1.3119655	1.3556269
C	-2.3514676	0.1968958	1.5560837
C	0.0240809	-0.7483572	1.5464177
C	0.7453824	-1.3160323	0.4449884
C	1.5727749	-2.4420447	0.7078335
C	1.7033937	-2.9750288	1.9919739
C	0.9959331	-2.4048745	3.0721509
C	0.1598273	-1.3105294	2.8454942
O	-0.5535451	3.7193529	0.4183955
H	-3.2624194	3.4138406	0.7985656
H	-4.2714651	1.2304532	1.5015322
H	-2.7555639	-0.7817643	1.8540149
H	2.1196722	-2.8922407	-0.1363837
H	2.3642676	-3.8411970	2.1611832
H	1.0999333	-2.8189897	4.0880986
H	-0.4065927	-0.8644197	3.6795261
N	-0.4311686	1.5383193	0.9824148
H	1.1435329	1.0777133	-1.9829699
C	1.1108779	0.5381239	-1.0164736
C	0.6659029	-0.7479829	-0.9481779
C	0.1936539	-1.6064361	-2.0644108
C	-0.7616451	-3.2272995	-4.2059009
C	-1.0822533	-3.5470289	-2.8759322
C	-0.6110365	-2.7482870	-1.8214930
C	0.5126253	-1.3050842	-3.4133187
C	0.0393637	-2.0992962	-4.4663745
H	-1.1272918	-3.8553737	-5.0351199
H	-1.7094212	-4.4272095	-2.6544714
H	-0.8813878	-3.0085416	-0.7844734
H	1.1592413	-0.4402662	-3.6334657
H	0.3090477	-1.8425405	-5.5048191
C	-1.2657020	4.9239144	0.1631691
H	-1.9903366	4.7973859	-0.6740308
H	-0.4972931	5.6669884	-0.1255121
H	-1.8012214	5.2805898	1.0731657

$vibrational spectrum

# mode	symmetry	wave number	IR intensity	selection rules	
#		cm**(-1)	km/mol	IR	RAMAN
1		0.00	0.00000	-	-
2		0.00	0.00000	-	-
3		0.00	0.00000	-	-

4		0.00	0.00000	-	-
5		0.00	0.00000	-	-
6		0.00	0.00000	-	-
7	a	26.43	0.64410	YES	YES
8	a	36.00	0.10988	YES	YES
9	a	39.31	0.64830	YES	YES
10	a	46.99	0.07932	YES	YES
11	a	53.88	0.60268	YES	YES
12	a	57.99	0.14448	YES	YES
13	a	78.15	0.85951	YES	YES
14	a	79.22	0.86036	YES	YES
15	a	86.49	0.36867	YES	YES
16	a	92.13	1.75821	YES	YES
17	a	96.91	0.41554	YES	YES
18	a	109.81	0.52623	YES	YES
19	a	112.58	0.93413	YES	YES
20	a	134.50	0.80920	YES	YES
21	a	151.79	0.07598	YES	YES
22	a	172.87	0.86313	YES	YES
23	a	194.28	0.18231	YES	YES
24	a	198.29	0.16023	YES	YES
25	a	218.11	0.73152	YES	YES
26	a	241.18	0.24161	YES	YES
27	a	259.58	0.18536	YES	YES
28	a	270.76	0.41493	YES	YES
29	a	285.93	0.46475	YES	YES
30	a	300.73	1.69595	YES	YES
31	a	330.64	1.18946	YES	YES
32	a	371.83	0.29010	YES	YES
33	a	404.19	0.17024	YES	YES
34	a	414.83	2.07391	YES	YES
35	a	442.03	3.05176	YES	YES
36	a	458.75	0.49513	YES	YES
37	a	466.64	2.23317	YES	YES
38	a	471.52	1.38698	YES	YES
39	a	473.56	6.02926	YES	YES
40	a	477.32	1.32248	YES	YES
41	a	495.82	2.27249	YES	YES
42	a	524.09	10.32641	YES	YES
43	a	527.91	4.64607	YES	YES
44	a	544.34	4.44421	YES	YES
45	a	551.51	6.18662	YES	YES
46	a	572.66	6.36547	YES	YES
47	a	585.87	2.13074	YES	YES
48	a	605.12	10.06900	YES	YES
49	a	612.62	11.75680	YES	YES
50	a	616.40	14.82095	YES	YES

3ga

```
SCF Energy (au) BP86/SV(P)                   -2508.432275552
SCF Energy (au) PBE0/def2-TZVPP              -2507.958331998
SCF Energy (au) PBE0/def2-TZVPP              -2507.9668305178 (Toluene Correction)
Zero Point Energy (au)                       0.3699565
Chemical Potential (kJ mol-1)                811.35
Dispersion Correction (au) PBE0/def2-TZVPP -0.08488781

xyz coordinates
51

Mn    1.2727708   -0.4332971    0.8077556
C     1.7707572   -0.1092857    2.5352243
C     2.7991138   -1.2895947    0.4156375
C     2.0107523    1.0823652    0.2975907
O     2.5731236    2.0592395   -0.0167825
O     3.7865517   -1.8502685    0.1476760
O     2.1262832    0.1019435    3.6266265
C    -1.2039849   -1.1041463    1.6676312
C    -1.4649532    1.1863673    1.1855316
C    -2.7829765    1.1473506    1.6963750
C    -3.3128516   -0.0465461    2.1956206
C    -2.5085134   -1.1959060    2.1787170
C    -0.2816629   -2.2851359    1.7060502
C     0.1710193   -2.9476128    0.5144462
C     0.7821341   -4.2238243    0.6584667
C     0.9927149   -4.8035228    1.9103597
C     0.5902302   -4.1259705    3.0824595
C    -0.0546454   -2.8940219    2.9733010
C    -0.9384016    2.4744860    0.6543132
H    -3.3838601    2.0688894    1.6725252
H    -4.3432384   -0.0855125    2.5848200
H    -2.8746612   -2.1647186    2.5513275
H     1.1086373   -4.7475062   -0.2551866
H     1.4833475   -5.7883762    1.9831589
H     0.7690507   -4.5707846    4.0745333
H    -0.4056870   -2.3746036    3.8804688
N    -0.6960826    0.0637399    1.1885210
C     0.5422277   -0.4556476   -2.3450926
C     1.6806026    0.2122683   -2.8646464
C     1.6670712    0.7770619   -4.1489600
C     0.5086847    0.7121400   -4.9447528
C    -0.6353203    0.0661173   -4.4435357
C    -0.6175203   -0.5130755   -3.1649267
H     2.5954765    0.2701861   -2.2546878
H     2.5725715    1.2772438   -4.5319369
H     0.4969203    1.1670741   -5.9491877
H    -1.5526615    0.0133452   -5.0541335
H    -1.5208462   -1.0092120   -2.7729391
C     0.5483680   -1.0846884   -1.0123511
C     0.0500190   -2.3411793   -0.8280562
H    -0.2844929   -2.9933694   -1.6624084
C    -0.1022246    4.9745170   -0.3583789
C    -0.5250942    4.8634252    0.9777925
C    -0.9495964    3.6232699    1.4789414
C    -0.5122545    2.5952404   -0.6859912
C    -0.0998301    3.8397572   -1.1866599
H     0.2288094    5.9491154   -0.7537595
H    -0.5213643    5.7475064    1.6364710
H    -1.2756174    3.5363344    2.5289025
H    -0.5093230    1.7116159   -1.3418794
H     0.2304268    3.9172061   -2.2349916
```

$vibrational spectrum

# mode	symmetry	wave number cm**(-1)	IR intensity km/mol	selection rules IR	RAMAN
1		0.00	0.00000	-	-
2		0.00	0.00000	-	-
3		0.00	0.00000	-	-
4		0.00	0.00000	-	-
5		0.00	0.00000	-	-
6		0.00	0.00000	-	-
7	a	25.84	0.04845	YES	YES
8	a	26.55	0.02052	YES	YES
9	a	35.80	0.32049	YES	YES
10	a	39.25	0.03499	YES	YES
11	a	50.44	0.01163	YES	YES
12	a	54.68	0.65930	YES	YES
13	a	62.61	0.01939	YES	YES
14	a	69.07	0.26530	YES	YES
15	a	80.18	1.09165	YES	YES
16	a	83.57	0.18719	YES	YES
17	a	91.28	0.07543	YES	YES
18	a	106.38	0.19122	YES	YES
19	a	107.60	0.29624	YES	YES
20	a	110.51	0.06144	YES	YES
21	a	125.01	1.02495	YES	YES
22	a	143.88	0.26257	YES	YES
23	a	154.72	0.83859	YES	YES
24	a	173.13	1.39150	YES	YES
25	a	190.08	0.40542	YES	YES
26	a	198.81	0.17350	YES	YES
27	a	230.09	0.74020	YES	YES
28	a	258.49	0.10339	YES	YES
29	a	265.26	0.79766	YES	YES
30	a	299.31	0.13424	YES	YES
31	a	320.51	1.06814	YES	YES
32	a	345.08	1.58179	YES	YES
33	a	372.24	0.09462	YES	YES
34	a	379.45	1.38213	YES	YES
35	a	400.84	0.39030	YES	YES
36	a	406.61	0.47180	YES	YES
37	a	418.10	1.20687	YES	YES
38	a	448.19	0.52402	YES	YES
39	a	455.32	0.83848	YES	YES
40	a	466.65	0.88780	YES	YES
41	a	474.11	0.33446	YES	YES
42	a	489.02	4.93957	YES	YES
43	a	501.61	1.47829	YES	YES
44	a	503.21	11.56780	YES	YES
45	a	523.20	2.12663	YES	YES
46	a	532.27	2.99148	YES	YES
47	a	540.69	5.81623	YES	YES
48	a	546.42	3.39454	YES	YES
49	a	556.83	2.45245	YES	YES
50	a	559.84	2.16353	YES	YES

3ga'

```
SCF Energy (au) BP86/SV(P)                    -2508.431196130
SCF Energy (au) PBE0/def2-TZVPP               -2507.957419935
SCF Energy (au) PBE0/def2-TZVPP               -2507.9666554808 (Toluene Correction)
Zero Point Energy (au)                        0.3699454
Chemical Potential (kJ mol-1)                 810.86
Dispersion Correction (au) PBE0/def2-TZVPP   -0.08233166

xyz coordinates
51

Mn    1.5028971    0.4619076    0.8244350
C     1.8610594    0.9959175    2.5377272
C     3.0723701   -0.3902685    0.6581873
C     2.2134124    1.8824475    0.0628351
O     2.7210324    2.7732632   -0.4990363
O     4.0913041   -0.9452745    0.5388870
O     2.1319073    1.3619097    3.6139335
C    -1.0799484   -0.2214387    1.2500738
C    -1.2802869    2.0496447    0.6658923
C    -2.6890924    1.9284459    0.6707401
C    -3.2912878    0.7052950    0.9841893
C    -2.4703122   -0.3910427    1.2953473
C    -0.1231398   -1.3378904    1.5565186
C     0.5226403   -2.0672512    0.5052292
C     1.2369224   -3.2447021    0.8533463
C     1.3388621   -3.6695889    2.1800907
C     0.7224657   -2.9332044    3.2154580
C    -0.0086079   -1.7861231    2.9021516
C    -0.6547343    3.3735215    0.4000120
H    -3.2976659    2.8196640    0.4539523
H    -4.3890445    0.6080301    0.9977374
H    -2.8893230   -1.3749234    1.5560449
H     1.7260810   -3.8169703    0.0487384
H     1.9121756   -4.5804815    2.4201307
H     0.8104040   -3.2633623    4.2631026
H    -0.5171513   -1.2181188    3.6987699
N    -0.5033870    0.9675818    0.9360635
H     1.0805268    0.2185027   -1.9755761
C     0.9985047   -0.2854715   -0.9926964
C     0.5072780   -1.5551744   -0.9051413
C     0.0395398   -2.4266975   -2.0123330
C    -0.9095803   -4.0676480   -4.1396019
C    -1.3142116   -4.3163081   -2.8172489
C    -0.8461257   -3.5069520   -1.7695609
C     0.4428470   -2.1974358   -3.3524435
C    -0.0275756   -3.0017621   -4.3993144
H    -1.2735282   -4.7034473   -4.9636520
H    -2.0055557   -5.1473674   -2.5972797
H    -1.1830726   -3.7081036   -0.7387922
H     1.1530767   -1.3830656   -3.5690219
H     0.3076621   -2.8027175   -5.4312424
C     0.4275050    5.9411982   -0.0593172
C     0.7627062    5.2324467    1.1068621
C     0.2286643    3.9558823    1.3363159
C    -0.9986846    4.0989691   -0.7624859
C    -0.4540578    5.3716305   -0.9938517
H     0.8562097    6.9404445   -0.2417441
H     1.4492572    5.6761343    1.8462932
H     0.4867038    3.4108470    2.2577910
H    -1.6816539    3.6493267   -1.5025631
H    -0.7159876    5.9197807   -1.9139531
```

```
$vibrational spectrum
#  mode     symmetry     wave number   IR intensity    selection rules
#                         cm**(-1)        km/mol         IR     RAMAN
     1                      0.00         0.00000         -       -
     2                      0.00         0.00000         -       -
     3                      0.00         0.00000         -       -
     4                      0.00         0.00000         -       -
     5                      0.00         0.00000         -       -
     6                      0.00         0.00000         -       -
     7         a           21.80         0.16220        YES     YES
     8         a           28.64         0.04894        YES     YES
     9         a           36.93         0.20170        YES     YES
    10         a           41.81         0.24131        YES     YES
    11         a           46.48         0.08780        YES     YES
    12         a           56.43         0.06689        YES     YES
    13         a           63.55         0.47475        YES     YES
    14         a           69.71         0.08274        YES     YES
    15         a           80.44         0.85743        YES     YES
    16         a           83.10         0.29376        YES     YES
    17         a           90.96         0.67737        YES     YES
    18         a           97.65         0.51042        YES     YES
    19         a          104.49         0.67564        YES     YES
    20         a          109.49         0.09221        YES     YES
    21         a          121.43         0.40199        YES     YES
    22         a          144.90         1.16747        YES     YES
    23         a          160.09         0.03778        YES     YES
    24         a          175.91         0.87185        YES     YES
    25         a          202.30         0.09861        YES     YES
    26         a          209.00         1.87276        YES     YES
    27         a          232.32         0.56181        YES     YES
    28         a          247.26         0.89539        YES     YES
    29         a          279.58         0.06946        YES     YES
    30         a          291.77         0.24440        YES     YES
    31         a          317.63         0.51329        YES     YES
    32         a          332.98         1.35842        YES     YES
    33         a          354.30         0.79297        YES     YES
    34         a          401.06         0.13085        YES     YES
    35         a          402.72         1.42056        YES     YES
    36         a          404.75         0.25181        YES     YES
    37         a          414.04         1.99353        YES     YES
    38         a          456.26         2.32840        YES     YES
    39         a          458.63         0.40697        YES     YES
    40         a          466.41         1.26669        YES     YES
    41         a          473.63         2.91763        YES     YES
    42         a          475.99         0.58484        YES     YES
    43         a          495.71         3.10966        YES     YES
    44         a          507.57         5.64543        YES     YES
    45         a          522.33        11.32701        YES     YES
    46         a          530.23         5.55672        YES     YES
    47         a          542.98         9.38660        YES     YES
    48         a          555.30         2.52328        YES     YES
    49         a          573.57         6.35314        YES     YES
    50         a          604.68         6.94203        YES     YES
```

3ha

SCF Energy (au) BP86/SV(P)	-2354.896525758	
SCF Energy (au) PBE0/def2-TZVPP	-2354.447582458	
SCF Energy (au) PBE0/def2-TZVPP	-2354.4561647301	(Toluene Correction)
Zero Point Energy (au)	0.3247184	
Chemical Potential (kJ mol^{-1})	698.72	
Dispersion Correction (au) PBE0/def2-TZVPP	-0.06734630	

45

Mn	1.8497483	1.1028289	0.4584999
C	2.3962619	1.3587991	2.1905971
C	3.5333785	0.9238504	-0.1600726
C	1.8660182	2.8121141	0.0884741
O	1.8382320	3.9560846	-0.1641401
O	4.6199855	0.8059896	-0.5663851
O	2.7520255	1.5276083	3.2875670
C	-0.7054563	-0.0098118	1.3823506
C	-2.1394295	0.0003492	1.7665265
C	0.1106602	-1.2432892	1.4872944
C	0.9370762	-1.6614085	0.3886899
C	1.6651054	-2.8726502	0.5552985
C	1.6208297	-3.6127409	1.7405413
C	0.8141070	-3.1833551	2.8134205
C	0.0544973	-2.0172268	2.6716259
H	2.2822125	-3.2274646	-0.2869896
H	2.2163630	-4.5362864	1.8305783
H	0.7747562	-3.7599084	3.7515646
H	-0.5937026	-1.6787484	3.4963007
N	-0.0911736	1.0514123	0.9302620
C	1.0975114	0.7866837	-2.6460164
C	1.9569420	1.8049844	-3.1326176
C	1.8791743	2.2488333	-4.4603469
C	0.9228710	1.7061809	-5.3368813
C	0.0454810	0.7109950	-4.8701619
C	0.1322518	0.2579281	-3.5462639
H	2.7068543	2.2457189	-2.4577649
H	2.5690828	3.0340286	-4.8122970
H	0.8536291	2.0660586	-6.3769160
H	-0.7189774	0.2877369	-5.5436486
H	-0.5735378	-0.5073957	-3.1825160
C	1.1784931	0.2943742	-1.2632088
C	0.9589983	-1.0244135	-0.9561433
H	0.9396469	-1.7668659	-1.7838619
C	-4.8945020	0.0414844	2.4180097
C	-4.3029510	-1.1350330	1.9253431
C	-2.9367949	-1.1594302	1.6077318
C	-2.7462638	1.1766638	2.2726661
C	-4.1106720	1.1961662	2.5941723
H	-5.9670312	0.0575576	2.6722177
H	-4.9124069	-2.0426069	1.7835704
H	-2.4797178	-2.0799137	1.2112054
H	-2.1355333	2.0780554	2.4498845
H	-4.5635049	2.1166377	2.9976601
H	-0.7005380	1.8694253	0.7741796

$vibrational spectrum

# mode	symmetry	wave number	IR intensity	selection rules	
#		cm**(-1)	km/mol	IR	RAMAN
1		0.00	0.00000	-	-
2		0.00	0.00000	-	-
3		0.00	0.00000	-	-
4		0.00	0.00000	-	-

5		0.00	0.00000	-	-
6		0.00	0.00000	-	-
7	a	18.81	0.42118	YES	YES
8	a	25.24	0.76632	YES	YES
9	a	32.01	0.00412	YES	YES
10	a	48.45	0.03318	YES	YES
11	a	51.30	0.16810	YES	YES
12	a	54.97	0.07239	YES	YES
13	a	63.80	0.33920	YES	YES
14	a	76.09	0.46646	YES	YES
15	a	82.01	0.53374	YES	YES
16	a	87.47	0.05751	YES	YES
17	a	95.81	0.15715	YES	YES
18	a	101.62	0.38770	YES	YES
19	a	121.65	0.62825	YES	YES
20	a	133.09	0.95562	YES	YES
21	a	158.91	0.65598	YES	YES
22	a	171.28	2.93053	YES	YES
23	a	204.45	8.52771	YES	YES
24	a	221.24	1.12574	YES	YES
25	a	225.57	2.57217	YES	YES
26	a	260.72	3.30447	YES	YES
27	a	262.54	0.90363	YES	YES
28	a	291.03	1.30239	YES	YES
29	a	312.22	11.28529	YES	YES
30	a	400.73	1.19342	YES	YES
31	a	405.42	0.45457	YES	YES
32	a	407.19	1.70779	YES	YES
33	a	420.52	2.13785	YES	YES
34	a	443.09	0.05262	YES	YES
35	a	445.72	2.31478	YES	YES
36	a	454.94	1.77653	YES	YES
37	a	468.28	3.39004	YES	YES
38	a	476.13	8.41032	YES	YES
39	a	481.68	2.49903	YES	YES
40	a	505.35	3.70312	YES	YES
41	a	515.80	16.83659	YES	YES
42	a	529.78	2.98947	YES	YES
43	a	534.51	2.41560	YES	YES
44	a	557.25	0.74365	YES	YES
45	a	569.79	5.61717	YES	YES
46	a	587.36	23.70126	YES	YES
47	a	610.02	1.28155	YES	YES
48	a	613.01	5.13310	YES	YES
49	a	618.55	45.97154	YES	YES
50	a	622.16	29.74323	YES	YES

3ha′

```
SCF Energy (au) BP86/SV(P)                   -2354.891997893
SCF Energy (au) PBE0/def2-TZVPP              -2354.444420890
SCF Energy (au) PBE0/def2-TZVPP              -2354.4536324230 (Toluene Correction)
Zero Point Energy (au)                       0.3244849
Chemical Potential (kJ mol-1)                697.48
Dispersion Correction (au) PBE0/def2-TZVPP -0.06855975

xyz coordinates
45

Mn    2.1473163    1.8360644    0.6549980
C     2.5134529    2.0827923    2.4407545
C     3.8276642    1.3205676    0.2474063
C     2.5110158    3.4912763    0.2106829
O     2.7127039    4.5988164   -0.1110798
O     4.9051974    0.9874349   -0.0456415
O     2.7825573    2.2571479    3.5616359
C    -0.5045838    1.0098222    1.1597239
C     0.3466433   -0.2156809    1.2987456
C     1.1533074   -0.6772435    0.2074169
C     1.9544921   -1.8298941    0.4274221
C     1.9730232   -2.4927127    1.6587154
C     1.1679062   -2.0335724    2.7208425
C     0.3570502   -0.9098951    2.5354940
H     2.5671394   -2.2034856   -0.4090707
H     2.6173836   -3.3767309    1.7954675
H     1.1768314   -2.5529085    3.6928652
H    -0.2797052   -0.5454997    3.3581044
N     0.1784474    2.0822057    0.8741866
H     1.7080949    1.7931007   -2.1537943
C     1.6406430    1.2400589   -1.1978645
C     1.1492211   -0.0302570   -1.1595934
C     0.6810864   -0.8366841   -2.3202024
C    -0.2729277   -2.3459176   -4.5442658
C    -0.6369745   -2.7028301   -3.2354055
C    -0.1647223   -1.9605573   -2.1409611
C     1.0454677   -0.4997397   -3.6491124
C     0.5730529   -1.2384981   -4.7421129
H    -0.6385616   -2.9299659   -5.4049812
H    -1.2979833   -3.5687365   -3.0615359
H    -0.4650140   -2.2553027   -1.1216221
H     1.7269371    0.3483022   -3.8237653
H     0.8789089   -0.9536970   -5.7629458
H    -0.3637364    2.9429415    0.7029938
C    -1.9679026    0.9717771    1.3689105
C    -4.7780709    0.8918705    1.7096646
C    -4.0775780    2.0974632    1.9001518
C    -2.6873017    2.1379152    1.7336900
C    -2.6839578   -0.2379989    1.1945194
C    -4.0764003   -0.2743310    1.3580252
H    -5.8718745    0.8613632    1.8428671
H    -4.6189613    3.0118872    2.1929712
H    -2.1503338    3.0831105    1.9209199
H    -2.1398434   -1.1520296    0.9082756
H    -4.6191108   -1.2217489    1.2065037

$vibrational spectrum
#  mode     symmetry     wave number   IR intensity    selection rules
#                         cm**(-1)        km/mol          IR     RAMAN
     1                        0.00         0.00000         -       -
     2                        0.00         0.00000         -       -
     3                        0.00         0.00000         -       -
```

4		0.00	0.00000	-	-
5		0.00	0.00000	-	-
6		0.00	0.00000	-	-
7	a	19.54	0.06232	YES	YES
8	a	30.72	0.02671	YES	YES
9	a	33.19	0.15313	YES	YES
10	a	35.77	0.03630	YES	YES
11	a	45.96	0.10263	YES	YES
12	a	48.02	0.04022	YES	YES
13	a	70.48	0.39725	YES	YES
14	a	74.21	0.99841	YES	YES
15	a	80.25	0.36004	YES	YES
16	a	82.52	0.06077	YES	YES
17	a	88.33	0.24855	YES	YES
18	a	98.18	0.28632	YES	YES
19	a	118.70	0.07417	YES	YES
20	a	139.85	0.35431	YES	YES
21	a	159.69	2.51650	YES	YES
22	a	180.91	1.89325	YES	YES
23	a	188.41	0.83797	YES	YES
24	a	202.24	0.22099	YES	YES
25	a	238.03	1.15483	YES	YES
26	a	272.89	5.68818	YES	YES
27	a	279.98	3.23812	YES	YES
28	a	299.79	0.96235	YES	YES
29	a	311.67	1.93608	YES	YES
30	a	399.61	0.79350	YES	YES
31	a	401.93	0.55369	YES	YES
32	a	405.62	1.29219	YES	YES
33	a	411.42	4.36505	YES	YES
34	a	448.49	2.32542	YES	YES
35	a	454.57	0.33329	YES	YES
36	a	462.84	0.31148	YES	YES
37	a	464.29	11.86901	YES	YES
38	a	473.01	1.22050	YES	YES
39	a	481.32	7.29869	YES	YES
40	a	509.53	3.69401	YES	YES
41	a	516.36	36.11068	YES	YES
42	a	528.45	2.40935	YES	YES
43	a	551.04	3.74614	YES	YES
44	a	572.47	7.19075	YES	YES
45	a	576.98	21.92925	YES	YES
46	a	607.33	4.65353	YES	YES
47	a	609.16	12.77565	YES	YES
48	a	611.63	38.74875	YES	YES
49	a	614.73	0.08764	YES	YES
50	a	621.80	51.19677	YES	YES

3ia

SCF Energy (au) BP86/SV(P)	-2183.142508002
SCF Energy (au) PBE0/def2-TZVPP	-2182.728425547
SCF Energy (au) PBE0/def2-TZVPP	-2182.7357796019 (Toluene Correction)
Zero Point Energy (au)	0.2600039
Chemical Potential (kJ mol^{-1})	539.07
Dispersion Correction (au) PBE0/def2-TZVPP	-0.05212511

xyz coordinates
37

Mn	1.1217044	1.2220039	0.8753182
C	1.8076113	1.3348070	2.5749408
C	2.7421494	1.2108299	0.1355115
C	1.0314197	2.9638585	0.6742824
O	0.9561462	4.1202638	0.5160197
O	3.8034237	1.1815147	-0.3512306
O	2.2450875	1.3934439	3.6532134
C	-1.4143192	0.1032722	1.9810110
C	-2.7959384	0.3246958	2.5515022
C	-0.8039152	-1.2334355	1.9498273
C	0.0519542	-1.6099950	0.8541980
C	0.6098266	-2.9207387	0.9125915
C	0.3739047	-3.7930724	1.9771542
C	-0.4827170	-3.4144254	3.0316683
C	-1.0800704	-2.1522323	2.9934884
H	1.2462492	-3.2504250	0.0744264
H	0.8490304	-4.7880668	1.9802317
H	-0.6839061	-4.1018357	3.8684900
H	-1.7572007	-1.8499947	3.8081085
O	-0.7925239	1.0949317	1.5367526
C	0.2629736	0.8812582	-2.1683061
C	0.9689452	2.0137198	-2.6526610
C	0.8763286	2.4070638	-3.9944473
C	0.0527202	1.6977131	-4.8871522
C	-0.6794746	0.5894331	-4.4235589
C	-0.5752960	0.1877177	-3.0853985
H	1.6024832	2.5887464	-1.9605507
H	1.4454542	3.2836622	-4.3459625
H	-0.0297790	2.0165111	-5.9395503
H	-1.3430462	0.0381636	-5.1108464
H	-1.1711905	-0.6675105	-2.7248941
C	0.3562945	0.4549763	-0.7681487
C	0.2344433	-0.8812744	-0.4193629
H	0.4020035	-1.6010875	-1.2503389
H	-3.1358146	1.3486402	2.2980282
H	-2.7730963	0.2339713	3.6619155
H	-3.5218654	-0.4271045	2.1737291

$vibrational spectrum

# mode	symmetry	wave number	IR intensity	selection rules	
#		cm**(-1)	km/mol	IR	RAMAN
1		0.00	0.00000	-	-
2		0.00	0.00000	-	-
3		0.00	0.00000	-	-
4		0.00	0.00000	-	-
5		0.00	0.00000	-	-
6		0.00	0.00000	-	-
7	a	14.40	2.54394	YES	YES
8	a	24.72	0.10778	YES	YES
9	a	43.63	1.36218	YES	YES
10	a	54.12	0.02016	YES	YES

11	a	60.67	0.24551	YES	YES
12	a	72.56	0.62244	YES	YES
13	a	79.27	0.63032	YES	YES
14	a	90.97	0.49506	YES	YES
15	a	95.26	0.11903	YES	YES
16	a	101.74	0.77547	YES	YES
17	a	111.63	1.15725	YES	YES
18	a	125.72	0.08378	YES	YES
19	a	152.51	0.33523	YES	YES
20	a	169.52	8.67190	YES	YES
21	a	192.37	0.29482	YES	YES
22	a	216.20	10.12995	YES	YES
23	a	232.41	0.24448	YES	YES
24	a	241.88	0.75526	YES	YES
25	a	252.52	6.68820	YES	YES
26	a	293.90	4.90373	YES	YES
27	a	353.55	2.36095	YES	YES
28	a	392.82	1.60206	YES	YES
29	a	406.09	0.60766	YES	YES
30	a	416.38	3.11805	YES	YES
31	a	431.45	0.83475	YES	YES
32	a	452.00	0.63243	YES	YES
33	a	467.01	0.40503	YES	YES
34	a	474.57	3.83516	YES	YES
35	a	487.49	12.99325	YES	YES
36	a	493.95	0.21123	YES	YES
37	a	520.07	1.81214	YES	YES
38	a	527.15	5.65477	YES	YES
39	a	533.50	0.52258	YES	YES
40	a	564.99	3.57873	YES	YES
41	a	579.71	0.36755	YES	YES
42	a	600.52	1.20567	YES	YES
43	a	611.17	1.41382	YES	YES
44	a	612.98	25.26251	YES	YES
45	a	621.48	7.53164	YES	YES
46	a	629.58	25.23277	YES	YES
47	a	645.56	30.57528	YES	YES
48	a	688.43	28.37147	YES	YES
49	a	697.66	30.34196	YES	YES
50	a	717.08	11.57712	YES	YES

3ia′

SCF Energy (au) BP86/SV(P)	-2183.136961706
SCF Energy (au) PBE0/def2-TZVPP	-2182.727556389
SCF Energy (au) PBE0/def2-TZVPP	-2182.7350096537 (Toluene Correction)
Zero Point Energy (au)	0.2598973
Chemical Potential (kJ mol^{-1})	537.84
Dispersion Correction (au) PBE0/def2-TZVPP	-0.05187819

xyz coordinates
37

Mn	1.1456731	2.4084287	1.0514434
C	1.2843480	3.0374348	2.7773269
C	2.8931301	2.1414828	0.8561337
C	1.3131216	4.0054815	0.3609113
O	1.3955239	5.0598688	-0.1377030
O	4.0299225	1.9255071	0.7060206
O	1.4135291	3.4230247	3.8688410
C	-1.5463574	1.2579627	1.3745898
C	-3.0483446	1.3691686	1.4009870
C	-0.8895210	-0.0592515	1.6022068
C	0.0720327	-0.5659509	0.6705188
C	0.6462642	-1.8285391	0.9599993
C	0.3247948	-2.5445601	2.1197813
C	-0.6257246	-2.0367553	3.0241373
C	-1.2423982	-0.8112986	2.7495235
H	1.3697638	-2.2486436	0.2426544
H	0.8152544	-3.5120111	2.3177643
H	-0.8882081	-2.5966910	3.9362526
H	-1.9917681	-0.4121785	3.4530209
O	-0.8668938	2.2793093	1.1438564
H	1.1975253	1.7970538	-1.6421925
C	0.9189164	1.4000598	-0.6460373
C	0.4425086	0.1143609	-0.6185621
C	0.3414973	-0.7211797	-1.8562369
H	-3.3556380	2.4322364	1.3398434
H	-3.4756974	0.9006292	2.3143817
H	-3.4705582	0.8113526	0.5333939
C	0.0811686	-2.2377162	-4.2519088
C	-0.8457575	-2.3966710	-3.2071173
C	-0.7134116	-1.6537369	-2.0239658
C	1.2731481	-0.5851976	-2.9146930
C	1.1430398	-1.3293998	-4.0968532
H	-0.0187059	-2.8250778	-5.1797095
H	-1.6839069	-3.1057620	-3.3152054
H	-1.4534085	-1.7869382	-1.2170713
H	2.1270325	0.1014893	-2.7953285
H	1.8881048	-1.2072921	-4.9010036

$vibrational spectrum

# mode	symmetry	wave number	IR intensity	selection rules	
#		cm**(-1)	km/mol	IR	RAMAN
1		0.00	0.00000	-	-
2		0.00	0.00000	-	-
3		0.00	0.00000	-	-
4		0.00	0.00000	-	-
5		0.00	0.00000	-	-
6		0.00	0.00000	-	-
7	a	11.03	0.04299	YES	YES
8	a	29.88	0.03834	YES	YES
9	a	43.82	0.16594	YES	YES
10	a	51.24	0.15359	YES	YES

11	a	61.74	0.30398	YES	YES
12	a	69.23	1.44302	YES	YES
13	a	79.86	0.04878	YES	YES
14	a	85.57	0.38966	YES	YES
15	a	88.81	0.11878	YES	YES
16	a	97.83	0.27936	YES	YES
17	a	106.39	0.50957	YES	YES
18	a	110.87	0.22317	YES	YES
19	a	146.59	2.94774	YES	YES
20	a	157.49	1.94876	YES	YES
21	a	204.75	0.39131	YES	YES
22	a	218.92	2.28317	YES	YES
23	a	231.59	0.37369	YES	YES
24	a	252.43	0.43101	YES	YES
25	a	289.71	0.21352	YES	YES
26	a	309.83	0.54719	YES	YES
27	a	358.37	0.71677	YES	YES
28	a	374.94	1.21258	YES	YES
29	a	404.39	0.02014	YES	YES
30	a	417.18	2.72831	YES	YES
31	a	427.00	1.39593	YES	YES
32	a	445.95	1.88235	YES	YES
33	a	457.11	1.40503	YES	YES
34	a	465.73	7.32504	YES	YES
35	a	486.02	2.33309	YES	YES
36	a	521.44	4.72483	YES	YES
37	a	523.49	5.61477	YES	YES
38	a	527.41	4.31855	YES	YES
39	a	547.92	4.01688	YES	YES
40	a	577.62	2.23764	YES	YES
41	a	598.63	2.96917	YES	YES
42	a	599.31	11.79126	YES	YES
43	a	612.20	1.68466	YES	YES
44	a	614.93	32.37495	YES	YES
45	a	618.76	29.53671	YES	YES
46	a	630.83	8.60057	YES	YES
47	a	657.53	6.90263	YES	YES
48	a	695.80	27.47499	YES	YES
49	a	696.46	8.46114	YES	YES
50	a	704.54	39.55734	YES	YES

3ja

SCF Energy (au) BP86/SV(P)	-2452.103080854
SCF Energy (au) PBE0/def2-TZVPP	-2451.641880652
SCF Energy (au) PBE0/def2-TZVPP	-2451.6499605316 (Toluene Correction)
Zero Point Energy (au)	0.3448731
Chemical Potential (kJ mol^{-1})	740.54
Dispersion Correction (au) PBE0/def2-TZVPP	-0.06861384

xyz coordinates
48

Mn	1.6497721	1.3358800	0.5377851
C	2.2607111	1.1039574	2.2578338
C	3.2884896	1.2193251	-0.1490047
C	1.7869671	3.0934275	0.5737376
O	1.8781603	4.2597576	0.5697864
O	4.3558709	1.1265886	-0.6172802
O	2.6536735	0.9511328	3.3429371
C	-1.0368873	0.4731728	1.4794316
C	-2.3887641	0.7350323	2.0440928
C	-0.4926578	-0.8470109	1.3727270
C	0.4605652	-1.1886026	0.3999303
C	1.0481149	-2.5666603	0.4574499
O	-0.3496116	1.4707664	1.0859053
C	0.6702105	1.4277704	-2.5020987
C	1.2395772	2.6775180	-2.8692160
C	1.0967138	3.1776742	-4.1690384
C	0.3650077	2.4529686	-5.1285574
C	-0.2254533	1.2226328	-4.7807425
C	-0.0754722	0.7160695	-3.4848411
H	1.7947466	3.2520937	-2.1120276
H	1.5514070	4.1454229	-4.4374168
H	0.2445661	2.8532503	-6.1490767
H	-0.8099279	0.6599920	-5.5274730
H	-0.5511774	-0.2390094	-3.2066295
C	0.8194577	0.9109833	-1.1540798
C	0.7721341	-0.4483760	-0.8194785
H	1.1284006	-1.1315150	-1.6198489
H	-0.8315661	-1.6271544	2.0700320
C	2.2169575	-5.1499094	0.5825091
C	2.9898023	-4.0412065	0.1967011
C	2.4100757	-2.7654005	0.1272300
C	0.2826793	-3.6927490	0.8410522
C	0.8598144	-4.9705594	0.8999324
H	2.6713797	-6.1531857	0.6332252
H	4.0574584	-4.1677738	-0.0476822
H	3.0296920	-1.8999209	-0.1607391
H	-0.7895347	-3.5740838	1.0665908
H	0.2402736	-5.8351928	1.1902115
C	-4.9417765	1.3110561	3.1085565
C	-4.5833454	-0.0176796	2.8192199
C	-3.3178408	-0.3043725	2.2870562
C	-2.7625783	2.0690374	2.3354150
C	-4.0287738	2.3534290	2.8623771
H	-5.9378888	1.5353865	3.5250813
H	-5.2993217	-0.8358795	3.0019444
H	-3.0572988	-1.3454709	2.0391041
H	-2.0348341	2.8714214	2.1397768
H	-4.3079682	3.3959661	3.0875984

$vibrational spectrum

# mode	symmetry	wave number cm**(-1)	IR intensity km/mol	selection rules IR	selection rules RAMAN
1		0.00	0.00000	-	-
2		0.00	0.00000	-	-
3		0.00	0.00000	-	-
4		0.00	0.00000	-	-
5		0.00	0.00000	-	-
6		0.00	0.00000	-	-
7	a	14.61	0.38846	YES	YES
8	a	22.13	1.40826	YES	YES
9	a	29.62	0.58524	YES	YES
10	a	35.83	0.11973	YES	YES
11	a	37.15	1.03095	YES	YES
12	a	45.28	0.39974	YES	YES
13	a	51.69	0.80298	YES	YES
14	a	53.97	0.16678	YES	YES
15	a	59.96	0.19192	YES	YES
16	a	83.78	0.20446	YES	YES
17	a	88.78	0.11842	YES	YES
18	a	94.65	0.05377	YES	YES
19	a	108.34	1.43645	YES	YES
20	a	112.52	0.66592	YES	YES
21	a	121.74	2.50328	YES	YES
22	a	137.87	2.51867	YES	YES
23	a	154.04	1.13695	YES	YES
24	a	159.72	19.21260	YES	YES
25	a	208.77	5.50424	YES	YES
26	a	223.45	5.51475	YES	YES
27	a	236.61	6.29007	YES	YES
28	a	244.48	0.51186	YES	YES
29	a	263.63	0.36410	YES	YES
30	a	300.33	15.23751	YES	YES
31	a	315.45	2.35486	YES	YES
32	a	354.74	4.40350	YES	YES
33	a	399.79	0.34017	YES	YES
34	a	401.89	0.37964	YES	YES
35	a	404.00	0.99737	YES	YES
36	a	410.72	1.43291	YES	YES
37	a	432.02	13.66021	YES	YES
38	a	444.42	0.94919	YES	YES
39	a	449.20	0.88613	YES	YES
40	a	469.99	3.80862	YES	YES
41	a	476.01	1.14658	YES	YES
42	a	490.75	5.89549	YES	YES
43	a	511.19	3.28314	YES	YES
44	a	523.69	2.96840	YES	YES
45	a	532.69	13.34996	YES	YES
46	a	537.60	6.45739	YES	YES
47	a	571.53	10.37027	YES	YES
48	a	595.22	21.30454	YES	YES
49	a	600.42	1.13653	YES	YES
50	a	609.38	0.96738	YES	YES

8ia

SCF Energy (au) BP86/SV(P)	-2183.167100726
SCF Energy (au) PBE0/def2-TZVPP	-2182.760093970
SCF Energy (au) PBE0/def2-TZVPP	-2182.7673435546 (Toluene Correction)
Zero Point Energy (au)	0.262048
Chemical Potential (kJ mol^{-1})	552.88
Dispersion Correction (au) PBE0/def2-TZVPP	-0.05528328

xyz coordinates
37

C	0.5519872	-2.6912867	-1.2913389
C	0.2840276	-0.7130500	-2.8426551
C	-0.3152585	0.3674579	-0.0427651
C	2.6292711	-1.3094929	-1.9255555
C	-1.1180588	-1.5613016	1.0385935
C	-1.8861600	-2.6710730	1.4213656
C	-0.5344483	-2.8386193	3.4524914
C	0.2436599	-1.7263580	3.0576751
C	-0.0589658	-1.0903227	1.8545506
C	0.6506036	0.0699627	1.1437708
C	1.0179321	1.2176860	2.0832109
Mn	1.0064560	-0.9825830	-1.2152724
O	1.7826055	-0.4273318	0.4228709
O	0.2895606	-3.8286872	-1.2814776
O	-0.1846590	-0.4983389	-3.8894827
C	-1.1895038	-0.7156615	-0.1640049
O	3.6722692	-1.5186156	-2.3927036
C	-1.5874721	-3.3030468	2.6447767
H	-1.9587835	-0.8286218	-0.9438598
C	-0.2589714	1.5834644	-0.8799974
C	-0.2088766	3.9319931	-2.4783775
C	0.9676993	3.4679743	-1.8653119
C	0.9489654	2.3093202	-1.0758683
C	-1.4363562	2.0636611	-1.5112907
C	-1.4104639	3.2233530	-2.2967212
H	-0.1902141	4.8431914	-3.0984616
H	1.9172306	4.0091726	-2.0092557
H	1.8825905	1.9313140	-0.6341402
H	-2.3891518	1.5325110	-1.3564038
H	-2.3404965	3.5823853	-2.7675948
H	1.0743472	-1.3783058	3.6934000
H	-0.3103288	-3.3497958	4.4034055
H	-2.1830567	-4.1722517	2.9692037
H	-2.7093477	-3.0381332	0.7864306
H	1.4649868	2.0723840	1.5358923
H	1.7650006	0.8569529	2.8231957
H	0.1213801	1.5800935	2.6317052

$vibrational spectrum

# mode	symmetry	wave number	IR intensity	selection rules	
#		cm**(-1)	km/mol	IR	RAMAN
1		0.00	0.00000	-	-
2		0.00	0.00000	-	-
3		0.00	0.00000	-	-
4		0.00	0.00000	-	-
5		0.00	0.00000	-	-
6		0.00	0.00000	-	-
7	a	20.11	0.20072	YES	YES
8	a	43.70	0.18262	YES	YES
9	a	55.91	0.39403	YES	YES
10	a	72.01	0.02060	YES	YES

11	a	78.66	0.27668	YES	YES
12	a	86.20	0.40743	YES	YES
13	a	91.56	0.15210	YES	YES
14	a	99.13	0.43175	YES	YES
15	a	106.62	0.83380	YES	YES
16	a	108.75	0.38617	YES	YES
17	a	124.81	0.80891	YES	YES
18	a	173.83	1.02082	YES	YES
19	a	187.33	0.44146	YES	YES
20	a	208.12	0.11055	YES	YES
21	a	236.87	0.28880	YES	YES
22	a	250.26	0.29553	YES	YES
23	a	262.29	2.69722	YES	YES
24	a	276.55	0.22091	YES	YES
25	a	311.10	7.63204	YES	YES
26	a	335.74	4.50892	YES	YES
27	a	358.90	3.92761	YES	YES
28	a	409.49	0.48452	YES	YES
29	a	415.74	1.38512	YES	YES
30	a	441.65	6.35928	YES	YES
31	a	449.05	0.51048	YES	YES
32	a	464.02	4.18320	YES	YES
33	a	477.83	10.23579	YES	YES
34	a	484.00	5.78995	YES	YES
35	a	503.35	9.08164	YES	YES
36	a	521.28	8.94887	YES	YES
37	a	532.86	1.55631	YES	YES
38	a	556.82	8.49744	YES	YES
39	a	560.73	5.82532	YES	YES
40	a	590.10	10.15338	YES	YES
41	a	599.96	27.22976	YES	YES
42	a	607.28	14.07331	YES	YES
43	a	610.71	3.00172	YES	YES
44	a	638.06	18.14658	YES	YES
45	a	648.93	20.96493	YES	YES
46	a	670.45	18.26347	YES	YES
47	a	692.11	31.38006	YES	YES
48	a	697.96	21.35305	YES	YES
49	a	704.56	12.89033	YES	YES
50	a	746.65	33.45622	YES	YES

8ja

```
SCF Energy (au) BP86/SV(P)                    -2452.112960024
SCF Energy (au) PBE0/def2-TZVPP               -2451.661992594
SCF Energy (au) PBE0/def2-TZVPP               -2451.6706401152 (Toluene Correction)
Zero Point Energy (au)                        0.3460163
Chemical Potential (kJ mol-1)                 747.33
Dispersion Correction (au) PBE0/def2-TZVPP   -0.07305111

xyz coordinates
48

C     0.8108323   -2.5903869   -1.8551545
C     0.3540829   -0.6479745   -3.4094316
C    -0.0826625    0.4169862   -0.5404790
C     2.7762166   -1.1195163   -2.6223677
C    -0.8478166   -1.4866598    0.6328914
C    -1.7366531   -2.6171487    0.9565024
C     0.2389347   -1.0390112    1.3320947
C     0.9318723    0.0981200    0.5907915
C     1.3462114    1.2268378    1.5268983
Mn    1.1834611   -0.8643311   -1.8247111
O     2.0238398   -0.4190367   -0.1702391
O     0.6133847   -3.7407970   -1.8329725
O    -0.1845942   -0.4812006   -4.4314630
C    -0.9733815   -0.6514769   -0.5916826
O     3.8052897   -1.2837979   -3.1362321
H    -1.7905066   -0.7560741   -1.3207365
C    -0.0049395    1.6024817   -1.4098848
C     0.1173724    3.9102760   -3.0585552
C     1.2408968    3.5139527   -2.3143373
C     1.1863709    2.3767047   -1.4952047
C    -1.1333642    2.0200555   -2.1687061
C    -1.0715674    3.1590736   -2.9776495
H     0.1631612    4.8064150   -3.6988284
H     2.1747461    4.0968736   -2.3679992
H     2.0717122    2.0692665   -0.9211398
H    -2.0760080    1.4540942   -2.0993076
H    -1.9618472    3.4706015   -3.5482432
C    -3.4330047   -4.8051935    1.5919634
C    -2.6637013   -4.1884179    2.5962573
C    -1.8294188   -3.1077696    2.2832143
C    -2.5224466   -3.2442275   -0.0407170
C    -3.3575429   -4.3279362    0.2732583
H    -4.0918794   -5.6539004    1.8389797
H    -2.7208029   -4.5500213    3.6363777
H    -1.2500667   -2.6188642    3.0831149
H    -2.4615598   -2.9022017   -1.0859113
H    -3.9514708   -4.8057075   -0.5233034
C     2.1262996    3.2434620    3.3417805
C     3.0871860    2.3796812    2.7891985
C     2.7002511    1.3750198    1.8854352
C     0.3845893    2.0930841    2.0859939
C     0.7729370    3.0974656    2.9870120
H     2.4308740    4.0329286    4.0488730
H     4.1503046    2.4888908    3.0619363
H     3.4429536    0.6954052    1.4389028
H    -0.6785673    1.9830107    1.8118488
H     0.0129662    3.7725691    3.4145818
H     0.6770557   -1.4816048    2.2373501
```

```
$vibrational spectrum
#  mode     symmetry     wave number   IR intensity    selection rules
#                         cm**(-1)        km/mol         IR     RAMAN
     1                      0.00          0.00000         -       -
     2                      0.00          0.00000         -       -
     3                      0.00          0.00000         -       -
     4                      0.00          0.00000         -       -
     5                      0.00          0.00000         -       -
     6                      0.00          0.00000         -       -
     7        a             9.39          0.51363        YES     YES
     8        a            32.12          0.20469        YES     YES
     9        a            34.81          0.10117        YES     YES
    10        a            37.44          0.46825        YES     YES
    11        a            40.24          0.01493        YES     YES
    12        a            45.91          0.16691        YES     YES
    13        a            51.66          0.12131        YES     YES
    14        a            67.30          0.24290        YES     YES
    15        a            73.61          0.08827        YES     YES
    16        a            82.74          0.23275        YES     YES
    17        a            90.31          0.58714        YES     YES
    18        a            96.41          0.25560        YES     YES
    19        a           106.21          1.03310        YES     YES
    20        a           122.44          0.07124        YES     YES
    21        a           135.04          0.29045        YES     YES
    22        a           159.90          0.70650        YES     YES
    23        a           171.85          2.70924        YES     YES
    24        a           202.85          1.28940        YES     YES
    25        a           216.36          0.94684        YES     YES
    26        a           226.97          1.21257        YES     YES
    27        a           240.13          3.80695        YES     YES
    28        a           258.11          1.61148        YES     YES
    29        a           265.91          0.05346        YES     YES
    30        a           335.01          4.01898        YES     YES
    31        a           383.95          0.80238        YES     YES
    32        a           401.10          0.41059        YES     YES
    33        a           401.14          0.52167        YES     YES
    34        a           404.62          0.48174        YES     YES
    35        a           412.48          2.77429        YES     YES
    36        a           420.42          1.67771        YES     YES
    37        a           443.53          7.46887        YES     YES
    38        a           450.80          1.71787        YES     YES
    39        a           466.08         11.60292        YES     YES
    40        a           472.43          5.63197        YES     YES
    41        a           480.54          4.86212        YES     YES
    42        a           497.71         15.38443        YES     YES
    43        a           512.43         20.50597        YES     YES
    44        a           531.11          2.32273        YES     YES
    45        a           542.35         12.22692        YES     YES
    46        a           563.25          9.03231        YES     YES
    47        a           599.68         11.71928        YES     YES
    48        a           604.55         11.06808        YES     YES
    49        a           609.38          3.49552        YES     YES
    50        a           610.81          2.20605        YES     YES
```

9ia

SCF Energy (au) BP86/SV(P)	-2183.138380453
SCF Energy (au) PBE0/def2-TZVPP	-2182.724742562
SCF Energy (au) PBE0/def2-TZVPP	-2182.7310254792 (Toluene Correction)
Zero Point Energy (au)	0.2609739
Chemical Potential (kJ mol^{-1})	548.90
Dispersion Correction (au) PBE0/def2-TZVPP	-0.05007606

xyz coordinates
37

C	-0.4555222	-1.0272451	3.0134137
C	-2.2322748	0.4111283	1.9710883
C	0.0765045	0.6592913	0.2153566
C	-2.0328828	-2.2131014	1.3040033
C	0.6943673	-1.7017693	0.2059772
C	1.4316334	-2.8323304	0.7623819
C	1.3005899	-4.0080826	-1.3831930
C	0.5988639	-2.9877527	-1.9733225
C	0.2029760	-1.8214566	-1.2085885
C	-0.5734114	-0.7912101	-1.6547933
C	-1.1030889	-0.4177771	-2.9922619
Mn	-0.9860359	-0.7326150	1.3497814
O	-0.9747193	0.1184265	-0.6077278
O	-0.0663219	-1.2180463	4.0996646
O	-3.0346241	1.1523496	2.3770901
C	0.9524247	-0.3565723	0.6718398
O	-2.7078744	-3.1600726	1.2635249
C	1.7089756	-3.9403243	0.0073344
H	1.7758198	-0.1248679	1.3632294
C	0.1894817	2.1233550	0.2802081
C	0.3726487	4.9465356	0.4286199
C	-0.6867555	4.3411891	-0.2687163
C	-0.7816514	2.9441210	-0.3418260
C	1.2592495	2.7430561	0.9719089
C	1.3436830	4.1395897	1.0490001
H	0.4435155	6.0448941	0.4884125
H	-1.4530753	4.9637220	-0.7593994
H	-1.6178905	2.4722011	-0.8791882
H	2.0349144	2.1291004	1.4564324
H	2.1816553	4.6031950	1.5951619
H	0.2850503	-3.0663822	-3.0270335
H	1.5562552	-4.9047089	-1.9718040
H	2.2602328	-4.7868424	0.4477199
H	1.7740923	-2.7639756	1.8084347
H	-0.8191214	-1.1743746	-3.7523658
H	-0.7027317	0.5686405	-3.3272306
H	-2.2149523	-0.3312879	-2.9831333

$vibrational spectrum

# mode	symmetry	wave number	IR intensity	selection rules	
#		cm**(-1)	km/mol	IR	RAMAN
1		0.00	0.00000	-	-
2		0.00	0.00000	-	-
3		0.00	0.00000	-	-
4		0.00	0.00000	-	-
5		0.00	0.00000	-	-
6		0.00	0.00000	-	-
7	a	32.09	0.16433	YES	YES
8	a	41.04	0.43457	YES	YES
9	a	43.30	0.08549	YES	YES
10	a	61.32	0.14316	YES	YES

11	a	70.63	0.24433	YES	YES
12	a	80.44	0.10310	YES	YES
13	a	88.72	0.56643	YES	YES
14	a	91.28	0.09064	YES	YES
15	a	101.03	0.17071	YES	YES
16	a	122.69	0.88529	YES	YES
17	a	137.08	0.61744	YES	YES
18	a	154.22	0.07845	YES	YES
19	a	160.24	2.97304	YES	YES
20	a	194.72	3.09354	YES	YES
21	a	210.73	1.39781	YES	YES
22	a	225.33	0.10436	YES	YES
23	a	264.83	0.46515	YES	YES
24	a	279.37	0.16220	YES	YES
25	a	328.31	2.31572	YES	YES
26	a	395.22	2.39126	YES	YES
27	a	401.33	1.58970	YES	YES
28	a	416.82	3.66140	YES	YES
29	a	423.99	7.52465	YES	YES
30	a	435.17	1.33824	YES	YES
31	a	461.94	5.83288	YES	YES
32	a	464.69	8.61675	YES	YES
33	a	473.03	2.00337	YES	YES
34	a	483.39	4.12031	YES	YES
35	a	485.54	1.74401	YES	YES
36	a	499.73	10.17819	YES	YES
37	a	505.07	2.18928	YES	YES
38	a	525.63	10.68491	YES	YES
39	a	528.37	6.68453	YES	YES
40	a	539.64	8.83679	YES	YES
41	a	569.83	17.33705	YES	YES
42	a	606.92	22.91671	YES	YES
43	a	609.38	19.50509	YES	YES
44	a	615.44	28.65066	YES	YES
45	a	639.94	47.13741	YES	YES
46	a	648.67	22.80887	YES	YES
47	a	677.37	47.26649	YES	YES
48	a	690.85	40.63817	YES	YES
49	a	700.88	5.60458	YES	YES
50	a	705.66	7.87861	YES	YES

9ja

SCF Energy (au) BP86/SV(P)	-2452.123348709
SCF Energy (au) PBE0/def2-TZVPP	-2451.669527954
SCF Energy (au) PBE0/def2-TZVPP	-2451.6778817812 (Toluene Correction)
Zero Point Energy (au)	0.3465202
Chemical Potential (kJ mol^{-1})	748.52
Dispersion Correction (au) PBE0/def2-TZVPP	-0.06711919

xyz coordinates
48

C	2.3481734	2.2431881	1.1948046
C	0.1669204	2.3188157	2.4292183
C	-0.4497452	1.1219581	-0.1618083
C	1.7887959	0.1560994	2.6481949
C	1.5063152	-0.3478334	-0.2672712
C	2.9328444	-0.6229345	-0.5999385
C	0.5954253	-1.4898840	-0.0218430
C	-0.6543657	-1.2326713	0.4334046
C	-1.8283792	-2.0885233	0.5937571
Mn	0.9803039	1.1277700	1.3506333
O	-0.8371988	0.1422389	0.8244142
O	3.2417433	2.9837327	1.0599128
O	-0.3559810	3.0919240	3.1280260
C	0.8256757	0.8453429	-0.7168583
O	2.3032906	-0.4872383	3.4703845
H	1.2711844	1.5624460	-1.4211007
C	-1.4756507	2.0835584	-0.5975933
C	-4.0855982	-3.7748885	0.9452271
C	-2.9467578	-4.2450724	0.2634206
C	-1.8353585	-3.4137417	0.0830389
C	-2.9808622	-1.6281341	1.2788312
C	-4.0956897	-2.4630130	1.4475238
H	-4.9620109	-4.4296990	1.0795436
H	-2.9293074	-5.2697910	-0.1430840
H	-0.9636572	-3.7922900	-0.4742328
H	-2.9923089	-0.6067439	1.6857237
H	-4.9814458	-2.0831672	1.9829698
H	0.8987698	-2.5058650	-0.3135795
C	-3.4319440	3.9575317	-1.4261029
C	-2.3158747	3.6942575	-2.2390909
C	-1.3504676	2.7605723	-1.8347964
C	-2.6048486	2.3545139	0.2113516
C	-3.5729147	3.2799432	-0.2017181
H	-4.1933155	4.6868346	-1.7478189
H	-2.2010025	4.2109463	-3.2061260
H	-0.4983866	2.5433499	-2.4980973
H	-2.7102842	1.8425986	1.1795225
H	-4.4441656	3.4791372	0.4434947
C	5.6523835	-1.2186915	-1.2127869
C	5.0527270	-0.0436213	-1.6970863
C	3.7148678	0.2504861	-1.3942853
C	3.5578032	-1.7978579	-0.1128142
C	4.8955628	-2.0926406	-0.4142897
H	6.7043340	-1.4473057	-1.4498788
H	5.6332163	0.6573574	-2.3196217
H	3.2815708	1.1826469	-1.7890620
H	2.9934402	-2.4831364	0.5401539
H	5.3521732	-3.0125058	-0.0126667

```
$vibrational spectrum
#  mode     symmetry     wave number   IR intensity    selection rules
#                         cm**(-1)        km/mol         IR     RAMAN
     1                      0.00         0.00000         -        -
     2                      0.00         0.00000         -        -
     3                      0.00         0.00000         -        -
     4                      0.00         0.00000         -        -
     5                      0.00         0.00000         -        -
     6                      0.00         0.00000         -        -
     7         a           10.18         0.33573        YES      YES
     8         a           25.47         0.04866        YES      YES
     9         a           28.98         0.47989        YES      YES
    10         a           37.98         0.33424        YES      YES
    11         a           40.14         0.15511        YES      YES
    12         a           43.28         0.33799        YES      YES
    13         a           47.51         0.07398        YES      YES
    14         a           64.19         0.02483        YES      YES
    15         a           65.43         0.04008        YES      YES
    16         a           85.18         0.26396        YES      YES
    17         a           86.74         0.47070        YES      YES
    18         a           99.55         0.10688        YES      YES
    19         a          112.34         0.21166        YES      YES
    20         a          133.07         0.22799        YES      YES
    21         a          156.64         0.23988        YES      YES
    22         a          176.24         0.31802        YES      YES
    23         a          178.08         5.06385        YES      YES
    24         a          201.08         1.21054        YES      YES
    25         a          224.13         0.38223        YES      YES
    26         a          231.65         0.53260        YES      YES
    27         a          253.66         0.13913        YES      YES
    28         a          283.75         0.39827        YES      YES
    29         a          339.79         1.84763        YES      YES
    30         a          370.05         0.66602        YES      YES
    31         a          397.59         0.44885        YES      YES
    32         a          399.17         1.01817        YES      YES
    33         a          400.76         0.01344        YES      YES
    34         a          409.24         6.36317        YES      YES
    35         a          419.18         9.63843        YES      YES
    36         a          429.06         3.29580        YES      YES
    37         a          446.88         0.94191        YES      YES
    38         a          454.75         2.00890        YES      YES
    39         a          471.78         6.65950        YES      YES
    40         a          475.15         4.60871        YES      YES
    41         a          491.20         5.83447        YES      YES
    42         a          497.32         7.85839        YES      YES
    43         a          505.28        16.47287        YES      YES
    44         a          529.69        11.38708        YES      YES
    45         a          542.19         1.29025        YES      YES
    46         a          556.07         1.17116        YES      YES
    47         a          577.70        32.51701        YES      YES
    48         a          594.52        11.60200        YES      YES
    49         a          610.95         9.31423        YES      YES
    50         a          611.61        15.19755        YES      YES
```

10ia

SCF Energy (au) BP86/SV(P)	-2183.171667761
SCF Energy (au) PBE0/def2-TZVPP	-2182.759271019
SCF Energy (au) PBE0/def2-TZVPP	-2182.7662187626 (Toluene Correction)
Zero Point Energy (au)	0.2623685
Chemical Potential (kJ mol^{-1})	552.90
Dispersion Correction (au) PBE0/def2-TZVPP	-0.05036288

xyz coordinates
37

C	1.7416408	-0.3110429	2.6964122
C	-0.8712760	0.4193370	2.4633608
C	-0.1669048	0.7917484	-0.6034849
C	-0.1250551	-2.0260195	2.8690518
C	1.2989200	-1.1605016	-0.1507975
C	2.5785955	-1.7819943	-0.2919384
C	1.5068238	-3.9769796	-0.2672980
C	0.2492646	-3.4050117	-0.1216549
C	0.1094744	-1.9805270	-0.0721054
C	-1.0938021	-1.1981647	0.2068743
Mn	0.2702500	-0.7301946	1.7199400
O	-1.2283806	-0.0104199	-0.6625917
O	2.6724097	-0.0025099	3.3308564
O	-1.6464613	1.1574311	2.9338273
C	1.0636290	0.2867299	-0.1509372
O	-0.3985336	-2.8882566	3.6113264
C	2.6744766	-3.1661645	-0.3522813
H	1.9213850	0.9676776	-0.0641243
C	-0.4078879	2.1686693	-1.0679434
C	-0.9215874	4.8009094	-1.9518210
C	0.4022624	4.3317557	-1.8682576
C	0.6593036	3.0255458	-1.4329481
C	-1.7371538	2.6507631	-1.1584131
C	-1.9892911	3.9564387	-1.5985609
H	-1.1209938	5.8296381	-2.2948752
H	1.2405463	4.9881441	-2.1531103
H	1.6979338	2.6616762	-1.3974042
H	-2.5646823	1.9881558	-0.8651030
H	-3.0271132	4.3225561	-1.6596933
C	-2.4511310	-1.8280817	0.3522992
H	3.4786001	-1.1497834	-0.3651810
H	3.6626138	-3.6431160	-0.4562814
H	1.6052489	-5.0741229	-0.3131083
H	-0.6497154	-4.0382788	-0.0630405
H	-3.1796535	-1.0938172	0.7576642
H	-2.4062060	-2.6792928	1.0632910
H	-2.8475492	-2.2028965	-0.6219486

$vibrational spectrum

# mode	symmetry	wave number	IR intensity	selection rules	
#		cm**(-1)	km/mol	IR	RAMAN
1		0.00	0.00000	-	-
2		0.00	0.00000	-	-
3		0.00	0.00000	-	-
4		0.00	0.00000	-	-
5		0.00	0.00000	-	-
6		0.00	0.00000	-	-
7	a	22.93	2.04808	YES	YES
8	a	35.63	0.05486	YES	YES
9	a	37.64	0.06628	YES	YES
10	a	67.71	1.08211	YES	YES

11	a	79.74	0.19435	YES	YES
12	a	83.10	0.33410	YES	YES
13	a	93.34	0.53032	YES	YES
14	a	98.58	0.34548	YES	YES
15	a	106.97	0.20771	YES	YES
16	a	132.40	2.84990	YES	YES
17	a	140.23	0.70162	YES	YES
18	a	150.70	0.84470	YES	YES
19	a	181.14	0.41538	YES	YES
20	a	207.09	0.50058	YES	YES
21	a	231.63	5.33756	YES	YES
22	a	262.88	0.13112	YES	YES
23	a	285.43	8.88123	YES	YES
24	a	299.28	0.21116	YES	YES
25	a	318.43	1.84098	YES	YES
26	a	327.54	4.75623	YES	YES
27	a	399.85	0.04054	YES	YES
28	a	416.07	4.37544	YES	YES
29	a	430.70	0.97133	YES	YES
30	a	433.74	7.94172	YES	YES
31	a	468.24	4.45534	YES	YES
32	a	469.79	6.79562	YES	YES
33	a	474.59	0.46909	YES	YES
34	a	489.99	3.20937	YES	YES
35	a	492.12	0.24457	YES	YES
36	a	506.72	8.96440	YES	YES
37	a	515.59	12.62066	YES	YES
38	a	527.61	7.60533	YES	YES
39	a	531.09	6.41458	YES	YES
40	a	559.16	2.49141	YES	YES
41	a	589.33	2.07303	YES	YES
42	a	607.23	29.54767	YES	YES
43	a	610.28	3.70786	YES	YES
44	a	639.55	48.54657	YES	YES
45	a	656.40	12.12776	YES	YES
46	a	660.05	24.40755	YES	YES
47	a	677.96	60.85678	YES	YES
48	a	686.84	36.23974	YES	YES
49	a	713.28	1.40244	YES	YES
50	a	728.90	69.19622	YES	YES

10ja

SCF Energy (au) BP86/SV(P)	-2452.145470468	
SCF Energy (au) PBE0/def2-TZVPP	-2451.692028912	
SCF Energy (au) PBE0/def2-TZVPP	-2451.7000699796	(Toluene Correction)
Zero Point Energy (au)	0.3474597	
Chemical Potential (kJ mol^{-1})	755.51	
Dispersion Correction (au) PBE0/def2-TZVPP	-0.06824413	

xyz coordinates
48

C	1.6780743	1.2309898	1.9923960
C	-0.9898958	1.2615352	1.9040674
C	-0.0394979	1.1257232	-0.7683092
C	0.4310896	-0.8285674	2.7266545
C	1.3244149	-0.8822765	-0.2962105
C	0.0619891	-1.4838559	0.0472746
C	-1.1331050	-0.7307102	-0.1021554
Mn	0.3600532	0.2340567	1.2849380
O	-1.1312380	0.2895431	-1.0807702
O	2.5330402	1.8902683	2.4346122
O	-1.8603645	1.9235846	2.3016812
C	1.2333480	0.5092709	-0.6593866
O	0.4648208	-1.5390475	3.6535217
H	2.1524689	1.1048530	-0.7607071
C	-0.2579604	2.5575621	-1.0564700
C	-0.6966560	5.2882393	-1.6795272
C	0.5287824	4.8748054	-1.1231468
C	0.7465173	3.5273047	-0.8115288
C	-1.4886779	2.9853354	-1.6077039
C	-1.7022905	4.3375931	-1.9170142
H	-0.8671356	6.3509757	-1.9180002
H	1.3210578	5.6136587	-0.9187918
H	1.7011818	3.2340919	-0.3462086
H	-2.2783306	2.2436561	-1.7939022
H	-2.6684209	4.6501937	-2.3464741
C	-2.4754089	-1.2349964	0.2527769
C	2.6058982	-1.6329494	-0.3207534
H	0.0287655	-2.4999361	0.4664834
C	-5.0553649	-2.2494116	0.8431010
C	-3.9315986	-2.7098572	1.5552067
C	-2.6564172	-2.2072815	1.2680186
C	-3.6124354	-0.7720371	-0.4502200
C	-4.8886191	-1.2782083	-0.1573178
H	-6.0586393	-2.6424943	1.0759038
H	-4.0521713	-3.4610638	2.3530786
H	-1.7964039	-2.5563690	1.8618409
H	-3.4834345	-0.0170514	-1.2389274
H	-5.7620081	-0.9074773	-0.7189706
C	5.0466716	-3.0764760	-0.4370671
C	4.8041327	-2.0249083	-1.3355181
C	3.5947439	-1.3125070	-1.2801693
C	2.8643005	-2.6954262	0.5781829
C	4.0694764	-3.4087702	0.5196244
H	5.9948251	-3.6371283	-0.4799934
H	5.5581945	-1.7596333	-2.0948183
H	3.4034500	-0.5111931	-2.0121849
H	2.1258729	-2.9477113	1.3563622
H	4.2529047	-4.2258964	1.2365219

$vibrational spectrum

# mode	symmetry	wave number cm**(-1)	IR intensity km/mol	selection rules IR	RAMAN
1		0.00	0.00000	-	-
2		0.00	0.00000	-	-
3		0.00	0.00000	-	-
4		0.00	0.00000	-	-
5		0.00	0.00000	-	-
6		0.00	0.00000	-	-
7	a	23.29	0.10653	YES	YES
8	a	35.24	0.53647	YES	YES
9	a	38.30	0.24444	YES	YES
10	a	41.19	0.96698	YES	YES
11	a	44.55	0.52232	YES	YES
12	a	48.02	0.54913	YES	YES
13	a	49.84	0.29528	YES	YES
14	a	59.13	0.39726	YES	YES
15	a	66.22	0.40964	YES	YES
16	a	86.93	0.62262	YES	YES
17	a	89.10	0.71655	YES	YES
18	a	103.42	0.07471	YES	YES
19	a	126.51	0.15680	YES	YES
20	a	130.66	0.25646	YES	YES
21	a	147.29	7.98522	YES	YES
22	a	172.29	10.05456	YES	YES
23	a	180.03	7.32537	YES	YES
24	a	210.81	4.81705	YES	YES
25	a	214.90	2.33947	YES	YES
26	a	245.05	2.54091	YES	YES
27	a	256.36	1.31831	YES	YES
28	a	296.20	2.60722	YES	YES
29	a	343.61	21.91165	YES	YES
30	a	369.80	7.55395	YES	YES
31	a	381.75	7.18937	YES	YES
32	a	399.35	0.30002	YES	YES
33	a	401.85	0.56329	YES	YES
34	a	402.76	0.87591	YES	YES
35	a	409.32	7.61748	YES	YES
36	a	428.58	6.12174	YES	YES
37	a	443.00	0.49891	YES	YES
38	a	466.45	8.99863	YES	YES
39	a	473.38	2.05015	YES	YES
40	a	486.00	0.75574	YES	YES
41	a	488.36	1.35608	YES	YES
42	a	503.69	5.89648	YES	YES
43	a	515.51	29.07783	YES	YES
44	a	522.48	28.37979	YES	YES
45	a	544.90	4.36957	YES	YES
46	a	555.70	32.56046	YES	YES
47	a	563.22	1.88397	YES	YES
48	a	594.11	46.25290	YES	YES
49	a	608.59	1.40299	YES	YES
50	a	610.70	4.43086	YES	YES

TS_{23aa}

```
SCF Energy (au) BP86/SV(P)                     -2427.864609133
SCF Energy (au) PBE0/def2-TZVPP                -2427.423479953
SCF Energy (au) PBE0/def2-TZVPP                -2427.4336311029 (Toluene Correction)
Zero Point Energy (au)                         0.2961349
Chemical Potential (kJ mol-1)                  626.52
Dispersion Correction (au) PBE0/def2-TZVPP -0.06311849

xyz coordinates
43

Mn    1.6788083    0.5413038    0.6660746
C     2.0503776    0.5296482    2.4309562
C     3.4045443    0.1742659    0.2830201
C     2.0529433    2.3065225    0.5143330
O     2.3294949    3.4417091    0.4733818
O     4.5278610    0.0167896    0.0295371
O     2.2814247    0.4988557    3.5712309
C    -1.0926686   -0.2583324    1.3776519
C    -0.9973400    2.0490016    0.9698394
C    -2.3651450    2.2050745    1.2189961
C    -3.1234895    1.0695381    1.5546272
C    -2.4812563   -0.1687169    1.6297222
C    -0.3111906   -1.4899509    1.4194569
C     1.0201526   -1.3705871    0.9948310
C     1.8629472   -2.5728500    1.0052334
O     1.2773948   -3.7544008    1.5323100
C    -0.0161149   -3.8397296    1.9367080
C    -0.8418725   -2.7488364    1.8724334
O     2.9976265   -2.6790794    0.5829983
C    -0.3753289   -5.2043941    2.4389469
N    -0.3659046    0.8571799    1.0459106
C     0.3346785    1.3859714   -2.3672480
C     0.7595590    2.7269160   -2.5583839
C     0.2076688    3.5125802   -3.5802728
C    -0.7871582    2.9913869   -4.4262850
C    -1.2257909    1.6668207   -4.2433593
C    -0.6763379    0.8725536   -3.2295837
C     0.8900099    0.5081948   -1.3681614
C     1.2206952   -0.6756461   -0.9953258
H    -0.3749161    2.9150525    0.6987359
H    -2.8212774    3.2044537    1.1470848
H    -4.2045597    1.1511865    1.7526742
H    -3.0496886   -1.0765220    1.8787698
H    -1.8814572   -2.8487494    2.2152355
H     1.5379814    3.1497117   -1.9072265
H     0.5606496    4.5483909   -3.7161426
H    -1.2210490    3.6149772   -5.2253032
H    -2.0054478    1.2462192   -4.9007374
H    -1.0214133   -0.1641280   -3.0867825
H     1.6181458   -1.6329900   -1.3457148
H    -1.4359054   -5.2432596    2.7599516
H     0.2713780   -5.4850809    3.3010068
H    -0.2090293   -5.9710504    1.6488693

$vibrational spectrum
#  mode     symmetry     wave number   IR intensity    selection rules
#                         cm**(-1)        km/mol         IR     RAMAN
     1         a          -172.74        0.00000        YES     YES
     2                       0.00        0.00000         -       -
     3                       0.00        0.00000         -       -
     4                       0.00        0.00000         -       -
     5                       0.00        0.00000         -       -
```

6		0.00	0.00000	-	-
7		0.00	0.00000	-	-
8	a	17.15	0.19902	YES	YES
9	a	25.26	0.83606	YES	YES
10	a	36.60	0.25361	YES	YES
11	a	50.78	1.18797	YES	YES
12	a	68.90	0.33269	YES	YES
13	a	75.37	0.27528	YES	YES
14	a	83.79	0.33993	YES	YES
15	a	86.74	0.04327	YES	YES
16	a	96.62	0.57822	YES	YES
17	a	109.43	0.42627	YES	YES
18	a	114.10	0.77591	YES	YES
19	a	125.09	0.29870	YES	YES
20	a	139.91	0.23751	YES	YES
21	a	147.81	0.17597	YES	YES
22	a	157.72	0.40932	YES	YES
23	a	160.75	0.93429	YES	YES
24	a	166.63	0.17092	YES	YES
25	a	197.22	0.23595	YES	YES
26	a	218.08	0.19371	YES	YES
27	a	240.38	0.46477	YES	YES
28	a	253.84	0.64059	YES	YES
29	a	263.01	0.61563	YES	YES
30	a	296.72	2.08692	YES	YES
31	a	326.39	1.40105	YES	YES
32	a	358.07	7.40260	YES	YES
33	a	400.87	3.17185	YES	YES
34	a	401.88	0.60941	YES	YES
35	a	413.51	2.56676	YES	YES
36	a	428.75	2.81648	YES	YES
37	a	445.21	21.76269	YES	YES
38	a	452.26	1.83643	YES	YES
39	a	464.49	7.14943	YES	YES
40	a	476.70	0.11278	YES	YES
41	a	488.43	2.37409	YES	YES
42	a	500.20	9.44866	YES	YES
43	a	518.27	3.03466	YES	YES
44	a	529.63	23.82817	YES	YES
45	a	541.63	1.06373	YES	YES
46	a	544.47	13.48474	YES	YES
47	a	549.76	9.80746	YES	YES
48	a	557.70	1.89472	YES	YES
49	a	578.36	4.12900	YES	YES
50	a	595.17	0.22690	YES	YES

TS_{23aa}'

```
SCF Energy (au) BP86/SV(P)                     -2427.853649810
SCF Energy (au) PBE0/def2-TZVPP                -2427.411853990
SCF Energy (au) PBE0/def2-TZVPP                -2427.4224059121 (Toluene Correction)
Zero Point Energy (au)                         0.2956693
Chemical Potential (kJ mol-1)                  626.31
Dispersion Correction (au) PBE0/def2-TZVPP    -0.06517125

xyz coordinates
43

Mn    1.0604984    1.9929986    0.6823022
C     1.1926923    1.9710117    2.4887800
C     2.8402352    1.7610039    0.5308806
C     1.3031491    3.7726719    0.5343487
O     1.4575616    4.9226638    0.4009624
O     3.9979345    1.6996050    0.4251379
O     1.2638844    1.9542238    3.6513366
C    -1.7427753    0.9781922    0.9238369
C    -1.7520834    3.3143000    0.7426142
C    -3.1498270    3.3684228    0.7892244
C    -3.8676255    2.1628212    0.8860943
C    -3.1576459    0.9614400    0.9491014
C    -0.8901215   -0.2022918    1.0187510
C     0.4721886    0.0135517    0.7444250
C     1.4114444   -1.0994791    0.9731314
O     0.8391756   -2.3394707    1.3652037
C    -0.4883289   -2.5283980    1.5730719
C    -1.3770827   -1.4975889    1.4105772
O     2.6192985   -1.0748372    0.8711519
C    -0.8132012   -3.9270560    1.9988310
N    -1.0561467    2.1591024    0.8084151
H     0.5555830    2.6948941   -2.1509651
C     0.6288065    1.9886473   -1.3183179
C     0.7477162    0.7162106   -1.1093196
H    -1.1579647    4.2371139    0.6524449
H    -3.6603325    4.3429790    0.7462084
H    -4.9694859    2.1626073    0.9105492
H    -3.6911847    0.0015311    1.0122905
H    -2.4402450   -1.6718158    1.6303188
C     1.1039835   -0.5017176   -1.8602694
H    -1.9022736   -4.0486906    2.1688486
H    -0.2713592   -4.1858413    2.9365708
H    -0.4852060   -4.6580329    1.2257502
C     1.7790484   -2.7511502   -3.4308975
C     0.5012548   -2.6531301   -2.8533448
C     0.1673545   -1.5412404   -2.0635187
C     2.3883301   -0.6098079   -2.4425055
C     2.7209197   -1.7283106   -3.2208056
H     2.0437175   -3.6285309   -4.0436556
H    -0.2420134   -3.4504317   -3.0172470
H    -0.8337747   -1.4639422   -1.6100762
H     3.1263228    0.1883341   -2.2657896
H     3.7275781   -1.8025624   -3.6644467

$vibrational spectrum
#  mode     symmetry     wave number   IR intensity    selection rules
#                         cm**(-1)        km/mol         IR     RAMAN
     1        a           -288.74        0.00000        YES     YES
     2                       0.00        0.00000         -       -
     3                       0.00        0.00000         -       -
     4                       0.00        0.00000         -       -
     5                       0.00        0.00000         -       -
```

6		0.00	0.00000	-	-
7		0.00	0.00000	-	-
8	a	21.46	0.39886	YES	YES
9	a	30.55	0.36529	YES	YES
10	a	45.61	0.14736	YES	YES
11	a	48.26	1.22449	YES	YES
12	a	61.67	1.09460	YES	YES
13	a	78.38	0.44833	YES	YES
14	a	80.46	0.36741	YES	YES
15	a	86.87	0.15774	YES	YES
16	a	87.52	0.03196	YES	YES
17	a	102.02	0.56412	YES	YES
18	a	117.64	0.72443	YES	YES
19	a	128.81	0.28060	YES	YES
20	a	139.44	0.17071	YES	YES
21	a	151.48	0.37273	YES	YES
22	a	154.69	1.64005	YES	YES
23	a	163.17	0.10362	YES	YES
24	a	183.63	4.33893	YES	YES
25	a	193.95	0.97564	YES	YES
26	a	205.28	1.95662	YES	YES
27	a	217.80	0.71034	YES	YES
28	a	241.30	0.10665	YES	YES
29	a	260.18	0.11090	YES	YES
30	a	294.13	2.09385	YES	YES
31	a	318.15	4.59114	YES	YES
32	a	336.13	5.10675	YES	YES
33	a	393.18	3.92944	YES	YES
34	a	401.13	0.22347	YES	YES
35	a	424.46	2.13993	YES	YES
36	a	442.37	0.36676	YES	YES
37	a	455.80	1.51566	YES	YES
38	a	461.35	3.17289	YES	YES
39	a	478.45	0.81725	YES	YES
40	a	486.10	4.10302	YES	YES
41	a	497.65	8.36675	YES	YES
42	a	504.82	14.06983	YES	YES
43	a	519.59	6.15730	YES	YES
44	a	527.76	18.80972	YES	YES
45	a	539.96	1.42932	YES	YES
46	a	550.42	22.84540	YES	YES
47	a	556.16	4.99382	YES	YES
48	a	559.43	1.35728	YES	YES
49	a	577.03	5.35828	YES	YES
50	a	595.77	4.13113	YES	YES

TS_{23ba}

SCF Energy (au) BP86/SV(P)	-2277.508412844	
SCF Energy (au) PBE0/def2-TZVPP	-2277.059214716	
SCF Energy (au) PBE0/def2-TZVPP	-2277.0663116709	(Toluene Correction)
Zero Point Energy (au)	0.2893905	
Chemical Potential (kJ mol^{-1})	617.01	
Dispersion Correction (au) PBE0/def2-TZVPP	-0.06065985	

xyz coordinates
41

Mn	1.5462735	0.3705217	0.7753154
C	1.9074785	0.6310002	2.5277213
C	3.2407096	-0.1119005	0.4629426
C	1.9480722	2.0777093	0.3447596
O	2.2410160	3.1806368	0.0936545
O	4.3448104	-0.4362008	0.2648014
O	2.1292592	0.7906648	3.6591366
C	-1.2537830	-0.2555350	1.5959263
C	-1.0996745	1.9697746	0.8633464
C	-2.4649227	2.1924425	1.0617323
C	-3.2557673	1.1294770	1.5353928
C	-2.6464858	-0.0988281	1.7968796
C	-0.5014315	-1.4808271	1.8581717
C	0.8516553	-1.5011387	1.3910424
C	1.6375491	-2.6423727	1.7084079
C	1.1063609	-3.7167233	2.4268538
C	-0.2425309	-3.7004240	2.8496778
C	-1.0364875	-2.5874485	2.5642042
H	-0.4503635	2.7714576	0.4801914
H	-2.8950271	3.1812287	0.8405819
H	-4.3388895	1.2594921	1.6936461
H	-3.2441232	-0.9503939	2.1538562
H	2.6862936	-2.6887826	1.3693071
H	1.7476473	-4.5817195	2.6667466
H	-0.6612248	-4.5507498	3.4116467
H	-2.0786672	-2.5634039	2.9225327
N	-0.5004788	0.7858067	1.1211501
C	0.3494049	0.6612376	-2.3782613
C	0.9745764	1.8354334	-2.8740594
C	0.5652123	2.4058176	-4.0878125
C	-0.4881425	1.8378149	-4.8263467
C	-1.1277044	0.6820037	-4.3411795
C	-0.7159981	0.0978621	-3.1365469
H	1.7966554	2.2946711	-2.3060925
H	1.0739469	3.3109476	-4.4593641
H	-0.8125999	2.2955425	-5.7753977
H	-1.9568402	0.2282210	-4.9096864
H	-1.2183045	-0.8065564	-2.7569113
C	0.7583943	0.0048373	-1.1586284
C	0.9391618	-1.1406739	-0.5838789
H	1.1481747	-2.1808689	-0.8504819

$vibrational spectrum

# mode	symmetry	wave number	IR intensity	selection rules	
#		cm**(-1)	km/mol	IR	RAMAN
1	a	-230.16	0.00000	YES	YES
2		0.00	0.00000	-	-
3		0.00	0.00000	-	-
4		0.00	0.00000	-	-
5		0.00	0.00000	-	-
6		0.00	0.00000	-	-
7		0.00	0.00000	-	-

8	a	15.33	0.05488	YES	YES
9	a	25.28	0.19576	YES	YES
10	a	45.61	0.05019	YES	YES
11	a	58.66	0.11086	YES	YES
12	a	71.59	0.08026	YES	YES
13	a	82.53	0.01510	YES	YES
14	a	89.55	0.28337	YES	YES
15	a	92.92	1.09647	YES	YES
16	a	100.46	0.46379	YES	YES
17	a	109.98	0.06435	YES	YES
18	a	116.38	0.03637	YES	YES
19	a	132.51	0.35585	YES	YES
20	a	162.15	1.17092	YES	YES
21	a	182.00	0.54087	YES	YES
22	a	187.25	0.55331	YES	YES
23	a	217.10	0.99499	YES	YES
24	a	233.54	0.82810	YES	YES
25	a	259.73	0.63802	YES	YES
26	a	278.83	0.12353	YES	YES
27	a	343.57	8.45214	YES	YES
28	a	359.38	1.36759	YES	YES
29	a	385.36	2.37851	YES	YES
30	a	402.36	0.07350	YES	YES
31	a	415.74	3.16983	YES	YES
32	a	430.74	1.25286	YES	YES
33	a	447.29	0.41322	YES	YES
34	a	453.95	8.13841	YES	YES
35	a	463.51	5.86149	YES	YES
36	a	474.07	1.00384	YES	YES
37	a	488.83	0.36260	YES	YES
38	a	496.72	7.61252	YES	YES
39	a	503.64	5.23803	YES	YES
40	a	522.87	0.37797	YES	YES
41	a	544.04	13.92394	YES	YES
42	a	546.27	13.93147	YES	YES
43	a	553.19	0.61857	YES	YES
44	a	583.04	24.49030	YES	YES
45	a	610.29	10.92190	YES	YES
46	a	614.19	12.97094	YES	YES
47	a	620.33	1.05024	YES	YES
48	a	625.80	33.87210	YES	YES
49	a	639.10	0.33601	YES	YES
50	a	656.26	9.87788	YES	YES

TS_{23ba}'

SCF Energy (au) BP86/SV(P) -2277.498753669
SCF Energy (au) PBE0/def2-TZVPP -2277.050675757
SCF Energy (au) PBE0/def2-TZVPP -2277.0579338980 (Toluene Correction)
Zero Point Energy (au) 0.2891519
Chemical Potential (kJ mol^{-1}) 617.56
Dispersion Correction (au) PBE0/def2-TZVPP -0.06291758

xyz coordinates
41

Mn	1.1693515	1.7942172	0.3501382
C	1.3530807	2.1824100	2.1120978
C	2.8984100	1.3543494	0.2491536
C	1.5418946	3.4606063	-0.2139974
O	1.7908638	4.5248368	-0.6268932
O	4.0275882	1.0587937	0.1914371
O	1.4670734	2.4424552	3.2421069
C	-1.6743451	1.0921558	0.9437799
C	-1.5381688	3.2923065	0.1440870
C	-2.9237216	3.4672754	0.2150309
C	-3.7137545	2.3901122	0.6558466
C	-3.0839075	1.1971132	1.0167486
C	-0.9017448	-0.0900528	1.3304482
C	0.4690251	-0.1133275	0.9201197
C	1.2675083	-1.2000501	1.3628840
C	0.7451521	-2.2018261	2.1868477
C	-0.6142475	-2.1778035	2.5683531
C	-1.4316588	-1.1289586	2.1356710
H	-0.8861808	4.1064682	-0.2083692
H	-3.3692387	4.4305340	-0.0775210
H	-4.8108328	2.4810647	0.7124324
H	-3.6776879	0.3328533	1.3496471
H	2.3232634	-1.2577098	1.0512727
H	1.4009614	-3.0169031	2.5362660
H	-1.0266261	-2.9709426	3.2128557
H	-2.4836241	-1.0931220	2.4637971
N	-0.9209399	2.1431123	0.4957934
H	0.6382497	1.8377141	-2.5520672
C	0.6896772	1.3458523	-1.5751871
C	0.6556402	0.1490567	-1.0778296
C	0.8042723	-1.2237752	-1.5915096
C	1.0787431	-3.7779246	-2.7828737
C	2.0089812	-2.7639563	-3.0681654
C	1.8725870	-1.4960946	-2.4811888
C	-0.1193572	-2.2559564	-1.3035661
C	0.0135775	-3.5173396	-1.9016755
H	1.1842179	-4.7734357	-3.2452973
H	2.8502730	-2.9600918	-3.7535367
H	2.6014809	-0.6992120	-2.6983808
H	-0.9532646	-2.0579155	-0.6121681
H	-0.7225716	-4.3068894	-1.6765880

$vibrational spectrum

# mode	symmetry	wave number	IR intensity	selection rules	
#		cm**(-1)	km/mol	IR	RAMAN
1	a	-281.20	0.00000	YES	YES
2		0.00	0.00000	-	-
3		0.00	0.00000	-	-
4		0.00	0.00000	-	-
5		0.00	0.00000	-	-
6		0.00	0.00000	-	-
7		0.00	0.00000	-	-

8	a	24.93	0.17999	YES	YES
9	a	27.66	0.48622	YES	YES
10	a	45.67	0.02077	YES	YES
11	a	62.41	0.22472	YES	YES
12	a	65.71	0.29527	YES	YES
13	a	78.12	0.03628	YES	YES
14	a	88.38	0.19623	YES	YES
15	a	91.74	0.15481	YES	YES
16	a	100.11	0.55229	YES	YES
17	a	104.64	0.31790	YES	YES
18	a	117.99	0.09532	YES	YES
19	a	140.73	0.44197	YES	YES
20	a	163.87	0.86445	YES	YES
21	a	173.92	2.70349	YES	YES
22	a	194.82	1.83630	YES	YES
23	a	215.19	2.41823	YES	YES
24	a	221.11	2.12687	YES	YES
25	a	248.39	0.85224	YES	YES
26	a	282.00	0.34236	YES	YES
27	a	311.11	3.32284	YES	YES
28	a	357.00	1.24876	YES	YES
29	a	402.97	0.19811	YES	YES
30	a	405.95	4.72270	YES	YES
31	a	412.17	4.75989	YES	YES
32	a	438.81	1.52677	YES	YES
33	a	459.78	0.38930	YES	YES
34	a	464.82	0.37031	YES	YES
35	a	476.22	0.84671	YES	YES
36	a	488.03	1.90597	YES	YES
37	a	495.73	1.86045	YES	YES
38	a	503.95	1.64839	YES	YES
39	a	508.14	20.16433	YES	YES
40	a	530.44	4.19986	YES	YES
41	a	536.50	13.61825	YES	YES
42	a	551.86	3.52941	YES	YES
43	a	558.62	18.82493	YES	YES
44	a	563.39	4.96911	YES	YES
45	a	607.86	39.79700	YES	YES
46	a	613.27	0.66942	YES	YES
47	a	619.75	1.86931	YES	YES
48	a	625.45	41.97016	YES	YES
49	a	639.98	3.75850	YES	YES
50	a	655.90	36.17631	YES	YES

TS_{23bb}
SCF Energy (au) BP86/SV(P) -2505.228414752
SCF Energy (au) PBE0/def2-TZVPP -2504.778820226
SCF Energy (au) PBE0/def2-TZVPP -2504.7885430275 (Toluene Correction)
Zero Point Energy (au) 0.3313323
Chemical Potential (kJ mol^{-1}) 705.79
Dispersion Correction (au) PBE0/def2-TZVPP -0.06493997

xyz coordinates
47

Mn	1.5438696	0.9121769	1.8459137
C	2.0798231	0.9719827	3.5736955
C	3.1974123	0.4569030	1.3335957
C	1.9102005	2.6474432	1.5317027
O	2.1536748	3.7650521	1.2941777
O	4.2787058	0.1493930	1.0206017
O	2.4160219	1.0083613	4.6878982
C	-1.1654630	0.1794923	2.8715540
C	-1.0519913	2.4881328	2.4653612
C	-2.3845969	2.6819223	2.8412507
C	-3.1387447	1.5606537	3.2329710
C	-2.5251532	0.3067525	3.2449353
C	-0.4090617	-1.0728962	2.8813346
C	0.8736495	-1.0406434	2.2486695
C	1.6714458	-2.2132932	2.3163424
C	1.2170098	-3.3654072	2.9657126
C	-0.0642883	-3.3954557	3.5605320
C	-0.8684506	-2.2533241	3.5161742
H	-0.4291977	3.3392628	2.1480412
H	-2.8179453	3.6938075	2.8222620
H	-4.1964514	1.6664260	3.5247371
H	-3.0947039	-0.5874665	3.5376316
H	2.6647491	-2.2217370	1.8373571
H	1.8666377	-4.2555965	3.0141119
H	-0.4225746	-4.3057289	4.0675956
H	-1.8534569	-2.2681725	4.0106361
N	-0.4512635	1.2776419	2.4717770
C	0.3693367	1.7188046	-1.1444068
C	0.6498899	0.8052351	-0.0221054
C	0.7090328	-0.4233841	0.3630955
H	0.6788933	-1.4385500	-0.0426014
O	-0.1688441	1.0330044	-2.3015015
H	-0.4003790	2.4718013	-0.8668055
H	1.2983409	2.2526720	-1.4426764
C	0.7049208	0.7742823	-3.3204458
O	1.8753878	1.1155984	-3.3111180
C	0.0472152	0.0363104	-4.4440085
C	-1.0840302	-1.3458045	-6.6194205
C	-1.8713107	-1.0393509	-5.4947766
C	-1.3103816	-0.3504427	-4.4085130
C	0.8333988	-0.2740593	-5.5746196
C	0.2691938	-0.9621104	-6.6578499
H	-1.5277977	-1.8874805	-7.4714750
H	-2.9315905	-1.3404367	-5.4649314
H	-1.9178860	-0.1074050	-3.5239907
H	1.8897097	0.0374432	-5.5794971
H	0.8870432	-1.2018104	-7.5389247

$vibrational spectrum

# mode	symmetry	wave number	IR intensity	selection rules	
#		cm**(-1)	km/mol	IR	RAMAN
1	a	-244.44	0.00000	YES	YES

2		0.00	0.00000	-	-
3		0.00	0.00000	-	-
4		0.00	0.00000	-	-
5		0.00	0.00000	-	-
6		0.00	0.00000	-	-
7		0.00	0.00000	-	-
8	a	10.09	0.14991	YES	YES
9	a	12.41	0.42334	YES	YES
10	a	19.22	0.07028	YES	YES
11	a	36.42	0.34341	YES	YES
12	a	49.37	0.00449	YES	YES
13	a	53.56	1.03068	YES	YES
14	a	63.81	0.10861	YES	YES
15	a	68.11	0.21311	YES	YES
16	a	81.72	0.07782	YES	YES
17	a	91.80	0.56193	YES	YES
18	a	94.63	0.43159	YES	YES
19	a	101.38	0.53503	YES	YES
20	a	106.77	0.28635	YES	YES
21	a	122.46	0.47390	YES	YES
22	a	144.26	0.30543	YES	YES
23	a	165.36	0.72460	YES	YES
24	a	171.49	0.24320	YES	YES
25	a	185.89	0.55184	YES	YES
26	a	213.01	0.47639	YES	YES
27	a	230.75	3.89429	YES	YES
28	a	239.47	1.28996	YES	YES
29	a	248.79	3.55678	YES	YES
30	a	275.42	0.73147	YES	YES
31	a	280.32	1.12081	YES	YES
32	a	328.93	26.14672	YES	YES
33	a	358.46	1.92623	YES	YES
34	a	362.92	3.07572	YES	YES
35	a	404.07	0.86355	YES	YES
36	a	404.41	2.01337	YES	YES
37	a	417.15	1.67101	YES	YES
38	a	431.57	5.04544	YES	YES
39	a	439.66	0.34171	YES	YES
40	a	446.69	1.08182	YES	YES
41	a	461.13	0.75367	YES	YES
42	a	471.59	3.36562	YES	YES
43	a	483.05	4.85382	YES	YES
44	a	487.66	0.99726	YES	YES
45	a	495.49	1.19401	YES	YES
46	a	503.25	1.96319	YES	YES
47	a	538.01	10.91463	YES	YES
48	a	541.54	18.16062	YES	YES
49	a	555.44	1.04452	YES	YES
50	a	608.16	29.89436	YES	YES

TS_{23bb}'

```
SCF Energy (au) BP86/SV(P)                  -2505.224054475
SCF Energy (au) PBE0/def2-TZVPP             -2504.774662695
SCF Energy (au) PBE0/def2-TZVPP             -2504.7841905779 (Toluene Correction)
Zero Point Energy (au)                      0.3314439
Chemical Potential (kJ mol-1)               709.37
Dispersion Correction (au) PBE0/def2-TZVPP  -0.06628063

xyz coordinates
47

Mn    1.2176947    1.8127312    1.4456319
C     1.4666299    2.4182820    3.1346110
C     2.8893178    1.1728893    1.4137244
C     1.7679512    3.3323829    0.6551328
O     2.1268865    4.2962021    0.1018704
O     3.9807488    0.7592320    1.4167611
O     1.6187517    2.8163816    4.2189751
C    -1.6838173    1.5142621    2.1055525
C    -1.2983862    3.5883820    1.0814943
C    -2.6544880    3.9279053    1.1212621
C    -3.5615029    3.0007085    1.6654012
C    -3.0715137    1.7883817    2.1557660
C    -1.0522446    0.3000024    2.6278138
C     0.3178775    0.0931789    2.2594279
C     0.9900761   -1.0164614    2.8399414
C     0.3403405   -1.8804578    3.7303729
C    -1.0174117   -1.6817832    4.0587049
C    -1.7050384   -0.5940762    3.5088041
H    -0.5586526    4.2812524    0.6514182
H    -2.9875741    4.8995658    0.7252030
H    -4.6407100    3.2219184    1.7026697
H    -3.7603152    1.0398129    2.5746974
H     2.0494275   -1.1997610    2.5938167
H     0.8985435   -2.7185790    4.1805668
H    -1.5292451   -2.3650339    4.7553365
H    -2.7527021   -0.4154062    3.8017342
N    -0.8163498    2.4183728    1.5543468
H     0.6856472    1.5157013   -1.4528148
C     0.6638975    1.1671894   -0.4166241
C     0.4808754    0.0959589    0.2799620
C     0.2934903   -1.3751688    0.0287064
H     1.1764943   -1.9454385    0.3875599
O     0.1108113   -1.6183489   -1.3743703
H    -0.6198903   -1.7588766    0.5291987
C     1.2148822   -2.0214416   -2.0778786
C     0.9116156   -2.2027878   -3.5308409
O     2.3038448   -2.2167652   -1.5675274
C     0.4570424   -2.5830452   -6.2818126
C    -0.5934419   -2.1600732   -5.4474376
C    -0.3701491   -1.9689868   -4.0754074
C     1.9629695   -2.6268606   -4.3723148
C     1.7357560   -2.8156257   -5.7425261
H     0.2779652   -2.7320770   -7.3598355
H    -1.5950647   -1.9776276   -5.8706516
H    -1.1845808   -1.6372763   -3.4145553
H     2.9540616   -2.8025317   -3.9254058
H     2.5594795   -3.1462033   -6.3964612

$vibrational spectrum
#  mode     symmetry     wave number   IR intensity    selection rules
#                         cm**(-1)        km/mol          IR     RAMAN
     1        a            -278.19         0.00000        YES     YES
```

2		0.00	0.00000	-	-
3		0.00	0.00000	-	-
4		0.00	0.00000	-	-
5		0.00	0.00000	-	-
6		0.00	0.00000	-	-
7		0.00	0.00000	-	-
8	a	14.29	0.20383	YES	YES
9	a	18.08	0.34253	YES	YES
10	a	22.29	0.24399	YES	YES
11	a	44.01	0.53700	YES	YES
12	a	48.83	0.03663	YES	YES
13	a	55.33	0.76764	YES	YES
14	a	62.75	0.67513	YES	YES
15	a	72.87	0.20665	YES	YES
16	a	81.93	0.03362	YES	YES
17	a	91.19	0.30181	YES	YES
18	a	97.97	0.10896	YES	YES
19	a	100.80	0.65510	YES	YES
20	a	104.64	0.37368	YES	YES
21	a	131.83	0.49765	YES	YES
22	a	143.99	0.40575	YES	YES
23	a	153.64	0.20252	YES	YES
24	a	174.60	0.14773	YES	YES
25	a	189.03	0.20652	YES	YES
26	a	212.84	2.82187	YES	YES
27	a	221.80	0.80722	YES	YES
28	a	240.53	0.72260	YES	YES
29	a	261.29	0.28153	YES	YES
30	a	281.46	0.06689	YES	YES
31	a	337.95	16.67654	YES	YES
32	a	345.86	3.26526	YES	YES
33	a	370.77	4.72862	YES	YES
34	a	372.63	8.71314	YES	YES
35	a	404.28	0.00737	YES	YES
36	a	416.30	4.57346	YES	YES
37	a	428.03	2.77797	YES	YES
38	a	438.60	0.63452	YES	YES
39	a	447.54	4.17340	YES	YES
40	a	457.06	1.37976	YES	YES
41	a	464.31	0.81569	YES	YES
42	a	474.49	7.60730	YES	YES
43	a	486.49	5.48000	YES	YES
44	a	492.07	3.12404	YES	YES
45	a	497.16	0.53026	YES	YES
46	a	520.26	8.99771	YES	YES
47	a	536.66	8.13746	YES	YES
48	a	539.91	7.75461	YES	YES
49	a	557.69	1.52783	YES	YES
50	a	594.61	14.24688	YES	YES

TS_{23bc}

SCF Energy (au) BP86/SV(P) -2281.108277972
SCF Energy (au) PBE0/def2-TZVPP -2280.675226907
SCF Energy (au) PBE0/def2-TZVPP -2280.6812177349 (Toluene Correction)
Zero Point Energy (au) 0.3569715
Chemical Potential (kJ mol^{-1}) 792.89
Dispersion Correction (au) PBE0/def2-TZVPP -0.06589746

xyz coordinates
47

Mn	1.2409727	0.0904313	1.1054278
C	1.7311248	0.3069725	2.8341453
C	2.9349362	-0.2493845	0.6484351
C	1.4660984	1.8319250	0.7125130
O	1.6364875	2.9606134	0.4571049
O	4.0432610	-0.4834398	0.3624492
O	2.0488129	0.4436701	3.9464924
C	-1.4110895	-0.8149669	2.1472777
C	-1.4941407	1.4652372	1.6033007
C	-2.8401766	1.5663616	1.9674951
C	-3.4994615	0.4091231	2.4211488
C	-2.7800988	-0.7837775	2.5083186
C	-0.5475761	-1.9909043	2.2519126
C	0.7415510	-1.8796712	1.6418711
C	1.6444047	-2.9638656	1.8098994
C	1.2824482	-4.1071377	2.5287496
C	-0.0064634	-4.2200319	3.0972038
C	-0.9113734	-3.1642600	2.9579791
H	-0.9461394	2.3464649	1.2361120
H	-3.3574921	2.5350863	1.8893364
H	-4.5644064	0.4401332	2.7038761
H	-3.2718994	-1.7047943	2.8544300
H	2.6470104	-2.9102543	1.3538155
H	2.0115115	-4.9257753	2.6532134
H	-0.2919077	-5.1243727	3.6582021
H	-1.9036072	-3.2389523	3.4322280
N	-0.7912019	0.3135281	1.6784625
C	0.2036167	0.4229103	-2.1177340
C	0.4513932	-0.2496748	-0.8076748
C	0.5544724	-1.4233179	-0.2809152
H	0.5633337	-2.4760312	-0.5790808
C	1.5251228	0.9180035	-2.7641032
H	-0.2221469	-0.3552697	-2.7995672
C	-0.8249443	1.5785412	-2.0498146
H	1.9931170	1.6754389	-2.0945966
C	1.2743359	1.5382902	-4.1498764
H	2.2434790	0.0717299	-2.8390196
C	-1.0745684	2.1931968	-3.4382736
H	-0.4370484	2.3638521	-1.3613850
H	-1.7785201	1.2070255	-1.6127079
H	2.2338584	1.9152600	-4.5702918
C	0.2347654	2.6710113	-4.0898355
H	0.9156396	0.7455923	-4.8492349
H	-1.7966169	3.0368433	-3.3533072
H	-1.5580063	1.4319299	-4.0963259
H	0.0361840	3.0674924	-5.1113184
H	0.6509482	3.5192177	-3.4963377

```
$vibrational spectrum
#  mode     symmetry     wave number   IR intensity    selection rules
#                         cm**(-1)        km/mol         IR     RAMAN
     1        a          -233.32          0.00000       YES     YES
     2                      0.00          0.00000        -       -
     3                      0.00          0.00000        -       -
     4                      0.00          0.00000        -       -
     5                      0.00          0.00000        -       -
     6                      0.00          0.00000        -       -
     7                      0.00          0.00000        -       -
     8        a            25.44          0.27019       YES     YES
     9        a            35.20          0.13176       YES     YES
    10        a            46.20          0.02342       YES     YES
    11        a            53.63          0.13003       YES     YES
    12        a            66.66          0.05869       YES     YES
    13        a            78.23          0.40040       YES     YES
    14        a            89.32          0.15455       YES     YES
    15        a            92.83          0.72234       YES     YES
    16        a           100.02          0.31098       YES     YES
    17        a           107.64          0.22848       YES     YES
    18        a           111.47          0.08287       YES     YES
    19        a           118.84          0.19875       YES     YES
    20        a           139.08          0.70360       YES     YES
    21        a           164.03          0.45277       YES     YES
    22        a           178.62          0.17963       YES     YES
    23        a           203.50          0.48532       YES     YES
    24        a           222.28          0.01933       YES     YES
    25        a           230.18          2.70261       YES     YES
    26        a           252.56          0.24883       YES     YES
    27        a           275.88          0.27280       YES     YES
    28        a           297.12          0.59696       YES     YES
    29        a           350.68          0.94622       YES     YES
    30        a           357.11          3.26921       YES     YES
    31        a           369.95          2.21091       YES     YES
    32        a           406.80          5.59754       YES     YES
    33        a           420.17          1.06675       YES     YES
    34        a           426.52          2.17763       YES     YES
    35        a           438.74          0.84404       YES     YES
    36        a           454.19         11.41550       YES     YES
    37        a           459.39          1.23093       YES     YES
    38        a           471.27          2.01419       YES     YES
    39        a           471.95          3.22404       YES     YES
    40        a           489.54          1.07058       YES     YES
    41        a           497.71          3.64178       YES     YES
    42        a           501.29          0.55328       YES     YES
    43        a           512.85          3.57526       YES     YES
    44        a           538.43          8.95694       YES     YES
    45        a           546.38         13.34263       YES     YES
    46        a           554.60          0.78629       YES     YES
    47        a           575.01          5.02399       YES     YES
    48        a           608.92         30.54046       YES     YES
    49        a           620.85          1.38396       YES     YES
    50        a           626.27         27.58342       YES     YES
```

TS_{23bc}'

SCF Energy (au) BP86/SV(P)	-2281.103977722	
SCF Energy (au) PBE0/def2-TZVPP	-2280.671218205	
SCF Energy (au) PBE0/def2-TZVPP	-2280.6773988784	(Toluene Correction)
Zero Point Energy (au)	0.3568424	
Chemical Potential (kJ mol^{-1})	797.65	
Dispersion Correction (au) PBE0/def2-TZVPP	-0.06661635	

xyz coordinates
47

Mn	1.0584974	2.0075745	0.8636094
C	1.4065610	2.2963033	2.6176727
C	2.7788548	1.6425535	0.5573960
C	1.3205956	3.7147665	0.3651363
O	1.4974053	4.8097286	-0.0032252
O	3.9057319	1.3940366	0.3681308
O	1.6234799	2.4911867	3.7462495
C	-1.7061683	1.1522758	1.6243631
C	-1.6886102	3.4310935	1.0701333
C	-3.0684950	3.5500041	1.2626764
C	-3.7950418	2.4032639	1.6319445
C	-3.1089848	1.2000750	1.8086790
C	-0.8726400	-0.0319920	1.8388759
C	0.4736055	0.0450664	1.3512083
C	1.3479316	-1.0237779	1.6922311
C	0.9071021	-2.1236832	2.4369192
C	-0.4377380	-2.2114362	2.8578647
C	-1.3174290	-1.1648302	2.5617230
H	-1.0871356	4.3015186	0.7649208
H	-3.5595699	4.5246115	1.1173136
H	-4.8867356	2.4495819	1.7772479
H	-3.6530995	0.2847545	2.0850452
H	2.4021464	-0.9837975	1.3745237
H	1.6198810	-2.9228353	2.7019266
H	-0.7867523	-3.0815597	3.4369294
H	-2.3524705	-1.2085626	2.9388834
N	-1.0173060	2.2713703	1.2407304
H	0.2052153	2.1992758	-1.9447688
C	0.3697731	1.6399617	-1.0188688
C	0.4261576	0.4189076	-0.5900383
C	0.4748275	-0.9544359	-1.2231924
C	-0.5425282	-1.0274437	-2.3907468
H	0.1864101	-1.7265873	-0.4769103
C	1.8966294	-1.2981526	-1.7380544
C	1.9297454	-2.6760491	-2.4233278
H	2.6279615	-1.2662767	-0.9017107
H	2.2091946	-0.5067970	-2.4582144
H	-1.5643380	-0.8028396	-2.0120097
C	-0.5107904	-2.4069750	-3.0738193
H	-0.2949055	-0.2366847	-3.1362682
H	-0.8600705	-3.1820754	-2.3511143
H	-1.2319186	-2.4215374	-3.9225519
H	1.7134871	-3.4671955	-1.6670317
H	2.9551625	-2.8796649	-2.8066906
C	0.9019421	-2.7681382	-3.5641145
H	0.9073472	-3.7886735	-4.0097542
H	1.1970819	-2.0659092	-4.3799218

$vibrational spectrum

# mode	symmetry	wave number cm**(-1)	IR intensity km/mol	selection rules IR	RAMAN
1	a	-292.01	0.00000	YES	YES
2	a	-3.78	0.00000	YES	YES
3		0.00	0.00000	-	-
4		0.00	0.00000	-	-
5		0.00	0.00000	-	-
6		0.00	0.00000	-	-
7		0.00	0.00000	-	-
8		0.00	0.00000	-	-
9	a	28.49	0.43330	YES	YES
10	a	46.44	0.00067	YES	YES
11	a	51.71	0.11284	YES	YES
12	a	64.85	0.27708	YES	YES
13	a	79.34	0.02072	YES	YES
14	a	89.33	0.29511	YES	YES
15	a	92.02	0.20021	YES	YES
16	a	101.05	0.72661	YES	YES
17	a	104.97	0.29552	YES	YES
18	a	111.48	0.11726	YES	YES
19	a	127.74	0.14195	YES	YES
20	a	163.52	1.93071	YES	YES
21	a	168.19	0.28006	YES	YES
22	a	171.57	0.06476	YES	YES
23	a	201.66	2.28831	YES	YES
24	a	221.68	0.90382	YES	YES
25	a	228.11	0.13987	YES	YES
26	a	240.12	0.50932	YES	YES
27	a	263.48	0.36210	YES	YES
28	a	281.08	0.10385	YES	YES
29	a	342.96	0.25244	YES	YES
30	a	356.62	1.72735	YES	YES
31	a	407.65	3.82895	YES	YES
32	a	413.53	1.94417	YES	YES
33	a	420.48	4.32053	YES	YES
34	a	430.59	1.93295	YES	YES
35	a	441.75	1.74368	YES	YES
36	a	460.81	2.24741	YES	YES
37	a	463.86	0.83593	YES	YES
38	a	483.58	1.45069	YES	YES
39	a	486.62	1.58686	YES	YES
40	a	495.34	0.90332	YES	YES
41	a	501.19	0.27970	YES	YES
42	a	520.05	2.89286	YES	YES
43	a	523.61	6.81593	YES	YES
44	a	539.03	12.66528	YES	YES
45	a	548.96	4.06695	YES	YES
46	a	559.92	3.76532	YES	YES
47	a	587.65	7.96722	YES	YES
48	a	609.87	30.76435	YES	YES
49	a	621.24	4.29310	YES	YES
50	a	627.98	39.61848	YES	YES

TS_{23bd}

SCF Energy (au) BP86/SV(P)	-2411.377999102	
SCF Energy (au) PBE0/def2-TZVPP	-2410.917172120	
SCF Energy (au) PBE0/def2-TZVPP	-2410.9254826611	(Toluene Correction)
Zero Point Energy (au)	0.3599214	
Chemical Potential (kJ mol^{-1})	787.62	
Dispersion Correction (au) PBE0/def2-TZVPP	-0.06897874	

xyz coordinates
49

Mn	1.6834630	-0.0525723	1.9499502
C	2.0090554	0.2577901	3.6992573
C	3.3713646	-0.5845235	1.6926125
C	2.1363629	1.6313506	1.4813795
O	2.4693042	2.7221616	1.2212537
O	4.4707655	-0.9441518	1.5293227
O	2.2100084	0.4495467	4.8302512
C	-1.1470986	-0.5976926	2.7197754
C	-0.9324977	1.5925442	1.9007414
C	-2.2982368	1.8471218	2.0506641
C	-3.1213617	0.8185427	2.5459622
C	-2.5420504	-0.4077008	2.8750646
C	-0.4262491	-1.8243502	3.0472378
C	0.9386815	-1.8829973	2.6156958
C	1.6931261	-3.0281449	2.9933610
C	1.1210401	-4.0697105	3.7279511
C	-0.2386122	-4.0167145	4.1126819
C	-1.0019301	-2.8981569	3.7716426
H	-0.2574610	2.3661431	1.5045454
H	-2.7036819	2.8331761	1.7769299
H	-4.2059321	0.9735543	2.6683121
H	-3.1647403	-1.2331882	3.2502307
H	2.7509778	-3.1042007	2.6890843
H	1.7393524	-4.9380311	4.0129915
H	-0.6882753	-4.8412553	4.6890756
H	-2.0532278	-2.8431750	4.0993318
N	-0.3619388	0.4102460	2.2236019
C	0.5722182	0.1518922	-1.2459288
C	1.1001983	1.3866554	-1.7046310
C	0.7455663	1.9262172	-2.9429411
C	-0.1863450	1.2707796	-3.8004005
C	-0.7356187	0.0382648	-3.3340738
C	-0.3620324	-0.4981225	-2.1018884
H	1.8215642	1.9339246	-1.0809394
H	1.2023613	2.8797746	-3.2433579
N	-0.5456798	1.8075638	-5.0264299
H	-1.4654832	-0.5137298	-3.9434549
H	-0.8068323	-1.4520759	-1.7746075
C	0.9425459	-0.4725647	-0.0024645
C	1.0810974	-1.5992534	0.6214019
H	1.3031505	-2.6483348	0.4068866
C	-1.4962568	1.1107966	-5.8746984
C	0.0455147	3.0575437	-5.4719449
H	-0.1894395	3.9030832	-4.7811889
H	-0.3548213	3.3171793	-6.4718110
H	1.1569677	2.9896755	-5.5569684
H	-1.1337429	0.0962807	-6.1692087
H	-1.6569254	1.6947161	-6.8027670
H	-2.4882151	0.9841219	-5.3774916

$vibrational spectrum

# mode	symmetry	wave number cm**(-1)	IR intensity km/mol	selection rules IR	RAMAN
1	a	-232.90	0.00000	YES	YES
2		0.00	0.00000	-	-
3		0.00	0.00000	-	-
4		0.00	0.00000	-	-
5		0.00	0.00000	-	-
6		0.00	0.00000	-	-
7		0.00	0.00000	-	-
8	a	17.63	0.42854	YES	YES
9	a	23.50	0.02888	YES	YES
10	a	39.27	0.17692	YES	YES
11	a	52.61	0.70633	YES	YES
12	a	60.93	0.75764	YES	YES
13	a	69.86	0.00642	YES	YES
14	a	73.50	0.05396	YES	YES
15	a	79.07	0.32040	YES	YES
16	a	87.05	0.20507	YES	YES
17	a	91.02	0.05740	YES	YES
18	a	93.52	1.26300	YES	YES
19	a	102.25	0.54171	YES	YES
20	a	109.71	0.09419	YES	YES
21	a	113.69	0.14201	YES	YES
22	a	133.88	0.09814	YES	YES
23	a	172.62	0.27658	YES	YES
24	a	173.33	0.33520	YES	YES
25	a	192.99	0.53918	YES	YES
26	a	203.92	0.52000	YES	YES
27	a	211.52	0.00193	YES	YES
28	a	232.77	1.06116	YES	YES
29	a	249.78	0.59980	YES	YES
30	a	259.75	0.23298	YES	YES
31	a	279.20	0.33588	YES	YES
32	a	294.51	4.85642	YES	YES
33	a	341.09	0.32281	YES	YES
34	a	358.51	3.22369	YES	YES
35	a	396.61	1.95742	YES	YES
36	a	412.41	1.72339	YES	YES
37	a	419.20	0.32576	YES	YES
38	a	428.07	21.42381	YES	YES
39	a	434.00	1.48273	YES	YES
40	a	443.83	5.47313	YES	YES
41	a	459.17	0.37495	YES	YES
42	a	467.34	6.59154	YES	YES
43	a	473.89	0.79606	YES	YES
44	a	481.82	2.47340	YES	YES
45	a	494.42	3.20775	YES	YES
46	a	502.22	4.26468	YES	YES
47	a	507.05	10.25813	YES	YES
48	a	526.69	7.26318	YES	YES
49	a	543.84	16.93748	YES	YES
50	a	545.01	27.45832	YES	YES

TS_{23bd}'

SCF Energy (au) BP86/SV(P) -2411.368746066
SCF Energy (au) PBE0/def2-TZVPP -2410.909311614
SCF Energy (au) PBE0/def2-TZVPP -2410.9178265345 (Toluene Correction)
Zero Point Energy (au) 0.3597223
Chemical Potential (kJ mol^{-1}) 787.33
Dispersion Correction (au) PBE0/def2-TZVPP -0.07129753

xyz coordinates
49

Mn	0.8765013	2.7963850	0.9256197
C	0.9665915	3.2424892	2.6802605
C	2.6063238	2.3530971	0.9199746
C	1.2790913	4.4416824	0.3257752
O	1.5517344	5.4917965	-0.1099508
O	3.7367907	2.0547615	0.9224713
O	1.0198641	3.5439671	3.8052743
C	-1.9912887	2.1018574	1.3960254
C	-1.8177428	4.2754438	0.5328868
C	-3.2051678	4.4498593	0.5301760
C	-4.0151754	3.3853344	0.9656546
C	-3.4030226	2.2059291	1.3950251
C	-1.2377681	0.9352140	1.8589081
C	0.1509035	0.9026020	1.5138263
C	0.9280650	-0.1658238	2.0340893
C	0.3691824	-1.1376972	2.8695596
C	-1.0078131	-1.1055827	3.1834053
C	-1.8056209	-0.0770676	2.6726767
H	-1.1489615	5.0789482	0.1874558
H	-3.6362272	5.4026418	0.1861606
H	-5.1138208	3.4754524	0.9651434
H	-4.0118098	1.3510936	1.7253487
H	1.9974218	-0.2319363	1.7741643
H	1.0090422	-1.9362397	3.2817040
H	-1.4492273	-1.8762165	3.8362410
H	-2.8727223	-0.0335685	2.9468625
N	-1.2177181	3.1397826	0.9516331
H	0.4686035	2.7499073	-1.9910079
C	0.4881411	2.2803288	-1.0018763
C	0.4290407	1.0882186	-0.4932435
C	0.6288202	-0.2901795	-0.9466634
C	1.0131190	-2.9201236	-2.0568575
C	1.9062441	-1.8495803	-2.3563812
C	1.7108367	-0.5755514	-1.8163986
C	-0.2496384	-1.3560608	-0.6375642
C	-0.0745392	-2.6310950	-1.1787563
N	1.1945533	-4.1852326	-2.5914633
H	2.7696160	-2.0099100	-3.0174565
H	2.4214364	0.2297555	-2.0624777
H	-1.1022642	-1.1757743	0.0361319
H	-0.7974877	-3.4146339	-0.9115329
C	0.2833252	-5.2604066	-2.2400371
C	2.3290919	-4.4509679	-3.4588670
H	3.3052120	-4.2826688	-2.9419781
H	2.3149097	-3.8095463	-4.3723163
H	2.2986772	-5.5081616	-3.7892528
H	-0.7685960	-5.0289145	-2.5330402
H	0.2886267	-5.4784424	-1.1443368
H	0.5848459	-6.1851661	-2.7709959

```
$vibrational spectrum
#  mode     symmetry     wave number   IR intensity    selection rules
#                         cm**(-1)        km/mol         IR     RAMAN
     1        a          -281.14         0.00000        YES     YES
     2                      0.00         0.00000         -       -
     3                      0.00         0.00000         -       -
     4                      0.00         0.00000         -       -
     5                      0.00         0.00000         -       -
     6                      0.00         0.00000         -       -
     7                      0.00         0.00000         -       -
     8        a            20.19         0.87179        YES     YES
     9        a            27.26         0.34281        YES     YES
    10        a            38.91         0.12241        YES     YES
    11        a            48.94         0.33750        YES     YES
    12        a            62.05         0.55669        YES     YES
    13        a            65.23         0.13583        YES     YES
    14        a            73.20         0.38913        YES     YES
    15        a            80.39         0.02581        YES     YES
    16        a            83.34         0.07330        YES     YES
    17        a            89.03         0.82535        YES     YES
    18        a            98.01         0.35279        YES     YES
    19        a           103.39         0.62865        YES     YES
    20        a           103.76         0.08370        YES     YES
    21        a           128.54         0.82791        YES     YES
    22        a           136.25         0.50399        YES     YES
    23        a           151.00         8.53754        YES     YES
    24        a           172.53         1.79403        YES     YES
    25        a           173.86         0.17583        YES     YES
    26        a           205.54         2.72256        YES     YES
    27        a           208.09         0.60019        YES     YES
    28        a           220.35         1.63172        YES     YES
    29        a           243.30         0.79741        YES     YES
    30        a           264.70         1.09374        YES     YES
    31        a           280.06         1.21745        YES     YES
    32        a           283.52         4.52251        YES     YES
    33        a           355.23         1.65612        YES     YES
    34        a           368.43        10.41136        YES     YES
    35        a           371.16        17.81186        YES     YES
    36        a           409.54         9.00014        YES     YES
    37        a           420.83         0.00760        YES     YES
    38        a           432.92         1.90686        YES     YES
    39        a           448.63         5.06107        YES     YES
    40        a           458.25         0.05615        YES     YES
    41        a           464.84         0.40177        YES     YES
    42        a           476.00         5.71754        YES     YES
    43        a           482.29         1.90114        YES     YES
    44        a           493.30         1.86918        YES     YES
    45        a           498.67         1.51402        YES     YES
    46        a           505.43        25.56968        YES     YES
    47        a           512.67         8.91216        YES     YES
    48        a           534.45         2.22502        YES     YES
    49        a           538.22        10.33803        YES     YES
    50        a           548.54         5.78802        YES     YES
```

TS_{23be}′

SCF Energy (au) BP86/SV(P)	-2391.942812015
SCF Energy (au) PBE0/def2-TZVPP	-2391.495806536
SCF Energy (au) PBE0/def2-TZVPP	-2391.5041329799 (Toluene Correction)
Zero Point Energy (au)	0.3207053
Chemical Potential (kJ mol^{-1})	692.34
Dispersion Correction (au) PBE0/def2-TZVPP	-0.06659232

xyz coordinates
45

Mn	0.8603316	2.3382340	0.6760216
C	0.9379708	2.8134409	2.4240064
C	2.5972322	1.9219831	0.6837214
C	1.2383753	3.9807492	0.0509475
O	1.4943236	5.0284896	-0.3993022
O	3.7314917	1.6393026	0.6929300
O	0.9821245	3.1300685	3.5447706
C	-2.0001052	1.6116006	1.1448314
C	-1.8528766	3.7743982	0.2505319
C	-3.2426123	3.9286207	0.2380344
C	-4.0396691	2.8590150	0.6846638
C	-3.4130628	1.6950717	1.1349044
C	-1.2323193	0.4638250	1.6312906
C	0.1593725	0.4460522	1.2975217
C	0.9491070	-0.6005383	1.8421360
C	0.3982303	-1.5675800	2.6885341
C	-0.9812609	-1.5513558	2.9909768
C	-1.7903207	-0.5429819	2.4581476
H	-1.1942219	4.5826265	-0.1032778
H	-3.6853800	4.8699977	-0.1221602
H	-5.1394155	2.9332059	0.6768765
H	-4.0114237	0.8367251	1.4749357
H	2.0217462	-0.6538559	1.5932197
H	1.0465926	-2.3497724	3.1180270
H	-1.4162982	-2.3185221	3.6519113
H	-2.8597754	-0.5109985	2.7242278
N	-1.2390138	2.6535486	0.6889858
H	0.4719437	2.2253396	-2.2449167
C	0.4880967	1.7815788	-1.2440234
C	0.4524668	0.6049359	-0.6992029
C	0.6761080	-0.7825776	-1.1266604
C	1.1038637	-3.3947654	-2.1684267
C	2.0076121	-2.3538623	-2.4722466
C	1.7857646	-1.0670649	-1.9553061
C	-0.2132866	-1.8444932	-0.8212391
C	-0.0108466	-3.1234504	-1.3391414
O	1.2167345	-4.6758329	-2.6162467
H	2.8885494	-2.5306660	-3.1066706
H	2.4962790	-0.2586425	-2.1904050
H	-1.0849297	-1.6524542	-0.1762487
H	-0.7078168	-3.9463402	-1.1155303
C	2.3137170	-5.0144689	-3.4480481
H	2.1995729	-6.0916702	-3.6844566
H	3.2895722	-4.8568574	-2.9300167
H	2.3074563	-4.4300586	-4.3986280

$vibrational spectrum

# mode	symmetry	wave number	IR intensity	selection rules	
#		cm**(-1)	km/mol	IR	RAMAN
1	a	-282.68	0.00000	YES	YES
2		0.00	0.00000	-	-
3		0.00	0.00000	-	-

4		0.00	0.00000	-	-
5		0.00	0.00000	-	-
6		0.00	0.00000	-	-
7		0.00	0.00000	-	-
8	a	20.94	0.84604	YES	YES
9	a	29.19	0.79127	YES	YES
10	a	45.47	0.13936	YES	YES
11	a	54.95	0.03365	YES	YES
12	a	65.46	0.30469	YES	YES
13	a	69.54	0.04339	YES	YES
14	a	82.63	0.10799	YES	YES
15	a	89.06	0.48148	YES	YES
16	a	96.18	0.65098	YES	YES
17	a	100.10	0.89539	YES	YES
18	a	104.29	0.41734	YES	YES
19	a	119.76	1.61318	YES	YES
20	a	133.23	0.66472	YES	YES
21	a	151.62	0.52672	YES	YES
22	a	160.18	5.85576	YES	YES
23	a	176.79	0.15200	YES	YES
24	a	190.84	1.83338	YES	YES
25	a	217.29	0.68853	YES	YES
26	a	229.68	2.61832	YES	YES
27	a	245.29	0.45810	YES	YES
28	a	266.00	0.50973	YES	YES
29	a	274.25	4.73900	YES	YES
30	a	282.77	0.36243	YES	YES
31	a	356.37	1.42389	YES	YES
32	a	367.34	4.02929	YES	YES
33	a	410.21	3.71430	YES	YES
34	a	411.57	16.17621	YES	YES
35	a	414.46	0.20458	YES	YES
36	a	433.24	4.05431	YES	YES
37	a	447.79	3.75264	YES	YES
38	a	461.59	0.27501	YES	YES
39	a	476.88	7.40511	YES	YES
40	a	481.37	0.22403	YES	YES
41	a	490.38	1.44038	YES	YES
42	a	496.63	4.12182	YES	YES
43	a	503.13	10.48972	YES	YES
44	a	521.41	27.61373	YES	YES
45	a	533.34	2.49739	YES	YES
46	a	537.21	9.83007	YES	YES
47	a	546.83	10.77086	YES	YES
48	a	552.96	7.48015	YES	YES
49	a	559.28	19.86247	YES	YES
50	a	608.71	41.44731	YES	YES

TS_{23be}

```
SCF Energy (au) BP86/SV(P)                     -2391.952065529
SCF Energy (au) PBE0/def2-TZVPP                -2391.503796793
SCF Energy (au) PBE0/def2-TZVPP                -2391.5119222258 (Toluene Correction)
Zero Point Energy (au)                         0.3209337
Chemical Potential (kJ mol-1)                  692.54
Dispersion Correction (au) PBE0/def2-TZVPP -0.06432154
```

xyz coordinates
45

```
Mn    0.8621936   -1.7437725    0.7912015
C     2.0691026   -2.3221484    2.0057398
C     0.3354707   -3.4232205    0.4714875
C     2.0477510   -1.8564604   -0.5663307
O     2.8360809   -1.9745973   -1.4217577
O    -0.0127487   -4.5200114    0.2724205
O     2.8389768   -2.6915675    2.7970921
C     0.7186338    0.8031975    2.3400358
C     2.1364778    1.0577432    0.4858988
C     2.3787566    2.4023179    0.7796241
C     1.7428917    2.9688187    1.8998988
C     0.9075607    2.1655473    2.6776365
C    -0.1123756   -0.1382387    3.0862403
C    -0.3468487   -1.4032020    2.4575729
C    -1.0799262   -2.3732746    3.1945911
C    -1.5781318   -2.0972743    4.4704671
C    -1.3719378   -0.8308322    5.0635798
C    -0.6428491    0.1382771    4.3711397
H     2.6030517    0.5818446   -0.3898668
H     3.0483642    2.9911698    0.1341671
H     1.8951081    4.0295186    2.1584286
H     0.3867636    2.5872098    3.5498997
H    -1.2721714   -3.3628597    2.7460214
H    -2.1328733   -2.8773296    5.0191101
H    -1.7684895   -0.6134081    6.0684463
H    -0.4549848    1.1142585    4.8487020
N     1.3329404    0.2712019    1.2367404
C    -0.7281873   -0.0773813   -1.6978335
C     0.0682062   -0.3445665   -2.8377042
C    -0.1602890    0.2987836   -4.0637801
C    -1.1912045    1.2563263   -4.1786851
C    -1.9875578    1.5500737   -3.0454890
C    -1.7625880    0.8964494   -1.8360872
H     0.8807869   -1.0823544   -2.7715149
H     0.4793908    0.0469182   -4.9224714
O    -1.4906495    1.9459369   -5.3146853
H    -2.7851059    2.3030503   -3.1476681
H    -2.3898731    1.1356930   -0.9621762
C    -0.5628082   -0.7472342   -0.4310331
C    -1.1738842   -1.1609112    0.6325697
H    -2.1688995   -1.4811356    0.9544857
C    -0.7396093    1.6854028   -6.4882063
H    -1.1636778    2.3394228   -7.2766994
H    -0.8310382    0.6206259   -6.8093990
H     0.3402008    1.9319926   -6.3518091
```

```
$vibrational spectrum
#  mode     symmetry     wave number   IR intensity    selection rules
#                         cm**(-1)        km/mol          IR     RAMAN
     1        a            -227.33        0.00000        YES     YES
     2                        0.00        0.00000         -       -
     3                        0.00        0.00000         -       -
```

4		0.00	0.00000	-	-
5		0.00	0.00000	-	-
6		0.00	0.00000	-	-
7		0.00	0.00000	-	-
8	a	19.41	0.41189	YES	YES
9	a	27.78	0.54285	YES	YES
10	a	40.10	0.12463	YES	YES
11	a	54.50	0.43267	YES	YES
12	a	67.65	0.31805	YES	YES
13	a	70.24	0.07862	YES	YES
14	a	86.69	0.50336	YES	YES
15	a	90.08	0.47264	YES	YES
16	a	91.94	1.11787	YES	YES
17	a	100.97	0.83703	YES	YES
18	a	109.03	0.03787	YES	YES
19	a	113.63	0.02923	YES	YES
20	a	123.87	0.95877	YES	YES
21	a	148.01	0.67670	YES	YES
22	a	176.03	0.53732	YES	YES
23	a	181.06	1.33249	YES	YES
24	a	202.81	0.52002	YES	YES
25	a	232.05	0.65903	YES	YES
26	a	233.23	0.92839	YES	YES
27	a	249.69	3.09180	YES	YES
28	a	259.35	0.42038	YES	YES
29	a	278.91	0.18843	YES	YES
30	a	287.88	3.04993	YES	YES
31	a	357.46	1.85353	YES	YES
32	a	367.87	5.71411	YES	YES
33	a	404.64	1.03540	YES	YES
34	a	413.15	1.34921	YES	YES
35	a	422.17	1.05395	YES	YES
36	a	428.34	9.27362	YES	YES
37	a	438.19	0.18566	YES	YES
38	a	449.62	3.72182	YES	YES
39	a	461.50	0.42404	YES	YES
40	a	472.12	2.83535	YES	YES
41	a	479.94	1.51383	YES	YES
42	a	486.82	0.41372	YES	YES
43	a	498.30	1.84853	YES	YES
44	a	507.44	9.78614	YES	YES
45	a	529.46	6.30823	YES	YES
46	a	544.91	28.96894	YES	YES
47	a	545.21	18.42229	YES	YES
48	a	553.60	0.31762	YES	YES
49	a	568.43	45.03624	YES	YES
50	a	610.58	21.48888	YES	YES

TS_{23bf}

```
SCF Energy (au) BP86/SV(P)                       -2376.678364254
SCF Energy (au) PBE0/def2-TZVPP                  -2376.251797975
SCF Energy (au)  PBE0/def2-TZVPP                 -2376.2588756073 (Toluene Correction)
Zero Point Energy (au)                           0.2815138
Chemical Potential (kJ mol-1)                    593.78
Dispersion Correction (au) PBE0/def2-TZVPP       -0.06085022

xyz coordinates
41

Mn    1.5619780    0.3660655    0.7861690
C     1.9208386    0.6208485    2.5404651
C     3.2541360   -0.1276676    0.4770720
C     1.9785849    2.0697894    0.3569203
O     2.2835054    3.1695638    0.1046869
O     4.3562761   -0.4595864    0.2814469
O     2.1400928    0.7768074    3.6726748
C    -1.2440467   -0.2454675    1.5945022
C    -1.0702898    1.9843787    0.8796638
C    -2.4348486    2.2155314    1.0724391
C    -3.2360103    1.1542217    1.5324193
C    -2.6368801   -0.0802472    1.7882012
C    -0.5011196   -1.4767078    1.8553073
C     0.8563183   -1.5017281    1.4009004
C     1.6332937   -2.6484384    1.7196354
C     1.0885552   -3.7252870    2.4242352
C    -0.2647148   -3.7051008    2.8323911
C    -1.0494699   -2.5854594    2.5475681
H    -0.4127089    2.7847534    0.5080550
H    -2.8564114    3.2094672    0.8580455
H    -4.3190959    1.2907115    1.6852720
H    -3.2426676   -0.9298586    2.1358996
H     2.6853571   -2.6982887    1.3913901
H     1.7225618   -4.5955856    2.6642926
H    -0.6941026   -4.5576641    3.3827492
H    -2.0952652   -2.5585120    2.8949469
N    -0.4812431    0.7936973    1.1302499
C     0.3501391    0.6559185   -2.3668529
C     0.9112785    1.8710060   -2.8413999
C     0.4926697    2.4378441   -4.0527881
C    -0.5125640    1.8032486   -4.7954455
C    -1.1025882    0.6096096   -4.3535508
C    -0.6698862    0.0429080   -3.1497629
H     1.6958594    2.3738398   -2.2581371
H     0.9341938    3.3739313   -4.4286426
F    -0.9275114    2.3519564   -5.9545333
H    -1.8929051    0.1390941   -4.9592008
H    -1.1268168   -0.8930985   -2.7913662
C     0.7735382    0.0070599   -1.1487214
C     0.9568754   -1.1398979   -0.5775206
H     1.1704449   -2.1796208   -0.8418947

$vibrational spectrum
#  mode     symmetry     wave number   IR intensity    selection rules
#                         cm**(-1)        km/mol          IR     RAMAN
     1        a            -227.53        0.00000        YES     YES
     2                        0.00        0.00000         -       -
     3                        0.00        0.00000         -       -
     4                        0.00        0.00000         -       -
     5                        0.00        0.00000         -       -
     6                        0.00        0.00000         -       -
     7                        0.00        0.00000         -       -
```

8	a	19.21	0.24348	YES	YES
9	a	23.75	0.12982	YES	YES
10	a	42.92	0.09885	YES	YES
11	a	56.70	0.11916	YES	YES
12	a	70.27	0.09686	YES	YES
13	a	76.59	0.08731	YES	YES
14	a	88.98	0.27995	YES	YES
15	a	92.03	0.96432	YES	YES
16	a	97.86	0.53452	YES	YES
17	a	108.76	0.12355	YES	YES
18	a	110.28	0.02049	YES	YES
19	a	116.73	0.06153	YES	YES
20	a	146.46	0.79840	YES	YES
21	a	177.78	0.27680	YES	YES
22	a	182.39	1.18811	YES	YES
23	a	205.49	1.46760	YES	YES
24	a	233.38	0.72356	YES	YES
25	a	258.43	0.38635	YES	YES
26	a	275.64	2.52772	YES	YES
27	a	278.75	0.07715	YES	YES
28	a	355.94	2.58164	YES	YES
29	a	363.22	3.60620	YES	YES
30	a	388.27	5.12682	YES	YES
31	a	406.29	2.57962	YES	YES
32	a	411.37	0.41426	YES	YES
33	a	423.31	3.72845	YES	YES
34	a	428.92	4.46412	YES	YES
35	a	445.07	0.06098	YES	YES
36	a	456.35	2.81889	YES	YES
37	a	466.29	6.63396	YES	YES
38	a	475.36	1.39436	YES	YES
39	a	486.90	0.50086	YES	YES
40	a	497.88	4.81588	YES	YES
41	a	504.43	12.18303	YES	YES
42	a	516.93	6.22702	YES	YES
43	a	540.83	14.84431	YES	YES
44	a	543.79	23.93456	YES	YES
45	a	553.24	0.35600	YES	YES
46	a	561.21	22.34925	YES	YES
47	a	611.02	24.09647	YES	YES
48	a	620.17	2.09154	YES	YES
49	a	623.93	31.33547	YES	YES
50	a	628.11	10.34672	YES	YES

TS_{23bf}'

```
SCF Energy (au) BP86/SV(P)                  -2376.669021186
SCF Energy (au) PBE0/def2-TZVPP             -2376.243801851
SCF Energy (au) PBE0/def2-TZVPP             -2376.2510386261 (Toluene Correction)
Zero Point Energy (au)                      0.2813228
Chemical Potential (kJ mol-1)               594.57
Dispersion Correction (au)  PBE0/def2-TZVPP -0.06310967

xyz coordinates
41

Mn    1.1679605    1.7937775    0.3566876
C     1.3550067    2.1748786    2.1202758
C     2.8948304    1.3467086    0.2513224
C     1.5482691    3.4604883   -0.2019107
O     1.8028072    4.5246093   -0.6113293
O     4.0222238    1.0452474    0.1892069
O     1.4715723    2.4296359    3.2510797
C    -1.6803990    1.1013854    0.9435919
C    -1.5330810    3.3042479    0.1537376
C    -2.9181923    3.4841053    0.2210215
C    -3.7138197    2.4080422    0.6542685
C    -3.0897143    1.2110733    1.0121351
C    -0.9131107   -0.0848258    1.3288097
C     0.4585613   -0.1118585    0.9211469
C     1.2528511   -1.2010783    1.3662344
C     0.7256611   -2.2017573    2.1886064
C    -0.6352314   -2.1751473    2.5649013
C    -1.4485718   -1.1236896    2.1304586
H    -0.8768868    4.1173775   -0.1931761
H    -3.3590507    4.4503817   -0.0685604
H    -4.8107081    2.5029422    0.7075116
H    -3.6879218    0.3476784    1.3392749
H     2.3096177   -1.2610650    1.0582935
H     1.3785562   -3.0180279    2.5406817
H    -1.0517775   -2.9678258    3.2072608
H    -2.5015099   -1.0854712    2.4550728
N    -0.9214283    2.1511209    0.5024351
H     0.6243956    1.8451641   -2.5424786
C     0.6810058    1.3514882   -1.5667166
C     0.6470050    0.1528502   -1.0719944
C     0.8050998   -1.2154115   -1.5896971
C     1.1005693   -3.7481923   -2.7710760
C     2.0104261   -2.7337013   -3.0964077
C     1.8558790   -1.4713673   -2.5055919
C    -0.0902993   -2.2668373   -1.2791782
C     0.0471259   -3.5285723   -1.8715097
F     1.2421296   -4.9643436   -3.3325375
H     2.8286622   -2.9435393   -3.8026004
H     2.5658972   -0.6638700   -2.7434700
H    -0.9133767   -2.0872068   -0.5704353
H    -0.6510337   -4.3494144   -1.6453449

$vibrational spectrum
#  mode     symmetry     wave number   IR intensity    selection rules
#                         cm**(-1)        km/mol          IR     RAMAN
     1        a            -281.12        0.00000        YES     YES
     2                        0.00        0.00000         -       -
     3                        0.00        0.00000         -       -
     4                        0.00        0.00000         -       -
     5                        0.00        0.00000         -       -
     6                        0.00        0.00000         -       -
     7                        0.00        0.00000         -       -
```

8	a	23.06	0.43019	YES	YES
9	a	33.28	0.22086	YES	YES
10	a	45.13	0.06902	YES	YES
11	a	57.85	0.03442	YES	YES
12	a	65.73	0.24950	YES	YES
13	a	74.52	0.20522	YES	YES
14	a	85.64	0.15124	YES	YES
15	a	90.27	0.39165	YES	YES
16	a	99.81	0.62535	YES	YES
17	a	104.52	0.34564	YES	YES
18	a	110.26	0.08384	YES	YES
19	a	136.48	0.24950	YES	YES
20	a	154.01	0.08206	YES	YES
21	a	167.10	3.25609	YES	YES
22	a	181.48	0.60014	YES	YES
23	a	205.28	1.40959	YES	YES
24	a	218.29	0.72221	YES	YES
25	a	245.32	1.21865	YES	YES
26	a	258.04	2.84291	YES	YES
27	a	281.62	0.30776	YES	YES
28	a	356.45	1.45448	YES	YES
29	a	359.88	1.62025	YES	YES
30	a	403.54	8.10795	YES	YES
31	a	412.41	1.13305	YES	YES
32	a	413.93	0.24644	YES	YES
33	a	430.46	0.39448	YES	YES
34	a	444.28	12.65374	YES	YES
35	a	449.03	11.34976	YES	YES
36	a	460.93	0.31257	YES	YES
37	a	478.37	8.32081	YES	YES
38	a	486.58	11.15331	YES	YES
39	a	492.15	10.15353	YES	YES
40	a	496.96	1.50645	YES	YES
41	a	507.24	2.14471	YES	YES
42	a	531.58	2.35931	YES	YES
43	a	536.44	13.07556	YES	YES
44	a	545.62	3.08104	YES	YES
45	a	552.27	11.54518	YES	YES
46	a	557.93	12.44836	YES	YES
47	a	607.80	42.28800	YES	YES
48	a	619.49	1.47328	YES	YES
49	a	624.35	37.81025	YES	YES
50	a	629.46	2.75821	YES	YES

TS_{23bg}

SCF Energy (au) BP86/SV(P)	-2614.321919435
SCF Energy (au) PBE0/def2-TZVPP	-2613.938388064
SCF Energy (au) PBE0/def2-TZVPP	-2613.9456534346 (Toluene Correction)
Zero Point Energy (au)	0.2938786
Chemical Potential (kJ mol^{-1})	609.72
Dispersion Correction (au) PBE0/def2-TZVPP	-0.06429204

xyz coordinates
44

```
Mn    1.6199261    0.1839127    1.2244550
C     1.9703585    0.4585406    2.9778204
C     3.3203503   -0.2897316    0.9266841
C     2.0127327    1.8912149    0.7828043
O     2.2972423    2.9925574    0.5179697
O     4.4275292   -0.6078570    0.7384601
O     2.1853940    0.6268440    4.1086720
C    -1.1792486   -0.4538706    2.0373772
C    -1.0355679    1.7700997    1.2989351
C    -2.4029705    1.9850299    1.4916835
C    -3.1890964    0.9188300    1.9654244
C    -2.5732740   -0.3050667    2.2331667
C    -0.4201235   -1.6737136    2.3090137
C     0.9355425   -1.6886456    1.8509875
C     1.7276002   -2.8220221    2.1778069
C     1.2003632   -3.8950022    2.9016578
C    -0.1504308   -3.8841555    3.3175383
C    -0.9510923   -2.7789871    3.0201217
H    -0.3899306    2.5751603    0.9165658
H    -2.8381934    2.9706082    1.2663466
H    -4.2734418    1.0429133    2.1195822
H    -3.1670728   -1.1587135    2.5914871
H     2.7779030   -2.8640172    1.8430276
H     1.8459733   -4.7541239    3.1503903
H    -0.5662476   -4.7332208    3.8834527
H    -1.9950363   -2.7600360    3.3732780
N    -0.4304319    0.5901520    1.5618163
C     0.4252095    0.4646606   -1.9214119
C     1.0845783    1.6109801   -2.4406344
C     0.6668475    2.1973363   -3.6394776
C    -0.4316259    1.6717741   -4.3481923
C    -1.1054294    0.5431403   -3.8415792
C    -0.6837424   -0.0528002   -2.6485551
H     1.9388036    2.0406499   -1.8981213
H     1.1940743    3.0839041   -4.0254955
C    -0.8489076    2.2954447   -5.6585145
H    -1.9705211    0.1313201   -4.3839526
H    -1.2180867   -0.9313327   -2.2533843
C     0.8396665   -0.1949227   -0.7088548
C     1.0375226   -1.3347500   -0.1287520
H     1.2599484   -2.3733239   -0.3917808
F    -0.7199966    3.6466930   -5.6358574
F    -2.1388102    2.0147342   -5.9680578
F    -0.0882877    1.8497925   -6.6939033
```

```
$vibrational spectrum
#  mode     symmetry     wave number   IR intensity    selection rules
#                         cm**(-1)        km/mol          IR     RAMAN
     1        a          -221.56         0.00000        YES     YES
     2                      0.00         0.00000         -       -
     3                      0.00         0.00000         -       -
     4                      0.00         0.00000         -       -
```

5		0.00	0.00000	-	-
6		0.00	0.00000	-	-
7		0.00	0.00000	-	-
8	a	7.57	0.08787	YES	YES
9	a	12.70	0.02691	YES	YES
10	a	17.57	0.40150	YES	YES
11	a	39.40	0.11062	YES	YES
12	a	53.16	0.26578	YES	YES
13	a	64.57	0.09205	YES	YES
14	a	68.37	0.13503	YES	YES
15	a	84.86	0.30126	YES	YES
16	a	88.58	0.21602	YES	YES
17	a	90.97	0.86229	YES	YES
18	a	101.75	0.61090	YES	YES
19	a	107.00	0.11636	YES	YES
20	a	111.52	0.04179	YES	YES
21	a	123.67	0.39127	YES	YES
22	a	166.48	2.85398	YES	YES
23	a	169.78	0.80858	YES	YES
24	a	189.44	0.55734	YES	YES
25	a	197.50	1.45027	YES	YES
26	a	205.67	1.38839	YES	YES
27	a	234.18	0.76864	YES	YES
28	a	258.19	0.93481	YES	YES
29	a	278.23	0.21455	YES	YES
30	a	294.42	2.80403	YES	YES
31	a	333.91	12.63884	YES	YES
32	a	358.35	1.03901	YES	YES
33	a	383.65	2.02331	YES	YES
34	a	400.39	5.40651	YES	YES
35	a	402.37	1.56811	YES	YES
36	a	411.18	1.44785	YES	YES
37	a	426.61	12.14994	YES	YES
38	a	435.36	0.54616	YES	YES
39	a	455.25	4.11501	YES	YES
40	a	464.66	8.39488	YES	YES
41	a	466.98	0.81202	YES	YES
42	a	476.39	0.76004	YES	YES
43	a	492.10	1.01598	YES	YES
44	a	497.58	11.50701	YES	YES
45	a	502.37	4.21196	YES	YES
46	a	521.29	1.91749	YES	YES
47	a	542.49	12.24062	YES	YES
48	a	545.04	10.18632	YES	YES
49	a	553.10	1.46029	YES	YES
50	a	564.73	0.25262	YES	YES

TS_{23bg}'

SCF Energy (au) BP86/SV(P) -2614.309477147
SCF Energy (au) PBE0/def2-TZVPP -2613.929233249
SCF Energy (au) PBE0/def2-TZVPP -2613.9365857123 (Toluene Correction)
Zero Point Energy (au) 0.2938482
Chemical Potential (kJ mol^{-1}) 615.77
Dispersion Correction (au) PBE0/def2-TZVPP -0.06657179

xyz coordinates
44

Mn	1.0446202	2.2196695	0.5499048
C	1.1981552	2.6359065	2.3093251
C	2.7832881	1.8101785	0.4800956
C	1.3941553	3.8859223	-0.0312781
O	1.6281967	4.9485527	-0.4553341
O	3.9179773	1.5340174	0.4444023
O	1.2902589	2.9124888	3.4368457
C	-1.7905760	1.4788949	1.1355548
C	-1.6847092	3.6702602	0.3070757
C	-3.0724214	3.8273872	0.3775804
C	-3.8472378	2.7460619	0.8344706
C	-3.2011110	1.5661279	1.2096823
C	-1.0016303	0.3123426	1.5382334
C	0.3728925	0.3073319	1.1399938
C	1.1861554	-0.7613311	1.5995115
C	0.6733319	-1.7655737	2.4267004
C	-0.6892004	-1.7600573	2.7968418
C	-1.5198851	-0.7277174	2.3494605
H	-1.0442952	4.4890153	-0.0557742
H	-3.5311239	4.7808319	0.0735908
H	-4.9451686	2.8235315	0.8929761
H	-3.7832541	0.6993749	1.5562845
H	2.2456846	-0.8032007	1.2981667
H	1.3391203	-2.5672179	2.7878006
H	-1.0941491	-2.5549752	3.4437029
H	-2.5744458	-0.7064679	2.6701195
N	-1.0519180	2.5341520	0.6732917
H	0.5631348	2.2112433	-2.3631115
C	0.6037548	1.7379934	-1.3765606
C	0.5771495	0.5524922	-0.8521550
C	0.7362246	-0.8300358	-1.3363065
C	1.0549481	-3.4210172	-2.4309260
C	1.9940182	-2.4157620	-2.7184140
C	1.8324125	-1.1303839	-2.1795329
C	-0.2001407	-1.8529545	-1.0541714
C	-0.0489507	-3.1298277	-1.6038352
C	1.2296801	-4.8283375	-2.9552798
H	2.8553113	-2.6375007	-3.3665245
H	2.5692766	-0.3425361	-2.4007842
H	-1.0604641	-1.6353141	-0.4029020
H	-0.7948139	-3.9112308	-1.3883314
F	1.5905075	-5.6834771	-1.9621783
F	2.1802621	-4.9093161	-3.9162865
F	0.0749786	-5.3095419	-3.4819251

$vibrational spectrum

# mode	symmetry	wave number	IR intensity	selection rules	
#		cm**(-1)	km/mol	IR	RAMAN
1	a	-255.12	0.00000	YES	YES
2		0.00	0.00000	-	-
3		0.00	0.00000	-	-
4		0.00	0.00000	-	-

5		0.00	0.00000	-	-
6		0.00	0.00000	-	-
7		0.00	0.00000	-	-
8	a	19.38	0.35992	YES	YES
9	a	27.80	0.03761	YES	YES
10	a	36.47	0.52845	YES	YES
11	a	39.58	0.21012	YES	YES
12	a	53.23	0.14211	YES	YES
13	a	64.72	0.08135	YES	YES
14	a	68.31	0.41793	YES	YES
15	a	84.55	0.02591	YES	YES
16	a	89.92	0.66128	YES	YES
17	a	98.38	0.68424	YES	YES
18	a	99.94	0.07422	YES	YES
19	a	106.78	0.31950	YES	YES
20	a	114.25	0.24696	YES	YES
21	a	134.60	0.04530	YES	YES
22	a	147.90	0.70488	YES	YES
23	a	158.95	0.91092	YES	YES
24	a	174.99	0.16022	YES	YES
25	a	205.88	0.13288	YES	YES
26	a	222.42	2.44858	YES	YES
27	a	227.63	0.35744	YES	YES
28	a	249.15	0.21159	YES	YES
29	a	278.70	1.18101	YES	YES
30	a	283.25	0.15136	YES	YES
31	a	349.26	9.91686	YES	YES
32	a	359.42	6.76641	YES	YES
33	a	383.55	2.45183	YES	YES
34	a	395.33	2.70311	YES	YES
35	a	398.54	1.47142	YES	YES
36	a	416.19	6.80839	YES	YES
37	a	430.08	0.93652	YES	YES
38	a	447.93	4.21251	YES	YES
39	a	457.31	0.25923	YES	YES
40	a	464.37	2.95976	YES	YES
41	a	475.72	0.71042	YES	YES
42	a	487.97	2.61682	YES	YES
43	a	494.87	3.04247	YES	YES
44	a	509.69	1.79537	YES	YES
45	a	521.65	1.12330	YES	YES
46	a	523.98	8.59362	YES	YES
47	a	536.63	14.48469	YES	YES
48	a	550.84	2.04644	YES	YES
49	a	557.50	1.58670	YES	YES
50	a	570.14	0.27068	YES	YES

TS_{23bh}

```
SCF Energy (au) BP86/SV(P)                   -2505.236367416
SCF Energy (au) PBE0/def2-TZVPP              -2504.785704894
SCF Energy (au) PBE0/def2-TZVPP              -2504.7950366914 (Toluene Correction)
Zero Point Energy (au)                       0.3307489
Chemical Potential (kJ mol-1)                706.50
Dispersion Correction (au) PBE0/def2-TZVPP   -0.06699044
```

```
xyz coordinates
47

Mn    1.6754286   -0.0989474    1.7320687
C     2.0246866    0.1907221    3.4826619
C     3.3761028   -0.5732624    1.4381564
C     2.0663400    1.6058582    1.2761918
O     2.3505924    2.7059428    1.0062560
O     4.4836663   -0.8920963    1.2525671
O     2.2394096    0.3678434    4.6123541
C    -1.1236676   -0.7355322    2.5456355
C    -0.9827449    1.4824181    1.7886419
C    -2.3512267    1.6956683    1.9750354
C    -3.1364053    0.6318319    2.4555878
C    -2.5185919   -0.5881900    2.7359582
C    -0.3629939   -1.9519531    2.8274500
C     0.9942964   -1.9662292    2.3738530
C     1.7871152   -3.0969841    2.7083437
C     1.2591085   -4.1680168    3.4344833
C    -0.0931520   -4.1578398    3.8456843
C    -0.8945836   -3.0551473    3.5412365
H    -0.3378479    2.2853166    1.4005864
H    -2.7881502    2.6779311    1.7388707
H    -4.2216541    0.7544255    2.6044017
H    -3.1116575   -1.4403217    3.0990710
H     2.8387260   -3.1386268    2.3775361
H     1.9051987   -5.0252121    3.6886868
H    -0.5096903   -5.0056651    4.4129586
H    -1.9400714   -3.0369371    3.8898672
N    -0.3756995    0.3062179    2.0637330
C     0.4636371    0.1596775   -1.4108760
C     1.1217846    1.2999242   -1.9468962
C     0.6946638    1.8756950   -3.1472259
C    -0.4168376    1.3493412   -3.8430565
C    -1.0859068    0.2230198   -3.3128248
C    -0.6556187   -0.3642270   -2.1220225
H     1.9853339    1.7276221   -1.4171679
H     1.2213671    2.7505785   -3.5564537
C    -0.9245870    1.9319464   -5.1192142
H    -1.9516267   -0.1796151   -3.8622928
H    -1.1828555   -1.2410020   -1.7133119
C     0.8882290   -0.4932444   -0.2000688
C     1.0969078   -1.6260932    0.3897569
H     1.3282855   -2.6646696    0.1347985
O    -0.1900944    3.0065661   -5.5221740
O    -1.8845255    1.5108534   -5.7466551
C    -0.6152721    3.6178162   -6.7452257
H    -0.5710537    2.8924294   -7.5861685
H     0.0841179    4.4583675   -6.9218394
H    -1.6584831    3.9917998   -6.6589589
```

```
$vibrational spectrum
#  mode     symmetry     wave number   IR intensity    selection rules
#                         cm**(-1)        km/mol          IR     RAMAN
     1        a            -220.95         0.00000        YES     YES
```

2		0.00	0.00000	-	-
3		0.00	0.00000	-	-
4		0.00	0.00000	-	-
5		0.00	0.00000	-	-
6		0.00	0.00000	-	-
7		0.00	0.00000	-	-
8	a	7.19	0.62059	YES	YES
9	a	17.95	0.53375	YES	YES
10	a	33.13	0.60224	YES	YES
11	a	50.45	0.43904	YES	YES
12	a	62.46	0.22710	YES	YES
13	a	65.98	0.05742	YES	YES
14	a	71.16	0.53438	YES	YES
15	a	81.74	0.48049	YES	YES
16	a	88.35	0.37958	YES	YES
17	a	91.20	0.84586	YES	YES
18	a	100.95	0.52271	YES	YES
19	a	104.34	0.18076	YES	YES
20	a	110.48	0.04179	YES	YES
21	a	114.82	0.38083	YES	YES
22	a	122.64	0.13392	YES	YES
23	a	151.79	1.20487	YES	YES
24	a	162.80	2.63536	YES	YES
25	a	172.13	1.96656	YES	YES
26	a	186.96	2.89815	YES	YES
27	a	196.98	0.65384	YES	YES
28	a	234.08	0.43297	YES	YES
29	a	243.10	2.46732	YES	YES
30	a	258.63	1.81873	YES	YES
31	a	278.49	0.18283	YES	YES
32	a	310.30	17.10652	YES	YES
33	a	330.95	7.58735	YES	YES
34	a	357.39	4.72527	YES	YES
35	a	366.76	16.85249	YES	YES
36	a	406.16	0.17136	YES	YES
37	a	410.28	2.19811	YES	YES
38	a	419.15	3.05301	YES	YES
39	a	432.70	3.35204	YES	YES
40	a	444.49	1.07229	YES	YES
41	a	458.63	2.25641	YES	YES
42	a	463.19	6.28765	YES	YES
43	a	468.90	5.35694	YES	YES
44	a	481.86	0.52039	YES	YES
45	a	494.74	3.15862	YES	YES
46	a	502.91	11.21556	YES	YES
47	a	510.39	13.39519	YES	YES
48	a	531.24	6.55006	YES	YES
49	a	544.18	13.64828	YES	YES
50	a	549.57	6.58159	YES	YES

TS_{23bh}'

```
SCF Energy (au) BP86/SV(P)                     -2505.225492213
SCF Energy (au) PBE0/def2-TZVPP                -2504.775890270
SCF Energy (au) PBE0/def2-TZVPP                -2504.7853054603 (Toluene Correction)
Zero Point Energy (au)                         0.3306400
Chemical Potential (kJ mol-1)                  709.19
Dispersion Correction (au) PBE0/def2-TZVPP    -0.06918112

xyz coordinates
47

Mn    0.9887955    2.7402352    0.7706369
C     1.0993447    3.1983377    2.5227002
C     2.7327679    2.3462166    0.7441052
C     1.3325783    4.3975058    0.1610897
O     1.5635823    5.4539063   -0.2801258
O     3.8701017    2.0799175    0.7359770
O     1.1627870    3.5019474    3.6453639
C    -1.8493390    1.9787834    1.3069888
C    -1.7496036    4.1506747    0.4281616
C    -3.1406970    4.2900659    0.4562208
C    -3.9135692    3.2089836    0.9170475
C    -3.2624680    2.0479015    1.3394548
C    -1.0562409    0.8339295    1.7602694
C     0.3267248    0.8359876    1.3934286
C     1.1423542   -0.2092920    1.8991227
C     0.6232379   -1.1958656    2.7438670
C    -0.7471626   -1.1977348    3.0836444
C    -1.5805428   -0.1897356    2.5881098
H    -1.1101961    4.9691260    0.0627906
H    -3.6031842    5.2293250    0.1159073
H    -5.0136938    3.2720352    0.9419270
H    -3.8421898    1.1812040    1.6903115
H     2.2090619   -0.2462592    1.6234060
H     1.2906992   -1.9780785    3.1427249
H    -1.1566153   -1.9788526    3.7444260
H    -2.6425742   -0.1732216    2.8834889
N    -1.1121824    3.0325985    0.8391478
H     0.5731256    2.6691468   -2.1492393
C     0.5999846    2.2146977   -1.1533827
C     0.5725363    1.0374637   -0.6096531
C     0.7569336   -0.3466514   -1.0761748
C     1.1032229   -2.9331540   -2.1894601
C     1.9936720   -1.8902474   -2.5201913
C     1.8227303   -0.6136950   -1.9733878
C    -0.1261273   -1.4013112   -0.7416334
C     0.0391434   -2.6750319   -1.2969620
C     1.3347123   -4.2748564   -2.8077003
H     2.8222398   -2.1055597   -3.2130084
H     2.5225023    0.1981937   -2.2265386
H    -0.9587920   -1.2106318   -0.0473453
H    -0.6601518   -3.4848910   -1.0415122
O     0.4172089   -5.1943266   -2.4003227
O     2.2358853   -4.5350583   -3.5888433
C     0.5740883   -6.5059498   -2.9548469
H     1.5647993   -6.9322792   -2.6864025
H     0.4918442   -6.4785578   -4.0629580
H    -0.2413344   -7.1169416   -2.5206298

$vibrational spectrum
#  mode     symmetry     wave number   IR intensity    selection rules
#                         cm**(-1)        km/mol          IR     RAMAN
     1        a            -279.75          0.00000        YES     YES
```

2		0.00	0.00000	-	-
3		0.00	0.00000	-	-
4		0.00	0.00000	-	-
5		0.00	0.00000	-	-
6		0.00	0.00000	-	-
7		0.00	0.00000	-	-
8	a	17.51	0.16059	YES	YES
9	a	24.76	0.87042	YES	YES
10	a	32.68	0.08852	YES	YES
11	a	47.97	0.12715	YES	YES
12	a	64.95	0.28703	YES	YES
13	a	67.25	0.27949	YES	YES
14	a	70.35	0.90578	YES	YES
15	a	82.07	0.00642	YES	YES
16	a	87.39	0.43598	YES	YES
17	a	94.66	0.19779	YES	YES
18	a	100.79	0.72579	YES	YES
19	a	103.57	0.34623	YES	YES
20	a	112.09	1.05407	YES	YES
21	a	118.76	0.83993	YES	YES
22	a	127.62	0.31218	YES	YES
23	a	145.61	0.61791	YES	YES
24	a	153.40	2.18467	YES	YES
25	a	166.37	1.25911	YES	YES
26	a	184.68	2.11024	YES	YES
27	a	213.11	0.34696	YES	YES
28	a	220.16	0.27071	YES	YES
29	a	238.70	4.94535	YES	YES
30	a	250.11	1.12507	YES	YES
31	a	280.11	0.16457	YES	YES
32	a	311.94	19.32206	YES	YES
33	a	342.79	4.65277	YES	YES
34	a	352.63	5.38192	YES	YES
35	a	359.74	5.76677	YES	YES
36	a	406.74	0.90136	YES	YES
37	a	409.68	8.43837	YES	YES
38	a	417.12	3.65096	YES	YES
39	a	439.06	1.80633	YES	YES
40	a	456.81	4.34689	YES	YES
41	a	463.91	1.20800	YES	YES
42	a	472.63	7.62376	YES	YES
43	a	481.41	4.75192	YES	YES
44	a	490.61	0.25573	YES	YES
45	a	496.13	1.41333	YES	YES
46	a	506.21	4.74702	YES	YES
47	a	530.13	5.95655	YES	YES
48	a	533.50	3.73254	YES	YES
49	a	537.58	8.44805	YES	YES
50	a	551.81	5.46093	YES	YES

TS_{23bi}

SCF Energy (au) BP86/SV(P)	-2508.400949832	
SCF Energy (au) PBE0/def2-TZVPP	-2507.915535463	
SCF Energy (au) PBE0/def2-TZVPP	-2507.9234924354	(Toluene Correction)
Zero Point Energy (au)	0.3685873	
Chemical Potential (kJ mol^{-1})	807.17	
Dispersion Correction (au) PBE0/def2-TZVPP	-0.08050516	

xyz coordinates
51

Mn	1.2284099	1.2096656	1.1382910
C	1.5620366	1.5138190	2.8913023
C	2.9273281	0.7258085	0.8612965
C	1.6434711	2.9064109	0.6896087
O	1.9471210	4.0056210	0.4325857
O	4.0370505	0.4075761	0.6800472
O	1.7693929	1.7061248	4.0206092
C	-1.5930377	0.6161711	1.9181808
C	-1.4230210	2.8073479	1.0951993
C	-2.7945880	3.0321862	1.2429397
C	-3.5965644	1.9873808	1.7374686
C	-2.9914033	0.7740528	2.0703341
C	-0.8419187	-0.5904372	2.2596235
C	0.5137302	-0.6407273	1.7988529
C	1.3020194	-1.7491283	2.2092470
C	0.7803488	-2.7527792	3.0305000
C	-0.5687414	-2.7094054	3.4470207
C	-1.3732091	-1.6351007	3.0566399
H	-0.7646249	3.5942705	0.6972160
H	-3.2206584	4.0087476	0.9665788
H	-4.6845633	2.1190964	1.8557951
H	-3.5965492	-0.0653700	2.4436361
H	2.3484940	-1.8228908	1.8712361
H	1.4288366	-3.5857440	3.3501090
H	-0.9813079	-3.5064344	4.0865512
H	-2.4159104	-1.5845359	3.4112702
N	-0.8284099	1.6382384	1.4219360
C	0.0945543	1.4033769	-2.0120869
C	0.6987102	2.5846759	-2.5170173
C	0.3105255	3.1154749	-3.7557732
C	-0.7014314	2.4984187	-4.5124083
C	-1.3199512	1.3340049	-4.0200295
C	-0.9286343	0.7904781	-2.7899662
H	1.4879791	3.0825054	-1.9351974
H	0.8025534	4.0277355	-4.1325043
H	-1.0100169	2.9242965	-5.4814419
H	-2.1159919	0.8413282	-4.6032712
H	-1.4131168	-0.1221004	-2.4074074
C	0.4849326	0.7919926	-0.7596752
C	0.6176539	-0.3872074	-0.2165945
C	0.9050220	-1.7370470	-0.7305169
C	1.4063271	-4.2630888	-1.9051931
C	2.2618486	-3.1800486	-2.1697585
C	2.0123522	-1.9246944	-1.5928466
C	0.0578509	-2.8380240	-0.4608263
C	0.3020509	-4.0862243	-1.0522565
H	1.6003697	-5.2471813	-2.3632658
H	3.1321351	-3.3104977	-2.8343011
H	2.6808079	-1.0744638	-1.8002598
H	-0.8045580	-2.7030931	0.2113908
H	-0.3757043	-4.9305813	-0.8428687

```
$vibrational spectrum
#  mode     symmetry     wave number   IR intensity    selection rules
#                         cm**(-1)        km/mol         IR     RAMAN
     1        a            -211.54         0.00000       YES     YES
     2                        0.00         0.00000        -       -
     3                        0.00         0.00000        -       -
     4                        0.00         0.00000        -       -
     5                        0.00         0.00000        -       -
     6                        0.00         0.00000        -       -
     7                        0.00         0.00000        -       -
     8        a              16.37         0.10076       YES     YES
     9        a              21.57         0.08172       YES     YES
    10        a              34.25         0.18295       YES     YES
    11        a              36.01         0.54213       YES     YES
    12        a              45.41         0.04774       YES     YES
    13        a              59.27         0.17249       YES     YES
    14        a              60.78         0.21687       YES     YES
    15        a              72.34         0.06198       YES     YES
    16        a              82.06         0.05727       YES     YES
    17        a              90.47         0.01476       YES     YES
    18        a              97.63         0.96153       YES     YES
    19        a             100.50         0.54914       YES     YES
    20        a             109.58         0.14237       YES     YES
    21        a             119.03         0.26936       YES     YES
    22        a             134.60         0.28199       YES     YES
    23        a             163.90         1.12153       YES     YES
    24        a             169.91         0.74455       YES     YES
    25        a             182.93         0.83635       YES     YES
    26        a             195.42         2.51480       YES     YES
    27        a             222.37         2.35535       YES     YES
    28        a             228.13         0.18482       YES     YES
    29        a             246.98         1.73153       YES     YES
    30        a             254.70         0.90979       YES     YES
    31        a             282.61         0.22690       YES     YES
    32        a             357.35         1.85677       YES     YES
    33        a             376.57         1.37678       YES     YES
    34        a             386.15         5.82901       YES     YES
    35        a             402.20         0.10506       YES     YES
    36        a             403.17         0.31570       YES     YES
    37        a             416.22         4.47644       YES     YES
    38        a             433.54         0.99814       YES     YES
    39        a             438.89         1.17057       YES     YES
    40        a             458.10         0.65946       YES     YES
    41        a             471.77         1.49662       YES     YES
    42        a             475.05         1.42520       YES     YES
    43        a             491.89         0.85399       YES     YES
    44        a             497.24         0.46976       YES     YES
    45        a             503.12         0.85840       YES     YES
    46        a             522.33        51.85901       YES     YES
    47        a             537.37         6.88208       YES     YES
    48        a             545.03         6.98089       YES     YES
    49        a             552.23         0.60839       YES     YES
    50        a             558.89         6.15442       YES     YES
```

TS_{23bj}

```
SCF Energy (au) BP86/SV(P)                    -2360.864038348
SCF Energy (au) PBE0/def2-TZVPP               -2360.429227641
SCF Energy (au) PBE0/def2-TZVPP               -2360.4352858128 (Toluene Correction)
Zero Point Energy (au)                        0.4312018
Chemical Potential (kJ mol-1)                 963.71
Dispersion Correction (au) PBE0/def2-TZVPP   -0.07320480
```

xyz coordinates
55

```
Mn    1.1107140    1.2303049    1.3664159
C     1.4536300    1.4391606    3.1321289
C     2.8216825    0.8326776    1.0473399
C     1.4253820    2.9528506    0.9656235
O     1.6383710    4.0679819    0.6816749
O     3.9410779    0.5625425    0.8435184
O     1.6673532    1.5794104    4.2692846
C    -1.6730865    0.4395534    2.1100267
C    -1.6057870    2.7156785    1.5460238
C    -2.9832332    2.8642757    1.7337919
C    -3.7346329    1.7350554    2.1086907
C    -3.0747851    0.5186962    2.2928447
C    -0.8665972   -0.7623583    2.3160031
C     0.4756185   -0.7158966    1.8125509
C     1.3177891   -1.8216256    2.1160740
C     0.8546450   -2.9182694    2.8506572
C    -0.4826357   -2.9653252    3.3041323
C    -1.3333770   -1.8882625    3.0373132
H    -0.9843578    3.5731816    1.2442892
H    -3.4533714    3.8483478    1.5829998
H    -4.8252653    1.8056594    2.2523417
H    -3.6384380   -0.3837800    2.5721509
H     2.3676456   -1.8105518    1.7788899
H     1.5448939   -3.7457250    3.0870740
H    -0.8465513   -3.8300140    3.8820871
H    -2.3633460   -1.9043005    3.4300284
N    -0.9591051    1.5423094    1.7231250
C     0.1966399    1.7365319   -1.7972506
C     0.3998843    0.9469695   -0.5521279
C     0.4971635   -0.2772839   -0.1349112
C     0.6203960   -1.6273671   -0.7939240
H     0.8380856   -1.4284989   -1.8701320
C    -0.6203238   -2.5383279   -0.6982553
H     1.5056578   -2.1689530   -0.3929317
H    -0.8285708   -2.7767922    0.3683386
C    -0.4533369   -3.8450511   -1.4918898
H    -1.5094005   -1.9814157   -1.0763700
C    -1.6815875   -4.7616080   -1.4184072
H     0.4416387   -4.3915483   -1.1109637
H    -0.2312817   -3.6059767   -2.5592191
H    -1.9058287   -5.0516221   -0.3672155
H    -1.5273465   -5.6969813   -2.0005963
H    -2.5876713   -4.2585816   -1.8259528
C     1.5184950    2.1420550   -2.4916082
H    -0.4194166    1.1371300   -2.5106304
H    -0.3910740    2.6606134   -1.5826174
H     2.1366295    2.7389659   -1.7842564
C     1.2940266    2.9459554   -3.7821047
H     2.1069365    1.2223677   -2.7176136
C     2.6003986    3.3476417   -4.4796392
H     0.7021094    3.8614864   -3.5442694
H     0.6646045    2.3496998   -4.4845199
```

```
H     3.2350785    3.9776542   -3.8167756
H     2.4033074    3.9279319   -5.4080876
H     3.2005533    2.4534281   -4.7631495

$vibrational spectrum
#  mode     symmetry     wave number   IR intensity    selection rules
#                         cm**(-1)        km/mol         IR     RAMAN
     1        a            -219.60         0.00000       YES     YES
     2                        0.00         0.00000        -       -
     3                        0.00         0.00000        -       -
     4                        0.00         0.00000        -       -
     5                        0.00         0.00000        -       -
     6                        0.00         0.00000        -       -
     7                        0.00         0.00000        -       -
     8        a               6.59         0.16626       YES     YES
     9        a              21.63         0.11480       YES     YES
    10        a              27.79         0.04135       YES     YES
    11        a              45.37         0.12128       YES     YES
    12        a              53.12         0.02177       YES     YES
    13        a              55.97         0.43534       YES     YES
    14        a              70.10         0.04223       YES     YES
    15        a              72.42         0.08127       YES     YES
    16        a              75.44         0.02293       YES     YES
    17        a              87.47         0.13587       YES     YES
    18        a              96.79         0.38697       YES     YES
    19        a              98.81         0.56577       YES     YES
    20        a             104.77         0.10079       YES     YES
    21        a             106.36         0.13085       YES     YES
    22        a             109.55         0.09909       YES     YES
    23        a             114.55         0.02645       YES     YES
    24        a             127.64         0.56038       YES     YES
    25        a             135.37         0.27014       YES     YES
    26        a             162.64         0.01310       YES     YES
    27        a             175.25         0.25308       YES     YES
    28        a             194.71         1.23061       YES     YES
    29        a             212.94         1.39155       YES     YES
    30        a             228.42         0.21344       YES     YES
    31        a             232.84         0.76318       YES     YES
    32        a             244.25         0.06565       YES     YES
    33        a             248.97         0.13199       YES     YES
    34        a             266.74         0.19040       YES     YES
    35        a             288.27         0.59620       YES     YES
    36        a             295.77         0.89610       YES     YES
    37        a             340.12         0.37202       YES     YES
    38        a             357.71         1.07517       YES     YES
    39        a             374.57         0.06299       YES     YES
    40        a             407.13         1.47999       YES     YES
    41        a             421.35         4.57145       YES     YES
    42        a             432.67         2.39157       YES     YES
    43        a             445.43         1.55153       YES     YES
    44        a             454.74         4.98736       YES     YES
    45        a             466.89         5.00115       YES     YES
    46        a             476.97         1.20250       YES     YES
    47        a             494.06         1.72617       YES     YES
    48        a             501.73         6.91228       YES     YES
    49        a             506.03         1.37450       YES     YES
    50        a             539.27         6.90485       YES     YES
```

TS_{23ca}

```
SCF Energy (au) BP86/SV(P)                     -2678.188164496
SCF Energy (au) PBE0/def2-TZVPP                -2677.701903263
SCF Energy (au) PBE0/def2-TZVPP                -2677.7129455643 (Toluene Correction)
Zero Point Energy (au)                         0.4155167
Chemical Potential (kJ mol-1)                  916.38
Dispersion Correction (au) PBE0/def2-TZVPP     -0.08439631
```

```
xyz coordinates
57

Mn    0.9098750    1.8356666   -0.2916640
C     1.2396347    1.7871900    1.4765609
C     2.6475571    1.4826921   -0.6359753
C     1.2684348    3.6041827   -0.4316902
O     1.5234693    4.7442112   -0.4771170
O     3.7804513    1.3542633   -0.8649219
O     1.4479138    1.7134857    2.6212918
C    -1.8802210    1.0356747    0.3493143
C    -1.7800896    3.3400611   -0.0736442
C    -3.1543107    3.4950630    0.1368629
C    -3.9182654    2.3601831    0.4620128
C    -3.2752138    1.1242711    0.5635844
C    -1.0968017   -0.1954282    0.4200711
C     0.2443544   -0.0788298    0.0221512
C     1.1020101   -1.2669893    0.0707704
N     0.5215603   -2.4547193    0.6223414
C    -0.8134899   -2.5505430    0.9935413
C    -1.6291399   -1.4425809    0.8747245
O     2.2686222   -1.2951855   -0.3465440
C    -1.3307536   -3.8699686    1.5038338
N    -1.1477021    2.1506025    0.0307723
C    -0.2902579    2.7328610   -3.3445501
C     0.3587554    3.9618873   -3.6353746
C    -0.1252227    4.8024539   -4.6468580
C    -1.2719680    4.4519655   -5.3818446
C    -1.9313876    3.2424671   -5.0980075
C    -1.4509279    2.3912803   -4.0941614
C     0.1940592    1.8083271   -2.3515034
C     0.4807960    0.6193957   -1.9634753
H    -1.1527441    4.2049584   -0.3367287
H    -3.6108193    4.4924433    0.0431678
H    -5.0045652    2.4403470    0.6306485
H    -3.8477972    0.2165580    0.8036044
H    -2.6803769   -1.5363013    1.1820166
H     1.2573669    4.2480346   -3.0692580
H     0.4019777    5.7466836   -4.8629965
H    -1.6512502    5.1200554   -6.1726422
H    -2.8320578    2.9567922   -5.6672691
H    -1.9685037    1.4439589   -3.8730518
H     0.8582417   -0.3508818   -2.3010040
H    -2.3920439   -3.7659231    1.8075188
H    -0.7512141   -4.2328524    2.3812089
H    -1.2788372   -4.6654035    0.7251127
C     1.4364970   -3.5980294    0.7741268
C     1.8947015   -3.8576669    2.2018580
H     2.3122862   -3.3335121    0.1421400
H     0.9677445   -4.5078727    0.3462925
C     2.8641164   -4.3778490    4.8109069
C     2.6104896   -5.4404944    3.9273811
C     2.1261966   -5.1802333    2.6345091
C     2.1530863   -2.7975710    3.0957061
C     2.6320497   -3.0567575    4.3904190
```

```
H     3.2410988   -4.5788708    5.8274084
H     2.7857707   -6.4813136    4.2470369
H     1.9267051   -6.0209632    1.9470027
H     1.9802871   -1.7558425    2.7791501
H     2.8298523   -2.2154333    5.0752334

$vibrational spectrum
#  mode     symmetry     wave number   IR intensity    selection rules
#                         cm**(-1)        km/mol         IR     RAMAN
     1        a           -158.39         0.00000        YES     YES
     2                       0.00         0.00000         -       -
     3                       0.00         0.00000         -       -
     4                       0.00         0.00000         -       -
     5                       0.00         0.00000         -       -
     6                       0.00         0.00000         -       -
     7                       0.00         0.00000         -       -
     8        a             16.31         0.16610        YES     YES
     9        a             19.21         0.14140        YES     YES
    10        a             20.70         0.05217        YES     YES
    11        a             24.15         0.13533        YES     YES
    12        a             28.69         0.60836        YES     YES
    13        a             43.50         0.11576        YES     YES
    14        a             55.93         2.17310        YES     YES
    15        a             70.63         0.18942        YES     YES
    16        a             75.08         0.18011        YES     YES
    17        a             83.27         0.01423        YES     YES
    18        a             85.27         0.56858        YES     YES
    19        a             97.48         0.55006        YES     YES
    20        a            103.95         0.30751        YES     YES
    21        a            111.46         0.39234        YES     YES
    22        a            115.39         0.63150        YES     YES
    23        a            126.26         0.45633        YES     YES
    24        a            145.84         0.24780        YES     YES
    25        a            153.38         0.50353        YES     YES
    26        a            162.83         0.10641        YES     YES
    27        a            192.28         0.83006        YES     YES
    28        a            192.55         0.52243        YES     YES
    29        a            204.96         1.16647        YES     YES
    30        a            213.33         0.26692        YES     YES
    31        a            245.14         0.49944        YES     YES
    32        a            252.28         0.39293        YES     YES
    33        a            255.49         0.28550        YES     YES
    34        a            260.15         1.10096        YES     YES
    35        a            305.23         0.85843        YES     YES
    36        a            326.12         2.04965        YES     YES
    37        a            350.42         7.12781        YES     YES
    38        a            368.62         4.52977        YES     YES
    39        a            383.52         3.78026        YES     YES
    40        a            400.95         0.02287        YES     YES
    41        a            401.52         0.22387        YES     YES
    42        a            411.81        10.99243        YES     YES
    43        a            430.02         7.43046        YES     YES
    44        a            440.22        13.87203        YES     YES
    45        a            450.45        13.59888        YES     YES
    46        a            460.14         1.30985        YES     YES
    47        a            464.12         4.29608        YES     YES
    48        a            468.65         9.67586        YES     YES
    49        a            478.93         0.85227        YES     YES
    50        a            492.04         2.00409        YES     YES
```

TS_{23ca}'

```
SCF Energy (au) BP86/SV(P)                   -2678.177478525
SCF Energy (au) PBE0/def2-TZVPP              -2677.690686045
SCF Energy (au) PBE0/def2-TZVPP              -2677.7015428126 (Toluene Correction)
Zero Point Energy (au)                       0.4152541
Chemical Potential (kJ mol-1)                916.57
Dispersion Correction (au) PBE0/def2-TZVPP -0.08703529

xyz coordinates
57

Mn    0.7138071    2.8980516   -0.5336869
C     1.1068390    2.9124074    1.2304921
C     2.4330774    2.5191573   -0.9176994
C     1.0673393    4.6427539   -0.8047677
O     1.2919132    5.7699489   -1.0160662
O     3.5587450    2.3776686   -1.1815661
O     1.3553517    2.9018747    2.3702301
C    -2.1057198    2.1386502    0.1223057
C    -1.9501941    4.4577347   -0.1693790
C    -3.3216873    4.6340174    0.0451723
C    -4.1161799    3.4991076    0.2881568
C    -3.5023884    2.2449093    0.3223966
C    -1.3448304    0.8924277    0.1766645
C    -0.0125015    0.9761958   -0.2680189
C     0.8685198   -0.1849968   -0.0775572
N     0.2665812   -1.3659019    0.4587460
C    -1.0716427   -1.4466455    0.8181379
C    -1.8769306   -0.3308541    0.6869901
O     2.0773055   -0.1866064   -0.3435158
C    -1.6044607   -2.7531520    1.3442330
N    -1.3467834    3.2502233   -0.1343219
H    -0.1032397    3.4828033   -3.3241380
C     0.0146097    2.8232305   -2.4592061
C     0.0568635    1.5599712   -2.1817017
H    -1.2997601    5.3214130   -0.3769251
H    -3.7533767    5.6464581    0.0168647
H    -5.2027305    3.5943436    0.4475574
H    -4.0977751    1.3370573    0.4986173
H    -2.9203268   -0.4004872    1.0261529
C     0.1335619    0.2634315   -2.8797375
H    -2.6641268   -2.6326257    1.6477354
H    -1.0283502   -3.1150192    2.2242253
H    -1.5641031   -3.5554622    0.5720072
C     1.1742491   -2.5126875    0.6429649
C     1.6278683   -2.7425968    2.0771552
H     2.0556937   -2.2718138    0.0110307
H     0.6944240   -3.4261753    0.2371900
C     2.5715018   -3.2122539    4.7051519
C     2.2125776   -4.2920695    3.8813847
C     1.7410718   -4.0560628    2.5787635
C     1.9956023   -1.6648834    2.9105629
C     2.4601761   -1.8991837    4.2149380
H     2.9379800   -3.3932633    5.7292854
H     2.2948495   -5.3264140    4.2551135
H     1.4581802   -4.9098036    1.9380594
H     1.9264470   -0.6309753    2.5351710
H     2.7442427   -1.0452621    4.8521320
C     0.2436835   -2.1439634   -4.3543183
C     1.4121581   -1.3909553   -4.1411569
C     1.3624037   -0.1939805   -3.4101481
C    -1.0348033   -0.5068422   -3.0829668
C    -0.9801775   -1.6970952   -3.8257625
```

```
H     0.2866425   -3.0809266   -4.9343947
H     2.3755773   -1.7373742   -4.5504159
H     2.2753711    0.3951156   -3.2379028
H    -1.9912614   -0.1537877   -2.6662400
H    -1.9018648   -2.2788318   -3.9939954

$vibrational spectrum
#  mode     symmetry     wave number   IR intensity    selection rules
#                         cm**(-1)        km/mol         IR     RAMAN
     1        a            -281.77         0.00000       YES     YES
     2                        0.00         0.00000        -       -
     3                        0.00         0.00000        -       -
     4                        0.00         0.00000        -       -
     5                        0.00         0.00000        -       -
     6                        0.00         0.00000        -       -
     7                        0.00         0.00000        -       -
     8        a              16.92         0.11677       YES     YES
     9        a              22.81         0.48793       YES     YES
    10        a              23.35         0.50714       YES     YES
    11        a              27.88         0.38305       YES     YES
    12        a              35.47         0.34503       YES     YES
    13        a              41.64         0.12674       YES     YES
    14        a              45.75         0.06490       YES     YES
    15        a              64.64         1.23398       YES     YES
    16        a              79.99         0.08681       YES     YES
    17        a              81.42         0.30846       YES     YES
    18        a              86.23         0.18885       YES     YES
    19        a              86.86         2.02877       YES     YES
    20        a             100.20         0.10912       YES     YES
    21        a             112.65         1.15652       YES     YES
    22        a             117.48         0.75015       YES     YES
    23        a             127.85         0.27572       YES     YES
    24        a             145.38         0.60008       YES     YES
    25        a             159.80         0.21392       YES     YES
    26        a             176.84         5.07115       YES     YES
    27        a             185.84         0.24957       YES     YES
    28        a             191.89         0.82374       YES     YES
    29        a             198.90         2.00031       YES     YES
    30        a             206.45         2.92257       YES     YES
    31        a             229.40         0.29347       YES     YES
    32        a             239.23         0.17811       YES     YES
    33        a             250.82         0.28528       YES     YES
    34        a             260.98         0.06373       YES     YES
    35        a             293.01         0.12201       YES     YES
    36        a             319.69         4.39261       YES     YES
    37        a             337.75         4.13786       YES     YES
    38        a             364.47         3.52406       YES     YES
    39        a             387.66         2.19276       YES     YES
    40        a             400.14         0.42898       YES     YES
    41        a             401.41         0.15064       YES     YES
    42        a             426.70         1.66304       YES     YES
    43        a             441.21        13.80089       YES     YES
    44        a             447.88         0.16682       YES     YES
    45        a             456.14         2.72252       YES     YES
    46        a             463.93         2.63819       YES     YES
    47        a             466.75         2.08337       YES     YES
    48        a             478.66         1.74876       YES     YES
    49        a             489.42         2.32141       YES     YES
    50        a             498.68         7.04635       YES     YES
```

TS_{23da}

SCF Energy (au) BP86/SV(P)	-2408.995112972
SCF Energy (au) PBE0/def2-TZVPP	-2408.528811114
SCF Energy (au) PBE0/def2-TZVPP	-2408.5368518917 (Toluene Correction)
Zero Point Energy (au)	0.3176652
Chemical Potential (kJ mol^{-1})	684.72
Dispersion Correction (au) PBE0/def2-TZVPP	-0.06668154

xyz coordinates
45

Mn	1.7275223	0.7999167	0.3898309
C	2.0895483	1.0816028	2.1407390
C	3.4216148	0.2952688	0.0980376
C	2.1216996	2.5065434	-0.0548414
O	2.3942681	3.6123101	-0.3122392
O	4.5224521	-0.0452347	-0.0826326
O	2.3138259	1.2506852	3.2695325
C	-1.0696555	0.1494737	1.1735573
C	-0.9452402	2.3772016	0.4726763
C	-2.3206936	2.5655412	0.6310084
C	-3.0971991	1.4675657	1.0421106
C	-2.4752166	0.2452548	1.3058193
N	-0.3278659	-0.9901403	1.4304791
C	1.0302861	-1.0331570	0.9837798
C	1.5669268	-2.2391538	1.4195374
C	0.5609475	-2.9614117	2.1463357
C	-0.6310173	-2.1594269	2.1567726
H	-0.3007874	3.1946902	0.1159326
H	-2.7694426	3.5462976	0.4137220
H	-4.1910040	1.5577266	1.1436460
H	-3.0675080	-0.6361346	1.5774146
H	2.5910470	-2.5809008	1.2200111
C	-1.7803764	-2.5716864	2.8541962
N	-0.3242258	1.2063843	0.7356993
C	0.6087933	1.1297135	-2.7764697
C	1.3596298	2.2162507	-3.2967806
C	0.9857201	2.8322139	-4.4990762
C	-0.1538459	2.4000883	-5.2007365
C	-0.9146119	1.3319305	-4.6916026
C	-0.5397590	0.7000288	-3.4987574
H	2.2522018	2.5678695	-2.7584966
H	1.5912388	3.6665749	-4.8906988
H	-0.4499531	2.8952950	-6.1401784
H	-1.8107552	0.9834489	-5.2321667
H	-1.1366376	-0.1372632	-3.1019966
C	0.9754901	0.4321249	-1.5662732
C	1.1101977	-0.7218231	-1.0021441
H	1.2974738	-1.7667095	-1.2600597
C	-1.7454693	-3.8123328	3.5148773
H	-2.6863943	-1.9549698	2.9228964
H	-2.6384791	-4.1486237	4.0663696
C	-0.5891616	-4.6246356	3.4900499
H	-0.5951833	-5.5931240	4.0167712
C	0.5654396	-4.2057824	2.8175284
H	1.4741595	-4.8294912	2.8158191

$vibrational spectrum

# mode	symmetry	wave number	IR intensity	selection rules	
#		cm**(-1)	km/mol	IR	RAMAN
1	a	-237.37	0.00000	YES	YES
2		0.00	0.00000	-	-
3		0.00	0.00000	-	-

4		0.00	0.00000	-	-
5		0.00	0.00000	-	-
6		0.00	0.00000	-	-
7		0.00	0.00000	-	-
8	a	16.90	0.00794	YES	YES
9	a	27.26	0.08611	YES	YES
10	a	33.16	0.11095	YES	YES
11	a	54.27	0.16625	YES	YES
12	a	68.89	0.30671	YES	YES
13	a	73.22	0.49740	YES	YES
14	a	76.42	0.13529	YES	YES
15	a	84.67	0.22794	YES	YES
16	a	93.42	0.06507	YES	YES
17	a	100.15	0.60557	YES	YES
18	a	107.55	0.04497	YES	YES
19	a	128.61	0.17288	YES	YES
20	a	137.13	0.25235	YES	YES
21	a	158.03	4.13126	YES	YES
22	a	169.02	0.72987	YES	YES
23	a	200.40	1.72411	YES	YES
24	a	211.16	2.18243	YES	YES
25	a	236.27	3.70321	YES	YES
26	a	239.25	2.39867	YES	YES
27	a	250.95	1.06395	YES	YES
28	a	300.23	1.10439	YES	YES
29	a	336.42	8.93386	YES	YES
30	a	362.82	4.04049	YES	YES
31	a	364.60	3.32119	YES	YES
32	a	400.79	6.52216	YES	YES
33	a	402.21	0.08097	YES	YES
34	a	425.77	8.35193	YES	YES
35	a	433.33	1.36559	YES	YES
36	a	451.84	7.72534	YES	YES
37	a	467.18	1.43404	YES	YES
38	a	482.53	1.07415	YES	YES
39	a	489.01	7.91623	YES	YES
40	a	497.36	1.87821	YES	YES
41	a	502.11	2.52264	YES	YES
42	a	513.86	2.19350	YES	YES
43	a	528.63	4.72170	YES	YES
44	a	536.72	19.06779	YES	YES
45	a	546.95	16.32610	YES	YES
46	a	566.74	0.55150	YES	YES
47	a	573.19	9.51230	YES	YES
48	a	590.52	8.76627	YES	YES
49	a	605.37	8.55967	YES	YES
50	a	611.88	3.41869	YES	YES

TS_{23da}′

```
SCF Energy (au) BP86/SV(P)                    -2408.986641492
SCF Energy (au) PBE0/def2-TZVPP               -2408.520986012
SCF Energy (au) PBE0/def2-TZVPP               -2408.5290620226 (Toluene Correction)
Zero Point Energy (au)                        0.3174132
Chemical Potential (kJ mol-1)                 684.77
Dispersion Correction (au) PBE0/def2-TZVPP   -0.06880878

xyz coordinates
45

Mn    1.3929405    2.1179321    0.0346053
C     1.5920354    2.4758324    1.8024534
C     3.1193805    1.6611721   -0.0761235
C     1.7583553    3.8001150   -0.4949705
O     1.9946440    4.8771940   -0.8799786
O     4.2431992    1.3514463   -0.1390355
O     1.7222591    2.7126864    2.9351880
C    -1.4348519    1.3721376    0.6037112
C    -1.3380177    3.5979133   -0.0999468
C    -2.7269111    3.7407853   -0.0340012
C    -3.4928823    2.6191133    0.3312722
C    -2.8484693    1.4195798    0.6425246
N    -0.6766299    0.2559087    0.9168210
C     0.7030024    0.2367931    0.5363423
C     1.2498842   -0.9340909    1.0457297
C     0.2394481   -1.6424615    1.7795163
C    -0.9732203   -0.8764538    1.7014801
H    -0.6975913    4.4361886   -0.4145309
H    -3.1929470    4.7058657   -0.2834772
H    -4.5933515    2.6730763    0.3596596
H    -3.4263285    0.5176568    0.8777316
H     2.2870153   -1.2597861    0.8958340
C    -2.1347038   -1.2771142    2.3859537
N    -0.6966783    2.4499950    0.2097932
H     0.8708690    2.2675579   -2.8666379
C     0.9239950    1.7187705   -1.9211631
C     0.8921691    0.5014069   -1.4887468
C     1.0615201   -0.8629432   -2.0061543
C    -2.0883262   -2.4734346    3.1229722
H    -3.0576234   -0.6815185    2.3865140
H    -2.9892199   -2.8001588    3.6674636
C    -0.9111694   -3.2539717    3.1835882
H    -0.9094097   -4.1884285    3.7685879
C     0.2537007   -2.8445431    2.5232639
H     1.1781725   -3.4410599    2.5878421
C     1.3873297   -3.4133154   -3.1850301
C     2.3456125   -2.4095796   -3.4036839
C     2.1833224   -1.1409638   -2.8239487
C     0.1079126   -1.8843728   -1.7845339
C     0.2664994   -3.1444326   -2.3776991
H     1.5134272   -4.4086686   -3.6423504
H     3.2295294   -2.6135106   -4.0305493
H     2.9334823   -0.3513558   -2.9890131
H    -0.7698230   -1.6806500   -1.1517711
H    -0.4915512   -3.9263131   -2.2055020

$vibrational spectrum
#  mode     symmetry     wave number   IR intensity    selection rules
#                         cm**(-1)        km/mol          IR     RAMAN
     1        a            -287.82        0.00000        YES     YES
     2                        0.00        0.00000         -       -
```

3		0.00	0.00000	-	-
4		0.00	0.00000	-	-
5		0.00	0.00000	-	-
6		0.00	0.00000	-	-
7		0.00	0.00000	-	-
8	a	20.67	0.60655	YES	YES
9	a	28.74	0.41890	YES	YES
10	a	42.36	0.06185	YES	YES
11	a	48.42	0.04076	YES	YES
12	a	62.97	0.44129	YES	YES
13	a	71.63	0.08685	YES	YES
14	a	77.65	0.26895	YES	YES
15	a	86.77	0.22815	YES	YES
16	a	89.66	0.18222	YES	YES
17	a	96.58	0.02826	YES	YES
18	a	102.25	0.40104	YES	YES
19	a	131.02	0.25623	YES	YES
20	a	132.36	0.74006	YES	YES
21	a	161.35	2.79207	YES	YES
22	a	176.38	7.58770	YES	YES
23	a	197.25	1.11695	YES	YES
24	a	205.64	1.84132	YES	YES
25	a	228.37	3.45227	YES	YES
26	a	233.19	0.79052	YES	YES
27	a	255.52	1.79123	YES	YES
28	a	300.11	0.94208	YES	YES
29	a	326.31	4.03321	YES	YES
30	a	363.15	4.81019	YES	YES
31	a	367.03	1.79414	YES	YES
32	a	402.97	0.07985	YES	YES
33	a	416.28	5.42703	YES	YES
34	a	433.86	0.86468	YES	YES
35	a	456.32	1.84925	YES	YES
36	a	458.93	1.96833	YES	YES
37	a	474.37	5.79367	YES	YES
38	a	484.31	0.73568	YES	YES
39	a	493.32	9.72999	YES	YES
40	a	497.86	19.72214	YES	YES
41	a	504.56	1.35928	YES	YES
42	a	516.71	2.70257	YES	YES
43	a	530.08	20.04411	YES	YES
44	a	538.14	5.12531	YES	YES
45	a	554.22	11.05044	YES	YES
46	a	555.69	18.32082	YES	YES
47	a	567.67	1.46299	YES	YES
48	a	591.43	10.61040	YES	YES
49	a	611.96	3.40366	YES	YES
50	a	613.21	0.62181	YES	YES

TS_{23ea}

SCF Energy (au) BP86/SV(P)	-2583.753044495	
SCF Energy (au) PBE0/def2-TZVPP	-2583.299586047	
SCF Energy (au) PBE0/def2-TZVPP	-2583.3092723309	(Toluene Correction)
Zero Point Energy (au)	0.3857030	
Chemical Potential (kJ mol^{-1})	843.61	
Dispersion Correction (au) PBE0/def2-TZVPP	-0.07220089	

xyz coordinates
53

Mn	2.3833058	0.8483205	0.3108484
C	2.6727450	1.1691657	2.0615271
C	4.1166155	0.4487440	0.0725329
C	2.6466621	2.5783517	-0.1538424
O	2.8190904	3.7029692	-0.4141811
O	5.2432486	0.1718222	-0.0696683
O	2.8494658	1.3606284	3.1967976
C	-0.4004547	0.1554101	0.9220468
C	-1.8785234	0.2888813	0.9662264
C	0.3311949	-1.0383896	1.3080514
C	1.7274833	-1.0184595	0.9764134
C	2.5353600	-2.1001426	1.4115396
C	1.9793464	-3.1718278	2.1320991
C	0.5878700	-3.1897654	2.4353188
C	-0.2173080	-2.1328742	2.0301947
H	3.6098354	-2.0926353	1.1731258
O	2.6855051	-4.2349131	2.6000951
H	0.1863604	-4.0397473	3.0079205
H	-1.2853858	-2.1288864	2.3014784
N	0.3690596	1.1381350	0.5004391
C	1.2656167	0.9684925	-2.9026942
C	1.7993636	2.1870718	-3.3979245
C	1.3830038	2.7006197	-4.6345102
C	0.4137082	2.0276644	-5.3995675
C	-0.1341568	0.8245695	-4.9168780
C	0.2844428	0.2986441	-3.6880383
H	2.5531545	2.7282996	-2.8080934
H	1.8191197	3.6441258	-5.0027592
H	0.0829049	2.4402888	-6.3669099
H	-0.8972491	0.2883857	-5.5058238
H	-0.1471660	-0.6420084	-3.3100178
C	1.6874677	0.3688457	-1.6591208
C	1.9467988	-0.7307784	-1.0314793
H	2.2581332	-1.7603291	-1.2274445
C	-4.7037107	0.6189425	0.9711380
C	-4.1194987	-0.5712115	0.4755624
C	-2.7333012	-0.7332381	0.4793554
C	-2.4747491	1.4726042	1.4537188
C	-3.8675218	1.6450218	1.4648803
O	-6.0608174	0.6777296	0.9309396
H	-4.7856424	-1.3557750	0.0837907
H	-2.2967469	-1.6576109	0.0685523
H	-1.8356938	2.2731621	1.8627868
H	-4.2889202	2.5770713	1.8687505
H	-0.1328790	1.9651192	0.1475545
C	4.0842964	-4.2897159	2.3632922
C	-6.7122521	1.8464633	1.4041348
H	4.6123053	-3.4217927	2.8227643
H	4.3166356	-4.3214205	1.2730434
H	4.4385375	-5.2266522	2.8382625
H	-6.5048590	2.0246182	2.4856943
H	-7.7977912	1.6687913	1.2676145

```
H    -6.4140099    2.7492149    0.8204629

$vibrational spectrum
#  mode     symmetry     wave number   IR intensity    selection rules
#                         cm**(-1)        km/mol         IR     RAMAN
     1        a            -208.55         0.00000       YES     YES
     2                        0.00         0.00000        -       -
     3                        0.00         0.00000        -       -
     4                        0.00         0.00000        -       -
     5                        0.00         0.00000        -       -
     6                        0.00         0.00000        -       -
     7                        0.00         0.00000        -       -
     8        a              15.47         0.07161       YES     YES
     9        a              16.51         0.10232       YES     YES
    10        a              33.01         0.47225       YES     YES
    11        a              38.56         0.38207       YES     YES
    12        a              43.92         0.70894       YES     YES
    13        a              46.39         0.32732       YES     YES
    14        a              61.18         0.20756       YES     YES
    15        a              73.20         0.46680       YES     YES
    16        a              75.48         0.18658       YES     YES
    17        a              86.94         0.06247       YES     YES
    18        a              89.04         0.29431       YES     YES
    19        a              97.18         0.31965       YES     YES
    20        a             105.22         2.00191       YES     YES
    21        a             113.00         0.98813       YES     YES
    22        a             125.65         0.79241       YES     YES
    23        a             134.73         1.05068       YES     YES
    24        a             151.30         1.78706       YES     YES
    25        a             160.31         1.44935       YES     YES
    26        a             179.53         0.32215       YES     YES
    27        a             188.52         0.23415       YES     YES
    28        a             197.77         0.33095       YES     YES
    29        a             213.48         0.36455       YES     YES
    30        a             233.72         0.26149       YES     YES
    31        a             241.14         0.49848       YES     YES
    32        a             260.07         0.76530       YES     YES
    33        a             265.83         2.10419       YES     YES
    34        a             297.40         3.78161       YES     YES
    35        a             321.18         0.63206       YES     YES
    36        a             338.79         4.50691       YES     YES
    37        a             342.71         7.05066       YES     YES
    38        a             367.37         3.38791       YES     YES
    39        a             401.37         1.93286       YES     YES
    40        a             402.87         0.12617       YES     YES
    41        a             416.51         0.58616       YES     YES
    42        a             418.70         2.95551       YES     YES
    43        a             435.27         1.24917       YES     YES
    44        a             449.98         3.46461       YES     YES
    45        a             454.81         2.85799       YES     YES
    46        a             468.62        10.92685       YES     YES
    47        a             482.98         0.52244       YES     YES
    48        a             493.07         3.92736       YES     YES
    49        a             499.16         7.85117       YES     YES
    50        a             501.98         5.34270       YES     YES
```

TS_{23ea}′

SCF Energy (au) BP86/SV(P)	-2583.738258985
SCF Energy (au) PBE0/def2-TZVPP	-2583.285628368
SCF Energy (au) PBE0/def2-TZVPP	-2583.2957410864 (Toluene Correction)
Zero Point Energy (au)	0.3849902
Chemical Potential (kJ mol^{-1})	841.35
Dispersion Correction (au) PBE0/def2-TZVPP	-0.07451576

xyz coordinates
53

Mn	2.1746510	1.9645105	-0.4690860
C	2.6450634	2.4259098	1.2145880
C	3.8781896	1.5626495	-0.8563270
C	2.3534054	3.6185339	-1.1566146
O	2.4489731	4.6785438	-1.6355252
O	4.9897225	1.2963138	-1.1038729
O	2.9325961	2.7213873	2.3051651
C	-0.5284013	1.3313376	0.5157799
C	0.2253290	0.1323297	0.8434505
C	1.5604853	0.0882302	0.2933244
C	2.4022337	-0.9653433	0.7115988
C	1.9613766	-1.9532893	1.6126250
C	0.6165517	-1.9494312	2.0721544
C	-0.2359884	-0.9152057	1.6749787
H	3.4304513	-1.0539457	0.3266320
O	2.8839293	-2.8864229	1.9698074
H	0.2372654	-2.7409785	2.7340992
H	-1.2772839	-0.9105281	2.0351930
N	0.1895135	2.2659540	-0.0653128
H	1.0940955	1.7766872	-3.2253331
C	1.3346492	1.3755574	-2.2357727
C	1.5101649	0.2213884	-1.6611739
C	1.7568852	-1.1496442	-2.1513247
C	2.1801818	-3.7175437	-3.2623512
C	3.0246732	-2.6504279	-3.6120825
C	2.8127054	-1.3738298	-3.0662641
C	0.9208420	-2.2337256	-1.7958153
C	1.1257378	-3.5032770	-2.3559522
H	2.3440281	-4.7186166	-3.6948865
H	3.8567578	-2.8102906	-4.3178720
H	3.4747769	-0.5355053	-3.3349262
H	0.0965800	-2.0672438	-1.0839022
H	0.4559344	-4.3353256	-2.0814602
H	-0.3288160	3.1216373	-0.3112567
C	-1.9823949	1.5233350	0.7739732
C	-4.7489168	1.9946365	1.2280657
C	-3.8909273	1.6593259	2.3018371
C	-2.5326718	1.4245519	2.0765794
C	-2.8455695	1.8705045	-0.2867300
C	-4.2146754	2.1003417	-0.0747566
O	-6.0547404	2.1985750	1.5494690
H	-4.3200517	1.5997385	3.3143149
H	-1.8771432	1.1958545	2.9321193
H	-2.4418454	1.9427006	-1.3104591
H	-4.8538629	2.3556583	-0.9324763
C	-6.9667337	2.5488890	0.5205927
H	-7.0386567	1.7516628	-0.2567495
H	-6.6867477	3.5119097	0.0316236
H	-7.9533948	2.6655222	1.0119679
C	2.5211775	-3.9033562	2.8892150
H	1.7176168	-4.5622496	2.4824823
H	2.1870458	-3.4786642	3.8650232

```
H     3.4352328   -4.5093316    3.0516238

$vibrational spectrum
#  mode     symmetry     wave number   IR intensity    selection rules
#                         cm**(-1)        km/mol         IR     RAMAN
     1        a            -296.98         0.00000       YES     YES
     2                        0.00         0.00000        -       -
     3                        0.00         0.00000        -       -
     4                        0.00         0.00000        -       -
     5                        0.00         0.00000        -       -
     6                        0.00         0.00000        -       -
     7                        0.00         0.00000        -       -
     8        a              18.22         0.22944       YES     YES
     9        a              24.31         0.09629       YES     YES
    10        a              29.57         0.35562       YES     YES
    11        a              32.77         0.12955       YES     YES
    12        a              43.30         0.28450       YES     YES
    13        a              48.40         1.17254       YES     YES
    14        a              52.25         0.33172       YES     YES
    15        a              65.12         0.13582       YES     YES
    16        a              78.99         0.14413       YES     YES
    17        a              85.48         0.13030       YES     YES
    18        a              86.69         0.50394       YES     YES
    19        a              94.09         0.75043       YES     YES
    20        a              99.10         2.62791       YES     YES
    21        a             100.03         0.58182       YES     YES
    22        a             125.70         0.20917       YES     YES
    23        a             143.38         0.32313       YES     YES
    24        a             145.97         0.79017       YES     YES
    25        a             152.13         1.76887       YES     YES
    26        a             177.98         0.13994       YES     YES
    27        a             193.14         1.65087       YES     YES
    28        a             203.26         3.59753       YES     YES
    29        a             208.08         0.83531       YES     YES
    30        a             231.47         1.19931       YES     YES
    31        a             239.87         1.29797       YES     YES
    32        a             244.45         2.03842       YES     YES
    33        a             253.73         1.06308       YES     YES
    34        a             288.48         1.17820       YES     YES
    35        a             306.57         4.12109       YES     YES
    36        a             314.39         1.27095       YES     YES
    37        a             332.66         0.94044       YES     YES
    38        a             355.46         0.48843       YES     YES
    39        a             402.18         0.02761       YES     YES
    40        a             411.11         4.82695       YES     YES
    41        a             412.86         3.42446       YES     YES
    42        a             423.23         0.47428       YES     YES
    43        a             452.15         0.15110       YES     YES
    44        a             459.15         2.51377       YES     YES
    45        a             466.24         2.50035       YES     YES
    46        a             479.31         5.60076       YES     YES
    47        a             491.13         4.47747       YES     YES
    48        a             495.62         4.84184       YES     YES
    49        a             499.16         4.12463       YES     YES
    50        a             512.83         3.08282       YES     YES
```

TS_{23ej}

SCF Energy (au) BP86/SV(P)	-2814.645959297	
SCF Energy (au) PBE0/def2-TZVPP	-2814.155882277	
SCF Energy (au) PBE0/def2-TZVPP	-2814.1661111849	(Toluene Correction)
Zero Point Energy (au)	0.4648176	
Chemical Potential (kJ mol^{-1})	1034.42	
Dispersion Correction (au) PBE0/def2-TZVPP	-0.09273178	

xyz coordinates
63

Mn	1.9306911	1.4020002	0.6830458
C	2.1838181	1.7355158	2.4392325
C	3.6641337	0.9860875	0.4910235
C	2.2510348	3.1212471	0.2232937
O	2.4712664	4.2408521	-0.0256646
O	4.7959451	0.7116684	0.3802884
O	2.3372093	1.9384693	3.5758549
C	-0.8818569	0.7680615	1.2417821
C	-2.3576784	0.9247761	1.2413331
C	-0.1766581	-0.4235535	1.6783525
C	1.2250130	-0.4418934	1.3598469
C	2.0102907	-1.5083179	1.8635362
C	1.4349860	-2.5222176	2.6499970
C	0.0410310	-2.5039740	2.9385402
C	-0.7481153	-1.4663571	2.4562785
H	3.0839960	-1.5361476	1.6273815
O	2.1235925	-3.5612119	3.1902086
H	-0.3769380	-3.3106860	3.5600084
H	-1.8189575	-1.4320486	2.7145749
N	-0.0842866	1.7267082	0.8160727
C	0.8260154	1.4855016	-2.5115280
C	1.2304284	2.7687591	-2.9640986
C	0.8216903	3.2503419	-4.2164899
C	-0.0126781	2.4769118	-5.0424273
C	-0.4330863	1.2069388	-4.6039751
C	-0.0216378	0.7153266	-3.3591703
H	1.8769689	3.3893508	-2.3273141
H	1.1563737	4.2473776	-4.5482167
H	-0.3388016	2.8627253	-6.0224091
H	-1.0902393	0.5914373	-5.2412450
H	-0.3540159	-0.2781091	-3.0184706
C	1.2445243	0.9213366	-1.2469878
C	1.4821055	-0.2274950	-0.6770971
C	1.9529371	-1.5431340	-1.1375304
C	-5.1764461	1.2964760	1.1526703
C	-4.5985342	0.0748947	0.7319111
C	-3.2157782	-0.1069035	0.7812849
C	-2.9480030	2.1390221	1.6560944
C	-4.3374039	2.3326881	1.6198692
O	-6.5306659	1.3738259	1.0713401
H	-5.2665135	-0.7177467	0.3601128
H	-2.7833855	-1.0560987	0.4266622
H	-2.3076940	2.9487000	2.0447980
H	-4.7543672	3.2893905	1.9665970
H	-0.5606966	2.5486784	0.4189116
C	3.5214601	-3.6573375	2.9519756
C	-7.1755115	2.5746779	1.4668446
H	4.0669060	-2.7732336	3.3570887
H	3.7460199	-3.7599460	1.8653516
H	3.8590608	-4.5694592	3.4840392
H	-6.9955334	2.8036794	2.5436660
H	-8.2595994	2.4068834	1.3080940

```
H    -6.8460474    3.4422745    0.8477132
C     2.8241651   -4.0194464   -2.2026938
C     3.5730824   -2.8515251   -2.4278871
C     3.1401779   -1.6223110   -1.9054453
C     1.2125691   -2.7276841   -0.9091549
C     1.6404862   -3.9514904   -1.4459562
H     3.1613463   -4.9833076   -2.6190666
H     4.5024448   -2.8937399   -3.0201148
H     3.7255107   -0.7070230   -2.0840277
H     0.2858002   -2.6785507   -0.3153716
H     1.0435503   -4.8619399   -1.2700714

$vibrational spectrum
#  mode     symmetry     wave number   IR intensity    selection rules
#                         cm**(-1)        km/mol         IR     RAMAN
     1        a           -194.57         0.00000       YES     YES
     2                       0.00         0.00000        -       -
     3                       0.00         0.00000        -       -
     4                       0.00         0.00000        -       -
     5                       0.00         0.00000        -       -
     6                       0.00         0.00000        -       -
     7                       0.00         0.00000        -       -
     8        a             16.41         0.09106       YES     YES
     9        a             18.69         0.13939       YES     YES
    10        a             24.84         0.06883       YES     YES
    11        a             32.84         0.18714       YES     YES
    12        a             36.53         0.67747       YES     YES
    13        a             43.45         0.75136       YES     YES
    14        a             45.03         0.09858       YES     YES
    15        a             49.04         0.02101       YES     YES
    16        a             57.92         0.23317       YES     YES
    17        a             63.05         0.29982       YES     YES
    18        a             74.95         0.57612       YES     YES
    19        a             76.87         0.04162       YES     YES
    20        a             86.54         0.07244       YES     YES
    21        a             92.38         0.33303       YES     YES
    22        a             98.55         0.34466       YES     YES
    23        a            106.72         2.23100       YES     YES
    24        a            114.66         0.40203       YES     YES
    25        a            124.04         0.64485       YES     YES
    26        a            137.05         1.94375       YES     YES
    27        a            150.70         1.72182       YES     YES
    28        a            160.05         1.53666       YES     YES
    29        a            168.98         0.45894       YES     YES
    30        a            186.71         1.45702       YES     YES
    31        a            189.01         0.60705       YES     YES
    32        a            198.14         2.37796       YES     YES
    33        a            222.80         0.28299       YES     YES
    34        a            230.16         0.23786       YES     YES
    35        a            237.45         1.45215       YES     YES
    36        a            249.75         1.01148       YES     YES
    37        a            253.15         0.14093       YES     YES
    38        a            261.15         2.60947       YES     YES
    39        a            294.73         4.15721       YES     YES
    40        a            323.23         3.05680       YES     YES
    41        a            339.69         0.67976       YES     YES
    42        a            357.65         4.33796       YES     YES
    43        a            374.48         0.60465       YES     YES
    44        a            398.95         3.36470       YES     YES
    45        a            402.20         0.12622       YES     YES
    46        a            405.90         2.63261       YES     YES
    47        a            416.83         0.45536       YES     YES
    48        a            419.15         2.87869       YES     YES
```

49	a	435.25	1.41876	YES	YES
50	a	450.38	0.19487	YES	YES

TS_{23fa}

```
SCF Energy (au) BP86/SV(P)                    -2391.953906106
SCF Energy (au) PBE0/def2-TZVPP               -2391.506807198
SCF Energy (au) PBE0/def2-TZVPP               -2391.5145469127 (Toluene Correction)
Zero Point Energy (au)                        0.3208594
Chemical Potential (kJ mol-1)                 692.48
Dispersion Correction (au) PBE0/def2-TZVPP -0.06636323

xyz coordinates
45

Mn    1.6823049   -0.1540813    0.7119304
C     2.0823667    0.0299876    2.4662496
C     3.3142989   -0.7703630    0.3633362
C     2.2916770    1.5080239    0.3104084
O     2.8051040    2.5272816    0.0645810
O     4.3832846   -1.1888982    0.1417157
O     2.3252325    0.1410141    3.5993230
C    -1.1529180   -0.6715825    1.5897830
C    -0.9430170    1.5831402    0.9670982
C    -2.3147607    1.8220671    1.1899400
C    -3.1142375    0.7522986    1.6168280
C    -2.5383478   -0.5024504    1.8168618
C    -0.4436773   -1.9368414    1.7767137
C     0.8891064   -2.0045914    1.2620044
C     1.6189977   -3.2041245    1.4916431
C     1.0557362   -4.2830285    2.1764479
C    -0.2732070   -4.2137822    2.6538585
C    -1.0127998   -3.0473244    2.4508552
O    -0.0854716    2.5354261    0.5597101
H    -2.7477230    2.8191459    1.0333578
H    -4.1919682    0.9070197    1.7894065
H    -3.1526392   -1.3556750    2.1355549
H     2.6507360   -3.2910846    1.1112435
H     1.6561477   -5.1924837    2.3484406
H    -0.7180477   -5.0658746    3.1926922
H    -2.0376043   -2.9848091    2.8513895
N    -0.3729834    0.3672597    1.1560503
C     0.3526155    0.3818633   -2.3524857
C     0.9943495    1.5649540   -2.8046371
C     0.5520736    2.2219353   -3.9617036
C    -0.5528760    1.7362858   -4.6840251
C    -1.2105295    0.5744263   -4.2394100
C    -0.7656216   -0.0968195   -3.0932606
H     1.8555215    1.9605836   -2.2470897
H     1.0769613    3.1301816   -4.3027952
H    -0.9020385    2.2615575   -5.5884173
H    -2.0800711    0.1837491   -4.7945666
H    -1.2829623   -1.0061058   -2.7462792
C     0.7881228   -0.3593225   -1.1930104
C     0.9329506   -1.5439861   -0.6899098
H     1.0768716   -2.5764308   -1.0226138
C    -0.5505492    3.8496392    0.2821474
H    -1.3018194    3.8476272   -0.5401194
H     0.3453502    4.4159264   -0.0394656
H    -0.9839389    4.3282658    1.1902184

$vibrational spectrum
#  mode     symmetry     wave number   IR intensity    selection rules
#                         cm**(-1)        km/mol          IR     RAMAN
     1        a            -228.81        0.00000        YES     YES
     2                        0.00        0.00000         -       -
     3                        0.00        0.00000         -       -
```

4		0.00	0.00000	-	-
5		0.00	0.00000	-	-
6		0.00	0.00000	-	-
7		0.00	0.00000	-	-
8	a	19.11	0.00795	YES	YES
9	a	25.55	0.42080	YES	YES
10	a	36.80	0.24743	YES	YES
11	a	50.02	0.01194	YES	YES
12	a	62.78	0.35860	YES	YES
13	a	74.19	1.54238	YES	YES
14	a	88.21	0.46670	YES	YES
15	a	90.75	0.18401	YES	YES
16	a	99.41	1.03121	YES	YES
17	a	106.93	0.10587	YES	YES
18	a	115.06	0.57837	YES	YES
19	a	124.51	0.24267	YES	YES
20	a	130.62	0.61017	YES	YES
21	a	142.11	0.86214	YES	YES
22	a	157.29	0.55479	YES	YES
23	a	184.92	0.19479	YES	YES
24	a	196.86	1.19340	YES	YES
25	a	215.56	1.28885	YES	YES
26	a	219.99	0.16940	YES	YES
27	a	237.14	1.67363	YES	YES
28	a	259.45	0.14314	YES	YES
29	a	289.72	0.20098	YES	YES
30	a	304.37	0.88957	YES	YES
31	a	332.98	0.97419	YES	YES
32	a	351.21	9.54902	YES	YES
33	a	385.30	2.59182	YES	YES
34	a	403.53	0.11870	YES	YES
35	a	406.94	0.93506	YES	YES
36	a	428.32	0.72794	YES	YES
37	a	447.17	1.62589	YES	YES
38	a	452.76	2.13332	YES	YES
39	a	465.85	10.10407	YES	YES
40	a	478.61	2.11244	YES	YES
41	a	492.64	1.85906	YES	YES
42	a	495.93	11.69786	YES	YES
43	a	510.21	5.47025	YES	YES
44	a	522.54	0.68273	YES	YES
45	a	538.58	11.69373	YES	YES
46	a	549.24	15.95716	YES	YES
47	a	551.00	3.74202	YES	YES
48	a	581.07	5.63391	YES	YES
49	a	584.20	14.70772	YES	YES
50	a	609.52	14.07838	YES	YES

TS_{23fa}′

```
SCF Energy (au) BP86/SV(P)                    -2391.943845351
SCF Energy (au) PBE0/def2-TZVPP               -2391.497946159
SCF Energy (au) PBE0/def2-TZVPP               -2391.5061895680 (Toluene Correction)
Zero Point Energy (au)                        0.3205041
Chemical Potential (kJ mol-1)                 691.29
Dispersion Correction (au) PBE0/def2-TZVPP -0.06768189

xyz coordinates
45

Mn    1.3110392    1.2919826    0.4372025
C     1.4509833    1.6980167    2.1987554
C     3.0228283    0.8213585    0.3786671
C     1.7953893    2.9380234   -0.1248740
O     2.2046709    3.9514035   -0.5338050
O     4.1482855    0.5024836    0.3461756
O     1.5310264    1.9758415    3.3279080
C    -1.5523798    0.5457394    1.0418146
C    -1.4986120    2.7521652    0.2485363
C    -2.9024106    2.8677863    0.3315387
C    -3.6342364    1.7570910    0.7744980
C    -2.9629776    0.5856278    1.1280883
C    -0.7457941   -0.6197127    1.4129148
C     0.6175561   -0.6239367    0.9817123
C     1.4303899   -1.7145122    1.3919974
C     0.9321047   -2.7317726    2.2105416
C    -0.4190106   -2.7209434    2.6216926
C    -1.2508336   -1.6730210    2.2171497
O    -0.7002393    3.7487217   -0.1755946
H    -3.4111534    3.8006983    0.0535798
H    -4.7332901    1.8131157    0.8378185
H    -3.5200531   -0.3020652    1.4585336
H     2.4804194   -1.7603028    1.0605214
H     1.6004942   -3.5474605    2.5338769
H    -0.8132982   -3.5238275    3.2654803
H    -2.2957236   -1.6488616    2.5674539
N    -0.8365671    1.6221262    0.5948643
H     0.7824923    1.3877611   -2.4572689
C     0.8270174    0.8827240   -1.4868665
C     0.7844700   -0.3219684   -1.0065775
C     0.9209701   -1.6842854   -1.5522209
C     1.1695360   -4.2139431   -2.8018412
C     2.1030016   -3.1991099   -3.0733444
C     1.9792576   -1.9436023   -2.4576369
C    -0.0051991   -2.7178798   -1.2778888
C     0.1145770   -3.9666638   -1.9047448
H     1.2648857   -5.1996778   -3.2869415
H     2.9370347   -3.3850051   -3.7704585
H     2.7109546   -1.1462934   -2.6638590
H    -0.8306913   -2.5304744   -0.5734895
H    -0.6238435   -4.7568831   -1.6894224
C    -1.2549939    4.9970395   -0.5660557
H    -1.9479386    4.8831263   -1.4315429
H    -0.3905350    5.6213350   -0.8662557
H    -1.7896031    5.4880350    0.2793667

$vibrational spectrum
#  mode     symmetry     wave number   IR intensity    selection rules
#                         cm**(-1)        km/mol          IR     RAMAN
     1        a            -281.82         0.00000        YES     YES
     2                        0.00         0.00000         -       -
     3                        0.00         0.00000         -       -
```

4		0.00	0.00000	-	-
5		0.00	0.00000	-	-
6		0.00	0.00000	-	-
7		0.00	0.00000	-	-
8	a	21.96	0.54731	YES	YES
9	a	27.17	0.31688	YES	YES
10	a	39.40	0.07178	YES	YES
11	a	45.13	0.01735	YES	YES
12	a	59.30	0.80359	YES	YES
13	a	73.87	1.42854	YES	YES
14	a	81.25	0.11047	YES	YES
15	a	89.84	0.12936	YES	YES
16	a	91.63	0.10392	YES	YES
17	a	109.10	0.05579	YES	YES
18	a	111.04	1.52700	YES	YES
19	a	129.12	0.58316	YES	YES
20	a	130.00	0.22055	YES	YES
21	a	142.74	0.77459	YES	YES
22	a	160.87	1.01154	YES	YES
23	a	184.29	0.59976	YES	YES
24	a	185.71	2.74327	YES	YES
25	a	209.39	0.21746	YES	YES
26	a	219.24	3.52486	YES	YES
27	a	226.92	2.15658	YES	YES
28	a	261.16	0.23288	YES	YES
29	a	284.51	0.53013	YES	YES
30	a	300.92	1.42087	YES	YES
31	a	315.13	2.22167	YES	YES
32	a	333.70	0.64837	YES	YES
33	a	402.41	0.38858	YES	YES
34	a	403.00	0.12920	YES	YES
35	a	410.69	4.78803	YES	YES
36	a	443.69	3.35847	YES	YES
37	a	457.29	0.26555	YES	YES
38	a	467.58	0.25933	YES	YES
39	a	475.36	3.06914	YES	YES
40	a	479.63	3.18434	YES	YES
41	a	502.35	7.87649	YES	YES
42	a	509.01	11.22374	YES	YES
43	a	510.08	10.04585	YES	YES
44	a	526.74	6.49210	YES	YES
45	a	541.34	9.55862	YES	YES
46	a	548.14	6.24154	YES	YES
47	a	558.45	15.13916	YES	YES
48	a	564.90	6.13221	YES	YES
49	a	581.38	3.49303	YES	YES
50	a	606.71	40.94505	YES	YES

TS$_{23ga}$

SCF Energy (au) BP86/SV(P)	-2508.390483009
SCF Energy (au) PBE0/def2-TZVPP	-2507.907823752
SCF Energy (au) PBE0/def2-TZVPP	-2507.9156889379 (Toluene Correction)
Zero Point Energy (au)	0.3677427
Chemical Potential (kJ mol^{-1})	805.67
Dispersion Correction (au) PBE0/def2-TZVPP	-0.08080308

xyz coordinates
51

Mn	1.5248441	-0.5845365	0.6397492
C	1.9479276	-0.1680022	2.3451840
C	3.1531787	-1.2378222	0.3611675
C	2.1434209	0.9801826	-0.0458451
O	2.6918521	1.9009823	-0.5073779
O	4.2221227	-1.6797369	0.1922384
O	2.2034064	0.0904312	3.4519353
C	-1.3262728	-1.0784282	1.4637251
C	-1.1652883	1.1729122	0.7906054
C	-2.5693532	1.2931026	0.7780946
C	-3.3670493	0.1875560	1.1054350
C	-2.7381636	-1.0002992	1.4764198
C	-0.5901441	-2.2833859	1.8490622
C	0.7694752	-2.3633513	1.4202424
C	1.5213038	-3.4987701	1.8290059
C	0.9525439	-4.5042281	2.6153884
C	-0.4005685	-4.4235477	3.0150732
C	-1.1636811	-3.3192696	2.6284128
C	-0.3684453	2.4183943	0.5898400
H	-3.0168067	2.2711242	0.5453896
H	-4.4666693	0.2630063	1.0922184
H	-3.3295190	-1.8843629	1.7555811
H	2.5741119	-3.5971104	1.5150369
H	1.5690844	-5.3632896	2.9303901
H	-0.8469301	-5.2157813	3.6375045
H	-2.2094528	-3.2406564	2.9683437
N	-0.5553920	-0.0151934	1.0669763
C	0.1552864	-0.4653066	-2.4658343
C	0.8115630	0.6114667	-3.1182067
C	0.3520392	1.0840650	-4.3563685
C	-0.7830842	0.5178294	-4.9641111
C	-1.4555771	-0.5377193	-4.3206713
C	-0.9942453	-1.0266136	-3.0914358
H	1.6965534	1.0694607	-2.6547579
H	0.8886911	1.9104129	-4.8516946
H	-1.1442849	0.8987999	-5.9335844
H	-2.3490344	-0.9888064	-4.7841610
H	-1.5217427	-1.8530576	-2.5889030
C	0.6129898	-1.0354979	-1.2186079
C	0.7942461	-2.1456615	-0.5795400
H	0.9486033	-3.2104797	-0.7768708
C	0.9675526	4.8921174	0.3197217
C	1.0864307	4.1530671	1.5096814
C	0.4248956	2.9235896	1.6455242
C	-0.4989074	3.1762558	-0.5944436
C	0.1746010	4.4006849	-0.7311774
H	1.4944440	5.8545831	0.2121793
H	1.7004501	4.5366872	2.3410568
H	0.5127752	2.3515817	2.5829314
H	-1.1148258	2.7888184	-1.4221905
H	0.0810448	4.9738029	-1.6683325

```
$vibrational spectrum
#  mode     symmetry     wave number   IR intensity    selection rules
#                         cm**(-1)        km/mol         IR     RAMAN
     1        a           -220.39         0.00000       YES     YES
     2                       0.00         0.00000        -       -
     3                       0.00         0.00000        -       -
     4                       0.00         0.00000        -       -
     5                       0.00         0.00000        -       -
     6                       0.00         0.00000        -       -
     7                       0.00         0.00000        -       -
     8        a              7.18         0.04481       YES     YES
     9        a             25.98         0.14963       YES     YES
    10        a             28.35         0.07413       YES     YES
    11        a             46.66         0.05710       YES     YES
    12        a             59.46         0.24527       YES     YES
    13        a             68.02         0.05833       YES     YES
    14        a             74.15         0.04441       YES     YES
    15        a             75.96         0.56208       YES     YES
    16        a             89.54         0.52008       YES     YES
    17        a             93.41         0.21133       YES     YES
    18        a            104.63         0.29995       YES     YES
    19        a            112.39         0.18400       YES     YES
    20        a            119.55         0.10028       YES     YES
    21        a            126.31         0.36676       YES     YES
    22        a            133.97         0.32997       YES     YES
    23        a            156.06         0.27131       YES     YES
    24        a            162.45         0.56834       YES     YES
    25        a            186.04         0.40033       YES     YES
    26        a            205.73         0.28924       YES     YES
    27        a            217.74         0.64486       YES     YES
    28        a            233.91         0.23953       YES     YES
    29        a            243.74         1.42955       YES     YES
    30        a            292.38         0.46312       YES     YES
    31        a            311.96         0.57499       YES     YES
    32        a            334.06         0.79096       YES     YES
    33        a            352.03         9.28848       YES     YES
    34        a            368.04         0.39060       YES     YES
    35        a            390.04         4.82528       YES     YES
    36        a            401.29         0.38297       YES     YES
    37        a            402.85         0.11789       YES     YES
    38        a            423.04         1.79015       YES     YES
    39        a            442.50         0.64033       YES     YES
    40        a            449.25         2.87375       YES     YES
    41        a            464.05         9.91506       YES     YES
    42        a            470.92         3.87025       YES     YES
    43        a            485.37         1.17087       YES     YES
    44        a            496.16         4.18584       YES     YES
    45        a            496.70         7.06281       YES     YES
    46        a            509.12         5.45970       YES     YES
    47        a            522.80         0.52314       YES     YES
    48        a            544.46        10.99192       YES     YES
    49        a            548.38        10.35088       YES     YES
    50        a            553.61         7.49606       YES     YES
```

TS_{23ga}'

SCF Energy (au) BP86/SV(P) -2508.381424342
SCF Energy (au) PBE0/def2-TZVPP -2507.899292682
SCF Energy (au) PBE0/def2-TZVPP -2507.9076218786 (Toluene Correction)
Zero Point Energy (au) 0.3672553
Chemical Potential (kJ mol^{-1}) 806.64
Dispersion Correction (au) PBE0/def2-TZVPP -0.08140660

xyz coordinates
51

Mn	1.2410302	0.5333857	0.2961025
C	1.5263352	1.0929493	1.9927023
C	2.9071938	-0.0668038	0.1951247
C	1.8221134	2.0394922	-0.5207088
O	2.3096189	2.9125814	-1.1210368
O	4.0048967	-0.4683008	0.1367038
O	1.6945615	1.4668466	3.0848500
C	-1.6597261	-0.0149824	0.9417047
C	-1.5364748	2.1990646	0.1567478
C	-2.9411974	2.2625908	0.0498340
C	-3.7179655	1.1449970	0.3851796
C	-3.0711123	0.0031825	0.8574349
C	-0.9090923	-1.1703598	1.4404657
C	0.4594018	-1.2690511	1.0439718
C	1.2150706	-2.3528122	1.5680757
C	0.6562456	-3.2665642	2.4657516
C	-0.7007262	-3.1607172	2.8435114
C	-1.4788625	-2.1225976	2.3227578
C	-0.7715030	3.4558025	-0.0874276
H	-3.4081654	3.2081733	-0.2644633
H	-4.8162981	1.1754567	0.2970408
H	-3.6447395	-0.8918064	1.1399669
H	2.2676031	-2.4767398	1.2651584
H	1.2811689	-4.0761009	2.8790807
H	-1.1414055	-3.8821409	3.5506334
H	-2.5291042	-2.0190188	2.6420475
N	-0.9031486	1.0552181	0.5435537
H	0.4499847	0.4216045	-2.5439982
C	0.5607354	-0.0039046	-1.5418071
C	0.5742943	-1.1626540	-0.9586258
C	0.7256616	-2.5658496	-1.3849620
C	1.0037271	-5.1939512	-2.3999772
C	1.9155938	-4.1931897	-2.7769453
C	1.7767054	-2.8884252	-2.2778509
C	-0.1787191	-3.5841268	-1.0032094
C	-0.0447128	-4.8831180	-1.5151326
H	1.1107196	-6.2183806	-2.7938696
H	2.7439438	-4.4283081	-3.4657565
H	2.4910807	-2.1013343	-2.5668104
H	-0.9984457	-3.3465788	-0.3070807
H	-0.7656718	-5.6628181	-1.2180546
C	0.5151894	5.9401442	-0.4778100
C	0.6249282	5.2747137	0.7554304
C	-0.0111497	4.0399951	0.9510863
C	-0.8937585	4.1414036	-1.3158616
C	-0.2447296	5.3703487	-1.5131783
H	1.0235453	6.9062034	-0.6319847
H	1.2129245	5.7201377	1.5746824
H	0.0709041	3.5264374	1.9220227
H	-1.4872165	3.6944123	-2.1308931
H	-0.3312525	5.8854934	-2.4841767

```
$vibrational spectrum
#  mode     symmetry     wave number   IR intensity    selection rules
#                         cm**(-1)        km/mol          IR     RAMAN
     1        a            -284.04         0.00000        YES     YES
     2                        0.00         0.00000         -       -
     3                        0.00         0.00000         -       -
     4                        0.00         0.00000         -       -
     5                        0.00         0.00000         -       -
     6                        0.00         0.00000         -       -
     7                        0.00         0.00000         -       -
     8        a              21.83         0.24742        YES     YES
     9        a              26.53         0.04054        YES     YES
    10        a              27.55         0.22997        YES     YES
    11        a              55.52         0.15441        YES     YES
    12        a              58.54         0.19895        YES     YES
    13        a              61.78         0.23479        YES     YES
    14        a              71.79         0.06660        YES     YES
    15        a              76.28         0.41529        YES     YES
    16        a              83.91         0.31739        YES     YES
    17        a              95.67         0.03798        YES     YES
    18        a              99.00         0.00329        YES     YES
    19        a             107.45         0.22463        YES     YES
    20        a             121.83         0.36120        YES     YES
    21        a             126.36         0.65725        YES     YES
    22        a             137.69         0.31483        YES     YES
    23        a             150.00         0.32541        YES     YES
    24        a             162.20         1.30034        YES     YES
    25        a             188.41         2.47324        YES     YES
    26        a             206.75         0.28675        YES     YES
    27        a             213.47         0.30054        YES     YES
    28        a             219.86         2.90087        YES     YES
    29        a             243.29         2.20485        YES     YES
    30        a             286.50         0.19870        YES     YES
    31        a             310.92         0.22004        YES     YES
    32        a             315.82         3.21257        YES     YES
    33        a             336.36         0.22768        YES     YES
    34        a             363.76         0.87637        YES     YES
    35        a             400.50         0.19138        YES     YES
    36        a             403.33         0.02312        YES     YES
    37        a             410.28         4.24302        YES     YES
    38        a             428.24         3.94233        YES     YES
    39        a             452.97         0.04614        YES     YES
    40        a             463.34         1.26011        YES     YES
    41        a             471.28         2.93625        YES     YES
    42        a             476.39         2.12371        YES     YES
    43        a             491.14         1.06595        YES     YES
    44        a             499.32         3.23268        YES     YES
    45        a             509.11        20.73100        YES     YES
    46        a             511.47         2.04412        YES     YES
    47        a             527.22         1.86051        YES     YES
    48        a             542.20        12.79466        YES     YES
    49        a             553.78         5.71313        YES     YES
    50        a             559.95        14.88453        YES     YES
```

TS_{23ha}

SCF Energy (au) BP86/SV(P)	-2354.862307833	
SCF Energy (au) PBE0/def2-TZVPP	-2354.406354751	
SCF Energy (au) PBE0/def2-TZVPP	-2354.4142715193	(Toluene Correction)
Zero Point Energy (au)	0.3226651	
Chemical Potential (kJ mol^{-1})	696.08	
Dispersion Correction (au) PBE0/def2-TZVPP	-0.06451629	

xyz coordinates
45

Mn	2.2084975	0.5250623	0.5478324
C	2.6176997	0.7441853	2.2909833
C	3.9129016	0.0878398	0.1836519
C	2.5072647	2.2676112	0.1533830
O	2.7042883	3.3973215	-0.0618477
O	5.0195021	-0.2048262	-0.0461420
O	2.8692686	0.8708948	3.4207629
C	-0.5551355	-0.0946769	1.2974896
C	-2.0218377	0.1023037	1.4525877
C	0.1473763	-1.3403631	1.5748185
C	1.5219795	-1.3471266	1.1580807
C	2.2933222	-2.4938345	1.4860373
C	1.7320956	-3.5737157	2.1751747
C	0.3709319	-3.5602620	2.5605212
C	-0.4165103	-2.4465547	2.2606540
H	3.3550933	-2.5382253	1.1887155
H	2.3632348	-4.4427922	2.4280768
H	-0.0603429	-4.4147024	3.1066682
H	-1.4689279	-2.4092071	2.5857970
N	0.2270115	0.8730877	0.8742984
C	0.9202487	0.8497547	-2.5944228
C	1.4599919	2.0800371	-3.0524654
C	0.9950442	2.6676223	-4.2376942
C	-0.0284697	2.0583820	-4.9852224
C	-0.5822600	0.8444312	-4.5379431
C	-0.1163384	0.2449687	-3.3610110
H	2.2572838	2.5714496	-2.4764361
H	1.4363998	3.6186356	-4.5793264
H	-0.3965910	2.5289268	-5.9117454
H	-1.3869890	0.3575539	-5.1140262
H	-0.5514575	-0.7048071	-3.0108143
C	1.3904782	0.1728235	-1.4093728
C	1.6563040	-0.9615126	-0.8514462
H	1.9301743	-1.9875303	-1.1118527
C	-4.8044947	0.5537602	1.6782051
C	-4.3265595	-0.6013673	1.0345916
C	-2.9461264	-0.8304895	0.9262954
C	-2.5138801	1.2629604	2.0949284
C	-3.8947102	1.4844429	2.2095130
H	-5.8894386	0.7282725	1.7672252
H	-5.0359508	-1.3295173	0.6079751
H	-2.5756396	-1.7276806	0.4049310
H	-1.8032385	1.9844021	2.5317119
H	-4.2629510	2.3874861	2.7235363
H	-0.2585430	1.7449751	0.6173225

$vibrational spectrum

# mode	symmetry	wave number	IR intensity	selection rules	
#		cm**(-1)	km/mol	IR	RAMAN
1	a	-211.06	0.00000	YES	YES
2		0.00	0.00000	-	-
3		0.00	0.00000	-	-

4		0.00	0.00000	-	-
5		0.00	0.00000	-	-
6		0.00	0.00000	-	-
7		0.00	0.00000	-	-
8	a	18.67	0.09950	YES	YES
9	a	21.60	0.02891	YES	YES
10	a	38.38	0.00779	YES	YES
11	a	48.05	0.09372	YES	YES
12	a	51.86	0.11616	YES	YES
13	a	69.55	0.22643	YES	YES
14	a	73.33	0.08989	YES	YES
15	a	80.49	0.12999	YES	YES
16	a	88.89	0.07702	YES	YES
17	a	94.68	0.23259	YES	YES
18	a	103.22	0.02377	YES	YES
19	a	110.35	0.25271	YES	YES
20	a	134.35	0.18439	YES	YES
21	a	143.04	0.44549	YES	YES
22	a	176.35	0.76100	YES	YES
23	a	195.95	0.47683	YES	YES
24	a	204.67	0.63859	YES	YES
25	a	212.39	0.45590	YES	YES
26	a	238.72	0.78590	YES	YES
27	a	269.37	1.77691	YES	YES
28	a	304.64	0.65671	YES	YES
29	a	340.16	8.47923	YES	YES
30	a	358.93	0.43972	YES	YES
31	a	395.36	1.89545	YES	YES
32	a	402.38	0.02315	YES	YES
33	a	404.64	0.38999	YES	YES
34	a	420.53	1.91042	YES	YES
35	a	436.65	0.58360	YES	YES
36	a	447.96	4.00709	YES	YES
37	a	461.40	20.24491	YES	YES
38	a	465.05	2.25747	YES	YES
39	a	485.47	0.85113	YES	YES
40	a	492.96	2.86485	YES	YES
41	a	497.08	3.33844	YES	YES
42	a	504.77	8.24943	YES	YES
43	a	521.80	0.45494	YES	YES
44	a	540.22	19.26739	YES	YES
45	a	544.86	15.56428	YES	YES
46	a	564.68	28.71926	YES	YES
47	a	590.89	1.48234	YES	YES
48	a	609.57	1.75365	YES	YES
49	a	611.76	5.86176	YES	YES
50	a	618.68	13.41133	YES	YES

TS_{23ia}

SCF Energy (au) BP86/SV(P)	-2183.113967934	
SCF Energy (au) PBE0/def2-TZVPP	-2182.694332982	
SCF Energy (au) PBE0/def2-TZVPP	-2182.7012637601	(Toluene Correction)
Zero Point Energy (au)	0.2578498	
Chemical Potential (kJ mol^{-1})	537.65	
Dispersion Correction (au) PBE0/def2-TZVPP	-0.05000930	

xyz coordinates
37

Mn	1.3012750	0.6735212	0.8636467
C	1.6923990	0.9350102	2.6106761
C	3.0056460	0.2982574	0.5195438
C	1.5195873	2.4381721	0.4701240
O	1.6680679	3.5695831	0.2467560
O	4.1188443	0.0253376	0.2925176
O	1.9402074	1.0881191	3.7374012
C	-1.4133247	0.0725285	1.6759428
C	-2.8893810	0.2622864	1.9251222
C	-0.7120733	-1.1720529	1.9402837
C	0.6484269	-1.1933217	1.4760537
C	1.4170669	-2.3495710	1.7900079
C	0.8606475	-3.4172984	2.4976908
C	-0.4970965	-3.3998829	2.9059424
C	-1.2792418	-2.2813597	2.6211696
H	2.4683728	-2.4084477	1.4609439
H	1.4898509	-4.2905435	2.7409042
H	-0.9242473	-4.2576205	3.4497033
H	-2.3326827	-2.2471590	2.9453367
O	-0.7429383	1.0326985	1.2089872
C	0.1325468	0.9718078	-2.2911245
C	0.7714301	2.1345748	-2.7930459
C	0.3824316	2.6896191	-4.0202216
C	-0.6628773	2.1152407	-4.7650935
C	-1.3143103	0.9685711	-4.2745778
C	-0.9219310	0.3997316	-3.0562753
H	1.5860925	2.5985052	-2.2181170
H	0.8988533	3.5882855	-4.3963692
H	-0.9726876	2.5619450	-5.7242020
H	-2.1370795	0.5107462	-4.8488892
H	-1.4327901	-0.4979350	-2.6718159
C	0.5245875	0.3310298	-1.0565555
C	0.7064571	-0.8183133	-0.4815302
H	0.9010254	-1.8573590	-0.7651214
H	-3.1924575	1.2771447	1.5999085
H	-3.1200868	0.1456830	3.0077219
H	-3.4886102	-0.4975339	1.3765547

# mode	symmetry	wave number	IR intensity	selection rules IR	selection rules RAMAN
#		cm**(-1)	km/mol	IR	RAMAN
1	a	-228.38	0.00000	YES	YES
2		0.00	0.00000	-	-
3		0.00	0.00000	-	-
4		0.00	0.00000	-	-
5		0.00	0.00000	-	-
6		0.00	0.00000	-	-
7		0.00	0.00000	-	-
8	a	18.98	0.12008	YES	YES
9	a	28.24	0.16870	YES	YES
10	a	56.30	0.07960	YES	YES

11	a	57.56	0.36639	YES	YES
12	a	73.76	0.43554	YES	YES
13	a	77.76	0.29842	YES	YES
14	a	90.84	0.23664	YES	YES
15	a	98.00	0.44156	YES	YES
16	a	103.22	0.33827	YES	YES
17	a	109.70	0.29581	YES	YES
18	a	123.22	0.04442	YES	YES
19	a	139.00	0.13164	YES	YES
20	a	160.46	0.60555	YES	YES
21	a	183.73	0.72674	YES	YES
22	a	202.28	0.19418	YES	YES
23	a	217.24	1.59049	YES	YES
24	a	246.57	1.24502	YES	YES
25	a	268.54	0.12494	YES	YES
26	a	284.53	0.22753	YES	YES
27	a	344.37	6.19449	YES	YES
28	a	378.18	1.40902	YES	YES
29	a	401.89	0.07019	YES	YES
30	a	410.53	2.74233	YES	YES
31	a	412.51	0.38710	YES	YES
32	a	437.47	1.28361	YES	YES
33	a	451.81	0.33644	YES	YES
34	a	461.91	10.89574	YES	YES
35	a	474.64	1.21843	YES	YES
36	a	489.37	4.15812	YES	YES
37	a	499.02	12.59916	YES	YES
38	a	507.71	2.91113	YES	YES
39	a	523.72	0.68396	YES	YES
40	a	536.45	21.31995	YES	YES
41	a	544.19	10.51342	YES	YES
42	a	587.29	45.28376	YES	YES
43	a	601.55	8.05846	YES	YES
44	a	605.21	3.61664	YES	YES
45	a	610.37	16.40323	YES	YES
46	a	618.62	32.05822	YES	YES
47	a	629.66	5.92647	YES	YES
48	a	650.99	6.48357	YES	YES
49	a	662.64	77.38872	YES	YES
50	a	683.14	75.60527	YES	YES

TS_{23ia}′

SCF Energy (au) BP86/SV(P)	-2183.103988116	
SCF Energy (au) PBE0/def2-TZVPP	-2182.684894240	
SCF Energy (au) PBE0/def2-TZVPP	-2182.6918100274	(Toluene Correction)
Zero Point Energy (au)	0.2574986	
Chemical Potential (kJ mol^{-1})	536.55	
Dispersion Correction (au) PBE0/def2-TZVPP	-0.05277314	

xyz coordinates
37

Mn	0.8656508	2.1918527	0.3154680
C	1.1790350	2.5549686	2.0654266
C	2.5879855	1.8447707	0.0639702
C	1.0592617	3.9108963	-0.2271348
O	1.1764375	5.0000906	-0.6170753
O	3.7169389	1.5924075	-0.1107837
O	1.3780395	2.7885345	3.1888876
C	-1.8851770	1.5606129	1.0314934
C	-3.3749241	1.7211766	1.2143033
C	-1.1676374	0.3385116	1.3637880
C	0.1928795	0.2976471	0.8966920
C	0.9769784	-0.8171162	1.3068905
C	0.4509880	-1.8082698	2.1384112
C	-0.9031338	-1.7728671	2.5530957
C	-1.7117010	-0.7081467	2.1532768
H	2.0212614	-0.8984200	0.9639711
H	1.0995558	-2.6375996	2.4677812
H	-1.3097820	-2.5737732	3.1913226
H	-2.7632831	-0.6596729	2.4818752
O	-1.2156307	2.5215978	0.5717577
H	0.0508076	2.2612256	-2.5157530
C	0.2007315	1.7823629	-1.5430835
C	0.2860068	0.5846236	-1.0359600
C	0.5162019	-0.7600306	-1.6056033
H	-3.6912993	2.7092654	0.8251728
H	-3.6424525	1.6592183	2.2930281
H	-3.9340442	0.9161822	0.6885007
C	0.9322564	-3.2571365	-2.8686490
C	1.8416603	-2.2050001	-3.0704459
C	1.6339749	-0.9629319	-2.4495997
C	-0.3846345	-1.8296872	-1.3973801
C	-0.1825077	-3.0636265	-2.0332442
H	1.0923530	-4.2302003	-3.3619071
H	2.7212031	-2.3489816	-3.7198076
H	2.3454830	-0.1362331	-2.6045609
H	-1.2567891	-1.6828597	-0.7402380
H	-0.9026940	-3.8833917	-1.8738868

$vibrational spectrum

# mode	symmetry	wave number	IR intensity	selection rules	
#		cm**(-1)	km/mol	IR	RAMAN
1	a	-289.58	0.00000	YES	YES
2		0.00	0.00000	-	-
3		0.00	0.00000	-	-
4		0.00	0.00000	-	-
5		0.00	0.00000	-	-
6		0.00	0.00000	-	-
7		0.00	0.00000	-	-
8	a	19.52	0.07940	YES	YES
9	a	29.50	0.44592	YES	YES
10	a	47.00	0.00881	YES	YES

11	a	66.99	0.70250	YES	YES
12	a	69.94	0.36195	YES	YES
13	a	75.16	0.35748	YES	YES
14	a	87.37	0.16474	YES	YES
15	a	92.36	0.09761	YES	YES
16	a	100.95	0.18832	YES	YES
17	a	105.08	0.60387	YES	YES
18	a	125.50	0.05780	YES	YES
19	a	145.83	0.68043	YES	YES
20	a	161.43	0.37167	YES	YES
21	a	189.20	2.75605	YES	YES
22	a	201.91	1.88209	YES	YES
23	a	214.20	1.99503	YES	YES
24	a	233.76	2.14063	YES	YES
25	a	247.68	0.55556	YES	YES
26	a	280.54	0.32179	YES	YES
27	a	305.53	1.16879	YES	YES
28	a	391.57	2.59632	YES	YES
29	a	400.96	0.00811	YES	YES
30	a	409.44	2.07552	YES	YES
31	a	418.83	5.94734	YES	YES
32	a	445.08	0.94194	YES	YES
33	a	466.52	0.52551	YES	YES
34	a	474.78	0.62650	YES	YES
35	a	486.70	3.71712	YES	YES
36	a	497.96	17.15639	YES	YES
37	a	510.90	0.65377	YES	YES
38	a	514.70	9.50928	YES	YES
39	a	523.80	7.85160	YES	YES
40	a	535.03	12.63137	YES	YES
41	a	562.79	13.26407	YES	YES
42	a	569.97	6.56249	YES	YES
43	a	599.46	22.92425	YES	YES
44	a	604.08	9.15845	YES	YES
45	a	610.41	63.96900	YES	YES
46	a	613.73	5.51951	YES	YES
47	a	628.85	10.60410	YES	YES
48	a	656.49	38.14813	YES	YES
49	a	681.82	76.06539	YES	YES
50	a	689.44	19.04587	YES	YES

TS_{23ja}

SCF Energy (au) BP86/SV(P) -2452.074142234
SCF Energy (au) PBE0/def2-TZVPP -2451.611763009
SCF Energy (au) PBE0/def2-TZVPP -2451.6198030278 (Toluene Correction)
Zero Point Energy (au) 0.3426121
Chemical Potential (kJ mol^{-1}) 738.90
Dispersion Correction (au) PBE0/def2-TZVPP -0.06697983

xyz coordinates
48

Mn	1.5654786	0.9681938	0.4627900
C	1.9182416	1.2711050	2.2078398
C	3.2854914	0.6398468	0.1205255
C	1.7410987	2.7390327	0.0539965
O	1.8481311	3.8736680	-0.1726503
O	4.4094621	0.4308376	-0.1165767
O	2.1443005	1.4632721	3.3334955
C	-1.1354830	0.3230288	1.1767272
C	-2.5924500	0.4860401	1.4246771
C	-0.4263610	-0.8978221	1.4068286
C	0.9262225	-0.8932344	1.0551879
C	1.7359861	-2.1102219	1.3285877
O	-0.4687028	1.3262799	0.7545599
C	0.4294030	1.3443794	-2.7203335
C	1.0947815	2.4917352	-3.2222126
C	0.6939823	3.0742199	-4.4328112
C	-0.3837235	2.5396352	-5.1609371
C	-1.0597128	1.4079526	-4.6693880
C	-0.6607253	0.8148338	-3.4650582
H	1.9398299	2.9201504	-2.6636380
H	1.2293198	3.9607478	-4.8111972
H	-0.6992564	3.0056133	-6.1089827
H	-1.9080140	0.9816840	-5.2305998
H	-1.1914557	-0.0692529	-3.0769267
C	0.8343937	0.6607428	-1.5140208
C	1.0386082	-0.4801990	-0.9434658
H	1.2698459	-1.5240326	-1.1830719
C	3.2211616	-4.4623286	1.9036248
C	1.9746975	-4.5548126	1.2605153
C	1.2370539	-3.3932483	0.9855944
C	3.0006889	-2.0360127	1.9592241
C	3.7266886	-3.1994438	2.2567531
H	3.8001881	-5.3739688	2.1256541
H	1.5719437	-5.5398198	0.9715006
H	0.2581377	-3.4736495	0.4834480
H	3.4100301	-1.0544150	2.2403214
H	4.7022422	-3.1159529	2.7634979
C	-5.3471196	0.8537170	1.9156071
C	-4.8631252	-0.4103499	1.5360898
C	-3.4918806	-0.5979408	1.3053365
C	-3.0919689	1.7596975	1.7845470
C	-4.4577647	1.9373832	2.0431648
H	-6.4229519	0.9971777	2.1104698
H	-5.5601838	-1.2565457	1.4170325
H	-3.1189453	-1.5882096	0.9971250
H	-2.3856525	2.5997060	1.8729524
H	-4.8346486	2.9278332	2.3479318
H	-0.9072833	-1.7670527	1.8862643

$vibrational spectrum

# mode	symmetry	wave number cm**(-1)	IR intensity km/mol	selection rules IR	selection rules RAMAN
1	a	-180.13	0.00000	YES	YES
2		0.00	0.00000	-	-
3		0.00	0.00000	-	-
4		0.00	0.00000	-	-
5		0.00	0.00000	-	-
6		0.00	0.00000	-	-
7		0.00	0.00000	-	-
8	a	16.16	0.08999	YES	YES
9	a	20.23	0.17454	YES	YES
10	a	34.71	0.06401	YES	YES
11	a	37.61	0.19479	YES	YES
12	a	43.85	0.24760	YES	YES
13	a	51.29	0.13596	YES	YES
14	a	54.50	0.27276	YES	YES
15	a	63.64	0.23335	YES	YES
16	a	74.41	0.09767	YES	YES
17	a	93.89	0.26720	YES	YES
18	a	99.92	0.26287	YES	YES
19	a	102.96	0.22256	YES	YES
20	a	105.64	0.21123	YES	YES
21	a	116.39	0.20491	YES	YES
22	a	143.20	0.60293	YES	YES
23	a	149.93	0.16503	YES	YES
24	a	190.48	1.23144	YES	YES
25	a	205.11	1.14525	YES	YES
26	a	214.53	0.46048	YES	YES
27	a	220.33	0.23064	YES	YES
28	a	237.52	0.32706	YES	YES
29	a	247.55	0.52677	YES	YES
30	a	282.57	2.82391	YES	YES
31	a	341.14	1.83436	YES	YES
32	a	353.75	3.35426	YES	YES
33	a	366.36	8.42762	YES	YES
34	a	401.05	0.75597	YES	YES
35	a	401.37	0.17593	YES	YES
36	a	404.09	1.14187	YES	YES
37	a	421.75	0.55163	YES	YES
38	a	438.05	4.88263	YES	YES
39	a	445.64	5.18937	YES	YES
40	a	467.10	3.82912	YES	YES
41	a	469.55	9.05303	YES	YES
42	a	492.57	4.87405	YES	YES
43	a	495.94	4.92155	YES	YES
44	a	498.59	9.00082	YES	YES
45	a	516.90	5.21897	YES	YES
46	a	522.34	2.90990	YES	YES
47	a	539.29	7.89970	YES	YES
48	a	550.81	20.25726	YES	YES
49	a	556.18	47.74073	YES	YES
50	a	584.71	36.46291	YES	YES

TS_{38ia}

```
SCF Energy (au) BP86/SV(P)                     -2183.131539912
SCF Energy (au) PBE0/def2-TZVPP                -2182.721878063
SCF Energy (au) PBE0/def2-TZVPP                -2182.7291178777 (Toluene Correction)
Zero Point Energy (au)                         0.2598847
Chemical Potential (kJ mol-1)                  548.92
Dispersion Correction (au) PBE0/def2-TZVPP -0.05353197

xyz coordinates
37

C     1.3367351   -0.3768756    2.2988397
C    -0.8696697    0.8718326    2.5792574
C     0.2856535    0.5555105   -0.1853000
C    -0.8282425   -1.6478829    2.9806592
C     1.2519233   -1.5778408   -0.5630382
C     2.2661993   -2.5523916   -0.6927707
C     0.7030816   -4.2683414    0.1152815
C    -0.3271217   -3.3227472    0.2125609
C    -0.0654066   -1.9849484   -0.1596621
C    -1.1360361   -0.9068470   -0.3388982
C    -1.5758540   -0.6336499   -1.7822888
Mn   -0.3279993   -0.5396906    1.6508554
O    -2.0534784   -0.8175555    0.5732889
O     2.4163054   -0.2735892    2.7286415
O    -1.2253325    1.7938260    3.1976814
C     1.3383776   -0.1411305   -0.7477564
O    -1.1596605   -2.3668982    3.8350984
C     1.9817374   -3.8823686   -0.3520192
H     2.1689385    0.3057951   -1.3249510
C    -0.0172476    1.9784906   -0.3980815
C    -0.6588862    4.7126596   -0.8344551
C    -1.6449917    3.8175246   -0.3849215
C    -1.3271176    2.4705144   -0.1582523
C     0.9650262    2.8969337   -0.8540831
C     0.6494950    4.2465958   -1.0614716
H    -0.9056341    5.7744510   -0.9996448
H    -2.6712222    4.1733747   -0.1962435
H    -2.1011877    1.7750955    0.2048468
H     1.9976462    2.5529194   -1.0266605
H     1.4329602    4.9443609   -1.4014742
H    -1.3413772   -3.6078647    0.5360664
H     0.5161972   -5.3184823    0.3920617
H     2.7777451   -4.6417863   -0.4299211
H     3.2777694   -2.2619577   -1.0205504
H    -0.7404145   -0.7332922   -2.5036776
H    -2.0291270    0.3746046   -1.8699356
H    -2.3597839   -1.3883484   -2.0190815

$vibrational spectrum
#  mode     symmetry     wave number   IR intensity    selection rules
#                         cm**(-1)        km/mol          IR     RAMAN
     1        a           -178.15         0.00000        YES     YES
     2                       0.00         0.00000         -       -
     3                       0.00         0.00000         -       -
     4                       0.00         0.00000         -       -
     5                       0.00         0.00000         -       -
     6                       0.00         0.00000         -       -
     7                       0.00         0.00000         -       -
     8        a              38.07        0.17468        YES     YES
     9        a              42.16        0.34856        YES     YES
    10        a              57.65        0.64808        YES     YES
    11        a              69.07        0.05984        YES     YES
```

12	a	79.69	1.20659	YES	YES
13	a	85.09	0.07459	YES	YES
14	a	91.20	0.11329	YES	YES
15	a	101.05	0.11238	YES	YES
16	a	108.83	0.07859	YES	YES
17	a	109.28	0.33871	YES	YES
18	a	129.11	1.29873	YES	YES
19	a	172.76	1.55207	YES	YES
20	a	197.82	1.60561	YES	YES
21	a	218.20	1.84289	YES	YES
22	a	229.25	1.86660	YES	YES
23	a	250.83	0.90614	YES	YES
24	a	261.45	5.14381	YES	YES
25	a	277.68	0.84444	YES	YES
26	a	299.35	0.12607	YES	YES
27	a	346.64	4.71924	YES	YES
28	a	365.64	21.12433	YES	YES
29	a	405.32	0.81981	YES	YES
30	a	442.72	2.88639	YES	YES
31	a	448.97	1.12164	YES	YES
32	a	463.59	3.13786	YES	YES
33	a	472.79	2.09847	YES	YES
34	a	486.71	13.64170	YES	YES
35	a	489.97	4.34087	YES	YES
36	a	498.19	5.76372	YES	YES
37	a	518.61	14.10605	YES	YES
38	a	526.22	1.99394	YES	YES
39	a	532.54	1.56273	YES	YES
40	a	538.83	2.68195	YES	YES
41	a	545.65	5.39873	YES	YES
42	a	578.06	50.23447	YES	YES
43	a	605.11	1.59629	YES	YES
44	a	612.32	2.01782	YES	YES
45	a	625.64	19.88850	YES	YES
46	a	631.86	24.17884	YES	YES
47	a	640.70	6.88353	YES	YES
48	a	687.72	43.96053	YES	YES
49	a	697.76	24.66806	YES	YES
50	a	707.48	1.42323	YES	YES

TS_{38ja}
SCF Energy (au) BP86/SV(P) -2452.085970536
SCF Energy (au) PBE0/def2-TZVPP -2451.630064256
SCF Energy (au) PBE0/def2-TZVPP -2451.6393433949 (Toluene Correction)
Zero Point Energy (au) 0.3438944
Chemical Potential (kJ mol^{-1}) 742.05
Dispersion Correction (au) PBE0/def2-TZVPP -0.07029183

xyz coordinates
48

C	1.4459651	-0.1758419	2.8322908
C	-0.8719835	0.8033845	3.3521899
C	0.0125768	0.8884238	0.4443620
C	-0.5083544	-1.7779318	3.3370157
C	1.0132788	-1.2451803	0.1905398
C	2.1795258	-2.1580859	0.1205537
C	-0.3067939	-1.6681079	0.4295187
C	-1.4034456	-0.6318798	0.2980345
C	-2.0187606	-0.3064184	-1.0455662
Mn	-0.2219443	-0.4141605	2.2012076
O	-2.1573556	-0.5843903	1.3513091
O	2.5026577	-0.0181497	3.3015286
O	-1.3089156	1.5830339	4.0958950
C	1.0950577	0.1951911	-0.0519171
O	-0.7006179	-2.6852305	4.0434619
H	1.9811696	0.6399284	-0.5426852
C	-0.2991287	2.3090379	0.2745308
C	-0.9605832	5.0546709	-0.0279566
C	-1.8643864	4.1740186	0.5920341
C	-1.5362569	2.8195546	0.7454660
C	0.5953798	3.2118961	-0.3598140
C	0.2720649	4.5675809	-0.5018172
H	-1.2138114	6.1218979	-0.1407718
H	-2.8323003	4.5476008	0.9648278
H	-2.2417561	2.1306412	1.2353915
H	1.5655869	2.8492619	-0.7365969
H	0.9880235	5.2532203	-0.9851804
C	4.3876931	-3.9295352	-0.0631820
C	4.4216323	-2.6878733	-0.7187161
C	3.3334881	-1.8068533	-0.6202468
C	2.1615645	-3.4136922	0.7770749
C	3.2539196	-4.2864338	0.6895042
H	5.2462816	-4.6173137	-0.1331958
H	5.3049295	-2.3975818	-1.3107729
H	3.3789063	-0.8420686	-1.1485920
H	1.2920379	-3.6977317	1.3904486
H	3.2228163	-5.2524159	1.2194816
C	-3.3354897	0.2099527	-3.4882376
C	-2.0171691	-0.2836324	-3.4855670
C	-1.3643536	-0.5440095	-2.2721703
C	-3.3403718	0.1856946	-1.0537263
C	-3.9954496	0.4391063	-2.2694879
H	-3.8483526	0.4108975	-4.4436166
H	-1.4963679	-0.4734897	-4.4387979
H	-0.3342230	-0.9371152	-2.2802273
H	-3.8489404	0.3411595	-0.0895448
H	-5.0318163	0.8173070	-2.2638959
H	-0.5956271	-2.7183371	0.5856160

$vibrational spectrum

# mode	symmetry	wave number	IR intensity	selection rules	
#		cm**(-1)	km/mol	IR	RAMAN
1	a	-257.50	0.00000	YES	YES
2		0.00	0.00000	-	-
3		0.00	0.00000	-	-
4		0.00	0.00000	-	-
5		0.00	0.00000	-	-
6		0.00	0.00000	-	-
7		0.00	0.00000	-	-
8	a	16.16	0.35618	YES	YES
9	a	19.51	0.10998	YES	YES
10	a	30.74	0.85372	YES	YES
11	a	37.52	0.01350	YES	YES
12	a	40.58	0.13918	YES	YES
13	a	47.33	0.01430	YES	YES
14	a	51.11	0.28303	YES	YES
15	a	59.59	0.80674	YES	YES
16	a	74.06	0.79660	YES	YES
17	a	84.46	0.71821	YES	YES
18	a	89.28	0.09853	YES	YES
19	a	100.80	0.74385	YES	YES
20	a	110.29	1.42942	YES	YES
21	a	124.54	0.19616	YES	YES
22	a	133.27	0.44369	YES	YES
23	a	157.79	2.33843	YES	YES
24	a	187.55	0.58258	YES	YES
25	a	203.50	1.02218	YES	YES
26	a	218.94	3.89951	YES	YES
27	a	235.58	1.10036	YES	YES
28	a	242.37	0.84239	YES	YES
29	a	249.53	0.58950	YES	YES
30	a	290.62	8.45380	YES	YES
31	a	355.98	18.12758	YES	YES
32	a	373.78	1.88651	YES	YES
33	a	396.89	1.42140	YES	YES
34	a	400.74	0.64740	YES	YES
35	a	402.24	0.55560	YES	YES
36	a	416.32	11.16342	YES	YES
37	a	438.96	1.67739	YES	YES
38	a	456.34	7.67132	YES	YES
39	a	462.49	3.16918	YES	YES
40	a	473.97	8.00078	YES	YES
41	a	478.28	0.19856	YES	YES
42	a	480.96	1.70836	YES	YES
43	a	494.62	8.40481	YES	YES
44	a	507.88	11.33733	YES	YES
45	a	517.95	4.88110	YES	YES
46	a	528.96	16.19087	YES	YES
47	a	541.68	2.52103	YES	YES
48	a	576.51	14.87999	YES	YES
49	a	588.03	27.43969	YES	YES
50	a	607.72	15.80706	YES	YES

TS_{39ia}

```
SCF Energy (au) BP86/SV(P)                    -2183.125430263
SCF Energy (au) PBE0/def2-TZVPP               -2182.704202665
SCF Energy (au) PBE0/def2-TZVPP               -2182.7106225495 (Toluene Correction)
Zero Point Energy (au)                        0.2592413
Chemical Potential (kJ mol-1)                 544.22
Dispersion Correction (au) PBE0/def2-TZVPP -0.04988049

xyz coordinates
37
C     1.6215828    0.0130222    2.8637253
C    -0.5552919    1.2926508    2.8384885
C     0.3174606    0.8443706    0.1505579
C    -0.5744828   -1.3750881    3.0075668
C     0.7261198   -1.6256458    0.3393932
C     1.7321269   -2.6532331    0.5664832
C     0.2907708   -4.3516046   -0.4519661
C    -0.7080140   -3.4292694   -0.6772707
C    -0.5704676   -2.0679884   -0.2341211
C    -1.5464973   -1.0762966   -0.3232095
C    -2.7473448   -1.0344039   -1.2212595
Mn    0.1193950   -0.0917515    1.9197951
O    -1.3230132    0.0275928    0.4074424
O     2.6326727    0.0755906    3.4481464
O    -0.9747757    2.1891404    3.4576197
C     1.1731735   -0.2558642    0.0899197
O    -1.0479384   -2.1858525    3.6955779
C     1.5161086   -3.9592029    0.1995257
H     2.2538640   -0.0815612   -0.0572638
C     0.3137643    2.1154790   -0.5445059
H    -1.6541747   -3.7437835   -1.1471547
H     0.1515968   -5.4016114   -0.7567116
H     2.2932074   -4.7178198    0.3894030
H     2.6884546   -2.3521242    1.0258805
H    -2.8894473   -1.9851829   -1.7739075
H    -2.6294363   -0.2159190   -1.9692374
H    -3.6716583   -0.8108671   -0.6431154
C     0.3086756    4.5642397   -1.9674185
C     0.8512904    3.4089457   -2.5600222
C     0.8486286    2.1954004   -1.8602233
C    -0.2351322    3.2887501    0.0356064
C    -0.2339798    4.4983769   -0.6702443
H     0.3059966    5.5188076   -2.5194580
H     1.2749335    3.4550747   -3.5766716
H     1.2615609    1.2851469   -2.3259539
H    -0.6594449    3.2401770    1.0493161
H    -0.6602843    5.4023044   -0.2047327

$vibrational spectrum
#  mode     symmetry     wave number   IR intensity    selection rules
#                         cm**(-1)        km/mol          IR     RAMAN
     1        a            -313.32         0.00000        YES     YES
     2                        0.00         0.00000         -       -
     3                        0.00         0.00000         -       -
     4                        0.00         0.00000         -       -
     5                        0.00         0.00000         -       -
     6                        0.00         0.00000         -       -
     7                        0.00         0.00000         -       -
     8        a              31.63         0.03949        YES     YES
     9        a              40.30         0.01775        YES     YES
    10        a              53.45         0.53751        YES     YES
    11        a              60.98         0.12144        YES     YES
    12        a              69.36         0.04757        YES     YES
```

13	a	80.92	0.16401	YES	YES
14	a	88.84	0.18539	YES	YES
15	a	91.39	0.29083	YES	YES
16	a	98.17	0.47781	YES	YES
17	a	104.25	0.53446	YES	YES
18	a	122.27	0.27990	YES	YES
19	a	147.04	0.95015	YES	YES
20	a	154.86	1.55796	YES	YES
21	a	192.89	0.67278	YES	YES
22	a	223.70	0.31201	YES	YES
23	a	235.13	0.54397	YES	YES
24	a	257.58	0.44732	YES	YES
25	a	279.00	0.69986	YES	YES
26	a	318.53	1.92914	YES	YES
27	a	387.67	4.46802	YES	YES
28	a	403.37	0.08995	YES	YES
29	a	417.51	2.41069	YES	YES
30	a	432.74	1.95993	YES	YES
31	a	441.60	2.11914	YES	YES
32	a	461.74	0.87129	YES	YES
33	a	477.70	3.83105	YES	YES
34	a	478.89	0.99679	YES	YES
35	a	482.93	1.29028	YES	YES
36	a	501.38	0.56334	YES	YES
37	a	510.65	3.16652	YES	YES
38	a	520.78	7.08188	YES	YES
39	a	535.43	14.46453	YES	YES
40	a	542.00	5.87021	YES	YES
41	a	566.54	4.99136	YES	YES
42	a	581.36	9.04895	YES	YES
43	a	603.70	18.18185	YES	YES
44	a	614.09	33.72873	YES	YES
45	a	631.46	6.20273	YES	YES
46	a	642.41	31.94297	YES	YES
47	a	657.03	5.78668	YES	YES
48	a	685.30	47.75021	YES	YES
49	a	688.80	33.16830	YES	YES
50	a	701.59	14.32620	YES	YES

TS$_{39ja}$

SCF Energy (au) BP86/SV(P) -2452.101612798
SCF Energy (au) PBE0/def2-TZVPP -2451.637427819
SCF Energy (au) PBE0/def2-TZVPP -2451.6455080397 (Toluene Correction)
Zero Point Energy (au) 0.3446137
Chemical Potential (kJ mol^{-1}) 746.48
Dispersion Correction (au) PBE0/def2-TZVPP -0.06671155

xyz coordinates
48

C	1.6911518	0.6228639	2.9599087
C	-0.5381033	1.8319302	2.8699088
C	0.5156193	1.3664366	0.2758119
C	-0.4804033	-0.7971369	3.1531878
C	0.8488824	-1.1533185	0.4898860
C	1.9238833	-2.1593889	0.7807363
C	-0.4299699	-1.6306059	-0.0101577
C	-1.4614591	-0.7019179	-0.1204781
C	-2.6568288	-0.7785329	-0.9969108
Mn	0.1931996	0.4441162	2.0089345
O	-1.3093976	0.4176004	0.5715432
O	2.6893899	0.7487136	3.5529774
O	-1.0018127	2.7373071	3.4424823
C	1.2831257	0.2088211	0.1935954
O	-0.9329596	-1.5831127	3.8847944
H	2.3746248	0.3488222	0.0759198
C	0.5527362	2.6114123	-0.4457787
C	-4.9714533	-0.8042816	-2.6347098
C	-4.0882095	-1.8996863	-2.6367533
C	-2.9367838	-1.8859868	-1.8347872
C	-3.5595053	0.3131106	-0.9985137
C	-4.6981174	0.3043616	-1.8148038
H	-5.8716749	-0.8147227	-3.2716781
H	-4.2965941	-2.7715272	-3.2790539
H	-2.2505973	-2.7475411	-1.8679982
H	-3.3418301	1.1706181	-0.3448872
H	-5.3818132	1.1696513	-1.8054271
H	-0.4897308	-2.6480544	-0.4272459
C	0.6920922	5.0397000	-1.8938761
C	0.9948020	3.8226459	-2.5346432
C	0.9269590	2.6209065	-1.8209363
C	0.2278337	3.8473862	0.1774812
C	0.3075455	5.0470284	-0.5391237
H	0.7528175	5.9872565	-2.4544869
H	1.2854998	3.8151227	-3.5979841
H	1.1568459	1.6635327	-2.3166701
H	-0.0799994	3.8505724	1.2334426
H	0.0669994	5.9995647	-0.0392820
C	3.9630695	-4.0219373	1.4494328
C	4.2023586	-3.0250814	0.4899196
C	3.1928921	-2.1067782	0.1579381
C	1.6877952	-3.1905233	1.7206998
C	2.6976743	-4.1047987	2.0573633
H	4.7601895	-4.7329263	1.7225311
H	5.1837171	-2.9583326	-0.0085284
H	3.3961008	-1.3491768	-0.6167166
H	0.7101998	-3.2455799	2.2256543
H	2.4992385	-4.8785330	2.8172817

$vibrational spectrum

# mode	symmetry	wave number	IR intensity	selection rules	
#		cm**(-1)	km/mol	IR	RAMAN
1	a	-101.84	0.00000	YES	YES
2		0.00	0.00000	-	-
3		0.00	0.00000	-	-
4		0.00	0.00000	-	-
5		0.00	0.00000	-	-
6		0.00	0.00000	-	-
7		0.00	0.00000	-	-
8	a	19.82	0.04668	YES	YES
9	a	26.78	0.15666	YES	YES
10	a	31.80	0.37669	YES	YES
11	a	40.20	0.20557	YES	YES
12	a	43.70	0.03615	YES	YES
13	a	47.80	0.24022	YES	YES
14	a	53.12	0.20842	YES	YES
15	a	58.20	0.16989	YES	YES
16	a	76.82	0.14507	YES	YES
17	a	88.16	0.32927	YES	YES
18	a	92.55	0.19181	YES	YES
19	a	101.18	0.19223	YES	YES
20	a	115.84	0.51378	YES	YES
21	a	134.32	0.28560	YES	YES
22	a	142.23	0.83050	YES	YES
23	a	169.68	0.93431	YES	YES
24	a	186.56	0.09386	YES	YES
25	a	206.28	1.66894	YES	YES
26	a	229.73	0.47084	YES	YES
27	a	237.12	0.49147	YES	YES
28	a	250.38	1.77895	YES	YES
29	a	261.02	3.38600	YES	YES
30	a	304.35	1.70317	YES	YES
31	a	353.95	5.54405	YES	YES
32	a	390.73	6.44978	YES	YES
33	a	400.70	0.74591	YES	YES
34	a	402.44	0.39983	YES	YES
35	a	403.13	0.39119	YES	YES
36	a	432.59	2.42700	YES	YES
37	a	445.95	6.51090	YES	YES
38	a	453.39	3.00818	YES	YES
39	a	469.58	1.35279	YES	YES
40	a	480.02	0.97809	YES	YES
41	a	484.42	0.28655	YES	YES
42	a	491.23	0.85186	YES	YES
43	a	510.47	5.91320	YES	YES
44	a	521.15	4.56488	YES	YES
45	a	532.73	7.72744	YES	YES
46	a	544.33	11.44481	YES	YES
47	a	556.94	15.98299	YES	YES
48	a	572.11	11.88796	YES	YES
49	a	592.99	11.20011	YES	YES
50	a	610.97	1.19647	YES	YES
