# Supplementary material for: A comprehensive understanding of carbon–carbon bond formation by alkyne migratory insertion into manganacycles
Source: Chem Sci. 2022 Jul 8;13(34):9902–13. doi: 10.1039/d2sc02562k (PMC9431456; doi:10.1039/d2sc02562k)
Supplement: SC-013-D2SC02562K-s002 [file SC-013-D2SC02562K-s002.pdf]

Electronic Supplementary Material (ESI) for Chemical Science.
This journal is © The Royal Society of Chemistry 2022

Electronic Supporting Information for

A comprehensive understanding of carbon-carbon bond formation through alkyne migratory insertion into manganacycles.

L. Anders Hammarback,[a] Jonathan B. Eastwood,[a] Thomas J. Burden,[a] Callum J. Pearce,[a] Ian P. Clark,[b] Michael Towrie,[b] Alan Robinson,[c] Ian J. S. Fairlamb*[a] and Jason M. Lynam*[a]

[a] Department of Chemistry, University of York, Heslington, York, YO10 5DD, UK ian.fairlamb@york.ac.uk, jason.lynam@york.ac.uk

[b] Central Laser Facility, STFC Rutherford Appleton Laboratory, Harwell Science and Innovation Campus, Didcot, Oxfordshire, OX11 0QX, UK.

[c] Syngenta Crop Protection AG, Münchwilen, Breitenloh 5, 433, Switzerland.

Table of Contents

1 Synthetic Methodology S3
1.1 General Considerations S3
1.2 General Ligand Synthesis S4
1.3 Synthesis of 2-Methoxy-6-phenylpyridine S5
1.4 Synthesis of 2,6-Diphenylpyridine S7
1.5 General Synthesis of Manganese Complexes S9
1.6 Synthesis of Tetracarbonyl (2-methoxy-6-(phenyl-κ,C2)-pyridine-κ,N) manganese(I), 1f S10
1.7 Synthesis of Tetracarbonyl (2-(phenyl-κ,C2)-6-phenylpyridine-κ,N) manganese(I), 1g S12
1.8 Synthesis of Tetracarbonyl (phenyl-κ,C2)-phenylmethanimine-κ,N) manganese(I) 1h S14
1.9 Synthesis of Tetracarbonyl (2-(acetyl-κ,O)benzene-κ,C2) manganese(I), 1i S16
2 TRIR Spectroscopy S18
2.1 Methodology S18
2.2 Compound Numbering S20
2.3 Studies in neat PhC_2H S21
2.3.1 TRIR study of **1e** in Neat PhC_2H S21
2.4 Variable alkyne concentration studies S22
2.4.1 Collated rate constants and kinetic profiles for the reaction between **1b** and PhC_2H in toluene ... S22
2.4.2 Collated rate constants and kinetic profiles for the reaction between **1e** and PhC_2H in toluene ... S26
2.4.3 Collated rate constants and kinetic profiles for the reaction between **1e** and PhC_2Ph in heptane. S31
2.5 Studies at constant concentration of alkyne in toluene solution S38
2.5.1 TRIR study of **1b** and CyC_2H in toluene solution S38
2.5.2 TRIR study of **1b** and PhC_2Ph in toluene solution S41
2.5.3 TRIR study of **1b** and $^{n}BuC_2{}^{n}Bu$ in toluene solution S43
2.5.4 TRIR study of **1b** and CF_3-4-$C_6H_4C_2H$ in toluene solution S45
2.5.5 TRIR study of **1b** and F-4-$C_6H_4C_2H$ in toluene solution S47
2.5.7 TRIR study of **1b** and MeO-4-$C_6H_4C_2H$ in toluene solution S49
2.5.8 TRIR study of **1b** and $MeCO_2$-4-$C_6H_4C_2H$ in toluene solution S51
2.5.9 TRIR study of **1b** and Me_2N-4-$C_6H_4C_2H$ in toluene solution S53
2.5.10 TRIR study of **1e** and $^{n}BuC_2{}^{n}Bu$ in toluene solution S55
2.5.11 TRIR study of **1f** and PhC_2H in toluene solution S57
2.5.12 TRIR study of **1g** and PhC_2H in toluene solution S59
2.5.13 TRIR study of **1h** and PhC_2H in toluene solution S61
2.5.14 TRIR study of **1i** and PhC_2H in toluene solution S64
3 DFT Calculations S66
3.1 Methodology S66
3.2 Computational analysis of the vibrational modes for complexes **8ia** and **10ia** S67
3.3 Computational analysis of the formation of **8ja** and **10ja** S68
4 References S70

1 Synthetic Methodology

1.1 General Considerations

Solvents and Reagents

Commercially sourced reagents were purchased from Acros Organics, Alfa Aesar, Fluorochem, or Sigma-Aldrich and used as received unless otherwise stated. Dry hexane was obtained from a Pure Solv MD-7 solvent system and stored under nitrogen Petroleum ether refers to the fraction of petroleum that is collected at 40−60 °C. Reactions requiring anhydrous conditions were carried out using Schlenk techniques (high vacuum, liquid nitrogen trap on a standard in-house built dual line). Room-temperature upper and lower limits are stated as 13−25 °C, but typically 21 °C was recorded. Complexes **1a-e**, were prepared as described previously.[1,2]

Chromatography

Thin-layer chromatography (TLC) was carried out using Merck 5554 aluminum-backed silica plates (silica gel 60 F254), and spots were visualized using UV light (254 nm). Where necessary, plates were stained and heated with potassium permanganate, anisaldehyde, or vanillin as appropriate. Retention factors (R_f) are reported along with the solvent system used in parenthesis. Flash column chromatography was performed according to the method reported by Still et al.[3] using Fluorochem silica gel 60 (particle size 40−63 µm) and a solvent system as stated in the text.

Melting Points

Melting points were recorded using a Stuart digital SMP3 machine using a temperature ramp of 3 °C min^{-1}.

Infrared Spectroscopy

Infrared spectra were obtained using a Unicam Research Series FTIR (KBr IR) or a Bruker ALPHA-Platinum FTIR Spectrometer with a platinum−diamond ATR sampling module. Where indicated, reactions were monitored in situ using a Mettler Toledo ReactIR ic10 with a K6 conduit SiComp (silicon) probe and MCT detector.

Nuclear Magnetic Resonance Spectroscopy

NMR spectra were obtained in the solvent indicated using a JEOL ECX400 or JEOL ECS400 spectrometer (400, and 101 MHz for ^{1}H and ^{13}C respectively) or a Bruker 500 (500 and 125 MHz for ^{1}H and ^{13}C respectively). Chemical shifts are reported in parts per million and were referenced to the residual non-deuterated solvent of the deuterated solvent used ($CHCl_3$ TMH = 7.26 and TMC = 77.16 ($CDCl_3$). Spectra were typically run at a temperature of 298 K. All ^{13}C NMR spectra were obtained with ^{1}H decoupling. NMR spectra were processed using MestReNova software (versions 11.0.3-18688, 2017 and 12.0.3-21384, 2018). For the ^{1}H NMR spectra,

the resolution varies from 0.15 to 0.5 Hz; the coupling constants have been quoted to ±0.5 Hz in all cases for consistency. ^{1}H NMR chemical shifts are reported to two decimal places, and ^{13}C NMR chemical shifts are reported to one decimal place.

Mass Spectrometry

Electrospray ionisation (ESI) mass spectrometry (MS) spectra were measured using a Bruker Daltronics micrOTOF MS, Agilent series 1200LC with electrospray ionization (ESI and APCI) or on a Thermo LCQ using electrospray ionization, with <5 ppm error recorded for all HRMS samples. Liquid induction field desorption ionisation (LIFDI) mass spectrometry was carried out using a Waters GCT Premier MS Agilent 7890A GC. Mass spectral data are quoted as the mass to charge ratio (m/z) in Daltons along with the relative peak height in brackets (base peak = 100). High resolution mass spectra (HRMS) are reported with <5 ppm error.

1.2 General Ligand Synthesis

The method was adapted from a literature procedure.[4] To a Schlenk tube under nitrogen was added the arylbromine (1.0 eq.), aryl boronic acid (1.5 eq.), $Pd_3(OAc)_6$ (0.5–2 mol% with respect to Pd), K_3PO_4 (2 eq.) and deoxygenated ethylene glycol (6 ml mmol^{-1}). The reaction was heated to 80 °C for 150 min or 16 hours and was thereafter allowed to cool to room temperature. Water (7.5 ml mmol^{-1}) and saturated brine (7.5 ml mmol^{-1}) was added and the product was extracted using CH_2Cl_2 (4 × 10 ml mmol^{-1}). The CH_2Cl_2 layers was dried over $MgSO_4$ and the solvent was removed under reduced pressure. The crude material was purified using flash column chromatography to yield the product.

1.3 Synthesis of 2-Methoxy-6-phenylpyridine

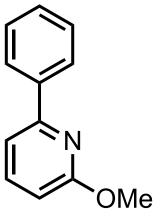

Synthesised using the general ligand synthesis procedure from 2-bromo-6-methoxypyridine (2.5 ml, 3.76 g, 20.0 mmol, 1 eq.), phenylboronic acid (3.66 g, 30.0 mmol, 1.5 eq.) and $Pd_3(OAc)_6$ (23 mg, 0.03 mmol, 0.5 mol% with respect to Pd). The crude material was purified by flash column chromatography (petrol/Et_2O, 97.5:2.5, *v*/*v*) to afford a colourless oil (2.69 g, 73%).

R_f 0.28 (petrol/Et_2O, 97.5:2.5, *v*/*v*); ^{1}H NMR (400 MHz, $CDCl_3$, δ): 8.07–8.03 (m, 2H), 7.63 (dd, J = 8.0, 7.5 Hz, 1H), 7.49–7.44 (m, 2H), 7.42–7.37 (m, 1H), 7.35 (dd, J = 7.5, 0.5 Hz, 1H), 6.70 (dd, J = 8.0, 0.5 Hz, 1H), 4.05 (s, 3H); ^{13}C NMR (101 MHz, $CDCl_3$, δ): 163.9, 154.8, 139.3, 139.2, 129.0, 128.7, 126.8, 112.9, 109.4, 53.4; ESI–MS *m*/*z* (ion, %): 186 ($[M+H]^+$, 100); ESI–HRMS *m*/*z*: 186.0910 (calc. for $C_{12}H_{12}NO$ 186.0913); IR (liquid–state): 3062, 2946, 2848, 1599, 1574, 1465, 1448, 1429, 1405, 1324, 1289, 1251, 1178, 1151, 1077, 1031, 1018, 986, 920, 878, 805, 760, 691, 659, 622, 607.

The analytical data obtained were in accordance with the literature.[5]

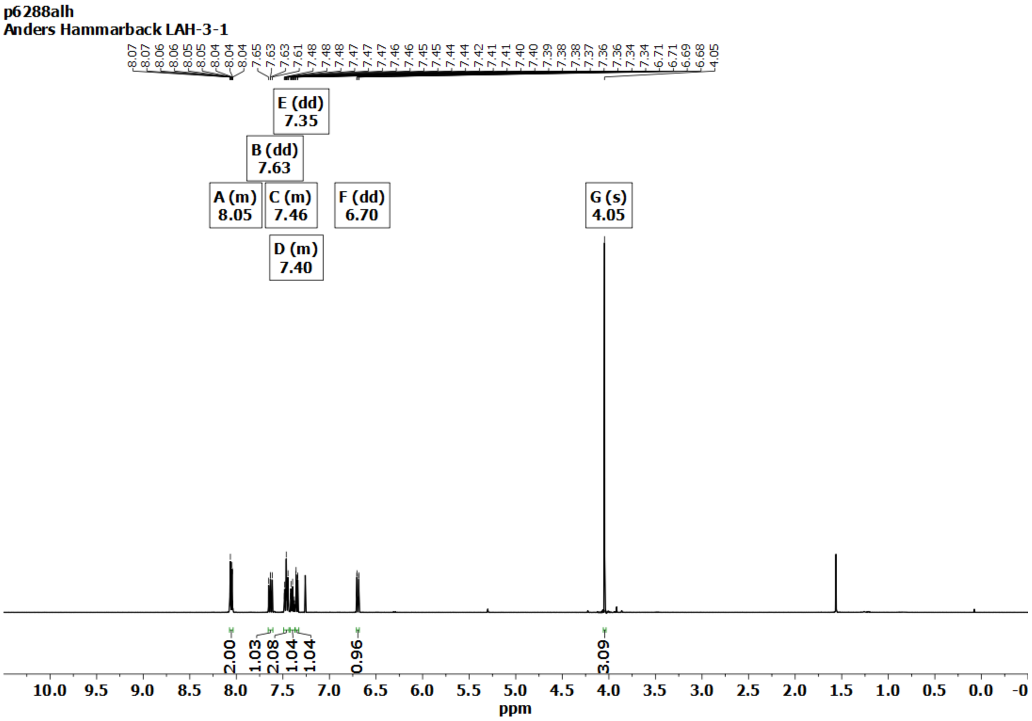

Figure S1 ^{1}H NMR spectrum of 2-methoxy-6-phenylpyridine (400 MHz, $CDCl_3$).

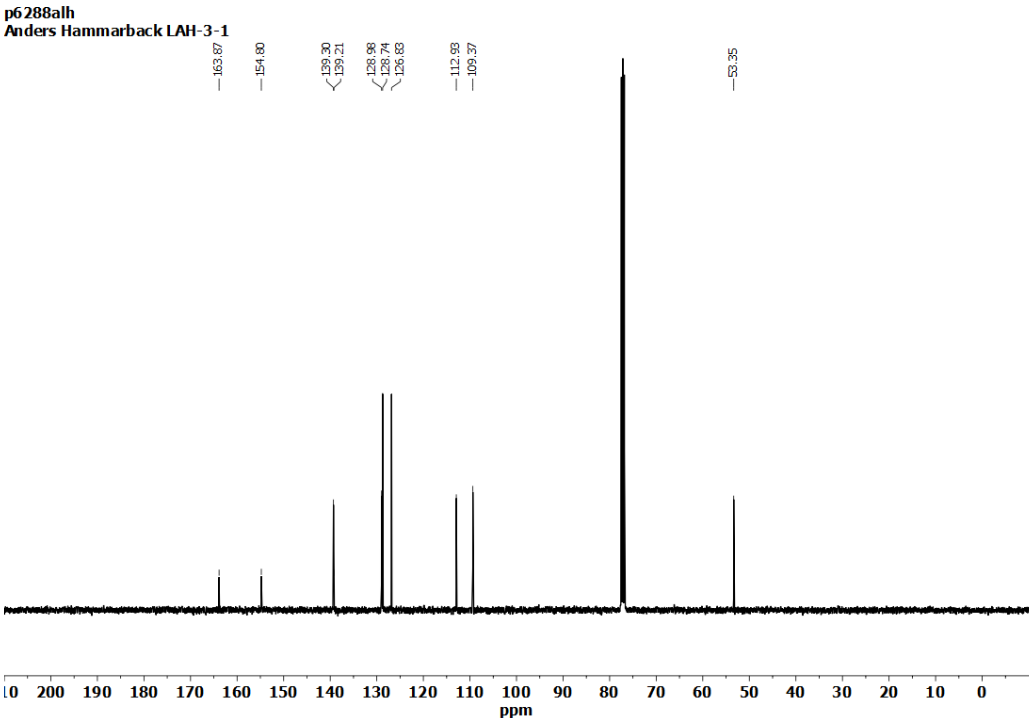

Figure S2 ^{13}C NMR spectrum of 2-Methoxy-6-phenylpyridine (101 MHz, $CDCl_3$).

1.4 Synthesis of 2,6-Diphenylpyridine

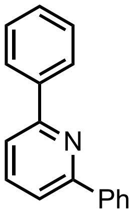

Synthesised using the general ligand synthesis procedure from 2,6-dibromopyridine (2.37 g, 10.0 mmol, 1 eq.), phenylboronic acid (2.28 g, 15.0 mmol, 1.5 eq.) and $Pd_3(OAc)_6$ (12 mg, 0.02 mmol, 0.5 mol% with respect to Pd). The crude material was purified by flash column chromatography (petrol/Et_2O, 9:1, *v*/*v*) to afford a white solid (1.63 g, 93%).

R_f 0.33 (petrol/Et_2O, 9:1, *v*/*v*); ^{1}H NMR (400 MHz, $CDCl_3$, δ): 8.18–8.14 (m, 4H), 7.83 (dd, *J* = 8.5, 7.0 Hz, 1H), 7.70 (d, J = 7.5 Hz, 2H), 7.54–7.48 (m, 4H), 7.46–7.41 (m, 2H); ^{13}C NMR (101 MHz, $CDCl_3$, δ): 157.0, 139.6, 137.6, 129.1, 128.8, 127.1, 118.8; ESI–MS *m*/*z* (ion, %): 232 ($[M+H]^+$, 100), 254 ($[M+Na]^+$, 3); ESI–HRMS *m*/*z*: 232.1118 $[M+H]^+$ (calc. for $C_{17}H_{13}N$ 232.1121); IR (liquid–state): 3053, 3035, 1597, 1563, 1491, 1450, 1436, 1390, 1314, 1257, 1186, 1167, 1157, 1102, 1074, 1025, 987, 973, 924, 821, 775, 753, 739, 696, 626, 611.

The analytical data obtained were in accordance with the literature.[5]

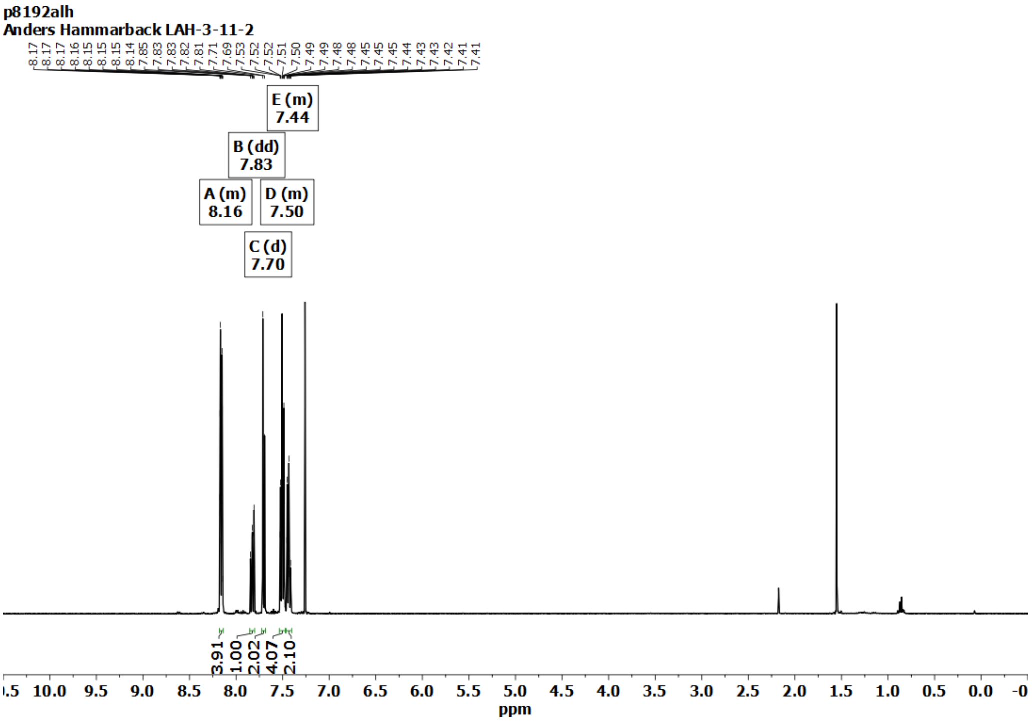

Figure S3 ^{1}H NMR spectrum of 2,6-diphenylpyridine (400 MHz, $CDCl_3$)

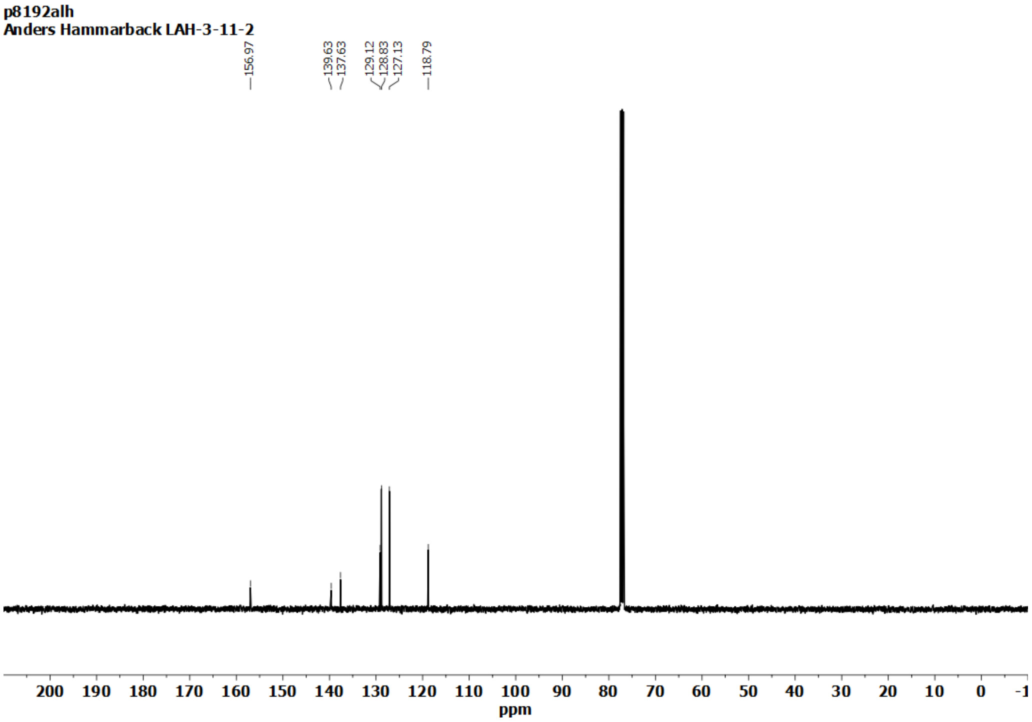

Figure S4 ^{13}C NMR spectrum of 2,6-diphenylpyridine (101 MHz, $CDCl_3$).

1.5 General Synthesis of Manganese Complexes

This method was adapted from a literature procedure.[4] To a Schlenk tube under nitrogen was added $MnBn(CO)_5$ (1 eq.), ligand to be cyclomanganated (1 eq.) and dry deoxygenated solvent. The reaction mixture was excluded from light and heated to reflux for 6-24 hours, until completion of the reaction given from monitoring by IR. When the reaction was complete, the reaction mixture was allowed to cool to room temperature and was filtered through cotton wool. CH_2Cl_2 was used to rinse the flask and cotton wool, before the solvent was removed under reduced pressure. The crude material was purified by flash column chromatography to afford the product. If significant amounts of silicon grease or oil was present, the material was dissolved in acetonitrile and washed with a small amount of hexane, before the acetonitrile layer was removed under reduced pressure.

1.6 Synthesis of Tetracarbonyl (2-methoxy-6-(phenyl-κ,C2)-pyridine-κ,N) manganese(I), 1f

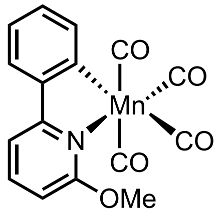

Synthesised using the general procedure to prepare manganese complexes from $MnBn(CO)_5$ (2.50 g, 9.15 mmol, 1 eq.), 2-methoxy-6-phenylpyridine (33 mg, 0.18 mmol, 1 eq.) and hexane (2.5 ml). The crude material was purified by flash column chromatography (petrol/Et_2O, 9:1, *v*/*v*) to afford a yellow solid (38 mg, 60%).

Mp 118–120 °C dec; R_f 0.02 (petrol/Et_2O, 9:1, *v*/*v*); ^{1}H NMR (500 MHz, $CDCl_3$, δ): 8.03 (d, J = 7.5 Hz, 1H), 7.81–7.73 (m, 2H), 7.54 (d, J = 8.0 Hz, 1H), 7.29–7.23 (m, 1H), 7.15 (dd, J = 7.5, 7.5, 1H), 6.58 (d, J = 8.0 Hz, 1H), 4.04 (s, 3H); ^{13}C NMR (126 MHz, $CDCl_3$, δ): 222.0, 214.8, 214.3, 175.9, 166.5, 166.2, 147.0, 130.0, 124.7, 123.8, 112.2, 102.6, 60.0; LIFDI-MS *m*/*z* (ion, %): 351 ($[M]^+$, 100); IR (CH_2Cl_2 solution, cm^{-1}): 2072, 1983, 1931, 1181, 1132, 1096, 1054, 1028, 1017, 1001.

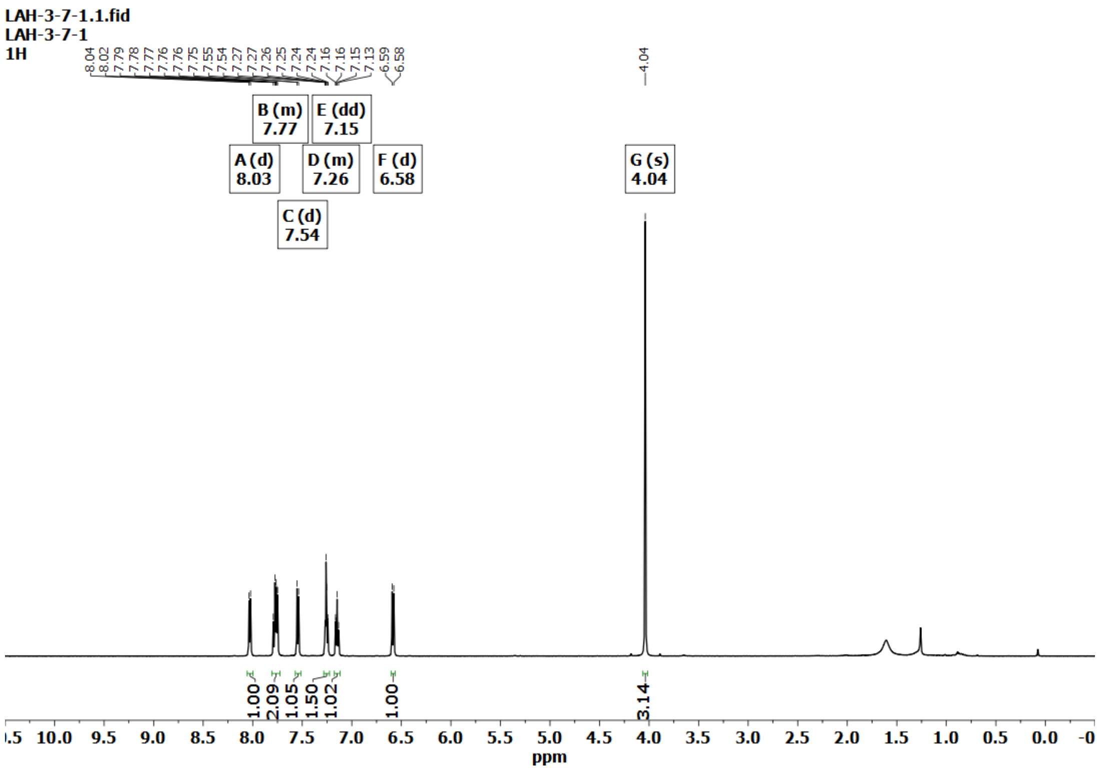

Figure S5 ^{1}H NMR spectrum of **1f** (500 MHz, $CDCl_3$).

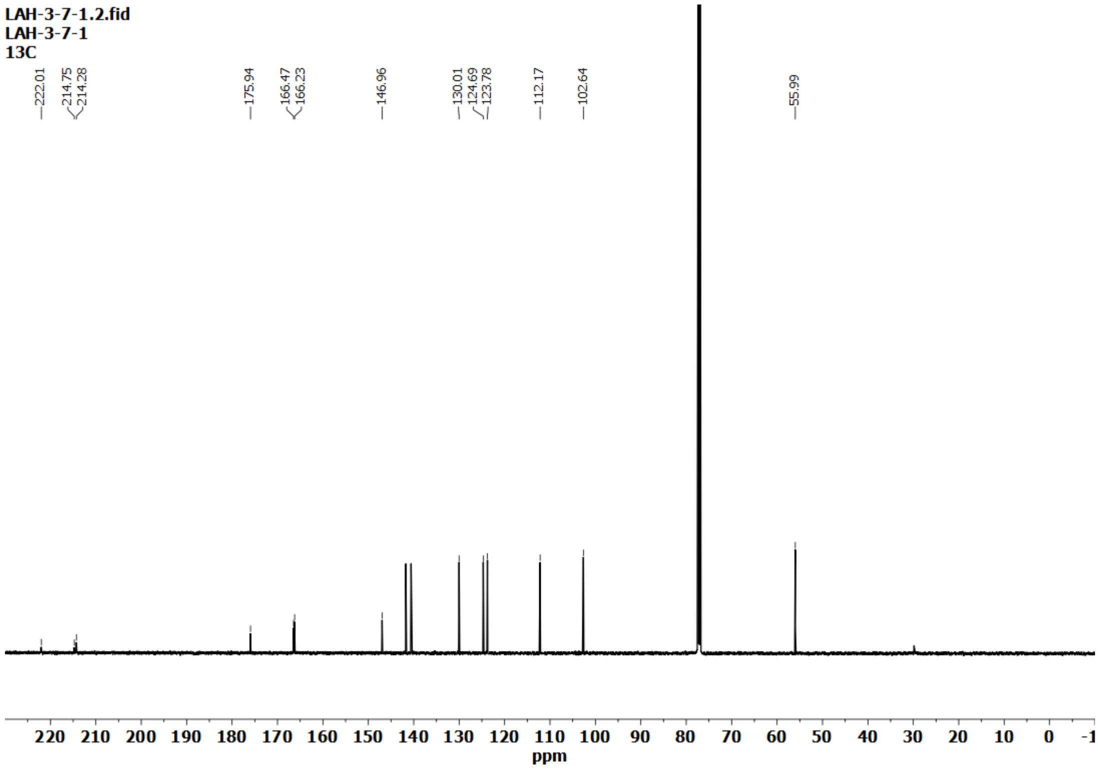

Figure S6 ^{13}C NMR spectrum of **1f** (126 MHz, $CDCl_3$).

1.7 Synthesis of Tetracarbonyl (2-(phenyl-κ,C2)-6-phenylpyridine-κ,N) manganese(I), 1g

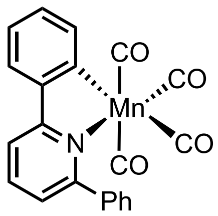

Synthesised using the general procedure to prepare manganese complexes from MnBn(CO)$_5$ (72 mg, 0.25 mmol, 1 eq.), 2,6-diphenylpyridine (58 mg, 0.25 mmol, 1 eq.) and hexane (5 ml). The crude material was purified by flash column chromatography (petrol/EtOAc, 9:1, *v*/*v*) to afford a yellow solid (56 mg, 57%).

Mp 132–133 °C dec; R_f 0.24 (petrol/EtOAc, 9:1, *v*/*v*); ^{1}H NMR (500 MHz, CDCl$_3$, δ): 8.01 (dd, *J* = 7.5, 1.5 Hz, 1H), 7.94 (dd, *J* = 8.0, 1.5 Hz, 1H), 7.85–7.79 (m, 2H), 7.56–7.51 (m, 3H), 7.43–7.39 (m, 2H), 7.29 (td, *J* = 7.5, 1.5 Hz, 1H), 7.20 (td, *J* = 7.5, 1.5 Hz, 1H), 7.29 (dd, *J* = 7.5, 1.5 Hz, 1H); ^{13}C NMR (126 MHz, CDCl$_3$, δ): 221.6, 214.6, 209.2, 175.0, 166.8, 165.6, 147.3, 143.7, 141.5, 137.5, 130.1, 129.7, 128.9, 128.7, 125.0, 124.0, 124.0, 117.9; LIFDI-MS *m*/*z* (ion, %): 397 ([M]$^+$, 100); LIFDI-HRMS *m*/*z*: 397.01389 [M]$^+$ (calculated for $C_{21}H_{12}NO_4Mn$ 397.01413); IR (CH_2Cl_2 solution, cm^{-1}): 2072, 1985, 1933, 1215, 1180.

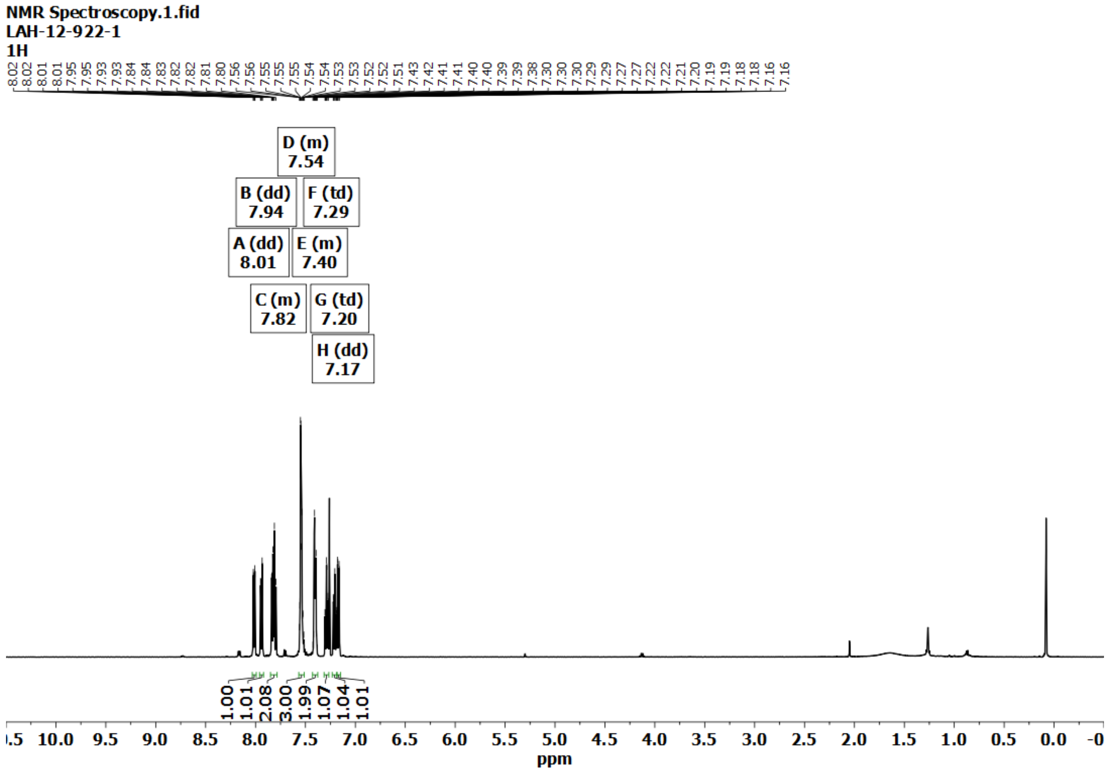

Figure S7 ^{1}H NMR spectrum of **1g** (500 MHz, $CDCl_3$).

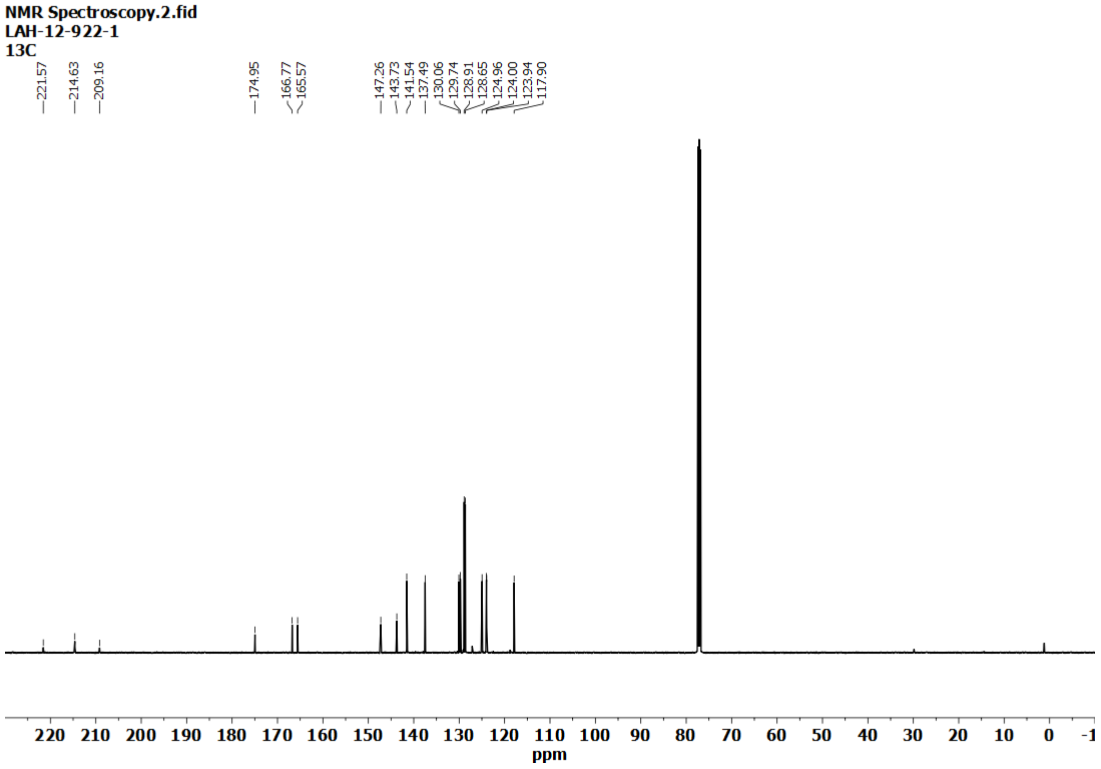

Figure S8 ^{13}C NMR spectrum of **1g** (126 MHz, $CDCl_3$).

1.8 Synthesis of Tetracarbonyl (phenyl-κ,C2)-phenylmethanimine-κ,N) manganese(I) 1h

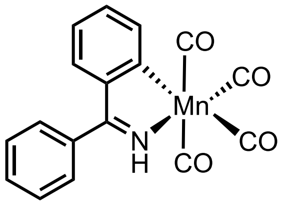

Complex **1h** was prepared in a two-step process involving the initial preparation of the pro-ligand, diphenylmethanimine, followed by a cyclomanganation reaction.

To an oven dried Schlenk equipped with a stirrer bar, that was evacuated and backfilled with N_2 five times, was added benzonitrile (1 equiv., 4.5 mmol, 0.46 mL), dry degassed THF (30 mL), and phenylmagnesium bromide (1.0 M in THF) (1 equiv., 4.5 mmol, 4.5 mL) dropwise. The reaction mixture was stirred under reflux for 16 hours, before being cooled down to room temperature. Dry methanol (3 mL) was added and the reaction mixture was stirred for 30 minutes, diluted with diethylether (60 mL), filtered through a celite® pad and solvent removed in *vacuo* to yield a yellow oil. The crude diphenylmethanimine (1 equiv., 1 mmol, 0.19 mL) and benzylmanganese(I)pentacarbonyl (1 equiv., 1 mmol, 286 mg) were added to an oven dried Schlenk equipped with a stirrer bar, that was evacuated and backfilled with N_2 five times. Dry degassed toluene (30 mL) was added and the reaction mixture heated to 100 °C for 2 hours. Solvent was removed in *vacuo*, the crude material was purified *via* flash column chromatography (hexane/EtOAc, 70:30, v/v) to afford a yellow solid (290 mg, 76% yield). Rf 0.43 (hexane/EtOAc, 70:30, v/v). ^{1}H NMR ($CDCl_3$, 500 MHz) δ (ppm); 7.15 (1H, td, *J* = 7.5, 1.0), 7.36 (1H, td, *J* = 7.5, 1.0), 7.54 (6H, m), 8.09 (1H, d, *J* = 7.5), 8.36 (1H, s). ^{13}C NMR ($CDCl_3$, 126 MHz) δ (ppm); 123.5, 127.6, 128.9, 130.8, 131.0, 131.3, 136.9, 141.8, 145.9, 183.7, 190.6, 213.5, 215.1, 220.0 LIFID-MS m/z: (M^+, 100%), 346.99848 (calculated for $C_{17}H_{10}NO_4Mn$ 346.99903) IR (hexane, cm^{-1}) 2076, 1991, 1983, 1944, 1750.

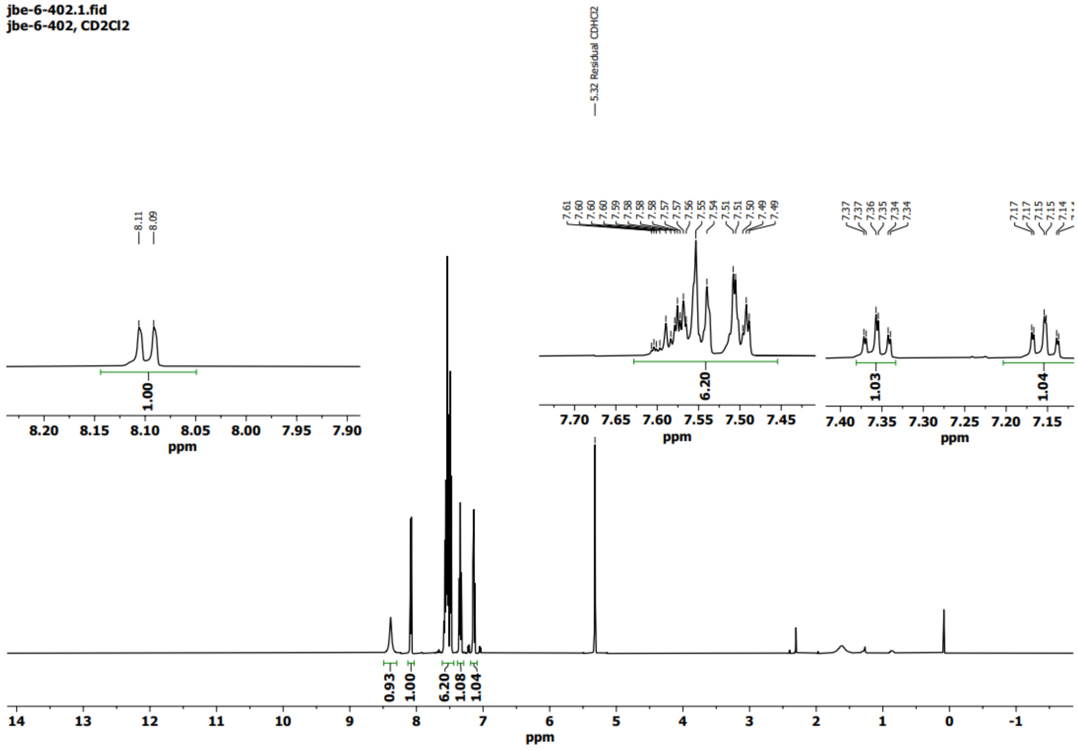

Figure S9 ^{1}H NMR spectrum of **1h** (500 MHz, $CDCl_3$).

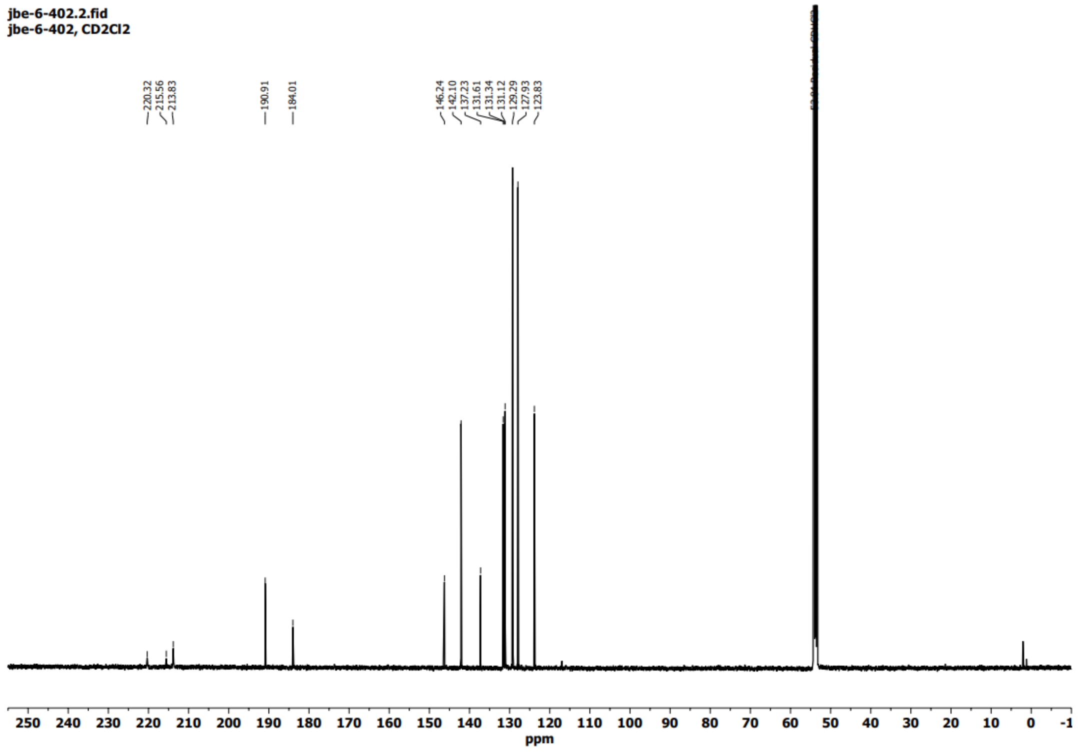

Figure S10 ^{13}C NMR spectrum of **1h** (126 MHz, $CDCl_3$).

1.9 Synthesis of Tetracarbonyl (2-(acetyl-κ,O)benzene-κ,C2) manganese(I), 1i

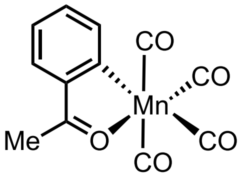

Synthesised using the general procedure to prepare manganese complexes from $MnBn(CO)_5$ (143 mg, 0.5 mmol, 1 eq.), acetophenone (58 µl, 0.5 mmol, 1 eq.) and hexane (10 ml). The crude material was purified by flash column chromatography (petrol/CH_2Cl_2, 9:1, *v/v*) to afford a yellow solid (79 mg, 55%).

Mp 116–117 °C dec; R_f 0.24 (petrol/CH_2Cl_2, 9:1, *v/v*); ^{1}H NMR (500 MHz, $CDCl_3$, δ): 7.40 (d, *J* = 7.5, 1H), 7.15 (d, *J* = 7.5 Hz, 2H), 6.72 (dd, *J* = 7.5, 7.5 Hz, 1H), 6.48 (dd, *J* = 7.5, 7.5 Hz, 1H); ^{13}C NMR (126 MHz, $CDCl_3$, δ): 220.4, 215.9, 212.2, 210.8, 192.8, 144.7, 140.9, 133.2, 131.0, 123.2, 23.9; LIFDI-MS *m/z* (ion, %): 286 ($[M]^+$, 100); LIFDI-HRMS *m/z*: 285.96780 $[M]^+$ (calculated for $C_{12}H_7NO_5Mn$ 285.96685); IR (CH_2Cl_2 solution, cm^{-1}): 2082, 1993, 1938, 1581, 1539, 1442, 1370, 1323.

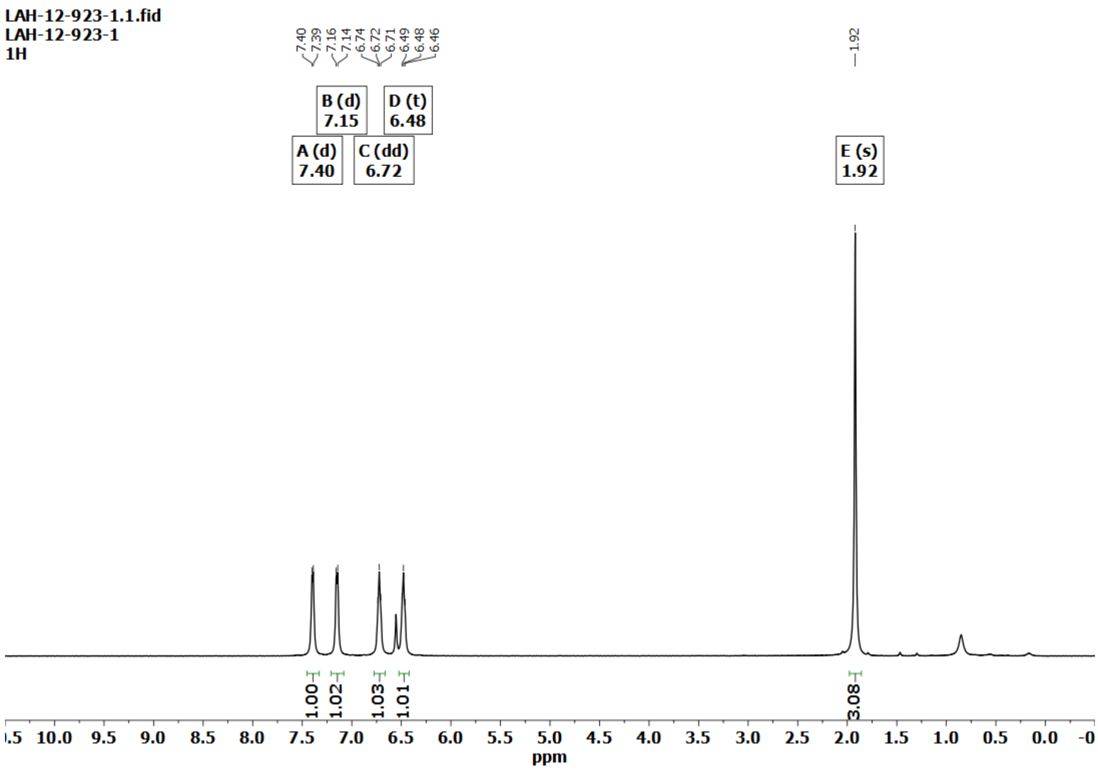

Figure S11 ^{1}H NMR spectrum of **1i** (500 MHz, $CDCl_3$).

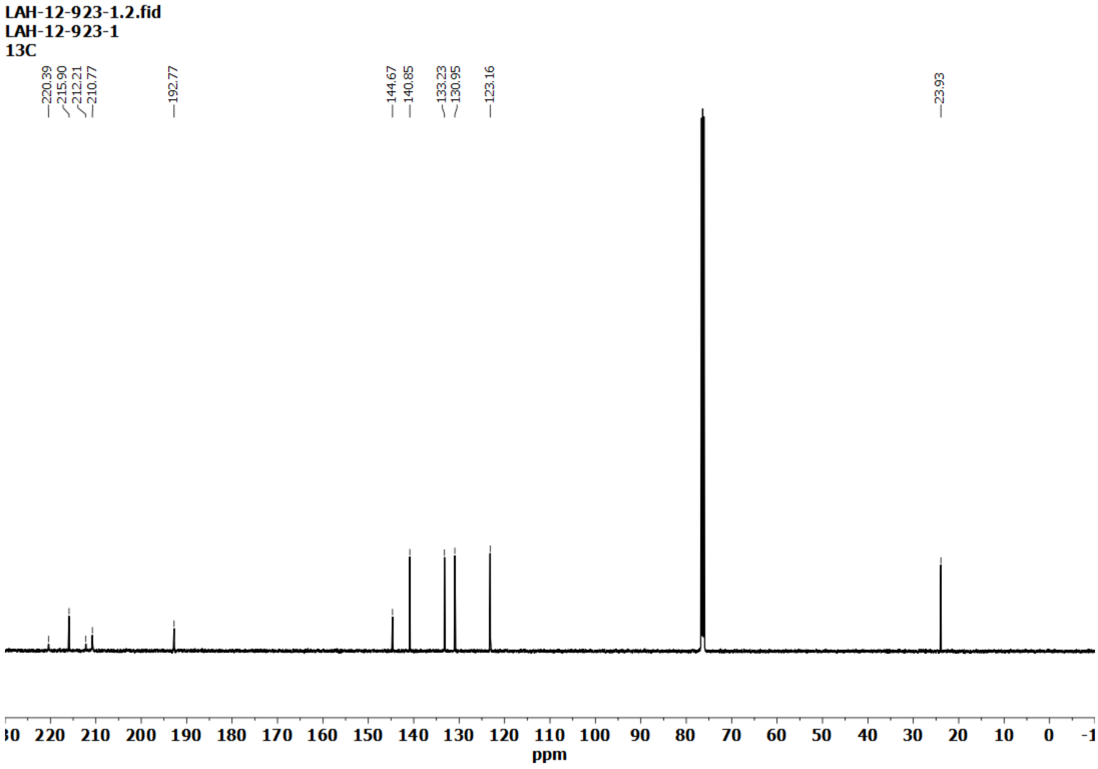

Figure S12 ^{13}C NMR spectrum of **1i** (126 MHz, $CDCl_3$).

2 TRIR Spectroscopy

2.1 Methodology

Time-resolved infra-red spectra were recorded on either the ULTRA A or LIFEtime instrument in the ULTRA facility at the Rutherford Appleton Laboratories using the Time-Resolved Multiple Probe Spectroscopy mode of operation, (hereafter $TR^{M}PS$).[6]

The experiments on ULTRA A were driven by a 10 kHz repetition rate Ti:Sapphire amplifier (Thales) as a probe source, producing 40 fs pulses at 800 nm. The Ti:Sapphire laser output was used to pump an Optical Parametric Amplifier (TOPAS, Light Conversion Ltd.) followed by a AgGaS Difference Frequency Mixing stage which produced tuneable mid-IR probe beam of ~ 500 cm^{-1} useable bandwidth. The IR probe beam was split to form reference and probe beams, which were passed through spectrographs onto MCT array detectors (IR Associates). The probe beam spot size at sample was ca. 80x80 μm^2. High speed data acquisition systems (Quantum Detectors) allowed 10 kHz acquisition and processing of the probe and reference pulses to generate a pump-on pump-off infrared absorption difference signal. The excitation source for the TRIR experiments was the output of the 1 kHz titanium sapphire amplifier (Spectra Physics Spitfire XP, 100 fs pulse length) equipped with another TOPAS OPA, pulse energy at sample attenuated down to 1 µJ and focused down to ca. 150 × 150 μm^2 spot). Both ULTRA amplifier and Spitfire amplifier were optically synchronised by sharing the same seed from 68 MHz Ti:Sapphire oscillator. The seed beam was delayed with an optical delay line before the 1 kHz amplifier to accommodate for the 100 fs – 14.7 ns time delays between pump and probe. To go beyond 14.7 ns and up to 100 µs, subsequent seed pulses are selected from the 68 MHz seed pulse train accompanied by the appropriate setting of the optical delay line. The polarisation of the excitation beam at sample was set to be at 54.7° with respect to the probe.

For the LIFEtime spectrometer, the pump source was the output of a Yb:KGW amplifier providing 15W, 260 fs pulses at 1030 nm with a 100 kHz repetition rate (Pharos). This was used to drive a BBO-based 515 nm pumped optical parametric amplifier (OPA) to deliver pulses at 355 nm. The pump beam was collimated, travelled over a computer programmable 0 - 16 ns optical delay (1200 mm long, double pass), and focused onto the sample. The pump energy at the sample was attenuated down to 500 nJ and focused down to a 120 × 120 μm^2 spot. The probe source was the output of a Yb:KGW amplifier providing 6 W, 180 fs pulses at 1030 nm with a 100 kHz repetition rate (Pharos). This drove two 3 W BBO/KTA based OPAs. The two Pharos sources share a common 80 MHz oscillator to allow for pump-probe delay steps of 12.5 ns. The probe beam was split to provide probe and reference pulses. To go beyond pump-probe delays of 12.5 ns, subsequent seed pulses were selected from the 80 MHz oscillator. Data were collected using pump-probe delays ranging from 1 ps to 988.5 µs. The probe beams were collimated, synchronised by a fixed optical delay, and focused by a gold

parabolic mirror onto the sample. The three beams were overlapped on the sample using a 50 μm pinhole. The probe beams were measured by two separate 128-element detectors. To cover the full spectroscopic window required, data from different detector positions were combined to generate the required spectra.

Solutions of the manganese complexes were prepared at a typical concentration 1.87 mmol dm^{-3} in toluene (e.g 12 mg of **1b** in 20 ml of toluene) in a thick-walled Duran flask from which light was excluded by covering in aluminium foil. The solution was then flowed (peristaltic pump) through a Harrick cell fitted with a 100 μm Teflon spacer. To ensure that the photoproducts were not themselves irradiated, data were acquired while continuously flowing and rastering the sample. The samples used for concentration-dependent experiments were prepared by dilution from stock solutions prepared using volumetric flasks and run in a randomised order. Typically, 10 ml of a stock solution of **1b** (121.4 mg in 100 ml of toluene) and the appropriate amount of a PhC_2H stock solution (2.3737 g in 25 ml toluene) were mixed and then diluted to a volume of 20 ml. This was then transferred to a Duran flask for the TRIR experiment. Experiments with a constant concentration of alkyne and manganese carbonyl complex were performed in a similar manner. For experiments between **1b** and a range of alkynes, a stock of **1b** (121.6 mg in 100 ml of toluene) was prepared. 10 ml of this stock was then taken and an accuraltey weighed amount of alkyne added. The total volume of the sample was then made to 20 ml.

Data were initially visualised in the ULTRA View version 2 software,[7] where baseline-correction was undertaken. The resulting spectra were then exported as comma-separated variable files into Origin2019.[8] The spectra were calibrated against samples of polystyrene (200 μm thick) and 1,4-dioxane. Kinetic analysis was performed by fitting to the *expgro*, *expdec* or *expgrodec* functions in Origin2019. All quoted errors are 95 % confidence limits.

In the case of the experiments between **1b** and CyC_2H and between **1i** and PhC_2H additional data analysis was performed. Over the course of the $TR^{M}PS$ experiment there is a global decrease in signal intensity due to both the continual flow of sample through the cell and the fact that the background spectrum is acquired using delays between 900 and 1000 μs.[6, 9] In order to compensate for these effects, the absorbance of the peaks was divided by the root-mean-square intensity of the spectrum at each pump-probe delay and, in the case of **1i** and PhC_2H a five-point smothing algorithm applied.

2.2 Compound Numbering

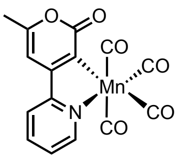

1a

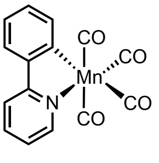

1b

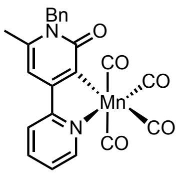

1c

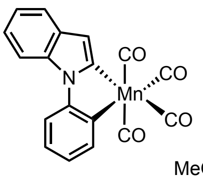

1d

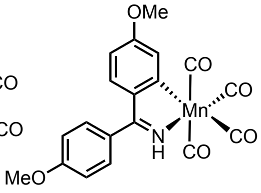

1e

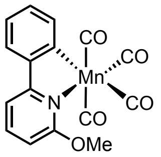

1f

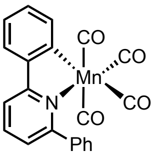

1g

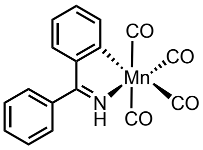

1h

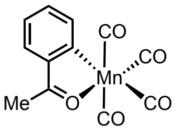

1i

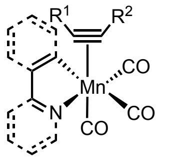

2

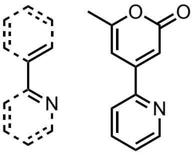

2aa R^1 = H R^2 = Ph
2aa' R^1 = Ph R^2 = H

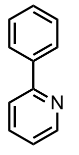

2ba R^1 = H R^2 = Ph
2ba' R^1 = Ph R^2 = H
2bb R^1 = H R^2 = $PhCO_2CH_2$
2bb' R^1 = $PhCO_2CH_2$ R^2 = H
2bc R^1 = H R^2 = Cy
2bc' R^1 = Cy R^2 = H
2bd R^1 = H R^2 = Me_2N-4-C_6H_5
2bd' R^1 = Me_2N-4-C_6H_5 R^2 = H
2be R^1 = H R^2 = MeO-4-C_6H_5
2be' R^1 = MeO-4-C_6H_5 R^2 = H
2bf R^1 = H R^2 = F-4-C_6H_5
2bf' R^1 = F-4-C_6H_5 R^2 = H
2bg R^1 = H R^2 = CF_3-4-C_6H_5
2bg' R^1 = CF_3-4-C_6H_5 R^2 = H
2bh R^1 = H R^2 = $MeCO_2$-4-C_6H_5
2bh' R^1 = $MeCO_2$-4-C_6H_5 R^2 = H
2bi R^1 = R^2 = Ph
2bj R^1 = R^2 = ^{n}Bu

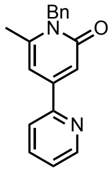

2ca R^1 = H R^2 = Ph
2ca' R^1 = Ph R^2 = H

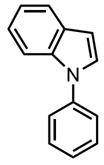

2da R^1 = H R^2 = Ph
2da' R^1 = Ph R^2 = H

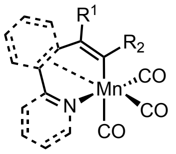

3

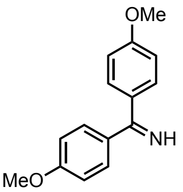

2ea R^1 = H R^2 = Ph
2ea' R^1 = Ph R^2 = H
2ej R^1 = R^2 = Ph

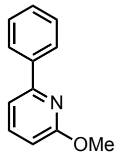

2fa R^1 = H R^2 = Ph
2fa' R^1 = Ph R^2 = H

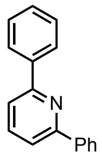

2ga R^1 = H R^2 = Ph
2ga' R^1 = Ph R^2 = H

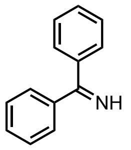

2ha R^1 = H R^2 = Ph
2ha' R^1 = Ph R^2 = H

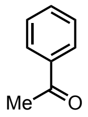

2ia R^1 = H R^2 = Ph
2ia' R^1 = Ph R^2 = H

The same letter labels are used for derivatives of the analogous compound **3**.

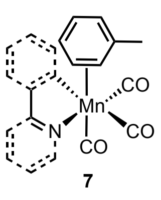

7

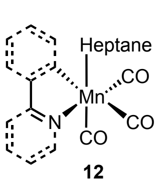

12

The same letter labels are used for derivatives of the analogous compound **1**.

Figure S13 Numbering scheme for compounds reported in the ESI

2.3 Studies in neat PhC_2H

2.3.1 TRIR study of **1e** in Neat PhC_2H

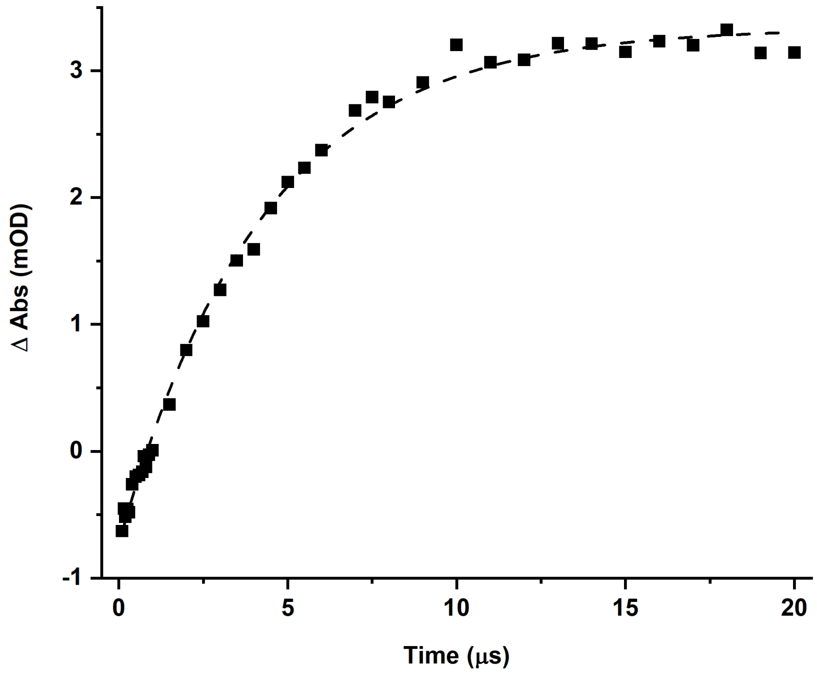

Figure S14 Kinetic plot for the reaction of **1e** with PhC_2H. The dashed line is a fit to an exponential growth function with constant k = (2.36 ± 0.15) × 10^5 s^{-1} (R^2 = 0.996) corresponding to the formation of **3ea**. The kinetic analysis was performed using data between 100 ns and 20 μs using the intensity of the peak at 1998 cm^{-1}.

2.4 Variable alkyne concentration studies

2.4.1 Collated rate constants and kinetic profiles for the reaction between **1b** and PhC_2H in toluene

The rate constants for the loss of the toluene complex **6b** and formation of **3ba** by monitoring the peak at 1942 cm^{-1} are shown in Table S1. Individual fits are shown in Figures S16 – S21.

Table S1 . Observed first order rate constants for the reaction between **1b** and PhC_2H in toluene solution

Entry	$[PhC_2H]$ mol dm^{-3}	Gain of peak at 1942 cm^{-1} k_{1obs} / s^{-1}	Loss of peak at 1942 cm^{-1} k_{2obs} / s^{-1}
1	0.0232	$(2.13 \pm 0.61) \times 10^6$	$(1.44 \pm 0.31) \times 10^5$
2	0.0465	$(2.65 \pm 1.23) \times 10^6$	$(1.42 \pm 0.32) \times 10^5$
3	0.116	$(3.09 \pm 2.67) \times 10^6$	$(1.53 \pm 0.27) \times 10^5$
4	0.232	$(8.04 \pm 2.80) \times 10^6$	$(1.47 \pm 0.25) \times 10^5$
5	0.464	$(1.61 \pm 0.47) \times 10^7$	$(1.45 \pm 0.21) \times 10^5$
6	0.921	$(3.51 \pm 1.10) \times 10^7$	$(1.67 \pm 0.28) \times 10^5$

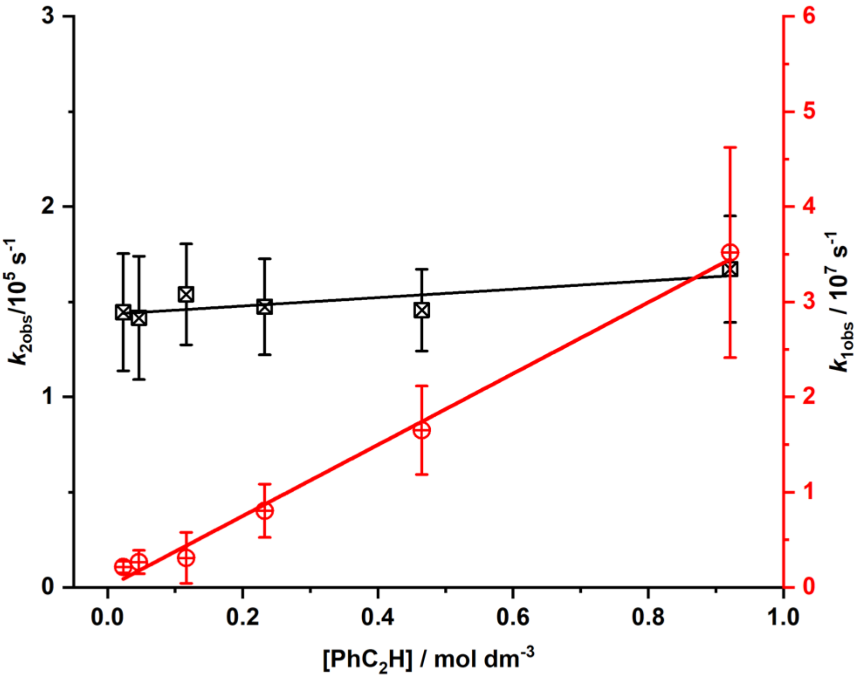

Figure S15 Pseudo first order analysis for the conversion of **6ba** to **2ba** (red circles) and **2ba** to **3ba** (black squares) using the rate constants derived from the loss of the peak at 1942 cm^{-1}. For **6ba** to **2ba**, the second order rate constant for toluene subsititution was determined from the gradient of the line as k_1 = (3.74 ± 0.16) 10^7 mol^{-1} dm^3 s^{-1} R^2 = 0.993. For **2ba** to **3ba**, the first order rate constant was determined from the intercept of the graph k_2 = (1.43 ± 0.03) 10^5 s^{-1}.

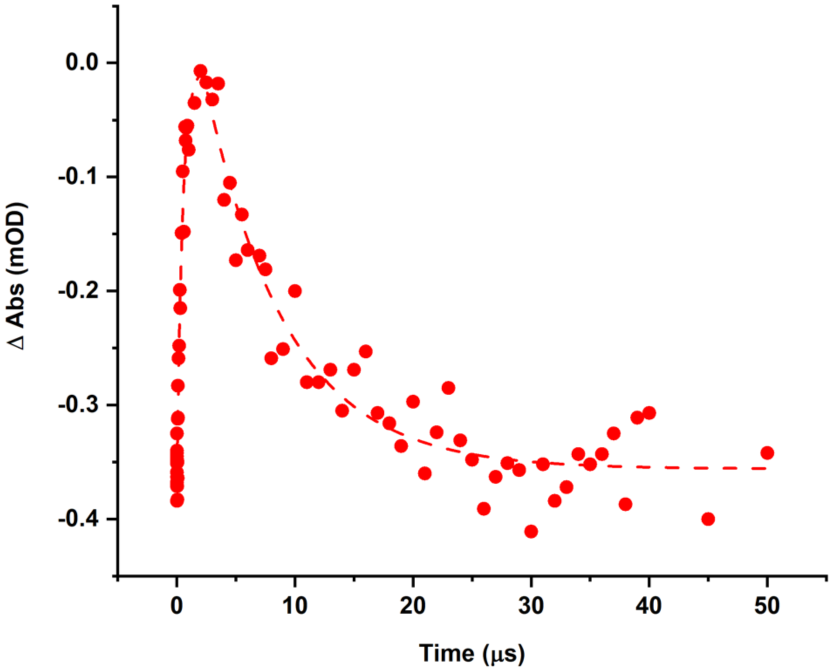

Figure S16 Kinetic plot for the reaction of **1b** in toluene with [PhC_2H] = 0.0232 mol dm^{-3}. The dashed line is a fit to an exponential growth and decay function with rate constants k_g = (2.13 ± 0.61) × 10^6 s^{-1} and k_d = (1.44 ± 0.31) × 10^5 s^{-1} for the growth and decay phases respectively (R^2 = 0.943) corresponding to the formation and loss of **2ea**. The kinetic analysis was performed using data between t = 1 ns and 50 μs using the intensity changes at 1942 cm^{-1}.

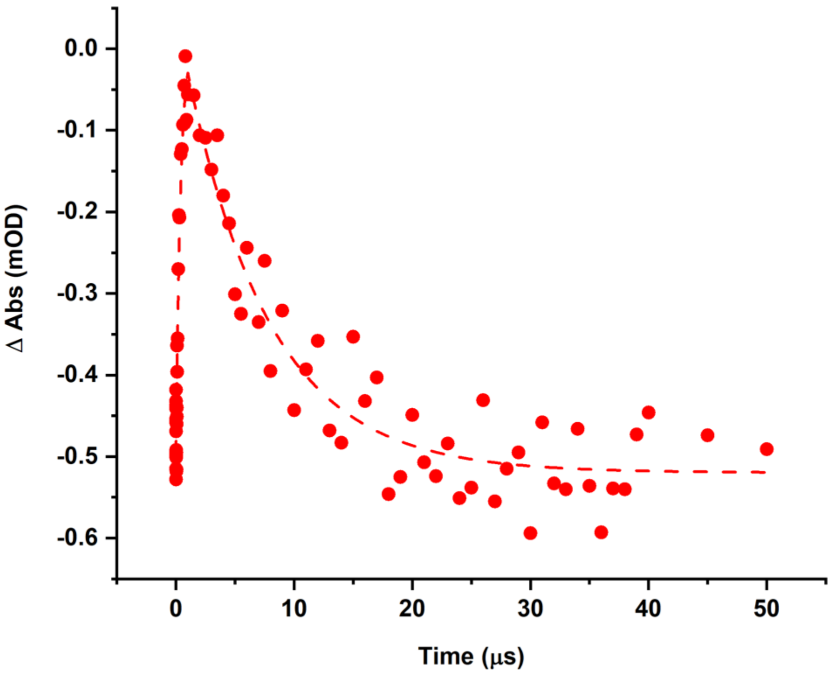

Figure S17 Kinetic plot for the reaction of **1b** in toluene with [PhC_2H] = 0.0465 mol dm^{-3}. The dashed line is a fit to an exponential growth and decay function with rate constants k_g = (2.65 ± 1.23) × 10^6 s^{-1} and k_d = (1.42 ± 0.32) × 10^5 s^{-1} for the growth and decay phases respectively (R^2 = 0.923) corresponding to the formation and loss of **2ea**. The kinetic analysis was performed using data between t = 1 ns and 50 μs using the intensity changes at 1942 cm^{-1}.

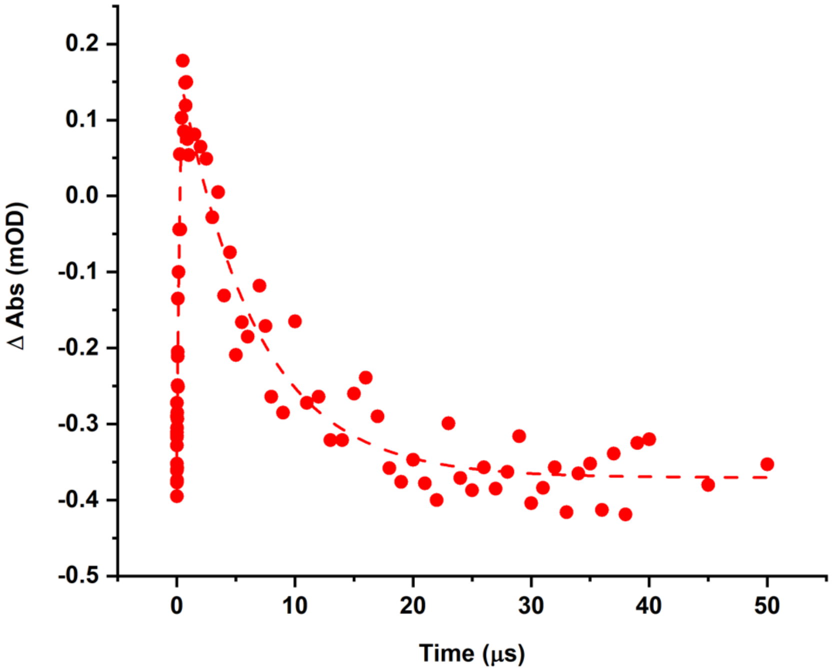

Figure S18 Kinetic plot for the reaction of **1b** in toluene with [PhC_2H] = 0.116 mol dm^{-3}. The dashed line is a fit to an exponential growth and decay function with rate constants k_g = (3.09 ± 2.67) × 10^6 s^{-1} and k_d = (1.53 ± 0.27) × 10^5 s^{-1} for the growth and decay phases respectively (R^2 = 0.942) corresponding to the formation and loss of **2ea**. The kinetic analysis was performed using data between t = 1 ns and 50 μs using the intensity changes at 1942 cm^{-1}.

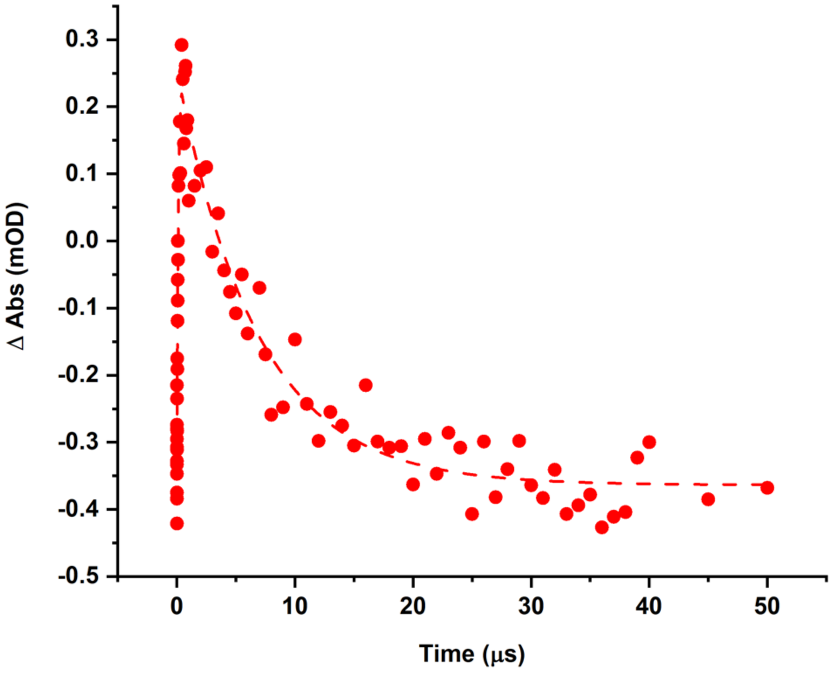

Figure S19 Kinetic plot for the reaction of **1b** in toluene with [PhC_2H] = 0.232mol dm^{-3}. The dashed line is a fit to an exponential growth and decay function with rate constants k_g = (8.04 ± 2.80) × 10^6 s^{-1} and k_d = (1.47 ± 0.25) × 10^5 s^{-1} for the growth and decay phases respectively (R^2 = 0.949) corresponding to the formation and loss of **2ea**. The kinetic analysis was performed using data between t = 1 ns and 50 μs using the intensity changes at 1942 cm^{-1}.

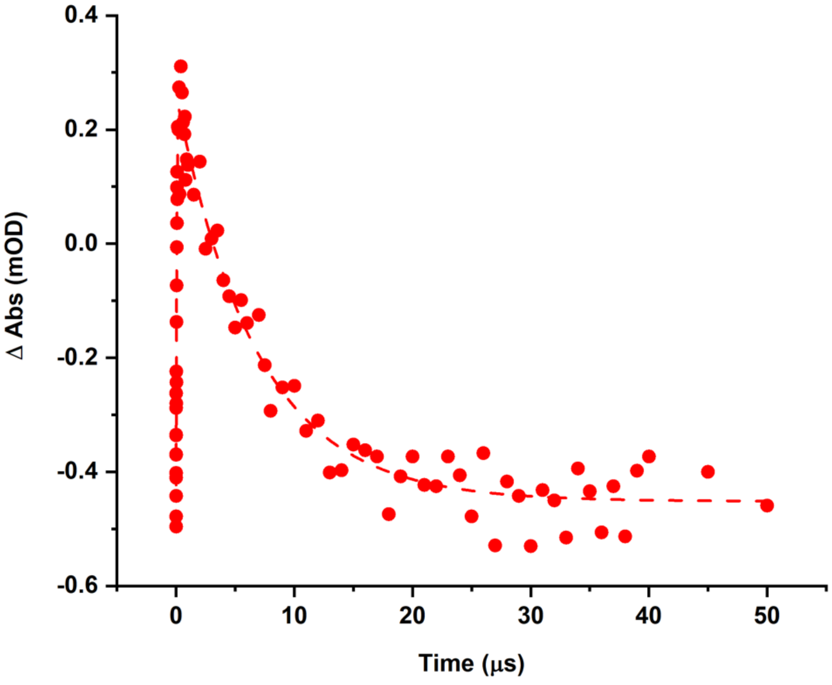

Figure S20 Kinetic plot for the reaction of **1b** in toluene with [PhC_2H] = 0.464mol dm^{-3}. The dashed line is a fit to an exponential growth and decay function with rate constants k_g = (1.61 ± 0.47) × 10^7 s^{-1} and k_d = (1.45 ± 0.21) × 10^5 s^{-1} for the growth and decay phases respectively (R^2 = 0.964) corresponding to the formation and loss of **2ea**. The kinetic analysis was performed using data between t = 1 ns and 50 μs using the intensity changes at 1942 cm^{-1}.

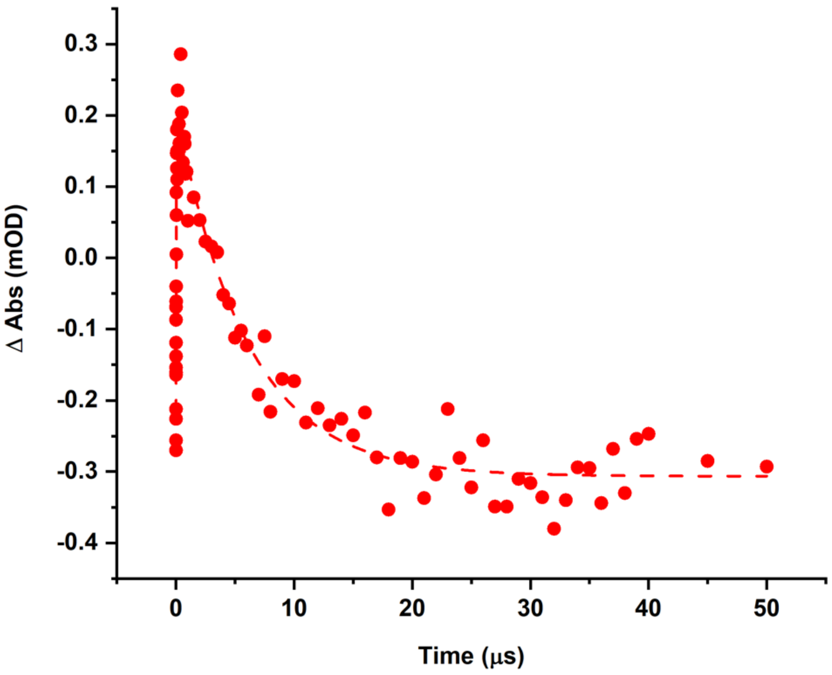

Figure S21 Kinetic plot for the reaction of **1b** in toluene with [PhC_2H] 0.921 mol dm^{-3}. The dashed line is a fit to an exponential growth and decay function with rate constants k_g = (3.51 ± 1.10) × 10^7 s^{-1} and k_d = (1.67 ± 0.28) × 10^5 s^{-1} for the growth and decay phases respectively (R^2 = 0.959) corresponding to the formation and loss of **2ea**. The kinetic analysis was performed using data between t = 1 ns and 50 μs using the intensity changes at 1942 cm^{-1}.

2.4.2 Collated rate constants and kinetic profiles for the reaction between **1e** and PhC_2H in toluene

The rate constants for the loss of the toluene complex **6e** and formation of **3ea** by monitoring the peak at 2008 cm^{-1} are shown in Table S2. Individual fits are shown in Figures S24 – S28.

Table S2 . Observed first order rate constants for the reaction between **1e** and PhC_2H in toluene solution

Entry	$[PhC_2H]$ mol dm^{-3}	Gain of peak at 2008 cm^{-1} k_{1obs} / s^{-1}	Loss of peak at 2008 cm^{-1} k_{2obs} / s^{-1}
1	0.023	$(2.85 \pm 0.46) \times 10^6$	$(2.35 \pm 0.18) \times 10^5$
2	0.046	$(4.56 \pm 0.69) \times 10^6$	$(2.30 \pm 0.68) \times 10^5$
3	0.116	$(7.81 \pm 2.24) \times 10^6$	$(1.87 \pm 0.23) \times 10^5$
4	0.232	$(1.68 \pm 0.22) \times 10^7$	$(2.36 \pm 0.15) \times 10^5$
5	0.464	$(3.43 \pm 0.45) \times 10^7$	$(2.19 \pm 0.17) \times 10^5$

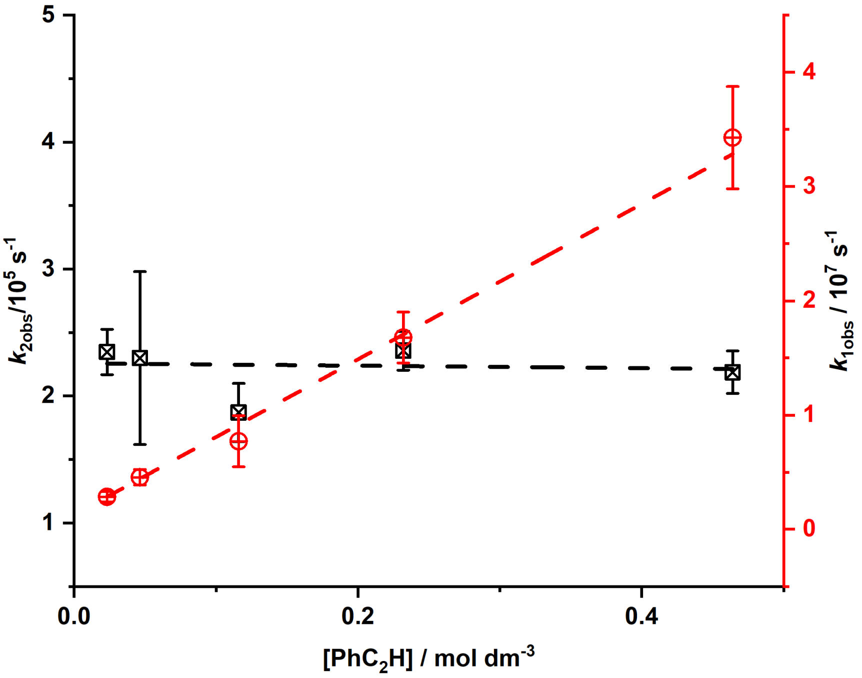

Figure S22 Pseudo first order analysis for the conversion of **6e** to **2ea** (red circles) and **2ea** to **3ea** (black squares) using the rate constants derived from the loss of the peak at 2008 cm^{-1}. For **6e** to **2ea**, the second order rate constant for toluene substitution was determined from the gradient of the line as $k_1 = (7.17 \pm 0.26)$ 10^7 mol^{-1} dm^3 s^{-1} $R^2 = 0.995$. For **2ea** to **3ea**, the first order rate constant was determined from the intercept of the graph $k_2 = (2.25 \pm 0.16)$ 10^5 s^{-1}.

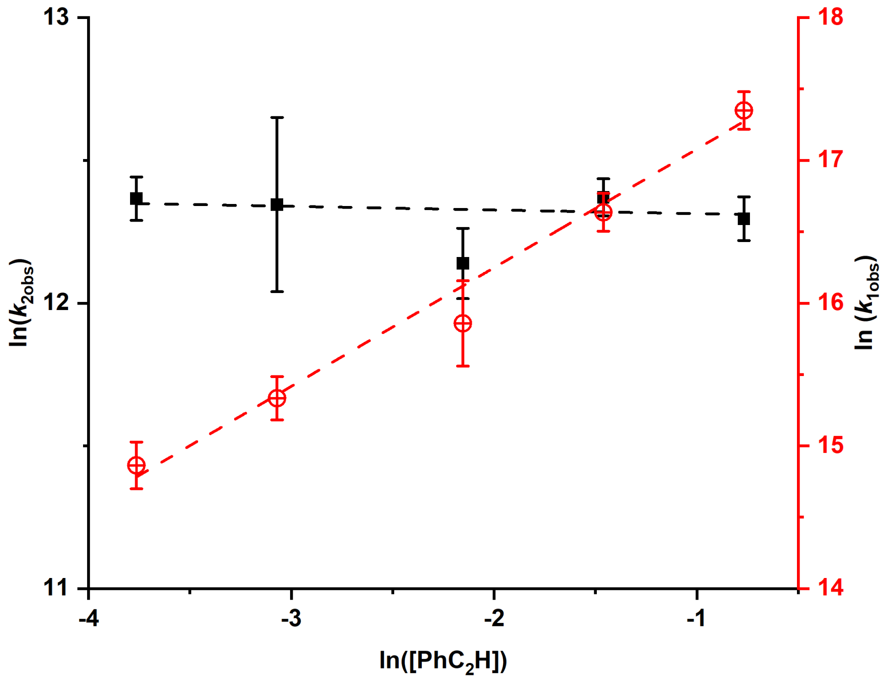

Figure S23 Plots of ln(k_{1obs}) and ln(k_{2obs}) versus ln([PhC_2H]) to determine the order for the conversion of **6e** to **2ea** (red circles) and **2ea** to **3ea** (black squares) using the rate constants derived from the loss of the peak at 2008 cm^{-1}. For **6e** to **2ea**, the gradient of the line is (0.83 ± 0.04) R^2 = 0.989 and **2ea** to **3ea** (-0.01 ± 0.03).

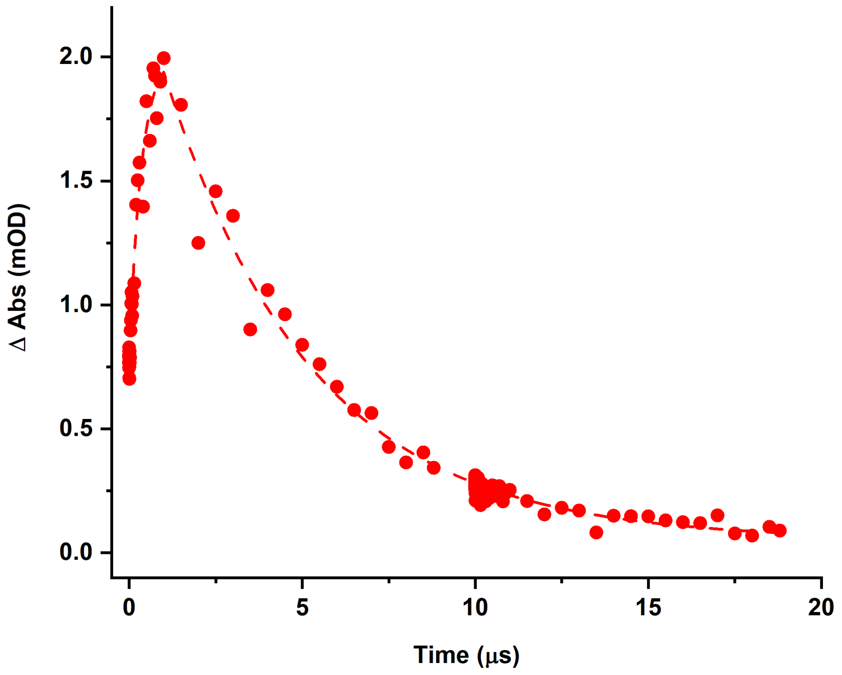

Figure S24 Kinetic plot for the reaction of **1e** in toluene with [PhC_2H] 0.023 mol dm^{-3}. The dashed line is a fit to an exponential growth and decay function with rate constants k_g = (2.85 ± 0.46) × 10^6 s^{-1} and k_d = (2.35 ± 0.18) × 10^5 s^{-1} for the growth and decay phases respectively (R^2 = 0.986) corresponding to the formation and loss of **2ea**. The kinetic analysis was performed using data between t = 0.1 ns and 18.8 μs using the intensity change at 2008 cm^{-1}.

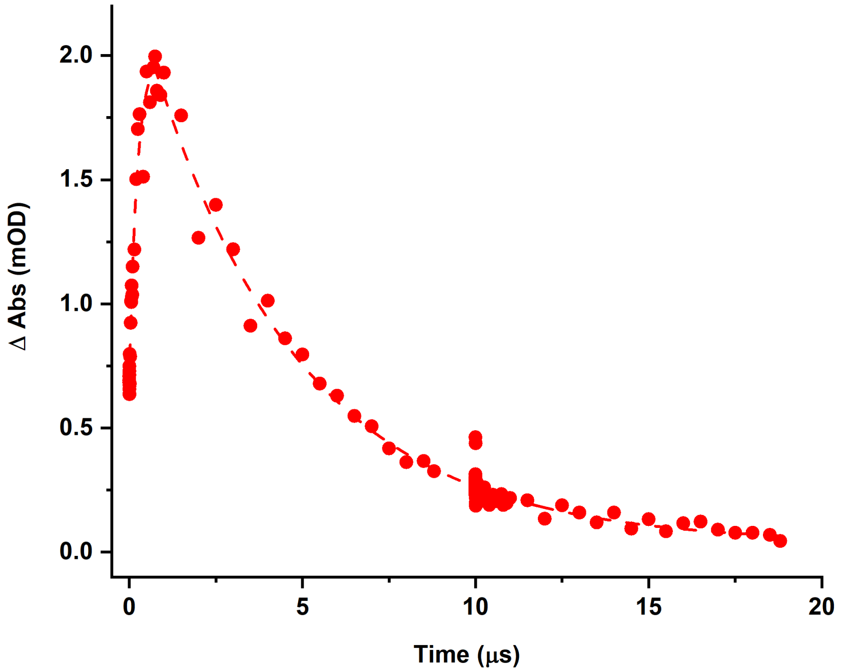

Figure S25 Kinetic plot for the reaction of **1e** in toluene with [PhC_2H] 0.046 mol dm^{-3}. The dashed line is a fit to an exponential growth and decay function with rate constants k_g = (4.65 ± 0.69) × 10^6 s^{-1} and k_d = (2.30 ± 0.68) × 10^5 s^{-1} for the growth and decay phases respectively (R^2 = 0.988) corresponding to the formation and loss of **2ea**. The kinetic analysis was performed using data between t = 0.1 ns and 18.8 μs using the intensity change at 2008 cm^{-1}

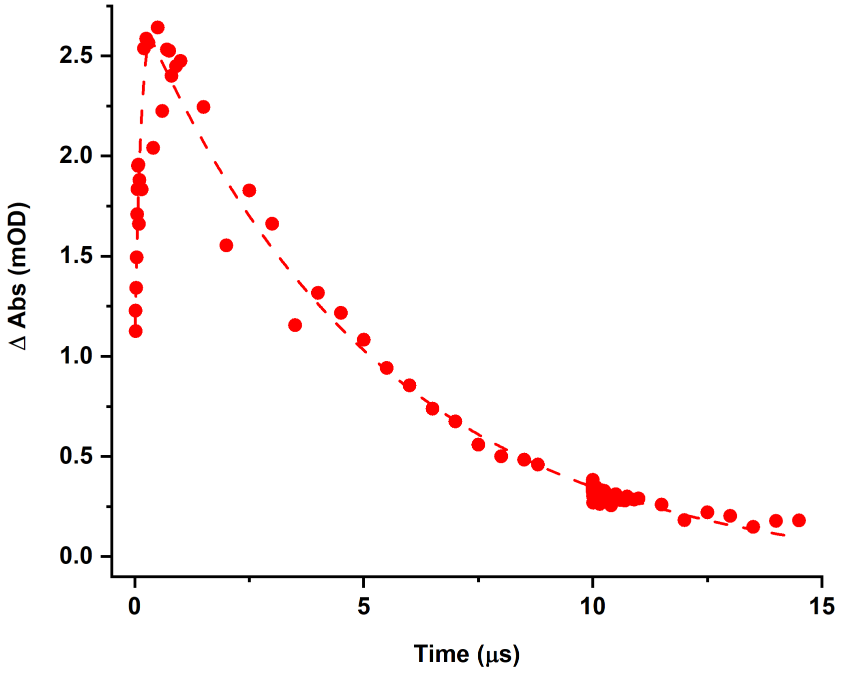

Figure S26 Kinetic plot for the reaction of **1e** in toluene with [PhC_2H] 0.116 mol dm^{-3}. The dashed line is a fit to an exponential growth and decay function with rate constants k_g = (7.81 ± 2.24) × 10^6 s^{-1} and k_d = (1.87 ± 0.23) × 10^5 s^{-1} for the growth and decay phases respectively (R^2 = 0.982) corresponding to the formation and loss of **2ea**. The kinetic analysis was performed using data between t = 10 ns and 14.5 μs using the intensity change at 2008 cm^{-1}.

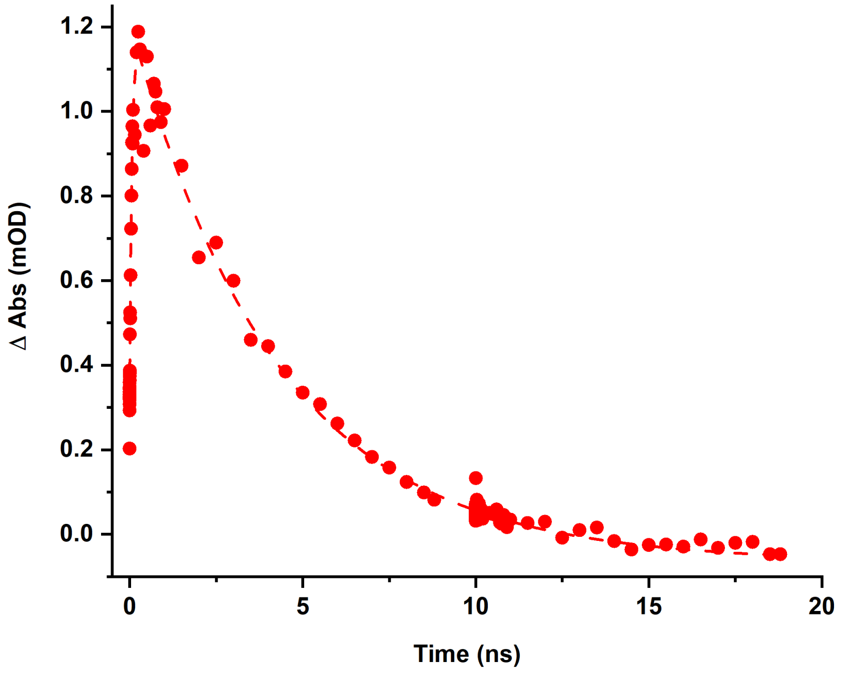

Figure S27 Kinetic plot for the reaction of **1e** in toluene with [PhC_2H] 0.232 mol dm^{-3}. The dashed line is a fit to an exponential growth and decay function with rate constants k_g = (1.68 ± 0.22) × 10^7 s^{-1} and k_d = (2.36 ± 0.15) × 10^5 s^{-1} for the growth and decay phases respectively (R^2 = 0.992) corresponding to the formation and loss of **2ea**. The kinetic analysis was performed using data between t = 100 ps and 18.8 μs using the intensity change at 2008 cm^{-1}.

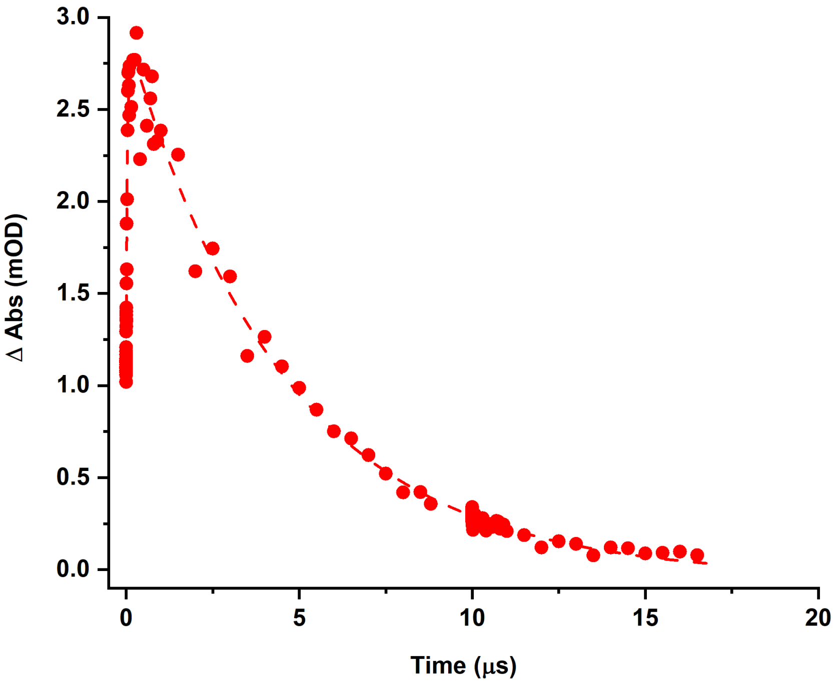

Figure S28 Kinetic plot for the reaction of **1e** in toluene with [PhC_2H] 0.464 mol dm^{-3}. The dashed line is a fit to an exponential growth and decay function with rate constants k_g = (3.32 ± 0.45) × 10^7 s^{-1} and k_d = (2.19 ± 0.17) × 10^5 s^{-1} for the growth and decay phases respectively (R^2 = 0.991) corresponding to the formation and loss of **2ea**. The kinetic analysis was performed using data between t = 0.1 ns and 17.0 μs using the intensity change at 2008 cm^{-1}.

2.4.3 Collated rate constants and kinetic profiles for the reaction between **1e** and PhC_2Ph in heptane

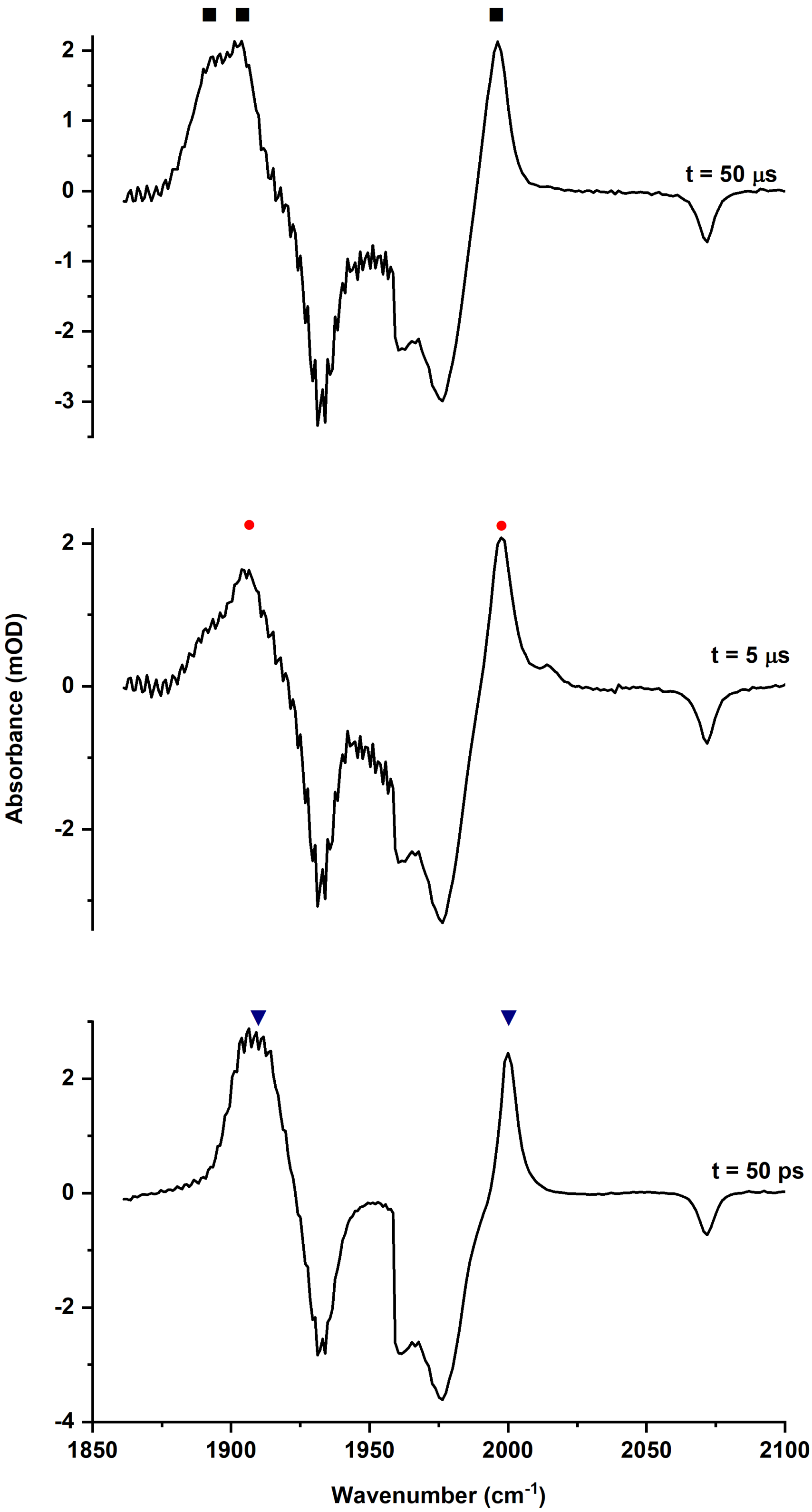

Figure S29 TRIR data for the reaction between **1e** and PhC_2Ph in heptane solution. TRIR data in the metal carbonyl region, the y-axis is change in absorbance, the negative peaks correspond to the bleach of the bands for **1b**, the positive bands show the growth and change of intermediates. Spectra with pump-probe delays of 50 ps, 5 μs, 50 μs are shown. Peaks labels are assigned to **12e** (blue triangles), **2ei** (red circles) and **3ei** (black squares).

The rate constants for the loss of the heptane complex **12e** and formation of **3ei** by monitoring the peak at 2008 cm^{-1} are shown in Table S3. Individual fits are shown in Figures S30 – S39.

Table S3 . Observed first order rate constants for the reaction between **1e** and PhC_2H in toluene solution

Entry	$[PhC_2Ph]$ mol dm^{-3}	Loss of peak at 2008 cm^{-1} k_{obs} / s^{-1}	Gain of **3ei** 1993 cm^{-1} k_{obs} / s^{-1}
1	0.0230	$(9.12 \pm 1.36) \times 10^7$	N. D.
2	0.0463	$(1.54 \pm 0.32) \times 10^8$	N. D.
3	0.116	$(3.60 \pm 0.52) \times 10^8$	$(1.79 \pm 0.20) \times 10^4$
4	0.231	$(7.28 \pm 0.67) \times 10^8$	$(2.36 \pm 0.15) \times 10^4$
5	0.464	$(1.20 \pm 0.22) \times 10^9$	$(2.99 \pm 0.21) \times 10^4$

The quality of the data recorded at alkyne concentratons of 0.023 and 0.0463 mol dm^{-3} was not sufficient to determine the rate constants for the formation of **3ei**. The data for the three other concentrations are shown in Figures S30 and S31 for completeness, however, linear fits to these data have not been performed.

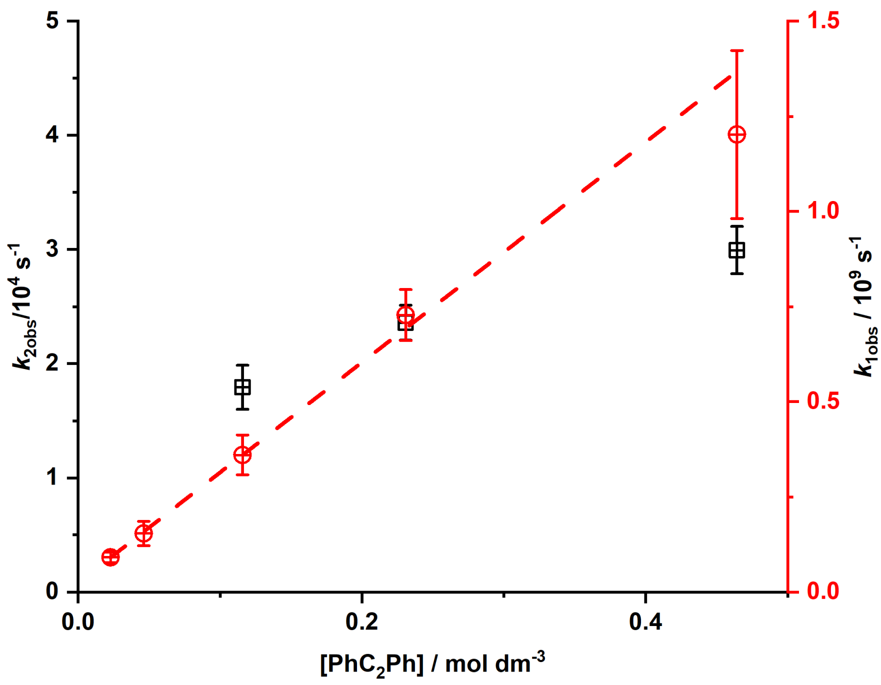

Figure S30 Pseudo first order analysis for the conversion of **12e** to **2ej** (red circles) and **2ej** to **3ej** (black squares) using the rate constants derived from the loss of the peaks at 2008 and 1992 cm^{-1} respectively. For **12e** to **2ej**, the second order rate constant for heptane substitution was determined from the gradient of the line as k_1 = (2.89 ± 0.14) 10^9 mol^{-1} dm^3 s^{-1} R^2 = 0.991. For **2ej** to **3ej**, the first order rate constant was determined by taking the mean of the three data points k_2 = (2.38 ± 0.11) 10^4 s^{-1}.

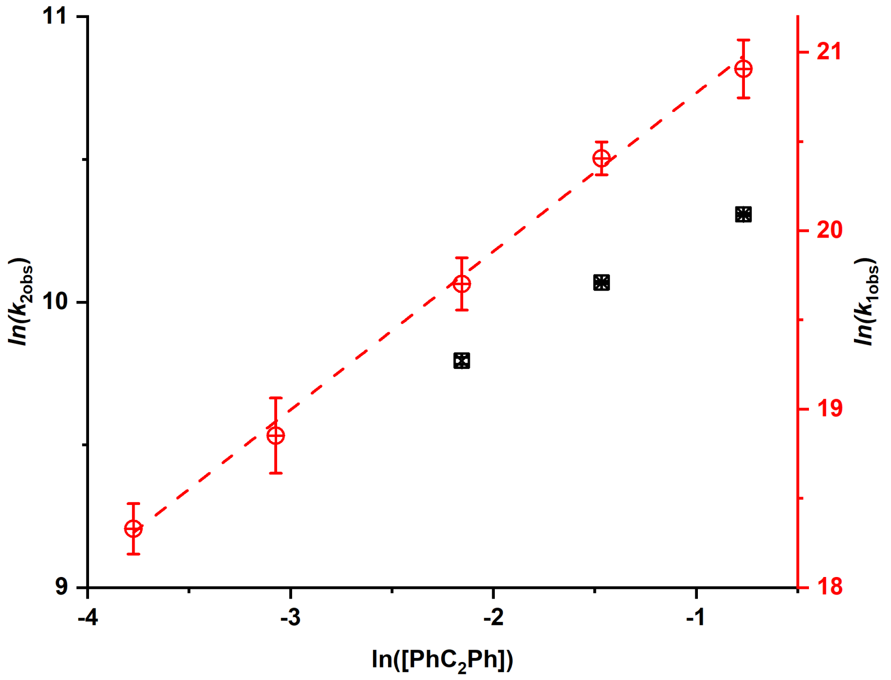

Figure S31 Plots of ln(k_{1obs}) and ln(k_{2obs}) versus ln([PhC_2Ph]) to determine the order for the conversion of **12e** to **2ej** (red circles) and **2ej** to **3ej** (black squares) using the rate constants derived from the loss of the peak at 2008 cm^{-1} and 1993 cm^{-1} respectively. For **12e** to **2ej**, the gradient of the line is (0.89 ± 0.03) R^2 = 0.996.

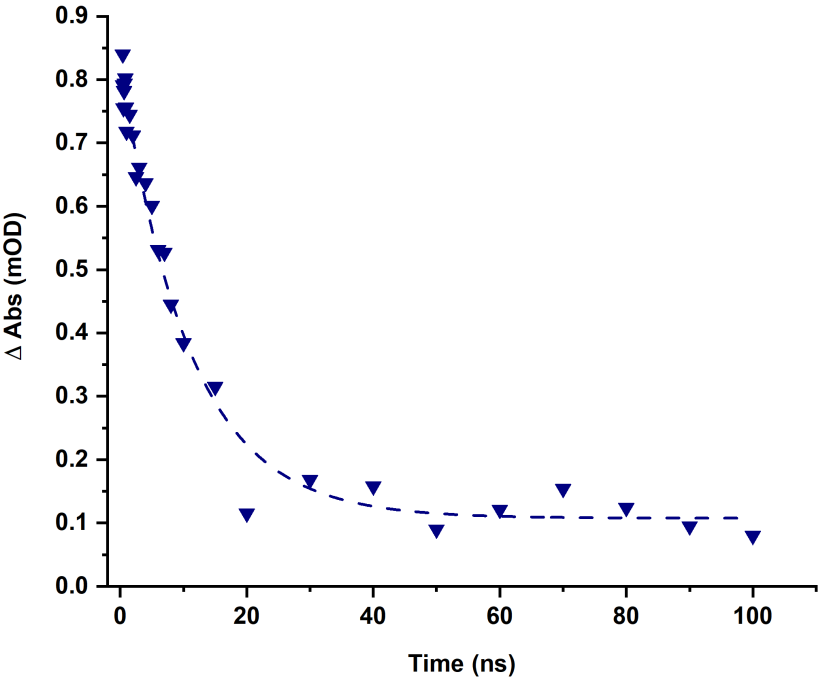

Figure S32 Kinetic plot for the reaction of **1e** in heptane with [PhC_2Ph] 0.0230 mol dm^{-3}. The dashed line is a fit to an exponential loss function with rate constant k = (9.12 ± 1.36) × 10^{7} s^{-1} (R^2 = 0.985) corresponding to the loss of **12e**. The kinetic analysis was performed using data between t = 40 ps and 100 ns using the intensity change at 2008 cm^{-1}.

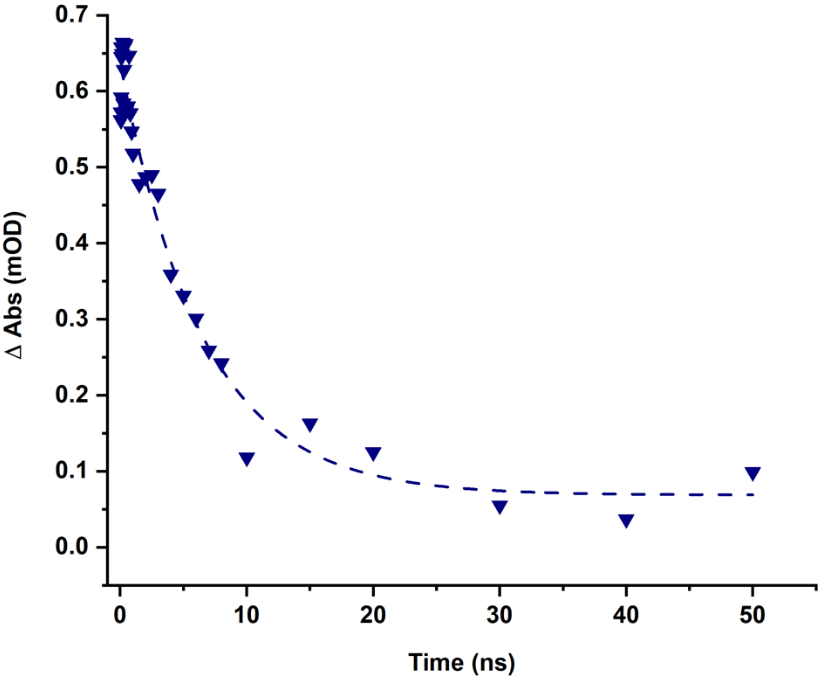

Figure S33 Kinetic plot for the reaction of **1e** in heptane with [PhC_2Ph] 0.0463 mol dm^{-3}. The dashed line is a fit to an exponential loss function with rate constant k = (1.54 ± 0.32) × 10^{8} s^{-1} (R^2 = 0.966) corresponding to the loss of **12e**. The kinetic analysis was performed using data between t = 60 ps and 50 ns using the intensity change at 2008 cm^{-1}.

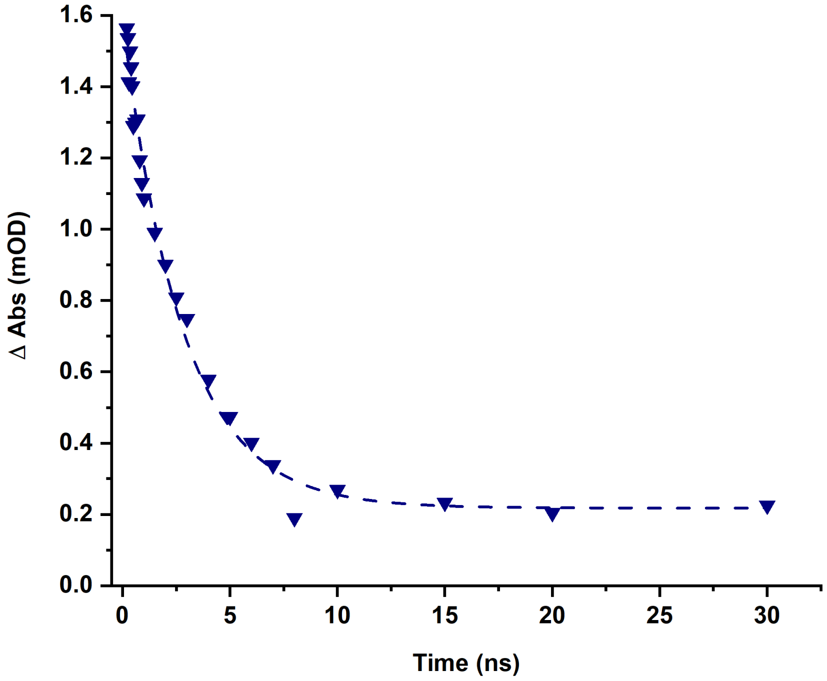

Figure S34 Kinetic plot for the reaction of **1e** in heptane with [PhC_2Ph] 0.116 mol dm^{-3}. The dashed line is a fit to an exponential loss function with rate constant k = (3.60 ± 0.52) × 10^8 s^{-1} (R^2 = 0.988) corresponding to the loss of **12e**. The kinetic analysis was performed using data between t = 200 ps and 30 ns using the intensity change at 2008 cm^{-1}.

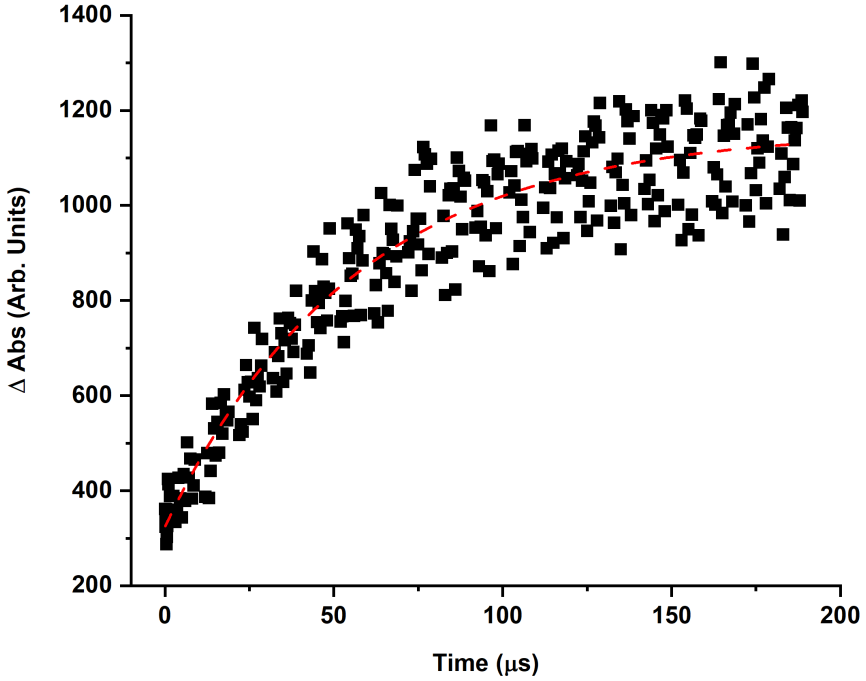

Figure S35 Kinetic plot for the reaction of **1e** in heptane with [PhC_2Ph] 0.116 mol dm^{-3}. The dashed line is a fit to an exponential growth function with rate constant k = (1.79 ± 0.20) × 10^4 s^{-1} (R^2 = 0.906) corresponding to the formation of **3ej**. The kinetic analysis was performed using data between t = 100 ns and 188.8 μs using the intensity change at 1993 cm^{-1}. The data artefacts arising at each probe pulse in the TR^{M}PS experiment have been removed.

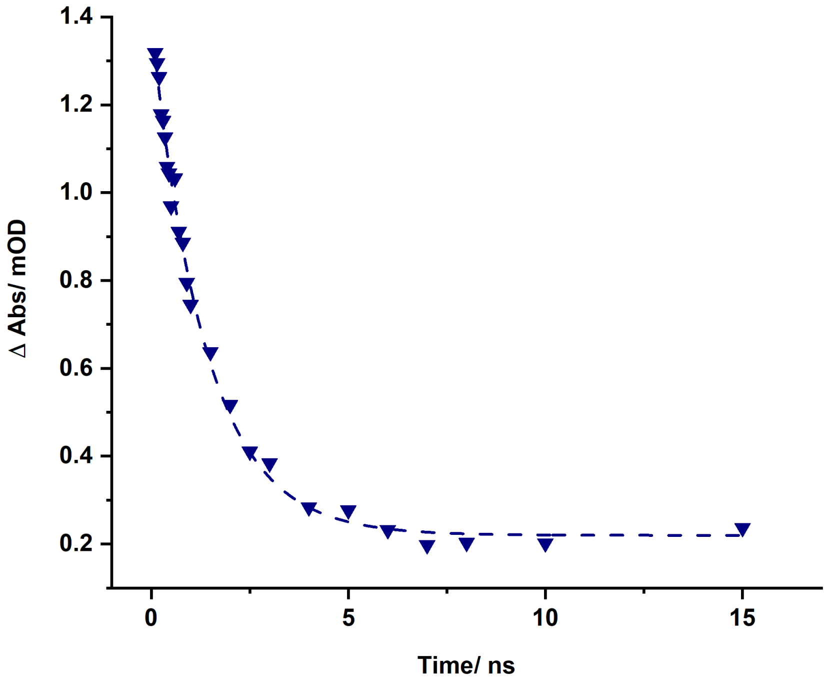

Figure S36 Kinetic plot for the reaction of **1e** in heptane with [PhC_2Ph] 0.231 mol dm^{-3}. The dashed line is a fit to an exponential loss function with rate constant k = (7.28 ± 0.67) × 10^{8} s^{-1} (R^2 = 0.995) corresponding to the loss of **12e**. The kinetic analysis was performed using data between t = 100 ps and 15 ns using the intensity change at 2008 cm^{-1}.

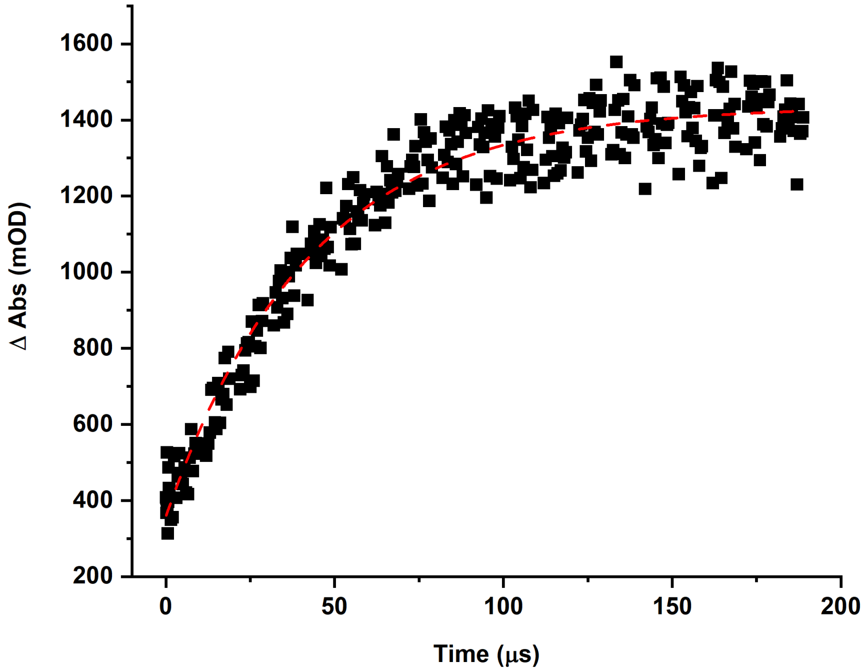

Figure S37 Kinetic plot for the reaction of **1e** in heptane with [PhC_2Ph] 0.231 mol dm^{-3}. The dashed line is a fit to an exponential growth function with rate constant k = (2.36 ± 0.15) × 10^{4} s^{-1} (R^2 = 0.955) corresponding to the formation of **3ej**. The kinetic analysis was performed using data between t = 100 ns and 188.8 μs using the intensity change at 1993 cm^{-1}. The data artefacts arising at each probe pulse in the TR^{M}PS experiment have been removed.

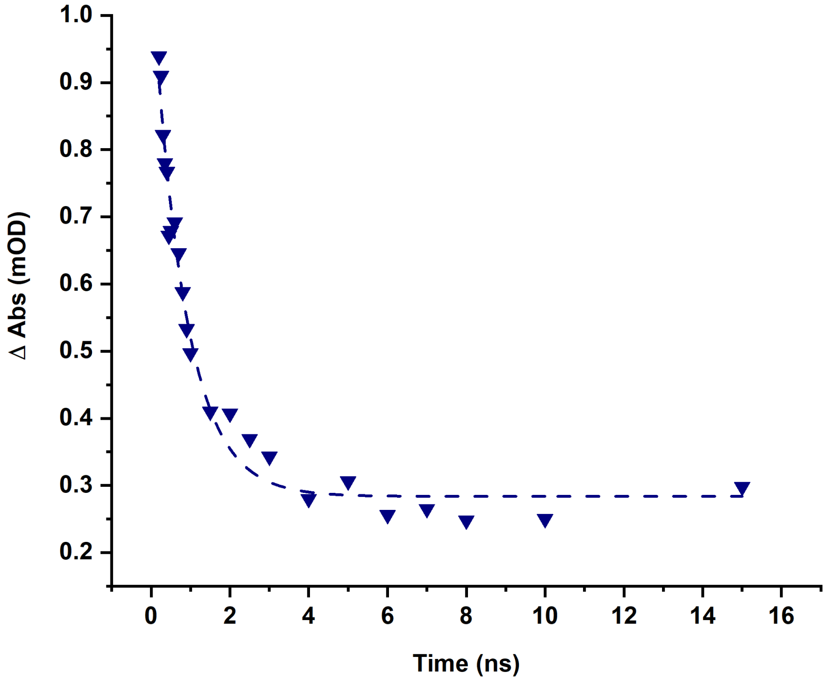

Figure S38 Kinetic plot for the reaction of **1e** in heptane with [PhC_2Ph] 0.464 mol dm^{-3}. The dashed line is a fit to an exponential loss function with rate constant k = (1.20 ± 0.22) × 10^{9} s^{-1} (R^2 = 0.979) corresponding to the loss of **12e**. The kinetic analysis was performed using data between t = 200 ps and 15 ns using the intensity change at 2008 cm^{-1}.

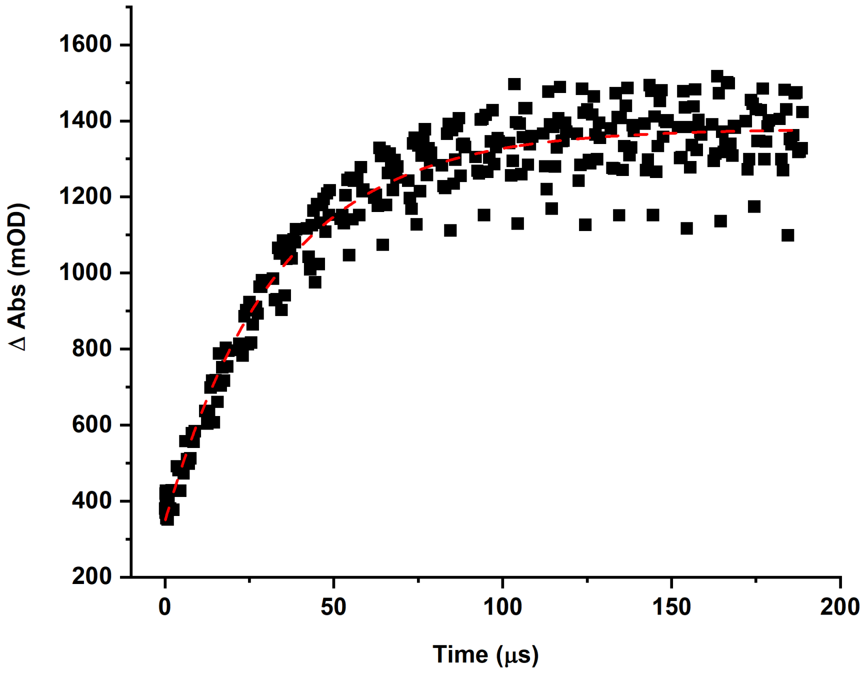

Figure S39 Kinetic plot for the reaction of **1e** in heptane with [PhC_2Ph] 0.464 mol dm^{-3}. The dashed line is a fit to an exponential growth function with rate constant k = (2.99 ± 0.21) × 10^{4} s^{-1} (R^2 = 0.942) corresponding to the formation of **3ej**. The kinetic analysis was performed using data between t = 100 ns and 188.8 μs using the intensity change at 1993 cm^{-1}. The data artefacts arising at each probe pulse in the TR^{M}PS experiment have been removed.

2.5 Studies at constant concentration of alkyne in toluene solution

2.5.1 TRIR study of **1b** and CyC_2H in toluene solution

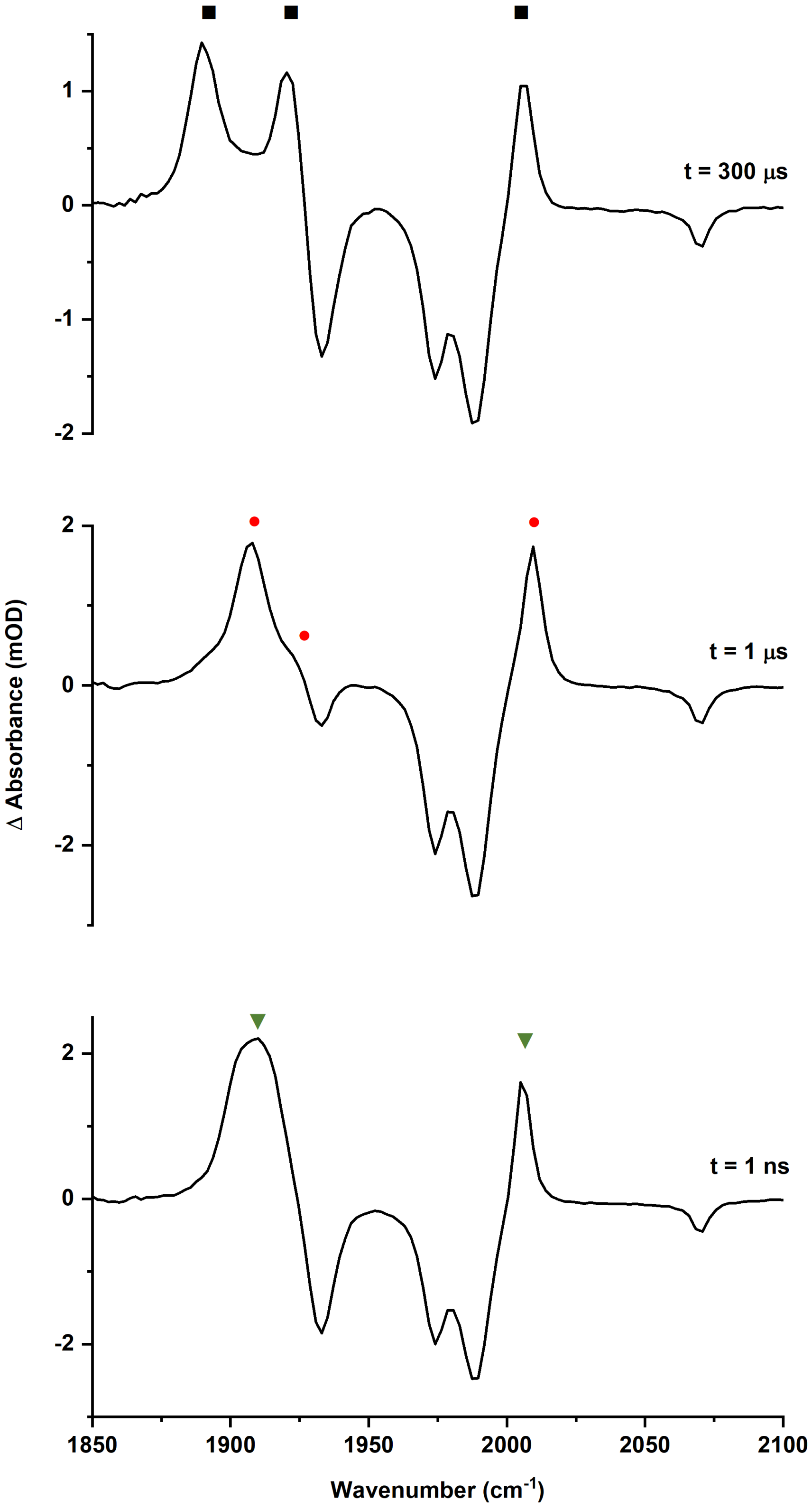

Figure S40 TRIR data for the reaction between **1b** and CyC_2H in toluene solution. TRIR data in the metal carbonyl region, the y-axis is change in absorbance, the negative peaks correspond to the bleach of the bands for **1b**, the positive bands show the growth and change of intermediates. Spectra with pump-probe delays of 1 ns, 1 μs, 300 μs are shown. Peaks labels are assigned to **6b** (green triangles), **2bc** (red circles) and **3bc** (black squares).

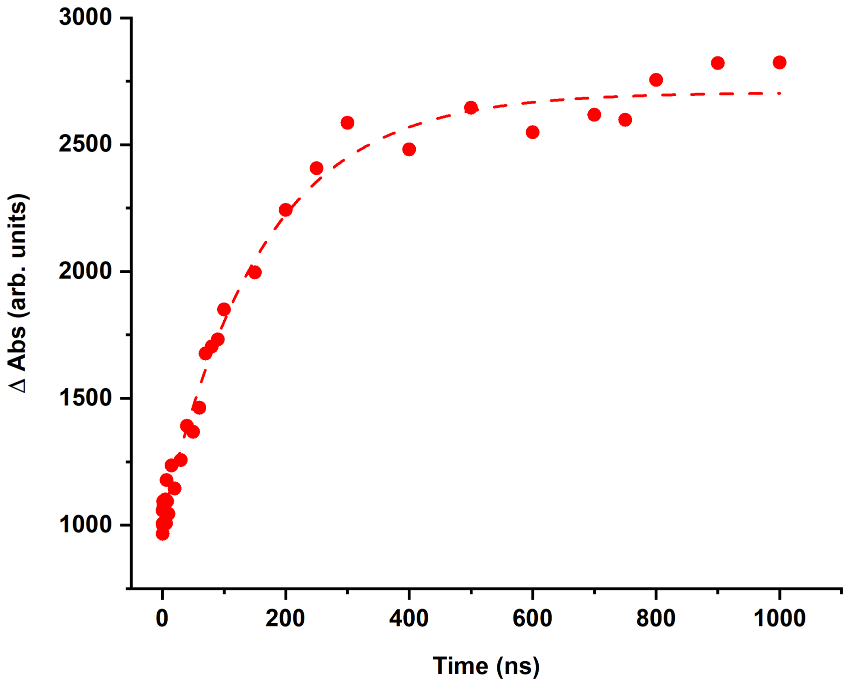

Figure S41 Kinetic plot for the reaction of **1b** in toluene with CyC_2H. The dashed line is a fit to an exponential growth function with rate constant k = (6.30 ± 0.71) × 10^6 s^{-1} (R^2 = 0.989) corresponding to the formation of **2bc**. The kinetic analysis was performed using data between t = 500 ps and 1 μs using the intensity changes at 2010 cm^{-1}. The data have been scaled by the root-mean-square intensity of the spectrum at each pump-probe delay.

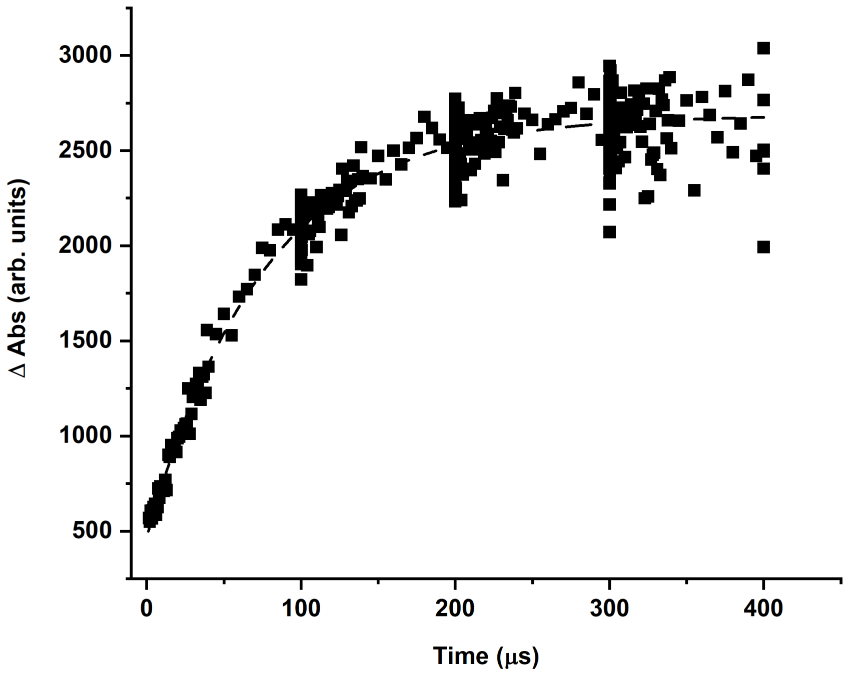

Figure S42 Kinetic plot for the reaction of **1b** in toluene with CyC_2H. The dashed line is a fit to an exponential growth function with rate constant k = (1.31 ± 0.06) × 10^4 s^{-1} (R^2 = 0.944) corresponding to the formation of **3bc**. The kinetic analysis was performed using data between t = 1.5 μs and 400 μs using the intensity changes at 1890 cm^{-1}. The data have been scaled by the root-mean-square intensity of the spectrum at each pump-probe delay.

2.5.2 TRIR study of **1b** and PhC_2Ph in toluene solution

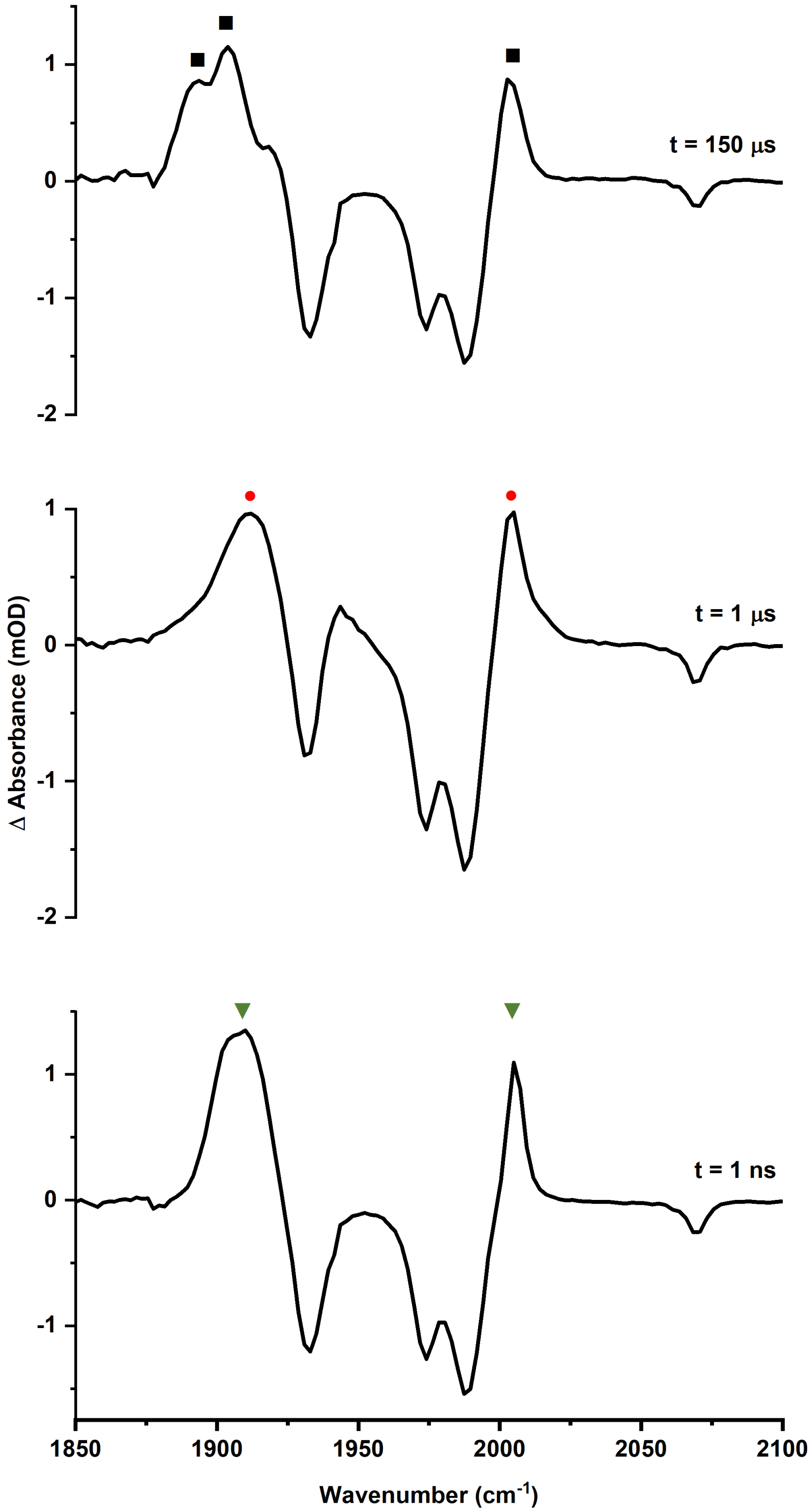

Figure S43 TRIR data for the reaction between **1b** and PhC_2Ph in toluene solution. TRIR data in the metal carbonyl region, the y-axis is change in absorbance, the negative peaks correspond to the bleach of the bands for **1b**, the positive bands show the growth and change of intermediates. Spectra with pump-probe delays of 1 ns, 1 µs, 150 µs are shown. Peaks labels are assigned to **6b** (green triangles), **2bi** (red circles) and **3bi** (black squares).

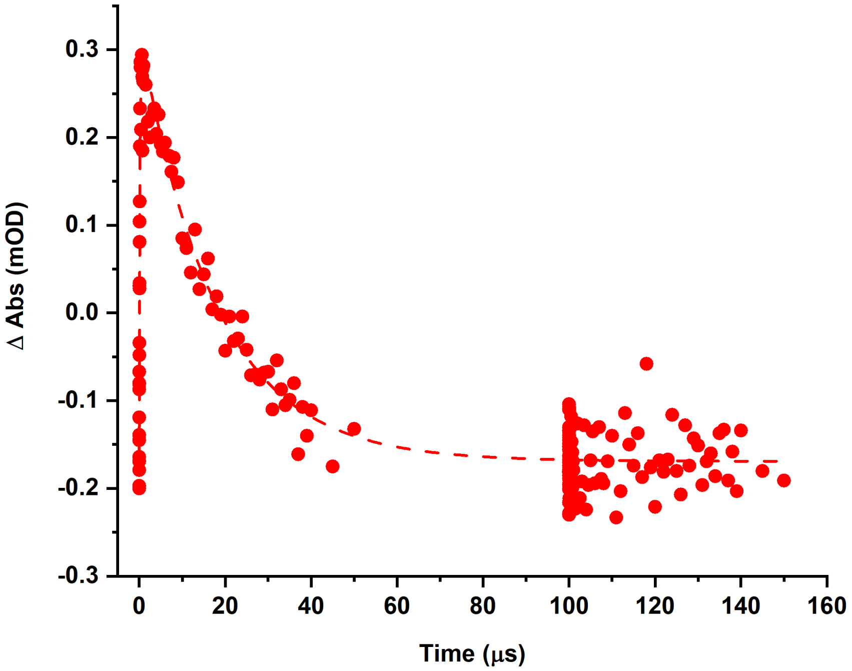

Figure S44 Kinetic plot for the reaction of **1b** in toluene with PhC_2Ph. The dashed line is a fit to an exponential growth and decay function with rate constants k_g = (11.95 ± 2.08) × 10^6 s^{-1} and k_d = (0.57 ± 0.06) × 10^5 s^{-1} for the growth and decay phases respectively (R^2 = 0.964) corresponding to the formation and loss of **2bi**. The kinetic analysis was performed using data between t = 1 ns and 150 μs using the intensity changes at 1944 cm^{-1}.

2.5.3 TRIR study of **1b** and $^{n}BuC_2{}^{n}Bu$ in toluene solution

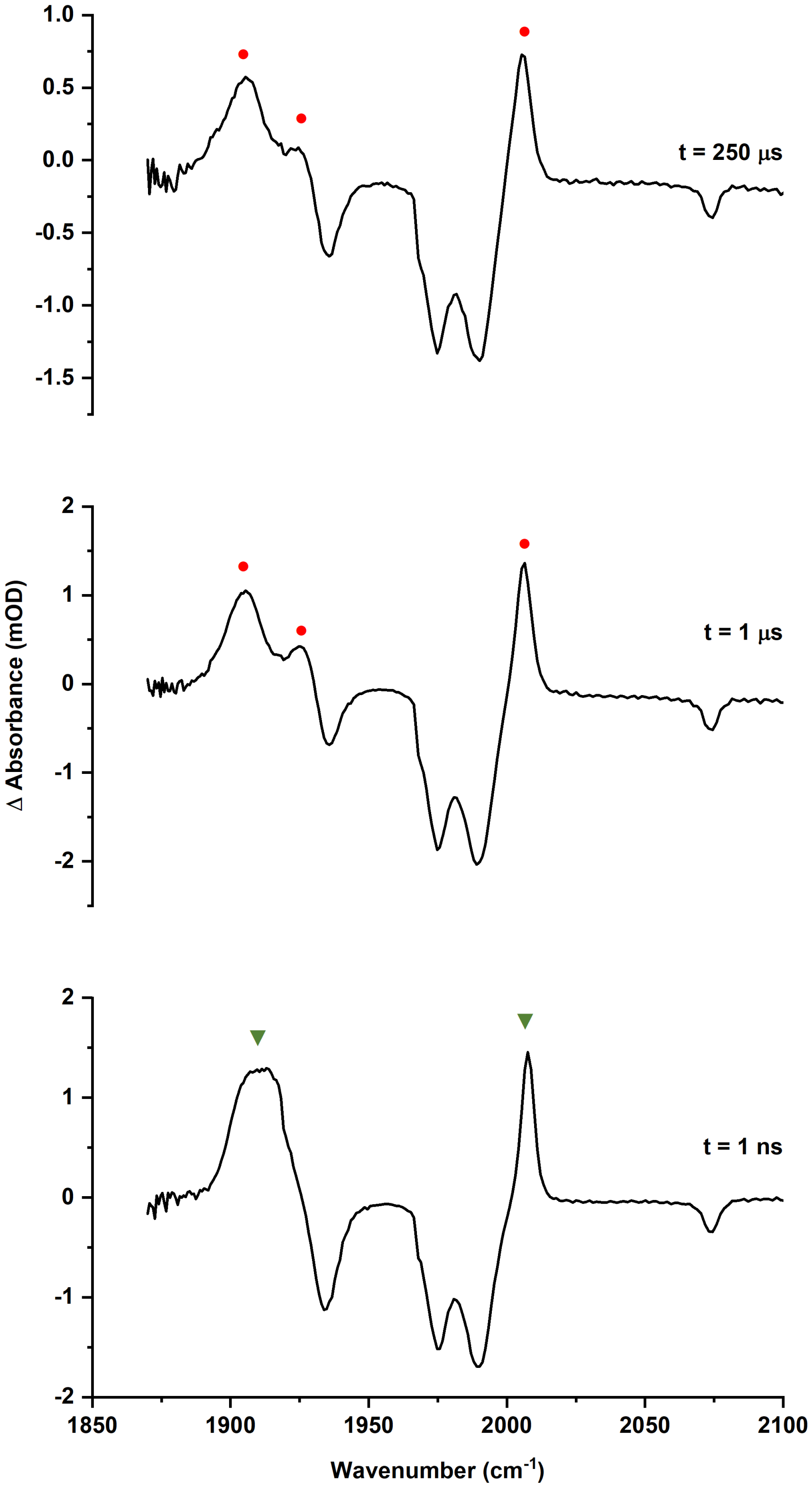

Figure S45 TRIR data for the reaction between **1b** and $^{n}BuPhC_2{}^{n}Bu$ in toluene solution. TRIR data in the metal carbonyl region, the y-axis is change in absorbance, the negative peaks correspond to the bleach of the bands for **1b**, the positive bands show the growth and change of intermediates. Spectra with pump-probe delays of 1 ns, 1 μs, 250 μs are shown. Peaks labels are assigned to **6b** (green triangles), **2bj** (red circles) and **3bj** (black squares).

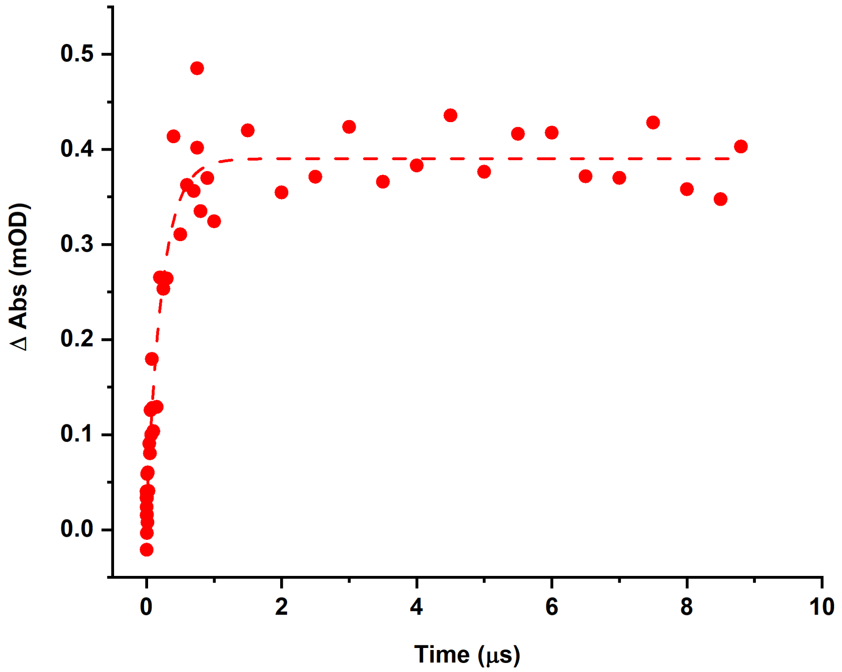

Figure S46 Kinetic plot for the reaction of **1b** in toluene solution with $^nBuPhC_2{}^nBu$. The dashed line is a fit to an exponential decay function with rate constant k = (4.36 ± 1.00) × 10^6 s^{-1} (R^2 = 0.953) corresponding to the formation of **2bj**. The kinetic analysis was performed using data between t = 1 ns and 8.8 μs using the intensity changes at 1925 cm^{-1}.

2.5.4 TRIR study of **1b** and CF_3-4-$C_6H_4C_2H$ in toluene solution

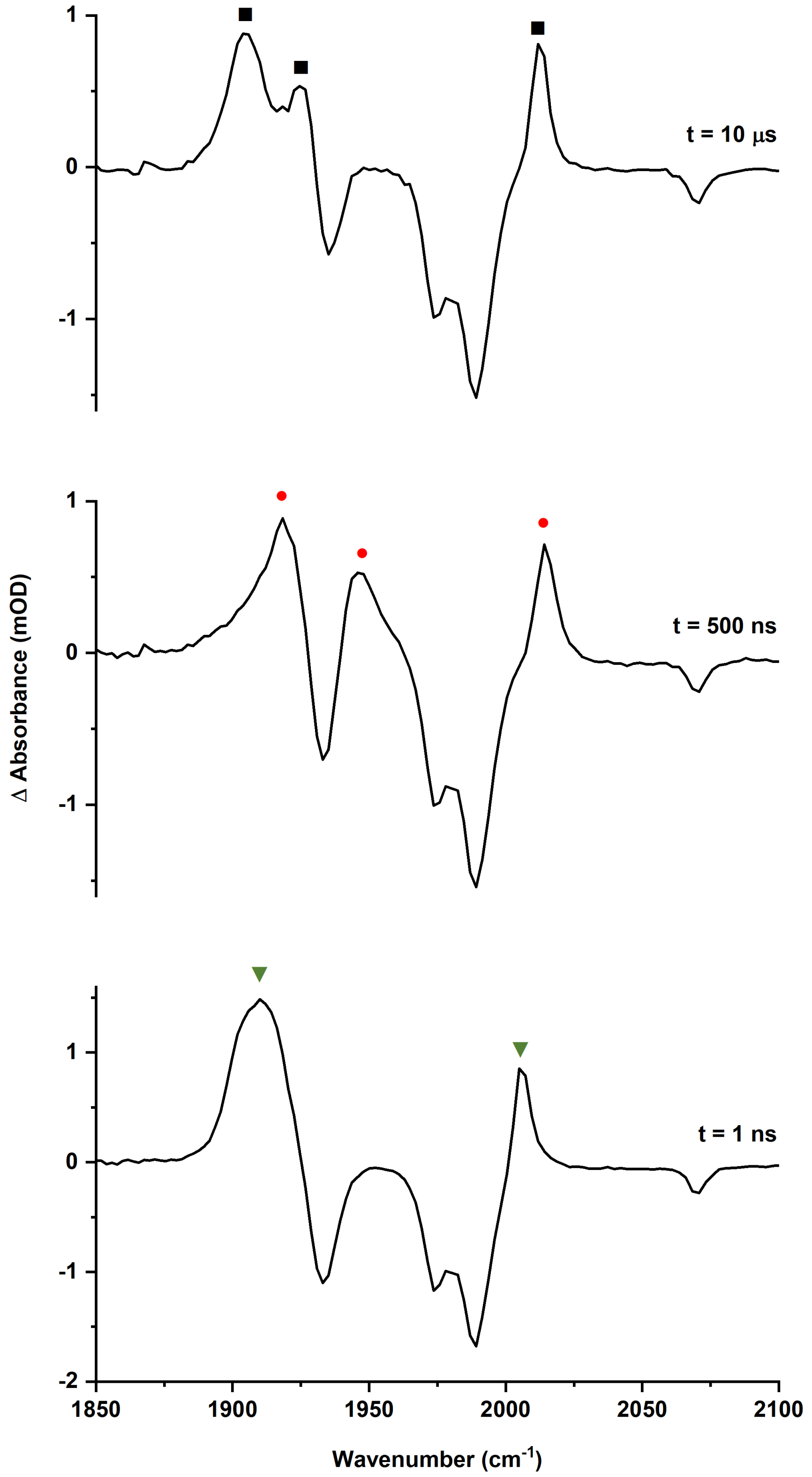

Figure S47 TRIR data for the reaction between **1b** and CF_3-4-$C_6H_4C_2H$ in toluene solution. TRIR data in the metal carbonyl region, the y-axis is change in absorbance, the negative peaks correspond to the bleach of the bands for **1b**, the positive bands show the growth and change of intermediates. Spectra with pump-probe delays of 1 ns, 500 ns, 10 μs are shown. Peaks labels are assigned to **6b** (green triangles), **2bg** (red circles) and **3bg** (black squares).

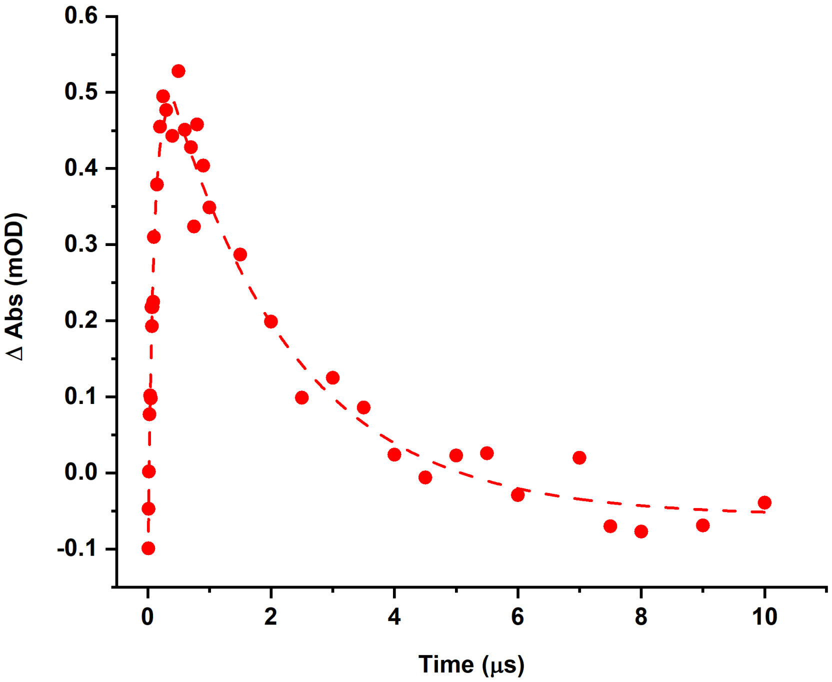

Figure S48 Kinetic plot for the reaction of **1b** in toluene with CF_3-4-$C_6H_5C_2H$. The dashed line is a fit to an exponential growth and decay function with rate constants k_g = (11.72 ± 3.30) × 10^6 s^{-1} and k_d = (4.86 ± 1.37) × 10^5 s^{-1} for the growth and decay phases respectively (R^2 = 0.964) corresponding to the formation and loss of **2eg**. The kinetic analysis was performed using data between t = 10 ns and 10 μs using the intensity changes at 1946 cm^{-1}.

2.5.5 TRIR study of **1b** and F-4-$C_6H_4C_2H$ in toluene solution

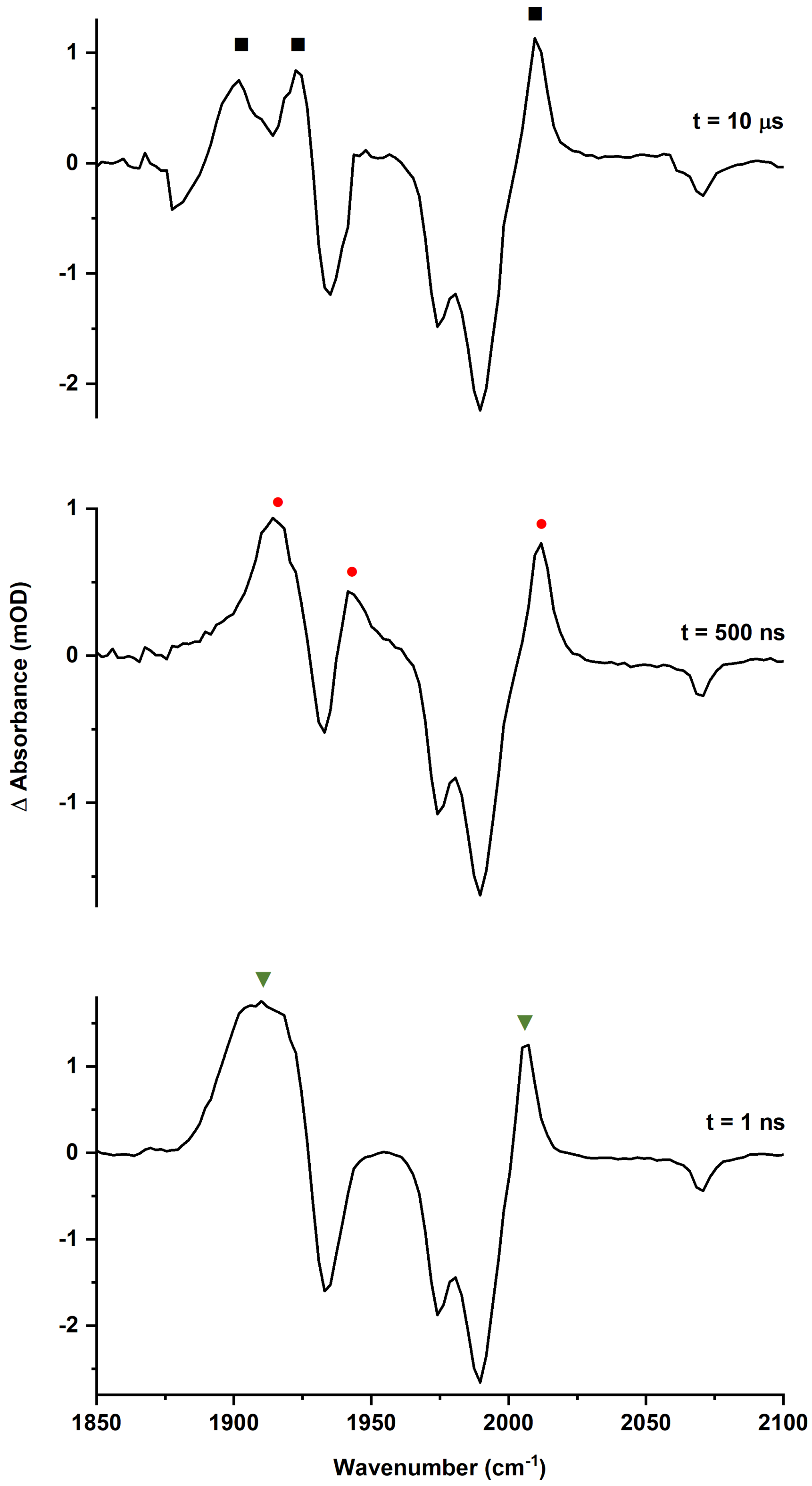

Figure S49 TRIR data for the reaction between **1b** and F-4-$C_6H_4C_2H$ in toluene solution. TRIR data in the metal carbonyl region, the y-axis is change in absorbance, the negative peaks correspond to the bleach of the bands for **1b**, the positive bands show the growth and change of intermediates. Spectra with pump-probe delays of 1 ns, 500 ns, 10 μs are shown. Peaks labels are assigned to **6b** (green triangles), **2bf** (red circles) and **3bf** (black squares).

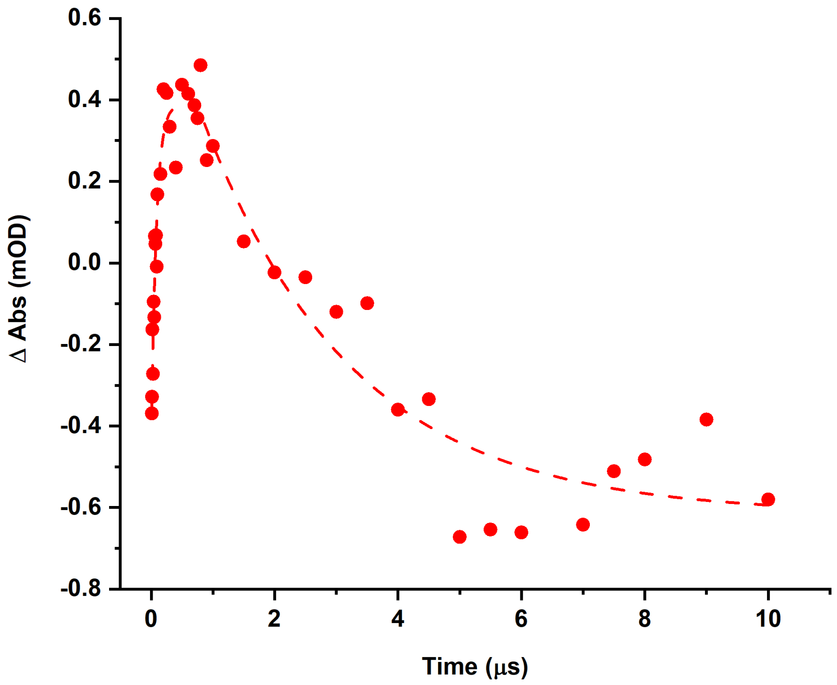

Figure S50 Kinetic plot for the reaction of **1b** in toluene with F-4-$C_6H_5C_2H$. The dashed line is a fit to an exponential growth and decay function with rate constants k_g = (12.07 ± 5.71) × 10^6 s^{-1} and k_d = (4.10 ± 1.74) × 10^5 s^{-1} for the growth and decay phases respectively (R^2 = 0.920) corresponding to the formation and loss of **2ef**. The kinetic analysis was performed using data between t = 10 ns and 10 μs using the intensity changes at 1942 cm^{-1}.

2.5.7 TRIR study of **1b** and MeO-4-$C_6H_4C_2H$ in toluene solution

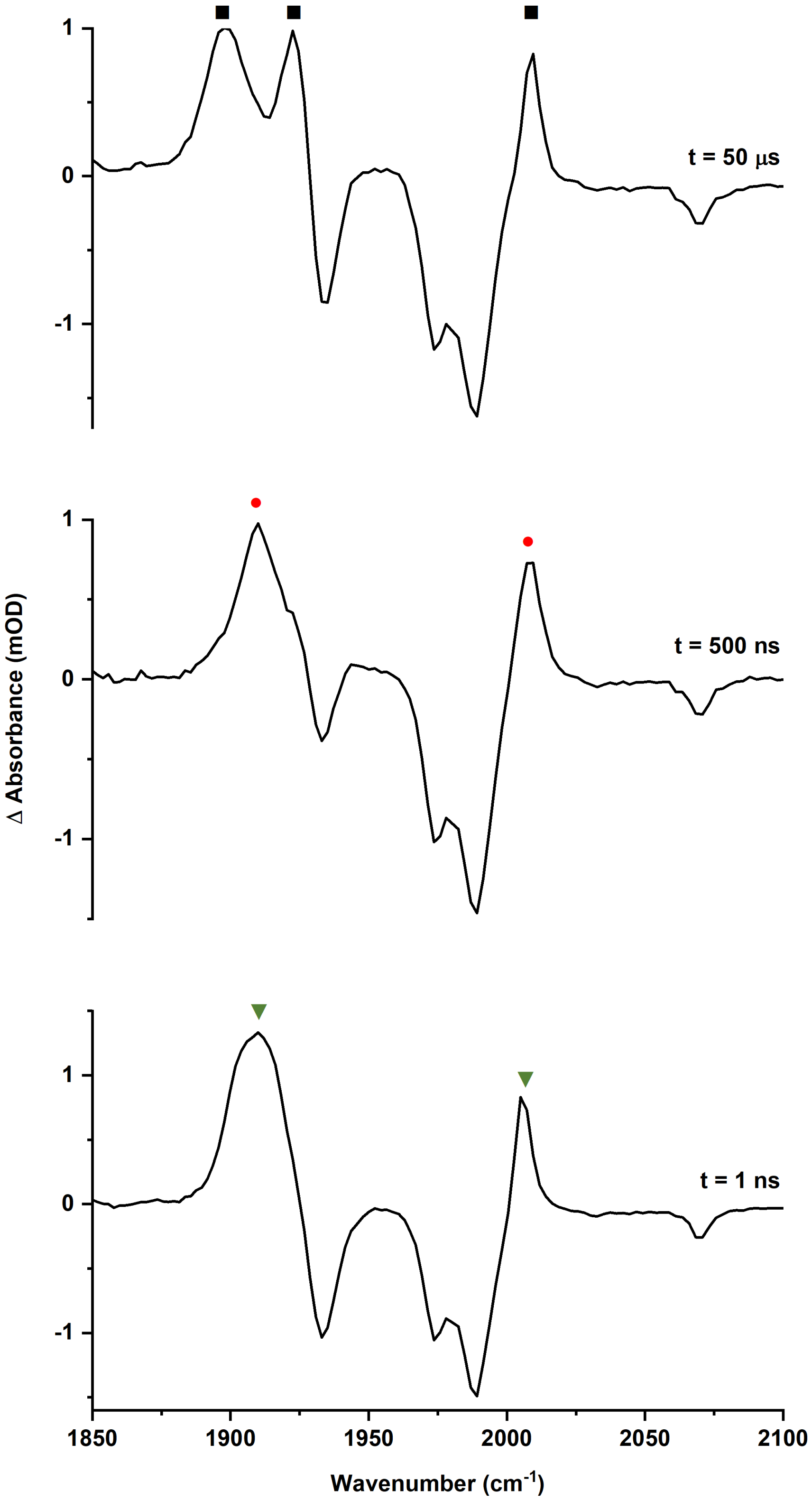

Figure S51 TRIR data for the reaction between **1b** and F-4-$C_6H_4C_2H$ in toluene solution. TRIR data in the metal carbonyl region, the y-axis is change in absorbance, the negative peaks correspond to the bleach of the bands for **1b**, the positive bands show the growth and change of intermediates. Spectra with pump-probe delays of 1 ns, 500 ns, 10 μs are shown. Peaks labels are assigned to **6b** (green triangles), **2be** (red circles) and **3be** (black squares).

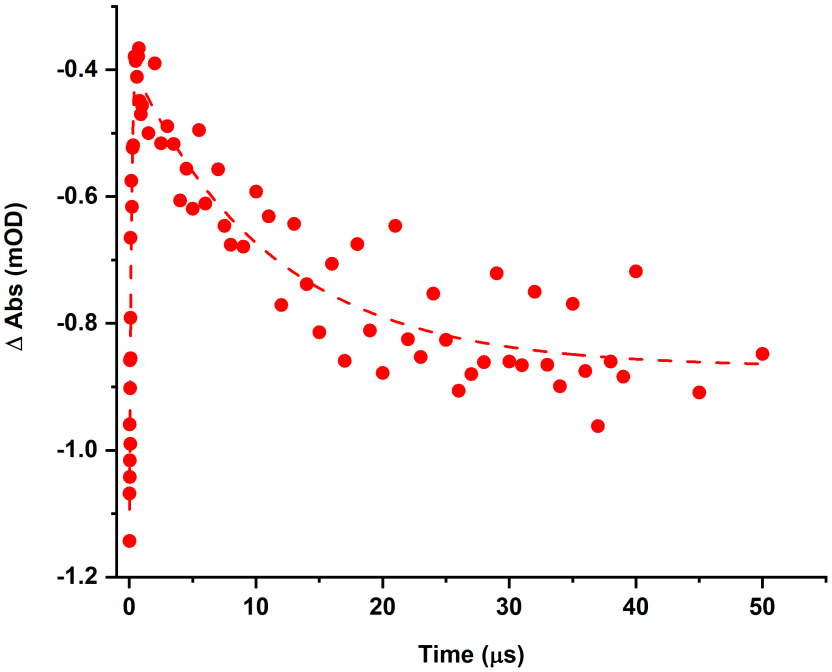

Figure S52 Kinetic plot for the reaction of **1b** in toluene with MeO-4-$C_6H_5C_2H$. The dashed line is a fit to an exponential growth and decay function with rate constants k_g = (6.36 ± 3.79) × 10^6 s^{-1} and k_d = (0.91 ± 0.32) × 10^5 s^{-1} for the growth and decay phases respectively (R^2 = 0.883) corresponding to the formation and loss of **2ea**. The kinetic analysis was performed using data between t = 10 ns and 50 μs using the intensity changes at 1933 cm^{-1}.

2.5.8 TRIR study of **1b** and $MeCO_2$-4-$C_6H_4C_2H$ in toluene solution

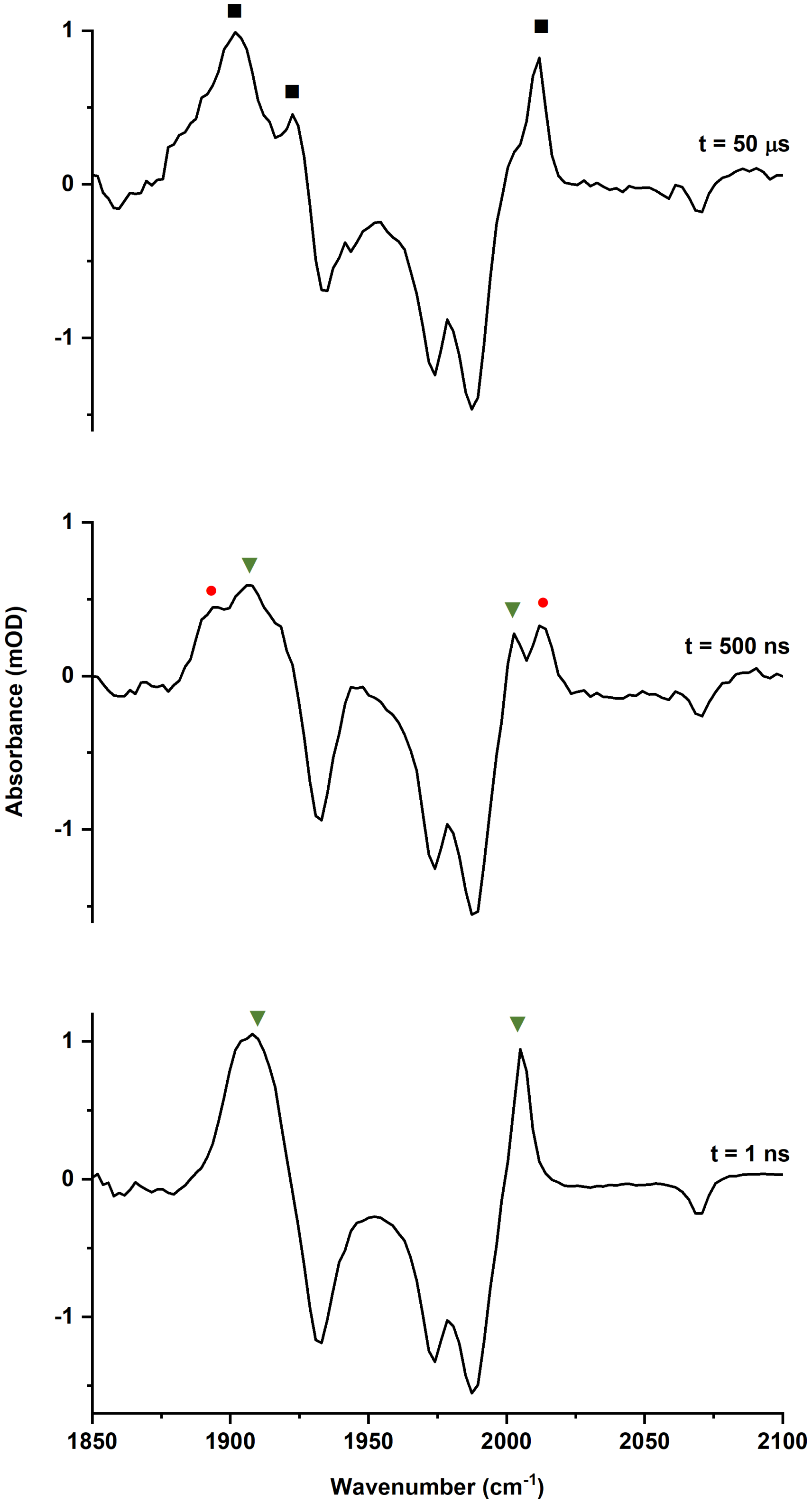

Figure S53 TRIR data for the reaction between **1b** and $MeCO_2$-4-$C_6H_4C_2H$ in toluene solution. TRIR data in the metal carbonyl region, the y-axis is change in absorbance, the negative peaks correspond to the bleach of the bands for **1b**, the positive bands show the growth and change of intermediates. Spectra with pump-probe delays of 1 ns, 500 ns, 50 ms are shown. Peaks labels are assigned to **7b** (green triangle), **2bh** (red circles) and **3bh** (black squares).

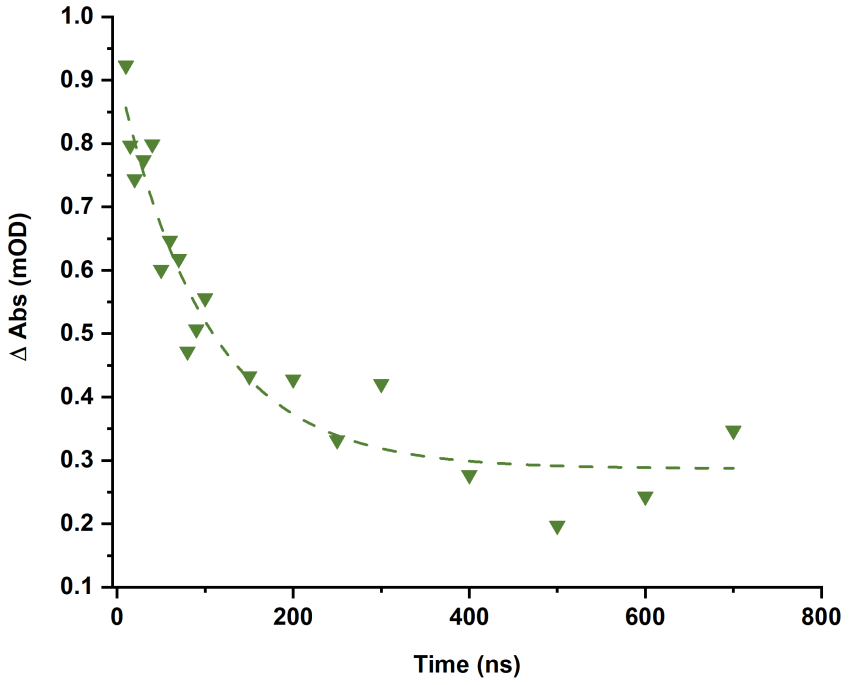

Figure S54 Kinetic plot for the reaction of **1b** in toluene solutions with $MeCO_2$-4-$C_6H_5C_2H$. The dashed line is a fit to an exponential decay function with rate constant $k = (9.95 \pm 4.3) \times 10^6\ s^{-1}$ ($R^2 = 0.910$) corresponding to the loss of **6b**. The kinetic analysis was performed using data between t = 10 ns and 700 ns using the intensity changes at 2005 cm^{-1}.

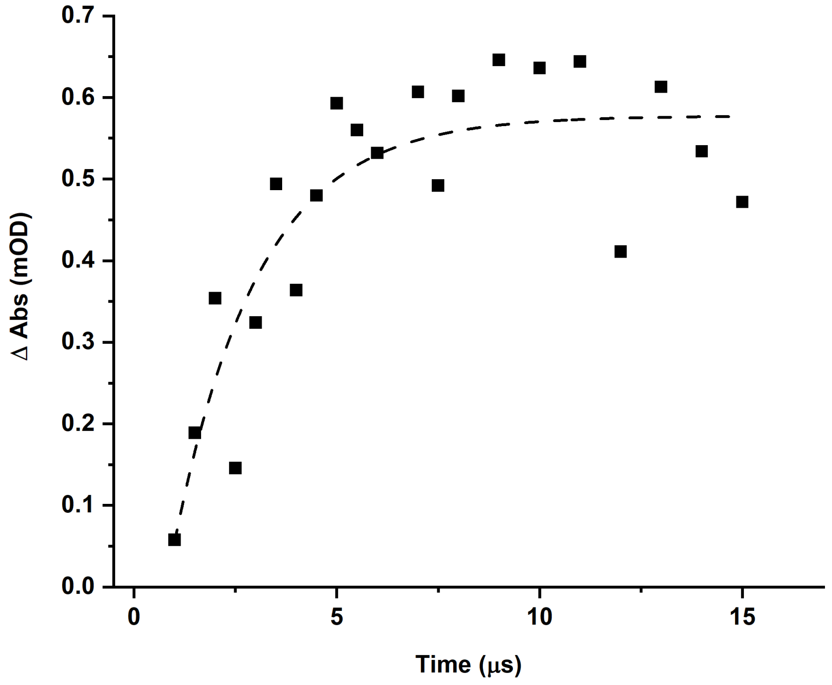

Figure S55 Kinetic plot for the reaction of **1b** in toluene solutions with $MeCO_2$-4-$C_6H_5C_2H$. The dashed line is a fit to an exponential growth function with rate constant $k = (4.79 \pm 2.90) \times 10^6\ s^{-1}$ ($R^2 = 0.743$) corresponding to the gain of **3bh**. The kinetic analysis was performed using data between t = 1 μs and 15 μs using the intensity changes at 1922 cm^{-1}.

2.5.9 TRIR study of **1b** and Me_2N-4-$C_6H_4C_2H$ in toluene solution

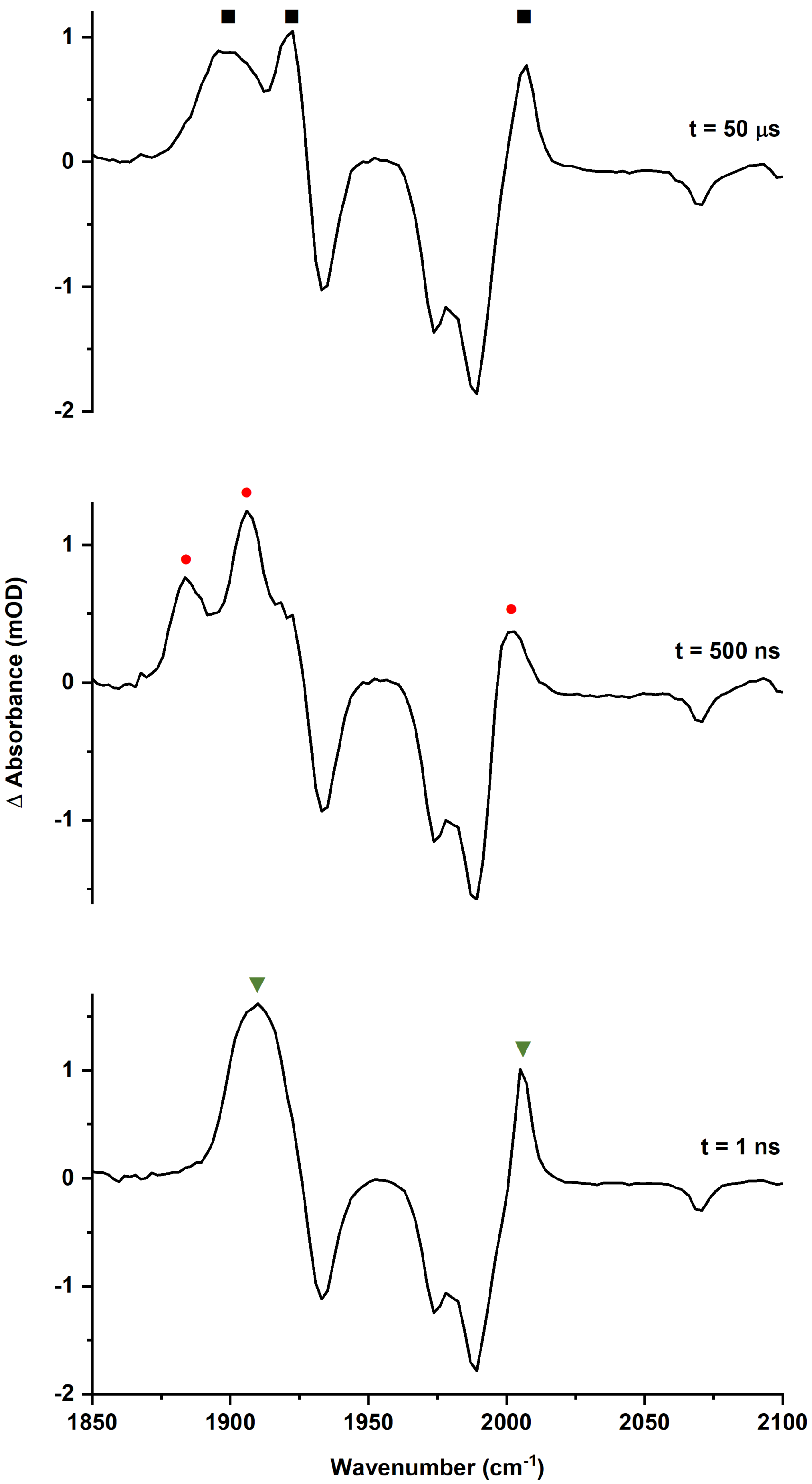

Figure S56 TRIR data for the reaction between **1b** and Me_2N-4-$C_6H_4C_2H$ in toluene solution. TRIR data in the metal carbonyl region, the y-axis is change in absorbance, the negative peaks correspond to the bleach of the bands for **1b**, the positive bands show the growth and change of intermediates. Spectra with pump-probe delays of 1 ns, 500 ns, 50 μs are shown. Peaks labels are assigned to **6b** (green triangles), **2bd** (red circles) and **3bd** (black squares).

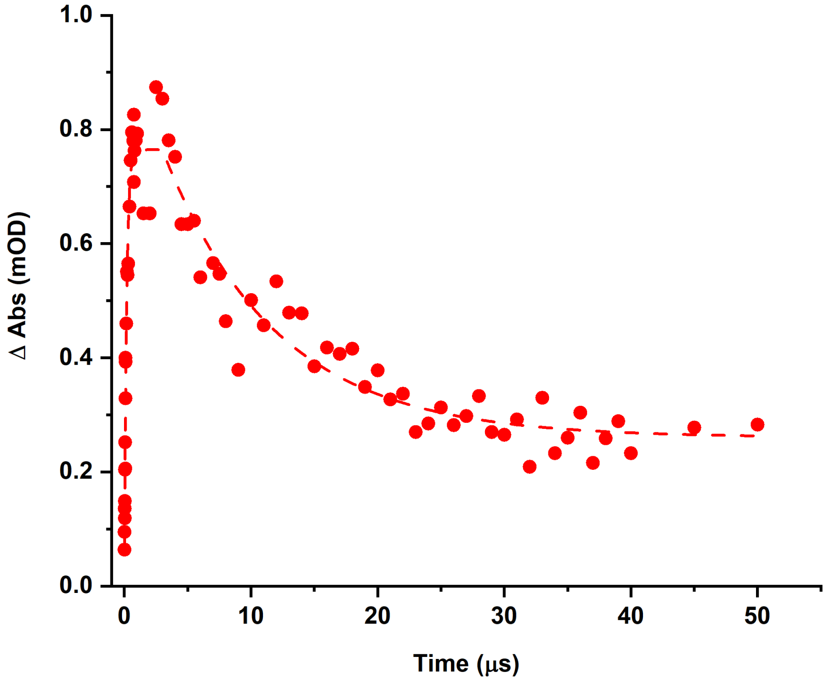

Figure S57 Kinetic plot for the reaction of **1b** in toluene with Me_2N-4-$C_6H_5C_2H$. The dashed line is a fit to an exponential growth and decay function with rate constants k_g = (5.76 ± 1.33) × 10^6 s^{-1} and k_d = (1.11 ± 0.31) × 10^5 s^{-1} for the growth and decay phases respectively (R^2 = 0.942) corresponding to the formation and loss of **2ea**. The kinetic analysis was performed using data between t = 10 ns and 50 μs using the intensity changes at 1884 cm^{-1}.

2.5.10 TRIR study of **1e** and $^{n}BuC_2{}^{n}Bu$ in toluene solution

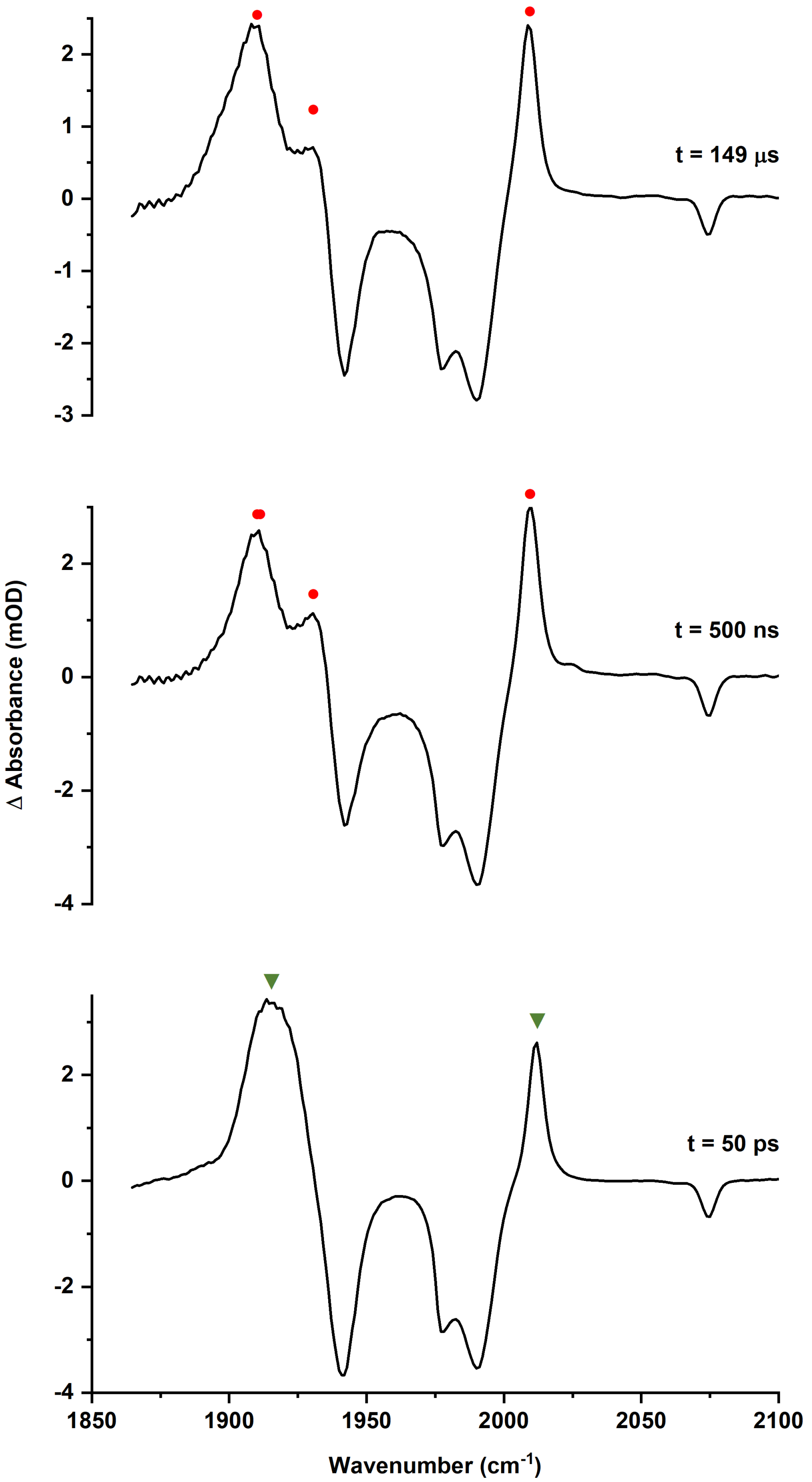

Figure S58 TRIR data for the reaction between **1e** and $^{n}BuC_2{}^{n}Bu$ in toluene solution. TRIR data in the metal carbonyl region, the y-axis is change in absorbance, the negative peaks correspond to the bleach of the bands for **1e**, the positive bands show the growth and change of intermediates. Spectra with pump-probe delays of 50 ps, 500 ns, 149 μs are shown. Peaks labels are assigned to **6e** (green triangles) and **2fj** (red circles).

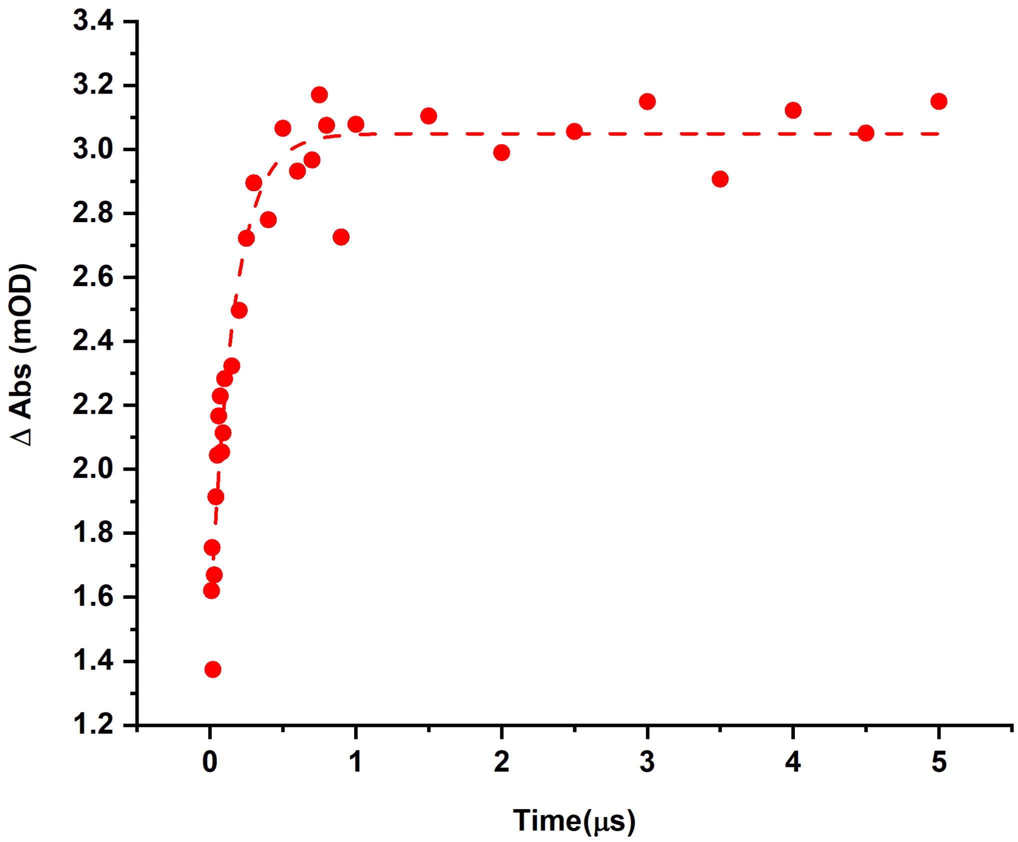

Figure S59 Kinetic plot for the reaction of **1e** in toluene with PhC_2H. The dashed line is a fit to an exponential growth function with rate constant k = (61.7 ± 1.7) × 10^6 s^{-1} (R^2 = 0.946) for the gain of **2ej**. The kinetic analysis was performed using data between t = 10 ns and 5 μs using the intensity changes at 2009 cm^{-1}

2.5.11 TRIR study of **1f** and PhC_2H in toluene solution

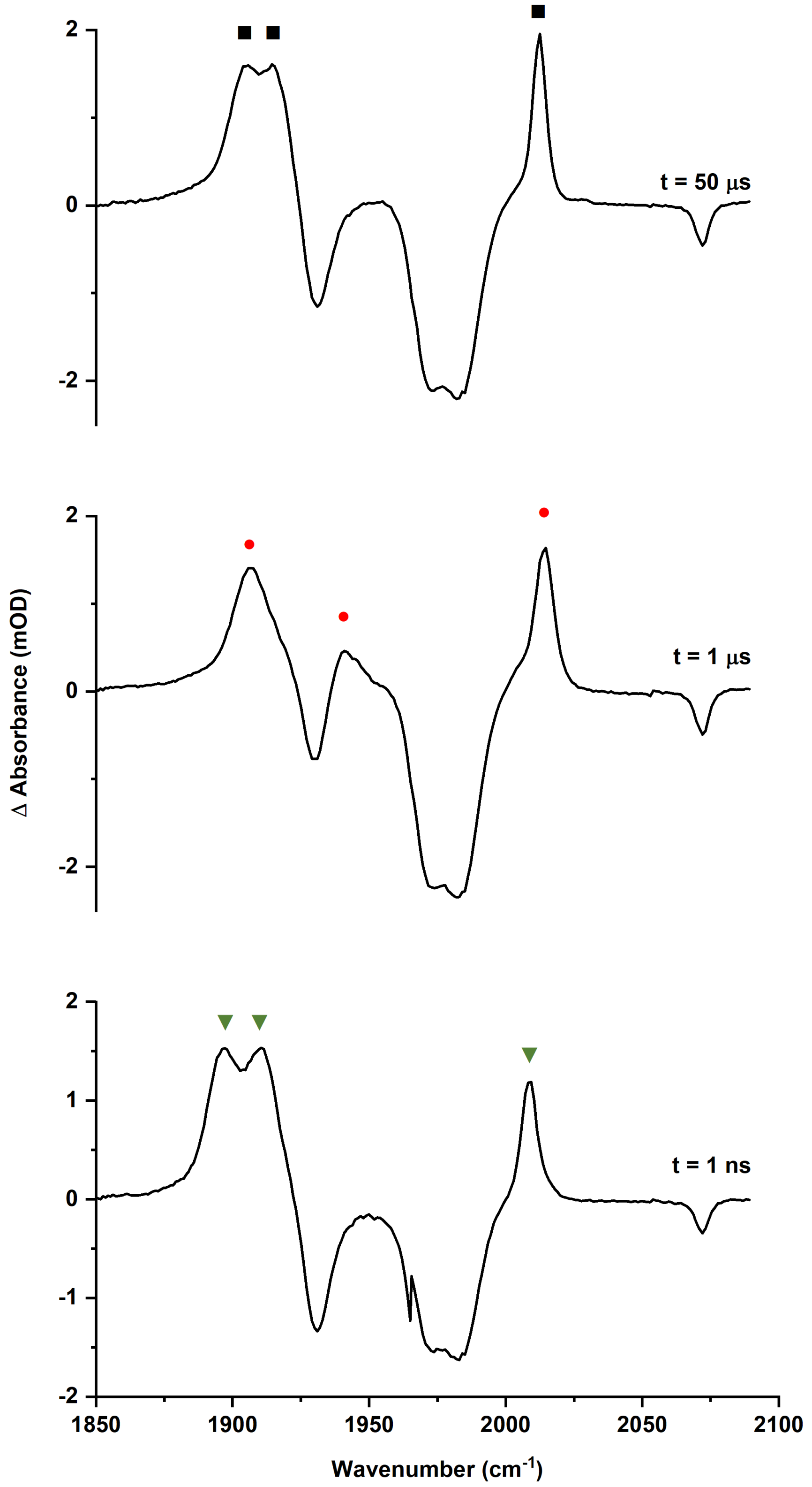

Figure S60 TRIR data for the reaction between **1f** and PhC_2H in toluene solution. TRIR data in the metal carbonyl region, the y-axis is change in absorbance, the negative peaks correspond to the bleach of the bands for **1f**, the positive bands show the growth and change of intermediates. Spectra with pump-probe delays of 1 ns, 1 μs, 50 μs are shown. Peaks labels are assigned to **6f** (green triangles), **2fa** (red circles) and **3fa** (black squares).

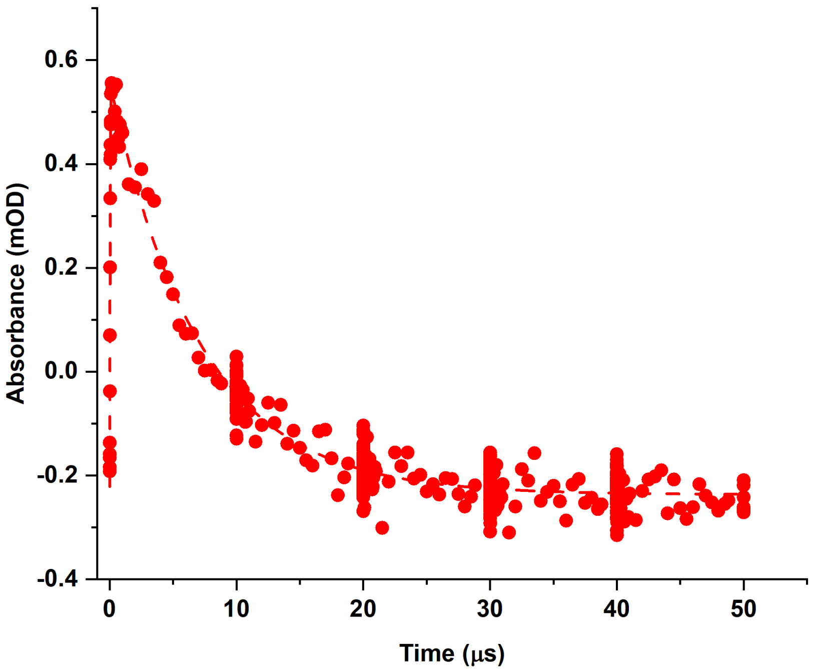

Figure S61 Kinetic plot for the reaction of **1f** in toluene with PhC_2H. The dashed line is a fit to an exponential growth and decay function with rate constants k_g = (32.4 ± 7.4) × 10^6 s^{-1} and k_d = (1.41 ± 0.05) × 10^5 s^{-1} for the growth and decay phases respectively (R^2 = 0.964) corresponding to the formation and loss of **2fa**. The kinetic analysis was performed using data between t = 5 ns and 50 μs using the intensity changes at 1941 cm^{-1}.

2.5.12 TRIR study of **1g** and PhC_2H in toluene solution

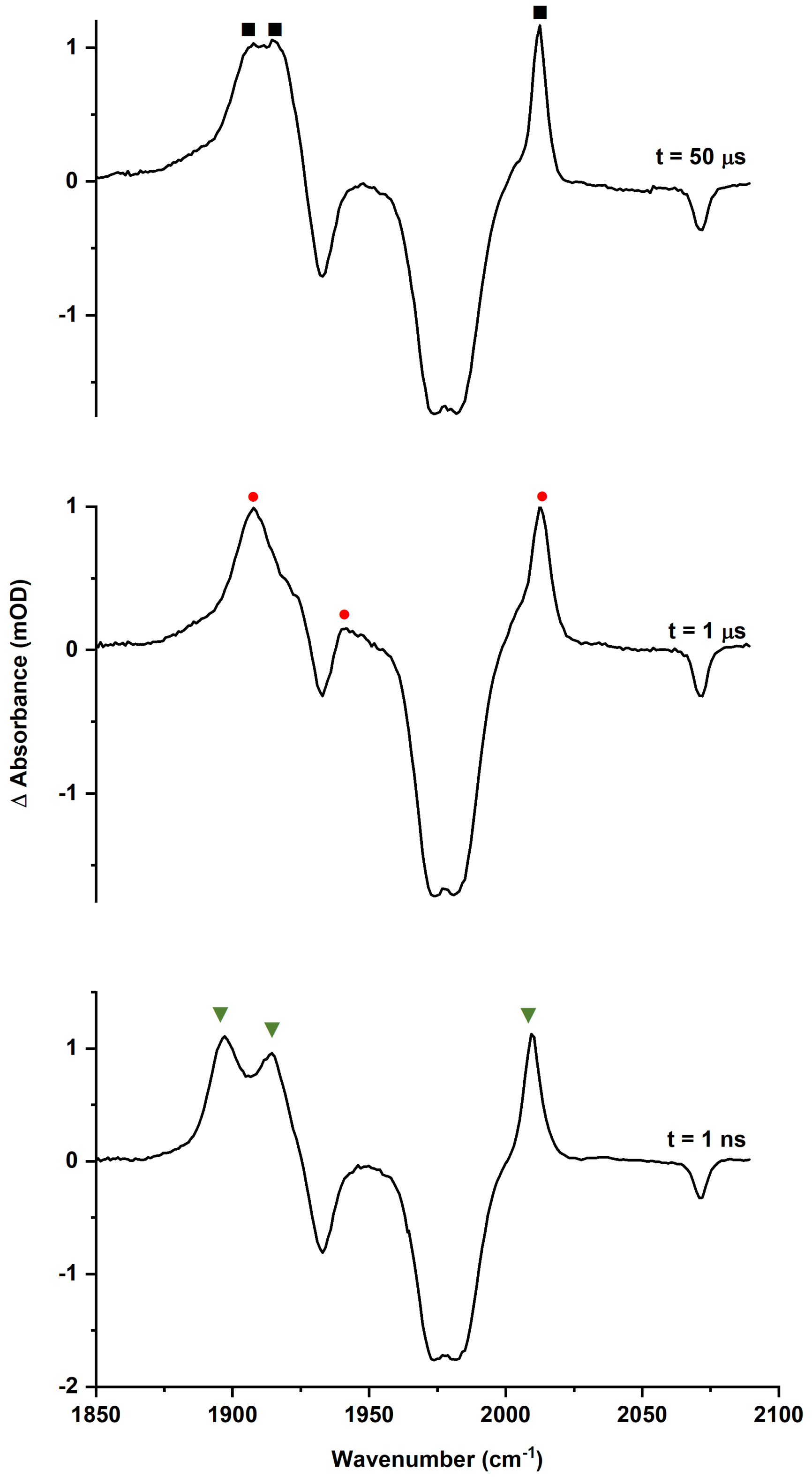

Figure S62 TRIR data for the reaction between **1g** and PhC_2H in toluene solution. TRIR data in the metal carbonyl region, the y-axis is change in absorbance, the negative peaks correspond to the bleach of the bands for **1f**, the positive bands show the growth and change of intermediates. Spectra with pump-probe delays of 1 ns, 1 μs, 50 μs are shown. Peaks labels are assigned to **6g** (green triangles), **2ga** (red circles) and **3ga** (black squares).

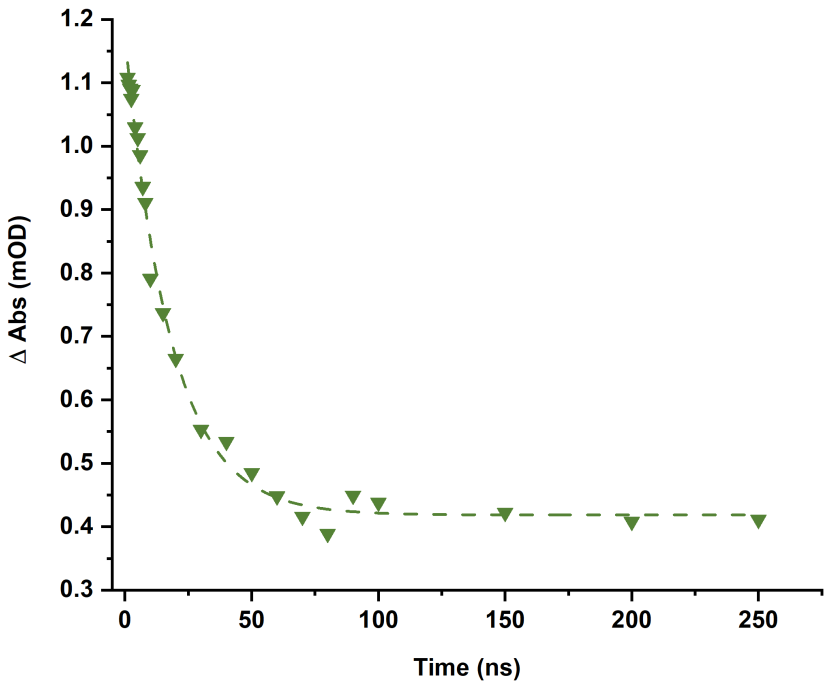

Figure S63 Kinetic plot for the reaction of **1g** in toluene with PhC_2H. The dashed line is a fit to an exponential decay function with rate constant k = (55.3 ± 6.5) × 10^6 s^{-1} (R^2 = 0.993) for the loss of **6g**. The kinetic analysis was performed using data between t = 1 ns and 250 ns using the intensity changes at 1897 cm^{-1}.

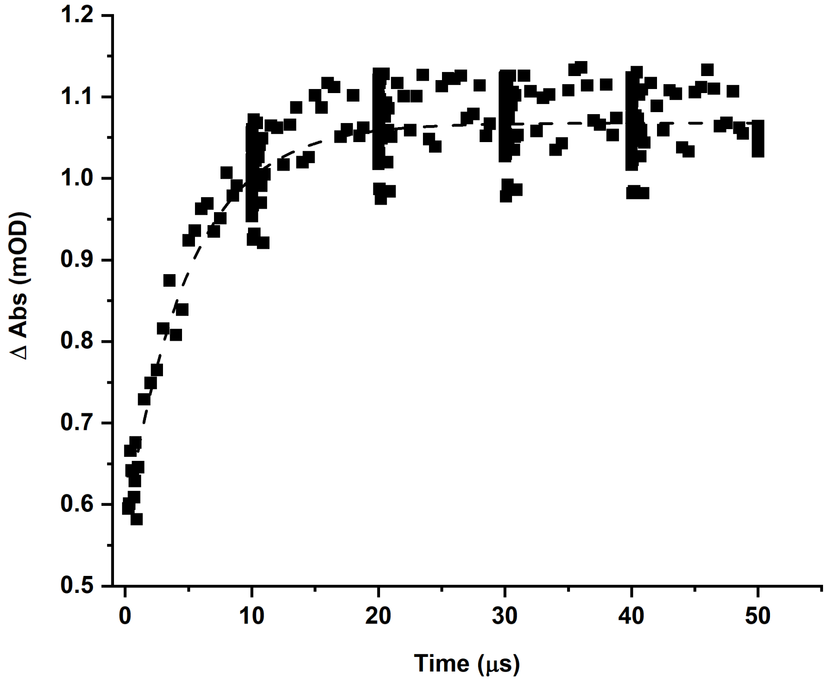

Figure S64 Kinetic plot for the reaction of **1g** in toluene with PhC_2H. The dashed line is a fit to an exponential growth function with rate constant k = (1.99 ± 0.13) × 10^5 s^{-1} (R^2 = 0.855) for the gain of **3ga**. The kinetic analysis was performed using data between t = 250 ns and 50 μs using the intensity changes at 1915 cm^{-1}.

2.5.13 TRIR study of **1h** and PhC_2H in toluene solution

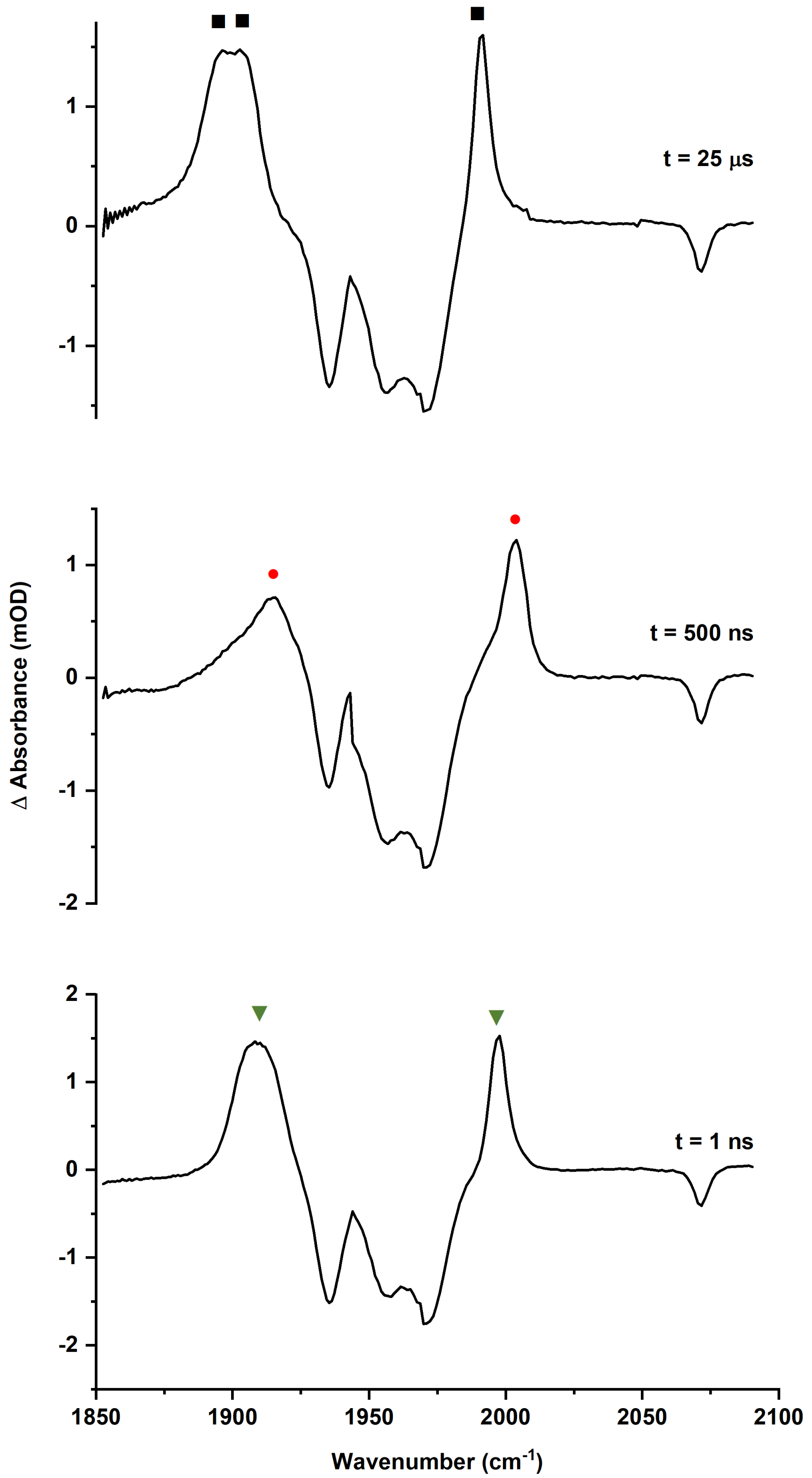

Figure S65 TRIR data for the reaction between **1h** and PhC_2H in toluene solution. TRIR data in the metal carbonyl region, the y-axis is change in absorbance, the negative peaks correspond to the bleach of the bands for **1f**, the positive bands show the growth and change of intermediates. Spectra with pump-probe delays of 1 ns, 500 ns, 25 μs are shown. Peaks labels are assigned to **6h** (green triangles), **2ha** (red circles) and **3ha** (black squares).

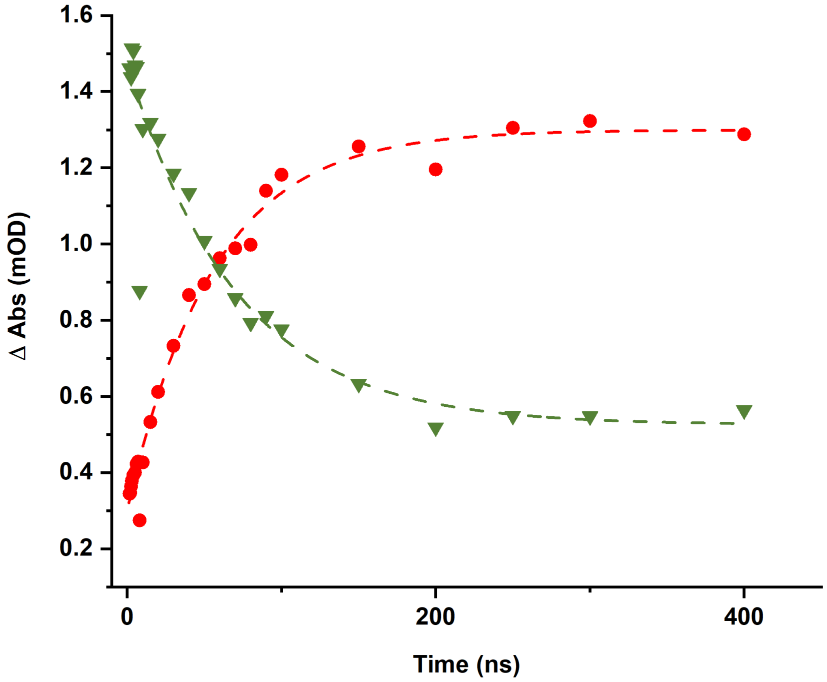

Figure S66 Kinetic plot for the reaction of **1h** in toluene with PhC_2H. The loss of the toluene complex **6h** is shown as green triangles and the green dashed line is a fit to an exponential function with rate constant k = (14.2 ± 5.9) × 10^6 s^{-1} (R^2 = 0.903). The gain of the alkyne complex **2ha** is shown as red circles and the red dashed line is a fit to an exponential growth function with rate constant k = (18.0 ± 2.8) × 10^6 s^{-1} (R^2 = 0.985). The kinetic analysis was performed using data between t = 1.5 ns and 400 ns using the intensity changes at 1998 cm^{-1} (for **6h**) and 2004 cm^{-1} (for **2ha**).

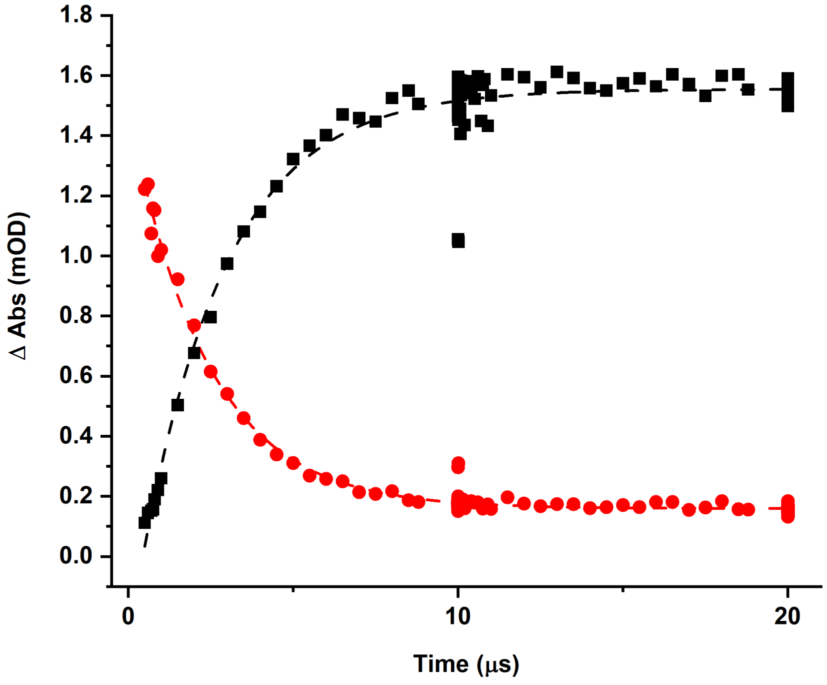

Figure S67 Kinetic plot for the reaction of **1h** in toluene with PhC_2H. The loss of the alkyne complex **3ha** is shown as red circle and the red dashed line is a fit to an exponential function with rate constant k = (4.23 ± 0.18) × 10^5 s^{-1} (R^2 = 0.991). The gain of complex **3ha** is shown as black squares circles and the black dashed line is a fit to an exponential growth function with rate constant k = (3.87 ± 0.33) × 10^5 s^{-1} (R^2 = 0.962). The kinetic analysis was performed using data between t = 500 ns and 20 μs using the intensity changes at 2004 cm^{-1} (for **2ha**) and 1991 cm^{-1} (for **3ha**).

2.5.14 TRIR study of **1i** and PhC_2H in toluene solution

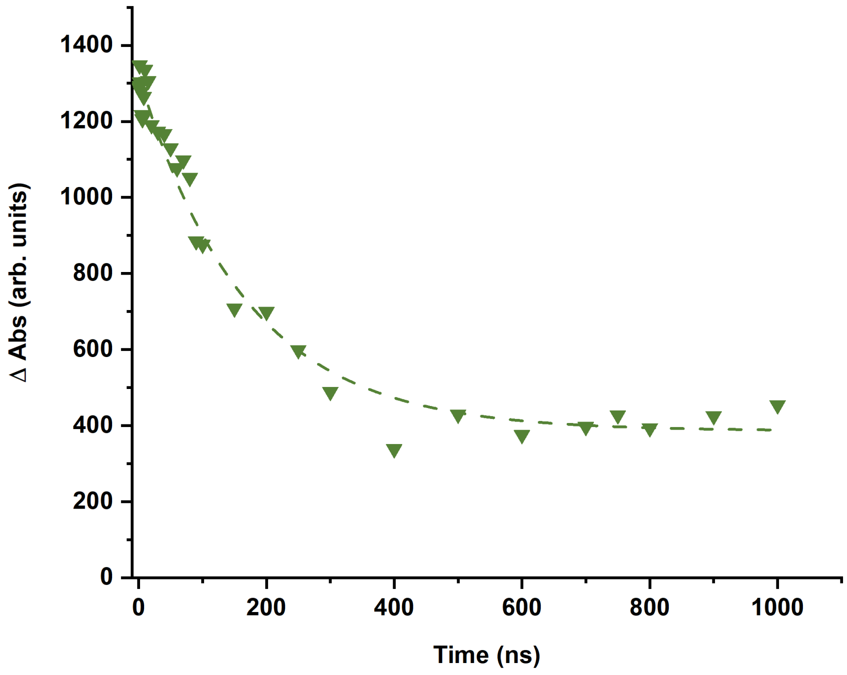

Figure S68 Kinetic plot for the reaction of **1g** in toluene solution with PhC_2H. The dashed line is a fit to an exponential decay function with rate constant k = (5.94 ± 1.01) × 10^6 s^{-1} (R^2 = 0.980) for the loss of **6i**. The kinetic analysis was performed using data between t = 1 ns and 1 μs using the intensity changes at 1903 cm^{-1}. The data have been scaled by the root-mean-square intensity of the spectrum at each pump-probe delay.

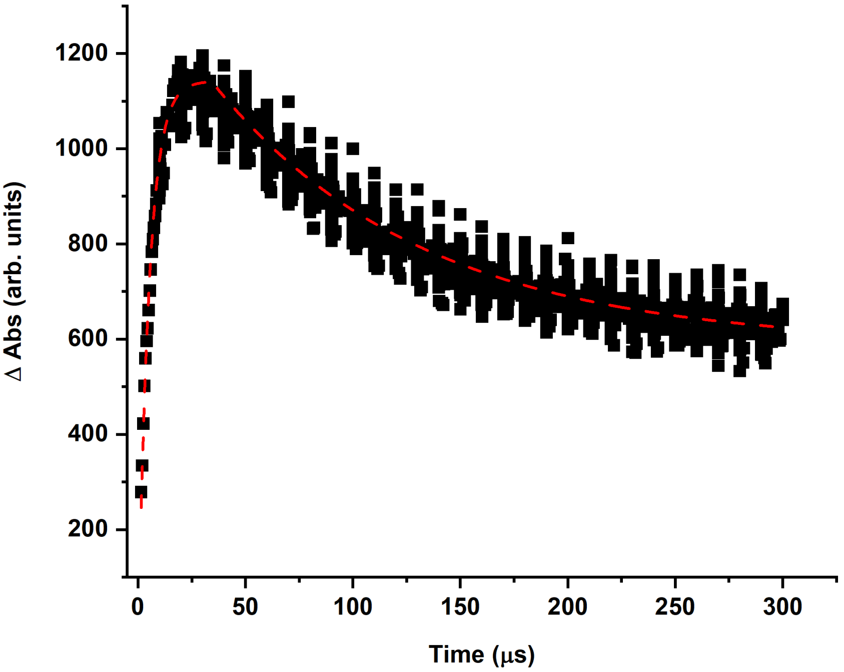

Figure S69 Kinetic plot for the reaction of **1i** in toluene solution with PhC_2H. The dashed red line is a fit to an exponential growth and decay function with k_g = (1.99 ± 0.08) × 10^5 s^{-1} and k_d = (1.02 ± 0.03) × 10^4 s^{-1} for the growth and decay phases respectively (R^2 = 0.964) corresponding to the formation and loss of **2fa**. The kinetic analysis was performed using data between t = 5 ns and 50 μs using the intensity changes at 1894 cm^{-1}. The data have been scaled by the root-mean-square intensity of the spectrum at each pump-probe delay and a six-point smooth Loess algorithm applied.

3 DFT Calculations

3.1 Methodology

Calculations to determine the relative energies of intermediates and transition states were performed using the TURBOMOLE V6.4 package using the resolution of identity (RI) approximation.[10-17] Initial optimisations were performed at the (RI-)BP86/SV(P) level, followed by frequency calculations at the same level. All minima were confirmed as such by the absence of imaginary frequencies. Single-point energies were then performed on the (RI-)BP86/SV(P) optimised geometries using the hybrid PBE0 functional and the flexible def2-TZVPP basis set. Energies, xyz coordinates and the first 50 lines of the vibrational spectra are presented. Solvation effects were modelled using COMSO[18] using the dielectronic constant of 2.38 for toluene and energies were corrected for dispertion using Grimme's D3-method with Becke-Johnson dampening.[19]

NBO calculations were performed with the NBO 7.0 software package.[20] Wavefunctions for analysis were generated at the PBE0/def2-TZVP level in Gaussian 16[21] using the (RI-)BP86/SV(P) optimised geometries. The resulting NBOs were visulalised in Multiwfn,[22] the resulting *.cub files were then exported to GaussView[23] to create the images in Figures 4 and 5 of the manuscript.

3.2 Computational analysis of the vibrational modes for complexes 8ia and 10ia

In order to aid with the assignement of the compound observed at long pump-probe delays in the experiment between **1i** and PhC_2H in toluene solution, a number of possible states were calculated and the predicated vibrational modes for the M-CO stretching modes at the bp86/SV(P) level. The calculated values were scaled using the empirical factor reported previously and compared to experimental values (Table S4).[2] The most significant finding is that the calculations predict that, in **8ai**, the higher energy symmetric stretching mode should be blue-shifted with respect to the band for **3ai**: this is observed experimentally. In contrast, the predicted band for 10ai is predicted to be red-shifted. When taken with the predicted barriers (Figure 6, main manuscript) these data support the assignement of the compound as **8ai**.

Table S4 Comparison of predicted and experimental vibrational frequencies for compounds **2ai**, **3ai**, **8ai** and **10ai**.

Compound	Scaled M-CO modes at the bp86/SV(P) level / cm^{-1}	Experimental frequency / cm^{-1}
**2ai**	1926<br>1960<br>2021	1914<br>1960<br>2023
**3ai**	1911<br>1924<br>2010	1984<br>1906<br>2010
**8ai**	1923<br>1942<br>2023	1912<br><br>2013
**9ai**	1917<br>1942<br>2012	
**10ai**	1904<br>1912<br>1990	N. A.

3.3 Computational analysis of the formation of 8ja and 10ja

In the main manuscript it was shown that complex **3ia** undergoes an additional reaction which was assigned to the formation of indenoate product **8ia**. This was based on the basis of the predicted vibrational frequencies (Section 3.2) and also the fact that the transition state to form this compound (a five-membered ring) was located at a lower energy to that for the formation of **10ia** (a six-membered ring). To provide further support for this assignment a reaction profile that was shown to produce the six-membered product was modelled.

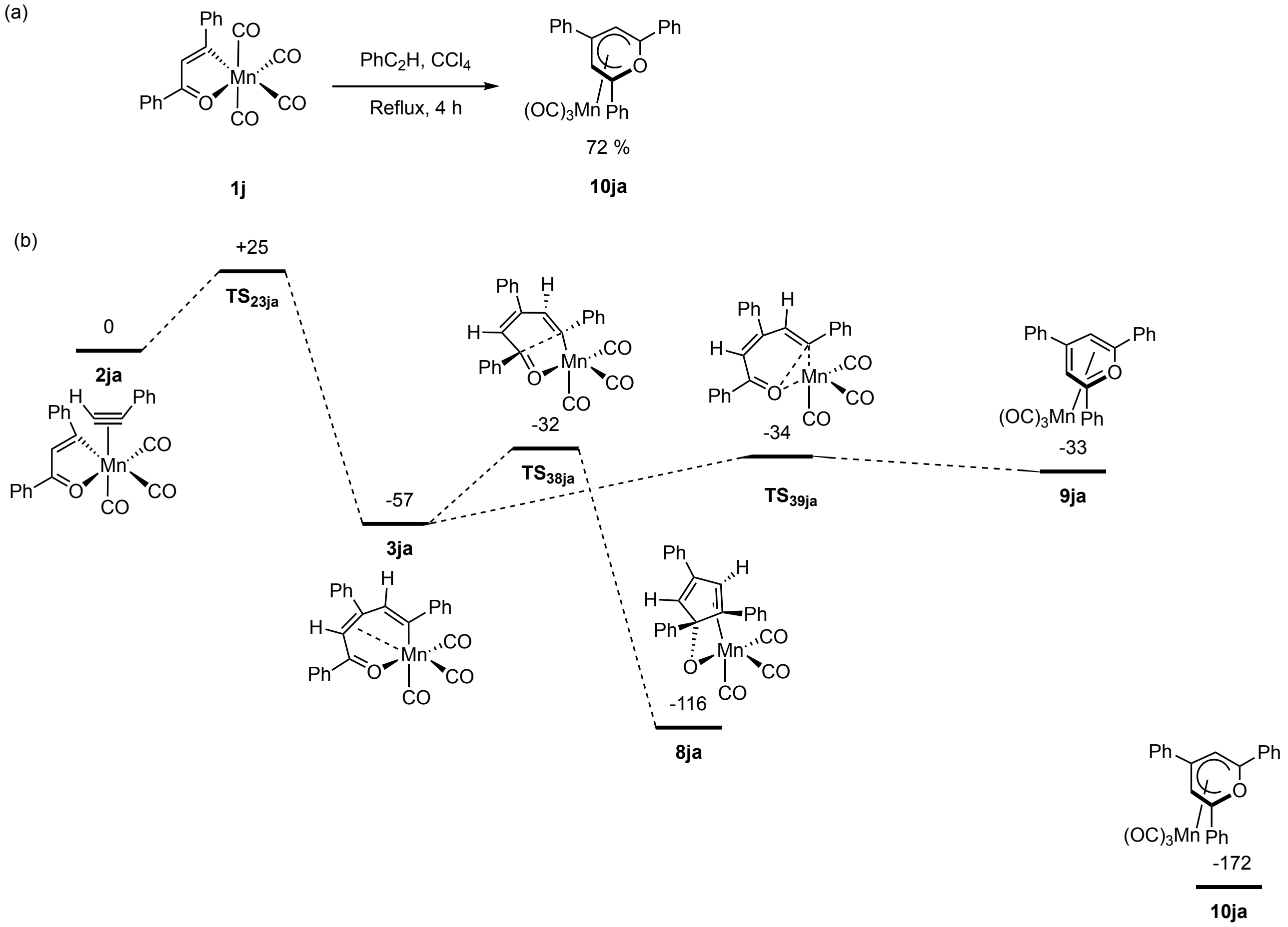

Figure S70 DFT-calculated pathways for Mn-promoted C–C bond formation from **2ja**. Energies are Free Energies at 298 K in kJ mol^{-1} at the D3-pbe0/def2-TZVPP//bp86/SV(P) level with COSMO solvation in toluene.

It has been demonstrated experimentally that the reaction of a cyclomanganated chalcone complex, **1j**, with PhC_2H results in the formation of the pyrillium product, **10ja** (Figure S70a).[24] Therefore the potential formation of **8ja** and **10ja** was modelled in an identical fashion to **8ia** and **10ia** in the main manuscript (Figure 70b). In this case it was found that the difference in energy between the transition states leading for the formation of the pyrillium (**TS$_{39ja}$**, -34 kJ mol^{-1}) and indenoate products (**TS$_{38ja}$**, -32 kJ mol^{-1}) is far smaller. This difference in energy was surveyed at at several different levels of theory (Table S5) and it was found that at all levels **TS$_{39ja}$** was the lower energy pathway, consistent with the experimentally observed formation of **10ja**, albeit at D3-pbe0/def2-TZVPP//bp86/SV(P) the difference is very small. In the case of **3ia**, which on the

basis of experimental data is expected to give the indenoate complex **8ai**, then **TS$_{38ia}$**,[25, 26] is the lower energy transition state, consistent with the formation of a five membered ring. These data therefore support assignment of the transient species in the manuscript to **8ia**.

Table S5 Comparison of energies for the formation of five- and six-memebered products from cyclomanganated complexes **3ia** and **3ja**. Energies are in kJ mol^{-1} and are relative to the corresponding alkyne complexes **2**, ΔE are zero-point energy-corrected electronic energies and ΔG are Gibbs energies.

**Computational level**	**TS$_{38ia}$**	**TS$_{39ia}$**	**TS$_{38ja}$**	**TS$_{38ja}$**
bp86/sv(p) ΔG	-11	0	-10	-47
bp86/sv(p) ΔE	-19	-5	-12	-51
pbe0/def2-TZVPP//bp86/SV(P) ΔG	-28	14	-21	-36
pbe0/def2-TZVPP//bp86/SV(P) ΔE	-36	9	-22	-40
pbe0/def2-TZVPP//bp86/SV(P) COSMO ΔG	-29	15	-25	-37
pbe0/def2-TZVPP//bp86/SV(P) COSMO ΔE	-37	10	-26	-40
D3-pbe0/def2-TZVPP//bp86/SV(P) COSMO ΔG	-37	17	-32	-34
D3-pbe0/def2-TZVPP//bp86/SV(P) COSMO ΔE	-45	12	-33	-38

4 References

1. R. He, Z.-T. Huang, Q.-Y. Zheng and C. Wang, *Angew. Chem. Int. Ed.*, 2014, **53**, 4950-4953.
2. L. A. Hammarback, I. P. Clark, I. V. Sazanovich, M. Towrie, A. Robinson, F. Clarke, S. Meyer, I. J. S. Fairlamb and J. M. Lynam, *Nat. Catal.*, 2018, **1**, 830-840.
3. W. C. Still, M. Kahn and A. Mitra, *The Journal of Organic Chemistry*, 1978, **43**, 2923-2925.
4. J. S. Ward, J. M. Lynam, J. W. B. Moir, D. E. Sanin, A. P. Mountford and I. J. S. Fairlamb, *Dalton Trans.*, 2012, **41**, 10514-10517.
5. J. Jin, Q. Wen, P. Lu and Y. Wang, *Chem. Commun.*, 2012, **48**, 9933-9935.
6. G. M. Greetham, P. M. Donaldson, C. Nation, I. V. Sazanovich, I. P. Clark, D. J. Shaw, A. W. Parker and M. Towrie, *Appl. Spectrosc.*, 2016, **70**, 645-653.
7. ULTRA View Data Analysis, M. R. Pollard and G. M. Greetham, STFC.
8. OriginPro, 2019, OriginLab Corporation, Northampton, MA, USA.
9. G. M. Greetham, D. Sole, I. P. Clark, A. W. Parker, M. R. Pollard and M. Towrie, *Rev. Sci. Instrum.*, 2012, **83**, 103107.
10. P. Császár and P. Pulay, *J. Mol. Struct.*, 1984, **114**, 31-34.
11. R. Ahlrichs, M. Bär, M. Häser, H. Horn and C. Kölmel, *Chem. Phys. Lett.*, 1989, **162**, 165-169.
12. P. Deglmann, F. Furche and R. Ahlrichs, *Chem. Phys. Lett.*, 2002, **362**, 511-518.
13. P. Deglmann, K. May, F. Furche and R. Ahlrichs, *Chem. Phys. Lett.*, 2004, **384**, 103-107.
14. K. Eichkorn, O. Treutler, H. Öhm, M. Häser and R. Ahlrichs, *Chem. Phys. Lett.*, 1995, **242**, 652-660.
15. K. Eichkorn, F. Weigend, O. Treutler and R. Ahlrichs, *Theor. Chem. Acc.*, 1997, **97**, 119-124.
16. O. Treutler and R. Ahlrichs, *J. Chem. Phys.*, 1995, **102**, 346-354.
17. M. von Arnim and R. Ahlrichs, *J. Chem. Phys.*, 1999, **111**, 9183-9190.
18. A. Klamt and G. Schuurmann, *J. Chem. Soc., Perkin Trans. 2*, 1993, 799-805.
19. S. Q. Niu, S. Zaric, C. A. Bayse, D. L. Strout and M. B. Hall, *Organometallics*, 1998, **17**, 5139-5147.
20. NBO 7.0, E. D. Glendening, J. K. Badenhoop, A. E. Reed, J. E. Carpenter, J. A. Bohmann, C. M. Morales, P. Karafiloglou, C. R. Landis and F. Weinhold, Theoretical Chemistry Institute, University of Wisconsin, Madison,
21. Gaussian 16 Rev. A.03, M. J. Frisch, G. W. Trucks, H. B. Schlegel, G. E. Scuseria, M. A. Robb, J. R. Cheeseman, G. Scalmani, V. Barone, G. A. Petersson, H. Nakatsuji, X. Li, M. Caricato, A. V. Marenich, J. Bloino, B. G. Janesko, R. Gomperts, B. Mennucci, H. P. Hratchian, J. V. Ortiz, A. F. Izmaylov, J. L. Sonnenberg, Williams, F. Ding, F. Lipparini, F. Egidi, J. Goings, B. Peng, A. Petrone, T. Henderson, D. Ranasinghe, V. G. Zakrzewski, J. Gao, N. Rega, G. Zheng, W. Liang, M. Hada, M. Ehara, K. Toyota, R. Fukuda, J. Hasegawa, M. Ishida, T. Nakajima, Y. Honda, O. Kitao, H. Nakai, T. Vreven, K. Throssell, J. A. Montgomery Jr., J. E. Peralta, F. Ogliaro, M. J. Bearpark, J. J. Heyd, E. N. Brothers, K. N. Kudin, V. N. Staroverov, T. A. Keith, R. Kobayashi, J. Normand, K. Raghavachari, A. P. Rendell, J. C. Burant, S. S. Iyengar, J. Tomasi, M. Cossi, J. M. Millam, M. Klene, C. Adamo, R. Cammi, J. W. Ochterski, R. L. Martin, K. Morokuma, O. Farkas, J. B. Foresman and D. J. Fox, Wallingford, CT
22. T. Lu and F. W. Chen, *J. Comput. Chem.*, 2012, **33**, 580-592.
23. GaussView, Version 6.1, R. Dennington, T. A. Keith and J. M. Millam, Semichem Inc., Shawnee Mission, KS, 2016.,
24. W. Tully, L. Main and B. K. Nicholson, *J. Organomet. Chem.*, 1996, **507**, 103-115.
25. N. P. Robinson, L. Main and B. K. Nicholson, *J. Organomet. Chem.*, 1989, **364**, C37-C39.
26. L. S. Liebeskind, J. R. Gasdaska, J. S. Mccallum and S. J. Tremont, *J. Org. Chem.*, 1989, **54**, 669-677.
